# Comparative clinical and genomic analysis of neurofibromatosis type 2-associated cranial and spinal meningiomas

**DOI:** 10.1038/s41598-020-69074-z

**Published:** 2020-07-28

**Authors:** Alexander Pemov, Ramita Dewan, Nancy F. Hansen, Settara C. Chandrasekharappa, Abhik Ray-Chaudhury, Kristine Jones, Wen Luo, John D. Heiss, James C. Mullikin, Prashant Chittiboina, Douglas R. Stewart, Ashok R. Asthagiri

**Affiliations:** 1 Clinical Genetics Branch, Division of Cancer Epidemiology and Genetics, National Cancer Institute, NIH, Rockville, MD USA; 2 Surgical Neurology Branch, National Institute of Neurological Disorders and Stroke, NIH, Bethesda, MD USA; 3 Cancer Genetics and Comparative Genomics Branch, National Human Genome Research Institute, NIH, Bethesda, MD USA; 4 Frederick National Laboratory for Cancer Research, Division of Cancer Epidemiology and Genetics, National Cancer Institute, Rockville, MD USA; 5 NIH Intramural Sequencing Center, National Human Genome Research Institute, NIH, Rockville, MD USA; 6 Present Address: Neuromuscular Disease Research Section, National Institute On Aging, NIH, Bethesda, MD USA; 7 Present Address: Department of Neurological Surgery, University of Virginia School of Medicine, Charlottesville, VA USA

**Keywords:** Genome-wide analysis of gene expression, PCR-based techniques

## Abstract

Neurofibromatosis type 2 (NF2) is an autosomal dominant Mendelian tumor predisposition disorder caused by germline pathogenic variants in the tumor suppressor *NF2*. Meningiomas are the second most common neoplasm in NF2, often occurring in multiple intracranial and spinal locations within the same patient. In this prospective longitudinal study, we assessed volumes and growth rates of ten spinal and ten cranial benign meningiomas in seven NF2 patients that concluded with surgical resection and performed whole-exome sequencing and copy-number variant (CNV) analysis of the tumors. Our comparison of the volume and the growth rate of NF2-associated spinal and cranial meningiomas point to the differences in timing of tumor initiation and/or to the differences in tumor progression (e.g., non-linear, saltatory growth) at these two anatomical locations. Genomic investigation of these tumors revealed that somatic inactivation of *NF2* is the principal and perhaps the only driver of tumor initiation; and that tumor progression likely occurs via accumulation of CNVs, rather than point mutations. Results of this study contribute to a better understanding of NF2-associated meningiomas clinical behavior and their genetic underpinnings.

## Introduction

Neurofibromatosis type 2 (NF2) is a rare hereditary neoplasia syndrome, with an estimated incidence of 1:25,000–1:33,000^1,2^. Although the hallmark and primary definitive diagnostic criterion of NF2 is the presence of bilateral vestibular schwannomas, meningiomas are the next most frequently identified tumor in these patients^3,4^. It is estimated that 45–58% of NF2 patients harbor intracranial meningiomas and 20% have spinal meningiomas^5–7^. Meningiomas in NF2 are typically WHO grade 1, slow-growing, benign tumors. When present, meningiomas in NF2 patients are often multiple, which contributes significantly to morbidity and mortality. Although symptoms are primarily due to the direct mass effect of tumor upon adjacent brain and cranial nerves, tumors may also evoke seizures and obstruct venous outflow causing cerebral edema^6^.

A case series examining 287 cranial meningiomas from NF2 patients confirmed that similar to their sporadic counterparts, the majority of NF2-associated meningiomas (95.8%) were grade 1 by WHO guidelines. Although only 4.2% of total meningiomas were grade 2 or 3, 35% of growing or symptomatic resected meningiomas were grade 2/3. Growth rate was generally slow, although 7.3% of meningiomas examined by MRI analysis displayed an annual volumetric growth rate of 20% or higher^8^. The cumulative burden of intracranial meningiomas in NF2 patients results in a 2.51-fold greater risk of mortality when compared to NF2 patients without intracranial meningiomas^9^.

Currently, complete surgical resection is the best treatment for symptomatic meningiomas, however, this poses a challenge when parasagittal, skull base, and ventral spinal tumors are considered^3^. The complete surgical resection of a meningioma and its adjacent infiltration into the dura of the skull base or sagittal sinus carries significant surgical morbidity, so a less-morbid approach combining surgical removal of free tumor followed by stereotactic radiosurgery (SRS) of tumor-infiltrated dura and vascular structures is often used to manage these tumors in non-NF2 patients^10^. This approach may not work for NF2-associated meningiomas because of the (1) uncertainty in deciding which tumor is symptomatic when multiple discrete meningiomas in widespread intracranial locations are progressing simultaneously, (2) presence of large areas of confluent tumors, mimicking *en plaque* meningioma, and (3) radiation therapy or SRS may have greater potential to cause malignant conversion of the meningioma and surrounding tissue in the setting of an underlying genetic tumor syndrome. According to consensus recommendations for treatments in patients with NF2, radiation therapy (either stereotactic radiosurgery or intensity modulated radiation therapy) should be used with caution since secondary malignancies after radiosurgery have been reported^11^. This is highlighted by the observation that spontaneous nervous system malignancies are very rare in NF2, at a prevalence of 725 per 100,000 (95% CI 253–1,197 per 100,000). However, after radiotherapy for benign tumors, the prevalence of nervous system malignancies in NF2 patients is substantially increased, at 4,717 per 100,000 (95% CI 681–8,753 per 100,000)^12^. Radiosurgery has been utilized to treat NF2-associated tumors in less surgically accessible locations, but its safety and efficacy has not been studied in a large case series or with long-term follow up^13^.

To better understand NF2-associated meningiomas, we performed longitudinal semi-annual clinical examinations and craniospinal MRI scans in seven unrelated NF2 patients, recording clinical symptoms and measuring the tumor volume of meningiomas that ultimately became symptomatic and required surgical removal. Cranial meningiomas are generally larger than spinal tumors at the time of symptom development due to the mass that must be achieved to result in clinical sequelae. Thus, we hypothesized that cranial meningiomas grow faster than their spinal counterparts since neither is associated with an earlier age at symptom onset. Subsequent to tumor resection, we performed whole-exome sequencing (WES) and copy-number variation (CNV) analyses to evaluate whether genetic profiling of these tumors based on location would reveal differences that could be reflected in tumor growth or size. To our knowledge, this is the first WES analysis of spinal and cranial meningiomas in multiple unrelated NF2 individuals.

## Results

### Clinical characteristics

A total of ten cranial and ten spinal meningiomas from seven patients, two males and five females, were included in volumetric and genomic analyses. All tumors that were resected were symptomatic or within the immediate vicinity of a symptomatic tumor, and therefore exposed with the surgical approach, necessitating its resection. In some cases, in which multiple tumors were resected (P3_C1, P3_C2), a single tumor could not be perfectly correlated with nonspecific patient symptoms (headache). Therefore, multiple space-occupying lesions were removed, with symptom improvement noted postoperatively. All tumors had demonstrated growth in the immediate period preceding surgical resection. All patients had multiple meningiomas and the median age at time of surgery was 25 and 26 years old (y.o.) (range: 22–26 and 9–43 y.o.) for spinal and cranial tumors respectively (Table 1). The mean interval of imaging was 1.00 year (range, 0.17–1.24 years).

**Table 1:** Volume and measures of growth rate of spinal and cranial meningiomas.

Tumor characteristics	Spinal	Cranial
Median pre-operative tumor volume (cm^3^)	0.5	3.4
IQR (cm^3^)	0.3	5.7
Range (cm^3^)	0.1–2.0	0.3–10.2
*P* value, tumor volumes, spinal vs cranial (Mann–Whitney)	*0.005*	
Median absolute tumor growth rate (cm^3^/year)	0.1	0.9
IQR (cm^3^/year)	0.2	2.4
Range (cm^3^/year)	0.02–1.1	0.04–5.6
*P* value, absolute tumor growth rates, spinal vs cranial (*t* test)	*0.045*	
Median relative tumor growth rate (percent/year)	50.2	49.5
IQR (percent/year)	47.7	47.4
Range (percent/year)	10–155	18–685
*P* value, relative tumor growth rates, spinal vs cranial (*t* test)	0.4	
Median age at surgery (years old)	25	26
IQR (years old)	3	18
Range (years old)	22–26	9–43
*P* value, age at surgery, spinal patients vs cranial patients (Mann–Whitney)	*0.049*	
Sex (Females/Males)	10/0	8/2

Non-parametric test (Mann–Whitney) was performed when distributions were not normal, otherwise *t* test was used. *IQR* inter-quartile range.

*P* values < 0.05 are italicized.

### Histologic findings

**Figure 1:**
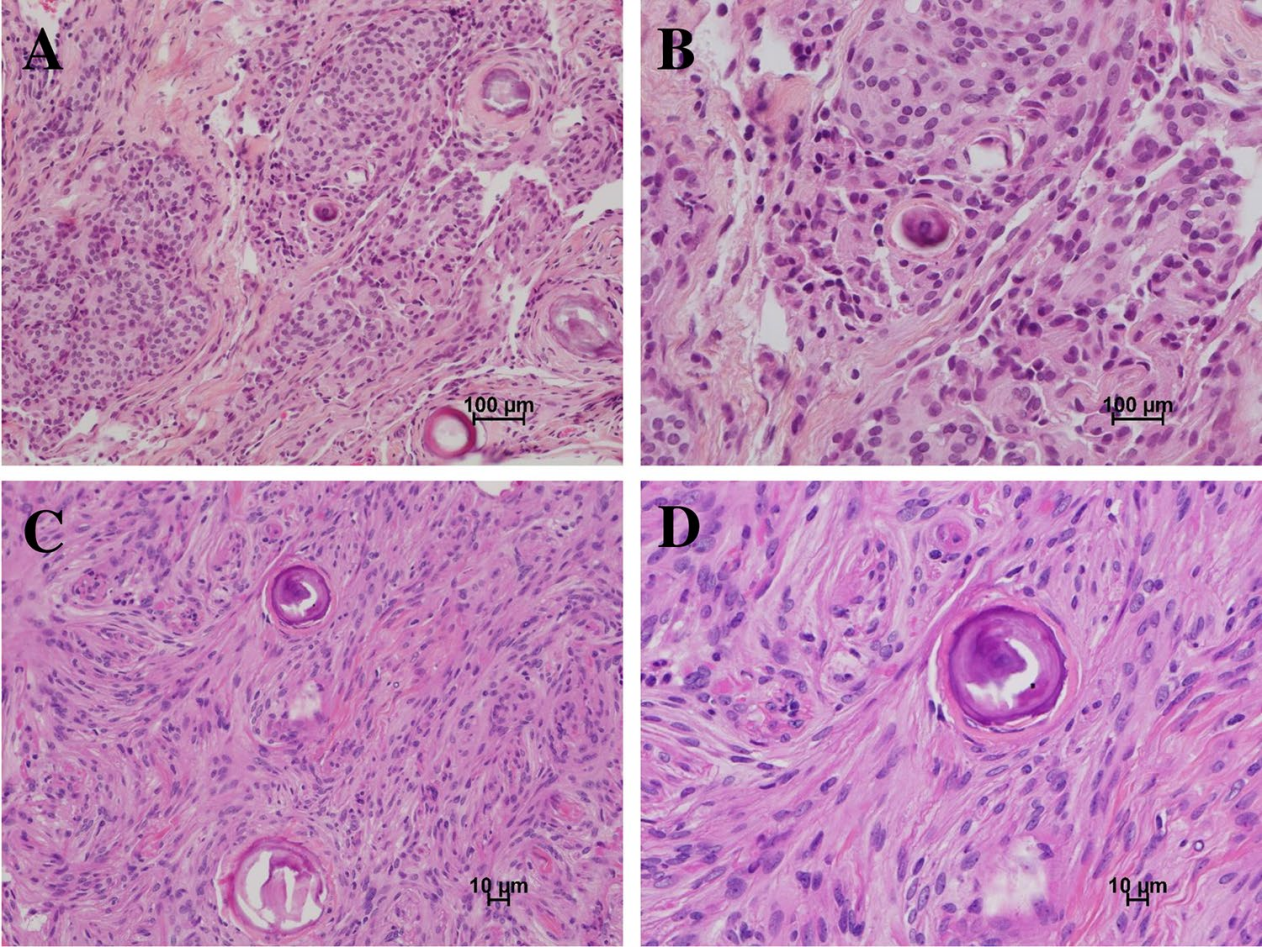
NF2-associated spinal and cranial meningioma histopathology as seen on H&E stain. (**A**) Spinal (100× magnification); (**B**) Spinal (200×); (**C**) Cranial (100×); (**D**) Cranial (200×).

All spinal and cranial neoplasms were classified as WHO grade 1 meningiomas. One of these cranial meningiomas (P6_C1) was noted to have high MIB1 staining (10–15%) and was considered worrisome for more aggressive clinical behavior but was graded as WHO grade 1 based on histopathology and the absence of > 4 mitotic figures per 10 high-power fields (hpf). This tumor had the highest relative growth rate (685%/year) among all meningiomas, and in addition to chromosome 22 loss contained large CNVs in chromosomes 1 and 17 comprising SWItch/SucroseNonFermentable (SWI/SNF) and polycomb repressor complex 2 (PRC2) genes. Representative H&E staining of the cranial and spinal meningiomas are shown in Fig. 1.

### Volume and growth rate of spinal and cranial meningiomas

**Figure 2:**
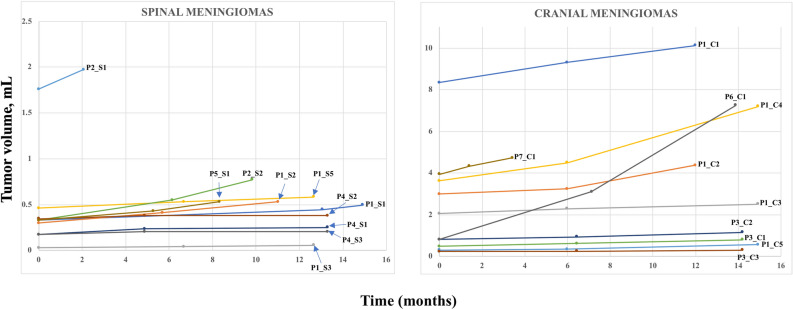
Longitudinal volume measurements of ten spinal and ten cranial meningiomas from seven NF2 patients. Tumor IDs are shown next to respective growth lines. P1 through P7 denote patients, “S” denotes spinal and “C” denotes cranial meningiomas. Note that due to substantial differences in the volumes of cranial and spinal meningiomas, the range of Y axis on the plots was adjusted accordingly to optimize visualization of the data.

MRI images of the tumors are shown in Supplementary Fig. S1. The median pre-operative tumor volume for cranial tumors was significantly larger than for spinal tumors (3.4 cm^3^ (IQR = 5.7) versus 0.5 cm^3^ (IQR = 0.3), *P* = 0.005) (Table 1). Median absolute tumor growth rate for cranial tumors was higher (with marginal significance) than for spinal tumors (0.9 cm^3^/year (IQR = 2.4) versus 0.1 cm^3^/year (IQR = 0.2), *P* = 0.045), while median relative tumor growth rates for cranial and spinal tumors were nearly identical, 49.5 percent/year (IQR = 47.4) versus 50.2 percent/year (IQR = 47.7), *P* = 0.4 (Table 1 and Fig. 2).

### Germline pathogenic variants and somatic mutations in *NF2*

We identified constitutive pathogenic variants in *NF2* in all seven patients. All germline variants were point substitutions. Five patients had nonsense C-to-T substitutions resulting in premature stop codons; the other two patients had substitutions affecting gene splicing (Table 2). Unlike the germline variants, somatic inactivation of *NF2* in the tumors occurred almost invariably via large deletions of chromosome 22. In 19 out of 20 meningiomas, we observed deletion of either the entire chromosome 22, or 22q-arm, resulting in loss of heterozygosity (LOH). None of the deletions were copy-neutral (Supplementary Fig. S2). We observed a small frameshifting deletion in a single cranial meningioma from patient P7 (Table 2).

**Table 2:** Germline pathogenic variants and somatic *NF2* mutations and CNVs in the tumors.

Patient	Sex	Germline pathogenic variant	Number of spinal meningiomas	Number of cranial meningiomas	Somatic mutation or CNV
P1	F	Nonsense, exon11, c.1021C > T, p.R341X	4	5	Chr22 LOH in all tumors
P2	F	Splice region, exon 2, c.241-9A > G	2	0	Chr22 LOH in both tumors
P3	F	Nonsense, exon2, c.193C > T, p.Q65X	0	3	Chr22 LOH in all tumors
P4	F	Nonsense, exon11, c.1030C > T, p.Q344X	3	0	Chr22 LOH in all tumors
P5	F	Splice site, exon4, c.447+1G > T	1	0	Chr22 LOH
P6	M	Nonsense, exon7, c.634C > T, p.Q212X	0	1	Chr22 LOH
P7	M	Nonsense, exon8, c.784C > T, p.R262X	0	1	Frameshift, exon12, c.1234delC, p.Q412fs

*LOH* Loss of Heterozygosity.

### Somatic mutation burden in the meningiomas

Next, we analyzed the burden of somatic mutations in 20 meningiomas. The median number of mutations per tumor besides *NF2* was 1 (range 0–5). We observed no somatic mutations in eight tumors, and one cranial meningioma had five somatic mutations (Table 3). In silico analysis of mutation pathogenicity showed that the majority of variants (13 of 19 unique substitutions) were benign or tolerated. All mutations were heterozygous. Six potentially pathogenic mutations in *PHRF1, ZC3H12B, H2AFV, DNAJC13, DNAH8, SCN2A* were non-recurrent; however, given the modest sample size it is difficult to fully evaluate the importance of these variants in the meningioma progression. There was no difference in somatic mutation burden between spinal and cranial tumors: of six potentially pathogenic variants, three were found in cranial meningiomas and three—in spinal neoplasms.

**Table 3:** Somatic mutations in meningiomas.

Gene	Chr	Position	Ref	Alt	Type	Germline depth	Tumor depth	PolyPhen2	SIFT	CADD score	Tumor ID
*PRDM9*	5	23527304	T	C	Missense	116\0	158\11	Benign	Tolerated	0.001	P1_spinal1
*PRDM9*	5	23527304	T	C	Missense	116\0	119\6	Benign	Tolerated	0.001	P1_cranial1
*PRDM9*	5	23527304	T	C	Missense	116\0	227\10	Benign	Tolerated	0.001	P1_cranial2
*PRDM9*	5	23527304	T	C	Missense	116\0	186\8	Benign	Tolerated	0.001	P1_cranial3
*PRDM9*	5	23527273	T	C	Synonymous	54\0	160\10	NA	NA	0.002	P3_cranial1
*CYP11A1*	15	74637262	G	A	Missense	1610\0	680\213	NA	NA	1.052	P1_cranial3
*PIBF1*	13	73409396	A	G	Synonymous	1994\0	1476\522	NA	NA	1.546	P1_cranial3
*PPEF2*	4	76797614	G	A	Synonymous	1443\0	855\534	NA	NA	4.544	P1_spinal1
*ENTPD4*	8	23299121	G	A	Synonymous	1238\0	1316\678	NA	NA	7.455	P2_spinal2
*ADCY9*	16	4043467	G	C	Synonymous	710\0	509\180	NA	NA	11.66	P1_cranial3
*OLFML2A*	9	127566620	A	T	Missense	14\1	7\6	Benign	Tolerated	14.41	P2_spinal1
*OLFML2A*	9	127566620	A	T	Missense	19\0	24\7	Benign	Tolerated	14.41	P4_spinal3
*PRDX3*	10	120933297	T	A	Synonymous	1998\0	1517\483	NA	NA	15.1	P1_cranial3
*IL17RE*	3	9944701	C	T	Missense	1993\0	1774\219	Benign	Deleterious	16.58	P1_cranial2
*OBSCN*	1	228509762	C	T	Missense	1445\0	1137\511	Benign	Tolerated	21.4	P1_cranial4
*UNC80*	2	210737600	A	T	Missense	1591\0	1796\203	Probably damaging	Tolerated	22.9	P2_spinal2
*ZFHX4*	8	77765258	C	T	Missense	22\1	17\5	Probably damaging	NA	23.3	P5_spinal1
***PHRF1***	**11**	**607296**	**C**	**T**	**Missense**	**879\0**	**664\256**	**Probably damaging**	**Deleterious**	**25.4**	**P1_cranial4**
***ZC3H12B***	**X**	**64721887**	**G**	**T**	**Missense**	**2000\0**	**1696\303**	**Probably damaging**	**Deleterious**	**25.8**	**P2_spinal2**
***H2AFV***	**7**	**44875257**	**C**	**T**	**Missense**	**523\0**	**308\128**	**Probably damaging**	**Deleterious**	**27.4**	**P1_cranial1**
***DNAJC13***	**3**	**132207178**	**G**	**T**	**Missense**	**1998\0**	**1000\805**	**Probably damaging**	**Deleterious**	**31**	**P1_spinal5**
***DNAH8***	**6**	**38874085**	**G**	**T**	**Missense**	**722\0**	**496\235**	**Probably damaging**	**Deleterious**	**33**	**P1_cranial1**
***SCN2A***	**2**	**166152585**	**C**	**A**	**Stop**	**1991\0**	**475\250**	**NA**	**NA**	**35**	**P1_spinal3**

Gene symbol, chromosome, chromosomal coordinates (hg19), reference and alternative alleles, type of mutation, reference and alternative alleles counts in germline and tumor, pathogenicity prediction by PolyPhen and SIFT, CADD phred score and tumor IDs are shown in columns from left to right. Mutations that were classified as probably damaging by PolyPhen2 and deleterious by SIFT and with CADD phred score above 25 were considered as pathogenic (highlighted in bold font). A single nonsense mutation in *SCN2A* was considered pathogenic as well. *NA* not applicable.

### Copy-number variation in the tumors

We next analyzed the CNV landscape in the tumors and compared the level of genomic instability in meningiomas at these two anatomical locations. Chromosome 22 deletion was observed in 19 of 20 tumors (10/10 spinal; 9/10 cranial) and was the most frequent large-scale chromosomal aberration (Supplementary Figs. S2 and S3) in both spinal and cranial meningiomas. Somewhat unexpectedly, we noticed that the growth rate of the only meningioma (P7_C1), in which the somatic inactivation of *NF2* was a small frameshifting indel rather than a loss of the entire chromosome 22, was slightly *higher* than the median growth rate of meningiomas with chromosome 22 LOH (73%/year vs*.* 50%/year). Thus, it appears that heterozygous loss of multiple genes residing in chromosome 22, including tumor suppressors, such as *CHEK2, LZTR1* and *SMARCB1*, confers no proliferative advantage to the cells.

Beyond chromosome 22 aberrations, cranial tumors displayed modestly elevated genomic instability: 3/10 cranial tumors harbored large deletions in chromosome 1p (samples P1_C3 and P6_C1), chromosome 17q (sample P6_C1) and chromosome X (sample P1_C4), while none of the spinal meningiomas did. In addition, 2/10 cranial meningiomas (P3_C2 and P3_C3) were nearly pentaploid, while the ploidy of all spinal meningiomas was close to normal diploid (Supplementary Table S1 and Supplementary Fig. S2A).

We also evaluated total CNV burden in tumors at these two anatomical locations (Supplementary Tables S1 and S2). The total number of CNVs among all cranial tumors (including total CNVs and CNVs outside of chromosome 22) was modestly higher than among spinal tumors. The median number of CNVs per tumor sample was also higher in cranial meningiomas compared to that in spinal neoplasms; however, this difference was not statistically significant by a *t* test (*P* = 0.71). The number of genes affected by the CNVs, including known cancer genes, was also higher in cranial tumors (Supplementary Tables S1 and S3).

Although we did identify several frequent gains outside of chromosome 22 (found in greater than or equal to  30% of samples) in both cranial and spinal tumors, these aberrations resided in common CNVs (*e.g.,* population polymorphisms), including clusters of T-cell receptor genes on chromosomes 7 and 14 (Supplementary Table S2), and, therefore, unlikely are deleterious. The majority of non-chromosome 22 CNVs were either non-recurrent or were present in a small number of tumors (Supplementary Tables S1 and S2). Overall, intra- and inter-patient variability between individual tumors was low, mostly due to relatively low mutation and CNV burden in the neoplasms. As noted above, chromosome 22 deletion was the most common genomic aberration, while the remaining CNVs were mostly specific for each neoplasm. We did not observe higher degree of similarity in the genomic architecture in multiple tumors resected from the same individual as opposed to neoplasms from different patients. The only exception was two cranial meningiomas C2 and C3 from patient P3: both tumors were polyploid with similar ploidies of 4.78 and 4.72, respectively, suggesting clonal origin of both neoplasms; however, most of their CNVs were unique, implying independent divergent evolution of each meningioma (Supplementary Fig. S2B).

In addition, we performed detailed genomic analyses of the fastest growing (685%/year) tumor in this study (Fig. 2, sample P6_C1). Interestingly, we did not observe any somatic point mutations in the tumor (*NF2* was somatically inactivated via chromosome 22 deletion). However, we detected signs of increased chromosomal instability: there were multiple loci of losses and gains in chromosomes 1 and 17 (Supplementary Fig. S3B). The analysis of these CNVs revealed that multiple oncogenes and tumor suppressor genes were affected by these chromosomal aberrations (Supplementary Table S4). Most strikingly, we found heterozygous loss of two tumor suppressor genes, *ARID1A* and *SMARCE1*, components of the chromatin remodeling complex SWI/SNF, and one copy gain of *SUZ12* and *EZH1*, the components of another essential chromatin remodeling complex PRC2.

## Discussion

In most circumstances, cranial tumors are larger than spinal tumors at the time of symptom development due to the capacity of the compartment from which they arise to accommodate them. An initial review of the literature describing several large surgical studies of sporadic meningiomas (NF2-associated tumor studies are more rare and usually underpowered) indicated that the mean age at surgery for sporadic cranial meningiomas was between 53.8 and 58.3 y.o.^14–17^ and the mean age at surgery for sporadic spinal meningiomas was between 53.0 and 69.0 y.o.^17–21^. In one study, in which both cranial (N = 9,806) and spinal (N = 483) tumors were operated on at the same medical center, the ages at surgery for both anatomical locations were essentially the same, 53.77 ± 28.63 y.o. and 53.76 ± 15.79 y.o.^17^, respectively. Our study highlighted that the relative growth rates of cranial and spinal tumors were similar, indicating similar division rates (divisions/year) of cranial and spinal tumor cells during the study. Because spinal tumors were smaller than cranial tumors, spinal tumors must have undergone fewer cell divisions than cranial tumors before the study began. We hypothesize that the spinal meningiomas had undergone fewer cell divisions than cranial tumors before the study because: 1) spinal meningioma initiation occurred later in life and/or 2) spinal meningiomas exhibited longer periods of quiescence while exhibiting variability in patterns of tumor growth as previously described (e.g., saltatory growth of tumors^22^). While these findings alone cannot immediately impact clinical practice, further longitudinal natural history studies may significantly be able to delineate which of the two hypothesis is more prevalent, and therefore have significant implications for an ideal time to begin surveillance imaging, and the frequency in which it is done. At this time, current recommendations remain rather ambiguous, with regional practice patterns and clinical findings dictating the age at initial craniospinal imaging and frequency of surveillance. Certainly, by identifying that spinal tumors may either initiate at a later time point in life, or demonstrate longer periods of quiescence, but still retain the ability to grow, it emphasizes that even with an initial screen without any spinal tumors in a patient with NF2, periodic surveillance is still requisite.

One limitation of our study is that all the tumors were observed over the short term (months), and the rate of volumetric growth was analyzed in the period of time immediately antecedent to surgical resection. Therefore, although these tumors likely did exhibit saltatory growth, one cannot distinguish what type of growth pattern they exhibited (saltatory, linear, exponential, etc.) over the duration of their existence due to the limited observation period included in this study. It is fundamentally important to identify the molecular mechanisms underlying these clinical observations on symptomatic tumors through detailed longitudinal natural history studies. Additional insights could come from comparing symptomatic and growing tumors with asymptomatic and/or non-growing meningiomas that are removed incidentally during surgery or procured at autopsy. Another limitation of this study is that modest number of NF2 patients and meningiomas that was analyzed rendered it underpowered; however, similar limitations are frequently encountered in the research of rare genetic disorders. In longitudinal prospective studies such as ours, as well as retrospective case–control investigations, the future challenges will include multi-center collaboration and standardization of clinical and research protocols.

In our study, cranial meningiomas were significantly larger than spinal tumors. A larger tumor mass implies a larger number of cells and, therefore, a larger number of cell divisions and associated somatic changes. Thus, the trend toward higher chromosomal instability in cranial meningiomas observed in this study could be explained by elevated number of cell cycles undergone by the neoplasms. Our findings are consistent with observations of Sayagues and colleagues^23^, who performed a large study of *sporadic* spinal and cranial meningiomas and found that cranial tumors generally have more chromosomal aberrations than spinal meningiomas. It also has been shown that meningioma progression to higher WHO grades is frequently associated with increased chromosomal losses and gains (reviewed in Perry et al., 2004^24^).

Significant progress has recently been made in elucidating the genomic architecture of sporadic meningiomas^25–29^. However, our understanding of the genomics of their NF2-associated counterparts, which are distinct from sporadic tumors due to heterozygous *NF2* inactivation in the zygote or early embryo, remains limited. Molecular mechanisms leading to increased genomic instability in NF2-associated tumors are poorly understood but some evidence suggests that it could result from *NF2* deficiency per se. Several reports have shown that *NF2* inactivation abrogates merlin function, affecting positioning of centrosomes in the interphase cell, proper orientation of spindles and, potentially, chromosomal stability^30–32^. Remarkably, in this study we found that somatic inactivation of *NF2*, the tumor initiation event, occurred via chromosome 22 loss in 95% of the tumors, and only in one case via a frameshifting indel. Beyond chromosome 22, CNV aberrations in the genomes of tumors were rare and mostly non-recurrent underscoring their random non-causal character. When found in more than one sample, the frequency of these CNVs was generally low in the sample set and appeared to be contributing to inter- or intra-patient variability equally. These observations are consistent with the widely accepted concept that in benign NF2-associated meningiomas inactivation of *NF2* is the main and most likely the only genetic event necessary for tumor initiation.

Somatic mutation burden in the tumors was low (median = 1, including *NF2*). One of the few possibly deleterious variants was found in *PHRF1* in the second fastest growing tumor in the study, a cranial meningioma (sample P1_C4). *PHRF1* encodes PHD and RING finger domain-containing protein 1, which acts as a tumor suppressor in breast cancer and promotes TGF-β/SMAD signaling by ensuring cytoplasmic re-localization of promyelocytic leukemia protein (PML)^33^. PML is involved in wide range of important cellular processes, including tumor suppression, transcriptional regulation, apoptosis, senescence, DNA damage response, and viral defense mechanisms^34^. In our previous study^35^, we exome-sequenced a fast-growing NF2-associated atypical meningioma and have shown that it carries a deleterious variant in *CAPN5* which encodes a protein also interacting with PML. These observations suggest that PML may play a role in meningioma progression and warrant further studies.

An interesting finding was that the fastest growing cranial meningioma (P6_C1) carried one-copy loss of *ARID1A* and *SMARCE1*, the subunits of SWI/SNF, and one-copy gain of *SUZ12* and *EZH1*, the components of PRC2. The SWI/SNF complex activates transcription of target genes by displacing and re-positioning nucleosomes on DNA in an ATP-dependent manner, while PRC2 inactivates transcription by tri-methylation of histone H3 lysine 27 (H3K27me3), thus targeting local chromatin for conversion into an inactive heterochromatic conformation. The two machineries have been shown to act in an opposing manner to establish and maintain the transcriptional balance of genes controlling pluripotency, proliferation and differentiation in embryonic and adult stages. A recent report showed increased H3K27me3 signal and *EZH2* overexpression in sporadic atypical meningiomas and a potential role of upregulated PRC2 in meningioma progression^27^. Finally, patients with familial multiple spinal meningiomas (without germline *NF2* pathogenic variants) were shown to frequently have germline heterozygous loss-of-function variants in *SMARCE1* and complete loss of the protein in the tumors^36^. These and our findings suggest that chromatin remodeling factors may play an important role in meningioma growth and progression.

In a recent study, Patel and colleagues performed comprehensive molecular and clinical profiling of a set of 160 meningiomas, which included all three WHO grades^37^. Unsupervised clustering of the tumors based on their whole-transcriptome profiles revealed three distinct groups that did not directly correlate the WHO grades, but reliably predicted aggressive clinical behavior such as frequency of tumor recurrence. The authors proposed a novel classification that links molecular profile of meningiomas with their potential to recur: (1) benign tumors that carry intact *NF2,* but have mutations in other genes (e.g., *TRAF7, AKT1, KLF4*); (2) benign tumors that carry biallelic loss of *NF2* and presented with SWI/SNF and PRC2 gene involvement; and (3) aggressive meningiomas with high risk of recurrence that have inactivated *NF2* and carry chromosome 1p loss and the loss of the repressor function of DREAM, a chromatin-remodeling complex involved in cell cycle progression^38^. These findings underscore the importance of molecular profiling of meningiomas and the limitations of the WHO grading system.

Most tumors analyzed in this study carried inactivated *NF2*, implying that targeting cellular pathways that are activated by the abrogated merlin function (e.g., Hippo, PI3K-AKT-mTOR, contact inhibition) could be beneficial for treating these tumors (reviewed in Apra et al., 2018^39^). For instance, mTOR inhibitors vistusertib and everolimus (the latter in combination with a somatostatin analog, octreotide) are currently tested in clinical trials for recurrent meningiomas (NCT03071874 and NCT02333565, respectively). A PTK2/FAK inhibitor GSK2256098 is currently being tested in a clinical trial with recurrent meningiomas carrying inactivated *NF2* (NCT02523014). In preclinical studies, GSK2256098 has been shown to inhibit tumor cell viability, anchorage-independent growth, and motility and in clinical trials in solid tumors this drug demonstrated cytostatic effects as a single agent and extended progression-free survival (reviewed in Mohanty et al., 2020^40^).

In conclusion, NF2-associated benign symptomatic cranial meningiomas had significantly larger volumes than meningiomas in the spine; however, the relative growth rates of cranial and spinal tumors were similar and low. *NF2* complete inactivation is the main, and possibly only, genetic event leading to meningioma initiation in NF2 patients. Chromosomal instability, which could be elevated by *NF2* haploinsufficiency and further increased by loss of the second copy of *NF2*, is likely a predominant route of tumor evolution. These findings begin to explain the similarities and subtle differences between cranial and spinal meningiomas in a defined tumor predisposition syndrome, in which differences in the underlying genomic architecture of histologically indistinguishable tumors may contribute to differences in clinical behavior and patient outcomes.

## Methods

### Patient information

Seven adult and three pediatric patients enrolled in a prospective NF2 natural history study (NIH Clinical Trial # 08-N-0044; clinicaltrials.gov identifier NCT00598351) were included. Tumor tissue and germline DNA (peripheral blood) were procured from study patients undergoing resection of symptomatic meningiomas under a different trial (NIH Clinical Trial # 03-N-0164; clinicaltrials.gov identifier NCT00060541). The studies were approved by the Combined Neuroscience Institutional Review Board and all research was performed in accordance with relevant guidelines and regulations. Informed written consent was obtained from all participants. Patients were diagnosed with NF2 by Manchester clinical criteria and/or harbored a causative germline pathogenic variant in the *NF2* gene.

### Surgical resection of tumors

Patients underwent tumor resection via conventional surgical approaches, with adequate tumor reserved for histopathologic diagnosis. The remainder of each tumor specimen was snap frozen in isobutane, embedded in optimal cutting temperature compound (PSL Equipment), and stored at − 80 °C.

### Histopathology analysis

Specimens submitted for histopathologic analysis were fixed in 10% buffered formalin immediately after removal, processed overnight, and embedded in paraffin. Five μm-thick sections were obtained and stained using the standard H&E method. When required for diagnosis, immunohistochemical evaluation and Ki-67 labeling index was performed to characterize the tumor.

### Imaging/tumor growth volumetric analysis

Patients underwent MRI on a 3 T MR scanner (Phillips) at the time of entry into Natural History Study of Patients With Neurofibromatosis Type 2 (NCT00598351) and thereafter semiannually and as needed to evaluate clinical symptoms. The volume of each the meningioma was calculated from measurements made on post-contrast T1-weighted images using the formula: volume = (maximum anteroposterior dimension X maximum mediolateral dimension × maximum craniocaudal dimension)/2^3^^.^ The dural tail was not included in any tumor measurements, since the dural tail of globular meningiomas may either contain tumor or represent engorged vasculature^41^.

Absolute and relative tumor volume growth rates were calculated by comparing tumor volume on the preoperative imaging study with tumor volume at the preceding study visit. Absolute tumor growth was calculated as tumor volume change between these measurements divided by time between the measurements and expressed as cm^3^/year. Relative tumor growth was calculated by dividing the absolute increase of tumor volume per year by the initial tumor volume and expressed as percent/year.

### Whole exome sequencing (WES) of tumors

Exome-sequencing of genomic DNA was performed as previously described^35^. Briefly, capture of the coding portion (exome) of genomic DNA and library preparation for next generation sequencing was done using Roche NimbleGen (Madison, WI) SeqCap EZ Exome + UTR library (64 Mb of coding exons and miRNA regions plus 32 Mb untranslated regions (UTR)) according to the manufacturer’s instructions. As an input, 1 μg of tumor and matching normal genomic DNA was used. Sequencing was completed on the Illumina HiSeq 2,500 system (Illumina, San Diego, CA, USA). Among the exomes sequenced, the average breadth of coverage was 89% (range 88–90%), and the average depth of coverage was 66X (range 54X–78X).

### WES data analysis

To call germline variants from matched normal samples, sequencing reads were aligned to NCBI Build GRCh37 (hg19) using Novoalign v.2.08.02 (https://www.novocraft.com/); bam2mpg for genotype calling and calculation of the quality score Most Probable Genotype (MPG)^42^ and ANNOVAR (https://annovar.openbioinformatics.org/en/latest/) for functional annotation of genetic variants^43^. The data was formatted in VarSifter^44^ format for further filtering. To detect somatic mutations, sequencing reads were aligned to NCBI Build GRCh37 (hg19) using Novoalign v.2.08.02, and point mutations and small indels were discovered for all tumor-normal pairs with the programs Mutect (v.1.1.4)^45^, SomaticSniper (v.1.0.5)^46^ and Shimmer (v.0.1.1)^47^. Variant allele frequencies (VAFs) for all somatic alterations predicted by any of the three programs were then calculated and tabulated in VarSifter format. Mutect and Shimmer were run with default parameters, while SomaticSniper variants were filtered as recommended by the program authors. Coding variants that were identified as somatic mutations by at least one mutation caller were further filtered through 1,000 Genomes, ExAC, ESP6500 and ClinSeq databases and variants that were present in these databases at frequency > 1% were removed from further consideration. The remaining variants were compared to the exclusion list of more than 2,000 genes described in Fajardo and co-authors^48^ and variants residing in these genes were excluded from further consideration.

### Somatic mutation validation and deep *NF2* mutation detection by Ampliseq/IonTorrent

Orthogonal validation of somatic mutations and exon sequencing of *NF2* was done as described previously^49^. Briefly, multiplex PCR primers for somatic mutation verification were designed using Ion Ampliseq Designer (v.3.0.1, Life Technologies, Grand Island, NY, USA). Multiplex PCR amplification, library preparation and sequencing on Ion Proton sequencer (Life Technologies) were performed per the manufacturer’s instructions. Reads from the IonTorrent Proton sequencer were filtered, and adaptor- and quality-trimmed. The resulting sequences were aligned to human reference genome hg19 using TMAP (Life Technologies). Resulting BAM files were further aligned using The Genome Analysis Toolkit (GATK, https://software.broadinstitute.org/gatk/). For missense and nonsense mutations, *NF2* cDNA was examined by reverse-transcription PCR (Superscript II, Invitrogen) using primers flanking the affected exon, followed by Sanger sequencing (ABI Big Dye 3) to screen for cryptic splicing errors.

### Copy-number variation (CNV) and loss of heterozygosity (LOH) analysis by Nexus and ASCAT

SNP genotyping was performed using HumanOmniExomeExpress BeadChip kits (Illumina, San Diego, CA) as per the manufacturer’s instructions. “Allele-Specific Copy-number Analysis of Tumors” (ASCAT, v.2.1) analysis was performed as previously described^50^. The paired CNV and LOH analysis of tumor and matching normal DNA was performed by using Nexus v.6.1 software (BioDiscovery Inc., Hawthorne, CA, USA) as described previously^51^. The analysis settings were selected based on the developer’s suggestions for analysis of tumor samples.

## Supplementary information

Supplementary Information.
