# Supplementary material for: Comparative clinical and genomic analysis of neurofibromatosis type 2-associated cranial and spinal meningiomas
Source: Sci Rep. 2020 Jul 28;10:12563. doi: 10.1038/s41598-020-69074-z (PMC7387487; doi:10.1038/s41598-020-69074-z)
Supplement: Supplementary file 1 — Supplementary Information. [file 41598_2020_69074_MOESM1_ESM.pdf]

Comparative clinical and genomic analysis of neurofibromatosis type 2-associated cranial and spinal meningiomas

Alexander Pemov[1&], Ramita Dewan[2&,#a], Nancy F. Hansen[3], Settara C. Chandrasekharappa[3], Abhik Ray-Chaudhury[2], Kristine Jones[4], Wen Luo[4], John D. Heiss[2], James C. Mullikin[3,5], Prashant Chittiboina[2], Douglas R. Stewart[1*] and Ashok R. Asthagiri[2*,#b]

[1]Clinical Genetics Branch, Division of Cancer Epidemiology and Genetics, National Cancer Institute, NIH, Rockville, MD, USA

[2]Surgical Neurology Branch, National Institute of Neurological Disorders and Stroke, NIH, Bethesda, MD, USA

[3]Cancer Genetics and Comparative Genomics Branch, National Human Genome Research Institute, NIH, Bethesda, MD, USA

[4]Cancer Genomics Research Laboratory, Division of Cancer Epidemiology and Genetics, Frederick National Laboratory for Cancer Research, Rockville, MD, USA

[5]NIH Intramural Sequencing Center, National Human Genome Research Institute, NIH, Rockville, MD, USA

[#a]Current Address: Neuromuscular Disease Research Section, National Institute on Aging, NIH, Bethesda, MD, USA

[#b]Current Address: Department of Neurological Surgery, University of Virginia School of Medicine, Charlottesville, VA, USA

*Corresponding authors

E-mails: drstewart@mail.nih.gov (DRS); ara5x@hscmail.mcc.virginia.edu (ARA)

[&]These authors contributed equally to this work.

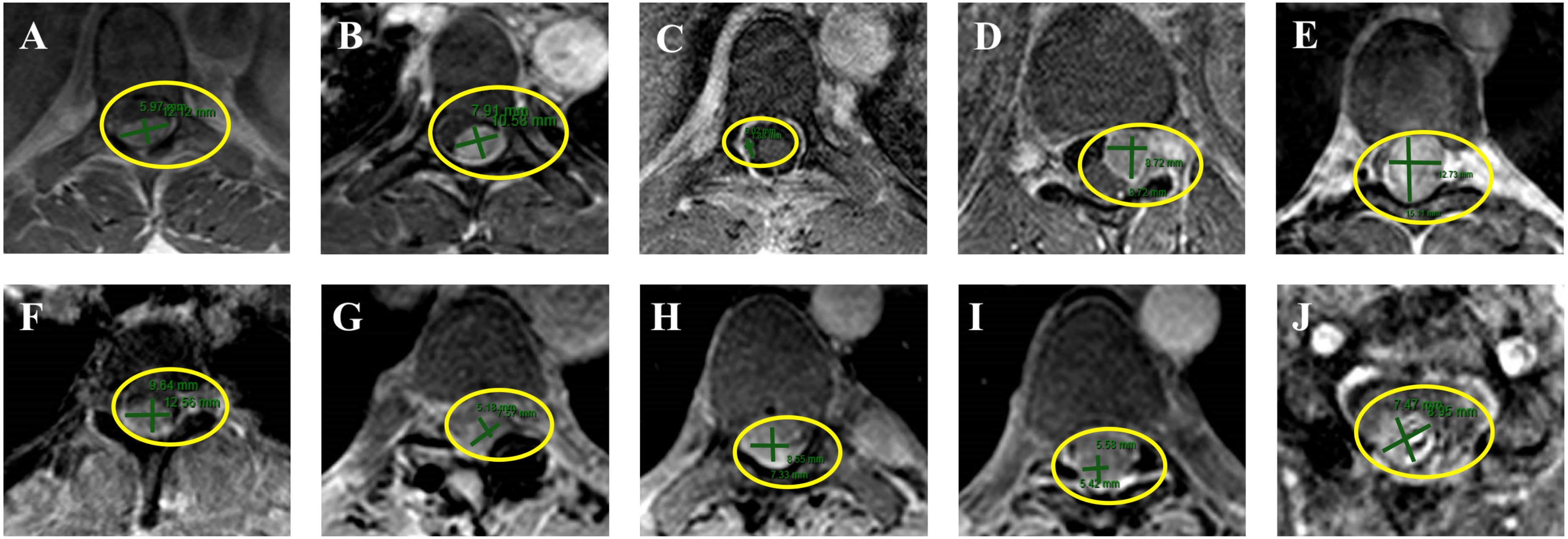

Supplementary Figure S1A. Axial T1 post-contrast MRI imaging of spinal meningiomas. A) P1_spinal1; B) P1_spinal2; C) P1_spinal3; D) P1_spinal5; E) P2_spinal1; F) P2_spinal2; G) P4_spinal1; H) P4_spinal2; I) P4_spinal3; J) P5_spinal1. Thick yellow ovals highlight the particular lesion, while thin green lines demarcate the areas used for tumor measurements.

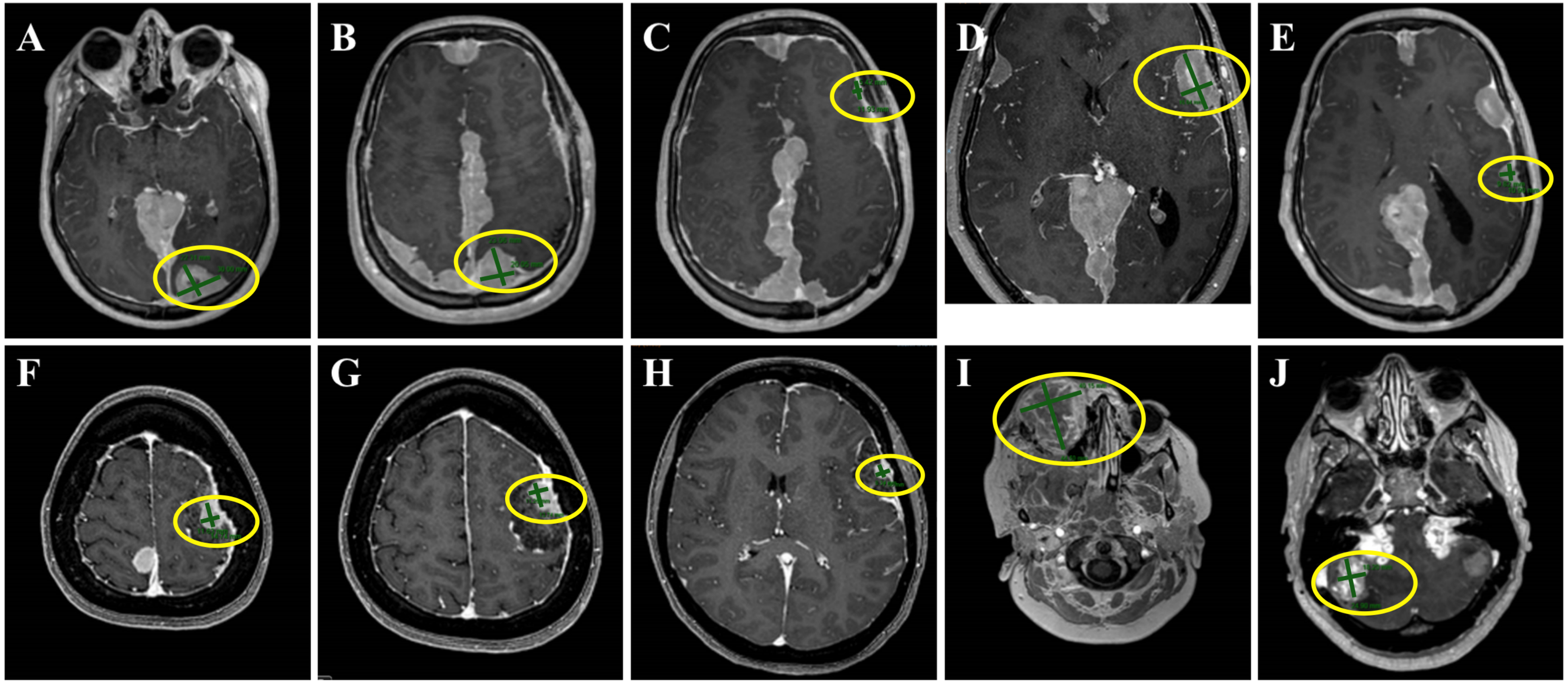

Supplementary Fig S1B. Axial T1 post-contrast MRI imaging of cranial meningiomas. A) P1_cranial1; B) P1_cranial2; C) P1_cranial3; D) P1_cranial4; E) P1_cranial5; F) P3_cranial1; G) P3_cranial2; H) P3_cranial3; I) P6_cranial1; J) P7_cranial1. Thick yellow ovals highlight the particular lesion, while thin green lines demarcate the areas used for tumor measurements.

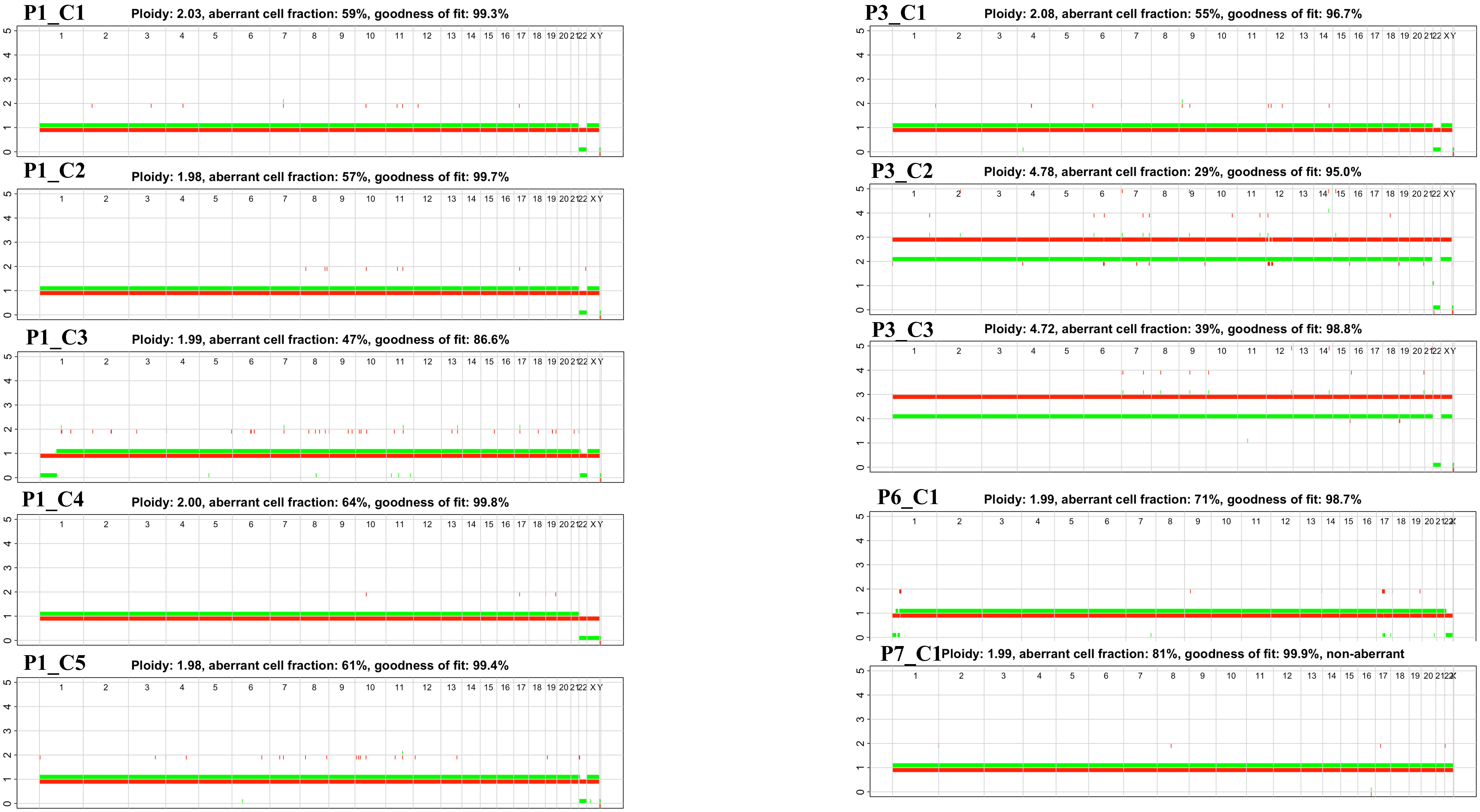

Supplementary Fig S2A. ASCAT analysis of cranial meningiomas. Homologous chromosomes are shown in red or green. Copy number is plotted on Y axis and individual chromosomes are shown on X axis.

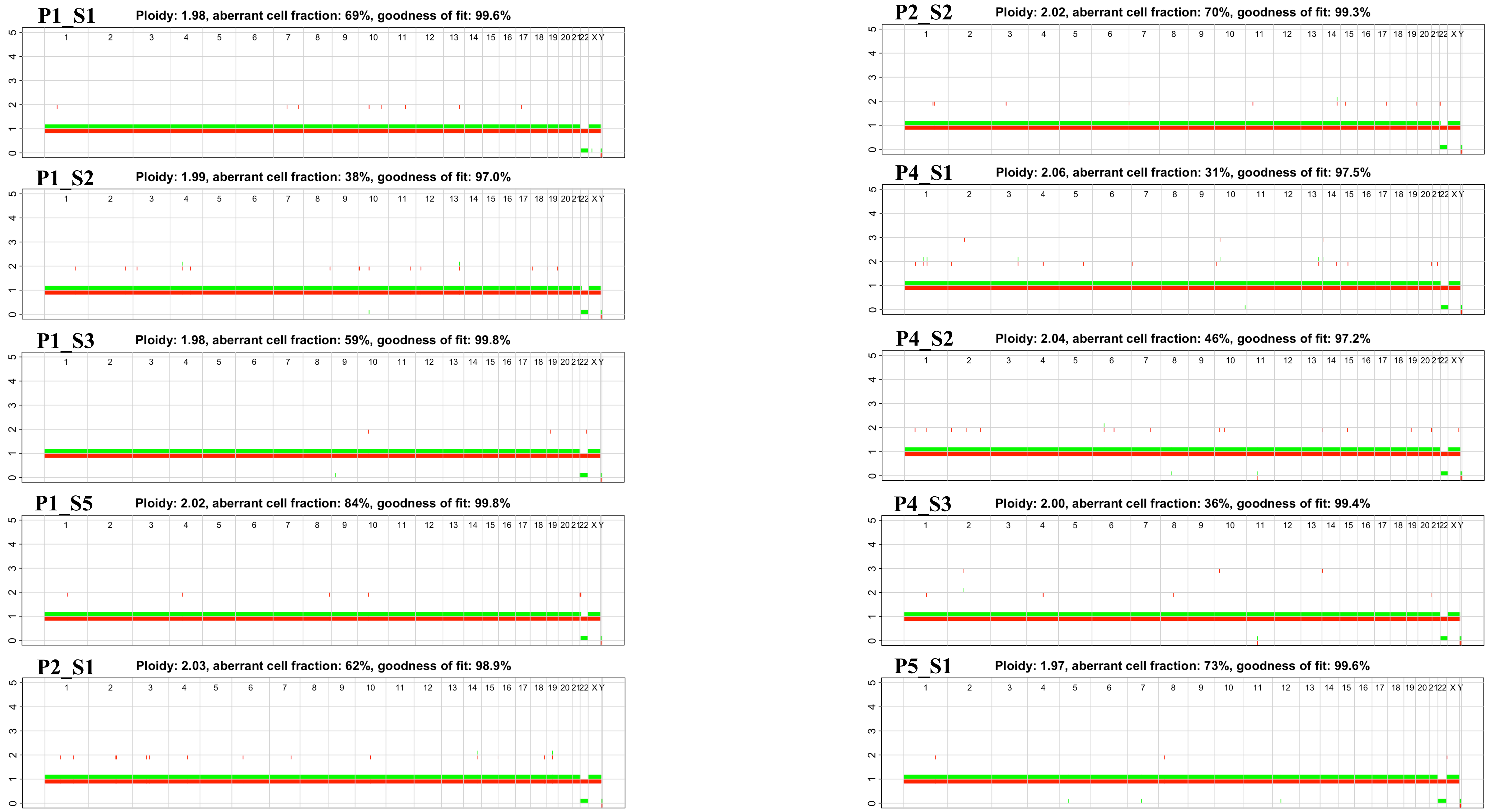

Supplementary Fig S2B. ASCAT analysis of spinal meningiomas. See legend to Supplementary Figure S2A.

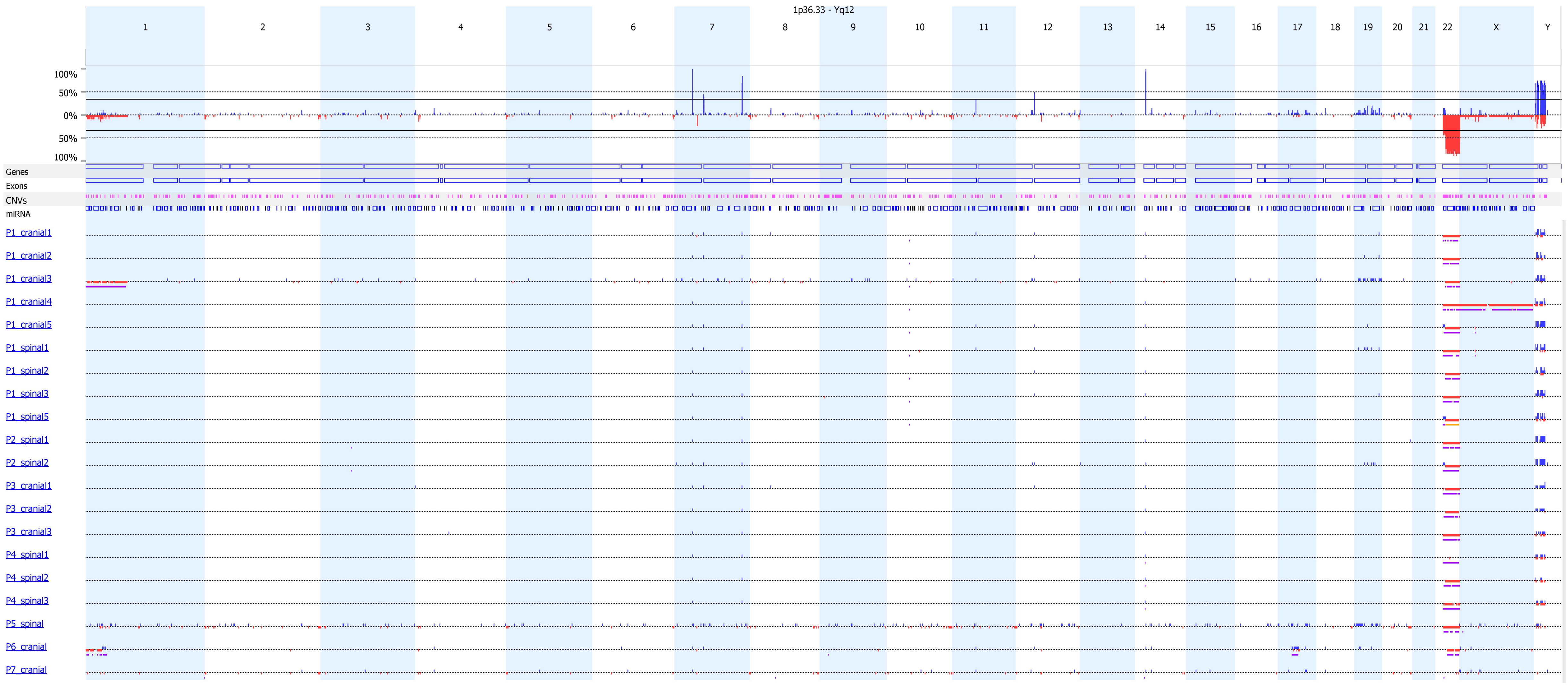

Supplementary Fig S3A. Nexus CNV analysis of meningiomas, whole genome view. Losses, gains, LOH and regions of allelic imbalance are shown in red, blue, gold and purple, respectively. Samples are shown in rows and chromosomes are shown in columns.

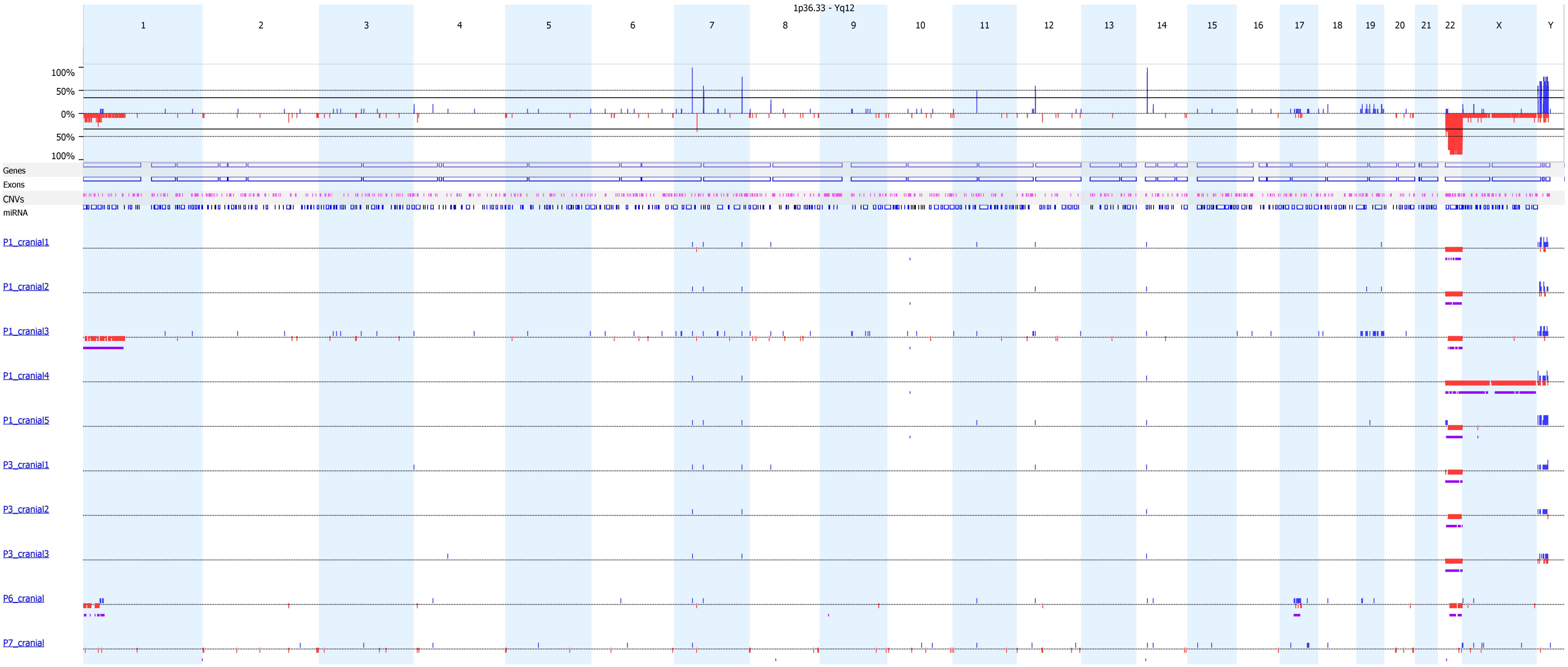

Supplementary Fig S3B. Nexus CNV analysis of ten cranial meningiomas, whole genome view. See legend to Supplementary Fig S3A.

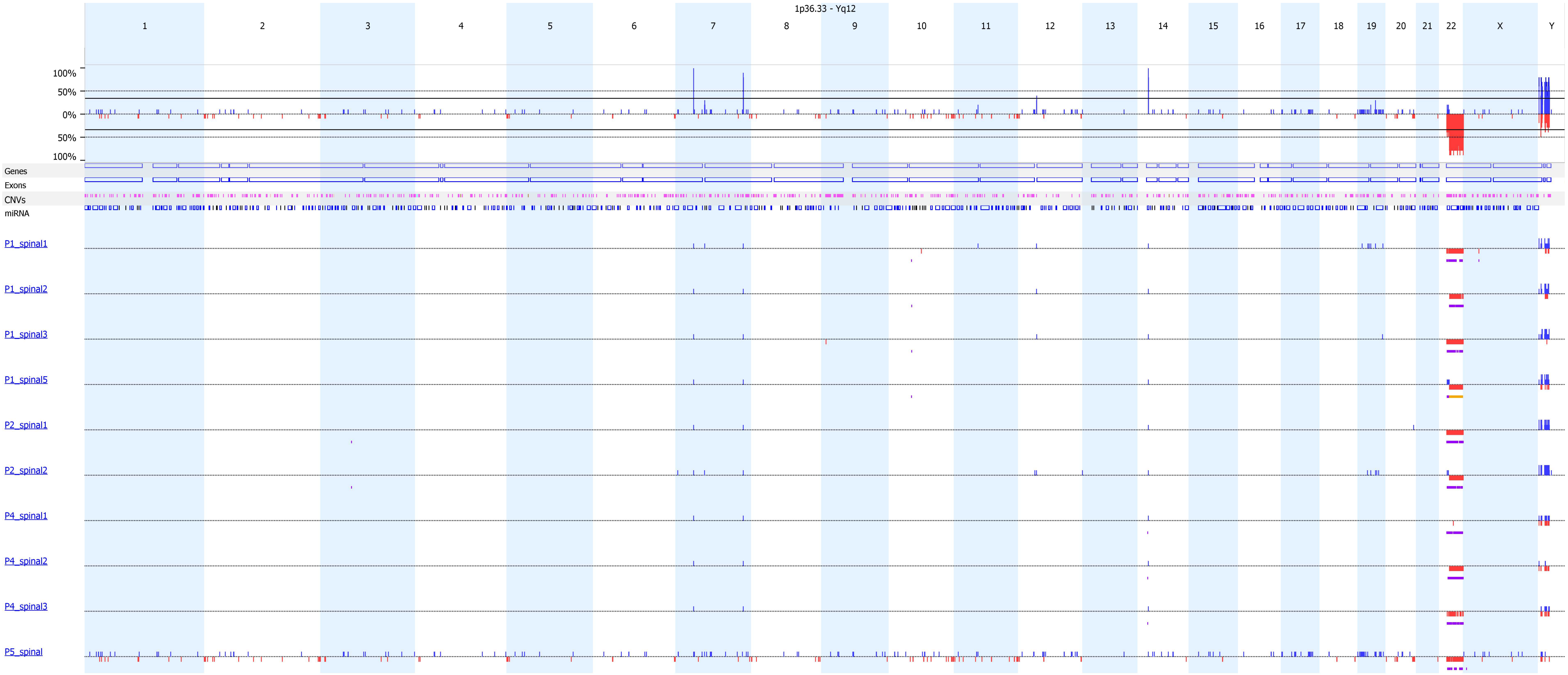

Supplementary Fig S3C. Nexus CNV analysis of ten spinal meningiomas, whole genome view. See legend to Supplementary Fig S3A.

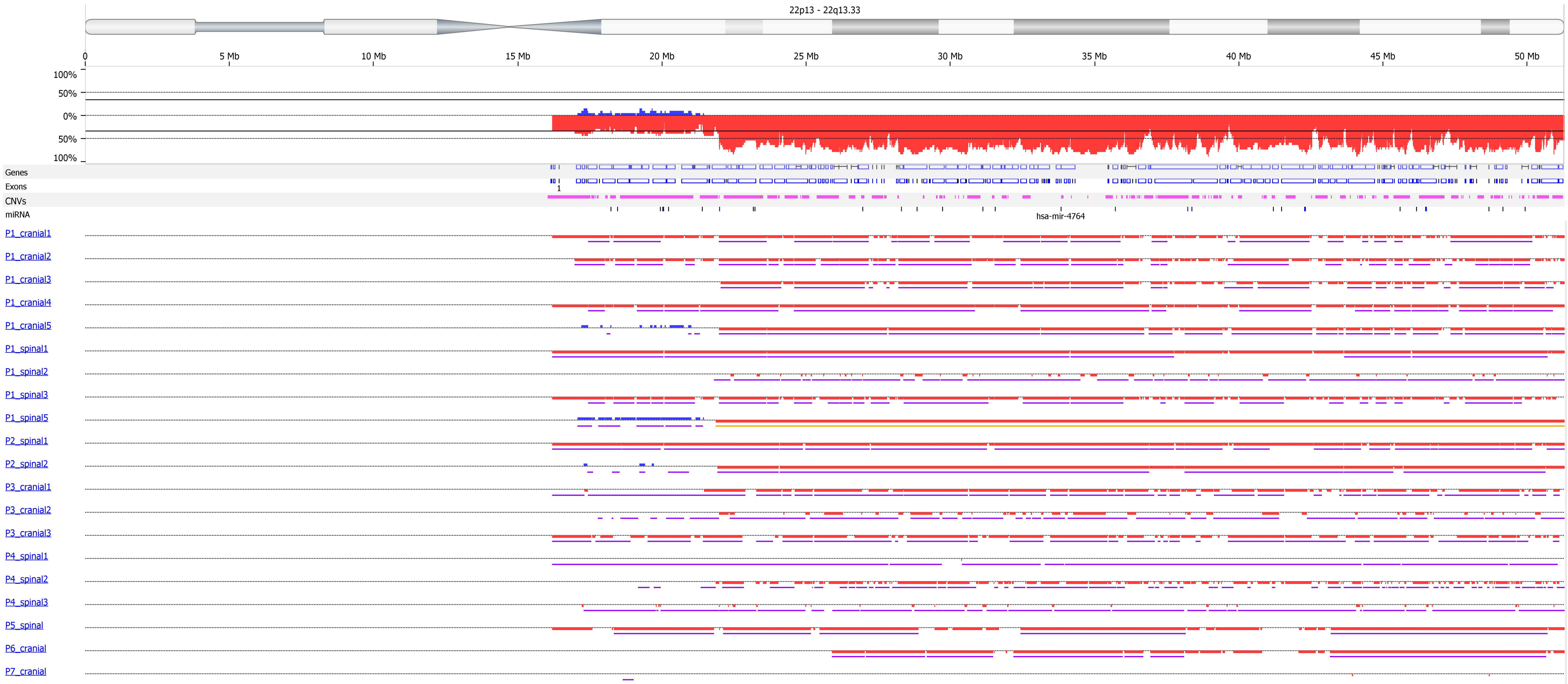

Supplementary Fig S3D. Nexus CNV analysis of meningiomas, chromosome 22. See legend to Supplementary Fig S3A.

Supplementary Table S1. Comparison of CNV burden in spinal and cranial meningiomas. MOR - minimally overlapped region of CNVs in 2 or more samples.

CNV metric	Spinal	Cranial
Total number of CNVs	650	800
Number of singleton CNVs	355	471
Number of MORs	295	329
All non-chr22 CNVs	357	548
Number of singleton non-chr22 CNVs	346	460
Number of non-chr22 MORs	11	88
Median number of CNV per tumor	67	100
Lowest number of CNV per tumor	33	50
Highest number of CNV per tumor	367	303
Number of genes in all CNVs	2,069	3,141
Number of cancer genes in all CNVs	54	107
Number of genes in MORs	660	1,065
Number of cancer genes in MORs	19	29
Number of samples with polyploidy	0 out of 10	2 out of 10
Number of samples with large non-chr22 aberrations	0 out of 10	3 out of 10

Supplementary Table S2A. CNVs in 10 spinal meningiomas, including CNVs in chromosome 22. Chromosomal coordinates of the region (hg19); the region length in bp; the chromosomal cytoband; frequency of the region occurrence in the tumor samples; percent of the region's overlap with a known CNV; type of the variation (gain, loss, or allelic imbalance); sample ID; type of the participation of a individual CNV in the region (complete or partial); gene symbols; microRNA symbols; number of genes in the region; and the number of microRNA genes found in the region are shown from left to right

Chromosomal region	Region length (bp)	Cytoband	Frequency of the CNV occurrence in samples	Overlap with known CNVs (%)	Type of CNV	Sample ID	Participation	Gene symbols	miRNAs symbols	Gene count	miRNAs count
chr14:22,501,912-22,972,885	470,973	q11.2	100	100	Gain;Gain;G	P1_S1;P1_S2;P1_S3;P1_S5;P2_S1;P2_S2;P4_	Complete;Com	*TCRA, TRA@, TRAV2*		42	0
chr7:38,307,524-38,365,774	58,250	p14.1	100	100	Gain;Gain;G	P1_S1;P1_S2;P1_S3;P1_S5;P2_S1;P2_S2;P4_	Complete;Com	*TRGC2, TARP, TCRG*		5	0
chr22:19,221,636-19,419,970	198,334	q11.21	90	8	AI;AI;AI;A	P1_S1;P1_S3;P1_S5;P2_S1;P2_S2;P4_S1;P4_	Complete;Com	*CLTCL1, HIRA, MRP*		3	0
chr22:22,119,537-22,298,200	178,663	1.21 - q11.	90	70	AI;AI;AI;A	P1_S1;P1_S2;P1_S3;P2_S1;P2_S2;P4_S1;P4_	Complete;Com	*MAPK1, PPM1F, LO*		3	0
chr22:22,316,816-22,381,035	64,219	q11.22	90	100	AI;AI;AI;A	P1_S1;P1_S2;P1_S3;P2_S1;P2_S2;P4_S1;P4_	Complete;Com	*TOP3B, PRAMENP, A*		4	0
chr22:22,454,702-22,501,225	46,523	q11.22	90	100	Loss;Loss;L	P1_S1;P1_S2;P1_S3;P1_S5;P2_S1;P2_S2;P4_	Complete;Com	*abParts*		1	0
chr22:22,501,225-22,841,767	340,542	q11.22	90	100	AI;AI;AI;A	P1_S1;P1_S2;P1_S3;P2_S1;P2_S2;P4_S1;P4_	Complete;Com	*VPREB1, LOC96610,*		6	0
chr22:23,286,985-23,318,822	31,837	q11.22	90	17	Loss;Loss;L	P1_S1;P1_S2;P1_S3;P1_S5;P2_S1;P2_S2;P4_	Complete;Complete;Complete;Comple			0	0
chr22:23,739,091-24,048,825	309,734	q11.23	90	100	AI;AI;AI;A	P1_S1;P1_S2;P1_S3;P2_S1;P2_S2;P4_S1;P4_	Complete;Com	*ZDHHC8P1, LINC01*		10	0
chr22:24,584,498-24,600,104	15,606	q11.23	90	100	AI;AI;AI;A	P1_S1;P1_S2;P1_S3;P2_S1;P2_S2;P4_S1;P4_	Complete;Com	*SUSD2*		1	0
chr22:24,979,645-24,984,260	4,615	q11.23	90	100	Loss;Loss;L	P1_S1;P1_S2;P1_S3;P1_S5;P2_S1;P2_S2;P4_	Complete;Com	*FAM211B, LRRC75B,*		3	0
chr22:25,158,441-25,166,642	8,201	q11.23	90	23	Loss;Loss;L	P1_S1;P1_S2;P1_S3;P1_S5;P2_S1;P2_S2;P4_	Complete;Com	*PIWIL3, TOP1P2*		2	0
chr22:26,168,407-26,174,472	6,065	q12.1	90	0	AI;AI;AI;A	P1_S1;P1_S2;P1_S3;P2_S1;P2_S2;P4_S1;P4_	Complete;Com	*MYO18B*		1	0
chr22:26,191,425-26,422,887	231,462	q12.1	90	0	AI;AI;AI;A	P1_S1;P1_S2;P1_S3;P2_S1;P2_S2;P4_S1;P4_	Complete;Com	*MYO18B*		1	0
chr22:26,484,688-26,563,648	78,960	q12.1	90	0	AI;AI;AI;A	P1_S1;P1_S2;P1_S3;P2_S1;P2_S2;P4_S1;P4_	Complete;Complete;Complete;Comple			0	0
chr22:26,638,419-26,706,137	67,718	q12.1	90	0	AI;AI;AI;A	P1_S1;P1_S2;P1_S3;P2_S1;P2_S2;P4_S1;P4_	Complete;Com	*SEZ6L*		1	0
chr22:26,737,988-26,859,906	121,918	q12.1	90	6	AI;AI;AI;A	P1_S1;P1_S2;P1_S3;P2_S1;P2_S2;P4_S1;P4_	Complete;Com	*SEZ6L, ASPHD2, HP*		3	0
chr22:26,887,868-26,943,707	55,839	q12.1	90	16	AI;AI;AI;A	P1_S1;P1_S2;P1_S3;P2_S1;P2_S2;P4_S1;P4_	Complete;Com	*SRRD, TFIP11, LOC1*		4	0
chr22:26,963,346-27,021,449	58,103	q12.1	90	0	AI;AI;AI;A	P1_S1;P1_S2;P1_S3;P2_S1;P2_S2;P4_S1;P4_	Complete;Com	*TPST2, CRYBB1, CR*		3	0
chr22:27,251,774-27,340,211	88,437	q12.1	90	4	AI;AI;AI;A	P1_S1;P1_S2;P1_S3;P2_S1;P2_S2;P4_S1;P4_	Complete;Com	*LOC110091768, LIN*		2	0
chr22:27,395,497-27,852,229	456,732	q12.1	90	0	AI;AI;AI;A	P1_S1;P1_S2;P1_S3;P2_S1;P2_S2;P4_S1;P4_	Complete;Com	*AK055980, LOC28448*		7	0
chr22:28,384,483-28,666,035	281,552	q12.1	90	10	AI;AI;AI;A	P1_S1;P1_S2;P1_S3;P2_S1;P2_S2;P4_S1;P4_	Complete;Com	*TTC28-AS1, TTC28*		2	0
chr22:28,756,994-28,769,854	12,860	q12.1	90	0	AI;AI;AI;A	P1_S1;P1_S2;P1_S3;P2_S1;P2_S2;P4_S1;P4_	Complete;Com	*TTC28*		1	0
chr22:30,494,664-30,585,047	90,383	q12.2	90	19	Loss;Loss;L	P1_S1;P1_S2;P1_S3;P1_S5;P2_S1;P2_S2;P4_	Complete;Com	*HORMAD2*		1	0
chr22:32,517,780-32,601,816	84,036	q12.3	90	58	AI;AI;AI;A	P1_S1;P1_S2;P1_S3;P2_S1;P2_S2;P4_S1;P4_	Complete;Com	*AP1B1P1, JB175027,*		5	0
chr22:32,651,300-33,137,499	486,199	q12.3	90	8	AI;AI;AI;A	P1_S1;P1_S2;P1_S3;P2_S1;P2_S2;P4_S1;P4_	Complete;Com	*SLC5A4, SLC5A4-AS1*		12	0
chr22:33,279,772-33,528,057	248,285	q12.3	90	10	AI;AI;AI;A	P1_S1;P1_S2;P1_S3;P2_S1;P2_S2;P4_S1;P4_	Complete;Com	*SYN3, LINC01640*		2	0
chr22:33,605,693-33,949,816	344,123	q12.3	90	32	AI;AI;AI;A	P1_S1;P1_S2;P1_S3;P2_S1;P2_S2;P4_S1;P4_	Complete;Com	*MIR4764, LARGE1, L*		3	0
chr22:33,981,320-34,134,002	152,682	q12.3	90	1	AI;AI;AI;A	P1_S1;P1_S2;P1_S3;P2_S1;P2_S2;P4_S1;P4_	Complete;Com	*LARGE1, LARGE, SN*		5	0
chr22:34,172,694-34,516,763	344,069	q12.3	90	3	AI;AI;AI;A	P1_S1;P1_S2;P1_S3;P2_S1;P2_S2;P4_S1;P4_	Complete;Com	*LARGE1, LARGE*		2	0
chr22:35,094,396-35,563,613	469,217	q12.3	90	26	AI;AI;AI;A	P1_S1;P1_S2;P1_S3;P2_S1;P2_S2;P4_S1;P4_	Complete;Com	*ISX-AS1, ISX, LINC01*		3	0
chr22:35,612,432-35,754,262	141,830	q12.3	90	1	AI;AI;AI;A	P1_S1;P1_S2;P1_S3;P2_S1;P2_S2;P4_S1;P4_	Complete;Com	*LINC01399, HMGXB*		5	0
chr22:35,779,143-36,005,801	226,658	q12.3	90	0	AI;AI;AI;A	P1_S1;P1_S2;P1_S3;P2_S1;P2_S2;P4_S1;P4_	Complete;Com	*HMOX1, MCM5, RAS*		4	0
chr22:38,934,541-38,964,668	30,127	q13.1	90	0	Loss;Loss;L	P1_S1;P1_S2;P1_S3;P1_S5;P2_S1;P2_S2;P4_	Complete;Com	*DMC1*		1	0
chr22:44,083,399-44,191,287	107,888	q13.2	90	5	Loss;Loss;L	P1_S1;P1_S2;P1_S3;P1_S5;P2_S1;P2_S2;P4_	Complete;Com	*EFCAB6*		1	0
chr22:44,210,031-44,244,803	34,772	q13.31	90	4	AI;AI;AI;A	P1_S1;P1_S2;P1_S3;P2_S1;P2_S2;P4_S1;P4_	Complete;Com	*AX747137, SULT4A1*		2	0
chr22:44,750,849-44,763,701	12,852	q13.31	90	0	AI;AI;AI;A	P1_S1;P1_S2;P1_S3;P2_S1;P2_S2;P4_S1;P4_	Complete;Complete;Complete;Comple			0	0
chr22:44,775,215-44,894,780	119,565	q13.31	90	0	AI;AI;AI;A	P1_S1;P1_S2;P1_S3;P2_S1;P2_S2;P4_S1;P4_	Complete;Com	*LINC01656, LDOC1L*		3	0

chr22:44,971,134-45,086,096	114,962	q13.31	90	53	AI;AI;AI;A	P1_S1;P1_S2;P1_S3;P2_S1;P2_S2;P4_S1;P4	Complete;Com	*LINC00229, PRR5*		2	0
chr22:45,110,950-45,130,687	19,737	q13.31	90	100	AI;AI;AI;A	P1_S1;P1_S2;P1_S3;P2_S1;P2_S2;P4_S1;P4	Complete;Com	*PRR5, PRR5-ARHGAI*		2	0
chr22:47,119,026-47,123,459	4,433	q13.31	90	0	AI;AI;AI;A	P1_S1;P1_S2;P1_S3;P2_S1;P2_S2;P4_S1;P4	Complete;Com	*CERK*		1	0
chr22:47,182,113-47,299,702	117,589	q13.31	90	2	AI;AI;AI;A	P1_S1;P1_S2;P1_S3;P2_S1;P2_S2;P4_S1;P4	Complete;Com	*TBC1D22A*		1	0
chr22:48,085,371-48,113,129	27,758	q13.31	90	0	AI;AI;AI;A	P1_S1;P1_S2;P1_S3;P2_S1;P2_S2;P4_S1;P4	Complete;Com	*LOC284930, AK09310*		2	0
chr22:48,186,567-48,257,077	70,510	q13.31	90	0	AI;AI;AI;A	P1_S1;P1_S2;P1_S3;P2_S1;P2_S2;P4_S1;P4	Complete;Com	*LOC284930, AK09310*		2	0
chr22:48,308,382-48,454,046	145,664	3.31 - q13.	90	1	AI;AI;AI;A	P1_S1;P1_S2;P1_S3;P2_S1;P2_S2;P4_S1;P4	Complete;Complete;Complete;Comple			0	0
chr22:48,620,417-48,695,781	75,364	q13.32	90	7	AI;AI;AI;A	P1_S1;P1_S2;P1_S3;P2_S1;P2_S2;P4_S1;P4	Complete;Com	*MIR3201*	hsa-mir-32	1	1
chr22:48,810,607-48,844,468	33,861	q13.32	90	17	AI;AI;AI;A	P1_S1;P1_S2;P1_S3;P2_S1;P2_S2;P4_S1;P4	Complete;Complete;Complete;Comple			0	0
chr22:48,921,561-49,054,548	132,987	q13.32	90	99	AI;AI;AI;A	P1_S1;P1_S2;P1_S3;P2_S1;P2_S2;P4_S1;P4	Complete;Com	*LOC284933, FAM19A*		2	0
chr22:49,061,794-49,335,462	273,668	q13.32	90	23	AI;AI;AI;A	P1_S1;P1_S2;P1_S3;P2_S1;P2_S2;P4_S1;P4	Complete;Com	*FAM19A5, MIR4535,*		4	0
chr22:49,396,462-49,397,269	807	q13.32	90	100	AI;AI;AI;A	P1_S1;P1_S2;P1_S3;P2_S1;P2_S2;P4_S1;P4	Complete;Complete;Complete;Comple			0	0
chr22:49,591,344-49,595,173	3,829	q13.33	90	100	AI;AI;AI;A	P1_S1;P1_S2;P1_S3;P2_S1;P2_S2;P4_S1;P4	Complete;Complete;Complete;Comple			0	0
chr22:49,715,245-49,767,001	51,756	q13.33	90	100	AI;AI;AI;A	P1_S1;P1_S2;P1_S3;P2_S1;P2_S2;P4_S1;P4	Complete;Complete;Complete;Comple			0	0
chr22:50,919,714-50,921,887	2,173	q13.33	90	0	Loss;Loss;L	P1_S1;P1_S2;P1_S3;P1_S5;P2_S1;P2_S2;P4	Complete;Com	*ADM2*		1	0
chr7:142,307,376-142,445,944	138,568	q34	90	86	Gain;Gain;G	P1_S1;P1_S2;P1_S3;P1_S5;P2_S1;P2_S2;P4	Complete;Com	*TCRBV5S1A1T, TCRB*		17	0
chr22:18,338,118-18,536,402	198,284	q11.21	80	100	AI;AI;AI;A	P1_S1;P1_S3;P1_S5;P2_S1;P2_S2;P4_S1;P4	Complete;Com	*MICAL3, M*	hsa-mir-64	4	1
chr22:19,717,933-19,789,597	71,664	q11.21	80	100	AI;AI;AI;A	P1_S1;P1_S3;P1_S5;P2_S1;P4_S1;P4_S2;P4	Complete;Com	*TBX1, GNB1L*		2	0
chr22:19,808,487-19,875,935	67,448	q11.21	80	23	AI;AI;AI;A	P1_S1;P1_S3;P1_S5;P2_S1;P4_S1;P4_S2;P4	Complete;Com	*GNB1L, RTL10, C22o*		4	0
chr22:19,951,537-19,958,199	6,662	q11.21	80	100	AI;AI;AI;A	P1_S1;P1_S3;P1_S5;P2_S1;P4_S1;P4_S2;P4	Complete;Com	*COMT, ARVCF*		2	0
chr22:20,211,999-20,937,988	725,989	q11.21	80	86	AI;AI;AI;A	P1_S1;P1_S3;P1_S5;P2_S1;P2_S2;P4_S1;P4	Complete;Com	*MIR1286, R*	hsa-mir-12	25	1
chr22:21,988,374-21,988,963	589	q11.21	80	100	Loss;Loss;L	P1_S1;P1_S3;P1_S5;P2_S1;P2_S2;P4_S2;P4	Complete;Com	*CCDC116*		1	0
chr22:22,298,200-22,316,816	18,616	q11.22	80	100	Loss;Loss;L	P1_S1;P1_S3;P1_S5;P2_S1;P2_S2;P4_S2;P4	Complete;Com	*PPM1F, TOP3B*		2	0
chr22:23,318,822-23,622,335	303,513	1.22 - q11.	80	21	AI;AI;AI;A	P1_S1;P1_S2;P1_S3;P2_S1;P2_S2;P4_S1;P4	Complete;Com	*GNAZ, RSPH14, RTD*		6	0
chr22:24,087,107-24,097,963	10,856	q11.23	80	100	AI;AI;AI;A	P1_S1;P1_S2;P2_S1;P2_S2;P4_S1;P4_S2;P4	Complete;Com	*ZNF70, VPREB3*		2	0
chr22:24,817,914-24,879,433	61,519	q11.23	80	100	Loss;Loss;L	P1_S1;P1_S2;P1_S3;P1_S5;P2_S1;P2_S2;P4	Complete;Com	*SPECC1L, SPECC1L-*		6	0
chr22:24,879,433-24,888,328	8,895	q11.23	80	100	AI;AI;AI;A	P1_S1;P1_S2;P1_S3;P2_S1;P2_S2;P4_S1;P4	Complete;Com	*ADORA2A-AS1, UPB1*		2	0
chr22:25,469,843-25,593,759	123,916	q11.23	80	100	AI;AI;AI;A	P1_S1;P1_S2;P2_S1;P2_S2;P4_S1;P4_S2;P4	Complete;Com	*LOC100128531, KIAA*		2	0
chr22:25,593,759-25,599,778	6,019	q11.23	80	100	Loss;Loss;L	P1_S1;P1_S2;P1_S3;P1_S5;P2_S1;P2_S2;P4	Complete;Com	*CRYBB3*		1	0
chr22:25,599,778-25,599,823	45	q11.23	80	100	AI;AI;AI;A	P1_S1;P1_S2;P2_S1;P2_S2;P4_S1;P4_S2;P4	Complete;Com	*CRYBB3*		1	0
chr22:25,908,822-26,160,351	251,529	q12.1	80	96	AI;AI;AI;A	P1_S1;P1_S2;P1_S3;P2_S1;P2_S2;P4_S1;P4	Complete;Com	*GRK3, ADRBK2, MYO*		3	0
chr22:26,174,472-26,191,425	16,953	q12.1	80	0	Loss;Loss;L	P1_S1;P1_S2;P1_S3;P1_S5;P2_S1;P2_S2;P4	Complete;Com	*MYO18B*		1	0
chr22:26,343,814-26,403,055	59,241	q12.1	80	0	Loss;Loss;L	P1_S1;P1_S2;P1_S3;P1_S5;P2_S1;P2_S2;P4	Complete;Com	*MYO18B*		1	0
chr22:26,563,648-26,591,989	28,341	q12.1	80	0	Loss;Loss;L	P1_S1;P1_S2;P1_S3;P1_S5;P2_S1;P2_S2;P4	Complete;Com	*SEZ6L*		1	0
chr22:26,859,906-26,887,868	27,962	q12.1	80	0	Loss;Loss;L	P1_S1;P1_S3;P1_S5;P2_S1;P2_S2;P4_S2;P4	Complete;Com	*HPS4, SRRD, TFIP11*		3	0
chr22:26,943,707-26,963,346	19,639	q12.1	80	33	Loss;Loss;L	P1_S1;P1_S2;P1_S3;P1_S5;P2_S1;P2_S2;P4	Complete;Com	*MIR548J, T*	hsa-mir-54	2	1
chr22:27,182,852-27,206,027	23,175	q12.1	80	0	AI;AI;AI;A	P1_S1;P1_S2;P2_S1;P2_S2;P4_S1;P4_S2;P4	Complete;Complete;Complete;Comple			0	0
chr22:27,990,011-28,262,876	272,865	q12.1	80	6	AI;AI;AI;A	P1_S1;P1_S2;P2_S1;P2_S2;P4_S1;P4_S2;P4	Complete;Com	*MN1, PITPNB*		2	0
chr22:28,262,876-28,371,513	108,637	q12.1	80	0	Loss;Loss;L	P1_S1;P1_S2;P1_S3;P1_S5;P2_S1;P2_S2;P4	Complete;Com	*AX748308,*	hsa-mir-3	5	2
chr22:28,371,513-28,376,047	4,534	q12.1	80	0	AI;AI;AI;A	P1_S1;P1_S2;P2_S1;P2_S2;P4_S1;P4_S2;P4	Complete;Com	*TTC28-AS1, TTC28*		2	0
chr22:28,666,035-28,756,994	90,959	q12.1	80	0	Loss;Loss;L	P1_S1;P1_S3;P1_S5;P2_S1;P2_S2;P4_S2;P4	Complete;Com	*TTC28*		1	0
chr22:28,769,854-28,899,874	130,020	q12.1	80	0	Loss;Loss;L	P1_S1;P1_S2;P1_S3;P1_S5;P2_S1;P2_S2;P4	Complete;Com	*TTC28, MIR5739*		2	0
chr22:29,042,413-29,445,679	403,266	q12.1	80	10	AI;AI;AI;A	P1_S1;P1_S2;P1_S3;P2_S1;P2_S2;P4_S1;P4	Complete;Com	*TTC28, CHEK2, HSC*		7	0
chr22:29,453,752-29,455,735	1,983	q12.1	80	0	Loss;Loss;L	P1_S1;P1_S3;P1_S5;P2_S1;P2_S2;P4_S2;P4	Complete;Com	*C22orf31*		1	0
chr22:29,455,735-29,530,684	74,949	q12.1	80	7	AI;AI;AI;A	P1_S1;P1_S2;P1_S3;P2_S1;P2_S2;P4_S1;P4	Complete;Com	*C22orf31, KREMEN1*		2	0
chr22:29,568,197-29,638,831	70,634	12.1 - q12.	80	4	AI;AI;AI;A	P1_S1;P1_S2;P1_S3;P2_S1;P2_S2;P4_S1;P4	Complete;Com	*EMID1*		1	0
chr22:29,638,831-29,660,647	21,816	q12.2	80	0	Loss;Loss;L	P1_S1;P1_S2;P1_S3;P1_S5;P2_S1;P2_S2;P4	Complete;Com	*EMID1, RHBDD3*		2	0

chr22:29,660,647-29,703,389	42,742	q12.2	80	10	AI;AI;AI;A	P1_S1;P1_S2;P1_S3;P2_S1;P2_S2;P4_S1;P4	Complete;Com	*RHBDD3, EWSR1, GA*		3	0
chr22:30,374,978-30,403,968	28,990	q12.2	80	0	Loss;Loss;L	P1_S1;P1_S3;P1_S5;P2_S1;P2_S2;P4_S1;P4	Complete;Com	*MTMR3, MIR6818*		2	0
chr22:30,403,968-30,415,960	11,992	q12.2	80	0	AI;AI;AI;A	P1_S1;P1_S2;P1_S3;P2_S1;P2_S2;P4_S1;P4	Complete;Com	*MTMR3, HORMAD2-*		2	0
chr22:30,416,408-30,424,172	7,764	q12.2	80	0	AI;AI;AI;A	P1_S1;P1_S2;P1_S3;P2_S1;P2_S2;P4_S1;P4	Complete;Com	*MTMR3, HORMAD2-*		2	0
chr22:30,592,664-30,766,431	173,767	q12.2	80	0	AI;AI;AI;A	P1_S1;P1_S2;P1_S3;P2_S1;P2_S2;P4_S1;P4	Complete;Com	*LIF-AS1, LIF, LOC91*		11	0
chr22:30,767,247-30,772,571	5,324	q12.2	80	0	AI;AI;AI;A	P1_S1;P1_S2;P1_S3;P2_S1;P2_S2;P4_S1;P4	Complete;Com	*CCDC157, KIAA1656*		2	0
chr22:30,778,142-30,900,705	122,563	q12.2	80	0	AI;AI;AI;A	P1_S1;P1_S2;P1_S3;P2_S1;P2_S2;P4_S1;P4	Complete;Com	*RNF215, SEC14L2, M*		7	0
chr22:31,006,967-31,011,630	4,663	q12.2	80	0	Loss;Loss;L	P1_S1;P1_S2;P1_S3;P1_S5;P2_S1;P2_S2;P4	Complete;Com	*TCN2*		1	0
chr22:31,238,551-31,258,819	20,268	q12.2	80	75	Loss;Loss;L	P1_S1;P1_S3;P1_S5;P2_S1;P2_S2;P4_S2;P4	Complete;Com	*OSBP2*		1	0
chr22:32,825,262-32,851,321	26,059	q12.3	80	0	Loss;Loss;L	P1_S1;P1_S2;P1_S3;P1_S5;P2_S1;P2_S2;P4	Complete;Com	*BPIFC*		1	0
chr22:33,402,723-33,496,313	93,590	q12.3	80	0	Loss;Loss;L	P1_S1;P1_S2;P1_S3;P1_S5;P2_S1;P2_S2;P4	Complete;Com	*SYN3*		1	0
chr22:33,528,057-33,605,693	77,636	q12.3	80	0	Loss;Loss;L	P1_S1;P1_S3;P1_S5;P2_S1;P2_S2;P4_S2;P4	Complete;Complete;Complete;Comple			0	0
chr22:33,728,899-33,777,229	48,330	q12.3	80	16	Loss;Loss;L	P1_S1;P1_S2;P1_S3;P1_S5;P2_S1;P2_S2;P4	Complete;Com	*LARGE1, LARGE*		2	0
chr22:34,516,763-34,664,494	147,731	q12.3	80	0	Loss;Loss;L	P1_S1;P1_S2;P1_S3;P1_S5;P2_S1;P2_S2;P4	Complete;Complete;Complete;Comple			0	0
chr22:34,859,206-35,094,396	235,190	q12.3	80	5	Loss;Loss;L	P1_S1;P1_S2;P1_S3;P1_S5;P2_S1;P2_S2;P4	Complete;Complete;Complete;Comple			0	0
chr22:35,563,613-35,589,939	26,326	q12.3	80	30	Loss;Loss;L	P1_S1;P1_S3;P1_S5;P2_S1;P2_S2;P4_S2;P4	Complete;Com	*LINC01399*		1	0
chr22:36,184,002-36,376,190	192,188	q12.3	80	43	Loss;Loss;L	P1_S1;P1_S2;P1_S3;P1_S5;P2_S1;P2_S2;P4	Complete;Com	*RBFOX2*		1	0
chr22:36,865,042-36,891,105	26,063	q12.3	80	100	AI;AI;AI;A	P1_S1;P1_S2;P2_S1;P2_S2;P4_S1;P4_S2;P4	Complete;Com	*TXN2, FOXRED2*		2	0
chr22:37,061,685-37,071,926	10,241	q12.3	80	0	Loss;Loss;L	P1_S1;P1_S2;P1_S3;P1_S5;P2_S1;P2_S2;P4	Complete;Com	*CACNG2*		1	0
chr22:37,395,954-37,414,754	18,800	q12.3	80	0	Loss;Loss;L	P1_S1;P1_S2;P1_S3;P1_S5;P2_S1;P2_S2;P4	Complete;Com	*TEX33, TST*		2	0
chr22:37,426,965-37,470,716	43,751	q12.3	80	1	AI;AI;AI;A	P1_S1;P1_S2;P1_S3;P2_S1;P4_S1;P4_S2;P4	Complete;Com	*KCTD17, TMPRSS6*		2	0
chr22:38,240,811-38,308,397	67,586	q13.1	80	38	Loss;Loss;L	P1_S1;P1_S2;P1_S3;P1_S5;P2_S1;P2_S2;P4	Complete;Com	*ANKRD54,*	hsa-mir-6	4	1
chr22:39,276,559-39,306,355	29,796	q13.1	80	39	Loss;Loss;L	P1_S1;P1_S2;P1_S3;P1_S5;P2_S1;P2_S2;P4	Complete;Complete;Complete;Comple			0	0
chr22:39,927,188-39,970,151	42,963	q13.1	80	22	Loss;Loss;L	P1_S1;P1_S3;P1_S5;P2_S1;P2_S2;P4_S2;P4	Complete;Com	*RPS19BP1, CACNA1I*		2	0
chr22:42,339,600-42,453,880	114,280	q13.2	80	11	Loss;Loss;L	P1_S1;P1_S2;P1_S3;P1_S5;P2_S1;P2_S2;P4	Complete;Com	*bK250D10.C22.8, CE*		5	0
chr22:43,194,487-43,543,192	348,705	q13.2	80	12	AI;AI;AI;A	P1_S2;P1_S3;P2_S1;P2_S2;P4_S1;P4_S2;P4	Complete;Com	*ARFGAP3, PACSIN2,*		7	0
chr22:43,656,786-44,073,976	417,190	q13.2	80	18	AI;AI;AI;A	P1_S1;P1_S2;P2_S1;P2_S2;P4_S1;P4_S2;P4	Complete;Com	*SCUBE1, LOC101927*		7	0
chr22:44,282,368-44,318,201	35,833	q13.31	80	0	Loss;Loss;L	P1_S1;P1_S3;P1_S5;P2_S1;P2_S2;P4_S2;P4	Complete;Com	*PNPLA5*		1	0
chr22:44,324,747-44,495,208	170,461	q13.31	80	7	AI;AI;AI;A	P1_S1;P1_S2;P1_S3;P2_S2;P4_S1;P4_S2;P4	Complete;Com	*PNPLA3, SAMM50, A*		4	0
chr22:44,763,701-44,775,215	11,514	q13.31	80	0	Loss;Loss;L	P1_S1;P1_S2;P1_S3;P1_S5;P2_S1;P2_S2;P4	Complete;Complete;Complete;Comple			0	0
chr22:45,192,062-45,258,269	66,207	q13.31	80	57	AI;AI;AI;A	P1_S1;P1_S2;P2_S1;P2_S2;P4_S1;P4_S2;P4	Complete;Com	*PRR5-ARHGAP8, AR*		2	0
chr22:45,418,544-45,550,785	132,241	q13.31	80	100	AI;AI;AI;A	P1_S1;P1_S2;P1_S3;P2_S1;P4_S1;P4_S2;P4	Complete;Com	*NUP50-DT, LOC1005*		2	0
chr22:45,585,325-45,622,341	37,016	q13.31	80	0	AI;AI;AI;A	P1_S1;P1_S2;P1_S3;P2_S1;P4_S1;P4_S2;P4	Complete;Com	*LOC10537*	hsa-mir-1	3	1
chr22:45,657,469-45,691,537	34,068	q13.31	80	100	AI;AI;AI;A	P1_S1;P1_S2;P1_S3;P2_S1;P4_S1;P4_S2;P4	Complete;Com	*UPK3A*		1	0
chr22:45,721,537-45,750,617	29,080	q13.31	80	100	AI;AI;AI;A	P1_S1;P1_S2;P2_S1;P2_S2;P4_S1;P4_S2;P4	Complete;Com	*FAM118A, DQ586951*		3	0
chr22:45,753,522-45,813,664	60,142	q13.31	80	100	Loss;Loss;L	P1_S1;P1_S3;P1_S5;P2_S1;P2_S2;P4_S2;P4	Complete;Com	*SMC1B, RIBC2*		2	0
chr22:45,813,664-45,821,992	8,328	q13.31	80	100	AI;AI;AI;A	P1_S1;P1_S2;P2_S1;P2_S2;P4_S1;P4_S2;P4	Complete;Com	*RIBC2*		1	0
chr22:45,864,757-45,968,777	104,020	q13.31	80	47	AI;AI;AI;A	P1_S1;P1_S2;P2_S1;P2_S2;P4_S1;P4_S2;P4	Complete;Com	*FBLN1*		1	0
chr22:46,022,842-46,507,299	484,457	q13.31	80	29	AI;AI;AI;A	P1_S1;P1_S2;P2_S1;P2_S2;P4_S1;P4_S2;P4	Complete;Com	*ATXN10, MIR4762, W*		12	0
chr22:46,507,299-46,588,194	80,895	q13.31	80	46	Loss;Loss;L	P1_S1;P1_S3;P1_S5;P2_S1;P2_S2;P4_S2;P4	Complete;Com	*MIRLET7B*	hsa-let-7a	6	2
chr22:46,706,492-46,722,521	16,029	q13.31	80	31	Loss;Loss;L	P1_S1;P1_S3;P1_S5;P2_S1;P2_S2;P4_S2;P4	Complete;Com	*GTSE1*		1	0
chr22:46,759,965-46,920,341	160,376	q13.31	80	1	AI;AI;AI;A	P1_S1;P1_S2;P2_S1;P2_S2;P4_S1;P4_S2;P4	Complete;Com	*CELSR1*		1	0
chr22:47,456,787-47,823,424	366,637	q13.31	80	44	AI;AI;AI;A	P1_S1;P1_S2;P2_S1;P2_S2;P4_S1;P4_S2;P4	Complete;Com	*TBC1D22A, LOC3396*		2	0
chr22:48,113,129-48,186,567	73,438	q13.31	80	0	Loss;Loss;L	P1_S1;P1_S2;P1_S3;P1_S5;P2_S1;P2_S2;P4	Complete;Com	*LOC284930, AK09310*		2	0
chr22:48,509,627-48,530,239	20,612	q13.32	80	21	Loss;Loss;L	P1_S1;P1_S2;P1_S3;P1_S5;P2_S1;P2_S2;P4	Complete;Complete;Complete;Comple			0	0
chr22:48,844,895-48,893,826	48,931	q13.32	80	90	Loss;Loss;L	P1_S1;P1_S2;P1_S3;P1_S5;P2_S1;P2_S2;P4	Complete;Com	*FAM19A5*		1	0
chr22:49,541,819-49,574,557	32,738	q13.33	80	100	AI;AI;AI;A	P1_S1;P1_S2;P1_S3;P2_S1;P2_S2;P4_S1;P4	Complete;Complete;Complete;Comple			0	0

chr22:49,595,173-49,658,538	63,365	q13.33	80	100	Loss;Loss;L	P1_S1;P1_S3;P1_S5;P2_S1;P2_S2;P4_S2;P4_	Complete;Complete;Complete;Comple			0	0
chr22:49,691,634-49,715,245	23,611	q13.33	80	100	Loss;Loss;L	P1_S1;P1_S3;P1_S5;P2_S1;P2_S2;P4_S2;P4_	Complete;Complete;Complete;Comple			0	0
chr22:49,827,749-50,181,057	353,308	q13.33	80	59	AI;AI;AI;A	P1_S1;P1_S2;P2_S1;P2_S2;P4_S1;P4_S2;P4_	Complete;Com	*C22orf34, BC033837,*		5	0
chr22:50,684,771-50,685,870	1,099	q13.33	80	0	Loss;Loss;L	P1_S1;P1_S2;P1_S3;P1_S5;P2_S1;P2_S2;P4_	Complete;Com	*HDAC10*		1	0
chr22:17,445,606-17,581,030	135,424	q11.1	70	100	AI;AI;AI;A	P1_S1;P1_S3;P1_S5;P2_S1;P2_S2;P4_S1;P4_	Complete;Com	*GAB4, CECR7, IL17R*		3	0
chr22:21,343,232-21,424,931	81,699	q11.21	70	100	AI;AI;AI;A	P1_S1;P1_S5;P2_S1;P4_S1;P4_S2;P4_S3;P5_	Complete;Com	*LZTR1, TH*	hsa-mir-6	12	1
chr22:21,799,313-21,807,793	8,480	q11.21	70	100	AI;AI;AI;A	P1_S1;P1_S2;P2_S1;P4_S1;P4_S2;P4_S3;P5_	Complete;Com	*HIC2*		1	0
chr22:23,503,731-23,622,335	118,604	q11.23	70	50	Loss;Loss;L	P1_S1;P1_S3;P1_S5;P2_S1;P2_S2;P4_S2;P5_	Complete;Com	*RAB36, BCR, FBXW4*		3	0
chr22:23,739,091-24,044,359	305,268	q11.23	70	100	Loss;Loss;L	P1_S1;P1_S3;P1_S5;P2_S1;P2_S2;P4_S2;P5_	Complete;Com	*ZDHHC8P1, LINC01*		10	0
chr22:24,097,963-24,135,056	37,093	q11.23	70	100	Loss;Loss;L	P1_S1;P1_S2;P1_S3;P1_S5;P2_S1;P2_S2;P5_	Complete;Com	*C22orf15, CHCHD10,*		4	0
chr22:24,984,260-25,158,441	174,181	q11.23	70	56	AI;AI;AI;A	P1_S1;P1_S2;P1_S3;P2_S1;P2_S2;P4_S1;P5_	Complete;Com	*FAM211B, LRRC75B,*		7	0
chr22:25,202,199-25,209,061	6,862	q11.23	70	0	AI;AI;AI;A	P1_S1;P1_S2;P1_S3;P2_S1;P2_S2;P4_S1;P4_	Complete;Com	*SGSM1*		1	0
chr22:25,212,862-25,231,496	18,634	q11.23	70	0	AI;AI;AI;A	P1_S1;P1_S2;P2_S1;P2_S2;P4_S1;P4_S2;P4_	Complete;Com	*SGSM1*		1	0
chr22:25,254,081-25,290,781	36,700	q11.23	70	1	Loss;Loss;L	P1_S1;P1_S3;P1_S5;P2_S1;P2_S2;P4_S2;P5_	Complete;Com	*SGSM1*		1	0
chr22:25,610,592-25,663,066	52,474	q11.23	70	100	Loss;Loss;L	P1_S1;P1_S3;P1_S5;P2_S1;P2_S2;P4_S2;P5_	Complete;Com	*CRYBB2*		1	0
chr22:25,786,192-26,160,351	374,159	11.23 - q12	70	97	Loss;Loss;L	P1_S1;P1_S3;P1_S5;P2_S1;P2_S2;P4_S2;P5_	Complete;Com	*MIR6817, CRYBB2P1*		5	0
chr22:26,428,062-26,444,466	16,404	q12.1	70	0	Loss;Loss;L	P1_S1;P1_S3;P1_S5;P2_S1;P2_S2;P4_S2;P5_	Complete;Complete;Complete;Comple			0	0
chr22:26,638,419-26,706,137	67,718	q12.1	70	0	AI;AI;AI;A	P1_S1;P1_S2;P1_S3;P2_S1;P2_S2;P4_S1;P4_	Complete;Com	*SEZ6L*		1	0
chr22:27,113,719-27,171,181	57,462	q12.1	70	3	Loss;Loss;L	P1_S1;P1_S3;P1_S5;P2_S1;P2_S2;P4_S2;P5_	Complete;Com	*AK026502, MIATNB*		2	0
chr22:27,340,211-27,395,497	55,286	q12.1	70	2	Loss;Loss;L	P1_S1;P1_S3;P1_S5;P2_S1;P2_S2;P4_S2;P5_	Complete;Complete;Complete;Comple			0	0
chr22:27,500,135-27,646,823	146,688	q12.1	70	0	Loss;Loss;L	P1_S1;P1_S3;P1_S5;P2_S1;P2_S2;P4_S2;P5_	Complete;Com	*LOC105372977, LINC*		3	0
chr22:27,715,526-27,796,145	80,619	q12.1	70	0	Loss;Loss;L	P1_S1;P1_S3;P1_S5;P2_S1;P2_S2;P4_S2;P5_	Complete;Complete;Complete;Comple			0	0
chr22:27,890,471-27,900,579	10,108	q12.1	70	0	Loss;Loss;L	P1_S1;P1_S3;P1_S5;P2_S1;P2_S2;P4_S2;P5_	Complete;Complete;Complete;Comple			0	0
chr22:27,955,271-27,990,011	34,740	q12.1	70	0	Loss;Loss;L	P1_S1;P1_S3;P1_S5;P2_S1;P2_S2;P4_S2;P5_	Complete;Complete;Complete;Comple			0	0
chr22:29,740,801-29,859,055	118,254	q12.2	70	38	AI;AI;AI;A	P1_S1;P1_S2;P1_S3;P2_S1;P2_S2;P4_S2;P4_	Complete;Com	*AP1B1, RFPL1S, RFP*		3	0
chr22:29,908,113-29,922,822	14,709	q12.2	70	0	Loss;Loss;L	P1_S1;P1_S3;P1_S5;P2_S1;P2_S2;P4_S2;P5_	Complete;Com	*THOC5*		1	0
chr22:30,772,571-30,778,142	5,571	q12.2	70	0	Loss;Loss;L	P1_S1;P1_S2;P1_S3;P1_S5;P2_S1;P2_S2;P5_	Complete;Com	*CCDC157, KIAA1656*		3	0
chr22:30,900,705-30,977,183	76,478	q12.2	70	2	Loss;Loss;L	P1_S1;P1_S3;P1_S5;P2_S1;P2_S2;P4_S2;P5_	Complete;Com	*SEC14L4, SEC14L6,*		4	0
chr22:31,011,630-31,117,945	106,315	q12.2	70	3	AI;AI;AI;A	P1_S1;P1_S2;P1_S3;P2_S1;P2_S2;P4_S1;P4_	Complete;Com	*TCN2, SLC35E4, DUS*		4	0
chr22:31,258,819-31,277,035	18,216	q12.2	70	21	AI;AI;AI;A	P1_S1;P1_S2;P1_S3;P2_S1;P2_S2;P4_S1;P4_	Complete;Com	*OSBP2*		1	0
chr22:31,500,763-31,522,142	21,379	q12.2	70	27	Loss;Loss;L	P1_S1;P1_S3;P1_S5;P2_S1;P2_S2;P4_S2;P5_	Complete;Com	*SELM, SELENOM, IN*		3	0
chr22:31,535,964-31,658,663	122,699	q12.2	70	2	AI;AI;AI;A	P1_S1;P1_S2;P2_S1;P2_S2;P4_S1;P4_S2;P4_	Complete;Com	*PLA2G3, MIR3928, R*		6	0
chr22:31,658,663-31,687,067	28,404	q12.2	70	5	Loss;Loss;L	P1_S1;P1_S3;P1_S5;P2_S1;P2_S2;P4_S2;P5_	Complete;Com	*LIMK2, PIK3IP1*		2	0
chr22:31,875,968-31,977,167	101,199	q12.2	70	5	AI;AI;AI;A	P1_S1;P1_S2;P2_S1;P2_S2;P4_S1;P4_S2;P4_	Complete;Com	*EIF4ENIF1, SFI1*		2	0
chr22:31,977,167-32,015,306	38,139	q12.2	70	99	Loss;Loss;L	P1_S1;P1_S3;P1_S5;P2_S1;P2_S2;P4_S2;P4_	Complete;Com	*SFI1, PISD*		2	0
chr22:32,601,816-32,630,985	29,169	q12.3	70	0	Loss;Loss;L	P1_S1;P1_S3;P1_S5;P2_S1;P2_S2;P4_S2;P5_	Complete;Com	*SLC5A4, SLC5A4-AS1*		2	0
chr22:34,014,899-34,134,002	119,103	q12.3	70	1	Loss;Loss;L	P1_S1;P1_S3;P1_S5;P2_S1;P2_S2;P4_S2;P5_	Complete;Com	*LARGE1, LARGE, SN*		5	0
chr22:35,754,262-35,779,143	24,881	q12.3	70	33	Loss;Loss;L	P1_S1;P1_S3;P1_S5;P2_S1;P2_S2;P4_S2;P5_	Complete;Com	*HMOX1*		1	0
chr22:35,813,220-35,910,928	97,708	q12.3	70	0	Loss;Loss;L	P1_S1;P1_S3;P1_S5;P2_S1;P2_S2;P4_S2;P5_	Complete;Com	*MCM5*		1	0
chr22:36,056,117-36,122,360	66,243	q12.3	70	33	Loss;Loss;L	P1_S1;P1_S3;P1_S5;P2_S1;P2_S2;P4_S2;P5_	Complete;Com	*APOL6, APOL5*		2	0
chr22:36,593,895-36,652,547	58,652	q12.3	70	67	Loss;Loss;L	P1_S1;P1_S3;P1_S5;P2_S1;P2_S2;P4_S2;P5_	Complete;Com	*APOL4, APOL2, AX7*		4	0
chr22:36,761,015-36,865,042	104,027	q12.3	70	75	Loss;Loss;L	P1_S1;P1_S3;P1_S5;P2_S1;P2_S2;P4_S2;P5_	Complete;Com	*MYH9, TXN2*		2	0
chr22:36,900,790-37,022,707	121,917	q12.3	70	93	AI;AI;AI;A	P1_S1;P1_S2;P2_S1;P4_S1;P4_S2;P4_S3;P5_	Complete;Com	*FOXRED2, EIF3D, C*		3	0
chr22:37,098,180-37,247,103	148,923	q12.3	70	83	AI;AI;AI;A	P1_S1;P1_S2;P2_S1;P4_S1;P4_S2;P4_S3;P5_	Complete;Com	*CACNG2, AK123632,*		7	0
chr22:37,247,103-37,251,427	4,324	q12.3	70	100	Loss;Loss;L	P1_S1;P1_S3;P1_S5;P2_S1;P2_S2;P4_S2;P5_	Complete;Com	*NCF4-AS1*		1	0
chr22:37,285,952-37,395,954	110,002	q12.3	70	68	AI;AI;AI;A	P1_S1;P1_S2;P1_S3;P2_S1;P4_S1;P4_S3;P5_	Complete;Com	*CSF2RB, LOC100506*		4	0
chr22:37,291,718-37,367,732	76,014	q12.3	70	91	Loss;Loss;L	P1_S1;P1_S3;P1_S5;P2_S1;P2_S2;P4_S2;P5_	Complete;Com	*CSF2RB, LOC100506*		3	0

chr22:37,576,965-37,679,717	102,752	12.3 - q13.	70	7	Loss;Loss;L	P1_S1;P1_S3;P1_S5;P2_S1;P2_S2;P4_S2;P5_	Complete;Com	*C1QTNF6, SSTR3, RA*	4	0
chr22:37,871,859-37,911,602	39,743	q13.1	70	2	Loss;Loss;L	P1_S1;P1_S3;P1_S5;P2_S1;P2_S2;P4_S2;P5_	Complete;Com	*MFNG, CARD10*	2	0
chr22:38,085,192-38,119,242	34,050	q13.1	70	100	Loss;Loss;L	P1_S1;P1_S3;P1_S5;P2_S1;P2_S2;P4_S2;P5_	Complete;Com	*NOL12, TRIOBP*	2	0
chr22:38,129,915-38,168,876	38,961	q13.1	70	100	Loss;Loss;L	P1_S1;P1_S3;P1_S5;P2_S1;P2_S2;P4_S2;P5_	Complete;Com	*TRIOBP*	1	0
chr22:38,348,816-38,518,580	169,764	q13.1	70	2	Loss;Loss;L	P1_S1;P1_S3;P1_S5;P2_S1;P2_S2;P4_S2;P5_	Complete;Com	*C22orf23, MIR6820, S*	10	0
chr22:38,994,343-39,130,336	135,993	q13.1	70	5	AI;AI;AI;A	P1_S2;P1_S3;P2_S1;P2_S2;P4_S1;P4_S2;P4	Complete;Com	*FAM227A, CBY1, BC*	6	0
chr22:39,130,336-39,136,368	6,032	q13.1	70	100	Loss;Loss;L	P1_S1;P1_S3;P1_S5;P2_S1;P2_S2;P4_S2;P5_	Complete;Com	*SUN2*	1	0
chr22:39,136,368-39,145,301	8,933	q13.1	70	100	AI;AI;AI;A	P1_S2;P1_S3;P2_S1;P2_S2;P4_S1;P4_S2;P4	Complete;Com	*SUN2*	1	0
chr22:39,394,046-39,413,725	19,679	q13.1	70	100	Loss;Loss;L	P1_S1;P1_S3;P1_S5;P2_S1;P2_S2;P4_S2;P5_	Complete;Com	*APOBEC3B-AS1, APC*	2	0
chr22:39,586,009-39,615,218	29,209	q13.1	70	2	Loss;Loss;L	P1_S1;P1_S3;P1_S5;P2_S1;P2_S2;P4_S3;P5_	Complete;Complete;Complete;Comple		0	0
chr22:40,032,285-40,305,518	273,233	q13.1	70	33	Loss;Loss;L	P1_S1;P1_S3;P1_S5;P2_S1;P2_S2;P4_S2;P5_	Complete;Com	*CACNA1I, ENTHD1,*	3	0
chr22:40,305,518-40,417,643	112,125	q13.1	70	5	AI;AI;AI;A	P1_S2;P1_S3;P2_S1;P2_S2;P4_S1;P4_S2;P4	Complete;Com	*GRAP2, FAM83F*	2	0
chr22:40,417,643-40,501,218	83,575	q13.1	70	6	Loss;Loss;L	P1_S1;P1_S3;P1_S5;P2_S1;P2_S2;P4_S2;P5_	Complete;Com	*FAM83F, LOC100130*	3	0
chr22:40,662,816-40,710,263	47,447	q13.1	70	0	Loss;Loss;L	P1_S1;P1_S3;P1_S5;P2_S1;P2_S2;P4_S2;P5_	Complete;Com	*TNRC6B*	1	0
chr22:40,752,588-40,805,711	53,123	q13.1	70	2	Loss;Loss;L	P1_S1;P1_S3;P1_S5;P2_S1;P2_S2;P4_S2;P5_	Complete;Com	*ADSL, SGSM3*	2	0
chr22:40,840,622-41,030,341	189,719	13.1 - q13.	70	4	Loss;Loss;L	P1_S1;P1_S2;P1_S3;P1_S5;P2_S1;P2_S2;P4	Complete;Com	*MKL1, MRTFA, MRT*	3	0
chr22:42,095,684-42,191,728	96,044	q13.2	70	7	Loss;Loss;L	P1_S1;P1_S3;P1_S5;P2_S1;P2_S2;P4_S2;P5_	Complete;Com	*MEI1, bK250D10.C22*	2	0
chr22:42,486,744-42,523,264	36,520	q13.2	70	29	Loss;Loss;L	P1_S1;P1_S3;P1_S5;P2_S1;P2_S2;P4_S2;P5_	Complete;Com	*NDUFA6, NDUFA6-D*	5	0
chr22:42,753,900-42,819,631	65,731	q13.2	70	80	Loss;Loss;L	P1_S1;P1_S3;P1_S5;P2_S1;P2_S2;P4_S2;P5_	Complete;Com	*LINC01315, BC03824*	3	0
chr22:43,194,487-43,474,791	280,304	q13.2	70	2	Loss;Loss;L	P1_S1;P1_S3;P1_S5;P2_S1;P2_S2;P4_S2;P5_	Complete;Com	*ARFGAP3, PACSIN2,*	5	0
chr22:43,543,192-43,559,424	16,232	q13.2	70	100	Loss;Loss;L	P1_S1;P1_S3;P1_S5;P2_S1;P2_S2;P4_S2;P5_	Complete;Com	*TSPO*	1	0
chr22:43,612,150-43,639,085	26,935	q13.2	70	100	Loss;Loss;L	P1_S1;P1_S3;P1_S5;P2_S1;P2_S2;P4_S2;P5_	Complete;Com	*SCUBE1*	1	0
chr22:44,934,263-44,971,134	36,871	q13.31	70	12	Loss;Loss;L	P1_S1;P1_S3;P1_S5;P2_S1;P2_S2;P4_S2;P5_	Complete;Com	*LINC00207*	1	0
chr22:45,086,096-45,110,950	24,854	q13.31	70	100	Loss;Loss;L	P1_S1;P1_S3;P1_S5;P2_S1;P2_S2;P4_S2;P5_	Complete;Com	*PRR5, PRR5-ARHGA*	2	0
chr22:45,157,620-45,192,062	34,442	q13.31	70	100	Loss;Loss;L	P1_S1;P1_S3;P1_S5;P2_S1;P2_S2;P4_S2;P5_	Complete;Com	*PRR5-ARHGAP8, AR*	2	0
chr22:45,292,220-45,309,730	17,510	q13.31	70	100	Loss;Loss;L	P1_S1;P1_S3;P1_S5;P2_S1;P2_S2;P4_S2;P5_	Complete;Com	*PHF21B*	1	0
chr22:45,309,730-45,363,884	54,154	q13.31	70	100	AI;AI;AI;A	P1_S1;P1_S2;P2_S2;P4_S1;P4_S2;P4_S3;P5_	Complete;Com	*PHF21B*	1	0
chr22:45,550,785-45,585,325	34,540	q13.31	70	65	Loss;Loss;L	P1_S1;P1_S3;P1_S5;P2_S1;P2_S2;P4_S2;P5_	Complete;Com	*NUP50-DT, LOC1005*	4	0
chr22:45,821,992-45,864,757	42,765	q13.31	70	100	Loss;Loss;L	P1_S1;P1_S2;P1_S3;P1_S5;P2_S1;P2_S5;P5	Complete;Com	*RIBC2*	1	0
chr22:46,615,500-46,643,971	28,471	q13.31	70	0	Loss;Loss;L	P1_S1;P1_S3;P1_S5;P2_S1;P2_S2;P4_S2;P5_	Complete;Com	*PPARA, CDPF1*	2	0
chr22:46,739,190-46,759,965	20,775	q13.31	70	0	Loss;Loss;L	P1_S1;P1_S3;P1_S5;P2_S1;P2_S2;P4_S2;P5_	Complete;Com	*TRMU, CELSR1*	2	0
chr22:46,931,068-47,002,993	71,925	q13.31	70	8	Loss;Loss;L	P1_S1;P1_S3;P1_S5;P2_S1;P2_S2;P4_S2;P5_	Complete;Com	*CELSR1, GRAMD4*	2	0
chr22:47,308,597-47,456,787	148,190	q13.31	70	84	Loss;Loss;L	P1_S1;P1_S3;P1_S5;P2_S1;P2_S2;P4_S2;P5_	Complete;Com	*FLJ32756, TBC1D22A*	3	0
chr22:47,646,775-47,687,810	41,035	q13.31	70	5	Loss;Loss;L	P1_S1;P1_S3;P1_S5;P2_S1;P2_S2;P4_S2;P5_	Complete;Complete;Complete;Comple		0	0
chr22:47,851,346-48,009,605	158,259	q13.31	70	1	Loss;Loss;L	P1_S1;P1_S3;P1_S5;P2_S1;P2_S2;P4_S2;P5_	Complete;Com	*BC037972, LINC0164*	2	0
chr22:48,257,077-48,308,382	51,305	q13.31	70	0	Loss;Loss;L	P1_S1;P1_S3;P1_S5;P2_S1;P2_S2;P4_S2;P5_	Complete;Complete;Complete;Comple		0	0
chr22:48,695,781-48,748,066	52,285	q13.32	70	7	Loss;Loss;L	P1_S1;P1_S3;P1_S5;P2_S1;P2_S2;P4_S2;P5_	Complete;Complete;Complete;Comple		0	0
chr22:48,766,851-48,810,607	43,756	q13.32	70	82	Loss;Loss;L	P1_S1;P1_S3;P1_S5;P2_S1;P2_S2;P4_S2;P5_	Complete;Complete;Complete;Comple		0	0
chr22:48,925,661-49,054,548	128,887	q13.32	70	99	Loss;Loss;L	P1_S1;P1_S3;P1_S5;P2_S1;P2_S2;P4_S2;P5_	Complete;Com	*LOC284933, FAM19A*	2	0
chr22:49,061,794-49,336,369	274,575	q13.32	70	23	Loss;Loss;L	P1_S1;P1_S3;P1_S5;P2_S1;P2_S2;P4_S2;P5_	Complete;Com	*FAM19A5, MIR4535,*	4	0
chr22:49,353,772-49,397,269	43,497	q13.32	70	100	Loss;Loss;L	P1_S1;P1_S3;P1_S5;P2_S1;P2_S2;P4_S2;P5_	Complete;Complete;Complete;Comple		0	0
chr22:49,457,421-49,505,726	48,305	q13.33	70	100	Loss;Loss;L	P1_S1;P1_S3;P1_S5;P2_S1;P2_S2;P4_S2;P5_	Complete;Complete;Complete;Comple		0	0
chr22:49,541,819-49,571,563	29,744	q13.33	70	100	Loss;Loss;L	P1_S1;P1_S3;P1_S5;P2_S1;P2_S2;P4_S2;P5_	Complete;Complete;Complete;Comple		0	0
chr22:49,767,001-49,825,892	58,891	q13.33	70	100	Loss;Loss;L	P1_S1;P1_S3;P1_S5;P2_S1;P2_S2;P4_S2;P5_	Complete;Com	*C22orf34, BC033837*	2	0
chr22:50,289,819-50,313,458	23,639	q13.33	70	9	Loss;Loss;L	P1_S1;P1_S3;P1_S5;P2_S1;P2_S2;P4_S2;P5_	Complete;Com	*ALG12, CRELD2*	2	0
chr22:50,319,124-50,503,509	184,385	q13.33	70	36	Loss;Loss;L	P1_S1;P1_S3;P1_S5;P2_S1;P2_S2;P4_S2;P5_	Complete;Com	*CRELD2, PIM3, MIR*	6	0
chr22:50,319,124-50,555,728	236,604	q13.33	70	50	AI;AI;AI;A	P1_S1;P1_S2;P2_S2;P4_S1;P4_S2;P4_S3;P5_	Complete;Com	*CRELD2, PIM3, MIR*	7	0

chr22:50,556,374-50,580,990	24,616	q13.33	70	100	Loss;Loss;L	P1_S1;P1_S3;P1_S5;P2_S1;P2_S2;P4_S2;P5_	Complete;Com	*MOV10L1*		1	0
chr22:50,580,990-50,644,720	63,730	q13.33	70	26	AI;AI;AI;A	P1_S1;P1_S2;P2_S2;P4_S1;P4_S2;P4_S3;P5_	Complete;Com	*MOV10L1, PANX2, T*		5	0
chr22:50,656,154-50,658,433	2,279	q13.33	70	0	Loss;Loss;L	P1_S1;P1_S3;P1_S5;P2_S1;P2_S2;P4_S2;P5_	Complete;Com	*TUBGCP6*		1	0
chr22:50,932,558-50,943,097	10,539	q13.33	70	0	Loss;Loss;L	P1_S1;P1_S3;P1_S5;P2_S1;P2_S2;P4_S2;P5_	Complete;Com	*LMF2*		1	0
chr22:50,944,324-50,961,549	17,225	q13.33	70	2	Loss;Loss;L	P1_S1;P1_S3;P1_S5;P2_S1;P2_S2;P4_S2;P5_	Complete;Com	*LMF2, NCAPH2*		2	0
chr22:50,967,622-50,989,710	22,088	q13.33	70	4	Loss;Loss;L	P1_S1;P1_S3;P1_S5;P2_S1;P2_S2;P4_S2;P5_	Complete;Com	*TYMP, ODF3B, KLHI*		4	0
chr22:17,793,490-18,021,648	228,158	11.1 - q11.2	60	23	AI;AI;AI;A	P1_S1;P1_S3;P1_S5;P2_S1;P4_S1;P4_S3;P1_	Complete;Com	*CECR2*		1	0
chr22:18,609,982-19,045,621	435,639	q11.21	60	100	AI;AI;AI;A	P1_S1;P1_S3;P2_S1;P4_S1;P4_S3;P5_S1;P1_	Complete;Com	*TUBA8, USP18, LOC*		23	0
chr22:19,966,005-20,050,913	84,908	q11.21	60	78	AI;AI;AI;A	P1_S1;P1_S3;P1_S5;P4_S1;P4_S3;P5_S1	Complete;Com	*ARVCF, MI*	hsa-mir-1	3	1
chr22:24,220,027-24,553,949	333,922	q11.23	60	100	Loss;Loss;L	P1_S1;P1_S3;P1_S5;P2_S1;P2_S2;P5_S1	Complete;Com	*SLC2A11, DQ601926,*		26	0
chr22:28,129,263-28,154,002	24,739	q12.1	60	6	Loss;Loss;L	P1_S1;P1_S3;P1_S5;P2_S1;P2_S2;P5_S1	Complete;Com	*MN1*		1	0
chr22:31,530,033-31,532,798	2,765	q12.2	60	0	Loss;Loss;L	P1_S1;P1_S3;P1_S5;P2_S1;P2_S2;P5_S1	Complete;Com	*INPP5J, PLA2G3*		2	0
chr22:32,027,963-32,109,833	81,870	q12.2	60	2	Loss;Loss;L	P1_S1;P1_S3;P1_S5;P2_S1;P2_S2;P4_S2	Complete;Com	*PISD, PRR14L*		2	0
chr22:32,140,420-32,205,631	65,211	12.2 - q12.	60	0	Loss;Loss;L	P1_S1;P1_S3;P1_S5;P2_S1;P2_S2;P4_S2	Complete;Com	*PRR14L, DEPDC5*		2	0
chr22:36,682,836-36,698,306	15,470	q12.3	60	100	Loss;Loss;L	P1_S1;P1_S3;P1_S5;P2_S1;P2_S2;P5_S1	Complete;Com	*MIR6819, MYH9*		2	0
chr22:37,500,018-37,530,853	30,835	q12.3	60	0	Loss;Loss;L	P1_S1;P1_S3;P1_S5;P2_S1;P2_S2;P5_S1	Complete;Com	*TMPRSS6, IL2RB*		2	0
chr22:37,827,453-37,871,859	44,406	q13.1	60	0	AI;AI;AI;A	P1_S2;P2_S1;P4_S1;P4_S2;P4_S3;P5_S1	Complete;Com	*MFNG*		1	0
chr22:37,957,241-38,085,192	127,951	q13.1	60	70	AI;AI;AI;A	P1_S2;P2_S1;P4_S1;P4_S2;P4_S3;P5_S1	Complete;Com	*CDC42EP1, LGALS2,*		11	0
chr22:38,121,996-38,168,876	46,880	q13.1	60	100	AI;AI;AI;A	P1_S2;P1_S3;P2_S1;P2_S2;P4_S1;P5_S1	Complete;Com	*TRIOBP*		1	0
chr22:38,221,473-38,240,811	19,338	q13.1	60	100	AI;AI;AI;A	P1_S2;P1_S3;P2_S1;P2_S2;P4_S1;P4_S3	Complete;Com	*GALR3, AN*	hsa-mir-6	3	1
chr22:38,308,397-38,879,126	570,729	q13.1	60	7	AI;AI;AI;A	P1_S2;P1_S3;P2_S1;P2_S2;P4_S1;P4_S3	Complete;Com	*MICALL1, C22orf23,*		22	0
chr22:39,413,725-39,440,933	27,208	q13.1	60	100	AI;AI;AI;A	P1_S2;P2_S1;P2_S2;P4_S1;P4_S2;P4_S3	Complete;Com	*APOBEC3C, APOBE*		4	0
chr22:39,713,688-39,773,618	59,930	q13.1	60	3	Loss;Loss;L	P1_S1;P1_S3;P1_S5;P2_S1;P2_S2;P5_S1	Complete;Com	*RPL3, SNORD43, SY*		4	0
chr22:41,030,341-41,075,630	45,289	q13.2	60	8	AI;AI;AI;A	P1_S2;P1_S3;P2_S1;P2_S2;P4_S1;P4_S3	Complete;Com	*MKL1, MRTFA, MCH*		3	0
chr22:41,158,493-41,270,534	112,041	q13.2	60	14	AI;AI;AI;A	P1_S2;P1_S3;P2_S2;P4_S1;P4_S2;P4_S3;P1_	Complete;Com	*SLC25A17, MIR4766,*		5	0
chr22:41,479,110-41,570,459	91,349	q13.2	60	0	Loss;Loss;L	P1_S1;P1_S3;P1_S5;P2_S1;P2_S2;P4_S2	Complete;Com	*MIR1281, E*	hsa-mir-1	2	1
chr22:42,610,191-42,753,900	143,709	q13.2	60	5	AI;AI;AI;A	P1_S2;P2_S1;P2_S2;P4_S1;P4_S2;P4_S3	Complete;Com	*TCF20, OGFRP1, LO*		3	0
chr22:42,688,681-42,692,758	4,077	q13.2	60	36	Loss;Loss;L	P1_S1;P1_S3;P1_S5;P2_S1;P2_S2;P5_S1	Complete;Complete;Complete;Comple			0	0
chr22:42,961,466-43,004,417	42,951	q13.2	60	100	Loss;Loss;L	P1_S1;P1_S3;P1_S5;P2_S1;P2_S2;P5_S1	Complete;Com	*SERHL2, RRP7B, RR*		4	0
chr22:43,017,713-43,030,177	12,464	q13.2	60	100	Loss;Loss;L	P1_S1;P1_S3;P1_S5;P2_S1;P2_S2;P4_S2	Complete;Com	*DL490307, CYB5R3*		2	0
chr22:44,527,465-44,615,962	88,497	q13.31	60	4	Loss;Loss;L	P1_S1;P1_S3;P1_S5;P2_S1;P2_S2;P5_S1	Complete;Com	*PARVB, TRNA_SeC,*		3	0
chr22:45,237,389-45,258,269	20,880	q13.31	60	67	Loss;Loss;L	P1_S1;P1_S3;P1_S5;P2_S1;P2_S2;P5_S1	Complete;Com	*PRR5-ARHGAP8, AR*		2	0
chr22:45,315,538-45,353,260	37,722	q13.31	60	100	Loss;Loss;L	P1_S1;P1_S3;P1_S5;P2_S1;P2_S2;P5_S1	Complete;Com	*PHF21B*		1	0
chr22:46,835,938-46,915,950	80,012	q13.31	60	3	Loss;Loss;L	P1_S1;P1_S3;P1_S5;P2_S1;P2_S2;P5_S1	Complete;Com	*CELSR1*		1	0
chr22:47,123,459-47,182,113	58,654	q13.31	60	1	Loss;Loss;L	P1_S1;P1_S5;P2_S1;P2_S2;P4_S2;P5_S1	Complete;Com	*CERK, TBC1D22A*		2	0
chr22:49,925,610-50,167,844	242,234	q13.33	60	40	Loss;Loss;L	P1_S1;P1_S3;P1_S5;P2_S1;P2_S2;P5_S1	Complete;Com	*C22orf34, BC033837,*		4	0
chr22:50,682,512-50,682,653	141	q13.33	60	0	AI;AI;AI;A	P1_S1;P1_S2;P2_S1;P4_S1;P4_S3;P5_S1	Complete;Com	*TUBGCP6*		1	0
chr22:50,685,870-50,716,852	30,982	q13.33	60	0	AI;AI;AI;A	P1_S2;P2_S1;P4_S1;P4_S3;P5_S1;P1_S1;P4_	Complete;Com	*HDAC10, MAPK12,*		4	0
chr22:50,749,449-50,825,766	76,317	q13.33	60	20	Loss;Loss;L	P1_S1;P1_S3;P1_S5;P2_S1;P2_S2;P5_S1	Complete;Com	*DENND6B, PPP6R2*		2	0
chr22:50,877,658-50,893,794	16,136	q13.33	60	0	Loss;Loss;L	P1_S1;P1_S3;P1_S5;P2_S1;P2_S2;P5_S1	Complete;Com	*PPP6R2, SBF1*		2	0
chr22:51,038,206-51,119,550	81,344	q13.33	60	45	Loss;Loss;L	P1_S1;P1_S5;P2_S1;P2_S2;P4_S2;P5_S1	Complete;Com	*MAPK8IP2, ARSA, SH*		3	0
chr22:17,222,716-17,289,009	66,293	q11.1	50	100	Loss;Loss;L	P1_S1;P1_S3;P2_S1;P4_S3;P5_S1	Complete;Com	*BC038197, LINC0166*		3	0
chr22:19,789,597-19,808,487	18,890	q11.21	50	83	Loss;Loss;L	P1_S1;P1_S3;P2_S1;P4_S3;P5_S1	Complete;Com	*GNB1L*		1	0
chr22:19,875,935-19,921,120	45,185	q11.21	50	16	Loss;Loss;L	P1_S1;P1_S3;P2_S1;P4_S3;P5_S1	Complete;Com	*TXNRD2*		1	0
chr22:19,943,137-19,951,537	8,400	q11.21	50	13	Loss;Loss;L	P1_S1;P1_S3;P2_S1;P4_S3;P5_S1	Complete;Com	*COMT, MIR4761*		2	0
chr22:39,461,395-39,496,369	34,974	q13.1	50	47	Loss;Loss;L	P1_S1;P1_S5;P2_S1;P2_S2;P5_S1	Complete;Com	*APOBEC3G, APOBE*		2	0
chr22:39,499,832-39,586,009	86,177	q13.1	50	0	AI;AI;AI;A	P1_S2;P2_S2;P4_S1;P4_S2;P4_S3	Complete;Com	*APOBEC3H, CBX7*		2	0

chr22:39,615,218-39,927,188	311,970	q13.1	50	2	AI;AI;AI;A	P1_S2;P2_S2;P4_S1;P4_S3;P2_S1;P4_S2	Complete;Com	*PDGFB, 5S_rRNA, SM*		18	0
chr22:51,038,206-51,064,242	26,036	q13.33	50	0	AI;AI;AI;A	P1_S2;P2_S1;P4_S1;P4_S2;P4_S3	Complete;Com	*MAPK8IP2, ARSA*		2	0
chr10:47,593,880-47,822,143	228,263	q11.22	40	100	AI;AI;AI;A	P1_S1;P1_S2;P1_S3;P1_S5	Complete;Com	*ANTXRLP1, AK05731*		6	0
chr12:38,029,427-38,150,241	120,814	q11	40	100	Gain;Gain;G	P1_S1;P1_S2;P1_S3;P2_S2	Complete;Complete;Complete;Comple			0	0
chr22:17,445,606-17,590,916	145,310	q11.1	40	100	Loss;Loss;L	P1_S1;P1_S3;P2_S1;P5_S1	Complete;Com	*GAB4, CECR7, IL17R*		3	0
chr22:18,264,049-18,305,320	41,271	q11.21	40	100	Loss;Loss;L	P1_S1;P1_S3;P2_S1;P5_S1	Complete;Com	*MICAL3*		1	0
chr22:18,338,118-18,608,846	270,728	q11.21	40	100	Loss;Loss;L	P1_S1;P1_S3;P2_S1;P5_S1	Complete;Com	*MICAL3, M*	hsa-mir-6	7	1
chr22:18,609,982-19,045,621	435,639	q11.21	40	100	AI;AI;AI;A	P1_S1;P1_S3;P2_S1;P4_S1;P4_S3;P5_S1;P1_	Complete;Com	*TUBA8, USP18, LOC*		23	0
chr22:19,127,408-19,600,755	473,347	q11.21	40	41	Loss;Loss;L	P1_S1;P1_S3;P2_S1;P5_S1	Complete;Com	*ESS2, DGCR14, GSC*		16	0
chr22:19,960,601-20,050,913	90,312	q11.21	40	79	Loss;Loss;L	P1_S1;P1_S3;P2_S1;P5_S1	Complete;Com	*ARVCF, MI*	hsa-mir-1	3	1
chr22:20,092,562-21,145,766	1,053,204	q11.21	40	90	Loss;Loss;L	P1_S1;P1_S3;P2_S1;P5_S1	Complete;Com	*DGCR8, TR*	hsa-mir-1	43	1
chr22:21,346,602-21,384,424	37,822	q11.21	40	100	Loss;Loss;L	P1_S1;P1_S3;P2_S1;P5_S1	Complete;Com	*LZTR1, THAP7, DQ5*		7	0
chr22:21,418,755-21,807,793	389,038	q11.21	40	100	Loss;Loss;L	P1_S1;P1_S3;P2_S1;P5_S1	Complete;Com	*BCRP2, POM121L7,*		17	0
chr14:21,353,205-21,421,877	68,672	q11.2	30	100	AI;AI;AI	P4_S1;P4_S2;P4_S3	Complete;Com	*RNASE3, ECRP, RNA*		3	0
chr19:37,898,990-37,906,504	7,514	q13.12	30	0	Gain;Gain;G	P1_S1;P2_S2;P5_S1	Complete;Com	*ZNF569*		1	0
chr7:61,747,794-62,126,898	379,104	q11.21	30	100	Gain;Gain;G	P1_S1;P2_S2;P5_S1	Complete;Complete;Complete			0	0
chr11:50,520,747-50,697,498	176,751	p11.12	20	100	Gain;Gain	P1_S1;P5_S1	Complete;Complete			0	0
chr19:27,862,694-28,061,733	199,039	q11	20	100	Gain;Gain	P1_S1;P2_S2	Complete;Complete			0	0
chr22:17,289,009-17,412,057	123,048	q11.1	20	100	Gain;Gain	P1_S5;P2_S2	Complete;Com	*XKR3, HSFY1P1, IGK*		3	0
chr22:19,221,636-19,419,970	198,334	q11.21	20	8	AI;AI;AI;A	P1_S1;P1_S3;P1_S5;P2_S1;P2_S2;P4_S1;P4	Complete;Com	*CLTCL1, HIRA, MRP*		3	0
chr22:19,645,205-19,729,114	83,909	q11.21	20	100	Gain;Gain	P1_S5;P2_S2	Complete;Com	*SEPT5, SEPT5-GP1B*		3	0
chr3:64,444,942-64,522,288	77,346	p14.1	20	0	AI;AI	P2_S1;P2_S2	Complete;Com	*ADAMTS9*		1	0
chr1:110,736,199-110,756,091	19,892	p13.3	10	0	Loss	P5_S1	Complete	*SLC6A17, LINC02586*		3	0
chr1:112,531,606-112,578,427	46,821	p13.2	10	0	Loss	P5_S1	Complete	*KCND3, LINC01750,*		3	0
chr1:113,425,388-113,450,930	25,542	p13.2	10	100	Gain	P5_S1	Complete			0	0
chr1:113,460,670-113,493,754	33,084	p13.2	10	100	Gain	P5_S1	Complete	*AKR7A2P1, SLC16A1*		2	0
chr1:150,260,171-150,431,301	171,130	21.2 - q21.	10	60	Gain	P5_S1	Complete	*MRPS21, PRPF3, RP*		3	0
chr1:150,604,371-150,884,523	280,152	q21.3	10	3	Gain	P5_S1	Complete	*GOLPH3L, HORMAD*		6	0
chr1:153,973,308-154,125,271	151,963	q21.3	10	3	Gain	P5_S1	Complete	*NUP210L, MIR5698*		2	0
chr1:175,490,384-175,593,871	103,487	q25.1	10	100	Loss	P5_S1	Complete	*TNR, AK093214, BC0*		3	0
chr1:179,095,618-179,268,053	172,435	q25.2	10	2	Gain	P5_S1	Complete	*ABL2, DD413682, SO*		3	0
chr1:201,175,686-201,183,028	7,342	q32.1	10	16	Loss	P5_S1	Complete	*IGFN1*		1	0
chr1:23,855,044-23,885,489	30,445	p36.12	10	0	Gain	P5_S1	Complete	*E2F2, AX748204, ID3*		3	0
chr1:235,360,466-235,600,430	239,964	q42.3	10	3	Gain	P5_S1	Complete	*ARID4B, GGPS1, TBC*		3	0
chr1:28,296,296-28,403,725	107,429	p35.3	10	0	Gain	P5_S1	Complete	*EYA3*		1	0
chr1:28,563,951-28,660,344	96,393	p35.3	10	6	Gain	P5_S1	Complete	*ATP5IF1, ATPIF1, JA*		5	0
chr1:28,677,518-29,301,914	624,396	p35.3	10	5	Gain	P5_S1	Complete	*PHACTR4, SNHG3, S*		19	0
chr1:30,497,110-30,544,270	47,160	p35.2	10	100	Loss	P5_S1	Complete	*BC042538, LINC0164*		2	0
chr1:30,709,695-30,981,393	271,698	p35.2	10	100	Loss	P5_S1	Complete			0	0
chr1:32,974,587-33,096,248	121,661	p35.1	10	6	Gain	P5_S1	Complete	*ZBTB8A, ZBTB8OS*		2	0
chr1:34,350,366-34,394,470	44,104	p35.1	10	0	Loss	P5_S1	Complete	*CSMD2, CSMD2-AS1*		3	0
chr1:35,665,487-35,836,212	170,725	p34.3	10	2	Gain	P5_S1	Complete	*ZMYM4, ZMYM4-AS1*		3	0
chr1:42,031,379-42,047,221	15,842	p34.2	10	0	Loss	P5_S1	Complete	*HIVEP3*		1	0
chr1:48,282,480-48,459,863	177,383	p33	10	0	Loss	P5_S1	Complete	*TRABD2B*		1	0
chr1:52,905,413-53,097,412	191,999	p32.3	10	1	Gain	P5_S1	Complete	*TUT4, ZCCHC11, GP*		3	0
chr1:53,188,909-53,319,562	130,653	p32.3	10	2	Gain	P5_S1	Complete	*ZYG11B, ZYG11A*		2	0
chr1:62,456,903-62,504,343	47,440	p31.3	10	0	Gain	P5_S1	Complete	*PATJ, INADL*		2	0

chr1:9,856,177-10,362,151	505,974	p36.22	10	2	Gain	P5_S1	Complete	*CLSTN1, C*	hsa-mir-1	10	1
chr10:104,854,616-104,936,970	82,354	4.32 - q24.	10	2	Gain	P5_S1	Complete	*NT5C2*		1	0
chr10:11,951,415-12,128,507	177,092	p14	10	35	Gain	P5_S1	Complete	*AK124930, UPF2, DH*		3	0
chr10:119,795,943-119,851,186	55,243	q26.11	10	0	Loss	P5_S1	Complete	*RAB11FIP2, CASC2*		2	0
chr10:12,164,048-12,398,553	234,505	p14 - p13	10	60	Gain	P5_S1	Complete	*DHTKD1, MIR548AK*		6	0
chr10:124,306,511-124,392,089	85,578	q26.13	10	100	Loss	P5_S1	Complete	*DMBT1*		1	0
chr10:130,457,915-130,580,794	122,879	q26.2	10	0	Loss	P5_S1	Complete			0	0
chr10:130,931,384-131,237,029	305,645	q26.3	10	0	Loss	P5_S1	Complete			0	0
chr10:132,490,370-132,534,610	44,240	q26.3	10	100	Loss	P5_S1	Complete			0	0
chr10:134,400,848-134,767,148	366,300	q26.3	10	56	Loss	P5_S1	Complete	*INPP5A, NKX6-2, TT*		5	0
chr10:135,301,554-135,403,443	101,889	q26.3	10	100	Loss	P5_S1	Complete	*CYP2E1, SYCE1, SPR*		3	0
chr10:18,673,662-18,688,735	15,073	p12.32	10	0	Gain	P5_S1	Complete	*CACNB2*		1	0
chr10:35,067,373-35,248,828	181,455	p11.21	10	100	Gain	P5_S1	Complete	*PARD3, PARD3-AS1*		2	0
chr10:44,374,378-44,445,303	70,925	q11.21	10	1	Loss	P5_S1	Complete	*LINC00840, LINC008*		2	0
chr10:49,892,016-50,081,894	189,878	1.22 - q11.	10	3	Loss	P5_S1	Complete	*WDFY4, WDFY4*		2	0
chr10:50,156,341-50,356,678	200,337	q11.23	10	5	Loss	P5_S1	Complete	*WDFY4, M*	hsa-mir-4	5	1
chr10:50,468,777-50,622,877	154,100	q11.23	10	0	Loss	P5_S1	Complete	*C10orf71-AS1, C10or*		3	0
chr10:67,726,868-67,761,351	34,483	q21.3	10	0	Loss	P1_S1	Complete	*CTNNA3*		1	0
chr10:69,725,768-69,924,000	198,232	q21.3	10	10	Gain	P5_S1	Complete	*HERC4, POU5F1P5,*		3	0
chr10:69,970,088-70,593,778	623,690	q21.3	10	7	Gain	P5_S1	Complete	*MYPN, AT*	hsa-mir-1	11	1
chr10:72,600,655-72,732,520	131,865	q22.1	10	20	Loss	P5_S1	Complete	*SGPL1, PCBD1, LOC*		3	0
chr10:73,051,506-73,115,860	64,354	q22.1	10	1	Loss	P5_S1	Complete	*UNC5B, SLC29A3*		2	0
chr10:74,184,444-74,655,781	471,337	q22.1	10	0	Gain	P5_S1	Complete	*MIR1256, MICU1, MI*		5	0
chr10:75,081,598-75,137,327	55,729	q22.2	10	14	Gain	P5_S1	Complete	*TTC18, CFAP70, ANX*		3	0
chr10:80,209,141-80,856,555	647,414	q22.3	10	2	Loss	P5_S1	Complete	*LINC00856, AX74798*		4	0
chr10:87,752,909-87,816,738	63,829	q23.1	10	0	Loss	P5_S1	Complete	*GRID1*		1	0
chr10:93,917,386-94,477,010	559,624	3.32 - q23.	10	5	Gain	P5_S1	Complete	*CPEB3, CPEB3_riboz*		8	0
chr11:11,338,061-11,419,657	81,596	p15.3	10	55	Loss	P5_S1	Complete	*CSNK2A3, GALNT18*		2	0
chr11:11,452,559-11,515,989	63,430	p15.3	10	0	Loss	P5_S1	Complete	*GALNT18*		1	0
chr11:115,915,104-115,964,320	49,216	q23.3	10	0	Loss	P5_S1	Complete			0	0
chr11:126,527,900-126,808,914	281,014	q24.2	10	0	Loss	P5_S1	Complete	*LOC101929427, DJ03*		3	0
chr11:126,877,184-126,891,762	14,578	q24.2	10	0	Loss	P5_S1	Complete			0	0
chr11:131,656,760-131,669,842	13,082	q25	10	100	Loss	P5_S1	Complete	*NTM*		1	0
chr11:133,063,692-133,088,235	24,543	q25	10	100	Loss	P5_S1	Complete	*OPCML*		1	0
chr11:133,367,803-133,431,131	63,328	q25	10	0	Loss	P5_S1	Complete	*OPCML*		1	0
chr11:134,412,787-134,474,259	61,472	q25	10	100	Loss	P5_S1	Complete			0	0
chr11:134,626,528-134,873,998	247,470	q25	10	89	Loss	P5_S1	Complete	*AK125040, LOC7293*		4	0
chr11:19,539,147-19,619,812	80,665	p15.1	10	0	Loss	P5_S1	Complete	*NAV2-AS4, MIR4486,*		4	0
chr11:2,335,352-2,427,906	92,554	p15.5	10	100	Loss	P5_S1	Complete	*TSPAN32, CD81-AS1,*		6	0
chr11:2,443,861-2,842,050	398,189	15.5 - p15.	10	43	Loss	P5_S1	Complete	*TRPM5, KCNQ1, KC*		3	0
chr11:45,249,023-45,531,664	282,641	p11.2	10	2	Loss	P5_S1	Complete	*PRDM11, SYT13, TR*		6	0
chr11:47,610,876-47,689,228	78,352	p11.2	10	37	Gain	P5_S1	Complete	*C1QTNF4, MTCH2, A*		3	0
chr11:47,812,262-48,062,180	249,918	p11.2	10	3	Gain	P5_S1	Complete	*NUP160, Y_RNA, PT*		3	0
chr11:58,875,056-58,930,065	55,009	q12.1	10	100	Loss	P5_S1	Complete	*FAM111B, FAM111A-*		4	0
chr11:78,512,928-78,625,515	112,587	q14.1	10	0	Loss	P5_S1	Complete	*TENM4, TENM4*		2	0
chr11:78,724,347-78,771,833	47,486	q14.1	10	0	Loss	P5_S1	Complete	*TENM4, TENM4*		2	0
chr11:79,125,002-79,187,735	62,733	q14.1	10	0	Loss	P5_S1	Complete	*TENM4, MIR5579*		2	0

chr11:9,304,585-9,682,443	377,858	p15.4	10	21	Gain	P5_S1	Complete	*TMEM41B, IPO7, SN*		9	0
chr12:110,559,495-110,807,789	248,294	q24.11	10	0	Gain	P5_S1	Complete	*IFT81, JA611269, AT*		3	0
chr12:110,940,880-111,046,496	105,616	q24.11	10	2	Gain	P5_S1	Complete	*BC064974, RAD9B, P*		3	0
chr12:111,141,120-111,176,353	35,233	q24.11	10	28	Gain	P5_S1	Complete	*PPP1CC*		1	0
chr12:111,933,303-111,954,884	21,581	q24.12	10	0	Gain	P5_S1	Complete	*ATXN2*		1	0
chr12:112,238,561-112,301,305	62,744	4.12 - q24.	10	100	Gain	P5_S1	Complete	*ALDH2, MAPKAPK5-*		3	0
chr12:118,535,619-118,851,609	315,990	q24.23	10	2	Gain	P5_S1	Complete	*VSIG10, PEBP1, TAO*		4	0
chr12:122,303,297-122,395,023	91,726	q24.31	10	2	Gain	P5_S1	Complete	*HPD, PSMD9, WDR6*		3	0
chr12:122,631,058-122,653,940	22,882	q24.31	10	71	Gain	P5_S1	Complete	*MLXIP, LRRC43*		2	0
chr12:122,959,294-123,118,754	159,460	q24.31	10	0	Gain	P5_S1	Complete	*ZCCHC8, SNORA9, R*		5	0
chr12:130,803,001-130,880,190	77,189	q24.33	10	100	Loss	P5_S1	Complete	*PIWIL1*		1	0
chr12:131,630,343-131,704,894	74,551	q24.33	10	100	Loss	P5_S1	Complete	*LINC01257, LOC116*		2	0
chr12:131,746,199-131,964,016	217,817	q24.33	10	100	Loss	P5_S1	Complete	*BC042649, LINC024*		4	0
chr12:133,779,171-133,851,895	72,724	q24.33	10	1	Gain	P2_S2	Complete	*ZNF268, ANHX*		2	0
chr12:2,250,067-2,598,551	348,484	p13.33	10	20	Loss	P5_S1	Complete	*CACNA1C-AS4, CAC*		3	0
chr12:31,608,536-32,126,315	517,779	p11.21	10	38	Gain	P5_S1	Complete	*DENND5B, U5, DEN*		10	0
chr12:32,144,079-32,370,401	226,322	p11.21	10	42	Gain	P5_S1	Complete	*KIAA1551, RESF1, R*		4	0
chr12:32,534,123-32,778,351	244,228	p11.21	10	69	Gain	P5_S1	Complete	*BICD1, FGD4*		2	0
chr12:34,554,847-34,756,688	201,841	p11.1	10	100	Gain	P2_S2	Complete			0	0
chr12:50,748,220-50,868,515	120,295	q13.12	10	0	Gain	P5_S1	Complete	*FAM186A, LARP4, S*		3	0
chr12:51,189,659-51,345,919	156,260	q13.12	10	1	Gain	P5_S1	Complete	*ATF1, TMPRSS12, M*		3	0
chr12:51,470,613-51,617,980	147,367	3.12 - q13.	10	4	Gain	P5_S1	Complete	*CSRNP2, LOC494150*		4	0
chr12:52,706,916-53,077,045	370,129	q13.13	10	41	Loss	P5_S1	Complete	*KRT83, KRT85, KRT8*		16	0
chr12:53,093,307-53,098,697	5,390	q13.13	10	100	Loss	P5_S1	Complete	*KRT77*		1	0
chr12:53,850,264-53,873,083	22,819	q13.13	10	5	Gain	P5_S1	Complete	*PCBP2, PCBP2-OT1*		2	0
chr12:56,874,648-56,944,886	70,238	q13.3	10	12	Gain	P5_S1	Complete	*GLS2, BC059370, SN*		4	0
chr12:7,871,634-7,960,432	88,798	p13.31	10	100	Gain	P5_S1	Complete	*CLEC4C, NANOGNB,*		3	0
chr12:95,401,549-95,453,891	52,342	q22	10	6	Gain	P5_S1	Complete	*NR2C1*		1	0
chr12:95,694,298-95,781,377	87,079	q22	10	0	Gain	P5_S1	Complete	*VEZT, MIR*	hsa-mir-3	3	1
chr12:95,945,553-96,001,537	55,984	q22	10	0	Gain	P5_S1	Complete			0	0
chr13:86,735,102-86,946,800	211,698	q31.1	10	3	Gain	P5_S1	Complete			0	0
chr14:101,507,650-101,660,126	152,476	q32.31	10	17	Loss	P5_S1	Complete	*MIR300, M*	hsa-mir-3	29	24
chr14:31,556,385-31,590,643	34,258	q12	10	10	Gain	P5_S1	Complete	*AP4S1, HECTD1*		2	0
chr14:31,627,365-31,768,403	141,038	q12	10	1	Gain	P5_S1	Complete	*HECTD1, HEATR5A*		2	0
chr14:34,882,747-35,716,707	833,960	13.1 - q13.	10	40	Gain	P5_S1	Complete	*SPTSSA, EAPP, SNX6*		13	0
chr14:50,100,462-50,266,644	166,182	q21.3	10	100	Gain	P5_S1	Complete	*DNAAF2, POLE2, NE*		5	0
chr14:64,066,261-64,407,219	340,958	q23.2	10	82	Gain	P5_S1	Complete	*WDR89, JA429503, S*		4	0
chr14:73,611,006-73,683,789	72,783	q24.2	10	0	Gain	P5_S1	Complete	*PSEN1, BC016143*		2	0
chr14:74,258,255-74,296,015	37,760	q24.3	10	0	Gain	P5_S1	Complete	*LOC100506476, BC0*		3	0
chr14:74,454,095-74,495,178	41,083	q24.3	10	100	Gain	P5_S1	Complete	*ENTPD5, BBOF1, CC*		3	0
chr15:20,071,673-20,771,825	700,152	11.1 - q11.	10	100	Gain	P5_S1	Complete	*DQ576041, DQ57147*		14	0
chr15:41,283,831-41,749,261	465,430	q15.1	10	3	Gain	P5_S1	Complete	*INO80, EXD1, CHP1,*		9	0
chr15:44,396,419-44,822,017	425,598	15.3 - q21.	10	1	Gain	P5_S1	Complete	*FRMD5, CASC4, CTL*		3	0
chr15:50,700,474-51,143,697	443,223	q21.2	10	2	Gain	P5_S1	Complete	*USP8, AX746640, US*		7	0
chr15:64,708,593-64,948,306	239,713	q22.31	10	6	Gain	P5_S1	Complete	*TRIP4, ZNF609*		2	0
chr15:70,127,025-70,238,468	111,443	q23	10	0	Loss	P5_S1	Complete	*LINC00593*		1	0
chr15:70,345,623-70,486,961	141,338	q23	10	29	Loss	P5_S1	Complete	*TLE3, MIR*	hsa-mir-6	3	1

chr16:11,862,720-11,931,009	68,289	p13.13	10	3	Gain	P5_S1	Complete	*ZC3H7A, BCAR4, RSI*		3	0
chr16:11,952,968-11,989,997	37,029	p13.13	10	0	Gain	P5_S1	Complete	*GSPT1*		1	0
chr16:68,946,640-69,151,586	204,946	q22.1	10	1	Gain	P5_S1	Complete	*TANGO6, HAS3*		2	0
chr16:69,286,861-69,345,759	58,898	q22.1	10	0	Gain	P5_S1	Complete	*SNTB2, VPS4A*		2	0
chr16:69,497,097-69,743,069	245,972	q22.1	10	1	Gain	P5_S1	Complete	*CYB5B, MI*	hsa-mir-1	4	1
chr16:74,514,554-74,684,513	169,959	q23.1	10	52	Gain	P5_S1	Complete	*GLG1, RFWD3*		2	0
chr17:1,022,288-1,161,130	138,842	p13.3	10	22	Gain	P5_S1	Complete	*ABR*		1	0
chr17:1,221,813-1,300,039	78,226	p13.3	10	7	Gain	P5_S1	Complete	*YWHAE*		1	0
chr17:1,503,214-1,538,680	35,466	p13.3	10	34	Gain	P5_S1	Complete	*SLC43A2, SCARF1*		2	0
chr17:1,633,844-1,637,634	3,790	p13.3	10	100	Gain	P5_S1	Complete	*WDR81*		1	0
chr17:2,461,706-2,561,661	99,955	p13.3	10	28	Gain	P5_S1	Complete	*DD413682, PAFAH1E*		2	0
chr17:21,833,289-22,034,501	201,212	p11.2	10	100	Gain	P5_S1	Complete	*FLJ36000, TRNA, JB1*		5	0
chr17:27,713,999-27,795,204	81,205	q11.2	10	4	Gain	P5_S1	Complete	*MIR4523, TAOK1*		2	0
chr17:29,108,685-29,232,273	123,588	q11.2	10	100	Gain	P5_S1	Complete	*SUZ12P1, CRLF3, AT*		4	0
chr17:30,482,658-30,581,133	98,475	q11.2	10	0	Gain	P5_S1	Complete	*RHOT1*		1	0
chr17:41,218,772-41,244,340	25,568	q21.31	10	2	Gain	P5_S1	Complete	*BRCA1*		1	0
chr17:41,245,666-41,342,263	96,597	q21.31	10	5	Gain	P5_S1	Complete	*BRCA1, NBR2, AK093*		6	0
chr17:56,800,662-57,283,762	483,100	q22	10	6	Gain	P5_S1	Complete	*RAD51C, P*	hsa-mir-4	8	2
chr17:57,293,276-57,938,064	644,788	q22 - q23.1	10	4	Gain	P5_S1	Complete	*GDPD1, M*	hsa-mir-2	11	1
chr17:58,764,043-58,977,259	213,216	q23.2	10	13	Gain	P5_S1	Complete	*BCAS3*		1	0
chr17:60,064,999-60,484,646	419,647	q23.2	10	43	Gain	P5_S1	Complete	*MED13, Mir_652, TB*		4	0
chr17:62,388,750-62,489,030	100,280	q23.3	10	2	Gain	P5_S1	Complete	*PECAM1, MILR1, PO*		3	0
chr17:62,537,255-62,744,994	207,739	23.3 - q24.	10	21	Gain	P5_S1	Complete	*SMURF2*		1	0
chr17:65,313,026-65,510,858	197,832	q24.2	10	66	Gain	P5_S1	Complete	*PSMD12, M*	hsa-mir-5	4	1
chr17:65,577,004-65,955,751	378,747	q24.2	10	33	Gain	P5_S1	Complete	*PITPNC1, NOL11, SN*		4	0
chr18:18,570,235-19,023,053	452,818	11.1 - q11.	10	1	Gain	P5_S1	Complete	*ROCK1, GREB1L*		2	0
chr18:19,110,947-19,487,345	376,398	q11.2	10	0	Gain	P5_S1	Complete	*ESCO1, SN*	hsa-mir-3	9	3
chr18:35,040,189-35,079,040	38,851	q12.2	10	0	Loss	P5_S1	Complete	*CELF4, SNORA111*		2	0
chr18:35,211,386-35,263,506	52,120	q12.2	10	0	Loss	P5_S1	Complete	*MIR4318*	hsa-mir-4	1	1
chr18:72,821,512-72,866,568	45,056	q22.3	10	100	Loss	P5_S1	Complete			0	0
chr18:73,022,307-73,049,243	26,936	q22.3	10	100	Loss	P5_S1	Complete			0	0
chr18:73,137,090-73,273,404	136,314	q23	10	100	Loss	P5_S1	Complete	*SMIM21*		1	0
chr19:10,420,979-10,444,027	23,048	p13.2	10	0	Gain	P5_S1	Complete	*FDX1L, FDX2, BC018*		4	0
chr19:10,601,111-10,644,656	43,545	p13.2	10	0	Gain	P5_S1	Complete	*KEAP1, S1PR5*		2	0
chr19:11,298,308-11,304,491	6,183	p13.2	10	100	Gain	P5_S1	Complete	*KANK2*		1	0
chr19:11,598,129-11,618,728	20,599	p13.2	10	0	Gain	P5_S1	Complete	*ZNF653, MIR7974, E*		3	0
chr19:11,729,871-11,889,513	159,642	p13.2	10	11	Gain	P5_S1	Complete	*ZNF627, ZNF833P, Z*		4	0
chr19:12,270,177-12,428,600	158,423	p13.2	10	35	Gain	P5_S1	Complete	*ZNF136, AX721123, L*		6	0
chr19:12,542,387-12,691,506	149,119	p13.2	10	100	Gain	P5_S1	Complete	*ZNF443, ZNF709, ZN*		4	0
chr19:13,679,542-13,879,427	199,885	p13.2	10	3	Gain	P5_S1	Complete	*CCDC130, MRI1*		2	0
chr19:14,542,081-14,573,953	31,872	p13.12	10	0	Gain	P5_S1	Complete	*PKN1*		1	0
chr19:17,361,212-17,370,439	9,227	p13.11	10	100	Gain	P5_S1	Complete	*USHBP1*		1	0
chr19:17,615,511-17,624,026	8,515	p13.11	10	0	Gain	P5_S1	Complete	*SLC27A1, LOC10050*		3	0
chr19:21,124,930-21,141,796	16,866	p12	10	18	Gain	P2_S2	Complete	*ZNF85*		1	0
chr19:21,224,667-21,270,879	46,212	p12	10	100	Gain	P5_S1	Complete	*ZNF430, ZNF714*		2	0
chr19:21,719,197-21,730,430	11,233	p12	10	100	Gain	P1_S1	Complete	*ZNF429*		1	0
chr19:24,378,791-24,518,609	139,818	p12 - p11	10	100	Gain	P1_S1	Complete			0	0

chr19:36,643,058-37,449,414	806,356	q13.12	10	27	Gain	P5_S1	Complete	*COX7A1, ZNF565, ZN*		28	0
chr19:37,591,022-37,738,433	147,411	q13.12	10	15	Gain	P5_S1	Complete	*ZNF420, ZNF585A, Z*		4	0
chr19:39,234,744-39,287,664	52,920	q13.2	10	13	Gain	P5_S1	Complete	*CAPN12, LGALS7, LC*		3	0
chr19:4,502,152-4,510,705	8,553	p13.3	10	8	Gain	P5_S1	Complete	*HDGFL2, HDGFRP2,*		3	0
chr19:4,594,523-4,690,297	95,774	p13.3	10	99	Gain	P5_S1	Complete	*TNFAIP8L1, C19orf1*		6	0
chr19:40,520,854-40,541,629	20,775	q13.2	10	1	Gain	P2_S2	Complete	*ZNF546, ZNF780B*		2	0
chr19:44,350,723-44,352,673	1,950	q13.31	10	0	Gain	P2_S2	Complete	*ZNF283*		1	0
chr19:46,029,706-46,078,250	48,544	q13.32	10	4	Gain	P5_S1	Complete	*VASP, OPA3*		2	0
chr19:48,285,243-48,502,016	216,773	q13.33	10	97	Gain	P5_S1	Complete	*SELENOW, SEPW1, A*		20	0
chr19:49,116,167-49,192,874	76,707	q13.33	10	100	Gain	P5_S1	Complete	*FAM83E, RPL18, SPH*		7	0
chr19:49,254,326-49,308,887	54,561	q13.33	10	100	Gain	P5_S1	Complete	*FUT1, FGF21, BCAT2*		3	0
chr19:52,537,268-52,538,142	874	q13.41	10	100	Gain	P1_S3	Complete	*ZNF432*		1	0
chr19:53,056,475-53,066,259	9,784	q13.41	10	100	Gain	P1_S1	Complete	*ZNF808*		1	0
chr19:54,193,671-54,292,736	99,065	q13.42	10	100	Gain	P5_S1	Complete	*MIR520A, M*	hsa-mir-52	39	38
chr19:6,942,952-7,125,438	182,486	p13.2	10	100	Gain	P5_S1	Complete	*ADGRE4P, EMR4P, F*		10	0
chr19:7,189,177-7,497,290	308,113	p13.2	10	100	Gain	P5_S1	Complete	*INSR, ARHGEF18*		2	0
chr19:7,708,136-7,734,029	25,893	p13.2	10	100	Gain	P5_S1	Complete	*STXBP2, RETN*		2	0
chr19:746,333-757,252	10,919	p13.3	10	100	Gain	P5_S1	Complete	*PALM, MISP*		2	0
chr19:8,201,078-8,375,392	174,314	p13.2	10	35	Gain	P5_S1	Complete	*FBN3, CERS4, CD32*		4	0
chr19:8,634,354-8,769,167	134,813	p13.2	10	8	Gain	P5_S1	Complete	*MYO1F, ADAMTS10*		2	0
chr19:8,924,646-8,953,859	29,213	p13.2	10	100	Gain	P5_S1	Complete	*ZNF558, MBD3L1*		2	0
chr19:868,758-890,414	21,656	p13.3	10	100	Gain	P5_S1	Complete	*MED16, MED16*		2	0
chr19:9,098,988-9,202,005	103,017	p13.2	10	0	Gain	P5_S1	Complete	*TRNA_Pseudo*		1	0
chr19:9,868,324-9,869,295	971	p13.2	10	100	Gain	P1_S1	Complete	*ZNF846*		1	0
chr2:0-470,504	470,504	p25.3	10	88	Loss	P5_S1	Complete	*FAM110C, SH3YL1, A*		6	0
chr2:1,204,824-1,276,701	71,877	p25.3	10	16	Loss	P5_S1	Complete	*SNTG2*		1	0
chr2:1,423,172-1,465,311	42,139	p25.3	10	100	Loss	P5_S1	Complete	*TPO*		1	0
chr2:102,806,372-102,836,648	30,276	q12.1	10	2	Loss	P5_S1	Complete	*IL1RL2*		1	0
chr2:119,364,013-119,419,375	55,362	q14.2	10	0	Loss	P5_S1	Complete			0	0
chr2:163,185,162-163,233,231	48,069	q24.2	10	3	Loss	P5_S1	Complete	*GCA, KCNH7*		2	0
chr2:17,679,264-17,697,234	17,970	p24.2	10	0	Loss	P5_S1	Complete	*RAD51AP2*		1	0
chr2:2,610,653-2,752,111	141,458	p25.3	10	5	Loss	P5_S1	Complete			0	0
chr2:203,040,470-203,420,006	379,536	33.1 - q33.	10	17	Gain	P5_S1	Complete	*LOC100652824, KIAA*		10	0
chr2:203,424,957-204,302,729	877,772	q33.2	10	7	Gain	P5_S1	Complete	*BMPR2, FAM117B, IC*		10	0
chr2:21,230,714-21,233,662	2,948	p24.1	10	0	Loss	P5_S1	Complete	*APOB*		1	0
chr2:218,623,066-218,689,541	66,475	q35	10	14	Loss	P5_S1	Complete	*TNS1*		1	0
chr2:238,239,548-238,307,688	68,140	q37.3	10	0	Loss	P5_S1	Complete	*COL6A3*		1	0
chr2:240,113,554-240,419,667	306,113	q37.3	10	13	Loss	P5_S1	Complete	*MGC16025*	hsa-mir-4	5	1
chr2:240,922,142-240,979,466	57,324	q37.3	10	1	Loss	P5_S1	Complete	*NDUFA10, OR6B2*		2	0
chr2:241,064,765-241,239,644	174,879	q37.3	10	7	Loss	P5_S1	Complete	*MYEOV2, COPS9, OT*		3	0
chr2:241,341,946-241,536,120	194,174	q37.3	10	25	Loss	P5_S1	Complete	*GPC1, PPI*	hsa-mir-1	9	1
chr2:241,566,650-241,577,867	11,217	q37.3	10	0	Loss	P5_S1	Complete	*GPR35*		1	0
chr2:242,929,198-243,199,373	270,175	q37.3	10	64	Loss	P5_S1	Complete	*LINC01237, LOC285*		6	0
chr2:32,292,566-32,719,210	426,644	p22.3	10	44	Gain	P5_S1	Complete	*SPAST, SLC30A6, NL*		5	0
chr2:44,147,455-44,186,364	38,909	p21	10	0	Gain	P5_S1	Complete	*LRPPRC*		1	0
chr2:55,490,816-55,711,259	220,443	p16.1	10	13	Gain	P5_S1	Complete	*MTIF2, PRORSD1P, C*		3	0
chr2:61,234,572-61,324,874	90,302	p16.1 - p15	10	4	Gain	P5_S1	Complete	*PUS10, PEX13, KIAA*		3	0

chr2:61,448,716-62,066,996	618,280	p15	10	3	Gain	P5_S1	Complete	*USP34, USP34, SNOR*		5	0
chr2:613,975-706,018	92,043	p25.3	10	100	Loss	P5_S1	Complete	*TMEM18*		1	0
chr2:62,069,341-62,132,310	62,969	p15	10	3	Gain	P5_S1	Complete	*FAM161A, CCT4, CO*		3	0
chr2:7,024,894-7,048,125	23,231	p25.2	10	0	Loss	P5_S1	Complete	*RSAD2*		1	0
chr2:71,827,856-71,912,130	84,274	p13.2	10	14	Loss	P5_S1	Complete	*DYSF*		1	0
chr2:72,102,149-72,148,034	45,885	p13.2	10	0	Loss	P5_S1	Complete			0	0
chr2:91,776,211-92,275,027	498,816	p11.1	10	100	Gain	P5_S1	Complete	*LOC654342, LSP1P4,*		6	0
chr20:1,456,967-1,469,429	12,462	p13	10	0	Loss	P5_S1	Complete	*SIRPB2*		1	0
chr20:19,361,675-19,419,518	57,843	p11.23	10	0	Loss	P5_S1	Complete	*SLC24A3*		1	0
chr20:22,941,973-23,013,280	71,307	p11.21	10	10	Loss	P5_S1	Complete	*BC045663*		1	0
chr20:23,026,383-23,095,191	68,808	p11.21	10	96	Loss	P5_S1	Complete	*AX747264, THBD, CL*		3	0
chr20:23,377,766-23,549,451	171,685	p11.21	10	0	Loss	P5_S1	Complete	*NAPB, CSTL1, CST11*		6	0
chr20:24,551,970-24,965,767	413,797	p11.21	10	17	Loss	P5_S1	Complete	*SYNDIG1, CST7, APM*		3	0
chr20:26,086,933-26,198,853	111,920	p11.1	10	100	Gain	P5_S1	Complete	*NCOR1P1,*	hsa-mir-6	4	1
chr20:34,288,695-34,484,941	196,246	1.22 - q11.	10	3	Gain	P5_S1	Complete	*ROMO1, RBM39, PHF*		3	0
chr20:35,443,730-35,507,557	63,827	q11.23	10	1	Gain	P5_S1	Complete	*SOGA1, TLDC2*		2	0
chr20:35,661,247-35,680,318	19,071	q11.23	10	23	Gain	P5_S1	Complete	*RBL1*		1	0
chr20:50,419,580-50,679,202	259,622	q13.2	10	5	Gain	P5_S1	Complete	*LINC01429*		1	0
chr20:56,137,880-56,211,556	73,676	q13.31	10	55	Loss	P5_S1	Complete	*PCK1, ZBP1*		2	0
chr20:56,274,892-56,360,068	85,176	q13.31	10	100	Loss	P5_S1	Complete	*PMEPA1, AK056098,*		3	0
chr20:56,466,703-56,606,936	140,233	3.31 - q13.	10	100	Loss	P5_S1	Complete	*MIR4532, LINC01742*		2	0
chr20:58,407,900-58,648,173	240,273	q13.33	10	41	Loss	P5_S1	Complete	*PHACTR3, SYCP2, PI*		6	0
chr20:58,452,574-58,464,462	11,888	q13.33	10	100	Gain	P2_S1	Complete	*SYCP2*		1	0
chr20:59,753,948-60,221,920	467,972	q13.33	10	26	Loss	P5_S1	Complete	*CDH4*		1	0
chr20:60,316,417-60,418,246	101,829	q13.33	10	2	Loss	P5_S1	Complete	*CDH4*		1	0
chr21:46,032,288-46,078,059	45,771	q22.3	10	100	Loss	P5_S1	Complete	*TSPEAR, KRTAP10-8,*		7	0
chr22:17,588,296-17,681,342	93,046	q11.1	10	33	Gain	P1_S5	Complete	*IL17RA, CECR6, TMI*		11	0
chr22:17,793,490-18,021,648	228,158	11.1 - q11.2	10	23	AI;AI;AI;A	P1_S1;P1_S3;P1_S5;P2_S1;P4_S1;P4_S3;P1_	Complete;Com	*CECR2*		1	0
chr22:18,031,637-18,272,169	240,532	q11.21	10	100	Gain	P1_S5	Complete	*CECR2, SL*	hsa-mir-3	10	1
chr22:18,368,347-18,559,540	191,193	q11.21	10	100	Gain	P1_S5	Complete	*MICAL3, M*	hsa-mir-6	4	1
chr22:18,577,540-19,089,683	512,143	q11.21	10	95	Gain	P1_S5	Complete	*TUBA8, USP18, LOC*		23	0
chr22:19,966,005-21,026,935	1,060,930	q11.21	10	89	Gain	P1_S5	Complete	*ARVCF, MI*	hsa-mir-1	41	3
chr22:21,166,489-21,328,962	162,473	q11.21	10	100	Gain	P1_S5	Complete	*PI4KA, SNAP29, CRK*		7	0
chr22:21,424,931-21,461,685	36,754	q11.21	10	100	Gain	P1_S5	Complete	*BCRP2*		1	0
chr22:21,864,590-51,304,566	29,439,976	1.21 - q13.	10	34	LOH	P1_S5	Complete	*PI4KAP2, R*	hsa-mir-3	690	15
chr3:10,589,880-10,968,701	378,821	p25.3	10	100	Loss	P5_S1	Complete	*ATP2B2, ATP2B2-IT2*		4	0
chr3:126,499,723-126,708,027	208,304	q21.3	10	52	Loss	P5_S1	Complete	*CHCHD6, PLXNA1*		2	0
chr3:126,735,272-127,129,291	394,019	q21.3	10	10	Loss	P5_S1	Complete	*PLXNA1, C3orf56, PR*		5	0
chr3:136,083,073-136,435,094	352,021	q22.3	10	2	Gain	P5_S1	Complete	*STAG1*		1	0
chr3:139,848,798-140,178,603	329,805	q23	10	75	Loss	P5_S1	Complete	*CLSTN2*		1	0
chr3:141,682,923-141,869,273	186,350	q23	10	35	Gain	P5_S1	Complete	*TFDP2*		1	0
chr3:169,852,066-169,962,326	110,260	q26.2	10	9	Gain	P5_S1	Complete	*PHC3, AY940074, BC*		4	0
chr3:196,471,506-196,609,779	138,273	q29	10	26	Gain	P5_S1	Complete	*PAK2, SENP5*		2	0
chr3:47,635,813-47,852,070	216,257	p21.31	10	100	Gain	P5_S1	Complete	*SNORD146, SMARCC*		3	0
chr3:47,957,692-48,162,174	204,482	p21.31	10	2	Gain	P5_S1	Complete	*MAP4*		1	0
chr3:49,212,692-49,287,915	75,223	p21.31	10	0	Gain	P5_S1	Complete	*KLHDC8B, C3orf84, I*		4	0
chr3:57,443,720-57,489,708	45,988	p14.3	10	6	Gain	P5_S1	Complete	*DNAH12*		1	0

chr3:57,822,686-57,898,652	75,966	p14.3	10	2	Gain	P5_S1	Complete	*SLMAP*		1	0
chr3:8,666,243-8,684,306	18,063	p26.1	10	0	Loss	P5_S1	Complete	*SSUH2*		1	0
chr3:90,229,593-91,000,000	770,407	11.1 - q11.	10	36	Gain	P5_S1	Complete			0	0
chr3:93,550,578-93,708,692	158,114	q11.1	10	44	Gain	P5_S1	Complete	*PROS1, ARL13B*		2	0
chr4:140,005,987-140,181,870	175,883	q31.1	10	6	Gain	P5_S1	Complete	*ELF2*		1	0
chr4:165,689,413-165,818,717	129,304	q32.3	10	0	Gain	P5_S1	Complete	*SMIM31, LOC100505*		4	0
chr4:189,209,272-189,229,400	20,128	q35.2	10	100	Gain	P5_S1	Complete			0	0
chr4:39,412,029-40,104,018	691,989	p14	10	6	Gain	P5_S1	Complete	*MIR5591, KLB, RPL9,*		13	0
chr4:40,122,482-40,160,717	38,235	p14	10	0	Gain	P5_S1	Complete	*N4BP2*		1	0
chr4:40,446,890-40,477,827	30,937	p14	10	0	Gain	P5_S1	Complete	*RBM47*		1	0
chr4:40,577,815-40,615,792	37,977	p14	10	0	Gain	P5_S1	Complete	*RBM47*		1	0
chr4:40,645,843-40,778,178	132,335	p14	10	76	Gain	P5_S1	Complete	*NSUN7*		1	0
chr4:52,706,270-52,844,535	138,265	q12	10	95	Gain	P5_S1	Complete	*DCUN1D4*		1	0
chr4:7,373,892-7,477,027	103,135	p16.1	10	7	Loss	P5_S1	Complete	*PSAPL1, M*	hsa-mir-4	3	1
chr4:7,522,756-7,707,444	184,688	p16.1	10	4	Loss	P5_S1	Complete	*SORCS2, DQ572409*		2	0
chr4:9,994,698-10,273,286	278,588	p16.1	10	100	Loss	P5_S1	Complete	*SLC2A9, M*	hsa-mir-3	12	1
chr5:0-353,968	353,968	p15.33	10	97	Loss	P5_S1	Complete	*PLEKHG4B, LRRC14*		7	0
chr5:134,576,679-134,870,152	293,473	q31.1	10	1	Loss	P5_S1	Complete	*C5orf66, LOC100996*		10	0
chr5:138,762,651-138,857,466	94,815	q31.2	10	6	Gain	P5_S1	Complete	*DNAJC18, ECSCR, SM*		4	0
chr5:17,386,349-17,707,319	320,970	p15.1	10	100	Gain	P5_S1	Complete	*LINC02111, LOC401*		5	0
chr5:2,466,690-2,507,608	40,918	p15.33	10	4	Loss	P5_S1	Complete			0	0
chr5:32,291,261-32,370,408	79,147	p13.3	10	21	Gain	P5_S1	Complete	*MTMR12, ZFR*		2	0
chr5:37,170,227-37,304,319	134,092	p13.2	10	7	Gain	P5_S1	Complete	*C5orf42, CPLANE1, L*		4	0
chr5:5,439,347-5,491,134	51,787	p15.32	10	0	Loss	P5_S1	Complete	*KIAA0947, ICE1*		2	0
chr5:68,491,633-68,709,767	218,134	q13.2	10	65	Gain	P5_S1	Complete	*CENPH, MRPS36, CD*		7	0
chr5:708,009-947,267	239,258	p15.33	10	100	Loss	P5_S1	Complete	*ZDHHC11B, ZDHHC*		4	0
chr6:107,378,766-107,594,064	215,298	q21	10	4	Gain	P5_S1	Complete	*BEND3, PDSS2*		2	0
chr6:110,983,560-111,345,084	361,524	q21	10	8	Gain	P5_S1	Complete	*CDK19, BC047513, S*		6	0
chr6:169,600,551-169,646,640	46,089	q27	10	10	Loss	P5_S1	Complete	*LOC101929523, THB*		2	0
chr6:170,089,068-170,176,689	87,621	q27	10	3	Loss	P5_S1	Complete	*WDR27, C6orf120, PH*		7	0
chr6:21,622,782-21,699,508	76,726	p22.3	10	47	Gain	P5_S1	Complete	*CASC15, LINC00340*		2	0
chr6:40,034,126-40,380,789	346,663	p21.2	10	15	Loss	P5_S1	Complete	*LINC00951, DQ5719*		4	0
chr6:40,617,101-41,040,802	423,701	p21.1	10	1	Loss	P5_S1	Complete	*Mir_652, LOC101929*		7	0
chr6:58,603,762-58,752,089	148,327	11.2 - p11.	10	100	Gain	P5_S1	Complete	*Mir_598*		1	0
chr6:74,123,461-74,169,133	45,672	q13	10	4	Gain	P5_S1	Complete	*DDX43, MB21D1, CG*		3	0
chr6:74,192,220-74,382,069	189,849	q13	10	2	Gain	P5_S1	Complete	*MTO1, SNORD141A,*		7	0
chr7:0-162,665	162,665	p22.3	10	94	Loss	P5_S1	Complete	*LOC102723672, AK0*		4	0
chr7:104,932,058-105,125,422	193,364	q22.3	10	100	Gain	P5_S1	Complete	*SRPK2, PUS7*		2	0
chr7:129,700,494-129,786,610	86,116	q32.2	10	0	Gain	P5_S1	Complete	*KLHDC10, LOC1001*		2	0
chr7:131,900,275-131,985,174	84,899	q32.3	10	0	Loss	P5_S1	Complete	*LOC101928807, PLX*		2	0
chr7:140,394,575-140,558,494	163,919	q34	10	12	Gain	P5_S1	Complete	*ADCK2, NDUFB2-AS*		6	0
chr7:148,768,204-148,797,689	29,485	q36.1	10	23	Gain	P5_S1	Complete	*ZNF786*		1	0
chr7:152,009,324-152,345,483	336,159	q36.1	10	45	Gain	P5_S1	Complete	*KMT2C, FABP5P3, L*		5	0
chr7:155,285,173-155,314,636	29,463	q36.3	10	0	Loss	P5_S1	Complete	*CNPY1*		1	0
chr7:40,029,172-40,130,132	100,960	p14.1	10	100	Gain	P5_S1	Complete	*CDK13*		1	0
chr7:48,311,730-48,320,206	8,476	p12.3	10	100	Loss	P5_S1	Complete	*AX746840, ABCA13*		2	0
chr7:5,101,081-5,106,052	4,971	p22.1	10	0	Gain	P2_S2	Complete	*RBAK-RBAKDN, RBA*		2	0

chr7:55,881,932-56,035,062	153,130	p11.2	10	100	Gain	P5_S1	Complete	*SEPT14, DD413584,*		6	0
chr7:56,184,193-56,629,607	445,414	p11.2	10	100	Gain	P5_S1	Complete	*LOC650226, BC03625*		6	0
chr7:6,190,125-6,448,610	258,485	p22.1	10	3	Gain	P5_S1	Complete	*USP42, CYTH3, FAM*		4	0
chr7:6,495,558-6,621,273	125,715	p22.1	10	74	Gain	P5_S1	Complete	*DAGLB, KDELR2, Mi*		5	0
chr7:65,447,242-65,547,414	100,172	q11.21	10	100	Gain	P5_S1	Complete	*GUSB, ASL*		2	0
chr7:65,706,484-65,751,273	44,789	q11.21	10	100	Gain	P5_S1	Complete	*TPST1*		1	0
chr7:73,120,434-73,243,452	123,018	q11.23	10	0	Gain	P5_S1	Complete	*STX1A, MII*	hsa-mir-4	7	1
chr7:73,642,744-73,965,401	322,657	q11.23	10	5	Gain	P5_S1	Complete	*LAT2, RFC2, CLIP2,*		4	0
chr7:75,216,932-75,400,968	184,036	q11.23	10	59	Gain	P5_S1	Complete	*HIP1, CCL26*		2	0
chr7:75,861,603-75,952,263	90,660	q11.23	10	20	Gain	P5_S1	Complete	*SRRM3, BC063788, H*		4	0
chr8:0-196,605	196,605	p23.3	10	95	Loss	P5_S1	Complete	*OR4F21, RPL23AP53*		3	0
chr8:1,028,643-1,126,798	98,155	p23.3	10	7	Loss	P5_S1	Complete	*ERICH1-AS1, DLGAP*		2	0
chr8:10,396,009-10,403,553	7,544	p23.1	10	0	Loss	P5_S1	Complete	*AK307207, PRSS55*		2	0
chr8:133,882,045-133,899,304	17,259	q24.22	10	0	Loss	P5_S1	Complete	*TG*		1	0
chr8:133,930,820-133,958,777	27,957	q24.22	10	0	Loss	P5_S1	Complete	*TG*		1	0
chr8:134,013,871-134,034,462	20,591	q24.22	10	57	Loss	P5_S1	Complete	*TG*		1	0
chr8:140,281,276-140,300,478	19,202	q24.3	10	0	Loss	P5_S1	Complete			0	0
chr8:140,511,304-140,814,769	303,465	q24.3	10	5	Loss	P5_S1	Complete	*KCNK9, TRAPPC9*		2	0
chr8:142,950,195-143,248,497	298,302	q24.3	10	24	Loss	P5_S1	Complete	*MIR4539*		1	0
chr8:143,305,370-143,467,380	162,010	q24.3	10	4	Loss	P5_S1	Complete	*TSNARE1*		1	0
chr8:143,722,202-143,807,273	85,071	q24.3	10	100	Loss	P5_S1	Complete	*JH8, JRK, PSCA, AX7*		7	0
chr8:306,153-427,526	121,373	p23.3	10	100	Loss	P5_S1	Complete	*FAM87A, FBXO25, A*		3	0
chr8:528,232-576,712	48,480	p23.3	10	100	Loss	P5_S1	Complete	*ERICH1*		1	0
chr8:67,616,270-67,799,681	183,411	q13.1	10	6	Gain	P5_S1	Complete	*C8orf44-SGK3, PTTG*		4	0
chr8:95,733,740-95,780,710	46,970	q22.1	10	0	Gain	P5_S1	Complete	*DPY19L4*		1	0
chr8:98,666,152-98,760,861	94,709	q22.1	10	78	Gain	P5_S1	Complete	*MTDH*		1	0
chr9:114,461,681-114,572,107	110,426	q31.3	10	0	Gain	P5_S1	Complete	*SHOC1, C9orf84*		2	0
chr9:114,978,726-115,032,852	54,126	q32	10	1	Gain	P5_S1	Complete	*MIR3134, PTBP3*		2	0
chr9:127,697,256-127,945,280	248,024	q33.3	10	1	Gain	P5_S1	Complete	*GOLGA1, SCAI, AX74*		4	0
chr9:132,692,954-132,789,900	96,946	q34.11	10	100	Gain	P5_S1	Complete	*FNBP1*		1	0
chr9:137,595,893-137,827,039	231,146	q34.3	10	100	Loss	P5_S1	Complete	*COL5A1, LOC101448*		11	0
chr9:19,175,332-19,297,919	122,587	p22.1	10	11	Gain	P5_S1	Complete	*DQ572382, DENND4*		2	0
chr9:33,998,845-34,091,050	92,205	p13.3	10	10	Gain	P5_S1	Complete	*UBAP2, DCAF12*		2	0
chr9:36,341,059-36,484,826	143,767	p13.2	10	3	Gain	P5_S1	Complete	*RNF38*		1	0
chr9:65,629,772-69,238,239	3,608,467	12 - q21.1	10	100	Gain	P5_S1	Complete	*DQ600787,*	hsa-mir-1	39	1
chr9:9,471,004-9,504,185	33,181	p23	10	0	Loss	P1_S3	Complete	*PTPRD*		1	0
chrX:100,363,067-100,414,491	51,424	q22.1	10	13	Gain	P5_S1	Complete	*CENPI*		1	0
chrX:101,909,439-101,910,711	1,272	q22.1	10	0	Loss	P5_S1	Complete	*GPRASP1, ARMCX5-*		2	0
chrX:114,816,926-115,019,324	202,398	q23	10	96	Gain	P5_S1	Complete	*PLS3, DANT1, DANT*		3	0
chrX:122,796,182-123,212,503	416,321	q25	10	5	Gain	P5_S1	Complete	*THOC2, XIAP, LOC1*		4	0
chrX:23,786,007-24,255,687	469,680	p22.11	10	59	Gain	P5_S1	Complete	*Y_RNA, AK310094, S*		10	0
chrX:32,382,097-32,614,692	232,595	p21.1	10	23	AI;Loss	P1_S1;P1_S1	Complete;Com	*DMD, JA783589, JA7*		15	0
chrX:38,984,318-39,511,591	527,273	p11.4	10	0	Loss	P5_S1	Complete	*LINC01281, LOC2864*		3	0
chrX:40,920,989-41,284,003	363,014	p11.4	10	46	Gain	P5_S1	Complete	*USP9X, LINC02601,*		4	0
chrX:44,247,637-44,822,479	574,842	p11.3	10	16	Gain	P5_S1	Complete	*FUNDC1, DUSP21, K*		3	0
chrX:53,828,732-54,442,202	613,470	p11.22	10	100	Gain	P5_S1	Complete	*PHF8, FAM120C, WN*		3	0
chrX:6,511,946-8,184,869	1,672,923	p22.31	10	49	AI	P5_S1	Complete	*PUDP, HD*	hsa-mir-6	8	1

chrX:688,166-2,300,717	1,612,551	p22.33	10	38	Gain	P5_S1	Complete	*DQ576039, CSF2RA,*	13	0

Supplementary Table S2B. CNVs in 10 cranial meningiomas, including CNVs in chromosome 22. Chromosomal coordinates of the region (hg19); the region length in bp; the chromosomal cytoband; frequency of the region occurrence in the tumor samples; percent of the region's overlap with a known CNV; type of the variation (gain, loss, or allelic imbalance); sample ID; type of the participation of an individual CNV in the region (complete or partial); gene symbols; microRNA symbols; number of genes in the region; and the number of microRNA genes found in the region are shown from left to right.

Chromosomal region	Region length (bp)	Cytoband	Frequency of the CNV occurrence in samples (%)	Overlap with known CNVs (%)	Type of CNV	Sample ID	Participation	Gene symbols	miRNAs symbols	Gene count	miRNAs count
chr7:38,316,293-38,342,505	26,212	p14.1	100	100	Gain;Gain;Gain;Gai	P1_C1;P1_C2;P1_C3;P1_(	Complete;Complete;(	TRGC2, TARP, TCRGC2, T		5	0
chr14:22,718,405-22,953,551	235,146	q11.2	100	100	Gain;Gain;Gain;Gai	P1_C1;P1_C2;P1_C3;P1_(	Complete;Complete;(	AV2S1A1, TCRA, TCR-alph		25	0
chr22:50,281,051-50,477,175	196,124	q13.33	90	24	Loss;Loss;Loss;Los	P1_C1;P1_C2;P1_C3;P1_(	Complete;Complete;(	ZBED4, ALG12, CRELD2, I		7	0
chr22:49,912,616-50,044,155	131,539	q13.33	90	73	AI;AI;AI;AI;AI;AI;	P1_C1;P1_C2;P1_C3;P1_(	Complete;Complete;(	C22orf34, BC033837, MIR3(		3	0
chr22:49,642,233-49,742,092	99,859	q13.33	90	100	AI;AI;AI;AI;AI;AI;	P1_C1;P1_C2;P1_C3;P1_(	Complete;Complete;Complete;Complete;Complete			0	0
chr22:49,592,445-49,624,513	32,068	q13.33	90	100	Loss;Loss;Loss;Los	P1_C1;P1_C2;P1_C3;P1_(	Complete;Complete;Complete;Complete;Complete			0	0
chr22:49,535,046-49,624,513	89,467	q13.33	90	100	AI;AI;AI;AI;AI;AI;	P1_C1;P1_C2;P1_C3;P1_(	Complete;Complete;Complete;Complete;Complete			0	0
chr22:49,198,414-49,505,329	306,915	3.32 - q13.	90	64	AI;AI;AI;AI;AI;AI;	P1_C1;P1_C2;P1_C3;P1_(	Complete;Complete;(	LINC01310, LOC100128946		2	0
chr22:49,149,429-49,169,627	20,198	q13.32	90	58	Loss;Loss;Loss;Los	P1_C1;P1_C2;P1_C3;P1_(	Complete;Complete;Complete;Complete;Complete			0	0
chr22:49,068,967-49,169,627	100,660	q13.32	90	18	AI;AI;AI;AI;AI;AI;	P1_C1;P1_C2;P1_C3;P1_(	Complete;Complete;(	FAM19A5		1	0
chr22:48,749,859-48,843,831	93,972	q13.32	90	44	Loss;Loss;Loss;Los	P1_C1;P1_C2;P1_C3;P1_(	Complete;Complete;Complete;Complete;Complete			0	0
chr22:48,687,600-49,060,080	372,480	q13.32	90	67	AI;AI;AI;AI;AI;AI;	P1_C1;P1_C2;P1_C3;P1_(	Complete;Complete;(	LOC284933, FAM19A5		2	0
chr22:48,687,600-48,709,377	21,777	q13.32	90	0	Loss;Loss;Loss;Los	P1_C1;P1_C2;P1_C3;P1_(	Complete;Complete;Complete;Complete;Complete			0	0
chr22:48,538,908-48,599,737	60,829	q13.32	90	0	AI;AI;AI;AI;AI;AI;	P1_C1;P1_C2;P1_C3;P1_(	Complete;Complete;Complete;Complete;Complete			0	0
chr22:48,505,376-48,538,908	33,532	q13.32	90	13	Loss;Loss;Loss;Los	P1_C1;P1_C2;P1_C3;P1_(	Complete;Complete;Complete;Complete;Complete			0	0
chr22:48,212,878-48,505,376	292,498	3.31 - q13.	90	1	AI;AI;AI;AI;AI;AI;	P1_C1;P1_C2;P1_C3;P1_(	Complete;Complete;(	LOC284930, AK093107		2	0
chr22:47,849,916-47,947,554	97,638	q13.31	90	2	Loss;Loss;Loss;Los	P1_C1;P1_C2;P1_C3;P1_(	Complete;Complete;(	BC037972, LINC01644		2	0
chr22:46,739,190-46,765,794	26,604	q13.31	90	0	Loss;Loss;Loss;Los	P1_C1;P1_C2;P1_C3;P1_(	Complete;Complete;(	TRMU, CELSR1		2	0
chr22:46,656,514-46,685,380	28,866	q13.31	90	100	Loss;Loss;Loss;Los	P1_C1;P1_C2;P1_C3;P1_(	Complete;Complete;(	PKDREJ, TTC38		2	0
chr22:46,543,586-46,586,018	42,432	q13.31	90	7	Loss;Loss;Loss;Los	P1_C1;P1_C2;P1_C3;P1_(	Complete;Complete;(	PPARA		1	0
chr22:46,128,319-46,318,905	190,586	q13.31	90	15	Loss;Loss;Loss;Los	P1_C1;P1_C2;P1_C3;P1_(	Complete;Complete;(	ATXN10, MIR4762, WNT7I		3	0
chr22:45,725,061-45,812,446	87,385	q13.31	90	100	Loss;Loss;Loss;Los	P1_C1;P1_C2;P1_C3;P1_(	Complete;Complete;(	FAM118A, SMC1B, RIBC2		3	0
chr22:45,683,023-45,692,691	9,668	q13.31	90	100	Loss;Loss;Loss;Los	P1_C1;P1_C2;P1_C3;P1_(	Complete;Complete;(	UPK3A		1	0
chr22:45,583,923-45,692,691	108,768	q13.31	90	46	AI;AI;AI;AI;AI;AI;	P1_C1;P1_C2;P1_C3;P1_(	Complete;Complete;(	LOC105373064	hsa-mir-1249	4	1
chr22:45,507,574-45,583,923	76,349	q13.31	90	86	Loss;Loss;Loss;Los	P1_C1;P1_C2;P1_C3;P1_(	Complete;Complete;(	NUP50-DT, LOC100506714		4	0
chr22:45,411,124-45,507,574	96,450	q13.31	90	100	AI;AI;AI;AI;AI;AI;	P1_C1;P1_C2;P1_C3;P1_(	Complete;Complete;Complete;Complete;Complete			0	0
chr22:44,823,922-45,130,687	306,765	q13.31	90	36	AI;AI;AI;AI;AI;AI;	P1_C1;P1_C2;P1_C3;P1_(	Complete;Complete;(	LINC01656, LDOC1L, RTL(		7	0
chr22:44,759,597-44,780,495	20,898	q13.31	90	0	AI;AI;AI;AI;AI;AI;	P1_C1;P1_C2;P1_C3;P1_(	Complete;Complete;Complete;Complete;Complete			0	0
chr22:44,034,243-44,129,722	95,479	q13.2	90	7	Loss;Loss;Loss;Los	P1_C1;P1_C2;P1_C3;P1_(	Complete;Complete;(	EFCAB6		1	0
chr22:43,934,762-43,976,418	41,656	q13.2	90	0	Loss;Loss;Loss;Los	P1_C1;P1_C2;P1_C3;P1_(	Complete;Complete;(	EFCAB6		1	0
chr22:42,191,588-42,366,775	175,187	q13.2	90	0	Loss;Loss;Loss;Los	P1_C1;P1_C2;P1_C3;P1_(	Complete;Complete;(	MEI1, CCDC1	hsa-mir-33a	13	1
chr22:40,814,516-40,814,665	149	q13.1	90	0	Loss;Loss;Loss;Los	P1_C1;P1_C2;P1_C3;P1_(	Complete;Complete;(	MKL1, MRTFA		2	0
chr22:38,876,442-38,999,399	122,957	q13.1	90	3	Loss;Loss;Loss;Los	P1_C1;P1_C2;P1_C3;P1_(	Complete;Complete;(	KDELR3, DDX17, DMC1, L		5	0
chr22:38,217,697-38,336,933	119,236	q13.1	90	41	Loss;Loss;Loss;Los	P1_C1;P1_C2;P1_C3;P1_(	Complete;Complete;(	GALR3, ANKR	hsa-mir-658,	6	2
chr22:38,153,666-38,166,504	12,838	q13.1	90	100	Loss;Loss;Loss;Los	P1_C1;P1_C2;P1_C3;P1_(	Complete;Complete;(	TRIOBP		1	0
chr22:37,387,286-37,492,139	104,853	q12.3	90	0	AI;AI;AI;AI;AI;AI;	P1_C1;P1_C2;P1_C3;P1_(	Complete;Complete;(	TEX33, TST, MPST, KCTD		5	0
chr22:37,285,952-37,291,718	5,766	q12.3	90	100	AI;AI;AI;AI;AI;AI;	P1_C1;P1_C2;P1_C3;P1_(	Complete;Complete;Complete;Complete;Complete			0	0
chr22:37,199,414-37,250,905	51,491	q12.3	90	100	AI;AI;AI;AI;AI;AI;	P1_C1;P1_C2;P1_C3;P1_(	Complete;Complete;(	PVALB, NCF4-AS1		2	0
chr22:36,980,526-37,071,926	91,400	q12.3	90	37	AI;AI;AI;AI;AI;AI;	P1_C1;P1_C2;P1_C3;P1_(	Complete;Complete;(	CACNG2		1	0
chr22:36,138,391-36,445,634	307,243	q12.3	90	40	Loss;Loss;Loss;Los	P1_C1;P1_C2;P1_C3;P1_(	Complete;Complete;(	RBFOX2		1	0
chr22:35,813,220-35,828,876	15,656	q12.3	90	0	AI;AI;AI;AI;AI;AI;	P1_C1;P1_C2;P1_C3;P1_(	Complete;Complete;(	MCM5		1	0

chr22:35,492,141-35,779,143	287,002	q12.3	90	32	AI;AI;AI;AI;AI;AI;	P1_C1;P1_C2;P1_C3;P1_(	Complete;Complete;(	LINC01399, HMGXB4, TOM		6	0
chr22:34,266,771-35,393,163	1,126,392	q12.3	90	4	Loss;Loss;Loss;Los	P1_C1;P1_C2;P1_C3;P1_(	Complete;Complete;(	LARGE1, LARGE, ISX-AS1		3	0
chr22:34,190,829-35,445,501	1,254,672	q12.3	90	3	AI;AI;AI;AI;AI;AI;	P1_C1;P1_C2;P1_C3;P1_(	Complete;Complete;(	LARGE1, LARGE, ISX-AS1		3	0
chr22:33,978,193-34,069,344	91,151	q12.3	90	0	Loss;Loss;Loss;Los	P1_C1;P1_C2;P1_C3;P1_(	Complete;Complete;(	LARGE1, LARGE		2	0
chr22:33,532,291-33,823,431	291,140	q12.3	90	8	Loss;Loss;Loss;Los	P1_C1;P1_C2;P1_C3;P1_(	Complete;Complete;(	LARGE1, LARGE		2	0
chr22:33,462,428-33,978,193	515,765	q12.3	90	22	AI;AI;AI;AI;AI;AI;	P1_C1;P1_C2;P1_C3;P1_(	Complete;Complete;(	LINC01640, MIR4764, LAR		4	0
chr22:33,152,871-33,226,424	73,553	q12.3	90	0	Loss;Loss;Loss;Los	P1_C1;P1_C2;P1_C3;P1_(	Complete;Complete;(	SYN3, TIMP3		2	0
chr22:33,145,279-33,152,871	7,592	q12.3	90	0	AI;AI;AI;AI;AI;AI;	P1_C1;P1_C2;P1_C3;P1_(	Complete;Complete;(	SYN3		1	0
chr22:32,894,427-33,116,211	221,784	q12.3	90	1	AI;AI;AI;AI;AI;AI;	P1_C1;P1_C2;P1_C3;P1_(	Complete;Complete;(	FBXO7, AK123891, 5S_rRN		4	0
chr22:32,835,363-32,858,582	23,219	q12.3	90	0	AI;AI;AI;AI;AI;AI;	P1_C1;P1_C2;P1_C3;P1_(	Complete;Complete;(	BPIFC		1	0
chr22:32,791,777-32,835,363	43,586	q12.3	90	1	Loss;Loss;Loss;Los	P1_C1;P1_C2;P1_C3;P1_(	Complete;Complete;(	RTCB, BPIFC		2	0
chr22:32,624,735-32,791,777	167,042	q12.3	90	22	AI;AI;AI;AI;AI;AI;	P1_C1;P1_C2;P1_C3;P1_(	Complete;Complete;(	SLC5A4, SLC5A4-AS1, JB1		7	0
chr22:32,508,722-32,624,735	116,013	q12.3	90	47	Loss;Loss;Loss;Los	P1_C1;P1_C2;P1_C3;P1_(	Complete;Complete;(	SLC5A1, AP1B1P1, JB1750		7	0
chr22:32,439,351-32,508,722	69,371	q12.3	90	44	AI;AI;AI;AI;AI;AI;	P1_C1;P1_C2;P1_C3;P1_(	Complete;Complete;(	Mir_1302, SLC5A1		2	0
chr22:32,188,783-32,266,822	78,039	12.2 - q12.	90	1	Loss;Loss;Loss;Los	P1_C1;P1_C2;P1_C3;P1_(	Complete;Complete;(	DEPDC5		1	0
chr22:31,924,763-31,946,308	21,545	q12.2	90	0	Loss;Loss;Loss;Los	P1_C1;P1_C2;P1_C3;P1_(	Complete;Complete;(	SFI1		1	0
chr22:30,766,515-30,770,742	4,227	q12.2	90	0	Loss;Loss;Loss;Los	P1_C1;P1_C2;P1_C3;P1_(	Complete;Complete;(	CCDC157, KIAA1656		2	0
chr22:30,417,372-30,594,988	177,616	q12.2	90	17	AI;AI;AI;AI;AI;AI;	P1_C1;P1_C2;P1_C3;P1_(	Complete;Complete;(	MTMR3, HORMAD2-AS1,		3	0
chr22:30,252,969-30,417,372	164,403	q12.2	90	19	Loss;Loss;Loss;Los	P1_C1;P1_C2;P1_C3;P1_(	Complete;Complete;(	MTMR3, MIR6818, HORMA		3	0
chr22:29,726,436-30,252,969	526,533	q12.2	90	25	AI;AI;AI;AI;AI;AI;	P1_C1;P1_C2;P1_C3;P1_(	Complete;Complete;(	SNORD125, MIR3653, AP1		14	0
chr22:29,626,511-29,726,436	99,925	q12.2	90	7	Loss;Loss;Loss;Los	P1_C1;P1_C2;P1_C3;P1_(	Complete;Complete;(	EMID1, RHBDD3, EWSR1,		6	0
chr22:29,445,679-29,626,511	180,832	12.1 - q12.	90	3	AI;AI;AI;AI;AI;AI;	P1_C1;P1_C2;P1_C3;P1_(	Complete;Complete;(	ZNRF3, C22orf31, KREMEN		4	0
chr22:29,182,334-29,288,580	106,246	q12.1	90	0	AI;AI;AI;AI;AI;AI;	P1_C1;P1_C2;P1_C3;P1_(	Complete;Complete;(	CCDC117, XBP1, ZNRF3		3	0
chr22:28,624,805-29,125,669	500,864	q12.1	90	8	AI;AI;AI;AI;AI;AI;	P1_C1;P1_C2;P1_C3;P1_(	Complete;Complete;(	TTC28, MIR5739, CHEK2		3	0
chr22:28,202,107-28,372,642	170,535	q12.1	90	0	AI;AI;AI;AI;AI;AI;	P1_C1;P1_C2;P1_C3;P1_(	Complete;Complete;(	AX748308, PIT	hsa-mir-3199	5	2
chr22:27,408,188-27,482,926	74,738	q12.1	90	1	Loss;Loss;Loss;Los	P1_C1;P1_C2;P1_C3;P1_(	Complete;Complete;(	AK055980, LOC284898		2	0
chr22:26,915,436-26,940,954	25,518	q12.1	90	0	Loss;Loss;Loss;Los	P1_C1;P1_C2;P1_C3;P1_(	Complete;Complete;(	TPST2		1	0
chr22:26,166,993-27,034,913	867,920	q12.1	90	3	AI;AI;AI;AI;AI;AI;	P1_C1;P1_C2;P1_C3;P1_(	Complete;Complete;(	MYO18B, SEZ	hsa-mir-548j	11	1
chr22:26,166,993-26,282,115	115,122	q12.1	90	0	Loss;Loss;Loss;Los	P1_C1;P1_C2;P1_C3;P1_(	Complete;Complete;(	MYO18B		1	0
chr22:25,927,190-26,160,351	233,161	q12.1	90	96	AI;AI;AI;AI;AI;AI;	P1_C1;P1_C2;P1_C3;P1_(	Complete;Complete;(	GRK3, ADRBK2, MYO18B		3	0
chr22:25,897,536-25,904,962	7,426	11.23 - q12	90	100	AI;AI;AI;AI;AI;AI;	P1_C1;P1_C2;P1_C3;P1_(	Complete;Complete;(	CRYBB2P1		1	0
chr7:142,378,751-142,457,152	78,401	q34	80	100	Gain;Gain;Gain;Gai	P1_C1;P1_C2;P1_C3;P1_(	Complete;Complete;(	TCRBV5S1A1T, TCRBV11S		10	0
chr22:50,979,540-50,988,851	9,311	q13.33	80	0	Loss;Loss;Loss;Los	P1_C1;P1_C2;P1_C3;P1_(	Complete;Complete;(	KLHDC7B		1	0
chr22:50,950,090-50,961,549	11,459	q13.33	80	0	Loss;Loss;Loss;Los	P1_C1;P1_C2;P1_C3;P1_(	Complete;Complete;(	NCAPH2		1	0
chr22:50,919,714-50,927,490	7,776	q13.33	80	0	Loss;Loss;Loss;Los	P1_C1;P1_C2;P1_C3;P1_(	Complete;Complete;(	ADM2, MIOX		2	0
chr22:50,561,954-50,566,869	4,915	q13.33	80	100	Loss;Loss;Loss;Los	P1_C1;P1_C2;P1_C3;P1_(	Complete;Complete;(	MOV10L1		1	0
chr22:50,051,768-50,106,099	54,331	q13.33	80	1	Loss;Loss;Loss;Los	P1_C1;P1_C2;P1_C3;P1_(	Complete;Complete;Complete;Complete;Complete			0	0
chr22:49,642,233-49,742,092	99,859	q13.33	80	100	AI;AI;AI;AI;AI;AI;	P1_C1;P1_C2;P1_C3;P1_(	Complete;Complete;Complete;Complete;Complete			0	0
chr22:47,646,775-47,764,670	117,895	q13.31	80	3	AI;AI;AI;AI;AI;AI;	P1_C1;P1_C3;P1_C4;P1_(	Complete;Complete;(	LOC339685		1	0
chr22:46,044,683-46,439,853	395,170	q13.31	80	18	AI;AI;AI;AI;AI;AI;	P1_C2;P1_C3;P1_C4;P1_(	Complete;Complete;(	ATXN10, MIR4762, WNT7I		5	0
chr22:45,315,538-45,353,260	37,722	q13.31	80	100	Loss;Loss;Loss;Los	P1_C1;P1_C2;P1_C3;P1_(	Complete;Complete;(	PHF21B		1	0
chr22:45,237,389-45,258,462	21,073	q13.31	80	67	Loss;Loss;Loss;Los	P1_C1;P1_C2;P1_C3;P1_(	Complete;Complete;(	PRR5-ARHGAP8, ARHGAI		2	0
chr22:45,190,310-45,216,244	25,934	q13.31	80	92	Loss;Loss;Loss;Los	P1_C1;P1_C2;P1_C3;P1_(	Complete;Complete;(	PRR5-ARHGAP8, ARHGAI		2	0
chr22:44,823,922-45,130,687	306,765	q13.31	80	36	AI;AI;AI;AI;AI;AI;	P1_C1;P1_C2;P1_C3;P1_(	Complete;Complete;(	LINC01656, LDOC1L, RTL(		7	0
chr22:44,759,597-44,780,495	20,898	q13.31	80	0	AI;AI;AI;AI;AI;AI;	P1_C1;P1_C2;P1_C3;P1_(	Complete;Complete;Complete;Complete;Complete			0	0
chr22:44,702,303-44,744,197	41,894	q13.31	80	9	AI;AI;AI;AI;AI;AI;	P1_C1;P1_C2;P1_C4;P1_(	Complete;Complete;(	SHISAL1, KIAA1644		2	0
chr22:44,521,645-44,553,772	32,127	q13.31	80	4	AI;AI;AI;AI;AI;AI;	P1_C2;P1_C3;P1_C4;P1_(	Complete;Complete;(	PARVB, TRNA_SeC		2	0
chr22:44,394,082-44,421,494	27,412	q13.31	80	15	Loss;Loss;Loss;Los	P1_C1;P1_C2;P1_C3;P1_(	Complete;Complete;(	PARVB		1	0
chr22:44,285,570-44,495,208	209,638	q13.31	80	6	AI;AI;AI;AI;AI;AI;	P1_C1;P1_C3;P1_C4;P1_(	Complete;Complete;(	PNPLA5, PNPLA3, SAMM5		5	0
chr22:44,285,570-44,342,134	56,564	q13.31	80	0	Loss;Loss;Loss;Los	P1_C1;P1_C2;P1_C3;P1_(	Complete;Complete;(	PNPLA5, PNPLA3		2	0

chr22:44,213,905-44,244,997	31,092	q13.31	80	5	AI;AI;AI;AI;AI;AI;	P1_C2;P1_C3;P1_C4;P1_	Complete;Complete;	AX747137, SULT4A1		2	0
chr22:43,768,475-43,926,536	158,061	q13.2	80	3	Loss;Loss;Loss;Los	P1_C1;P1_C2;P1_C3;P1_	Complete;Complete;	LINC01639, MPPED1, EFC		4	0
chr22:43,167,430-43,213,537	46,107	q13.2	80	24	Loss;Loss;Loss;Los	P1_C1;P1_C2;P1_C3;P1_	Complete;Complete;	CS330190, DQ595055, ARF		3	0
chr22:42,795,566-42,890,315	94,749	q13.2	80	100	Loss;Loss;Loss;Los	P1_C1;P1_C2;P1_C3;P1_	Complete;Complete;	NFAM1		1	0
chr22:42,486,744-42,522,673	35,929	q13.2	80	28	Loss;Loss;Loss;Los	P1_C1;P1_C2;P1_C3;P1_	Complete;Complete;	NDUFA6, NDUFA6-DT, ND		5	0
chr22:40,662,913-41,403,299	740,386	13.1 - q13.	80	6	AI;AI;AI;AI;AI;AI;	P1_C1;P1_C2;P1_C3;P1_	Complete;Complete;	TNRC6B, ADSL, SGSM3, M		14	0
chr22:40,032,285-40,501,218	468,933	q13.1	80	21	AI;AI;AI;AI;AI;AI;	P1_C1;P1_C2;P1_C3;P1_	Complete;Complete;	CACNA1I, ENTHD1, GRAP		6	0
chr22:39,927,188-39,970,151	42,963	q13.1	80	22	Loss;Loss;Loss;Los	P1_C1;P1_C2;P1_C3;P1_	Complete;Complete;	RPS19BP1, CACNA1I		2	0
chr22:39,276,559-39,340,959	64,400	q13.1	80	18	Loss;Loss;Loss;Los	P1_C1;P1_C2;P1_C3;P1_	Complete;Complete;Complete;Complete;Complete			0	0
chr22:38,364,378-38,510,877	146,499	q13.1	80	1	Loss;Loss;Loss;Los	P1_C1;P1_C2;P1_C3;P1_	Complete;Complete;	SOX10, AK098727, MIR453		8	0
chr22:38,010,853-38,109,336	98,483	q13.1	80	100	Loss;Loss;Loss;Los	P1_C1;P1_C2;P1_C3;P1_	Complete;Complete;	GGA1, LOC101927051, AK		9	0
chr22:37,794,788-37,911,602	116,814	q13.1	80	1	Loss;Loss;Loss;Los	P1_C1;P1_C2;P1_C3;P1_	Complete;Complete;	ELFN2, MFNG, CARD10		3	0
chr22:37,609,894-37,696,119	86,225	q13.1	80	8	Loss;Loss;Loss;Los	P1_C1;P1_C2;P1_C3;P1_	Complete;Complete;	RAC2, CYTH4		2	0
chr22:37,599,507-37,603,601	4,094	12.3 - q13.	80	0	Loss;Loss;Loss;Los	P1_C1;P1_C2;P1_C4;P1_	Complete;Complete;	SSTR3		1	0
chr22:37,387,088-37,387,286	198	q12.3	80	0	Loss;Loss;Loss;Los	P1_C1;P1_C2;P1_C3;P1_	Complete;Complete;	TEX33		1	0
chr22:37,291,718-37,370,471	78,753	q12.3	80	88	Loss;Loss;Loss;Los	P1_C1;P1_C2;P1_C3;P1_	Complete;Complete;	CSF2RB, LOC100506241, L		3	0
chr22:37,093,963-37,199,414	105,451	q12.3	80	72	Loss;Loss;Loss;Los	P1_C1;P1_C2;P1_C3;P1_	Complete;Complete;	CACNG2, AK123632, LOC		6	0
chr22:36,587,795-36,627,114	39,319	q12.3	80	36	Loss;Loss;Loss;Los	P1_C1;P1_C2;P1_C3;P1_	Complete;Complete;	APOL4, APOL2		2	0
chr22:35,946,138-35,982,736	36,598	q12.3	80	0	Loss;Loss;Loss;Los	P1_C1;P1_C2;P1_C3;P1_	Complete;Complete;	RASD2		1	0
chr22:35,828,876-35,907,566	78,690	q12.3	80	1	Loss;Loss;Loss;Los	P1_C1;P1_C2;P1_C3;P1_	Complete;Complete;Complete;Complete;Complete			0	0
chr22:35,492,141-35,742,724	250,583	q12.3	80	33	Loss;Loss;Loss;Los	P1_C1;P1_C2;P1_C3;P1_	Complete;Complete;	LINC01399, HMGXB4, TO		5	0
chr22:33,263,183-33,321,033	57,850	q12.3	80	4	AI;AI;AI;AI;AI;AI;	P1_C1;P1_C2;P1_C3;P1_	Complete;Complete;	SYN3		1	0
chr22:32,894,427-33,116,211	221,784	q12.3	80	1	AI;AI;AI;AI;AI;AI;	P1_C1;P1_C2;P1_C3;P1_	Complete;Complete;	FBXO7, AK123891, 5S_rR		4	0
chr22:32,266,822-32,270,300	3,478	q12.3	80	0	AI;AI;AI;AI;AI;AI;	P1_C1;P1_C2;P1_C3;P1_	Complete;Complete;	DEPDC5		1	0
chr22:31,981,848-32,014,851	33,003	q12.2	80	100	Loss;Loss;Loss;Los	P1_C1;P1_C2;P1_C3;P1_	Complete;Complete;	SFI1, PISD		2	0
chr22:31,514,266-31,532,798	18,532	q12.2	80	0	Loss;Loss;Loss;Los	P1_C1;P1_C2;P1_C3;P1_	Complete;Complete;	INPP5J, PLA2G3		2	0
chr22:31,331,893-31,486,489	154,596	q12.2	80	19	Loss;Loss;Loss;Los	P1_C1;P1_C2;P1_C3;P1_	Complete;Complete;	MORC2, TUG1, SMTN		3	0
chr22:31,108,420-31,254,092	145,672	q12.2	80	12	Loss;Loss;Loss;Los	P1_C1;P1_C2;P1_C3;P1_	Complete;Complete;	MIR3200, OSB	hsa-mir-320	2	1
chr22:30,863,216-30,951,573	88,357	q12.2	80	2	Loss;Loss;Loss;Los	P1_C1;P1_C2;P1_C3;P1_	Complete;Complete;	SEC14L3, SDC4P, SEC14L4		5	0
chr22:30,661,895-30,672,894	10,999	q12.2	80	0	AI;AI;AI;AI;AI;AI;	P1_C1;P1_C2;P1_C4;P1_	Complete;Complete;	OSM		1	0
chr22:28,084,119-28,100,489	16,370	q12.1	80	0	AI;AI;AI;AI;AI;AI;	P1_C1;P1_C2;P1_C4;P1_	Complete;Complete;Complete;Complete;Complete			0	0
chr22:28,012,256-28,063,368	51,112	q12.1	80	0	Loss;Loss;Loss;Los	P1_C1;P1_C2;P1_C3;P1_	Complete;Complete;Complete;Complete;Complete			0	0
chr22:27,955,271-27,966,012	10,741	q12.1	80	0	AI;AI;AI;AI;AI;AI;	P1_C1;P1_C2;P1_C4;P1_	Complete;Complete;Complete;Complete;Complete			0	0
chr22:27,865,630-27,895,250	29,620	q12.1	80	0	AI;AI;AI;AI;AI;AI;	P1_C2;P1_C3;P1_C4;P1_	Complete;Complete;Complete;Complete;Complete			0	0
chr22:27,787,130-27,808,250	21,120	q12.1	80	3	AI;AI;AI;AI;AI;AI;	P1_C2;P1_C3;P1_C4;P1_	Complete;Complete;Complete;Complete;Complete			0	0
chr22:27,708,582-27,787,130	78,548	q12.1	80	0	Loss;Loss;Loss;Los	P1_C1;P1_C2;P1_C3;P1_	Complete;Complete;	CR936633, LINC02554		2	0
chr22:27,169,036-27,171,181	2,145	q12.1	80	37	Loss;Loss;Loss;Los	P1_C1;P1_C2;P1_C3;P1_	Complete;Complete;	MIATNB		1	0
chr22:27,072,717-27,171,181	98,464	q12.1	80	4	AI;AI;AI;AI;AI;AI;	P1_C1;P1_C2;P1_C4;P1_	Complete;Complete;	AK026502, MIATNB		2	0
chr22:25,283,797-25,332,835	49,038	q11.23	80	0	AI;AI;AI;AI;AI;AI;	P1_C1;P1_C2;P1_C3;P1_	Complete;Complete;	SGSM1, TMEM211		2	0
chr22:25,131,186-25,223,774	92,588	q11.23	80	4	AI;AI;AI;AI;AI;AI;	P1_C1;P1_C2;P1_C3;P1_	Complete;Complete;	PIWIL3, TOP1P2, SGSM1		3	0
chr22:24,973,696-25,131,186	157,490	q11.23	80	68	Loss;Loss;Loss;Los	P1_C1;P1_C2;P1_C3;P1_	Complete;Complete;	SNRPD3, FAM211B, LRRC		8	0
chr22:24,608,626-24,973,696	365,070	q11.23	80	100	AI;AI;AI;AI;AI;AI;	P1_C1;P1_C2;P1_C3;P1_	Complete;Complete;	GGT5, UNQ2565, POM121L		12	0
chr22:24,220,027-24,248,926	28,899	q11.23	80	100	Loss;Loss;Loss;Los	P1_C1;P1_C2;P1_C3;P1_	Complete;Complete;	SLC2A11, DQ601926, MIF,		12	0
chr22:23,271,953-23,633,547	361,594	1.22 - q11.	80	27	AI;AI;AI;AI;AI;AI;	P1_C1;P1_C2;P1_C3;P1_	Complete;Complete;	GNAZ, RSPH14, RTDR1, R		6	0
chr22:22,349,526-22,791,105	441,579	q11.22	80	100	AI;AI;AI;AI;AI;AI;	P1_C1;P1_C2;P1_C3;P1_	Complete;Complete;	PRAMENP, AK131325, BC		8	0
chr22:22,349,526-22,531,323	181,797	q11.22	80	100	Loss;Loss;Loss;Los	P1_C1;P1_C2;P1_C3;P1_	Complete;Complete;	PRAMENP, AK131325, BC		4	0
chr22:22,034,751-22,314,591	279,840	1.21 - q11.	80	55	Loss;Loss;Loss;Los	P1_C1;P1_C2;P1_C3;P1_	Complete;Complete;	PPIL2, YPEL1, MAPK1, PP		6	0
chr22:51,044,109-51,065,042	20,933	q13.33	70	0	Loss;Loss;Loss;Los	P1_C1;P1_C2;P1_C4;P1_	Complete;Complete;	MAPK8IP2, ARSA		2	0
chr22:50,656,629-50,661,423	4,794	q13.33	70	0	Loss;Loss;Loss;Los	P1_C1;P1_C2;P1_C3;P1_	Complete;Complete;	TUBGCP6		1	0
chr22:50,515,289-50,537,894	22,605	q13.33	70	100	Loss;Loss;Loss;Los	P1_C1;P1_C2;P1_C3;P1_	Complete;Complete;	MLC1, MOV10L1		2	0

chr22:50,187,114-50,189,615	2,501	q13.33	70	100	AI;AI;AI;AI;AI;AI;	P1_C1;P1_C3;P1_C4;P1_	Complete;Complete;	BRD1		1	0
chr22:49,805,641-49,825,892	20,251	q13.33	70	100	Loss;Loss;Loss;Los	P1_C1;P1_C2;P1_C3;P1_	Complete;Complete;	C22orf34, BC033837		2	0
chr22:47,344,809-47,413,091	68,282	q13.31	70	66	AI;AI;AI;AI;AI;AI;	P1_C1;P1_C2;P1_C5;P3_	Complete;Complete;	TBC1D22A		1	0
chr22:47,150,677-47,273,824	123,147	q13.31	70	2	AI;AI;AI;AI;AI;AI;	P1_C2;P1_C3;P1_C4;P3_	Complete;Complete;	TBC1D22A		1	0
chr22:47,086,006-47,119,026	33,020	q13.31	70	0	Loss;Loss;Loss;Los	P1_C1;P1_C2;P1_C3;P1_	Complete;Complete;	CERK		1	0
chr22:46,872,061-46,893,817	21,756	q13.31	70	10	Loss;Loss;Loss;Los	P1_C1;P1_C2;P1_C3;P1_	Complete;Complete;	CELSR1		1	0
chr22:45,284,520-45,309,730	25,210	q13.31	70	100	Loss;Loss;Loss;Los	P1_C1;P1_C2;P1_C4;P1_	Complete;Complete;	PHF21B		1	0
chr22:44,702,303-44,744,197	41,894	q13.31	70	9	AI;AI;AI;AI;AI;AI;	P1_C1;P1_C2;P1_C4;P1_	Complete;Complete;	SHISAL1, KIAA1644		2	0
chr22:43,603,135-43,639,085	35,950	q13.2	70	100	Loss;Loss;Loss;Los	P1_C1;P1_C2;P1_C3;P1_	Complete;Complete;	LOC105373051, SCUBE1		2	0
chr22:43,498,137-43,563,649	65,512	q13.2	70	82	AI;AI;AI;AI;AI;AI;	P1_C1;P1_C2;P1_C5;P3_	Complete;Complete;	BIK, MCAT, TSPO, TTLL12		4	0
chr22:41,621,683-41,740,831	119,148	q13.2	70	78	AI;AI;AI;AI;AI;AI;	P1_C1;P1_C2;P1_C3;P1_	Complete;Complete;	L3MBTL2, CHADL, MIR68		5	0
chr22:39,757,115-39,886,305	129,190	q13.1	70	0	AI;AI;AI;AI;AI;AI;	P1_C1;P1_C2;P1_C3;P1_	Complete;Complete;	SYNGR1, TAB1, LOC10050		5	0
chr22:39,127,628-39,132,048	4,420	q13.1	70	100	Loss;Loss;Loss;Los	P1_C1;P1_C2;P1_C3;P1_	Complete;Complete;	GTPBP1, SUN2		2	0
chr22:37,543,503-37,578,529	35,026	q12.3	70	2	Loss;Loss;Loss;Los	P1_C1;P1_C2;P1_C4;P1_	Complete;Complete;	IL2RB, C1QTNF6		2	0
chr22:36,661,819-36,670,589	8,770	q12.3	70	100	Loss;Loss;Loss;Los	P1_C1;P1_C2;P1_C4;P1_	Complete;Complete;	APOL1		1	0
chr22:34,130,684-34,134,002	3,318	q12.3	70	0	AI;AI;AI;AI;AI;AI;	P1_C3;P1_C4;P1_C5;P3_	Complete;Complete;	LARGE1, LARGE, LARGE-		3	0
chr22:34,088,957-34,105,225	16,268	q12.3	70	0	Loss;Loss;Loss;Los	P1_C1;P1_C2;P1_C3;P1_	Complete;Complete;	LARGE1, LARGE, SNORA		4	0
chr22:33,263,183-33,321,033	57,850	q12.3	70	4	AI;AI;AI;AI;AI;AI;	P1_C1;P1_C2;P1_C3;P1_	Complete;Complete;	SYN3		1	0
chr22:31,535,988-31,824,599	288,611	q12.2	70	4	Loss;Loss;Loss;Los	P1_C1;P1_C2;P1_C3;P1_	Complete;Complete;	PLA2G3, MIR3928, RNF185		12	0
chr22:30,770,742-30,851,738	80,996	q12.2	70	0	AI;AI;AI;AI;AI;AI;	P1_C3;P1_C4;P1_C5;P3_	Complete;Complete;	CCDC157, KIAA1656, RNF		6	0
chr22:30,661,895-30,672,894	10,999	q12.2	70	0	AI;AI;AI;AI;AI;AI;	P1_C1;P1_C2;P1_C4;P1_	Complete;Complete;	OSM		1	0
chr22:28,202,107-28,372,642	170,535	q12.1	70	0	AI;AI;AI;AI;AI;AI;	P1_C1;P1_C2;P1_C3;P1_	Complete;Complete;	AX748308, PIT	hsa-mir-3199	5	2
chr22:28,170,453-28,190,592	20,139	q12.1	70	31	AI;AI;AI;AI;AI;AI;	P1_C1;P1_C4;P1_C5;P3_	Complete;Complete;	MN1		1	0
chr22:27,541,022-27,669,385	128,363	q12.1	70	1	Loss;Loss;Loss;Los	P1_C1;P1_C2;P1_C4;P1_	Complete;Complete;	LOC105372977, LINC01638		3	0
chr22:27,500,135-27,520,689	20,554	q12.1	70	0	Loss;Loss;Loss;Los	P1_C2;P1_C4;P1_C5;P3_	Complete;Complete;Complete;Complete;Complete			0	0
chr22:25,283,797-25,332,835	49,038	q11.23	70	0	AI;AI;AI;AI;AI;AI;	P1_C1;P1_C2;P1_C3;P1_	Complete;Complete;	SGSM1, TMEM211		2	0
chr22:23,918,975-24,039,414	120,439	q11.23	70	100	Loss;Loss;Loss;Los	P1_C1;P1_C2;P1_C3;P1_	Complete;Complete;	IGLL1, DQ586720, C22orf4		6	0
chr22:23,700,773-23,853,388	152,615	q11.23	70	100	AI;AI;AI;AI;AI;AI;	P1_C2;P1_C3;P1_C4;P1_	Complete;Complete;	CES5AP1, ZDHHC8P1, LIN		5	0
chr7:61,747,794-62,359,388	611,594	q11.21	60	100	Gain;Gain;Gain;Gai	P1_C1;P1_C2;P1_C3;P1_	Complete;Complete;Complete;Complete;Complete			0	0
chr7:142,459,821-142,460,843	1,022	q34	60	100	Gain;Gain;Gain;Gai	P1_C2;P1_C4;P1_C5;P3_	Complete;Complete;	TCRBV5S1A1T, TCRBV2S		5	0
chr22:51,156,372-51,304,566	148,194	q13.33	60	57	Loss;Loss;Loss;Los	P1_C1;P1_C2;P1_C3;P1_	Complete;Complete;	SHANK3, BC050343, LOC1		6	0
chr22:50,687,978-50,715,719	27,741	q13.33	60	0	Loss;Loss;Loss;Los	P1_C1;P1_C2;P1_C4;P1_	Complete;Complete;	HDAC10, MAPK12, MAPK		4	0
chr22:50,477,175-50,566,869	89,694	q13.33	60	100	AI;AI;AI;AI;AI;AI	P1_C3;P1_C4;P1_C5;P3_	Complete;Complete;	TTLL8, MLC1, MOV10L1		3	0
chr22:50,187,114-50,189,615	2,501	q13.33	60	100	AI;AI;AI;AI;AI;AI;	P1_C1;P1_C3;P1_C4;P1_	Complete;Complete;	BRD1		1	0
chr22:47,413,091-47,563,032	149,941	q13.31	60	100	Loss;Loss;Loss;Los	P1_C1;P1_C2;P1_C3;P1_	Complete;Complete;	TBC1D22A		1	0
chr22:45,896,059-45,968,777	72,718	q13.31	60	24	AI;AI;AI;AI;AI;AI	P1_C2;P1_C4;P3_C1;P3_	Complete;Complete;	FBLN1		1	0
chr22:45,826,456-45,896,059	69,603	q13.31	60	100	Loss;Loss;Loss;Los	P1_C1;P1_C2;P1_C3;P1_	Complete;Complete;	RIBC2		1	0
chr22:44,557,937-44,611,651	53,714	q13.31	60	6	Loss;Loss;Loss;Los	P1_C1;P1_C2;P1_C3;P1_	Complete;Complete;	PARVB, PARVG		2	0
chr22:44,521,645-44,553,772	32,127	q13.31	60	4	AI;AI;AI;AI;AI;AI;	P1_C2;P1_C3;P1_C4;P1_	Complete;Complete;	PARVB, TRNA_SeC		2	0
chr22:44,032,504-44,034,035	1,531	q13.2	60	1	AI;AI;AI;AI;AI;AI	P1_C3;P1_C5;P3_C1;P3_	Complete;Complete;	EFCAB6		1	0
chr22:43,167,430-43,434,324	266,894	q13.2	60	6	AI;AI;AI;AI;AI;AI	P1_C1;P1_C2;P1_C5;P3_	Complete;Complete;	CS330190, DQ595055, ARF		4	0
chr22:42,366,775-42,453,880	87,105	q13.2	60	14	AI;AI;AI;AI;AI;AI	P1_C1;P1_C3;P1_C4;P1_	Complete;Complete;	SEPT3, WBP2NL		2	0
chr22:41,621,683-41,740,831	119,148	q13.2	60	78	AI;AI;AI;AI;AI;AI;	P1_C1;P1_C2;P1_C3;P1_	Complete;Complete;	L3MBTL2, CHADL, MIR68		5	0
chr22:39,440,420-39,440,933	513	q13.1	60	100	Loss;Loss;Loss;Los	P1_C1;P1_C2;P1_C3;P1_	Complete;Complete;	APOBEC3F, APOBEC3G		2	0
chr22:39,207,355-39,226,975	19,620	q13.1	60	0	Loss;Loss;Loss;Los	P1_C1;P1_C2;P1_C4;P1_	Complete;Complete;	NPTXR		1	0
chr22:37,523,864-37,532,724	8,860	q12.3	60	0	Loss;Loss;Loss;Los	P1_C1;P1_C2;P1_C3;P1_	Complete;Complete;	IL2RB		1	0
chr22:37,261,056-37,273,391	12,335	q12.3	60	100	Loss;Loss;Loss;Los	P1_C1;P1_C2;P1_C3;P1_	Complete;Complete;	NCF4-AS1, NCF4		2	0
chr22:36,445,634-36,700,131	254,497	q12.3	60	55	AI;AI;AI;AI;AI;AI	P1_C4;P1_C5;P3_C1;P3_	Complete;Complete;	APOL3, APOL4, APOL2, A		7	0
chr22:36,135,817-36,138,391	2,574	q12.3	60	100	AI;AI;AI;AI;AI;AI	P1_C4;P1_C5;P3_C1;P3_	Complete;Complete;	RBFOX2		1	0
chr22:27,899,740-27,921,860	22,120	q12.1	60	0	Loss;Loss;Loss;Los	P1_C1;P1_C2;P1_C3;P1_	Complete;Complete;Complete;Complete;Complete			0	0

chr22:27,251,774-27,291,880	40,106	q12.1	60	7	Loss;Loss;Loss;Los	P1_C2;P1_C4;P1_C5;P3_(	Complete;Complete;(	LOC110091768		1	0
chr22:24,087,107-24,142,678	55,571	q11.23	60	100	Loss;Loss;Loss;Los	P1_C1;P1_C2;P1_C3;P1_(	Complete;Complete;(	ZNF70, VPREB3, C22orf15,		6	0
chr22:23,271,953-23,633,547	361,594	1.22 - q11.	60	27	AI;AI;AI;AI;AI;AI;	P1_C1;P1_C2;P1_C3;P1_(	Complete;Complete;(	GNAZ, RSPH14, RTDR1, R.		6	0
chr22:21,975,126-21,977,306	2,180	q11.21	60	77	AI;AI;AI;AI;AI;AI	P1_C1;P1_C2;P1_C4;P3_(	Complete;Complete;(	UBE2L3		1	0
chr22:20,970,530-21,002,516	31,986	q11.21	60	100	AI;AI;AI;AI;AI;AI	P1_C2;P1_C4;P1_C5;P3_(	Complete;Complete;(	BC035867		1	0
chr22:19,605,170-19,833,903	228,733	q11.21	60	88	AI;AI;AI;AI;AI;AI	P1_C1;P1_C2;P1_C4;P3_(	Complete;Complete;(	SEPT5, SEPT5-GP1BB, GP1		7	0
chr22:18,640,401-18,914,012	273,611	q11.21	60	100	AI;AI;AI;AI;AI;AI	P1_C1;P1_C2;P3_C1;P3_(	Complete;Complete;(	USP18, LOC105379550, AK		15	0
chr22:17,775,868-17,942,778	166,910	11.1 - q11.2	60	4	AI;AI;AI;AI;AI;AI	P1_C1;P1_C2;P1_C4;P3_(	Complete;Complete;(	CECR2		1	0
chr12:37,961,768-38,255,277	293,509	q11 - q12	60	100	Gain;Gain;Gain;Gai	P1_C1;P1_C2;P1_C3;P1_(	Complete;Complete;Complete;Complete;Complete			0	0
chr22:50,912,557-50,919,714	7,157	q13.33	50	0	AI;AI;AI;AI;AI	P1_C3;P1_C5;P3_C1;P3_(	Complete;Complete;(	SBF1		1	0
chr22:50,728,699-50,750,702	22,003	q13.33	50	0	Loss;Loss;Loss;Los	P1_C2;P1_C4;P1_C5;P3_(	Complete;Complete;(	PLXNB2, DENND6B		2	0
chr22:50,647,092-50,662,657	15,565	q13.33	50	0	AI;AI;AI;AI;AI	P1_C4;P1_C5;P3_C1;P3_(	Complete;Complete;(	SELENOO, SELO, TUBGCI		3	0
chr22:50,647,092-50,655,711	8,619	q13.33	50	0	Loss;Loss;Loss;Los	P1_C4;P1_C5;P3_C1;P3_(	Complete;Complete;(	SELENOO, SELO		2	0
chr22:46,938,735-46,979,986	41,251	q13.31	50	4	Loss;Loss;Loss;Los	P1_C1;P1_C2;P1_C4;P1_(	Complete;Complete;(	GRAMD4		1	0
chr22:46,931,363-47,062,485	131,122	q13.31	50	9	AI;AI;AI;AI;AI	P1_C4;P3_C1;P3_C2;P3_(	Complete;Complete;(	CELSR1, GRAMD4		2	0
chr22:46,893,817-46,923,646	29,829	q13.31	50	0	AI;AI;AI;AI;AI	P1_C4;P1_C5;P3_C1;P3_(	Complete;Complete;(	CELSR1		1	0
chr22:42,961,466-42,981,017	19,551	q13.2	50	100	Loss;Loss;Loss;Los	P1_C2;P1_C4;P1_C5;P3_(	Complete;Complete;(	SERHL2, RRP7B, RRP7BP,		4	0
chr22:39,481,605-39,494,815	13,210	q13.1	50	0	Loss;Loss;Loss;Los	P1_C2;P1_C3;P1_C4;P1_(	Complete;Complete;(	APOBEC3G, APOBEC3H		2	0
chr22:37,603,601-37,699,423	95,822	q13.1	50	8	AI;AI;AI;AI;AI	P1_C5;P3_C1;P3_C2;P3_(	Complete;Complete;(	SSTR3, RAC2, CYTH4		3	0
chr22:36,879,901-36,893,147	13,246	q12.3	50	100	AI;AI;AI;AI;AI;Los	P1_C4;P1_C5;P3_C1;P3_(	Complete;Complete;(	FOXRED2		1	0
chr22:31,842,284-32,014,851	172,567	q12.2	50	24	AI;AI;AI;AI;AI	P1_C1;P1_C2;P1_C3;P1_(	Complete;Complete;(	EIF4ENIF1, SFI1, PISD		3	0
chr22:31,535,988-31,840,955	304,967	q12.2	50	4	AI;AI;AI;AI;AI	P1_C2;P1_C3;P1_C5;P3_(	Complete;Complete;(	PLA2G3, MIR3928, RNF185		14	0
chr22:24,220,027-24,487,738	267,711	q11.23	50	100	AI;AI;AI;AI;AI	P1_C2;P1_C5;P3_C1;P3_(	Complete;Complete;(	SLC2A11, DQ601926, MIF,		26	0
chr22:21,458,848-21,800,427	341,579	q11.21	50	100	Loss;Loss;Loss;Los	P1_C1;P1_C2;P1_C4;P3_(	Complete;Complete;(	BCRP2, POM121L7, DQ570		17	0
chr22:21,125,309-21,141,584	16,275	q11.21	50	100	AI;AI;AI;AI;AI	P1_C2;P1_C4;P1_C5;P3_(	Complete;Complete;(	SERPIND1, PI4KA		2	0
chr22:19,135,021-19,183,186	48,165	q11.21	50	0	AI;AI;AI;AI;AI	P1_C1;P1_C2;P1_C4;P3_(	Complete;Complete;(	GSC2, LINC01311, SLC25A		4	0
chr22:17,445,606-17,549,874	104,268	q11.1	50	100	AI;AI;AI;AI;AI;Los	P1_C1;P1_C2;P1_C4;P3_(	Complete;Complete;(	GAB4, CECR7		2	0
chr22:17,315,373-17,435,507	120,134	q11.1	50	100	Loss;Loss;Loss;Los	P1_C1;P1_C2;P1_C4;P3_(	Complete;Complete;(	IGKV1-12		1	0
chr11:50,472,623-50,697,498	224,875	p11.12	50	100	Gain;Gain;Gain;Gai	P1_C1;P1_C3;P1_C5;P6_(	Complete;Complete;Complete;Complete;Complete			0	0
chr10:47,593,880-47,694,995	101,115	q11.22	50	100	AI;AI;AI;AI;AI	P1_C1;P1_C2;P1_C3;P1_(	Complete;Complete;(	ANTXRLP1, AK057316, AN		3	0
chr7:48,315,976-48,318,967	2,991	p12.3	40	100	Loss;Loss;Loss;Los	P1_C1;P1_C3;P6_C1;P7_(	Complete;Complete;(	AX746840, ABCA13		2	0
chr22:50,861,422-50,898,259	36,837	q13.33	40	0	AI;AI;AI;AI	P1_C3;P1_C4;P1_C5;P3_(	Complete;Complete;(	PPP6R2, AB372727, SBF1		3	0
chr22:42,689,981-42,890,315	200,334	q13.2	40	65	AI;AI;AI;AI	P1_C5;P3_C1;P3_C2;P3_(	Complete;Complete;(	LINC01315, BC038245, NF/		3	0
chr22:39,583,599-39,620,068	36,469	q13.1	40	1	Loss;Loss;Loss;Los	P1_C1;P1_C2;P1_C4;P1_(	Complete;Complete;(	PDGFB		1	0
chr22:38,611,993-38,616,133	4,140	q13.1	40	0	Loss;Loss;Loss;Los	P1_C2;P1_C4;P1_C5;P6_(	Complete;Complete;(	MAFF, BC127823, DKFZp6		4	0
chr22:38,522,410-38,679,172	156,762	q13.1	40	1	AI;AI;AI;AI	P1_C5;P3_C1;P3_C2;P3_(	Complete;Complete;(	PLA2G6, MAFF, BC127823		6	0
chr22:21,134,286-21,141,584	7,298	q11.21	40	100	Loss;Loss;Loss;Los	P1_C1;P1_C2;P1_C4;P3_(	Complete;Complete;(	SERPIND1, PI4KA		2	0
chr22:20,810,864-21,002,516	191,652	q11.21	40	100	Loss;Loss;Loss;Los	P1_C1;P1_C2;P1_C4;P3_(	Complete;Complete;(	KLHL22, MED15, BC03586		3	0
chr22:20,147,325-20,775,648	628,323	q11.21	40	84	AI;AI;AI;AI	P1_C4;P3_C1;P3_C2;P3_(	Complete;Complete;(	LOC284865, LI	hsa-mir-1286	24	1
chr22:20,128,060-20,760,417	632,357	q11.21	40	84	Loss;Loss;Loss;Los	P1_C1;P1_C2;P1_C4;P3_(	Complete;Complete;(	ZDHHC8, LOC	hsa-mir-1286	27	1
chr22:19,962,583-20,042,368	79,785	q11.21	40	76	Loss;Loss;Loss;Los	P1_C1;P1_C2;P1_C4;P3_(	Complete;Complete;(	ARVCF, MIR1	hsa-mir-185	3	1
chr22:19,506,798-19,958,199	451,401	q11.21	40	73	Loss;Loss;Loss;Los	P1_C1;P1_C2;P1_C4;P3_(	Complete;Complete;(	CDC45, CLDN5, LINC0089		14	0
chr22:19,135,021-19,397,885	262,864	q11.21	40	6	Loss;Loss;Loss;Los	P1_C1;P1_C2;P1_C4;P3_(	Complete;Complete;(	GSC2, LINC01311, SLC25A		5	0
chr22:18,914,012-19,036,931	122,919	q11.21	40	100	Loss;Loss;Loss;Los	P1_C1;P1_C2;P1_C4;P3_(	Complete;Complete;(	PRODH, DQ585141, DGCR		8	0
chr22:18,221,554-18,300,865	79,311	q11.21	40	100	Loss;Loss;Loss;Los	P1_C1;P1_C2;P1_C4;P3_(	Complete;Complete;(	BID, MIR3198-	hsa-mir-3198	4	1
chr22:18,083,812-18,182,111	98,299	q11.21	40	100	AI;AI;AI;AI	P1_C1;P1_C5;P3_C1;P3_(	Complete;Complete;(	ATP6V1E1, BCL2L13		2	0
chr22:18,034,793-18,182,111	147,318	q11.21	40	100	Loss;Loss;Loss;Los	P1_C1;P1_C2;P1_C4;P3_(	Complete;Complete;(	CECR2, SLC25A18, LOC10		5	0
chr22:17,866,593-18,019,423	152,830	11.1 - q11.2	40	32	Loss;Loss;Loss;Los	P1_C1;P1_C2;P1_C4;P3_(	Complete;Complete;(	CECR2		1	0
chr22:17,588,637-17,690,391	101,754	q11.1	40	30	Loss;Loss;Loss;Los	P1_C1;P1_C2;P1_C4;P3_(	Complete;Complete;(	IL17RA, CECR6, TMEM121		11	0
chr22:17,445,606-17,549,874	104,268	q11.1	40	100	AI;AI;AI;AI;AI;Los	P1_C1;P1_C2;P1_C4;P3_(	Complete;Complete;(	GAB4, CECR7		2	0

chr8:43,460,491-43,674,370	213,879	p11.1	30	100	Gain;Gain;Gain	P1_C1;P1_C3;P3_C1	Complete;Complete;Complete			0	0
chr22:50,873,998-50,893,408	19,410	q13.33	30	0	Loss;Loss;Loss	P1_C1;P1_C4;P1_C5	Complete;Complete;	PPP6R2, SBF1		2	0
chr22:47,190,688-47,223,714	33,026	q13.31	30	0	Loss;Loss;Loss	P1_C1;P1_C4;P6_C1	Complete;Complete;	TBC1D22A		1	0
chr22:39,505,448-39,527,598	22,150	q13.1	30	0	Loss;Loss;Loss	P1_C4;P1_C5;P6_C1	Complete;Complete;	CBX7		1	0
chr22:39,146,642-39,440,933	294,291	q13.1	30	48	AI;AI;AI	P1_C5;P3_C1;P3_C2	Complete;Complete;	SUN2, DNAL4, NPTXR, CB		12	0
chr22:21,346,602-21,384,424	37,822	q11.21	30	100	Loss;Loss;Loss	P1_C1;P1_C2;P1_C4	Complete;Complete;	LZTR1, THAP7, DQ574263,		7	0
chr22:17,435,507-17,442,570	7,063	q11.1	30	100	AI;AI;AI	P1_C2;P3_C1;P3_C3	Complete;Complete;Complete			0	0
chr22:16,975,655-17,315,373	339,718	q11.1	30	100	AI;AI;AI	P1_C2;P3_C1;P3_C3	Complete;Complete;	DQ571479, CCT8L2, TPTE		8	0
chr1:30,640,464-30,657,099	16,635	p35.2	30	100	Loss;Loss;Loss	P1_C3;P6_C1;P7_C1	Complete;Complete;Complete			0	0
chrX:740,019-1,402,609	662,590	p22.33	20	55	Gain;Gain	P6_C1;P7_C1	Complete;Complete	DQ576039, CSF2RA, CRLF		3	0
chrX:38,984,318-39,426,437	442,119	p11.4	20	0	Loss;Loss	P1_C4;P7_C1	Complete;Complete	LINC01281, LOC286442, LI		3	0
chrX:32,821,372-33,017,944	196,572	p21.1	20	8	AI;AI	P1_C4;P1_C5	Complete;Complete	DMD, JA783513, JA783507,		7	0
chrX:32,653,566-32,821,372	167,806	p21.1	20	9	Loss;Loss	P1_C4;P1_C5	Complete;Complete	DMD, MIR548	hsa-mir-548f	5	1
chrX:32,520,932-32,653,566	132,634	p21.1	20	10	AI;AI	P1_C4;P1_C5	Complete;Complete	DMD, JA783548, JA783867,		6	0
chrX:32,476,215-32,520,932	44,717	p21.1	20	91	Loss;Loss	P1_C4;P1_C5	Complete;Complete	DMD, JA783415, JA783560,		5	0
chrX:32,398,544-32,476,215	77,671	p21.1	20	1	AI;AI	P1_C4;P1_C5	Complete;Complete	DMD, JA202330, JA783579,		5	0
chrX:24,073,875-24,193,755	119,880	p22.11	20	100	Gain;Gain	P6_C1;P7_C1	Complete;Complete	EIF2S3, ZFX-AS1, ZFX		3	0
chrX:23,846,331-23,928,519	82,188	p22.11	20	33	Gain;Gain	P6_C1;P7_C1	Complete;Complete	RPL9, APOO, CXorf58		3	0
chrX:17,608,995-17,757,011	148,016	p22.13	20	0	Loss;Loss	P1_C4;P7_C1	Complete;Complete	NHS, SCML1		2	0
chrX:150,830,685-150,840,724	10,039	q28	20	0	Loss;Loss	P1_C4;P6_C1	Complete;Complete	PASD1		1	0
chrX:12,201,134-12,233,556	32,422	p22.2	20	0	Loss;Loss	P1_C4;P6_C1	Complete;Complete	FRMPD4		1	0
chrX:107,808,354-107,879,164	70,810	q22.3	20	0	Loss;Loss	P1_C3;P1_C4	Complete;Complete	COL4A5		1	0
chr4:7,435,824-7,437,211	1,387	p16.1	20	0	Loss;Loss	P6_C1;P7_C1	Complete;Complete	PSAPL1, SORCS2		2	0
chr4:39,604,400-39,838,356	233,956	p14	20	8	Gain;Gain	P6_C1;P7_C1	Complete;Complete	SMIM14, BC040333, UBE2		4	0
chr4:366,590-438,014	71,424	p16.3	20	100	Gain;Gain	P1_C3;P3_C1	Complete;Complete	ZNF141, ABCA11P, ZNF72		3	0
chr22:38,129,915-38,153,666	23,751	q13.1	20	100	AI;AI	P1_C5;P3_C2	Complete;Complete	TRIOBP		1	0
chr22:36,879,901-36,893,147	13,246	q12.3	20	100	AI;AI;AI;AI;AI;Los	P1_C4;P1_C5;P3_C1;P3_	Complete;Complete;	FOXRED2		1	0
chr2:179,613,414-179,615,962	2,548	q31.2	20	0	Loss;Loss	P6_C1;P7_C1	Complete;Complete	TTN		1	0
chr19:53,056,614-53,066,259	9,645	q13.41	20	100	Gain;Gain	P1_C1;P1_C3	Complete;Complete	ZNF808		1	0
chr19:52,537,268-52,538,142	874	q13.41	20	100	Gain;Gain	P1_C2;P1_C3	Complete;Complete	ZNF432		1	0
chr19:36,996,231-37,038,568	42,337	q13.12	20	0	Gain;Gain	P1_C3;P6_C1	Complete;Complete	ZNF260, ZNF529		2	0
chr19:36,857,232-36,945,488	88,256	q13.12	20	11	Gain;Gain	P1_C3;P6_C1	Complete;Complete	ZFP14, ZFP82, LOC644189,		4	0
chr19:27,862,694-28,135,850	273,156	q11	20	100	Gain;Gain	P1_C3;P1_C5	Complete;Complete	AK075337		1	0
chr19:21,716,320-21,720,496	4,176	p12	20	100	Gain;Gain	P1_C2;P1_C3	Complete;Complete	ZNF429		1	0
chr19:12,454,948-12,461,889	6,941	p13.2	20	100	Gain;Gain	P1_C3;P6_C1	Complete;Complete	ZNF442		1	0
chr19:12,186,566-12,192,228	5,662	p13.2	20	0	Gain;Gain	P1_C3;P6_C1	Complete;Complete	ZNF844		1	0
chr18:18,570,235-18,751,151	180,916	q11.1	20	3	Gain;Gain	P6_C1;P7_C1	Complete;Complete	ROCK1		1	0
chr14:35,307,619-35,344,875	37,256	q13.2	20	0	Gain;Gain	P6_C1;P7_C1	Complete;Complete	BAZ1A		1	0
chr12:52,961,642-52,967,401	5,759	q13.13	20	9	Loss;Loss	P6_C1;P7_C1	Complete;Complete	KRT74		1	0
chr1:7,869,964-8,049,903	179,939	p36.23	20	2	AI;AI	P1_C3;P6_C1	Complete;Complete	PER3, UTS2, TNFRSF9, TR		6	0
chr1:7,853,282-7,869,964	16,682	p36.23	20	0	Loss;Loss	P1_C3;P6_C1	Complete;Complete	PER3		1	0
chr1:6,636,869-7,853,282	1,216,413	6.31 - p36.	20	2	AI;AI	P1_C3;P6_C1	Complete;Complete	TAS1R1, ZBTB48, KLHL21		12	0
chr1:6,610,482-6,636,869	26,387	p36.31	20	22	Loss;Loss	P1_C3;P6_C1	Complete;Complete	NOL9, TAS1R1		2	0
chr1:5,958,005-6,610,482	652,477	p36.31	20	35	AI;AI	P1_C3;P6_C1	Complete;Complete	NPHP4, NPHP4	hsa-mir-425	20	1
chr1:5,949,942-5,958,005	8,063	p36.31	20	0	Loss;Loss	P1_C3;P6_C1	Complete;Complete	NPHP4, NPHP4		2	0
chr1:43,806,113-43,854,818	48,705	p34.2	20	0	AI;AI	P1_C3;P6_C1	Complete;Complete	MPL, CDC20, MIR6734, EL		5	0
chr1:43,608,641-43,785,044	176,403	p34.2	20	1	AI;AI	P1_C3;P6_C1	Complete;Complete	AK309744, FAM183A, DL4		10	0
chr1:40,981,271-41,347,650	366,379	p34.2	20	11	AI;AI	P1_C3;P6_C1	Complete;Complete	EXO5, ZNF684	hsa-mir-30e,	10	2
chr1:40,882,598-40,980,681	98,083	p34.2	20	12	AI;AI;Gain	P1_C3;P6_C1;P6_C1	Complete;Complete;	SMAP2, ZFP69B, ZFP69, EX		4	0
chr1:4,864,615-4,927,992	63,377	p36.32	20	1	Loss;Loss	P1_C3;P6_C1	Complete;Complete			0	0

chr1:4,597,360-4,685,447	88,087	p36.32	20	0	Loss;Loss	P1_C3;P6_C1	Complete;Complete	LINC01646		1	0
chr1:4,442,897-4,541,407	98,510	p36.32	20	0	Loss;Loss	P1_C3;P6_C1	Complete;Complete	LINC01777, LOC284661		2	0
chr1:4,207,376-5,949,942	1,742,566	6.32 - p36.	20	10	AI;AI	P1_C3;P6_C1	Complete;Complete	LINC01777, LOC284661, LI		10	0
chr1:39,884,102-40,539,413	655,311	34.3 - p34.	20	25	AI;AI	P1_C3;P6_C1	Complete;Complete	MACF1, BMP8A, OXCT2P1		23	0
chr1:39,097,607-39,876,129	778,522	p34.3	20	9	AI;AI	P1_C3;P6_C1	Complete;Complete	RRAGC, MYCBP, GJA9-MY		12	0
chr1:37,940,154-38,917,393	977,239	p34.3	20	3	AI;AI	P1_C3;P6_C1	Complete;Complete	ZC3H12A, MIR6732, MIR55		26	0
chr1:37,507,761-37,611,548	103,787	p34.3	20	1	Loss;Loss	P1_C3;P7_C1	Complete;Complete			0	0
chr1:36,625,781-37,890,108	1,264,327	p34.3	20	8	AI;AI	P1_C3;P6_C1	Complete;Complete	MAP7D1, THR	hsa-mir-4255	12	1
chr1:36,238,746-36,550,625	311,879	p34.3	20	2	AI;AI;Gain	P1_C3;P6_C1;P6_C1	Complete;Complete;(	AGO4, AGO1, AK025726, A		5	0
chr1:35,286,085-35,361,311	75,226	p34.3	20	1	AI;AI	P1_C3;P6_C1	Complete;Complete	SMIM12, DLGAP3		2	0
chr1:35,250,985-35,286,085	35,100	p34.3	20	35	Loss;Loss	P1_C3;P6_C1	Complete;Complete	GJB3, GJA4		2	0
chr1:34,837,557-35,052,807	215,250	p34.3	20	6	Loss;Loss	P1_C3;P6_C1	Complete;Complete			0	0
chr1:34,667,156-35,250,985	583,829	p34.3	20	7	AI;AI	P1_C3;P6_C1	Complete;Complete	C1orf94, MIR5	hsa-mir-552	6	1
chr1:34,657,642-34,667,156	9,514	p34.3	20	75	Loss;Loss	P1_C3;P6_C1	Complete;Complete	C1orf94		1	0
chr1:34,251,396-34,657,642	406,246	35.1 - p34.	20	1	AI;AI	P1_C3;P6_C1	Complete;Complete	CSMD2, HMGB4, CSMD2-A		5	0
chr1:34,177,832-34,251,396	73,564	p35.1	20	0	Loss;Loss	P1_C3;P6_C1	Complete;Complete	CSMD2		1	0
chr1:33,229,096-34,177,832	948,736	p35.1	20	12	AI;AI	P1_C3;P6_C1	Complete;Complete	KIAA1522, YARS, S100PBI		21	0
chr1:32,342,851-32,834,096	491,245	35.2 - p35.	20	5	AI;AI	P1_C3;P6_C1	Complete;Complete	PTP4A2, KHDRBS1, MIR55		19	0
chr1:32,256,409-32,342,851	86,442	p35.2	20	0	Loss;Loss	P1_C3;P6_C1	Complete;Complete	BC069257, SPOCD1		2	0
chr1:32,092,630-32,256,409	163,779	p35.2	20	0	AI;AI	P1_C3;P6_C1	Complete;Complete	HCRTR1, AX7	hsa-mir-4254	9	1
chr1:32,083,323-32,092,630	9,307	p35.2	20	0	Loss;Loss	P1_C3;P6_C1	Complete;Complete	HCRTR1		1	0
chr1:31,989,222-32,034,839	45,617	p35.2	20	11	Loss;Loss	P1_C3;P6_C1	Complete;Complete	LINC01226, LOC284551		2	0
chr1:30,657,099-32,034,839	1,377,740	p35.2	20	44	AI;AI	P1_C3;P6_C1	Complete;Complete	MATN1, MATN1-AS1, MIR		20	0
chr1:3,024,988-3,332,394	307,406	p36.32	20	27	Loss;Loss	P6_C1;P7_C1	Complete;Complete	MIR4251, PRD	hsa-mir-4251	2	1
chr1:29,573,696-30,640,464	1,066,768	35.3 - p35.	20	46	AI;AI	P1_C3;P6_C1	Complete;Complete	PTPRU, LINC01756, BC042		4	0
chr1:29,260,519-29,394,266	133,747	p35.3	20	4	Loss;Loss	P1_C3;P6_C1	Complete;Complete	EPB41		1	0
chr1:26,650,741-26,682,372	31,631	p36.11	20	4	Loss;Loss	P1_C3;P6_C1	Complete;Complete	AIM1L, CRYBG2		2	0
chr1:24,853,163-26,623,937	1,770,774	p36.11	20	29	AI;AI;Loss	P1_C3;P6_C1;P6_C1	Complete;Complete;(	RCAN3, LOC100506985, N(		44	0
chr1:16,727,256-16,763,653	36,397	p36.13	20	20	Loss;Loss	P1_C3;P6_C1	Complete;Complete	SPATA21		1	0
chr1:16,085,070-16,500,273	415,203	6.21 - p36.	20	45	AI;AI	P1_C3;P6_C1	Complete;Complete	FBLIM1, UQCRHL, FLJ374		13	0
chr1:16,069,568-16,085,070	15,502	p36.21	20	74	Loss;Loss	P1_C3;P6_C1	Complete;Complete	SLC25A34-AS1, TMEM82,		3	0
chr1:15,753,530-15,793,967	40,437	p36.21	20	30	Loss;Loss	P1_C3;P6_C1	Complete;Complete	EFHD2, CTRC, CELA2A		3	0
chr1:14,005,776-15,753,530	1,747,754	p36.21	20	6	AI;AI	P1_C3;P6_C1	Complete;Complete	PRDM2, AK124197, KAZN-		11	0
chr1:12,812,129-12,837,651	25,522	p36.21	20	100	Loss;Loss	P1_C3;P6_C1	Complete;Complete	C1orf158, PRAMEF12		2	0
chr1:12,779,573-12,786,171	6,598	p36.21	20	100	Loss;Loss	P1_C3;P6_C1	Complete;Complete	AADACL3		1	0
chr1:10,977,709-11,010,423	32,714	p36.22	20	100	Loss;Loss	P1_C3;P6_C1	Complete;Complete	C1orf127		1	0
chr1:10,511,101-10,523,645	12,544	p36.22	20	0	Loss;Loss	P1_C3;P6_C1	Complete;Complete	CENPS-CORT, APITD1-CO		4	0
chr1:1,535,600-4,121,665	2,586,065	6.33 - p36.	20	65	AI;AI	P1_C3;P6_C1	Complete;Complete	LOC10537858(	hsa-mir-4251	71	2
chrX:99,686,905-102,191,794	2,504,889	q22.1	10	12	AI;Loss	P1_C4;P1_C4	Complete;Complete	TNMD, TSPAN6, SRPX2, S		43	0
chrX:97,109,893-99,651,586	2,541,693	21.33 - q22	10	6	AI;Loss	P1_C4;P1_C4	Complete;Complete	Mir_340, XRCC6P5, PCDH1		3	0
chrX:9,941,005-10,087,293	146,288	p22.2	10	0	Loss	P1_C4	Complete	WWC3		1	0
chrX:9,862,984-9,865,363	2,379	p22.2	10	0	Loss	P1_C4	Complete	SHROOM2		1	0
chrX:9,814,533-9,862,984	48,451	p22.2	10	0	AI	P1_C4	Complete	SHROOM2		1	0
chrX:9,690,318-9,814,533	124,215	p22.2	10	1	Loss	P1_C4	Complete	GPR143, SHROOM2		2	0
chrX:9,548,930-9,640,322	91,392	p22.2	10	7	Loss	P1_C4	Complete	TBL1X		1	0
chrX:84,382,064-97,073,640	12,691,576	21.1 - q21.3	10	42	AI;Loss	P1_C4;P1_C4	Complete;Complete	LOC101928128	hsa-mir-1321	30	3
chrX:73,745,121-84,360,643	10,615,522	13.2 - q21.	10	6	AI;Loss	P1_C4;P1_C4	Complete;Complete	SLC16A2, RLII	hsa-mir-384,	48	4
chrX:71,564,631-73,569,177	2,004,546	13.1 - q13.	10	12	AI;Loss	P1_C4;P1_C4	Complete;Complete	HDAC8, PHKA	hsa-mir-421,	33	4
chrX:71,394,240-71,455,330	61,090	q13.1	10	12	Loss	P1_C4	Complete	ERCC6L, PIN4		2	0
chrX:71,338,015-71,394,240	56,225	q13.1	10	1	AI	P1_C4	Complete	NHSL2, RTL5, RGAG4, FLJ		4	0

chrX:70,839,840-71,338,015	498,175	q13.1	10	61	Loss	P1_C4	Complete	BCYRN1, LOC100129291, I		9	0
chrX:70,357,165-70,812,128	454,963	q13.1	10	100	Loss	P1_C4	Complete	MED12, NLGN3, GJB1, ZM		12	0
chrX:70,160,409-70,294,689	134,280	q13.1	10	100	AI;Loss	P1_C4;P1_C4	Complete;Complete	SNX12		1	0
chrX:69,498,038-70,145,724	647,686	q13.1	10	78	AI;Loss	P1_C4;P1_C4	Complete;Complete	ARR3, RAB41, PDZD11, KI		11	0
chrX:68,624,022-69,478,500	854,478	q13.1	10	0	AI;Loss	P1_C4;P1_C4	Complete;Complete	FAM155B, EDA, MIR676, A		10	0
chrX:68,382,804-68,524,265	141,461	q13.1	10	0	AI;Loss	P1_C4;P1_C4	Complete;Complete	PJA1, LINC00269		2	0
chrX:68,146,237-68,320,225	173,988	q13.1	10	47	Loss	P1_C4	Complete			0	0
chrX:67,966,690-68,146,237	179,547	q13.1	10	100	AI	P1_C4	Complete	EFNB1		1	0
chrX:63,445,950-67,966,690	4,520,740	11.2 - q13.	10	24	Loss	P1_C4	Complete	ASB12, MTMR	hsa-mir-223	18	1
chrX:61,835,206-63,409,452	1,574,246	11.1 - q11.	10	27	Loss	P1_C4	Complete	SPIN4, AK1302	hsa-mir-1468	8	1
chrX:55,247,953-58,409,750	3,161,797	11.21 - p11	10	28	Loss	P1_C4	Complete	PAGE5, PAGE3, LOC10042		21	0
chrX:55,039,684-55,185,606	145,922	p11.21	10	100	Loss	P1_C4	Complete	ALAS2, PAGE2B, PAGE2, I		4	0
chrX:53,624,056-54,956,804	1,332,748	1.22 - p11.	10	100	AI;Loss	P1_C4;P1_C4	Complete;Complete	HUWE1, PHF8, FAM120C,		13	0
chrX:49,114,392-53,057,918	3,943,526	1.23 - p11.	10	35	AI;Loss	P1_C4;P1_C4	Complete;Complete	FOXP3, FLICR	hsa-mir-532,	93	8
chrX:49,032,926-49,069,847	36,921	p11.23	10	100	Loss	P1_C4	Complete	PRICKLE3, SYP, SYP-AS1,		4	0
chrX:48,842,429-48,933,243	90,814	p11.23	10	100	Loss	P1_C4	Complete	GRIPAP1, TFE3, CCDC120,		5	0
chrX:48,460,318-48,655,131	194,813	p11.23	10	100	Loss	P1_C4	Complete	WDR13, WAS, SUV39H1, C		5	0
chrX:48,338,774-48,380,511	41,737	p11.23	10	100	Loss	P1_C4	Complete	FTSJ1, LOC101927635, POI		4	0
chrX:47,511,659-48,311,022	799,363	p11.23	10	68	Loss	P1_C4	Complete	UXT, UXT-AS1, LOC10013		20	0
chrX:47,084,260-48,311,022	1,226,762	p11.23	10	45	AI	P1_C4	Complete	CDK16, USP11, SNORA11C		32	0
chrX:47,084,260-47,425,413	341,153	p11.23	10	2	Loss	P1_C4	Complete	CDK16, USP11, SNORA11C		7	0
chrX:44,250,177-44,699,893	449,716	p11.3	10	21	Gain	P7_C1	Complete	FUNDC1		1	0
chrX:40,885,573-41,284,003	398,430	p11.4	10	42	Gain	P7_C1	Complete	USP9X, LINC02601, 5S_rRN		4	0
chrX:39,966,240-47,062,395	7,096,155	11.4 - p11.2	10	11	Loss	P1_C4	Complete	BCOR, LOC10	hsa-mir-221,	63	2
chrX:34,971,979-47,062,395	12,090,416	21.1 - p11.2	10	12	AI	P1_C4	Complete	MAGEB16, CX	hsa-mir-221,	92	2
chrX:3,541,301-9,486,472	5,945,171	2.33 - p22.	10	48	Loss	P1_C4	Complete	PRKX, PRKX-A	hsa-mir-651	31	1
chrX:3,325,312-9,486,472	6,161,160	2.33 - p22.	10	46	AI	P1_C4	Complete	SNORA48B, PI	hsa-mir-651	32	1
chrX:3,229,552-3,325,312	95,760	p22.33	10	19	Loss	P1_C4	Complete	MXRA5		1	0
chrX:299,022-1,411,880	1,112,858	p22.33	10	37	AI;Loss	P1_C4;P1_C4	Complete;Complete	PPP2R3B, SHOX, DQ57603		5	0
chrX:22,578,824-24,329,852	1,751,028	p22.11	10	18	AI;Loss	P1_C4;P1_C4	Complete;Complete	PTCHD1-AS, LOC10087306		17	0
chrX:22,292,557-22,554,841	262,284	p22.11	10	8	Loss	P1_C4	Complete	ZNF645, CBLL2, PTCHD1-A		4	0
chrX:2,936,648-3,216,495	279,847	p22.33	10	10	AI;Loss	P1_C4;P1_C4	Complete;Complete	ARSH, ARSF, LINC01546, C		4	0
chrX:2,727,632-2,799,215	71,583	p22.33	10	100	Loss	P1_C4	Complete	XG, GYG2		2	0
chrX:2,576,718-2,695,521	118,803	p22.33	10	1	Loss	P1_C4	Complete	CD99, XGY2, XG		3	0
chrX:18,996,079-22,291,205	3,295,126	2.13 - p22.	10	22	AI;Loss	P1_C4;P1_C4	Complete;Complete	PHKA2, GPR64	hsa-mir-1308	29	1
chrX:153,940,179-155,270,560	1,330,381	q28	10	96	AI;Loss	P1_C4;P1_C4	Complete;Complete	GAB3, SNORA	hsa-mir-1184	38	3
chrX:153,688,233-153,692,697	4,464	q28	10	100	Loss	P1_C4	Complete	PLXNA3		1	0
chrX:153,414,892-153,688,233	273,341	q28	10	100	AI	P1_C4	Complete	OPN1LW, TEX28, OPN1MV		23	0
chrX:153,297,256-153,414,892	117,636	q28	10	100	Loss	P1_C4	Complete	MECP2, OPN1LW		2	0
chrX:153,247,994-153,291,683	43,689	q28	10	100	Loss	P1_C4	Complete	TMEM187, IRA	hsa-mir-718	4	1
chrX:152,469,090-153,247,994	778,904	q28	10	100	AI	P1_C4	Complete	MAGEA1, PNN	hsa-mir-3202	36	2
chrX:152,469,090-152,710,671	241,581	q28	10	100	Loss	P1_C4	Complete	MAGEA1, PNMA6F, ZNF27		9	0
chrX:152,144,701-152,226,564	81,863	q28	10	69	Loss	P1_C4	Complete	PNMA5, PNMA3		2	0
chrX:151,131,234-152,103,004	971,770	q28	10	53	AI;Loss	P1_C4;P1_C4	Complete;Complete	GABRE, MAGI	hsa-mir-105-	26	3
chrX:151,022,381-151,120,778	98,397	q28	10	10	Loss	P1_C4	Complete	MAGEA4-AS1, MAGEA4		2	0
chrX:150,350,327-150,908,733	558,406	q28	10	14	AI	P1_C4	Complete	VMA21, LOC105377213, PA		6	0
chrX:14,040,257-18,909,656	4,869,399	22.2 - p22.1	10	7	AI	P1_C4	Complete	GEMIN8, UBE2E4P, GLRA		50	0
chrX:136,117,688-150,334,531	14,216,843	q26.3 - q28	10	19	AI;Loss	P1_C4;P1_C4	Complete;Complete	ZIC3, LINC008	hsa-mir-504,	99	25
chrX:135,095,266-136,112,574	1,017,308	q26.3	10	8	AI;Loss	P1_C4;P1_C4	Complete;Complete	SLC9A6, FHL1	hsa-mir-934	16	1
chrX:13,235,668-13,983,814	748,146	p22.2	10	1	Loss	P1_C4	Complete	LINC02154, GS1-600G8.3, I		14	0

chrX:129,220,677-135,047,043	5,826,366	26.1 - q26.	10	11	AI;Loss	P1_C4;P1_C4	Complete;Complete	ELF4, AIFM1,	hsa-mir-363,	98	12
chrX:128,920,376-129,061,911	141,535	q26.1	10	33	Loss	P1_C4	Complete	SASH3, ZDHHC9, UTP14A		3	0
chrX:125,371,689-128,893,613	3,521,924	q25 - q26.1	10	41	AI;Loss	P1_C4;P1_C4	Complete;Complete	DCAF12L1, PRR32, CXorf6		9	0
chrX:123,004,158-123,269,043	264,885	q25	10	0	Gain	P7_C1	Complete	XIAP, LOC101928402, STA		3	0
chrX:119,732,294-125,297,952	5,565,658	q24 - q25	10	18	AI;Loss	P1_C4;P1_C4	Complete;Complete	MCTS1, C1GA	hsa-mir-220a	37	1
chrX:119,509,834-119,703,279	193,445	q24	10	0	Loss	P1_C4	Complete	ATP1B4, LAMP2, CUL4B		3	0
chrX:119,006,479-119,500,087	493,608	q24	10	8	Loss	P1_C4	Complete	NDUFA1, AKAP14, NKAP,		15	0
chrX:114,444,931-119,500,087	5,055,156	q23 - q24	10	37	AI	P1_C4	Complete	LRCH2, LUZP	hsa-mir-1277	56	2
chrX:114,444,931-118,817,505	4,372,574	q23 - q24	10	41	Loss	P1_C4	Complete	LRCH2, LUZP	hsa-mir-1277	36	2
chrX:111,326,811-114,419,335	3,092,524	q23	10	60	AI;Loss	P1_C4;P1_C4	Complete;Complete	RTL4, ZCCHC	hsa-mir-4329	19	7
chrX:109,697,785-111,200,966	1,503,181	q23	10	1	Loss	P1_C4	Complete	RGAG1, RTL9, TDGF1P3, C		14	0
chrX:107,979,298-109,694,148	1,714,850	q22.3 - q23	10	5	AI;Loss	P1_C4;P1_C4	Complete;Complete	IRS4, LOC1019	hsa-mir-652	17	1
chrX:104,463,817-107,977,755	3,513,938	q22.3	10	8	AI	P1_C4	Complete	IL1RAPL2, TEX13A, NRK,		33	0
chrX:103,318,445-104,454,509	1,136,064	22.2 - q22.	10	12	AI;Loss	P1_C4;P1_C4	Complete;Complete	ZCCHC18, LOC286437, SL		6	0
chrX:102,979,511-103,077,802	98,291	q22.2	10	14	Loss	P1_C4	Complete	GLRA4, PLP1, RAB9B		3	0
chrX:102,195,390-102,966,557	771,167	22.1 - q22.	10	7	AI;Loss	P1_C4;P1_C4	Complete;Complete	LINC00630, BEX1, NXF3, E		21	0
chrX:10,176,906-13,983,814	3,806,908	p22.2	10	17	AI	P1_C4	Complete	CLCN4, U6, MID1, Mir_652		36	0
chrX:10,105,769-10,176,906	71,137	p22.2	10	0	Loss	P1_C4	Complete	WWC3, CLCN4		2	0
chrX:1,482,799-2,472,499	989,700	p22.33	10	19	AI;Loss	P1_C4;P1_C4	Complete;Complete	IL3RA, CRLF2, SLC25A6, I		11	0
chrX:1,472,307-1,499,802	27,495	p22.33	10	100	Gain	P7_C1	Complete	IL3RA, CRLF2		2	0
chrX:1,427,577-1,463,086	35,509	p22.33	10	100	Loss	P1_C4	Complete	CSF2RA, IL3RA, CRLF2		3	0
chr9:99,521,121-99,581,466	60,345	q22.33	10	80	Gain	P1_C3	Complete	ZNF510, ZNF782		2	0
chr9:95,594,959-95,610,711	15,752	q22.31	10	0	Gain	P1_C3	Complete	ANKRD19P, ZNF484		2	0
chr9:65,629,772-69,238,239	3,608,467	12 - q21.1	10	100	Gain	P1_C3	Complete	DQ600787, AK	hsa-mir-1299	39	1
chr9:17,576,554-17,849,639	273,085	p22.2	10	22	AI	P6_C1	Complete	SH3GL2		1	0
chr9:137,777,585-137,809,972	32,387	q34.3	10	100	Loss	P7_C1	Complete	FCN2, FCN1		2	0
chr9:137,656,205-137,707,833	51,628	q34.3	10	100	Loss	P7_C1	Complete	COL5A1		1	0
chr9:122,763,700-122,904,242	140,542	q33.2	10	38	Loss	P6_C1	Complete			0	0
chr9:122,278,536-122,391,755	113,219	q33.1	10	12	Loss	P6_C1	Complete			0	0
chr9:116,854,255-116,871,425	17,170	q32	10	75	Loss	P7_C1	Complete	KIF12		1	0
chr9:104,152,584-104,184,097	31,513	q31.1	10	1	Gain	P1_C3	Complete	MRPL50, ZNF189, ALDOB		3	0
chr8:72,955,983-72,964,123	8,140	q13.3	10	100	Loss	P1_C3	Complete	LOC100132891, MSC-AS1,		3	0
chr8:69,349,964-69,503,478	153,514	q13.2	10	1	Gain	P1_C3	Complete	C8orf34		1	0
chr8:617,755-647,007	29,252	p23.3	10	100	Loss	P7_C1	Complete	ERICH1		1	0
chr8:53,832,888-53,948,445	115,557	q11.23	10	100	AI	P7_C1	Complete	NPBWR1		1	0
chr8:5,532,431-5,533,822	1,391	p23.2	10	100	Loss	P1_C3	Complete			0	0
chr8:421,067-462,460	41,393	p23.3	10	100	Loss	P7_C1	Complete	AK056623, TDRP		2	0
chr8:39,445,543-39,535,440	89,897	p11.22	10	100	Loss	P1_C3	Complete	ADAM18		1	0
chr8:143,662,172-143,746,406	84,234	q24.3	10	82	Loss	P7_C1	Complete	ARC, LOC101928087, JH8,		4	0
chr8:142,836,252-143,192,639	356,387	q24.3	10	52	Loss	P7_C1	Complete	MIR1302-7, MI	hsa-mir-1302	2	1
chr8:142,446,922-142,455,808	8,886	q24.3	10	100	Loss	P7_C1	Complete	MROH5		1	0
chr8:133,898,916-133,906,154	7,238	q24.22	10	0	Loss	P7_C1	Complete	TG		1	0
chr8:125,988,984-125,989,909	925	q24.13	10	0	Gain	P1_C3	Complete	ZNF572		1	0
chr8:12,877,594-12,879,237	1,643	p22	10	0	Loss	P1_C3	Complete	TRMT9B, KIAA1456		2	0
chr8:110,421,287-110,442,154	20,867	q23.1	10	0	Loss	P1_C3	Complete	PKHD1L1		1	0
chr8:105,071,716-105,213,705	141,989	q22.3	10	0	Loss	P1_C3	Complete	RIMS2		1	0
chr8:0-256,689	256,689	p23.3	10	96	Loss	P7_C1	Complete	OR4F21, RPL23AP53, ZNF		3	0
chr8:0-197,812	197,812	p23.3	10	95	Gain	P1_C3	Complete	OR4F21, RPL23AP53, ZNF		3	0
chr7:92,731,789-92,736,577	4,788	q21.2	10	0	Gain	P1_C3	Complete	SAMD9		1	0
chr7:91,622,253-91,631,303	9,050	q21.2	10	0	Gain	P1_C3	Complete	AKAP9		1	0

chr7:5,101,081-5,107,231	6,150	p22.1	10	0	Gain	P1_C3	Complete	RBAK-RBAKDN, RBAK		2	0
chr7:16,408,694-16,442,855	34,161	p21.2	10	100	Gain	P1_C3	Complete	ISPD		1	0
chr7:157,554,106-157,759,935	205,829	q36.3	10	100	Loss	P7_C1	Complete	LOC100506585, PTPRN2		2	0
chr7:15,608,900-15,699,426	90,526	p21.2	10	8	Gain	P1_C3	Complete	MEOX2		1	0
chr7:117,149,145-117,177,746	28,601	q31.2	10	0	Loss	P1_C3	Complete	CFTR		1	0
chr7:106,649,484-106,666,904	17,420	q22.3	10	0	Gain	P1_C3	Complete			0	0
chr6:97,618,872-97,679,377	60,505	q16.1	10	0	Loss	P1_C3	Complete	MMS22L, MIR548H3		2	0
chr6:87,966,423-87,970,087	3,664	q14.3	10	0	Gain	P1_C3	Complete	ZNF292		1	0
chr6:74,193,675-74,261,920	68,245	q13	10	4	Gain	P7_C1	Complete	MTO1, SNORD141A, SNOR		6	0
chr6:61,000,000-62,229,529	1,229,529	q11.1	10	28	Gain	P6_C1	Complete			0	0
chr6:46,657,807-46,659,906	2,099	p12.3	10	0	Loss	P1_C3	Complete	TDRD6		1	0
chr6:46,135,313-46,139,211	3,898	p21.1	10	0	Loss	P1_C3	Complete	ENPP5		1	0
chr6:40,635,045-40,765,578	130,533	p21.1	10	3	Loss	P7_C1	Complete			0	0
chr6:40,126,876-40,347,588	220,712	p21.2	10	2	Loss	P7_C1	Complete	LINC00951, DQ571907, TD		3	0
chr6:27,416,385-27,426,363	9,978	p22.1	10	0	Gain	P1_C3	Complete	ZNF184		1	0
chr6:169,634,881-169,648,338	13,457	q27	10	35	Loss	P7_C1	Complete	LOC101929523, THBS2		2	0
chr6:169,334,689-169,438,747	104,058	q27	10	1	Loss	P7_C1	Complete	LOC105378146, LOC10192		2	0
chr6:168,746,922-168,973,885	226,963	q27	10	47	Loss	P7_C1	Complete	SMOC2		1	0
chr6:146,260,717-146,276,271	15,554	q24.3	10	0	Gain	P1_C3	Complete	SHPRH		1	0
chr6:117,686,547-117,705,835	19,288	q22.1	10	0	Loss	P1_C3	Complete	ROS1, GOPC		2	0
chr5:68,664,058-68,709,767	45,709	q13.2	10	100	Gain	P7_C1	Complete	AK6, TAF9, RAD17		3	0
chr5:624,589-640,672	16,083	p15.33	10	100	Loss	P7_C1	Complete	CEP72		1	0
chr5:45,992,925-46,399,093	406,168	p12 - p11	10	100	Gain	P1_C3	Complete			0	0
chr5:2,663,987-2,749,729	85,742	p15.33	10	27	Loss	P7_C1	Complete	LSINCT5, IRX2		2	0
chr5:2,001,955-2,107,805	105,850	p15.33	10	34	Loss	P7_C1	Complete			0	0
chr5:178,358,292-178,361,655	3,363	q35.3	10	0	Gain	P1_C3	Complete	ZFP2		1	0
chr5:178,306,858-178,311,255	4,397	q35.3	10	0	Gain	P1_C3	Complete	ZNF354B		1	0
chr5:135,223,765-135,284,779	61,014	q31.1	10	0	Loss	P7_C1	Complete	SLC25A48, IL9, FBXL21P,		5	0
chr5:134,610,869-134,830,296	219,427	q31.1	10	1	Loss	P7_C1	Complete	C5orf66, LOC100996485, A		7	0
chr5:13,913,886-13,916,913	3,027	p15.2	10	100	Loss	P1_C3	Complete	DNAH5		1	0
chr5:0-286,460	286,460	p15.33	10	97	Loss	P7_C1	Complete	PLEKHG4B, LRRC14B, CC		6	0
chr4:71,024,361-71,043,230	18,869	q13.3	10	100	Gain	P3_C3	Complete	C4orf40, PRR27		2	0
chr4:126,390,837-126,402,865	12,028	q28.1	10	0	Gain	P1_C3	Complete	FAT4		1	0
chr4:10,027,555-10,339,123	311,568	p16.1	10	92	Loss	P7_C1	Complete	SLC2A9, MIR3	hsa-mir-3138	12	1
chr4:0-191,453	191,453	p16.3	10	95	Gain	P1_C3	Complete	ZNF595, ZNF718		2	0
chr3:93,537,290-93,652,298	115,008	q11.1	10	71	Gain	P7_C1	Complete	PROS1		1	0
chr3:88,103,767-88,190,781	87,014	p11.1	10	0	Gain	P1_C3	Complete	CGGBP1, ZNF654		2	0
chr3:77,441,998-77,500,555	58,557	p12.3	10	100	Loss	P1_C3	Complete	ROBO2		1	0
chr3:77,150,133-77,325,579	175,446	p12.3	10	2	Loss	P1_C3	Complete	ROBO2		1	0
chr3:76,932,116-77,074,810	142,694	p12.3	10	1	Loss	P1_C3	Complete	ROBO2		1	0
chr3:76,438,793-76,482,223	43,430	p12.3	10	100	Loss	P1_C3	Complete	ROBO2		1	0
chr3:44,759,622-44,776,382	16,760	p21.31	10	0	Gain	P1_C3	Complete	ZNF502, ZNF501		2	0
chr3:37,365,171-37,368,112	2,941	p22.2	10	0	Gain	P1_C3	Complete	GOLGA4		1	0
chr3:29,892,053-29,932,801	40,748	p24.1	10	0	Gain	P1_C3	Complete	RBMS3, Mir_544		2	0
chr3:22,202,592-22,210,987	8,395	p24.3	10	100	Loss	P1_C3	Complete	ZNF385D		1	0
chr3:167,051,224-167,069,002	17,778	q26.1	10	0	Loss	P1_C3	Complete	ZBBX		1	0
chr3:167,018,528-167,028,639	10,111	q26.1	10	0	Loss	P1_C3	Complete	ZBBX		1	0
chr3:139,785,476-140,120,148	334,672	q23	10	57	Loss	P7_C1	Complete	CLSTN2		1	0
chr3:126,868,645-126,972,634	103,989	q21.3	10	2	Loss	P7_C1	Complete	C3orf56		1	0
chr3:125,851,664-125,882,316	30,652	q21.3	10	0	Loss	P7_C1	Complete	ALDH1L1		1	0

chr3:121,414,271-121,417,185	2,914	q13.33	10	0	Gain	P1_C3	Complete	GOLGB1		1	0
chr3:10,799,126-10,993,508	194,382	p25.3	10	100	Loss	P7_C1	Complete	LINC00606, SLC6A11		2	0
chr22:20,909,265-21,025,306	116,041	q11.21	10	100	Gain	P1_C5	Complete	MED15, BC035867		2	0
chr22:20,269,409-20,763,446	494,037	q11.21	10	80	Gain	P1_C5	Complete	KIAA1653, DGCR6L, LOC7		20	0
chr22:20,098,732-20,130,397	31,665	q11.21	10	100	Gain	P1_C5	Complete	DGCR8, TRMT2A, MIR681		6	0
chr22:19,966,908-20,007,681	40,773	q11.21	10	54	Gain	P1_C5	Complete	ARVCF, TANGO2		2	0
chr22:19,950,153-19,958,199	8,046	q11.21	10	97	Gain	P1_C5	Complete	COMT, MIR4761, ARVCF		3	0
chr22:19,729,281-19,808,798	79,517	q11.21	10	96	Gain	P1_C5	Complete	TBX1, GNB1L		2	0
chr22:19,590,918-19,678,745	87,827	q11.21	10	100	Gain	P1_C5	Complete			0	0
chr22:19,227,805-19,310,253	82,448	q11.21	10	20	Gain	P1_C5	Complete	CLTCL1		1	0
chr22:18,210,513-18,246,153	35,640	q11.21	10	100	Gain	P1_C5	Complete	BCL2L13, DQ570096, BID		3	0
chr22:17,862,207-17,946,185	83,978	11.1 - q11.2	10	1	Gain	P1_C5	Complete	CECR2		1	0
chr22:17,211,364-17,444,653	233,289	q11.1	10	100	Gain	P1_C5	Complete	BC038197, LINC01665, XK		6	0
chr20:59,979,125-60,297,508	318,383	q13.33	10	39	Loss	P7_C1	Complete	CDH4, AK097866, LOC100		3	0
chr20:58,348,452-58,490,548	142,096	3.32 - q13.	10	50	Loss	P7_C1	Complete	PHACTR3, SYCP2		2	0
chr20:54,041,419-54,158,579	117,160	q13.2	10	0	Loss	P6_C1	Complete	LINC01441, LINC01440		2	0
chr20:45,126,594-45,137,134	10,540	q13.12	10	0	Gain	P1_C3	Complete	ZNF334		1	0
chr20:40,687,854-40,701,384	13,530	q12	10	100	Loss	P7_C1	Complete			0	0
chr20:24,952,121-24,995,906	43,785	p11.21	10	2	Loss	P7_C1	Complete	APMAP, ACSS1		2	0
chr20:24,117,232-24,446,751	329,519	p11.21	10	6	Loss	P7_C1	Complete	FLJ33581, LINC01721		2	0
chr20:23,643,422-23,743,152	99,730	p11.21	10	100	Loss	P7_C1	Complete	CST4, CST1		2	0
chr2:73,676,039-73,678,971	2,932	p13.1	10	0	Gain	P1_C3	Complete	ALMS1		1	0
chr2:71,839,801-71,912,130	72,329	p13.2	10	0	Loss	P7_C1	Complete	DYSF		1	0
chr2:455,271-1,376,873	921,602	p25.3	10	50	Loss	P7_C1	Complete	LINC01874, LINC01875, LC		9	0
chr2:241,398,552-241,509,978	111,426	q37.3	10	17	Loss	P7_C1	Complete	GPC1, ANKMY1, DUSP28,		4	0
chr2:239,941,660-240,095,935	154,275	q37.3	10	11	Loss	P7_C1	Complete	MIR4440, MIR4441, HDAC4		3	0
chr2:238,284,545-238,307,688	23,143	q37.3	10	0	Loss	P7_C1	Complete	COL6A3		1	0
chr2:238,239,548-238,253,882	14,334	q37.3	10	0	Loss	P7_C1	Complete	COL6A3		1	0
chr2:203,431,235-203,632,877	201,642	q33.2	10	4	Gain	P7_C1	Complete	BMPR2, FAM117B		2	0
chr2:2,472,754-2,520,606	47,852	p25.3	10	3	Loss	P7_C1	Complete			0	0
chr2:196,866,165-196,889,757	23,592	q32.3	10	0	Loss	P1_C3	Complete	DNAH7		1	0
chr2:186,672,055-186,673,716	1,661	q32.1	10	100	Loss	P1_C3	Complete	FSIP2		1	0
chr2:179,498,140-179,539,809	41,669	q31.2	10	29	Loss	P6_C1	Complete	MIR548N, TTN, TTN, TTN		4	0
chr2:179,456,516-179,466,174	9,658	q31.2	10	0	Loss	P7_C1	Complete	MIR548N, TTN-AS1, TTN		3	0
chr2:170,487,854-170,497,220	9,366	q31.1	10	10	Gain	P1_C3	Complete	PPIG		1	0
chr2:119,696,304-119,751,928	55,624	q14.2	10	0	Loss	P7_C1	Complete	MARCO		1	0
chr2:1,664,674-1,729,667	64,993	p25.3	10	9	Loss	P7_C1	Complete	PXDN		1	0
chr2:1,499,788-1,501,643	1,855	p25.3	10	100	Loss	P7_C1	Complete	TPO		1	0
chr2:0-280,262	280,262	p25.3	10	86	Loss	P7_C1	Complete	FAM110C, SH3YL1, ACP1,		5	0
chr19:9,865,817-9,869,987	4,170	p13.2	10	100	Gain	P1_C3	Complete	ZNF846		1	0
chr19:8,909,362-8,922,581	13,219	p13.2	10	100	Gain	P1_C3	Complete	ZNF558		1	0
chr19:57,065,635-57,089,526	23,891	q13.43	10	36	Gain	P1_C3	Complete	ZFP28, BX647249, ZNF470		3	0
chr19:57,036,336-57,047,528	11,192	q13.43	10	0	Gain	P1_C3	Complete	ZNF471		1	0
chr19:56,880,280-56,954,002	73,722	q13.43	10	0	Gain	P1_C3	Complete	ZNF542, ZNF542P, ZNF582		6	0
chr19:53,953,792-53,969,482	15,690	q13.42	10	100	Gain	P1_C3	Complete	ZNF765-ZNF761, ZNF761		2	0
chr19:53,740,245-53,741,421	1,176	q13.42	10	100	Gain	P1_C3	Complete	ZNF677		1	0
chr19:53,643,102-53,669,096	25,994	q13.42	10	100	Gain	P1_C3	Complete	ZNF347, ZNF665		2	0
chr19:53,571,086-53,573,260	2,174	q13.41	10	100	Gain	P1_C3	Complete	ZNF160		1	0
chr19:53,301,803-53,344,911	43,108	q13.41	10	100	Gain	P1_C3	Complete	ZNF28, ZNF468		2	0
chr19:53,116,195-53,116,982	787	q13.41	10	100	Gain	P1_C3	Complete	ZNF83		1	0

chr19:52,868,973-52,889,869	20,896	q13.41	10	19	Gain	P1_C3	Complete	ZNF610, ZNF880, AF08616		3	0
chr19:52,824,893-52,839,988	15,095	q13.41	10	0	Gain	P1_C3	Complete	ZNF480, AK097759, ZNF61		3	0
chr19:52,609,348-52,623,143	13,795	q13.41	10	100	Gain	P1_C3	Complete	ZNF616		1	0
chr19:52,496,293-52,499,542	3,249	q13.41	10	100	Gain	P1_C3	Complete	AK128361, ZNF615		2	0
chr19:44,978,870-44,982,169	3,299	q13.31	10	100	Gain	P1_C3	Complete	ZNF180		1	0
chr19:44,790,306-44,833,575	43,269	q13.31	10	2	Gain	P1_C3	Complete	ZNF235, ZNF112, ZFP112		3	0
chr19:44,680,062-44,740,684	60,622	q13.31	10	0	Gain	P1_C3	Complete	ZNF226, ZNF227, ZNF235		3	0
chr19:44,660,578-44,662,056	1,478	q13.31	10	0	Gain	P1_C3	Complete	ZNF234		1	0
chr19:44,599,622-44,612,377	12,755	q13.31	10	14	Gain	P1_C3	Complete	ZNF224, LOC100379224		2	0
chr19:44,469,699-44,485,636	15,937	q13.31	10	2	Gain	P1_C3	Complete	ZNF221		1	0
chr19:44,345,900-44,418,841	72,941	q13.31	10	0	Gain	P1_C3	Complete	ZNF283, ZNF404, LOC1005		4	0
chr19:40,517,839-40,588,941	71,102	q13.2	10	5	Gain	P1_C3	Complete	ZNF546, ZNF780B, ZNF780		3	0
chr19:38,123,246-38,164,432	41,186	q13.12	10	0	Gain	P1_C3	Complete	ZFP30, ZNF781		2	0
chr19:38,055,551-38,056,962	1,411	q13.12	10	0	Gain	P1_C3	Complete	ZNF571-AS1, ZNF571, ZNF		3	0
chr19:37,877,473-37,905,144	27,671	q13.12	10	0	Gain	P1_C3	Complete	ZNF527, ZNF569		2	0
chr19:37,618,114-37,677,948	59,834	q13.12	10	0	Gain	P1_C3	Complete	ZNF420, ZNF585A, ZNF585		3	0
chr19:37,179,322-37,441,930	262,608	q13.12	10	23	Gain	P1_C3	Complete	ZNF567, ZNF850, AX74737		10	0
chr19:35,434,480-35,435,611	1,131	q13.11	10	0	Gain	P1_C3	Complete	ZNF30		1	0
chr19:24,302,116-24,518,609	216,493	p12 - p11	10	100	Gain	P1_C3	Complete	ZNF254, HAVCR1P1		2	0
chr19:21,989,927-21,992,560	2,633	p12	10	100	Gain	P1_C3	Complete	ZNF43		1	0
chr19:21,055,220-21,141,796	86,576	p12	10	80	Gain	P1_C3	Complete	ZNF85		1	0
chr19:20,113,887-20,120,656	6,769	p12	10	100	Gain	P1_C3	Complete	ZNF682		1	0
chr19:19,992,095-20,050,462	58,367	13.11 - p1	10	100	Gain	P1_C3	Complete	ZNF253, ZNF93		2	0
chr19:19,820,946-19,823,562	2,616	p13.11	10	0	Gain	P1_C3	Complete	ZNF14		1	0
chr19:12,297,183-12,385,214	88,031	p13.2	10	24	Gain	P1_C3	Complete	ZNF136, AX721123, LOC10		5	0
chr19:11,942,914-12,063,197	120,283	p13.2	10	100	Gain	P1_C3	Complete	ZNF440, AX747599, ZNF43		6	0
chr19:11,886,910-11,917,904	30,994	p13.2	10	26	Gain	P1_C3	Complete	ZNF441, ZNF491		2	0
chr18:9,254,426-9,257,868	3,442	p11.22	10	0	Gain	P1_C3	Complete	ANKRD12		1	0
chr17:80,543,825-80,544,460	635	q25.3	10	100	Gain	P1_C3	Complete	FOXK2		1	0
chr17:60,232,717-60,435,430	202,713	q23.2	10	69	Gain	P7_C1	Complete	Mir_652, TBC1D3P2		2	0
chr17:58,522,657-58,596,702	74,045	q23.2	10	3	Gain	P7_C1	Complete	APPBP2		1	0
chr17:57,785,690-57,856,830	71,140	q23.1	10	1	Gain	P6_C1	Complete	VMP1		1	0
chr17:57,109,494-57,268,231	158,737	q22	10	15	Gain	P7_C1	Complete	TRIM37, BC01	hsa-mir-454,	6	2
chr17:45,352,153-45,462,701	110,548	q21.32	10	0	Loss	P6_C1	Complete	ITGB3, THCAT158, AX748		4	0
chr17:43,472,805-45,234,631	1,761,826	1.31 - q21.	10	100	Loss	P6_C1	Complete	AX747793, AR	hsa-mir-431	49	1
chr17:42,877,811-43,373,464	495,653	q21.31	10	3	Loss	P6_C1	Complete	GJC1, HIGD1B, EFTUD2, C		25	0
chr17:42,643,635-42,841,449	197,814	q21.31	10	7	Loss	P6_C1	Complete	LINC01180, MEIOC, C17or		6	0
chr17:42,483,189-42,643,635	160,446	q21.31	10	0	Gain	P6_C1	Complete	GPATCH8, FZD2		2	0
chr17:41,170,205-42,483,189	1,312,984	q21.31	10	10	AI	P6_C1	Complete	VAT1, RND2,	hsa-mir-2117	55	1
chr17:41,170,205-41,569,311	399,106	q21.31	10	26	Gain	P6_C1	Complete	VAT1, RND2,	hsa-mir-2117	17	1
chr17:40,865,367-40,931,054	65,687	21.2 - q21.3	10	8	Gain	P6_C1	Complete	EZH1, BC047651, RAMP2-A		5	0
chr17:40,694,197-40,716,961	22,764	q21.2	10	30	Gain	P6_C1	Complete	NAGLU, BC043620, HSD17		5	0
chr17:40,572,713-40,610,086	37,373	q21.2	10	0	Gain	P6_C1	Complete	PTRF, CAVIN1		2	0
chr17:39,623,397-40,572,713	949,316	q21.2	10	36	AI	P6_C1	Complete	KRT32, KRT35, KRT36, KR		48	0
chr17:39,059,866-39,593,520	533,654	q21.2	10	75	AI	P6_C1	Complete	KRT23, KRT39, KRT40, KR		44	0
chr17:38,483,714-39,059,866	576,152	q21.2	10	2	Loss	P6_C1	Complete	RARA-AS1, RARA, GJD3,		18	0
chr17:38,398,281-38,483,714	85,433	21.1 - q21.	10	0	Gain	P6_C1	Complete	WIPF2, CDC6, RARA		3	0
chr17:38,254,073-38,303,951	49,878	q21.1	10	6	Gain	P6_C1	Complete	NR1D1, TRNA_SeC, MSL1,		5	0
chr17:37,672,348-38,254,073	581,725	q12 - q21.1	10	10	AI	P6_C1	Complete	CDK12, NEUROD2, PPP1R		25	0
chr17:37,570,588-37,672,348	101,760	q12	10	0	Gain	P6_C1	Complete	MED1, CDK12		2	0

chr17:37,342,709-37,557,267	214,558	q12	10	3	AI;Gain	P6_C1;P6_C1	Complete;Complete	CACNB1, RPL19, STAC2, F		4	0
chr17:37,297,287-37,319,084	21,797	q12	10	18	Gain	P6_C1	Complete	PLXDC1, ARL5C		2	0
chr17:37,066,637-37,223,923	157,286	q12	10	15	Gain	P6_C1	Complete	LASP1, MIR6779, LINC006		9	0
chr17:36,179,875-37,223,923	1,044,048	q12	10	59	AI	P6_C1	Complete	YWHAEP7, LOC284100, DC		51	0
chr17:36,137,066-36,179,875	42,809	q12	10	100	Gain	P6_C1	Complete			0	0
chr17:35,917,877-36,137,066	219,189	q12	10	32	AI	P6_C1	Complete	SYNRG, MIR378J, DDX52,		4	0
chr17:35,917,877-36,031,415	113,538	q12	10	0	Gain	P6_C1	Complete	SYNRG, MIR378J, DDX52		3	0
chr17:35,632,081-35,819,506	187,425	q12	10	100	Gain	P6_C1	Complete	ACACA, C17orf78, TADA2		3	0
chr17:34,051,150-35,632,081	1,580,931	q12	10	67	AI	P6_C1	Complete	AP2B1, RASL1	hsa-mir-2909	55	1
chr17:33,917,012-34,051,150	134,138	q12	10	6	Gain	P6_C1	Complete	AP2B1		1	0
chr17:32,340,948-32,942,574	601,626	q12	10	81	Loss	P6_C1	Complete	ASIC2, BC062794, LINC019		11	0
chr17:30,789,283-33,816,223	3,026,940	q11.2 - q12	10	31	AI	P6_C1	Complete	PSMD11, AK307275, CDK5		34	0
chr17:30,652,705-30,789,283	136,578	q11.2	10	10	Gain	P6_C1	Complete	C17orf75, MIR	hsa-mir-632	4	1
chr17:29,873,697-30,587,181	713,484	q11.2	10	20	Gain	P6_C1	Complete	TRNA_Thr, MI	hsa-mir-193a	15	2
chr17:29,783,603-30,587,181	803,578	q11.2	10	18	AI	P6_C1	Complete	RAB11FIP4, M	hsa-mir-193a	17	2
chr17:21,977,286-22,242,355	265,069	11.2 - p11.	10	100	Gain	P7_C1	Complete	TRNA, JB137816, MTRNR2		4	0
chr16:86,478,529-86,501,120	22,591	q24.1	10	0	Loss	P7_C1	Complete			0	0
chr16:731,415-736,000	4,585	p13.3	10	100	Gain	P1_C3	Complete	STUB1, JMJD8, WDR24		3	0
chr16:71,481,155-71,484,833	3,678	q22.2	10	0	Gain	P1_C3	Complete	ZNF23		1	0
chr16:31,922,202-31,933,060	10,858	p11.2	10	100	Gain	P1_C3	Complete	ZNF267		1	0
chr15:50,836,810-50,972,610	135,800	q21.2	10	1	Gain	P7_C1	Complete	USP50, TRPM7, TRPM7		3	0
chr15:20,071,673-20,771,825	700,152	11.1 - q11.	10	100	Gain	P7_C1	Complete	DQ576041, DQ571479, BC1		14	0
chr14:60,903,046-60,933,593	30,547	q23.1	10	0	Loss	P1_C3	Complete	C14orf39		1	0
chr14:19,327,823-20,444,803	1,116,980	q11.2	10	100	AI	P7_C1	Complete	OR11H12, LINC02297, LOC		42	0
chr14:104,843,202-104,987,571	144,369	q32.33	10	99	Loss	P7_C1	Complete	AX746996		1	0
chr14:101,539,257-101,636,468	97,211	q32.31	10	1	Loss	P7_C1	Complete	MEG9, BC148240, LINC022		3	0
chr13:65,199,451-65,223,125	23,674	q21.31	10	0	Loss	P1_C3	Complete			0	0
chr12:85,406,598-85,425,717	19,119	q21.31	10	0	Loss	P1_C3	Complete	TSPAN19		1	0
chr12:80,733,458-80,761,420	27,962	q21.31	10	0	Loss	P1_C3	Complete	OTOGL		1	0
chr12:52,665,539-52,804,288	138,749	q13.13	10	83	Loss	P7_C1	Complete	KRT86, KRT81, KRT83, KR		6	0
chr12:34,433,267-35,800,000	1,366,733	p11.1 - q11	10	31	Gain	P1_C3	Complete			0	0
chr12:32,134,535-32,137,538	3,003	p11.21	10	0	Gain	P1_C3	Complete	KIAA1551, RESF1		2	0
chr12:31,821,063-32,029,691	208,628	p11.21	10	63	Gain	P7_C1	Complete	ETFBKMT, METTL20, AM		4	0
chr12:21,329,564-21,331,875	2,311	p12.1	10	100	Loss	P1_C3	Complete	SLCO1B1		1	0
chr12:21,199,750-21,242,964	43,214	p12.2	10	100	Loss	P1_C3	Complete	SLCO1B3, SLCO1B7		2	0
chr12:133,500,911-133,792,201	291,290	q24.33	10	84	Gain	P1_C3	Complete	ZNF605, ZNF26, LOC10192		9	0
chr12:131,752,611-131,861,853	109,242	q24.33	10	100	Loss	P7_C1	Complete	BC042649, LINC02415, LIN		4	0
chr12:131,620,646-131,675,762	55,116	q24.33	10	100	Loss	P7_C1	Complete	GPR133, ADGRD1, LINC01		4	0
chr12:122,967,411-123,060,114	92,703	q24.31	10	0	Gain	P7_C1	Complete	ZCCHC8, SNORA9, RSRC2		4	0
chr12:114,552,977-114,615,733	62,756	q24.21	10	0	Loss	P7_C1	Complete			0	0
chr11:2,611,513-2,770,719	159,206	p15.5	10	32	Loss	P7_C1	Complete	KCNQ1, KCNQ1OT1		2	0
chr11:2,434,299-2,444,227	9,928	p15.5	10	100	Loss	P7_C1	Complete	TRPM5		1	0
chr11:2,062,613-2,088,838	26,225	p15.5	10	8	Gain	P1_C3	Complete			0	0
chr11:134,717,764-134,896,811	179,047	q25	10	84	Loss	P7_C1	Complete	AK130852, LOC100507548		2	0
chr11:113,270,412-113,272,453	2,041	q23.2	10	100	Loss	P7_C1	Complete	ANKK1		1	0
chr11:102,660,761-102,663,452	2,691	q22.2	10	0	Loss	P1_C3	Complete	MMP1, WTAPP1		2	0
chr10:94,167,942-94,224,457	56,515	q23.33	10	10	Gain	P7_C1	Complete	MARK2P9, AK124121, IDE		3	0
chr10:90,350,284-90,357,404	7,120	q23.31	10	100	Loss	P1_C3	Complete	LIPJ		1	0
chr10:80,481,660-80,762,906	281,246	q22.3	10	1	Loss	P7_C1	Complete	AX747983, ZMIZ1-AS1		2	0
chr10:70,235,908-70,267,466	31,558	q21.3	10	16	Gain	P7_C1	Complete	SLC25A16		1	0

chr10:60,369,809-60,415,296	45,487	q21.1	10	0	Gain	P1_C3	Complete	BICC1		1	0
chr10:50,530,713-50,532,747	2,034	q11.23	10	0	Loss	P7_C1	Complete	C10orf71		1	0
chr10:49,911,229-50,341,936	430,707	q11.23	10	6	Loss	P7_C1	Complete	WDFY4, WDF	hsa-mir-4294	7	1
chr10:42,467,126-42,819,463	352,337	q11.21	10	100	Gain	P1_C3	Complete			0	0
chr10:134,649,258-134,783,002	133,744	q26.3	10	67	Loss	P7_C1	Complete	TTC40, CFAP46, LINC0116		6	0
chr10:131,426,841-131,696,690	269,849	q26.3	10	0	Loss	P7_C1	Complete	MGMT, MIR42	hsa-mir-4297	3	1
chr10:1,692,729-1,738,136	45,407	p15.3	10	4	Loss	P7_C1	Complete	ADARB2		1	0
chr1:979,185-1,093,517	114,332	p36.33	10	100	Loss	P6_C1	Complete	AGRN, AK310350, LOC100		8	0
chr1:9,656,788-9,795,378	138,590	p36.22	10	19	Loss	P6_C1	Complete	TMEM201, PIK3CD-AS1, C		7	0
chr1:9,063,251-9,365,743	302,492	6.23 - p36.	10	29	Loss	P6_C1	Complete	SLC2A7, SLC2	hsa-mir-34a	9	1
chr1:87,023,185-87,039,174	15,989	p22.3	10	100	Loss	P1_C3	Complete	CLCA4, CLCA4-AS1		2	0
chr1:85,010,878-85,037,636	26,758	p22.3	10	0	Loss	P1_C3	Complete	SPATA1, CTBS		2	0
chr1:84,895,911-84,935,738	39,827	31.1 - p22.	10	0	Loss	P1_C3	Complete			0	0
chr1:84,294,745-84,649,712	354,967	p31.1	10	24	Loss	P1_C3	Complete	BC036594, LINC01725, LO		8	0
chr1:83,695,087-84,895,911	1,200,824	p31.1	10	36	AI	P1_C3	Complete	BC043544, LINC01712, BC		15	0
chr1:83,585,827-83,695,087	109,260	p31.1	10	100	Loss	P1_C3	Complete	LINC01362		1	0
chr1:82,987,363-83,585,827	598,464	p31.1	10	63	AI	P1_C3	Complete	U80773, LINC01361, LINC		3	0
chr1:82,876,615-82,987,363	110,748	p31.1	10	0	Loss	P1_C3	Complete			0	0
chr1:82,041,786-82,876,615	834,829	p31.1	10	1	AI	P1_C3	Complete	LPHN2, ADGRL2, Mir_544		3	0
chr1:81,984,467-82,041,786	57,319	p31.1	10	0	Loss	P1_C3	Complete	LOC101927434, LPHN2, AI		3	0
chr1:81,891,838-81,984,467	92,629	p31.1	10	8	AI	P1_C3	Complete	LOC101927434, LPHN2, AI		3	0
chr1:81,779,595-81,891,838	112,243	p31.1	10	57	Loss	P1_C3	Complete	LPHN2, ADGRL2		2	0
chr1:80,461,852-81,779,595	1,317,743	p31.1	10	100	AI	P1_C3	Complete	LINC01781, LPHN2, ADGR		3	0
chr1:80,461,852-81,618,394	1,156,542	p31.1	10	100	Loss	P1_C3	Complete	LINC01781		1	0
chr1:80,074,062-80,394,896	320,834	p31.1	10	100	AI;Loss	P1_C3;P1_C3	Complete;Complete			0	0
chr1:8,279,708-8,614,184	334,476	p36.23	10	14	Loss	P6_C1	Complete	SLC45A1, BC113958, RERE		5	0
chr1:79,858,355-79,992,282	133,927	p31.1	10	17	Loss	P1_C3	Complete			0	0
chr1:79,381,237-79,631,578	250,341	p31.1	10	17	Loss	P1_C3	Complete	ELTD1, ADGRL4		2	0
chr1:78,764,689-79,992,282	1,227,593	p31.1	10	9	AI	P1_C3	Complete	MGC27382, PTGFR, IFI44L		7	0
chr1:78,764,689-79,067,071	302,382	p31.1	10	5	Loss	P1_C3	Complete	MGC27382, PTGFR		2	0
chr1:78,204,351-78,679,325	474,974	p31.1	10	6	AI;Loss	P1_C3;P1_C3	Complete;Complete	USP33, MIGA1, FAM73A, N		8	0
chr1:77,943,052-78,053,358	110,306	p31.1	10	0	Loss	P1_C3	Complete	AK5, ZZZ3		2	0
chr1:77,506,270-77,842,351	336,081	p31.1	10	4	AI;Loss	P1_C3;P1_C3	Complete;Complete	ST6GALNAC5, MIR7156, A		5	0
chr1:76,882,130-77,418,871	536,741	p31.1	10	30	AI	P1_C3	Complete	ST6GALNAC3, LINC02567,		3	0
chr1:76,862,102-76,882,130	20,028	p31.1	10	0	Loss	P1_C3	Complete	ST6GALNAC3		1	0
chr1:75,097,454-76,862,102	1,764,648	p31.1	10	8	AI	P1_C3	Complete	C1orf173, ERICH3, CRYZ,		18	0
chr1:75,038,685-76,516,226	1,477,541	p31.1	10	9	Loss	P1_C3	Complete	CR627203, ERICH3-AS1, C		19	0
chr1:74,372,848-75,038,685	665,837	p31.1	10	5	AI	P1_C3	Complete	LRRIQ3, FPGT, FPGT-TNN		7	0
chr1:74,372,848-74,707,481	334,633	p31.1	10	9	Loss	P1_C3	Complete	LRRIQ3, FPGT, FPGT-TNN		4	0
chr1:71,847,244-74,333,047	2,485,803	p31.1	10	20	Loss	P1_C3	Complete	NEGR1-IT1, NEGR1, BC04		5	0
chr1:71,005,838-74,333,047	3,327,209	p31.1	10	18	AI	P1_C3	Complete	BC041441, LIN	hsa-mir-186	12	1
chr1:71,005,838-71,511,289	505,451	p31.1	10	17	Loss	P1_C3	Complete	BC041441, LINC01788, PTG		3	0
chr1:70,885,469-70,902,004	16,535	p31.1	10	0	Loss	P1_C3	Complete	CTH		1	0
chr1:70,575,284-70,651,230	75,946	p31.1	10	3	Loss	P1_C3	Complete	LRRC7, LRRC40		2	0
chr1:70,505,116-70,551,256	46,140	p31.1	10	0	Loss	P1_C3	Complete	LRRC7		1	0
chr1:70,060,521-70,290,434	229,913	p31.1	10	11	Loss	P1_C3	Complete	LRRC7		1	0
chr1:69,682,164-70,505,116	822,952	31.2 - p31.	10	8	AI	P1_C3	Complete	LINC01758, LRRC7, PIN1P		3	0
chr1:69,622,172-69,682,164	59,992	p31.2	10	0	Loss	P1_C3	Complete	LINC01707		1	0
chr1:68,840,655-69,085,434	244,779	31.3 - p31.	10	5	Loss	P1_C3	Complete	RPE65, DEPDC1, DEPDC1-		4	0
chr1:68,405,797-69,622,172	1,216,375	31.3 - p31.	10	2	AI	P1_C3	Complete	GNG12-AS1, D	hsa-mir-1262	10	1

chr1:68,370,341-68,405,797	35,456	p31.3	10	0	Loss	P1_C3	Complete	GNG12-AS1		1	0
chr1:68,046,291-68,370,341	324,050	p31.3	10	2	AI	P1_C3	Complete	GADD45A, GNG12, U7, GN		4	0
chr1:67,555,967-68,029,315	473,348	p31.3	10	1	AI	P1_C3	Complete	C1orf141, IL23R, IL12RB2,		4	0
chr1:67,555,967-67,772,557	216,590	p31.3	10	0	Loss	P1_C3	Complete	C1orf141, IL23R		2	0
chr1:67,263,282-67,376,005	112,723	p31.3	10	13	Loss	P1_C3	Complete	INSL5, WDR78		2	0
chr1:66,988,684-67,019,088	30,404	p31.3	10	0	Loss	P1_C3	Complete	SGIP1		1	0
chr1:66,901,573-66,957,011	55,438	p31.3	10	0	Loss	P1_C3	Complete			0	0
chr1:66,621,739-67,263,282	641,543	p31.3	10	1	AI	P1_C3	Complete	PDE4B, MIR31	hsa-mir-3117	5	1
chr1:66,543,232-66,621,739	78,507	p31.3	10	1	Loss	P1_C3	Complete	PDE4B, U4		2	0
chr1:66,170,681-66,269,188	98,507	p31.3	10	2	Loss	P1_C3	Complete	PDE4B		1	0
chr1:64,557,776-66,543,232	1,985,456	p31.3	10	10	AI	P1_C3	Complete	ROR1, BC0409	hsa-mir-101-	22	1
chr1:64,076,996-64,541,466	464,470	p31.3	10	2	AI	P1_C3	Complete	PGM1, Mir_544, ROR1		3	0
chr1:64,001,362-64,076,996	75,634	p31.3	10	0	Loss	P1_C3	Complete	EFCAB7, DLEU2L, PGM1		3	0
chr1:63,579,044-63,627,425	48,381	p31.3	10	3	Loss	P1_C3	Complete	LINC00466		1	0
chr1:63,023,781-64,001,362	977,581	p31.3	10	1	AI	P1_C3	Complete	DOCK7, ANGPTL3, ATG4C		13	0
chr1:62,850,023-63,023,781	173,758	p31.3	10	0	Loss	P1_C3	Complete	USP1, DOCK7		2	0
chr1:62,739,633-62,740,576	943	p31.3	10	0	Loss	P1_C3	Complete	KANK4		1	0
chr1:62,658,334-62,675,845	17,511	p31.3	10	0	Loss	P1_C3	Complete	L1TD1		1	0
chr1:62,271,228-62,658,334	387,106	p31.3	10	9	AI	P1_C3	Complete	PATJ, INADL,	hsa-mir-3116	4	2
chr1:62,224,719-62,271,228	46,509	p31.3	10	0	Loss	P1_C3	Complete	PATJ, INADL		2	0
chr1:61,223,186-62,224,719	1,001,533	32.1 - p31.	10	2	AI	P1_C3	Complete	LOC101926964, AK097193,		10	0
chr1:61,173,805-61,223,186	49,381	p32.1	10	0	Loss	P1_C3	Complete	LOC101926964, AK097193		2	0
chr1:60,363,611-61,173,805	810,194	p32.1	10	6	AI	P1_C3	Complete	CYP2J2, C1orf87, LINC017		5	0
chr1:60,300,944-60,363,611	62,667	p32.1	10	0	Loss	P1_C3	Complete	HOOK1, CYP2J2		2	0
chr1:57,641,292-60,300,944	2,659,652	32.2 - p32.	10	5	AI	P1_C3	Complete	DAB1, BC047487, BC03397		18	0
chr1:57,599,516-57,641,292	41,776	p32.2	10	0	Loss	P1_C3	Complete	DAB1		1	0
chr1:57,383,529-57,599,516	215,987	p32.2	10	3	AI	P1_C3	Complete	C8A, C8B, DAB1		3	0
chr1:57,309,182-57,383,529	74,347	p32.2	10	0	Loss	P1_C3	Complete	C8A		1	0
chr1:57,185,587-57,228,189	42,602	p32.2	10	4	Loss	P1_C3	Complete	FYB2, C1orf168		2	0
chr1:56,930,780-57,185,587	254,807	p32.2	10	2	AI	P1_C3	Complete	PPAP2B, PLPP3, LOC10192		6	0
chr1:55,528,204-56,897,195	1,368,991	32.3 - p32.	10	3	AI	P1_C3	Complete	PCSK9, USP24, LOC100507		10	0
chr1:55,519,773-55,528,204	8,431	p32.3	10	0	Loss	P1_C3	Complete	PCSK9		1	0
chr1:55,266,707-55,519,773	253,066	p32.3	10	19	AI	P1_C3	Complete	TTC22, LEXM, C1orf177, D		9	0
chr1:55,242,629-55,266,707	24,078	p32.3	10	0	Loss	P1_C3	Complete	TTC22		1	0
chr1:55,054,837-55,184,252	129,415	p32.3	10	32	Loss	P1_C3	Complete	ACOT11, FAM151A, MROH		6	0
chr1:54,562,238-55,184,252	622,014	p32.3	10	12	AI	P1_C3	Complete	TCEANC2, CDCP2, CYB5R		12	0
chr1:54,433,403-54,562,238	128,835	p32.3	10	3	Loss	P1_C3	Complete	LRRC42, LDLRAD1, TMEM		5	0
chr1:54,307,305-54,433,403	126,098	p32.3	10	3	AI	P1_C3	Complete	YIPF1, DIO1, HSPB11, LRR		4	0
chr1:54,255,873-54,307,305	51,432	p32.3	10	0	Loss	P1_C3	Complete	NDC1		1	0
chr1:53,683,501-54,255,873	572,372	p32.3	10	20	AI	P1_C3	Complete	C1orf123, CZIB, MAGOH, L		11	0
chr1:53,675,769-53,683,501	7,732	p32.3	10	0	Loss	P1_C3	Complete	CPT2, C1orf123, CZIB		3	0
chr1:53,372,022-53,675,769	303,747	p32.3	10	23	AI	P1_C3	Complete	ECHDC2, SCP2, PODN, SL(		6	0
chr1:53,183,173-53,372,022	188,849	p32.3	10	3	Loss	P1_C3	Complete	ZYG11B, ZYG11A, ECHDC		3	0
chr1:52,854,534-53,183,173	328,639	p32.3	10	1	AI	P1_C3	Complete	ORC1, PRPF38A, TUT4, ZC		9	0
chr1:52,829,304-52,854,534	25,230	p32.3	10	0	Loss	P1_C3	Complete	CC2D1B, ORC1		2	0
chr1:51,672,190-52,829,304	1,157,114	p32.3	10	9	AI	P1_C3	Complete	LINC01562, RN	hsa-mir-761	17	1
chr1:51,564,451-51,672,190	107,739	p32.3	10	14	Loss	P1_C3	Complete	C1orf185, LINC01562		2	0
chr1:49,344,279-50,559,491	1,215,212	p33	10	10	Loss	P1_C3	Complete	LOC101929721, AGBL4, AC		4	0
chr1:48,405,504-51,564,451	3,158,947	p33 - p32.3	10	5	AI	P1_C3	Complete	TRABD2B, SKINTL, SKINT		15	0
chr1:48,375,457-48,405,504	30,047	p33	10	0	Loss	P1_C3	Complete	TRABD2B		1	0

chr1:47,849,489-48,375,457	525,968	p33	10	2	AI	P1_C3	Complete	AX748181, LINC01389, FO		6	0
chr1:47,717,517-47,849,489	131,972	p33	10	5	Loss	P1_C3	Complete	STIL, CMPK1, LINC01389		3	0
chr1:46,746,180-47,717,517	971,337	p34.1 - p33	10	13	AI	P1_C3	Complete	LRRC41, UQCRH, NSUN4,		28	0
chr1:46,743,685-46,746,180	2,495	p34.1	10	0	Loss	P1_C3	Complete	RAD54L, LRRC41		2	0
chr1:46,500,462-46,512,006	11,544	p34.1	10	21	Loss	P1_C3	Complete	MAST2, PIK3R3, LOC1101		3	0
chr1:46,300,865-46,500,462	199,597	p34.1	10	6	AI	P1_C3	Complete	MAST2		1	0
chr1:46,173,353-46,300,865	127,512	p34.1	10	8	Loss	P1_C3	Complete	IPP, MAST2		2	0
chr1:43,785,044-43,806,113	21,069	p34.2	10	0	Loss	P1_C3	Complete	TIE1, MPL		2	0
chr1:42,524,413-42,616,276	91,863	p34.2	10	0	Loss	P1_C3	Complete			0	0
chr1:42,046,315-42,524,413	478,098	p34.2	10	0	AI	P1_C3	Complete	HIVEP3		1	0
chr1:42,024,588-42,046,315	21,727	p34.2	10	0	Loss	P1_C3	Complete	HIVEP3		1	0
chr1:40,981,271-41,089,463	108,192	p34.2	10	10	Gain	P6_C1	Complete	EXO5, ZNF684, RIMS3		3	0
chr1:40,882,598-40,980,681	98,083	p34.2	10	12	AI;AI;Gain	P1_C3;P6_C1;P6_C1	Complete;Complete;(	SMAP2, ZFP69B, ZFP69, EX		4	0
chr1:40,539,413-40,869,908	330,495	p34.2	10	10	Gain	P6_C1	Complete	PPT1, RLF, TMCO2, ZMPS		6	0
chr1:39,876,129-39,884,102	7,973	p34.3	10	0	Loss	P1_C3	Complete	MACF1, KIAA0754		2	0
chr1:39,412,023-39,710,788	298,765	p34.3	10	14	Gain	P6_C1	Complete	AKIRIN1, NDUFS5, Mir_56		5	0
chr1:39,097,607-39,293,840	196,233	p34.3	10	11	Loss	P1_C3	Complete			0	0
chr1:38,751,352-38,917,393	166,041	p34.3	10	2	Loss	P1_C3	Complete			0	0
chr1:36,553,483-36,625,781	72,298	p34.3	10	0	Gain	P6_C1	Complete	TEKT2, ADPRHL2, COL8A		5	0
chr1:36,238,746-36,550,625	311,879	p34.3	10	2	AI;AI;Gain	P1_C3;P6_C1;P6_C1	Complete;Complete;(	AGO4, AGO1, AK025726, A		5	0
chr1:36,055,940-36,172,748	116,808	p34.3	10	1	Gain	P6_C1	Complete	TFAP2E, PSMB2		2	0
chr1:35,574,559-35,609,297	34,738	p34.3	10	3	Loss	P1_C3	Complete	ZMYM1		1	0
chr1:35,479,290-35,902,952	423,662	p34.3	10	2	Gain	P6_C1	Complete	ZMYM6, ZMYM1, BX5378		9	0
chr1:35,380,394-35,479,290	98,896	p34.3	10	0	Loss	P1_C3	Complete	DLGAP3, LOC653160, TMI		5	0
chr1:27,970,491-28,421,615	451,124	36.11 - p35	10	14	Loss	P6_C1	Complete	LINC02574, IFI6, FAM76A,		11	0
chr1:27,724,551-27,875,310	150,759	p36.11	10	18	Loss	P6_C1	Complete	WASF2, AHDC1		2	0
chr1:27,215,989-27,410,945	194,956	p36.11	10	6	Loss	P6_C1	Complete	GPN2, GPATCH3, NR0B2, I		10	0
chr1:26,921,861-27,178,392	256,531	p36.11	10	1	Loss	P6_C1	Complete	LOC101928728, ARID1A, P		5	0
chr1:26,682,372-29,260,519	2,578,147	36.11 - p35	10	12	AI	P1_C3	Complete	ZNF683, LIN28	hsa-mir-1976	74	1
chr1:247,822,077-248,312,884	490,807	q44	10	100	AI	P7_C1	Complete	OR13G1, OR6F1, OR14A2,		20	0
chr1:24,853,163-26,623,937	1,770,774	p36.11	10	29	AI;AI;Loss	P1_C3;P6_C1;P6_C1	Complete;Complete;(	RCAN3, LOC100506985, N(		44	0
chr1:24,478,080-24,488,271	10,191	p36.11	10	0	Loss	P1_C3	Complete	IFNLR1		1	0
chr1:24,433,055-24,435,829	2,774	p36.11	10	0	Loss	P1_C3	Complete	MYOM3		1	0
chr1:23,759,728-24,433,055	673,327	6.12 - p36.	10	20	AI	P1_C3	Complete	ASAP3, BC038455, E2F2, L		24	0
chr1:23,738,696-23,759,728	21,032	p36.12	10	0	Loss	P1_C3	Complete	TCEA3, ASAP3		2	0
chr1:227,820,384-227,852,739	32,355	q42.13	10	34	Gain	P1_C3	Complete	ZNF678		1	0
chr1:22,922,297-23,738,696	816,399	p36.12	10	4	AI	P1_C3	Complete	EPHA8, MIR61	hsa-mir-4253	23	2
chr1:22,909,649-22,922,297	12,648	p36.12	10	0	Loss	P1_C3	Complete	EPHA8		1	0
chr1:22,176,800-22,909,649	732,849	p36.12	10	25	AI	P1_C3	Complete	HSPG2, CELA3B, CELA3A		10	0
chr1:22,169,273-22,176,800	7,527	p36.12	10	100	Loss	P1_C3	Complete	HSPG2		1	0
chr1:21,070,820-22,169,273	1,098,453	p36.12	10	32	AI	P1_C3	Complete	HP1BP3, MIR1	hsa-mir-1256	14	1
chr1:21,048,082-21,070,820	22,738	p36.12	10	0	Loss	P1_C3	Complete	SH2D5, HP1BP3		2	0
chr1:21,012,602-21,016,228	3,626	p36.12	10	0	Loss	P1_C3	Complete	KIF17		1	0
chr1:20,475,649-21,012,602	536,953	p36.12	10	4	AI	P1_C3	Complete	PLA2G2F, PLA2G2C, UBXI		17	0
chr1:20,456,280-20,475,649	19,369	p36.12	10	0	Loss	P1_C3	Complete	PLA2G2F		1	0
chr1:196,963,333-196,977,676	14,343	q31.3	10	100	Loss	P1_C3	Complete	CFHR5		1	0
chr1:19,076,861-20,456,280	1,379,419	6.13 - p36.	10	8	AI	P1_C3	Complete	TAS1R2, MIR4	hsa-mir-1290	33	1
chr1:19,011,666-19,076,861	65,195	p36.13	10	0	Loss	P1_C3	Complete	PAX7		1	0
chr1:18,806,851-18,808,014	1,163	p36.13	10	0	Loss	P1_C3	Complete	KLHDC7A		1	0
chr1:171,175,203-171,190,847	15,644	q24.3	10	100	Gain	P1_C3	Complete	FMO2		1	0

chr1:17,548,895-17,731,753	182,858	p36.13	10	22	Loss	P1_C3	Complete	PADI1, Mir_584, PADI3, MI		6	0
chr1:16,763,653-18,806,851	2,043,198	p36.13	10	41	AI	P1_C3	Complete	SPATA21, NECAP2, LINC0		46	0
chr1:12,253,008-12,606,559	353,551	p36.22	10	53	Loss	P6_C1	Complete	TNFRSF1B, VPS13D, SNOI		4	0
chr1:112,546,269-112,571,200	24,931	p13.2	10	0	Loss	P7_C1	Complete			0	0
chr1:11,865,993-12,202,875	336,882	p36.22	10	4	Loss	P6_C1	Complete	MTHFR, CLCN6, NPPA-AS		11	0
chr1:11,010,423-12,779,573	1,769,150	6.22 - p36.	10	31	AI	P1_C3	Complete	C1orf127, TARDBP, MASP2		43	0
chr1:10,523,645-10,708,791	185,146	p36.22	10	25	AI	P1_C3	Complete	DFFA, PEX14, CASZ1		3	0
chr1:1,116,668-1,435,417	318,749	p36.33	10	100	Loss	P6_C1	Complete	TTLL10, TNFRSF18, TNFR		34	0
chr1:0-909,867	909,867	p36.33	10	99	Loss	P6_C1	Complete	LOC102725121	hsa-mir-1302	47	1

Supplementary Table S2C. CNVs in 10 spinal meningiomas, excluding CNVs in chromosome 22. Chromosomal coordinates of the region (hg19); the region length in bp; the chromosomal cytoband; frequency of the region occurrence in the tumor samples; percent of the region's overlap with a known CNV; type of the variation (gain, loss, or allelic imbalance); sample ID; type of the participation of an individual CNV in the region (complete or partial); gene symbols; microRNA symbols; number of genes in the region; and the number of microRNA genes found in the region are shown from left to right.

Chromosomal region	Region length (bp)	Cytoband	Frequency of the CNV occurrence in samples (%)	Overlap with known CNVs (%)	Type of CNV	Sample ID	Participation	Gene symbols	miRNAs symbols	Gene count	miRNAs count
chr7:38,307,524-38,365,774	58,250	p14.1	100	100	Gain;Gain;(	P1_S1;P1_S2;P1_S3;P	Complete;Compl	*TRGC2, TARP, TCRG*		5	0
chr14:22,501,912-22,972,885	470,973	q11.2	100	100	Gain;Gain;(	P1_S1;P1_S2;P1_S3;P	Complete;Compl	*TCRA, TRA@, TRAV2*		42	0
chr7:142,307,376-142,445,944	138,568	q34	90	86	Gain;Gain;(	P1_S1;P1_S2;P1_S3;P	Complete;Compl	*TCRBV5S1A1T, TCRE*		17	0
chr10:47,593,880-47,822,143	228,263	q11.22	40	100	AI;AI;AI;A	P1_S1;P1_S2;P1_S3;P	Complete;Compl	*ANTXRLP1, AK05731*		6	0
chr12:38,029,427-38,150,241	120,814	q11	40	100	Gain;Gain;(	P1_S1;P1_S2;P1_S3;P	Complete;Complete;Complete;Complete			0	0
chr7:61,747,794-62,126,898	379,104	q11.21	30	100	Gain;Gain;(	P1_S1;P2_S2;P5_S1	Complete;Complete;Complet			0	0
chr14:21,353,205-21,421,877	68,672	q11.2	30	100	AI;AI;AI	P4_S1;P4_S2;P4_S3	Complete;Compl	*RNASE3, ECRP, RNA*		3	0
chr19:37,898,990-37,906,504	7,514	q13.12	30	0	Gain;Gain;(	P1_S1;P2_S2;P5_S1	Complete;Compl	*ZNF569*		1	0
chr3:64,444,942-64,522,288	77,346	p14.1	20	0	AI;AI	P2_S1;P2_S2	Complete;Compl	*ADAMTS9*		1	0
chr11:50,520,747-50,697,498	176,751	p11.12	20	100	Gain;Gain	P1_S1;P5_S1	Complete;Complete			0	0
chr19:27,862,694-28,061,733	199,039	q11	20	100	Gain;Gain	P1_S1;P2_S2	Complete;Complete			0	0
chr1:110,736,199-110,756,091	19,892	p13.3	10	0	Loss	P5_S1	Complete	*SLC6A17, LINC02586*		3	0
chr1:112,531,606-112,578,427	46,821	p13.2	10	0	Loss	P5_S1	Complete	*KCND3, LINC01750,*		3	0
chr1:113,425,388-113,450,930	25,542	p13.2	10	100	Gain	P5_S1	Complete			0	0
chr1:113,460,670-113,493,754	33,084	p13.2	10	100	Gain	P5_S1	Complete	*AKR7A2P1, SLC16A1*		2	0
chr1:150,260,171-150,431,301	171,130	q21.2 - q21.3	10	60	Gain	P5_S1	Complete	*MRPS21, PRPF3, RPI*		3	0
chr1:150,604,371-150,884,523	280,152	q21.3	10	3	Gain	P5_S1	Complete	*GOLPH3L, HORMAD*		6	0
chr1:153,973,308-154,125,271	151,963	q21.3	10	3	Gain	P5_S1	Complete	*NUP210L, MIR5698*		2	0
chr1:175,490,384-175,593,871	103,487	q25.1	10	100	Loss	P5_S1	Complete	*TNR, AK093214, BC0*		3	0
chr1:179,095,618-179,268,053	172,435	q25.2	10	2	Gain	P5_S1	Complete	*ABL2, DD413682, SO*		3	0
chr1:201,175,686-201,183,028	7,342	q32.1	10	16	Loss	P5_S1	Complete	*IGFN1*		1	0
chr1:23,855,044-23,885,489	30,445	p36.12	10	0	Gain	P5_S1	Complete	*E2F2, AX748204, ID3*		3	0
chr1:235,360,466-235,600,430	239,964	q42.3	10	3	Gain	P5_S1	Complete	*ARID4B, GGPS1, TB(*		3	0
chr1:28,296,296-28,403,725	107,429	p35.3	10	0	Gain	P5_S1	Complete	*EYA3*		1	0
chr1:28,563,951-28,660,344	96,393	p35.3	10	6	Gain	P5_S1	Complete	*ATP5IF1, ATPIF1, JA*		5	0
chr1:28,677,518-29,301,914	624,396	p35.3	10	5	Gain	P5_S1	Complete	*PHACTR4, SNHG3, S*		19	0
chr1:30,497,110-30,544,270	47,160	p35.2	10	100	Loss	P5_S1	Complete	*BC042538, LINC0164*		2	0
chr1:30,709,695-30,981,393	271,698	p35.2	10	100	Loss	P5_S1	Complete			0	0
chr1:32,974,587-33,096,248	121,661	p35.1	10	6	Gain	P5_S1	Complete	*ZBTB8A, ZBTB8OS*		2	0
chr1:34,350,366-34,394,470	44,104	p35.1	10	0	Loss	P5_S1	Complete	*CSMD2, CSMD2-AS1,*		3	0
chr1:35,665,487-35,836,212	170,725	p34.3	10	2	Gain	P5_S1	Complete	*ZMYM4, ZMYM4-AS1*		3	0
chr1:42,031,379-42,047,221	15,842	p34.2	10	0	Loss	P5_S1	Complete	*HIVEP3*		1	0
chr1:48,282,480-48,459,863	177,383	p33	10	0	Loss	P5_S1	Complete	*TRABD2B*		1	0
chr1:52,905,413-53,097,412	191,999	p32.3	10	1	Gain	P5_S1	Complete	*TUT4, ZCCHC11, GP*		3	0

chr1:53,188,909-53,319,562	130,653	p32.3	10	2	Gain	P5_S1	Complete	*ZYG11B, ZYG11A*		2	0
chr1:62,456,903-62,504,343	47,440	p31.3	10	0	Gain	P5_S1	Complete	*PATJ, INADL*		2	0
chr1:9,856,177-10,362,151	505,974	p36.22	10	2	Gain	P5_S1	Complete	*CLSTN1, C*	hsa-mir-1	10	1
chr2:0-470,504	470,504	p25.3	10	88	Loss	P5_S1	Complete	*FAM110C, SH3YL1, A*		6	0
chr2:1,204,824-1,276,701	71,877	p25.3	10	16	Loss	P5_S1	Complete	*SNTG2*		1	0
chr2:1,423,172-1,465,311	42,139	p25.3	10	100	Loss	P5_S1	Complete	*TPO*		1	0
chr2:102,806,372-102,836,648	30,276	q12.1	10	2	Loss	P5_S1	Complete	*IL1RL2*		1	0
chr2:119,364,013-119,419,375	55,362	q14.2	10	0	Loss	P5_S1	Complete			0	0
chr2:163,185,162-163,233,231	48,069	q24.2	10	3	Loss	P5_S1	Complete	*GCA, KCNH7*		2	0
chr2:17,679,264-17,697,234	17,970	p24.2	10	0	Loss	P5_S1	Complete	*RAD51AP2*		1	0
chr2:2,610,653-2,752,111	141,458	p25.3	10	5	Loss	P5_S1	Complete			0	0
chr2:203,040,470-203,420,006	379,536	q33.1 - q33.2	10	17	Gain	P5_S1	Complete	*LOC100652824, KIAA*		10	0
chr2:203,424,957-204,302,729	877,772	q33.2	10	7	Gain	P5_S1	Complete	*BMPR2, FAM117B, IC*		10	0
chr2:21,230,714-21,233,662	2,948	p24.1	10	0	Loss	P5_S1	Complete	*APOB*		1	0
chr2:218,623,066-218,689,541	66,475	q35	10	14	Loss	P5_S1	Complete	*TNS1*		1	0
chr2:238,239,548-238,307,688	68,140	q37.3	10	0	Loss	P5_S1	Complete	*COL6A3*		1	0
chr2:240,113,554-240,419,667	306,113	q37.3	10	13	Loss	P5_S1	Complete	*MGC16025*	hsa-mir-4	5	1
chr2:240,922,142-240,979,466	57,324	q37.3	10	1	Loss	P5_S1	Complete	*NDUFA10, OR6B2*		2	0
chr2:241,064,765-241,239,644	174,879	q37.3	10	7	Loss	P5_S1	Complete	*MYEOV2, COPS9, OT*		3	0
chr2:241,341,946-241,536,120	194,174	q37.3	10	25	Loss	P5_S1	Complete	*GPC1, PP1*	hsa-mir-1	9	1
chr2:241,566,650-241,577,867	11,217	q37.3	10	0	Loss	P5_S1	Complete	*GPR35*		1	0
chr2:242,929,198-243,199,373	270,175	q37.3	10	64	Loss	P5_S1	Complete	*LINC01237, LOC285*		6	0
chr2:32,292,566-32,719,210	426,644	p22.3	10	44	Gain	P5_S1	Complete	*SPAST, SLC30A6, NL*		5	0
chr2:44,147,455-44,186,364	38,909	p21	10	0	Gain	P5_S1	Complete	*LRPPRC*		1	0
chr2:55,490,816-55,711,259	220,443	p16.1	10	13	Gain	P5_S1	Complete	*MTIF2, PRORSD1P,*		3	0
chr2:61,234,572-61,324,874	90,302	p16.1 - p15	10	4	Gain	P5_S1	Complete	*PUS10, PEX13, KIAA*		3	0
chr2:61,448,716-62,066,996	618,280	p15	10	3	Gain	P5_S1	Complete	*USP34, USP34, SNOR*		5	0
chr2:613,975-706,018	92,043	p25.3	10	100	Loss	P5_S1	Complete	*TMEM18*		1	0
chr2:62,069,341-62,132,310	62,969	p15	10	3	Gain	P5_S1	Complete	*FAM161A, CCT4, CO*		3	0
chr2:7,024,894-7,048,125	23,231	p25.2	10	0	Loss	P5_S1	Complete	*RSAD2*		1	0
chr2:71,827,856-71,912,130	84,274	p13.2	10	14	Loss	P5_S1	Complete	*DYSF*		1	0
chr2:72,102,149-72,148,034	45,885	p13.2	10	0	Loss	P5_S1	Complete			0	0
chr2:91,776,211-92,275,027	498,816	p11.1	10	100	Gain	P5_S1	Complete	*LOC654342, LSP1P4,*		6	0
chr3:10,589,880-10,968,701	378,821	p25.3	10	100	Loss	P5_S1	Complete	*ATP2B2, ATP2B2-IT2*		4	0
chr3:126,499,723-126,708,027	208,304	q21.3	10	52	Loss	P5_S1	Complete	*CHCHD6, PLXNA1*		2	0
chr3:126,735,272-127,129,291	394,019	q21.3	10	10	Loss	P5_S1	Complete	*PLXNA1, C3orf56, PR*		5	0
chr3:136,083,073-136,435,094	352,021	q22.3	10	2	Gain	P5_S1	Complete	*STAG1*		1	0
chr3:139,848,798-140,178,603	329,805	q23	10	75	Loss	P5_S1	Complete	*CLSTN2*		1	0
chr3:141,682,923-141,869,273	186,350	q23	10	35	Gain	P5_S1	Complete	*TFDP2*		1	0
chr3:169,852,066-169,962,326	110,260	q26.2	10	9	Gain	P5_S1	Complete	*PHC3, AY940074, BC*		4	0
chr3:196,471,506-196,609,779	138,273	q29	10	26	Gain	P5_S1	Complete	*PAK2, SENP5*		2	0
chr3:47,635,813-47,852,070	216,257	p21.31	10	100	Gain	P5_S1	Complete	*SNORD146, SMARCC*		3	0
chr3:47,957,692-48,162,174	204,482	p21.31	10	2	Gain	P5_S1	Complete	*MAP4*		1	0
chr3:49,212,692-49,287,915	75,223	p21.31	10	0	Gain	P5_S1	Complete	*KLHDC8B, C3orf84,*		4	0
chr3:57,443,720-57,489,708	45,988	p14.3	10	6	Gain	P5_S1	Complete	*DNAH12*		1	0
chr3:57,822,686-57,898,652	75,966	p14.3	10	2	Gain	P5_S1	Complete	*SLMAP*		1	0

chr3:8,666,243-8,684,306	18,063	p26.1	10	0	Loss	P5_S1	Complete	*SSUH2*		1	0
chr3:90,229,593-91,000,000	770,407	p11.1 - q11.1	10	36	Gain	P5_S1	Complete			0	0
chr3:93,550,578-93,708,692	158,114	q11.1	10	44	Gain	P5_S1	Complete	*PROS1, ARL13B*		2	0
chr4:140,005,987-140,181,870	175,883	q31.1	10	6	Gain	P5_S1	Complete	*ELF2*		1	0
chr4:165,689,413-165,818,717	129,304	q32.3	10	0	Gain	P5_S1	Complete	*SMIM31, LOC100505*		4	0
chr4:189,209,272-189,229,400	20,128	q35.2	10	100	Gain	P5_S1	Complete			0	0
chr4:39,412,029-40,104,018	691,989	p14	10	6	Gain	P5_S1	Complete	*MIR5591, KLB, RPL9,*		13	0
chr4:40,122,482-40,160,717	38,235	p14	10	0	Gain	P5_S1	Complete	*N4BP2*		1	0
chr4:40,446,890-40,477,827	30,937	p14	10	0	Gain	P5_S1	Complete	*RBM47*		1	0
chr4:40,577,815-40,615,792	37,977	p14	10	0	Gain	P5_S1	Complete	*RBM47*		1	0
chr4:40,645,843-40,778,178	132,335	p14	10	76	Gain	P5_S1	Complete	*NSUN7*		1	0
chr4:52,706,270-52,844,535	138,265	q12	10	95	Gain	P5_S1	Complete	*DCUN1D4*		1	0
chr4:7,373,892-7,477,027	103,135	p16.1	10	7	Loss	P5_S1	Complete	*PSAPL1, M*	hsa-mir-4	3	1
chr4:7,522,756-7,707,444	184,688	p16.1	10	4	Loss	P5_S1	Complete	*SORCS2, DQ572409*		2	0
chr4:9,994,698-10,273,286	278,588	p16.1	10	100	Loss	P5_S1	Complete	*SLC2A9, M*	hsa-mir-3	12	1
chr5:0-353,968	353,968	p15.33	10	97	Loss	P5_S1	Complete	*PLEKHG4B, LRRC14*		7	0
chr5:134,576,679-134,870,152	293,473	q31.1	10	1	Loss	P5_S1	Complete	*C5orf66, LOC100996*		10	0
chr5:138,762,651-138,857,466	94,815	q31.2	10	6	Gain	P5_S1	Complete	*DNAJC18, ECSCR, SN*		4	0
chr5:17,386,349-17,707,319	320,970	p15.1	10	100	Gain	P5_S1	Complete	*LINC02111, LOC401*		5	0
chr5:2,466,690-2,507,608	40,918	p15.33	10	4	Loss	P5_S1	Complete			0	0
chr5:32,291,261-32,370,408	79,147	p13.3	10	21	Gain	P5_S1	Complete	*MTMR12, ZFR*		2	0
chr5:37,170,227-37,304,319	134,092	p13.2	10	7	Gain	P5_S1	Complete	*C5orf42, CPLANE1, L*		4	0
chr5:5,439,347-5,491,134	51,787	p15.32	10	0	Loss	P5_S1	Complete	*KIAA0947, ICE1*		2	0
chr5:68,491,633-68,709,767	218,134	q13.2	10	65	Gain	P5_S1	Complete	*CENPH, MRPS36, CD*		7	0
chr5:708,009-947,267	239,258	p15.33	10	100	Loss	P5_S1	Complete	*ZDHHC11B, ZDHHC*		4	0
chr6:107,378,766-107,594,064	215,298	q21	10	4	Gain	P5_S1	Complete	*BEND3, PDSS2*		2	0
chr6:110,983,560-111,345,084	361,524	q21	10	8	Gain	P5_S1	Complete	*CDK19, BC047513, S*		6	0
chr6:169,600,551-169,646,640	46,089	q27	10	10	Loss	P5_S1	Complete	*LOC101929523, THB*		2	0
chr6:170,089,068-170,176,689	87,621	q27	10	3	Loss	P5_S1	Complete	*WDR27, C6orf120, PH*		7	0
chr6:21,622,782-21,699,508	76,726	p22.3	10	47	Gain	P5_S1	Complete	*CASC15, LINC00340*		2	0
chr6:40,034,126-40,380,789	346,663	p21.2	10	15	Loss	P5_S1	Complete	*LINC00951, DQ5719*		4	0
chr6:40,617,101-41,040,802	423,701	p21.1	10	1	Loss	P5_S1	Complete	*Mir_652, LOC101929*		7	0
chr6:58,603,762-58,752,089	148,327	p11.2 - p11.1	10	100	Gain	P5_S1	Complete	*Mir_598*		1	0
chr6:74,123,461-74,169,133	45,672	q13	10	4	Gain	P5_S1	Complete	*DDX43, MB21D1, CG*		3	0
chr6:74,192,220-74,382,069	189,849	q13	10	2	Gain	P5_S1	Complete	*MTO1, SNORD141A,*		7	0
chr7:0-162,665	162,665	p22.3	10	94	Loss	P5_S1	Complete	*LOC102723672, AK0*		4	0
chr7:104,932,058-105,125,422	193,364	q22.3	10	100	Gain	P5_S1	Complete	*SRPK2, PUS7*		2	0
chr7:129,700,494-129,786,610	86,116	q32.2	10	0	Gain	P5_S1	Complete	*KLHDC10, LOC1001*		2	0
chr7:131,900,275-131,985,174	84,899	q32.3	10	0	Loss	P5_S1	Complete	*LOC101928807, PLX*		2	0
chr7:140,394,575-140,558,494	163,919	q34	10	12	Gain	P5_S1	Complete	*ADCK2, NDUFB2-AS*		6	0
chr7:148,768,204-148,797,689	29,485	q36.1	10	23	Gain	P5_S1	Complete	*ZNF786*		1	0
chr7:152,009,324-152,345,483	336,159	q36.1	10	45	Gain	P5_S1	Complete	*KMT2C, FABP5P3, L*		5	0
chr7:155,285,173-155,314,636	29,463	q36.3	10	0	Loss	P5_S1	Complete	*CNPY1*		1	0
chr7:40,029,172-40,130,132	100,960	p14.1	10	100	Gain	P5_S1	Complete	*CDK13*		1	0
chr7:48,311,730-48,320,206	8,476	p12.3	10	100	Loss	P5_S1	Complete	*AX746840, ABCA13*		2	0
chr7:5,101,081-5,106,052	4,971	p22.1	10	0	Gain	P2_S2	Complete	*RBAK-RBAKDN, RBA*		2	0

chr7:55,881,932-56,035,062	153,130	p11.2	10	100	Gain	P5_S1	Complete	*SEPT14, DD413584, Z*		6	0
chr7:56,184,193-56,629,607	445,414	p11.2	10	100	Gain	P5_S1	Complete	*LOC650226, BC03625*		6	0
chr7:6,190,125-6,448,610	258,485	p22.1	10	3	Gain	P5_S1	Complete	*USP42, CYTH3, FAM2*		4	0
chr7:6,495,558-6,621,273	125,715	p22.1	10	74	Gain	P5_S1	Complete	*DAGLB, KDELR2, MI*		5	0
chr7:65,447,242-65,547,414	100,172	q11.21	10	100	Gain	P5_S1	Complete	*GUSB, ASL*		2	0
chr7:65,706,484-65,751,273	44,789	q11.21	10	100	Gain	P5_S1	Complete	*TPST1*		1	0
chr7:73,120,434-73,243,452	123,018	q11.23	10	0	Gain	P5_S1	Complete	*STX1A, MII*	hsa-mir-42	7	1
chr7:73,642,744-73,965,401	322,657	q11.23	10	5	Gain	P5_S1	Complete	*LAT2, RFC2, CLIP2, C*		4	0
chr7:75,216,932-75,400,968	184,036	q11.23	10	59	Gain	P5_S1	Complete	*HIP1, CCL26*		2	0
chr7:75,861,603-75,952,263	90,660	q11.23	10	20	Gain	P5_S1	Complete	*SRRM3, BC063788, H*		4	0
chr8:0-196,605	196,605	p23.3	10	95	Loss	P5_S1	Complete	*OR4F21, RPL23AP53*		3	0
chr8:1,028,643-1,126,798	98,155	p23.3	10	7	Loss	P5_S1	Complete	*ERICH1-AS1, DLGAP*		2	0
chr8:10,396,009-10,403,553	7,544	p23.1	10	0	Loss	P5_S1	Complete	*AK307207, PRSS55*		2	0
chr8:133,882,045-133,899,304	17,259	q24.22	10	0	Loss	P5_S1	Complete	*TG*		1	0
chr8:133,930,820-133,958,777	27,957	q24.22	10	0	Loss	P5_S1	Complete	*TG*		1	0
chr8:134,013,871-134,034,462	20,591	q24.22	10	57	Loss	P5_S1	Complete	*TG*		1	0
chr8:140,281,276-140,300,478	19,202	q24.3	10	0	Loss	P5_S1	Complete			0	0
chr8:140,511,304-140,814,769	303,465	q24.3	10	5	Loss	P5_S1	Complete	*KCNK9, TRAPPC9*		2	0
chr8:142,950,195-143,248,497	298,302	q24.3	10	24	Loss	P5_S1	Complete	*MIR4539*		1	0
chr8:143,305,370-143,467,380	162,010	q24.3	10	4	Loss	P5_S1	Complete	*TSNARE1*		1	0
chr8:143,722,202-143,807,273	85,071	q24.3	10	100	Loss	P5_S1	Complete	*JH8, JRK, PSCA, AX7*		7	0
chr8:306,153-427,526	121,373	p23.3	10	100	Loss	P5_S1	Complete	*FAM87A, FBXO25, AI*		3	0
chr8:528,232-576,712	48,480	p23.3	10	100	Loss	P5_S1	Complete	*ERICH1*		1	0
chr8:67,616,270-67,799,681	183,411	q13.1	10	6	Gain	P5_S1	Complete	*C8orf44-SGK3, PTTG*		4	0
chr8:95,733,740-95,780,710	46,970	q22.1	10	0	Gain	P5_S1	Complete	*DPY19L4*		1	0
chr8:98,666,152-98,760,861	94,709	q22.1	10	78	Gain	P5_S1	Complete	*MTDH*		1	0
chr9:114,461,681-114,572,107	110,426	q31.3	10	0	Gain	P5_S1	Complete	*SHOC1, C9orf84*		2	0
chr9:114,978,726-115,032,852	54,126	q32	10	1	Gain	P5_S1	Complete	*MIR3134, PTBP3*		2	0
chr9:127,697,256-127,945,280	248,024	q33.3	10	1	Gain	P5_S1	Complete	*GOLGA1, SCAI, AX74*		4	0
chr9:132,692,954-132,789,900	96,946	q34.11	10	100	Gain	P5_S1	Complete	*FNBP1*		1	0
chr9:137,595,893-137,827,039	231,146	q34.3	10	100	Loss	P5_S1	Complete	*COL5A1, LOC101448*		11	0
chr9:19,175,332-19,297,919	122,587	p22.1	10	11	Gain	P5_S1	Complete	*DQ572382, DENND4*		2	0
chr9:33,998,845-34,091,050	92,205	p13.3	10	10	Gain	P5_S1	Complete	*UBAP2, DCAF12*		2	0
chr9:36,341,059-36,484,826	143,767	p13.2	10	3	Gain	P5_S1	Complete	*RNF38*		1	0
chr9:65,629,772-69,238,239	3,608,467	q12 - q21.11	10	100	Gain	P5_S1	Complete	*DQ600787,*	hsa-mir-11	39	1
chr9:9,471,004-9,504,185	33,181	p23	10	0	Loss	P1_S3	Complete	*PTPRD*		1	0
chr10:104,854,616-104,936,970	82,354	q24.32 - q24.33	10	2	Gain	P5_S1	Complete	*NT5C2*		1	0
chr10:11,951,415-12,128,507	177,092	p14	10	35	Gain	P5_S1	Complete	*AK124930, UPF2, DH*		3	0
chr10:119,795,943-119,851,186	55,243	q26.11	10	0	Loss	P5_S1	Complete	*RAB11FIP2, CASC2*		2	0
chr10:12,164,048-12,398,553	234,505	p14 - p13	10	60	Gain	P5_S1	Complete	*DHTKD1, MIR548AK*		6	0
chr10:124,306,511-124,392,089	85,578	q26.13	10	100	Loss	P5_S1	Complete	*DMBT1*		1	0
chr10:130,457,915-130,580,794	122,879	q26.2	10	0	Loss	P5_S1	Complete			0	0
chr10:130,931,384-131,237,029	305,645	q26.3	10	0	Loss	P5_S1	Complete			0	0
chr10:132,490,370-132,534,610	44,240	q26.3	10	100	Loss	P5_S1	Complete			0	0
chr10:134,400,848-134,767,148	366,300	q26.3	10	56	Loss	P5_S1	Complete	*INPP5A, NKX6-2, TT*		5	0
chr10:135,301,554-135,403,443	101,889	q26.3	10	100	Loss	P5_S1	Complete	*CYP2E1, SYCE1, SPR*		3	0

chr10:18,673,662-18,688,735	15,073	p12.32	10	0	Gain	P5_S1	Complete	*CACNB2*		1	0
chr10:35,067,373-35,248,828	181,455	p11.21	10	100	Gain	P5_S1	Complete	*PARD3, PARD3-AS1*		2	0
chr10:44,374,378-44,445,303	70,925	q11.21	10	1	Loss	P5_S1	Complete	*LINC00840, LINC008*		2	0
chr10:49,892,016-50,081,894	189,878	q11.22 - q11.23	10	3	Loss	P5_S1	Complete	*WDFY4, WDFY4*		2	0
chr10:50,156,341-50,356,678	200,337	q11.23	10	5	Loss	P5_S1	Complete	*WDFY4, MI*	hsa-mir-4	5	1
chr10:50,468,777-50,622,877	154,100	q11.23	10	0	Loss	P5_S1	Complete	*C10orf71-AS1, C10or*		3	0
chr10:67,726,868-67,761,351	34,483	q21.3	10	0	Loss	P1_S1	Complete	*CTNNA3*		1	0
chr10:69,725,768-69,924,000	198,232	q21.3	10	10	Gain	P5_S1	Complete	*HERC4, POU5F1P5,*		3	0
chr10:69,970,088-70,593,778	623,690	q21.3	10	7	Gain	P5_S1	Complete	*MYPN, ATC*	hsa-mir-1	11	1
chr10:72,600,655-72,732,520	131,865	q22.1	10	20	Loss	P5_S1	Complete	*SGPL1, PCBD1, LOC*		3	0
chr10:73,051,506-73,115,860	64,354	q22.1	10	1	Loss	P5_S1	Complete	*UNC5B, SLC29A3*		2	0
chr10:74,184,444-74,655,781	471,337	q22.1	10	0	Gain	P5_S1	Complete	*MIR1256, MICU1, MI*		5	0
chr10:75,081,598-75,137,327	55,729	q22.2	10	14	Gain	P5_S1	Complete	*TTC18, CFAP70, ANX*		3	0
chr10:80,209,141-80,856,555	647,414	q22.3	10	2	Loss	P5_S1	Complete	*LINC00856, AX74798*		4	0
chr10:87,752,909-87,816,738	63,829	q23.1	10	0	Loss	P5_S1	Complete	*GRID1*		1	0
chr10:93,917,386-94,477,010	559,624	q23.32 - q23.33	10	5	Gain	P5_S1	Complete	*CPEB3, CPEB3_riboz*		8	0
chr11:11,338,061-11,419,657	81,596	p15.3	10	55	Loss	P5_S1	Complete	*CSNK2A3, GALNT18*		2	0
chr11:11,452,559-11,515,989	63,430	p15.3	10	0	Loss	P5_S1	Complete	*GALNT18*		1	0
chr11:115,915,104-115,964,320	49,216	q23.3	10	0	Loss	P5_S1	Complete			0	0
chr11:126,527,900-126,808,914	281,014	q24.2	10	0	Loss	P5_S1	Complete	*LOC101929427, DJ03*		3	0
chr11:126,877,184-126,891,762	14,578	q24.2	10	0	Loss	P5_S1	Complete			0	0
chr11:131,656,760-131,669,842	13,082	q25	10	100	Loss	P5_S1	Complete	*NTM*		1	0
chr11:133,063,692-133,088,235	24,543	q25	10	100	Loss	P5_S1	Complete	*OPCML*		1	0
chr11:133,367,803-133,431,131	63,328	q25	10	0	Loss	P5_S1	Complete	*OPCML*		1	0
chr11:134,412,787-134,474,259	61,472	q25	10	100	Loss	P5_S1	Complete			0	0
chr11:134,626,528-134,873,998	247,470	q25	10	89	Loss	P5_S1	Complete	*AK125040, LOC72930*		4	0
chr11:19,539,147-19,619,812	80,665	p15.1	10	0	Loss	P5_S1	Complete	*NAV2-AS4, MIR4486,*		4	0
chr11:2,335,352-2,427,906	92,554	p15.5	10	100	Loss	P5_S1	Complete	*TSPAN32, CD81-AS1,*		6	0
chr11:2,443,861-2,842,050	398,189	p15.5 - p15.4	10	43	Loss	P5_S1	Complete	*TRPM5, KCNQ1, KCN*		3	0
chr11:45,249,023-45,531,664	282,641	p11.2	10	2	Loss	P5_S1	Complete	*PRDM11, SYT13, TRN*		6	0
chr11:47,610,876-47,689,228	78,352	p11.2	10	37	Gain	P5_S1	Complete	*C1QTNF4, MTCH2, A*		3	0
chr11:47,812,262-48,062,180	249,918	p11.2	10	3	Gain	P5_S1	Complete	*NUP160, Y_RNA, PTF*		3	0
chr11:58,875,056-58,930,065	55,009	q12.1	10	100	Loss	P5_S1	Complete	*FAM111B, FAM111A-*		4	0
chr11:78,512,928-78,625,515	112,587	q14.1	10	0	Loss	P5_S1	Complete	*TENM4, TENM4*		2	0
chr11:78,724,347-78,771,833	47,486	q14.1	10	0	Loss	P5_S1	Complete	*TENM4, TENM4*		2	0
chr11:79,125,002-79,187,735	62,733	q14.1	10	0	Loss	P5_S1	Complete	*TENM4, MIR5579*		2	0
chr11:9,304,585-9,682,443	377,858	p15.4	10	21	Gain	P5_S1	Complete	*TMEM41B, IPO7, SN*		9	0
chr12:110,559,495-110,807,789	248,294	q24.11	10	0	Gain	P5_S1	Complete	*IFT81, JA611269, AT*		3	0
chr12:110,940,880-111,046,496	105,616	q24.11	10	2	Gain	P5_S1	Complete	*BC064974, RAD9B, P*		3	0
chr12:111,141,120-111,176,353	35,233	q24.11	10	28	Gain	P5_S1	Complete	*PPP1CC*		1	0
chr12:111,933,303-111,954,884	21,581	q24.12	10	0	Gain	P5_S1	Complete	*ATXN2*		1	0
chr12:112,238,561-112,301,305	62,744	q24.12 - q24.13	10	100	Gain	P5_S1	Complete	*ALDH2, MAPKAPK5-*		3	0
chr12:118,535,619-118,851,609	315,990	q24.23	10	2	Gain	P5_S1	Complete	*VSIG10, PEBP1, TAO*		4	0
chr12:122,303,297-122,395,023	91,726	q24.31	10	2	Gain	P5_S1	Complete	*HPD, PSMD9, WDR6*		3	0
chr12:122,631,058-122,653,940	22,882	q24.31	10	71	Gain	P5_S1	Complete	*MLXIP, LRRC43*		2	0
chr12:122,959,294-123,118,754	159,460	q24.31	10	0	Gain	P5_S1	Complete	*ZCCHC8, SNORA9, R*		5	0

chr12:130,803,001-130,880,190	77,189	q24.33	10	100	Loss	P5_S1	Complete	*PIWIL1*		1	0
chr12:131,630,343-131,704,894	74,551	q24.33	10	100	Loss	P5_S1	Complete	*LINC01257, LOC1164*		2	0
chr12:131,746,199-131,964,016	217,817	q24.33	10	100	Loss	P5_S1	Complete	*BC042649, LINC0241*		4	0
chr12:133,779,171-133,851,895	72,724	q24.33	10	1	Gain	P2_S2	Complete	*ZNF268, ANHX*		2	0
chr12:2,250,067-2,598,551	348,484	p13.33	10	20	Loss	P5_S1	Complete	*CACNA1C-AS4, CACI*		3	0
chr12:31,608,536-32,126,315	517,779	p11.21	10	38	Gain	P5_S1	Complete	*DENND5B, U5, DENI*		10	0
chr12:32,144,079-32,370,401	226,322	p11.21	10	42	Gain	P5_S1	Complete	*KIAA1551, RESF1, RI*		4	0
chr12:32,534,123-32,778,351	244,228	p11.21	10	69	Gain	P5_S1	Complete	*BICD1, FGD4*		2	0
chr12:34,554,847-34,756,688	201,841	p11.1	10	100	Gain	P2_S2	Complete			0	0
chr12:50,748,220-50,868,515	120,295	q13.12	10	0	Gain	P5_S1	Complete	*FAM186A, LARP4, SN*		3	0
chr12:51,189,659-51,345,919	156,260	q13.12	10	1	Gain	P5_S1	Complete	*ATF1, TMPRSS12, MI*		3	0
chr12:51,470,613-51,617,980	147,367	q13.12 - q13.13	10	4	Gain	P5_S1	Complete	*CSRNP2, LOC494150*		4	0
chr12:52,706,916-53,077,045	370,129	q13.13	10	41	Loss	P5_S1	Complete	*KRT83, KRT85, KRT8*		16	0
chr12:53,093,307-53,098,697	5,390	q13.13	10	100	Loss	P5_S1	Complete	*KRT77*		1	0
chr12:53,850,264-53,873,083	22,819	q13.13	10	5	Gain	P5_S1	Complete	*PCBP2, PCBP2-OT1*		2	0
chr12:56,874,648-56,944,886	70,238	q13.3	10	12	Gain	P5_S1	Complete	*GLS2, BC059370, SN*		4	0
chr12:7,871,634-7,960,432	88,798	p13.31	10	100	Gain	P5_S1	Complete	*CLEC4C, NANOGNB,*		3	0
chr12:95,401,549-95,453,891	52,342	q22	10	6	Gain	P5_S1	Complete	*NR2C1*		1	0
chr12:95,694,298-95,781,377	87,079	q22	10	0	Gain	P5_S1	Complete	*VEZT, MIR*	hsa-mir-3	3	1
chr12:95,945,553-96,001,537	55,984	q22	10	0	Gain	P5_S1	Complete			0	0
chr13:86,735,102-86,946,800	211,698	q31.1	10	3	Gain	P5_S1	Complete			0	0
chr14:101,507,650-101,660,126	152,476	q32.31	10	17	Loss	P5_S1	Complete	*MIR300, M*	hsa-mir-3	29	24
chr14:31,556,385-31,590,643	34,258	q12	10	10	Gain	P5_S1	Complete	*AP4S1, HECTD1*		2	0
chr14:31,627,365-31,768,403	141,038	q12	10	1	Gain	P5_S1	Complete	*HECTD1, HEATR5A*		2	0
chr14:34,882,747-35,716,707	833,960	q13.1 - q13.2	10	40	Gain	P5_S1	Complete	*SPTSSA, EAPP, SNX6*		13	0
chr14:50,100,462-50,266,644	166,182	q21.3	10	100	Gain	P5_S1	Complete	*DNAAF2, POLE2, NE*		5	0
chr14:64,066,261-64,407,219	340,958	q23.2	10	82	Gain	P5_S1	Complete	*WDR89, JA429503, S*		4	0
chr14:73,611,006-73,683,789	72,783	q24.2	10	0	Gain	P5_S1	Complete	*PSEN1, BC016143*		2	0
chr14:74,258,255-74,296,015	37,760	q24.3	10	0	Gain	P5_S1	Complete	*LOC100506476, BC0*		3	0
chr14:74,454,095-74,495,178	41,083	q24.3	10	100	Gain	P5_S1	Complete	*ENTPD5, BBOF1, CC*		3	0
chr15:20,071,673-20,771,825	700,152	q11.1 - q11.2	10	100	Gain	P5_S1	Complete	*DQ576041, DQ57147*		14	0
chr15:41,283,831-41,749,261	465,430	q15.1	10	3	Gain	P5_S1	Complete	*INO80, EXD1, CHP1,*		9	0
chr15:44,396,419-44,822,017	425,598	q15.3 - q21.1	10	1	Gain	P5_S1	Complete	*FRMD5, CASC4, CTD*		3	0
chr15:50,700,474-51,143,697	443,223	q21.2	10	2	Gain	P5_S1	Complete	*USP8, AX746640, USI*		7	0
chr15:64,708,593-64,948,306	239,713	q22.31	10	6	Gain	P5_S1	Complete	*TRIP4, ZNF609*		2	0
chr15:70,127,025-70,238,468	111,443	q23	10	0	Loss	P5_S1	Complete	*LINC00593*		1	0
chr15:70,345,623-70,486,961	141,338	q23	10	29	Loss	P5_S1	Complete	*TLE3, MIR*	hsa-mir-6	3	1
chr16:11,862,720-11,931,009	68,289	p13.13	10	3	Gain	P5_S1	Complete	*ZC3H7A, BCAR4, RSI*		3	0
chr16:11,952,968-11,989,997	37,029	p13.13	10	0	Gain	P5_S1	Complete	*GSPT1*		1	0
chr16:68,946,640-69,151,586	204,946	q22.1	10	1	Gain	P5_S1	Complete	*TANGO6, HAS3*		2	0
chr16:69,286,861-69,345,759	58,898	q22.1	10	0	Gain	P5_S1	Complete	*SNTB2, VPS4A*		2	0
chr16:69,497,097-69,743,069	245,972	q22.1	10	1	Gain	P5_S1	Complete	*CYB5B, MI*	hsa-mir-1	4	1
chr16:74,514,554-74,684,513	169,959	q23.1	10	52	Gain	P5_S1	Complete	*GLG1, RFWD3*		2	0
chr17:1,022,288-1,161,130	138,842	p13.3	10	22	Gain	P5_S1	Complete	*ABR*		1	0
chr17:1,221,813-1,300,039	78,226	p13.3	10	7	Gain	P5_S1	Complete	*YWHAE*		1	0
chr17:1,503,214-1,538,680	35,466	p13.3	10	34	Gain	P5_S1	Complete	*SLC43A2, SCARF1*		2	0

chr17:1,633,844-1,637,634	3,790	p13.3	10	100	Gain	P5_S1	Complete	*WDR81*		1	0
chr17:2,461,706-2,561,661	99,955	p13.3	10	28	Gain	P5_S1	Complete	*DD413682, PAFAH1E*		2	0
chr17:21,833,289-22,034,501	201,212	p11.2	10	100	Gain	P5_S1	Complete	*FLJ36000, TRNA, JB1*		5	0
chr17:27,713,999-27,795,204	81,205	q11.2	10	4	Gain	P5_S1	Complete	*MIR4523, TAOK1*		2	0
chr17:29,108,685-29,232,273	123,588	q11.2	10	100	Gain	P5_S1	Complete	*SUZ12P1, CRLF3, AT*		4	0
chr17:30,482,658-30,581,133	98,475	q11.2	10	0	Gain	P5_S1	Complete	*RHOT1*		1	0
chr17:41,218,772-41,244,340	25,568	q21.31	10	2	Gain	P5_S1	Complete	*BRCA1*		1	0
chr17:41,245,666-41,342,263	96,597	q21.31	10	5	Gain	P5_S1	Complete	*BRCA1, NBR2, AK093*		6	0
chr17:56,800,662-57,283,762	483,100	q22	10	6	Gain	P5_S1	Complete	*RAD51C, P*	hsa-mir-4	8	2
chr17:57,293,276-57,938,064	644,788	q22 - q23.1	10	4	Gain	P5_S1	Complete	*GDPD1, M*	hsa-mir-2	11	1
chr17:58,764,043-58,977,259	213,216	q23.2	10	13	Gain	P5_S1	Complete	*BCAS3*		1	0
chr17:60,064,999-60,484,646	419,647	q23.2	10	43	Gain	P5_S1	Complete	*MED13, Mir_652, TB*		4	0
chr17:62,388,750-62,489,030	100,280	q23.3	10	2	Gain	P5_S1	Complete	*PECAM1, MILR1, PO*		3	0
chr17:62,537,255-62,744,994	207,739	q23.3 - q24.1	10	21	Gain	P5_S1	Complete	*SMURF2*		1	0
chr17:65,313,026-65,510,858	197,832	q24.2	10	66	Gain	P5_S1	Complete	*PSMD12, M*	hsa-mir-5	4	1
chr17:65,577,004-65,955,751	378,747	q24.2	10	33	Gain	P5_S1	Complete	*PITPNC1, NOL11, SN*		4	0
chr18:18,570,235-19,023,053	452,818	q11.1 - q11.2	10	1	Gain	P5_S1	Complete	*ROCK1, GREB1L*		2	0
chr18:19,110,947-19,487,345	376,398	q11.2	10	0	Gain	P5_S1	Complete	*ESCO1, SN*	hsa-mir-3	9	3
chr18:35,040,189-35,079,040	38,851	q12.2	10	0	Loss	P5_S1	Complete	*CELF4, SNORA111*		2	0
chr18:35,211,386-35,263,506	52,120	q12.2	10	0	Loss	P5_S1	Complete	*MIR4318*	hsa-mir-4	1	1
chr18:72,821,512-72,866,568	45,056	q22.3	10	100	Loss	P5_S1	Complete			0	0
chr18:73,022,307-73,049,243	26,936	q22.3	10	100	Loss	P5_S1	Complete			0	0
chr18:73,137,090-73,273,404	136,314	q23	10	100	Loss	P5_S1	Complete	*SMIM21*		1	0
chr19:10,420,979-10,444,027	23,048	p13.2	10	0	Gain	P5_S1	Complete	*FDX1L, FDX2, BC01*		4	0
chr19:10,601,111-10,644,656	43,545	p13.2	10	0	Gain	P5_S1	Complete	*KEAP1, S1PR5*		2	0
chr19:11,298,308-11,304,491	6,183	p13.2	10	100	Gain	P5_S1	Complete	*KANK2*		1	0
chr19:11,598,129-11,618,728	20,599	p13.2	10	0	Gain	P5_S1	Complete	*ZNF653, MIR7974, E*		3	0
chr19:11,729,871-11,889,513	159,642	p13.2	10	11	Gain	P5_S1	Complete	*ZNF627, ZNF833P, Z*		4	0
chr19:12,270,177-12,428,600	158,423	p13.2	10	35	Gain	P5_S1	Complete	*ZNF136, AX721123, L*		6	0
chr19:12,542,387-12,691,506	149,119	p13.2	10	100	Gain	P5_S1	Complete	*ZNF443, ZNF709, ZN*		4	0
chr19:13,679,542-13,879,427	199,885	p13.2	10	3	Gain	P5_S1	Complete	*CCDC130, MRI1*		2	0
chr19:14,542,081-14,573,953	31,872	p13.12	10	0	Gain	P5_S1	Complete	*PKN1*		1	0
chr19:17,361,212-17,370,439	9,227	p13.11	10	100	Gain	P5_S1	Complete	*USHBP1*		1	0
chr19:17,615,511-17,624,026	8,515	p13.11	10	0	Gain	P5_S1	Complete	*SLC27A1, LOC100507*		3	0
chr19:21,124,930-21,141,796	16,866	p12	10	18	Gain	P2_S2	Complete	*ZNF85*		1	0
chr19:21,224,667-21,270,879	46,212	p12	10	100	Gain	P5_S1	Complete	*ZNF430, ZNF714*		2	0
chr19:21,719,197-21,730,430	11,233	p12	10	100	Gain	P1_S1	Complete	*ZNF429*		1	0
chr19:24,378,791-24,518,609	139,818	p12 - p11	10	100	Gain	P1_S1	Complete			0	0
chr19:36,643,058-37,449,414	806,356	q13.12	10	27	Gain	P5_S1	Complete	*COX7A1, ZNF565, ZN*		28	0
chr19:37,591,022-37,738,433	147,411	q13.12	10	15	Gain	P5_S1	Complete	*ZNF420, ZNF585A, Z*		4	0
chr19:39,234,744-39,287,664	52,920	q13.2	10	13	Gain	P5_S1	Complete	*CAPN12, LGALS7, L*		3	0
chr19:4,502,152-4,510,705	8,553	p13.3	10	8	Gain	P5_S1	Complete	*HDGFL2, HDGFRP2,*		3	0
chr19:4,594,523-4,690,297	95,774	p13.3	10	99	Gain	P5_S1	Complete	*TNFAIP8L1, C19orf1*		6	0
chr19:40,520,854-40,541,629	20,775	q13.2	10	1	Gain	P2_S2	Complete	*ZNF546, ZNF780B*		2	0
chr19:44,350,723-44,352,673	1,950	q13.31	10	0	Gain	P2_S2	Complete	*ZNF283*		1	0
chr19:46,029,706-46,078,250	48,544	q13.32	10	4	Gain	P5_S1	Complete	*VASP, OPA3*		2	0

chr19:48,285,243-48,502,016	216,773	q13.33	10	97	Gain	P5_S1	Complete	*SELENOW, SEPW1, A*		20	0
chr19:49,116,167-49,192,874	76,707	q13.33	10	100	Gain	P5_S1	Complete	*FAM83E, RPL18, SPH*		7	0
chr19:49,254,326-49,308,887	54,561	q13.33	10	100	Gain	P5_S1	Complete	*FUT1, FGF21, BCAT2*		3	0
chr19:52,537,268-52,538,142	874	q13.41	10	100	Gain	P1_S3	Complete	*ZNF432*		1	0
chr19:53,056,475-53,066,259	9,784	q13.41	10	100	Gain	P1_S1	Complete	*ZNF808*		1	0
chr19:54,193,671-54,292,736	99,065	q13.42	10	100	Gain	P5_S1	Complete	*MIR520A, M*	hsa-mir-52	39	38
chr19:6,942,952-7,125,438	182,486	p13.2	10	100	Gain	P5_S1	Complete	*ADGRE4P, EMR4P, F*		10	0
chr19:7,189,177-7,497,290	308,113	p13.2	10	100	Gain	P5_S1	Complete	*INSR, ARHGEF18*		2	0
chr19:7,708,136-7,734,029	25,893	p13.2	10	100	Gain	P5_S1	Complete	*STXBP2, RETN*		2	0
chr19:746,333-757,252	10,919	p13.3	10	100	Gain	P5_S1	Complete	*PALM, MISP*		2	0
chr19:8,201,078-8,375,392	174,314	p13.2	10	35	Gain	P5_S1	Complete	*FBN3, CERS4, CD320*		4	0
chr19:8,634,354-8,769,167	134,813	p13.2	10	8	Gain	P5_S1	Complete	*MYO1F, ADAMTS10*		2	0
chr19:8,924,646-8,953,859	29,213	p13.2	10	100	Gain	P5_S1	Complete	*ZNF558, MBD3L1*		2	0
chr19:868,758-890,414	21,656	p13.3	10	100	Gain	P5_S1	Complete	*MED16, MED16*		2	0
chr19:9,098,988-9,202,005	103,017	p13.2	10	0	Gain	P5_S1	Complete	*TRNA_Pseudo*		1	0
chr19:9,868,324-9,869,295	971	p13.2	10	100	Gain	P1_S1	Complete	*ZNF846*		1	0
chr20:1,456,967-1,469,429	12,462	p13	10	0	Loss	P5_S1	Complete	*SIRPB2*		1	0
chr20:19,361,675-19,419,518	57,843	p11.23	10	0	Loss	P5_S1	Complete	*SLC24A3*		1	0
chr20:22,941,973-23,013,280	71,307	p11.21	10	10	Loss	P5_S1	Complete	*BC045663*		1	0
chr20:23,026,383-23,095,191	68,808	p11.21	10	96	Loss	P5_S1	Complete	*AX747264, THBD, CD*		3	0
chr20:23,377,766-23,549,451	171,685	p11.21	10	0	Loss	P5_S1	Complete	*NAPB, CSTL1, CST11*		6	0
chr20:24,551,970-24,965,767	413,797	p11.21	10	17	Loss	P5_S1	Complete	*SYNDIG1, CST7, APM*		3	0
chr20:26,086,933-26,198,853	111,920	p11.1	10	100	Gain	P5_S1	Complete	*NCOR1P1,*	hsa-mir-66	4	1
chr20:34,288,695-34,484,941	196,246	q11.22 - q11.23	10	3	Gain	P5_S1	Complete	*ROMO1, RBM39, PHF*		3	0
chr20:35,443,730-35,507,557	63,827	q11.23	10	1	Gain	P5_S1	Complete	*SOGA1, TLDC2*		2	0
chr20:35,661,247-35,680,318	19,071	q11.23	10	23	Gain	P5_S1	Complete	*RBL1*		1	0
chr20:50,419,580-50,679,202	259,622	q13.2	10	5	Gain	P5_S1	Complete	*LINC01429*		1	0
chr20:56,137,880-56,211,556	73,676	q13.31	10	55	Loss	P5_S1	Complete	*PCK1, ZBP1*		2	0
chr20:56,274,892-56,360,068	85,176	q13.31	10	100	Loss	P5_S1	Complete	*PMEPA1, AK056098,*		3	0
chr20:56,466,703-56,606,936	140,233	q13.31 - q13.32	10	100	Loss	P5_S1	Complete	*MIR4532, LINC01742*		2	0
chr20:58,407,900-58,648,173	240,273	q13.33	10	41	Loss	P5_S1	Complete	*PHACTR3, SYCP2, PI*		6	0
chr20:58,452,574-58,464,462	11,888	q13.33	10	100	Gain	P2_S1	Complete	*SYCP2*		1	0
chr20:59,753,948-60,221,920	467,972	q13.33	10	26	Loss	P5_S1	Complete	*CDH4*		1	0
chr20:60,316,417-60,418,246	101,829	q13.33	10	2	Loss	P5_S1	Complete	*CDH4*		1	0
chr21:46,032,288-46,078,059	45,771	q22.3	10	100	Loss	P5_S1	Complete	*TSPEAR, KRTAP10-8,*		7	0
chrX:100,363,067-100,414,491	51,424	q22.1	10	13	Gain	P5_S1	Complete	*CENPI*		1	0
chrX:101,909,439-101,910,711	1,272	q22.1	10	0	Loss	P5_S1	Complete	*GPRASP1, ARMCX5-(*		2	0
chrX:114,816,926-115,019,324	202,398	q23	10	96	Gain	P5_S1	Complete	*PLS3, DANT1, DANT2*		3	0
chrX:122,796,182-123,212,503	416,321	q25	10	5	Gain	P5_S1	Complete	*THOC2, XIAP, LOC10*		4	0
chrX:23,786,007-24,255,687	469,680	p22.11	10	59	Gain	P5_S1	Complete	*Y_RNA, AK310094, SA*		10	0
chrX:32,382,097-32,614,692	232,595	p21.1	10	23	AI;Loss	P1_S1;P1_S1	Complete;Compl	*DMD, JA783589, JA7*		15	0
chrX:38,984,318-39,511,591	527,273	p11.4	10	0	Loss	P5_S1	Complete	*LINC01281, LOC2864*		3	0
chrX:40,920,989-41,284,003	363,014	p11.4	10	46	Gain	P5_S1	Complete	*USP9X, LINC02601, 5*		4	0
chrX:44,247,637-44,822,479	574,842	p11.3	10	16	Gain	P5_S1	Complete	*FUNDC1, DUSP21, K*		3	0
chrX:53,828,732-54,442,202	613,470	p11.22	10	100	Gain	P5_S1	Complete	*PHF8, FAM120C, WN*		3	0
chrX:6,511,946-8,184,869	1,672,923	p22.31	10	49	AI	P5_S1	Complete	*PUDP, HD*	hsa-mir-65	8	1

chrX:688,166-2,300,717	1,612,551	p22.33	10	38	Gain	P5_S1	Complete	*DQ576039, CSF2RA,*	13	0

Supplementary Table S2D. CNVs in 10 cranial meningiomas, excluding CNVs in chromosome 22. Chromosomal coordinates of the region (hg19); the region length in bp; the chromosomal cytoband; frequency of the region occurrence ir the tumor samples; percent of the region's overlap with a known CNV; type of the variation (gain, loss, or allelic imbalance); sample ID; type of the participation of an individual CNV in the region (complete or partial); gene symbols; microR symbols; number of genes in the region; and the number of microRNA genes found in the region are shown from left to right.

Chromosomal region	Region length (bp)	Cytoband	Frequency of the CNV occurrence in samples (%)	Overlap with known CNVs (%)	Type of CNV	Sample ID	Participation	Gene symbols	miRNAs symbols	Gene count	miRNAs count
chr7:38,316,293-38,342,505	26,212	p14.1	100	100	Gain;Gain;Gain;Gai	P1_C1;P1_C2;P1_C3;P1_C4;P1_	Complete;Complete;Con	TRGC2, TARP, TCRGC2, TCR		5	0
chr14:22,718,405-22,953,551	235,146	q11.2	100	100	Gain;Gain;Gain;Gai	P1_C1;P1_C2;P1_C3;P1_C4;P1_	Complete;Complete;Con	AV2S1A1, TCRA, TCR-alpha,		25	0
chr7:142,378,751-142,457,152	78,401	q34	80	100	Gain;Gain;Gain;Gai	P1_C1;P1_C2;P1_C3;P1_C4;P1_	Complete;Complete;Con	TCRBV5S1A1T, TCRBV11S1		10	0
chr7:142,459,821-142,460,843	1,022	q34	60	100	Gain;Gain;Gain;Gai	P1_C2;P1_C4;P1_C5;P3_C1;P3_	Complete;Complete;Con	TCRBV5S1A1T, TCRBV2S1,		5	0
chr7:61,747,794-62,359,388	611,594	q11.21	60	100	Gain;Gain;Gain;Gai	P1_C1;P1_C2;P1_C3;P1_C5;P3_	Complete;Complete;Complete;Complete;Complete;Com			0	0
chr12:37,961,768-38,255,277	293,509	q11 - q12	60	100	Gain;Gain;Gain;Gai	P1_C1;P1_C2;P1_C3;P1_C5;P3_	Complete;Complete;Complete;Complete;Complete;Com			0	0
chr10:47,593,880-47,694,995	101,115	q11.22	50	100	AI;AI;AI;AI;AI	P1_C1;P1_C2;P1_C3;P1_C4;P1_	Complete;Complete;Con	ANTXRLP1, AK057316, ANT		3	0
chr11:50,472,623-50,697,498	224,875	p11.12	50	100	Gain;Gain;Gain;Gai	P1_C1;P1_C3;P1_C5;P6_C1;P7_	Complete;Complete;Complete;Complete;Complete			0	0
chr7:48,315,976-48,318,967	2,991	p12.3	40	100	Loss;Loss;Loss;Los	P1_C1;P1_C3;P6_C1;P7_C1	Complete;Complete;Con	AX746840, ABCA13		2	0
chr1:30,640,464-30,657,099	16,635	p35.2	30	100	Loss;Loss;Loss	P1_C3;P6_C1;P7_C1	Complete;Complete;Complete			0	0
chr8:43,460,491-43,674,370	213,879	p11.1	30	100	Gain;Gain;Gain	P1_C1;P1_C3;P3_C1	Complete;Complete;Complete			0	0
chr1:10,977,709-11,010,423	32,714	p36.22	20	100	Loss;Loss	P1_C3;P6_C1	Complete;Complete	C1orf127		1	0
chr1:12,779,573-12,786,171	6,598	p36.21	20	100	Loss;Loss	P1_C3;P6_C1	Complete;Complete	AADACL3		1	0
chr1:12,812,129-12,837,651	25,522	p36.21	20	100	Loss;Loss	P1_C3;P6_C1	Complete;Complete	C1orf158, PRAMEF12		2	0
chr4:366,590-438,014	71,424	p16.3	20	100	Gain;Gain	P1_C3;P3_C1	Complete;Complete	ZNF141, ABCA11P, ZNF721		3	0
chr19:12,454,948-12,461,889	6,941	p13.2	20	100	Gain;Gain	P1_C3;P6_C1	Complete;Complete	ZNF442		1	0
chr19:21,716,320-21,720,496	4,176	p12	20	100	Gain;Gain	P1_C2;P1_C3	Complete;Complete	ZNF429		1	0
chr19:27,862,694-28,135,850	273,156	q11	20	100	Gain;Gain	P1_C3;P1_C5	Complete;Complete	AK075337		1	0
chr19:52,537,268-52,538,142	874	q13.41	20	100	Gain;Gain	P1_C2;P1_C3	Complete;Complete	ZNF432		1	0
chr19:53,056,614-53,066,259	9,645	q13.41	20	100	Gain;Gain	P1_C1;P1_C3	Complete;Complete	ZNF808		1	0
chrX:24,073,875-24,193,755	119,880	p22.11	20	100	Gain;Gain	P6_C1;P7_C1	Complete;Complete	EIF2S3, ZFX-AS1, ZFX		3	0
chrX:32,476,215-32,520,932	44,717	p21.1	20	91	Loss;Loss	P1_C4;P1_C5	Complete;Complete	DMD, JA783415, JA783560, J		5	0
chr1:34,657,642-34,667,156	9,514	p34.3	20	75	Loss;Loss	P1_C3;P6_C1	Complete;Complete	C1orf94		1	0
chr1:16,069,568-16,085,070	15,502	p36.21	20	74	Loss;Loss	P1_C3;P6_C1	Complete;Complete	SLC25A34-AS1, TMEM82, FB		3	0
chr1:1,535,600-4,121,665	2,586,065	36.33 - p36.3	20	65	AI;AI	P1_C3;P6_C1	Complete;Complete	LOC105378586, A	hsa-mir-4251	71	2
chrX:740,019-1,402,609	662,590	p22.33	20	55	Gain;Gain	P6_C1;P7_C1	Complete;Complete	DQ576039, CSF2RA, CRLF2		3	0
chr1:29,573,696-30,640,464	1,066,768	p35.3 - p35.2	20	46	AI;AI	P1_C3;P6_C1	Complete;Complete	PTPRU, LINC01756, BC04253		4	0
chr1:16,085,070-16,500,273	415,203	36.21 - p36.1	20	45	AI;AI	P1_C3;P6_C1	Complete;Complete	FBLIM1, UQCRHL, FLJ37453		13	0
chr1:30,657,099-32,034,839	1,377,740	p35.2	20	44	AI;AI	P1_C3;P6_C1	Complete;Complete	MATN1, MATN1-AS1, MIR44		20	0
chr1:5,958,005-6,610,482	652,477	p36.31	20	35	AI;AI	P1_C3;P6_C1	Complete;Complete	NPHP4, NPHP4, I	hsa-mir-4252	20	1
chr1:35,250,985-35,286,085	35,100	p34.3	20	35	Loss;Loss	P1_C3;P6_C1	Complete;Complete	GJB3, GJA4		2	0
chrX:23,846,331-23,928,519	82,188	p22.11	20	33	Gain;Gain	P6_C1;P7_C1	Complete;Complete	RPL9, APOO, CXorf58		3	0
chr1:15,753,530-15,793,967	40,437	p36.21	20	30	Loss;Loss	P1_C3;P6_C1	Complete;Complete	EFHD2, CTRC, CELA2A		3	0
chr1:24,853,163-26,623,937	1,770,774	p36.11	20	29	AI;AI;Loss	P1_C3;P6_C1;P6_C1	Complete;Complete;Con	RCAN3, LOC100506985, NCM		44	0
chr1:3,024,988-3,332,394	307,406	p36.32	20	27	Loss;Loss	P6_C1;P7_C1	Complete;Complete	MIR4251, PRDM	hsa-mir-4251	2	1
chr1:39,884,102-40,539,413	655,311	p34.3 - p34.2	20	25	AI;AI	P1_C3;P6_C1	Complete;Complete	MACF1, BMP8A, OXCT2P1, I		23	0
chr1:6,610,482-6,636,869	26,387	p36.31	20	22	Loss;Loss	P1_C3;P6_C1	Complete;Complete	NOL9, TAS1R1		2	0
chr1:16,727,256-16,763,653	36,397	p36.13	20	20	Loss;Loss	P1_C3;P6_C1	Complete;Complete	SPATA21		1	0
chr1:33,229,096-34,177,832	948,736	p35.1	20	12	AI;AI	P1_C3;P6_C1	Complete;Complete	KIAA1522, YARS, S100PBP, I		21	0
chr1:40,882,598-40,980,681	98,083	p34.2	20	12	AI;AI;Gain	P1_C3;P6_C1;P6_C1	Complete;Complete;Con	SMAP2, ZFP69B, ZFP69, EXC		4	0
chr19:36,857,232-36,945,488	88,256	q13.12	20	11	Gain;Gain	P1_C3;P6_C1	Complete;Complete	ZFP14, ZFP82, LOC644189, Z		4	0
chr1:40,981,271-41,347,650	366,379	p34.2	20	11	AI;AI	P1_C3;P6_C1	Complete;Complete	EXO5, ZNF684, F	hsa-mir-30e,	10	2
chr1:31,989,222-32,034,839	45,617	p35.2	20	11	Loss;Loss	P1_C3;P6_C1	Complete;Complete	LINC01226, LOC284551		2	0
chrX:32,520,932-32,653,566	132,634	p21.1	20	10	AI;AI	P1_C4;P1_C5	Complete;Complete	DMD, JA783548, JA783867, J		6	0
chr1:4,207,376-5,949,942	1,742,566	36.32 - p36.3	20	10	AI;AI	P1_C3;P6_C1	Complete;Complete	LINC01777, LOC284661, LIN		10	0
chr1:39,097,607-39,876,129	778,522	p34.3	20	9	AI;AI	P1_C3;P6_C1	Complete;Complete	RRAGC, MYCBP, GJA9-MYC		12	0

chr12:52,961,642-52,967,401	5,759	q13.13	20	9	Loss;Loss	P6_C1;P7_C1	Complete;Complete	KRT74		1	0
chrX:32,653,566-32,821,372	167,806	p21.1	20	9	Loss;Loss	P1_C4;P1_C5	Complete;Complete	DMD, MIR548F5	hsa-mir-548f	5	1
chr4:39,604,400-39,838,356	233,956	p14	20	8	Gain;Gain	P6_C1;P7_C1	Complete;Complete	SMIM14, BC040333, UBE2K,		4	0
chrX:32,821,372-33,017,944	196,572	p21.1	20	8	AI;AI	P1_C4;P1_C5	Complete;Complete	DMD, JA783513, JA783507, JA		7	0
chr1:36,625,781-37,890,108	1,264,327	p34.3	20	8	AI;AI	P1_C3;P6_C1	Complete;Complete	MAP7D1, THRAI	hsa-mir-4255	12	1
chr1:34,667,156-35,250,985	583,829	p34.3	20	7	AI;AI	P1_C3;P6_C1	Complete;Complete	C1orf94, MIR552,	hsa-mir-552	6	1
chr1:14,005,776-15,753,530	1,747,754	p36.21	20	6	AI;AI	P1_C3;P6_C1	Complete;Complete	PRDM2, AK124197, KAZN-AS		11	0
chr1:34,837,557-35,052,807	215,250	p34.3	20	6	Loss;Loss	P1_C3;P6_C1	Complete;Complete			0	0
chr1:32,342,851-32,834,096	491,245	p35.2 - p35.1	20	5	AI;AI	P1_C3;P6_C1	Complete;Complete	PTP4A2, KHDRBS1, MIR5585		19	0
chr1:29,260,519-29,394,266	133,747	p35.3	20	4	Loss;Loss	P1_C3;P6_C1	Complete;Complete	EPB41		1	0
chr1:26,650,741-26,682,372	31,631	p36.11	20	4	Loss;Loss	P1_C3;P6_C1	Complete;Complete	AIM1L, CRYBG2		2	0
chr1:37,940,154-38,917,393	977,239	p34.3	20	3	AI;AI	P1_C3;P6_C1	Complete;Complete	ZC3H12A, MIR6732, MIR5581		26	0
chr18:18,570,235-18,751,151	180,916	q11.1	20	3	Gain;Gain	P6_C1;P7_C1	Complete;Complete	ROCK1		1	0
chr1:36,238,746-36,550,625	311,879	p34.3	20	2	AI;AI;Gain	P1_C3;P6_C1;P6_C1	Complete;Complete;Con	AGO4, AGO1, AK025726, AG		5	0
chr1:6,636,869-7,853,282	1,216,413	36.31 - p36.2	20	2	AI;AI	P1_C3;P6_C1	Complete;Complete	TAS1R1, ZBTB48, KLHL21, P		12	0
chr1:7,869,964-8,049,903	179,939	p36.23	20	2	AI;AI	P1_C3;P6_C1	Complete;Complete	PER3, UTS2, TNFRSF9, TRNA		6	0
chr1:4,864,615-4,927,992	63,377	p36.32	20	1	Loss;Loss	P1_C3;P6_C1	Complete;Complete			0	0
chr1:35,286,085-35,361,311	75,226	p34.3	20	1	AI;AI	P1_C3;P6_C1	Complete;Complete	SMIM12, DLGAP3		2	0
chr1:43,608,641-43,785,044	176,403	p34.2	20	1	AI;AI	P1_C3;P6_C1	Complete;Complete	AK309744, FAM183A, DL489		10	0
chrX:32,398,544-32,476,215	77,671	p21.1	20	1	AI;AI	P1_C4;P1_C5	Complete;Complete	DMD, JA202330, JA783579, JA		5	0
chr1:37,507,761-37,611,548	103,787	p34.3	20	1	Loss;Loss	P1_C3;P7_C1	Complete;Complete			0	0
chr1:34,251,396-34,657,642	406,246	p35.1 - p34.3	20	1	AI;AI	P1_C3;P6_C1	Complete;Complete	CSMD2, HMGB4, CSMD2-AS		5	0
chrX:17,608,995-17,757,011	148,016	p22.13	20	0	Loss;Loss	P1_C4;P7_C1	Complete;Complete	NHS, SCML1		2	0
chr1:4,597,360-4,685,447	88,087	p36.32	20	0	Loss;Loss	P1_C3;P6_C1	Complete;Complete	LINC01646		1	0
chr1:4,442,897-4,541,407	98,510	p36.32	20	0	Loss;Loss	P1_C3;P6_C1	Complete;Complete	LINC01777, LOC284661		2	0
chr1:10,511,101-10,523,645	12,544	p36.22	20	0	Loss;Loss	P1_C3;P6_C1	Complete;Complete	CENPS-CORT, APITD1-CORT		4	0
chr1:32,083,323-32,092,630	9,307	p35.2	20	0	Loss;Loss	P1_C3;P6_C1	Complete;Complete	HCRTR1		1	0
chr1:32,092,630-32,256,409	163,779	p35.2	20	0	AI;AI	P1_C3;P6_C1	Complete;Complete	HCRTR1, AX747	hsa-mir-4254	9	1
chr1:32,256,409-32,342,851	86,442	p35.2	20	0	Loss;Loss	P1_C3;P6_C1	Complete;Complete	BC069257, SPOCD1		2	0
chr1:34,177,832-34,251,396	73,564	p35.1	20	0	Loss;Loss	P1_C3;P6_C1	Complete;Complete	CSMD2		1	0
chr1:43,806,113-43,854,818	48,705	p34.2	20	0	AI;AI	P1_C3;P6_C1	Complete;Complete	MPL, CDC20, MIR6734, ELOV		5	0
chr1:5,949,942-5,958,005	8,063	p36.31	20	0	Loss;Loss	P1_C3;P6_C1	Complete;Complete	NPHP4, NPHP4		2	0
chr1:7,853,282-7,869,964	16,682	p36.23	20	0	Loss;Loss	P1_C3;P6_C1	Complete;Complete	PER3		1	0
chr2:179,613,414-179,615,962	2,548	q31.2	20	0	Loss;Loss	P6_C1;P7_C1	Complete;Complete	TTN		1	0
chr4:7,435,824-7,437,211	1,387	p16.1	20	0	Loss;Loss	P6_C1;P7_C1	Complete;Complete	PSAPL1, SORCS2		2	0
chr14:35,307,619-35,344,875	37,256	q13.2	20	0	Gain;Gain	P6_C1;P7_C1	Complete;Complete	BAZ1A		1	0
chr19:12,186,566-12,192,228	5,662	p13.2	20	0	Gain;Gain	P1_C3;P6_C1	Complete;Complete	ZNF844		1	0
chr19:36,996,231-37,038,568	42,337	q13.12	20	0	Gain;Gain	P1_C3;P6_C1	Complete;Complete	ZNF260, ZNF529		2	0
chrX:107,808,354-107,879,164	70,810	q22.3	20	0	Loss;Loss	P1_C3;P1_C4	Complete;Complete	COL4A5		1	0
chrX:12,201,134-12,233,556	32,422	p22.2	20	0	Loss;Loss	P1_C4;P6_C1	Complete;Complete	FRMPD4		1	0
chrX:150,830,685-150,840,724	10,039	q28	20	0	Loss;Loss	P1_C4;P6_C1	Complete;Complete	PASD1		1	0
chrX:38,984,318-39,426,437	442,119	p11.4	20	0	Loss;Loss	P1_C4;P7_C1	Complete;Complete	LINC01281, LOC286442, LINC		3	0
chr1:1,116,668-1,435,417	318,749	p36.33	10	100	Loss	P6_C1	Complete	TTLL10, TNFRSF18, TNFRSF		34	0
chr1:171,175,203-171,190,847	15,644	q24.3	10	100	Gain	P1_C3	Complete	FMO2		1	0
chr1:196,963,333-196,977,676	14,343	q31.3	10	100	Loss	P1_C3	Complete	CFHR5		1	0
chr1:22,169,273-22,176,800	7,527	p36.12	10	100	Loss	P1_C3	Complete	HSPG2		1	0
chr1:247,822,077-248,312,884	490,807	q44	10	100	AI	P7_C1	Complete	OR13G1, OR6F1, OR14A2, OR		20	0
chr1:80,074,062-80,394,896	320,834	p31.1	10	100	AI;Loss	P1_C3;P1_C3	Complete;Complete			0	0
chr1:80,461,852-81,618,394	1,156,542	p31.1	10	100	Loss	P1_C3	Complete	LINC01781		1	0
chr1:80,461,852-81,779,595	1,317,743	p31.1	10	100	AI	P1_C3	Complete	LINC01781, LPHN2, ADGRL2		3	0
chr1:83,585,827-83,695,087	109,260	p31.1	10	100	Loss	P1_C3	Complete	LINC01362		1	0
chr1:87,023,185-87,039,174	15,989	p22.3	10	100	Loss	P1_C3	Complete	CLCA4, CLCA4-AS1		2	0
chr1:979,185-1,093,517	114,332	p36.33	10	100	Loss	P6_C1	Complete	AGRN, AK310350, LOC10028		8	0
chr2:1,499,788-1,501,643	1,855	p25.3	10	100	Loss	P7_C1	Complete	TPO		1	0
chr2:186,672,055-186,673,716	1,661	q32.1	10	100	Loss	P1_C3	Complete	FSIP2		1	0
chr3:10,799,126-10,993,508	194,382	p25.3	10	100	Loss	P7_C1	Complete	LINC00606, SLC6A11		2	0

chr3:22,202,592-22,210,987	8,395	p24.3	10	100	Loss	P1_C3	Complete	ZNF385D		1	0
chr3:76,438,793-76,482,223	43,430	p12.3	10	100	Loss	P1_C3	Complete	ROBO2		1	0
chr3:77,441,998-77,500,555	58,557	p12.3	10	100	Loss	P1_C3	Complete	ROBO2		1	0
chr4:71,024,361-71,043,230	18,869	q13.3	10	100	Gain	P3_C3	Complete	C4orf40, PRR27		2	0
chr5:13,913,886-13,916,913	3,027	p15.2	10	100	Loss	P1_C3	Complete	DNAH5		1	0
chr5:45,992,925-46,399,093	406,168	p12 - p11	10	100	Gain	P1_C3	Complete			0	0
chr5:624,589-640,672	16,083	p15.33	10	100	Loss	P7_C1	Complete	CEP72		1	0
chr5:68,664,058-68,709,767	45,709	q13.2	10	100	Gain	P7_C1	Complete	AK6, TAF9, RAD17		3	0
chr7:157,554,106-157,759,935	205,829	q36.3	10	100	Loss	P7_C1	Complete	LOC100506585, PTPRN2		2	0
chr7:16,408,694-16,442,855	34,161	p21.2	10	100	Gain	P1_C3	Complete	ISPD		1	0
chr8:142,446,922-142,455,808	8,886	q24.3	10	100	Loss	P7_C1	Complete	MROH5		1	0
chr8:39,445,543-39,535,440	89,897	p11.22	10	100	Loss	P1_C3	Complete	ADAM18		1	0
chr8:421,067-462,460	41,393	p23.3	10	100	Loss	P7_C1	Complete	AK056623, TDRP		2	0
chr8:5,532,431-5,533,822	1,391	p23.2	10	100	Loss	P1_C3	Complete			0	0
chr8:53,832,888-53,948,445	115,557	q11.23	10	100	AI	P7_C1	Complete	NPBWR1		1	0
chr8:617,755-647,007	29,252	p23.3	10	100	Loss	P7_C1	Complete	ERICH1		1	0
chr8:72,955,983-72,964,123	8,140	q13.3	10	100	Loss	P1_C3	Complete	LOC100132891, MSC-AS1, TR		3	0
chr9:137,656,205-137,707,833	51,628	q34.3	10	100	Loss	P7_C1	Complete	COL5A1		1	0
chr9:137,777,585-137,809,972	32,387	q34.3	10	100	Loss	P7_C1	Complete	FCN2, FCN1		2	0
chr9:65,629,772-69,238,239	3,608,467	q12 - q21.11	10	100	Gain	P1_C3	Complete	DQ600787, AK30	hsa-mir-1299	39	1
chr10:42,467,126-42,819,463	352,337	q11.21	10	100	Gain	P1_C3	Complete			0	0
chr10:90,350,284-90,357,404	7,120	q23.31	10	100	Loss	P1_C3	Complete	LIPJ		1	0
chr11:113,270,412-113,272,453	2,041	q23.2	10	100	Loss	P7_C1	Complete	ANKK1		1	0
chr11:2,434,299-2,444,227	9,928	p15.5	10	100	Loss	P7_C1	Complete	TRPM5		1	0
chr12:131,620,646-131,675,762	55,116	q24.33	10	100	Loss	P7_C1	Complete	GPR133, ADGRD1, LINC0125		4	0
chr12:131,752,611-131,861,853	109,242	q24.33	10	100	Loss	P7_C1	Complete	BC042649, LINC02415, LINC0		4	0
chr12:21,199,750-21,242,964	43,214	p12.2	10	100	Loss	P1_C3	Complete	SLCO1B3, SLCO1B7		2	0
chr12:21,329,564-21,331,875	2,311	p12.1	10	100	Loss	P1_C3	Complete	SLCO1B1		1	0
chr14:19,327,823-20,444,803	1,116,980	q11.2	10	100	AI	P7_C1	Complete	OR11H12, LINC02297, LOC64		42	0
chr15:20,071,673-20,771,825	700,152	q11.1 - q11.2	10	100	Gain	P7_C1	Complete	DQ576041, DQ571479, BC107		14	0
chr16:31,922,202-31,933,060	10,858	p11.2	10	100	Gain	P1_C3	Complete	ZNF267		1	0
chr16:731,415-736,000	4,585	p13.3	10	100	Gain	P1_C3	Complete	STUB1, JMJD8, WDR24		3	0
chr17:21,977,286-22,242,355	265,069	p11.2 - p11.1	10	100	Gain	P7_C1	Complete	TRNA, JB137816, MTRNR2L1		4	0
chr17:35,632,081-35,819,506	187,425	q12	10	100	Gain	P6_C1	Complete	ACACA, C17orf78, TADA2A		3	0
chr17:36,137,066-36,179,875	42,809	q12	10	100	Gain	P6_C1	Complete			0	0
chr17:43,472,805-45,234,631	1,761,826	21.31 - q21.3	10	100	Loss	P6_C1	Complete	AX747793, ARH(	hsa-mir-4315	49	1
chr17:80,543,825-80,544,460	635	q25.3	10	100	Gain	P1_C3	Complete	FOXK2		1	0
chr19:11,942,914-12,063,197	120,283	p13.2	10	100	Gain	P1_C3	Complete	ZNF440, AX747599, ZNF439,		6	0
chr19:19,992,095-20,050,462	58,367	p13.11 - p12	10	100	Gain	P1_C3	Complete	ZNF253, ZNF93		2	0
chr19:20,113,887-20,120,656	6,769	p12	10	100	Gain	P1_C3	Complete	ZNF682		1	0
chr19:21,989,927-21,992,560	2,633	p12	10	100	Gain	P1_C3	Complete	ZNF43		1	0
chr19:24,302,116-24,518,609	216,493	p12 - p11	10	100	Gain	P1_C3	Complete	ZNF254, HAVCR1P1		2	0
chr19:44,978,870-44,982,169	3,299	q13.31	10	100	Gain	P1_C3	Complete	ZNF180		1	0
chr19:52,496,293-52,499,542	3,249	q13.41	10	100	Gain	P1_C3	Complete	AK128361, ZNF615		2	0
chr19:52,609,348-52,623,143	13,795	q13.41	10	100	Gain	P1_C3	Complete	ZNF616		1	0
chr19:53,116,195-53,116,982	787	q13.41	10	100	Gain	P1_C3	Complete	ZNF83		1	0
chr19:53,301,803-53,344,911	43,108	q13.41	10	100	Gain	P1_C3	Complete	ZNF28, ZNF468		2	0
chr19:53,571,086-53,573,260	2,174	q13.41	10	100	Gain	P1_C3	Complete	ZNF160		1	0
chr19:53,643,102-53,669,096	25,994	q13.42	10	100	Gain	P1_C3	Complete	ZNF347, ZNF665		2	0
chr19:53,740,245-53,741,421	1,176	q13.42	10	100	Gain	P1_C3	Complete	ZNF677		1	0
chr19:53,953,792-53,969,482	15,690	q13.42	10	100	Gain	P1_C3	Complete	ZNF765-ZNF761, ZNF761		2	0
chr19:8,909,362-8,922,581	13,219	p13.2	10	100	Gain	P1_C3	Complete	ZNF558		1	0
chr19:9,865,817-9,869,987	4,170	p13.2	10	100	Gain	P1_C3	Complete	ZNF846		1	0
chr20:23,643,422-23,743,152	99,730	p11.21	10	100	Loss	P7_C1	Complete	CST4, CST1		2	0
chr20:40,687,854-40,701,384	13,530	q12	10	100	Loss	P7_C1	Complete			0	0
chrX:1,427,577-1,463,086	35,509	p22.33	10	100	Loss	P1_C4	Complete	CSF2RA, IL3RA, CRLF2		3	0

chrX:1,472,307-1,499,802	27,495	p22.33	10	100	Gain	P7_C1	Complete	IL3RA, CRLF2		2	0
chrX:152,469,090-152,710,671	241,581	q28	10	100	Loss	P1_C4	Complete	MAGEA1, PNMA6F, ZNF275,		9	0
chrX:152,469,090-153,247,994	778,904	q28	10	100	AI	P1_C4	Complete	MAGEA1, PNMA	hsa-mir-3202	36	2
chrX:153,247,994-153,291,683	43,689	q28	10	100	Loss	P1_C4	Complete	TMEM187, IRAK	hsa-mir-718	4	1
chrX:153,297,256-153,414,892	117,636	q28	10	100	Loss	P1_C4	Complete	MECP2, OPN1LW		2	0
chrX:153,414,892-153,688,233	273,341	q28	10	100	AI	P1_C4	Complete	OPN1LW, TEX28, OPN1MW2		23	0
chrX:153,688,233-153,692,697	4,464	q28	10	100	Loss	P1_C4	Complete	PLXNA3		1	0
chrX:2,727,632-2,799,215	71,583	p22.33	10	100	Loss	P1_C4	Complete	XG, GYG2		2	0
chrX:48,338,774-48,380,511	41,737	p11.23	10	100	Loss	P1_C4	Complete	FTSJ1, LOC101927635, PORC		4	0
chrX:48,460,318-48,655,131	194,813	p11.23	10	100	Loss	P1_C4	Complete	WDR13, WAS, SUV39H1, GL		5	0
chrX:48,842,429-48,933,243	90,814	p11.23	10	100	Loss	P1_C4	Complete	GRIPAP1, TFE3, CCDC120, P		5	0
chrX:49,032,926-49,069,847	36,921	p11.23	10	100	Loss	P1_C4	Complete	PRICKLE3, SYP, SYP-AS1, C		4	0
chrX:53,624,056-54,956,804	1,332,748	11.22 - p11.2	10	100	AI;Loss	P1_C4;P1_C4	Complete;Complete	HUWE1, PHF8, FAM120C, W		13	0
chrX:55,039,684-55,185,606	145,922	p11.21	10	100	Loss	P1_C4	Complete	ALAS2, PAGE2B, PAGE2, FA		4	0
chrX:67,966,690-68,146,237	179,547	q13.1	10	100	AI	P1_C4	Complete	EFNB1		1	0
chrX:70,160,409-70,294,689	134,280	q13.1	10	100	AI;Loss	P1_C4;P1_C4	Complete;Complete	SNX12		1	0
chrX:70,357,165-70,812,128	454,963	q13.1	10	100	Loss	P1_C4	Complete	MED12, NLGN3, GJB1, ZMY		12	0
chr14:104,843,202-104,987,571	144,369	q32.33	10	99	Loss	P7_C1	Complete	AX746996		1	0
chr1:0-909,867	909,867	p36.33	10	99	Loss	P6_C1	Complete	LOC102725121, I	hsa-mir-1302	47	1
chr5:0-286,460	286,460	p15.33	10	97	Loss	P7_C1	Complete	PLEKHG4B, LRRC14B, CCD		6	0
chr8:0-256,689	256,689	p23.3	10	96	Loss	P7_C1	Complete	OR4F21, RPL23AP53, ZNF59		3	0
chrX:153,940,179-155,270,560	1,330,381	q28	10	96	AI;Loss	P1_C4;P1_C4	Complete;Complete	GAB3, SNORA36	hsa-mir-1184	38	3
chr8:0-197,812	197,812	p23.3	10	95	Gain	P1_C3	Complete	OR4F21, RPL23AP53, ZNF59		3	0
chr4:0-191,453	191,453	p16.3	10	95	Gain	P1_C3	Complete	ZNF595, ZNF718		2	0
chr4:10,027,555-10,339,123	311,568	p16.1	10	92	Loss	P7_C1	Complete	SLC2A9, MIR313	hsa-mir-3138	12	1
chr2:0-280,262	280,262	p25.3	10	86	Loss	P7_C1	Complete	FAM110C, SH3YL1, ACP1, F		5	0
chr11:134,717,764-134,896,811	179,047	q25	10	84	Loss	P7_C1	Complete	AK130852, LOC100507548		2	0
chr12:133,500,911-133,792,201	291,290	q24.33	10	84	Gain	P1_C3	Complete	ZNF605, ZNF26, LOC1019285		9	0
chr12:52,665,539-52,804,288	138,749	q13.13	10	83	Loss	P7_C1	Complete	KRT86, KRT81, KRT83, KRT		6	0
chr8:143,662,172-143,746,406	84,234	q24.3	10	82	Loss	P7_C1	Complete	ARC, LOC101928087, JH8, JR		4	0
chr17:32,340,948-32,942,574	601,626	q12	10	81	Loss	P6_C1	Complete	ASIC2, BC062794, LINC0198		11	0
chr19:21,055,220-21,141,796	86,576	p12	10	80	Gain	P1_C3	Complete	ZNF85		1	0
chr9:99,521,121-99,581,466	60,345	q22.33	10	80	Gain	P1_C3	Complete	ZNF510, ZNF782		2	0
chrX:69,498,038-70,145,724	647,686	q13.1	10	78	AI;Loss	P1_C4;P1_C4	Complete;Complete	ARR3, RAB41, PDZD11, KIF4		11	0
chr9:116,854,255-116,871,425	17,170	q32	10	75	Loss	P7_C1	Complete	KIF12		1	0
chr17:39,059,866-39,593,520	533,654	q21.2	10	75	AI	P6_C1	Complete	KRT23, KRT39, KRT40, KRT		44	0
chr3:93,537,290-93,652,298	115,008	q11.1	10	71	Gain	P7_C1	Complete	PROS1		1	0
chrX:152,144,701-152,226,564	81,863	q28	10	69	Loss	P1_C4	Complete	PNMA5, PNMA3		2	0
chr17:60,232,717-60,435,430	202,713	q23.2	10	69	Gain	P7_C1	Complete	Mir_652, TBC1D3P2		2	0
chrX:47,511,659-48,311,022	799,363	p11.23	10	68	Loss	P1_C4	Complete	UXT, UXT-AS1, LOC1001339		20	0
chr10:134,649,258-134,783,002	133,744	q26.3	10	67	Loss	P7_C1	Complete	TTC40, CFAP46, LINC01166,		6	0
chr17:34,051,150-35,632,081	1,580,931	q12	10	67	AI	P6_C1	Complete	AP2B1, RASL10	hsa-mir-2909	55	1
chr12:31,821,063-32,029,691	208,628	p11.21	10	63	Gain	P7_C1	Complete	ETFBKMT, METTL20, AMN1		4	0
chr1:82,987,363-83,585,827	598,464	p31.1	10	63	AI	P1_C3	Complete	U80773, LINC01361, LINC013		3	0
chrX:70,839,840-71,338,015	498,175	q13.1	10	61	Loss	P1_C4	Complete	BCYRN1, LOC100129291, LO		9	0
chrX:111,326,811-114,419,335	3,092,524	q23	10	60	AI;Loss	P1_C4;P1_C4	Complete;Complete	RTL4, ZCCHC16,	hsa-mir-4329	19	7
chr17:36,179,875-37,223,923	1,044,048	q12	10	59	AI	P6_C1	Complete	YWHAEP7, LOC284100, DQ5		51	0
chr1:81,779,595-81,891,838	112,243	p31.1	10	57	Loss	P1_C3	Complete	LPHN2, ADGRL2		2	0
chr3:139,785,476-140,120,148	334,672	q23	10	57	Loss	P7_C1	Complete	CLSTN2		1	0
chrX:151,131,234-152,103,004	971,770	q28	10	53	AI;Loss	P1_C4;P1_C4	Complete;Complete	GABRE, MAGEA	hsa-mir-105-	26	3
chr1:12,253,008-12,606,559	353,551	p36.22	10	53	Loss	P6_C1	Complete	TNFRSF1B, VPS13D, SNORA		4	0
chr8:142,836,252-143,192,639	356,387	q24.3	10	52	Loss	P7_C1	Complete	MIR1302-7, MIR4	hsa-mir-1302	2	1
chr20:58,348,452-58,490,548	142,096	13.32 - q13.3	10	50	Loss	P7_C1	Complete	PHACTR3, SYCP2		2	0
chr2:455,271-1,376,873	921,602	p25.3	10	50	Loss	P7_C1	Complete	LINC01874, LINC01875, LOC		9	0
chrX:3,541,301-9,486,472	5,945,171	22.33 - p22.3	10	48	Loss	P1_C4	Complete	PRKX, PRKX-AS	hsa-mir-651	31	1
chr6:168,746,922-168,973,885	226,963	q27	10	47	Loss	P7_C1	Complete	SMOC2		1	0

chrX:68,146,237-68,320,225	173,988	q13.1	10	47	Loss	P1_C4	Complete			0	0
chrX:3,325,312-9,486,472	6,161,160	22.33 - p22.3	10	46	AI	P1_C4	Complete	SNORA48B, PRK	hsa-mir-651	32	1
chrX:47,084,260-48,311,022	1,226,762	p11.23	10	45	AI	P1_C4	Complete	CDK16, USP11, SNORA11C, Z		32	0
chrX:84,382,064-97,073,640	12,691,576	21.1 - q21.33	10	42	AI;Loss	P1_C4;P1_C4	Complete;Complete	LOC101928128, Z	hsa-mir-1321	30	3
chrX:40,885,573-41,284,003	398,430	p11.4	10	42	Gain	P7_C1	Complete	USP9X, LINC02601, 5S_rRNA		4	0
chrX:114,444,931-118,817,505	4,372,574	q23 - q24	10	41	Loss	P1_C4	Complete	LRCH2, LUZP4, I	hsa-mir-1277	36	2
chr1:16,763,653-18,806,851	2,043,198	p36.13	10	41	AI	P1_C3	Complete	SPATA21, NECAP2, LINC017		46	0
chrX:125,371,689-128,893,613	3,521,924	q25 - q26.1	10	41	AI;Loss	P1_C4;P1_C4	Complete;Complete	DCAF12L1, PRR32, CXorf64,		9	0
chr20:59,979,125-60,297,508	318,383	q13.33	10	39	Loss	P7_C1	Complete	CDH4, AK097866, LOC10012		3	0
chr9:122,763,700-122,904,242	140,542	q33.2	10	38	Loss	P6_C1	Complete			0	0
chrX:299,022-1,411,880	1,112,858	p22.33	10	37	AI;Loss	P1_C4;P1_C4	Complete;Complete	PPP2R3B, SHOX, DQ576039,		5	0
chrX:114,444,931-119,500,087	5,055,156	q23 - q24	10	37	AI	P1_C4	Complete	LRCH2, LUZP4, I	hsa-mir-1277	56	2
chr17:39,623,397-40,572,713	949,316	q21.2	10	36	AI	P6_C1	Complete	KRT32, KRT35, KRT36, KRT		48	0
chr19:57,065,635-57,089,526	23,891	q13.43	10	36	Gain	P1_C3	Complete	ZFP28, BX647249, ZNF470		3	0
chr1:83,695,087-84,895,911	1,200,824	p31.1	10	36	AI	P1_C3	Complete	BC043544, LINC01712, BC03		15	0
chrX:49,114,392-53,057,918	3,943,526	11.23 - p11.2	10	35	AI;Loss	P1_C4;P1_C4	Complete;Complete	FOXP3, FLICR, P	hsa-mir-532,	93	8
chr6:169,634,881-169,648,338	13,457	q27	10	35	Loss	P7_C1	Complete	LOC101929523, THBS2		2	0
chr1:227,820,384-227,852,739	32,355	q42.13	10	34	Gain	P1_C3	Complete	ZNF678		1	0
chr5:2,001,955-2,107,805	105,850	p15.33	10	34	Loss	P7_C1	Complete			0	0
chrX:128,920,376-129,061,911	141,535	q26.1	10	33	Loss	P1_C4	Complete	SASH3, ZDHHC9, UTP14A		3	0
chr17:35,917,877-36,137,066	219,189	q12	10	32	AI	P6_C1	Complete	SYNRG, MIR378J, DDX52, H		4	0
chr1:21,070,820-22,169,273	1,098,453	p36.12	10	32	AI	P1_C3	Complete	HP1BP3, MIR125	hsa-mir-1256	14	1
chr1:55,054,837-55,184,252	129,415	p32.3	10	32	Loss	P1_C3	Complete	ACOT11, FAM151A, MROH7,		6	0
chr11:2,611,513-2,770,719	159,206	p15.5	10	32	Loss	P7_C1	Complete	KCNQ1, KCNQ1OT1		2	0
chr1:11,010,423-12,779,573	1,769,150	36.22 - p36.2	10	31	AI	P1_C3	Complete	C1orf127, TARDBP, MASP2,		43	0
chr17:30,789,283-33,816,223	3,026,940	q11.2 - q12	10	31	AI	P6_C1	Complete	PSMD11, AK307275, CDK5R		34	0
chr12:34,433,267-35,800,000	1,366,733	p11.1 - q11	10	31	Gain	P1_C3	Complete			0	0
chr1:76,882,130-77,418,871	536,741	p31.1	10	30	AI	P1_C3	Complete	ST6GALNAC3, LINC02567, S		3	0
chr17:40,694,197-40,716,961	22,764	q21.2	10	30	Gain	P6_C1	Complete	NAGLU, BC043620, HSD17B		5	0
chr2:179,498,140-179,539,809	41,669	q31.2	10	29	Loss	P6_C1	Complete	MIR548N, TTN, TTN, TTN		4	0
chr1:24,853,163-26,623,937	1,770,774	p36.11	10	29	AI;AI;Loss	P1_C3;P6_C1;P6_C1	Complete;Complete;Con	RCAN3, LOC100506985, NCM		44	0
chr1:9,063,251-9,365,743	302,492	36.23 - p36.2	10	29	Loss	P6_C1	Complete	SLC2A7, SLC2A5	hsa-mir-34a	9	1
chr6:61,000,000-62,229,529	1,229,529	q11.1	10	28	Gain	P6_C1	Complete			0	0
chrX:55,247,953-58,409,750	3,161,797	p11.21 - p11.1	10	28	Loss	P1_C4	Complete	PAGE5, PAGE3, LOC1004217		21	0
chr5:2,663,987-2,749,729	85,742	p15.33	10	27	Loss	P7_C1	Complete	LSINCT5, IRX2		2	0
chrX:61,835,206-63,409,452	1,574,246	q11.1 - q11.2	10	27	Loss	P1_C4	Complete	SPIN4, AK130294	hsa-mir-1468	8	1
chr19:11,886,910-11,917,904	30,994	p13.2	10	26	Gain	P1_C3	Complete	ZNF441, ZNF491		2	0
chr17:41,170,205-41,569,311	399,106	q21.31	10	26	Gain	P6_C1	Complete	VAT1, RND2, BR	hsa-mir-2117	17	1
chr1:22,176,800-22,909,649	732,849	p36.12	10	25	AI	P1_C3	Complete	HSPG2, CELA3B, CELA3A, L		10	0
chr1:10,523,645-10,708,791	185,146	p36.22	10	25	AI	P1_C3	Complete	DFFA, PEX14, CASZ1		3	0
chrX:63,445,950-67,966,690	4,520,740	q11.2 - q13.1	10	24	Loss	P1_C4	Complete	ASB12, MTMR8,	hsa-mir-223	18	1
chr1:84,294,745-84,649,712	354,967	p31.1	10	24	Loss	P1_C3	Complete	BC036594, LINC01725, LOC1		8	0
chr19:12,297,183-12,385,214	88,031	p13.2	10	24	Gain	P1_C3	Complete	ZNF136, AX721123, LOC1002		5	0
chr1:53,372,022-53,675,769	303,747	p32.3	10	23	AI	P1_C3	Complete	ECHDC2, SCP2, PODN, SLC1		6	0
chr19:37,179,322-37,441,930	262,608	q13.12	10	23	Gain	P1_C3	Complete	ZNF567, ZNF850, AX747375,		10	0
chrX:18,996,079-22,291,205	3,295,126	22.13 - p22.1	10	22	AI;Loss	P1_C4;P1_C4	Complete;Complete	PHKA2, GPR64,	hsa-mir-1308	29	1
chr9:17,576,554-17,849,639	273,085	p22.2	10	22	AI	P6_C1	Complete	SH3GL2		1	0
chr1:17,548,895-17,731,753	182,858	p36.13	10	22	Loss	P1_C3	Complete	PADI1, Mir_584, PADI3, MIR		6	0
chr1:46,500,462-46,512,006	11,544	p34.1	10	21	Loss	P1_C3	Complete	MAST2, PIK3R3, LOC110117		3	0
chrX:44,250,177-44,699,893	449,716	p11.3	10	21	Gain	P7_C1	Complete	FUNDC1		1	0
chr1:71,847,244-74,333,047	2,485,803	p31.1	10	20	Loss	P1_C3	Complete	NEGR1-IT1, NEGR1, BC0413		5	0
chr1:53,683,501-54,255,873	572,372	p32.3	10	20	AI	P1_C3	Complete	C1orf123, CZIB, MAGOH, LO		11	0
chr17:29,873,697-30,587,181	713,484	q11.2	10	20	Gain	P6_C1	Complete	TRNA_Thr, MIR	hsa-mir-193a	15	2
chr1:23,759,728-24,433,055	673,327	36.12 - p36.1	10	20	AI	P1_C3	Complete	ASAP3, BC038455, E2F2, LO		24	0
chrX:3,229,552-3,325,312	95,760	p22.33	10	19	Loss	P1_C4	Complete	MXRA5		1	0
chr1:9,656,788-9,795,378	138,590	p36.22	10	19	Loss	P6_C1	Complete	TMEM201, PIK3CD-AS1, C1o		7	0

chrX:136,117,688-150,334,531	14,216,843	q26.3 - q28	10	19	AI;Loss	P1_C4;P1_C4	Complete;Complete	ZIC3, LINC00889	hsa-mir-504,	99	25
chrX:1,482,799-2,472,499	989,700	p22.33	10	19	AI;Loss	P1_C4;P1_C4	Complete;Complete	IL3RA, CRLF2, SLC25A6, LIN		11	0
chr19:52,868,973-52,889,869	20,896	q13.41	10	19	Gain	P1_C3	Complete	ZNF610, ZNF880, AF086165		3	0
chr1:55,266,707-55,519,773	253,066	p32.3	10	19	AI	P1_C3	Complete	TTC22, LEXM, C1orf177, DHC		9	0
chr17:37,297,287-37,319,084	21,797	q12	10	18	Gain	P6_C1	Complete	PLXDC1, ARL5C		2	0
chr1:27,724,551-27,875,310	150,759	p36.11	10	18	Loss	P6_C1	Complete	WASF2, AHDC1		2	0
chr1:71,005,838-74,333,047	3,327,209	p31.1	10	18	AI	P1_C3	Complete	BC041441, LINC0	hsa-mir-186	12	1
chrX:22,578,824-24,329,852	1,751,028	p22.11	10	18	AI;Loss	P1_C4;P1_C4	Complete;Complete	PTCHD1-AS, LOC100873065,		17	0
chrX:119,732,294-125,297,952	5,565,658	q24 - q25	10	18	AI;Loss	P1_C4;P1_C4	Complete;Complete	MCTS1, C1GALT	hsa-mir-220a	37	1
chr17:29,783,603-30,587,181	803,578	q11.2	10	18	AI	P6_C1	Complete	RAB11FIP4, MIR	hsa-mir-193a	17	2
chrX:10,176,906-13,983,814	3,806,908	p22.2	10	17	AI	P1_C4	Complete	CLCN4, U6, MID1, Mir_652, A		36	0
chr2:241,398,552-241,509,978	111,426	q37.3	10	17	Loss	P7_C1	Complete	GPC1, ANKMY1, DUSP28, RN		4	0
chr1:71,005,838-71,511,289	505,451	p31.1	10	17	Loss	P1_C3	Complete	BC041441, LINC01788, PTGE		3	0
chr1:79,381,237-79,631,578	250,341	p31.1	10	17	Loss	P1_C3	Complete	ELTD1, ADGRL4		2	0
chr1:79,858,355-79,992,282	133,927	p31.1	10	17	Loss	P1_C3	Complete			0	0
chr10:70,235,908-70,267,466	31,558	q21.3	10	16	Gain	P7_C1	Complete	SLC25A16		1	0
chr17:57,109,494-57,268,231	158,737	q22	10	15	Gain	P7_C1	Complete	TRIM37, BC0172	hsa-mir-454,	6	2
chr17:37,066,637-37,223,923	157,286	q12	10	15	Gain	P6_C1	Complete	LASP1, MIR6779, LINC00672		9	0
chrX:102,979,511-103,077,802	98,291	q22.2	10	14	Loss	P1_C4	Complete	GLRA4, PLP1, RAB9B		3	0
chr1:39,412,023-39,710,788	298,765	p34.3	10	14	Gain	P6_C1	Complete	AKIRIN1, NDUFS5, Mir_562,		5	0
chr19:44,599,622-44,612,377	12,755	q13.31	10	14	Gain	P1_C3	Complete	ZNF224, LOC100379224		2	0
chr1:8,279,708-8,614,184	334,476	p36.23	10	14	Loss	P6_C1	Complete	SLC45A1, BC113958, RERE-A		5	0
chr1:27,970,491-28,421,615	451,124	p36.11 - p35.3	10	14	Loss	P6_C1	Complete	LINC02574, IFI6, FAM76A, ST		11	0
chr1:51,564,451-51,672,190	107,739	p32.3	10	14	Loss	P1_C3	Complete	C1orf185, LINC01562		2	0
chrX:150,350,327-150,908,733	558,406	q28	10	14	AI	P1_C4	Complete	VMA21, LOC105377213, PAS		6	0
chr1:46,746,180-47,717,517	971,337	p34.1 - p33	10	13	AI	P1_C3	Complete	LRRC41, UQCRH, NSUN4, FA		28	0
chr1:67,263,282-67,376,005	112,723	p31.3	10	13	Loss	P1_C3	Complete	INSL5, WDR78		2	0
chrX:99,686,905-102,191,794	2,504,889	q22.1	10	12	AI;Loss	P1_C4;P1_C4	Complete;Complete	TNMD, TSPAN6, SRPX2, SYT		43	0
chr1:26,682,372-29,260,519	2,578,147	p36.11 - p35.3	10	12	AI	P1_C3	Complete	ZNF683, LIN28A	hsa-mir-1976	74	1
chrX:71,564,631-73,569,177	2,004,546	q13.1 - q13.2	10	12	AI;Loss	P1_C4;P1_C4	Complete;Complete	HDAC8, PHKA1,	hsa-mir-421,	33	4
chr1:40,882,598-40,980,681	98,083	p34.2	10	12	AI;AI;Gain	P1_C3;P6_C1;P6_C1	Complete;Complete;Com	SMAP2, ZFP69B, ZFP69, EXO		4	0
chrX:34,971,979-47,062,395	12,090,416	p21.1 - p11.23	10	12	AI	P1_C4	Complete	MAGEB16, CXor	hsa-mir-221,	92	2
chr9:122,278,536-122,391,755	113,219	q33.1	10	12	Loss	P6_C1	Complete			0	0
chrX:71,394,240-71,455,330	61,090	q13.1	10	12	Loss	P1_C4	Complete	ERCC6L, PIN4		2	0
chrX:103,318,445-104,454,509	1,136,064	q22.2 - q22.3	10	12	AI;Loss	P1_C4;P1_C4	Complete;Complete	ZCCHC18, LOC286437, SLC2		6	0
chr1:54,562,238-55,184,252	622,014	p32.3	10	12	AI	P1_C3	Complete	TCEANC2, CDCP2, CYB5RL,		12	0
chr1:39,097,607-39,293,840	196,233	p34.3	10	11	Loss	P1_C3	Complete			0	0
chr1:70,060,521-70,290,434	229,913	p31.1	10	11	Loss	P1_C3	Complete	LRRC7		1	0
chrX:129,220,677-135,047,043	5,826,366	q26.1 - q26.3	10	11	AI;Loss	P1_C4;P1_C4	Complete;Complete	ELF4, AIFM1, RA	hsa-mir-363,	98	12
chr2:239,941,660-240,095,935	154,275	q37.3	10	11	Loss	P7_C1	Complete	MIR4440, MIR4441, HDAC4		3	0
chrX:39,966,240-47,062,395	7,096,155	p11.4 - p11.23	10	11	Loss	P1_C4	Complete	BCOR, LOC1019	hsa-mir-221,	63	2
chr17:37,672,348-38,254,073	581,725	q12 - q21.1	10	10	AI	P6_C1	Complete	CDK12, NEUROD2, PPP1R1B		25	0
chrX:151,022,381-151,120,778	98,397	q28	10	10	Loss	P1_C4	Complete	MAGEA4-AS1, MAGEA4		2	0
chrX:2,936,648-3,216,495	279,847	p22.33	10	10	AI;Loss	P1_C4;P1_C4	Complete;Complete	ARSH, ARSF, LINC01546, CX		4	0
chr17:41,170,205-42,483,189	1,312,984	q21.31	10	10	AI	P6_C1	Complete	VAT1, RND2, BR	hsa-mir-2117	55	1
chr1:64,557,776-66,543,232	1,985,456	p31.3	10	10	AI	P1_C3	Complete	ROR1, BC040909	hsa-mir-101-	22	1
chr1:40,981,271-41,089,463	108,192	p34.2	10	10	Gain	P6_C1	Complete	EXO5, ZNF684, RIMS3		3	0
chr2:170,487,854-170,497,220	9,366	q31.1	10	10	Gain	P1_C3	Complete	PPIG		1	0
chr1:49,344,279-50,559,491	1,215,212	p33	10	10	Loss	P1_C3	Complete	LOC101929721, AGBL4, AGB		4	0
chr10:94,167,942-94,224,457	56,515	q23.33	10	10	Gain	P7_C1	Complete	MARK2P9, AK124121, IDE		3	0
chr17:30,652,705-30,789,283	136,578	q11.2	10	10	Gain	P6_C1	Complete	C17orf75, MIR63	hsa-mir-632	4	1
chr1:40,539,413-40,869,908	330,495	p34.2	10	10	Gain	P6_C1	Complete	PPT1, RLF, TMCO2, ZMPSTE		6	0
chr2:1,664,674-1,729,667	64,993	p25.3	10	9	Loss	P7_C1	Complete	PXDN		1	0
chr1:62,271,228-62,658,334	387,106	p31.3	10	9	AI	P1_C3	Complete	PATJ, INADL, M	hsa-mir-3116	4	2
chr1:78,764,689-79,992,282	1,227,593	p31.1	10	9	AI	P1_C3	Complete	MGC27382, PTGFR, IFI44L, II		7	0
chr1:51,672,190-52,829,304	1,157,114	p32.3	10	9	AI	P1_C3	Complete	LINC01562, RNF	hsa-mir-761	17	1

chr1:75,038,685-76,516,226	1,477,541	p31.1	10	9	Loss	P1_C3	Complete	CR627203, ERICH3-AS1, C1or		19	0
chr1:74,372,848-74,707,481	334,633	p31.1	10	9	Loss	P1_C3	Complete	LRRIQ3, FPGT, FPGT-TNNI3		4	0
chrX:119,006,479-119,500,087	493,608	q24	10	8	Loss	P1_C4	Complete	NDUFA1, AKAP14, NKAP, RI		15	0
chr7:15,608,900-15,699,426	90,526	p21.2	10	8	Gain	P1_C3	Complete	MEOX2		1	0
chr1:46,173,353-46,300,865	127,512	p34.1	10	8	Loss	P1_C3	Complete	IPP, MAST2		2	0
chrX:104,463,817-107,977,755	3,513,938	q22.3	10	8	AI	P1_C4	Complete	IL1RAPL2, TEX13A, NRK, SE		33	0
chr1:69,682,164-70,505,116	822,952	p31.2 - p31.1	10	8	AI	P1_C3	Complete	LINC01758, LRRC7, PIN1P1		3	0
chr1:81,891,838-81,984,467	92,629	p31.1	10	8	AI	P1_C3	Complete	LOC101927434, LPHN2, ADG		3	0
chr1:75,097,454-76,862,102	1,764,648	p31.1	10	8	AI	P1_C3	Complete	C1orf173, ERICH3, CRYZ, TY		18	0
chr1:19,076,861-20,456,280	1,379,419	36.13 - p36.1	10	8	AI	P1_C3	Complete	TAS1R2, MIR469	hsa-mir-129(	33	1
chr17:40,865,367-40,931,054	65,687	q21.2 - q21.31	10	8	Gain	P6_C1	Complete	EZH1, BC047651, RAMP2-AS		5	0
chrX:22,292,557-22,554,841	262,284	p22.11	10	8	Loss	P1_C4	Complete	ZNF645, CBLL2, PTCHD1-AS		4	0
chr11:2,062,613-2,088,838	26,225	p15.5	10	8	Gain	P1_C3	Complete			0	0
chrX:135,095,266-136,112,574	1,017,308	q26.3	10	8	AI;Loss	P1_C4;P1_C4	Complete;Complete	SLC9A6, FHL1, M	hsa-mir-934	16	1
chrX:9,548,930-9,640,322	91,392	p22.2	10	7	Loss	P1_C4	Complete	TBL1X		1	0
chrX:14,040,257-18,909,656	4,869,399	p22.2 - p22.13	10	7	AI	P1_C4	Complete	GEMIN8, UBE2E4P, GLRA2,		50	0
chrX:102,195,390-102,966,557	771,167	q22.1 - q22.2	10	7	AI;Loss	P1_C4;P1_C4	Complete;Complete	LINC00630, BEX1, NXF3, BE		21	0
chr17:42,643,635-42,841,449	197,814	q21.31	10	7	Loss	P6_C1	Complete	LINC01180, MEIOC, C17orf1(		6	0
chr10:49,911,229-50,341,936	430,707	q11.23	10	6	Loss	P7_C1	Complete	WDFY4, WDFY4	hsa-mir-4294	7	1
chr20:24,117,232-24,446,751	329,519	p11.21	10	6	Loss	P7_C1	Complete	FLJ33581, LINC01721		2	0
chr1:46,300,865-46,500,462	199,597	p34.1	10	6	AI	P1_C3	Complete	MAST2		1	0
chr1:60,363,611-61,173,805	810,194	p32.1	10	6	AI	P1_C3	Complete	CYP2J2, C1orf87, LINC01748,		5	0
chrX:97,109,893-99,651,586	2,541,693	q21.33 - q22.1	10	6	AI;Loss	P1_C4;P1_C4	Complete;Complete	Mir_340, XRCC6P5, PCDH19		3	0
chr17:38,254,073-38,303,951	49,878	q21.1	10	6	Gain	P6_C1	Complete	NR1D1, TRNA_SeC, MSL1, D		5	0
chr1:27,215,989-27,410,945	194,956	p36.11	10	6	Loss	P6_C1	Complete	GPN2, GPATCH3, NR0B2, NU		10	0
chr17:33,917,012-34,051,150	134,138	q12	10	6	Gain	P6_C1	Complete	AP2B1		1	0
chr1:78,204,351-78,679,325	474,974	p31.1	10	6	AI;Loss	P1_C3;P1_C3	Complete;Complete	USP33, MIGA1, FAM73A, NE		8	0
chrX:73,745,121-84,360,643	10,615,522	q13.2 - q21.1	10	6	AI;Loss	P1_C4;P1_C4	Complete;Complete	SLC16A2, RLIM,	hsa-mir-384,	48	4
chr1:68,840,655-69,085,434	244,779	p31.3 - p31.2	10	5	Loss	P1_C3	Complete	RPE65, DEPDC1, DEPDC1-AS		4	0
chr1:78,764,689-79,067,071	302,382	p31.1	10	5	Loss	P1_C3	Complete	MGC27382, PTGFR		2	0
chr19:40,517,839-40,588,941	71,102	q13.2	10	5	Gain	P1_C3	Complete	ZNF546, ZNF780B, ZNF780A		3	0
chr1:57,641,292-60,300,944	2,659,652	p32.2 - p32.1	10	5	AI	P1_C3	Complete	DAB1, BC047487, BC033978,		18	0
chr1:47,717,517-47,849,489	131,972	p33	10	5	Loss	P1_C3	Complete	STIL, CMPK1, LINC01389		3	0
chrX:107,979,298-109,694,148	1,714,850	q22.3 - q23	10	5	AI;Loss	P1_C4;P1_C4	Complete;Complete	IRS4, LOC101928	hsa-mir-652	17	1
chr1:48,405,504-51,564,451	3,158,947	p33 - p32.3	10	5	AI	P1_C3	Complete	TRABD2B, SKINTL, SKINT11		15	0
chr1:74,372,848-75,038,685	665,837	p31.1	10	5	AI	P1_C3	Complete	LRRIQ3, FPGT, FPGT-TNNI3		7	0
chr1:22,922,297-23,738,696	816,399	p36.12	10	4	AI	P1_C3	Complete	EPHA8, MIR6127	hsa-mir-4253	23	2
chr2:203,431,235-203,632,877	201,642	q33.2	10	4	Gain	P7_C1	Complete	BMPR2, FAM117B		2	0
chr1:20,475,649-21,012,602	536,953	p36.12	10	4	AI	P1_C3	Complete	PLA2G2F, PLA2G2C, UBXN1		17	0
chr1:11,865,993-12,202,875	336,882	p36.22	10	4	Loss	P6_C1	Complete	MTHFR, CLCN6, NPPA-AS1,		11	0
chr1:57,185,587-57,228,189	42,602	p32.2	10	4	Loss	P1_C3	Complete	FYB2, C1orf168		2	0
chr10:1,692,729-1,738,136	45,407	p15.3	10	4	Loss	P7_C1	Complete	ADARB2		1	0
chr1:77,506,270-77,842,351	336,081	p31.1	10	4	AI;Loss	P1_C3;P1_C3	Complete;Complete	ST6GALNAC5, MIR7156, AF1		5	0
chr6:74,193,675-74,261,920	68,245	q13	10	4	Gain	P7_C1	Complete	MTO1, SNORD141A, SNORD		6	0
chr1:54,307,305-54,433,403	126,098	p32.3	10	3	AI	P1_C3	Complete	YIPF1, DIO1, HSPB11, LRRC4		4	0
chr17:58,522,657-58,596,702	74,045	q23.2	10	3	Gain	P7_C1	Complete	APPBP2		1	0
chr1:57,383,529-57,599,516	215,987	p32.2	10	3	AI	P1_C3	Complete	C8A, C8B, DAB1		3	0
chr17:42,877,811-43,373,464	495,653	q21.31	10	3	Loss	P6_C1	Complete	GJC1, HIGD1B, EFTUD2, CCI		25	0
chr17:37,342,709-37,557,267	214,558	q12	10	3	AI;Gain	P6_C1;P6_C1	Complete;Complete	CACNB1, RPL19, STAC2, FB2		4	0
chr6:40,635,045-40,765,578	130,533	p21.1	10	3	Loss	P7_C1	Complete			0	0
chr1:35,574,559-35,609,297	34,738	p34.3	10	3	Loss	P1_C3	Complete	ZMYM1		1	0
chr2:2,472,754-2,520,606	47,852	p25.3	10	3	Loss	P7_C1	Complete			0	0
chr1:54,433,403-54,562,238	128,835	p32.3	10	3	Loss	P1_C3	Complete	LRRC42, LDLRAD1, TMEM5		5	0
chr1:53,183,173-53,372,022	188,849	p32.3	10	3	Loss	P1_C3	Complete	ZYG11B, ZYG11A, ECHDC2		3	0
chr1:70,575,284-70,651,230	75,946	p31.1	10	3	Loss	P1_C3	Complete	LRRC7, LRRC40		2	0
chr1:63,579,044-63,627,425	48,381	p31.3	10	3	Loss	P1_C3	Complete	LINC00466		1	0

chr1:55,528,204-56,897,195	1,368,991	p32.3 - p32.2	10	3	AI	P1_C3	Complete	PCSK9, USP24, LOC10050763		10	0
chr17:38,483,714-39,059,866	576,152	q21.2	10	2	Loss	P6_C1	Complete	RARA-AS1, RARA, GJD3, TO		18	0
chr1:56,930,780-57,185,587	254,807	p32.2	10	2	AI	P1_C3	Complete	PPAP2B, PLPP3, LOC1019299		6	0
chr1:68,046,291-68,370,341	324,050	p31.3	10	2	AI	P1_C3	Complete	GADD45A, GNG12, U7, GNG		4	0
chr1:47,849,489-48,375,457	525,968	p33	10	2	AI	P1_C3	Complete	AX748181, LINC01389, FOXE		6	0
chr1:36,238,746-36,550,625	311,879	p34.3	10	2	AI;AI;Gain	P1_C3;P6_C1;P6_C1	Complete;Complete;Con	AGO4, AGO1, AK025726, AG		5	0
chr19:44,469,699-44,485,636	15,937	q13.31	10	2	Gain	P1_C3	Complete	ZNF221		1	0
chr3:77,150,133-77,325,579	175,446	p12.3	10	2	Loss	P1_C3	Complete	ROBO2		1	0
chrX:47,084,260-47,425,413	341,153	p11.23	10	2	Loss	P1_C4	Complete	CDK16, USP11, SNORA11C, Z		7	0
chr19:44,790,306-44,833,575	43,269	q13.31	10	2	Gain	P1_C3	Complete	ZNF235, ZNF112, ZFP112		3	0
chr1:68,405,797-69,622,172	1,216,375	p31.3 - p31.2	10	2	AI	P1_C3	Complete	GNG12-AS1, DIR	hsa-mir-1262	10	1
chr1:66,170,681-66,269,188	98,507	p31.3	10	2	Loss	P1_C3	Complete	PDE4B		1	0
chr1:61,223,186-62,224,719	1,001,533	p32.1 - p31.3	10	2	AI	P1_C3	Complete	LOC101926964, AK097193, N		10	0
chr1:35,479,290-35,902,952	423,662	p34.3	10	2	Gain	P6_C1	Complete	ZMYM6, ZMYM1, BX537811,		9	0
chr1:38,751,352-38,917,393	166,041	p34.3	10	2	Loss	P1_C3	Complete			0	0
chr1:64,076,996-64,541,466	464,470	p31.3	10	2	AI	P1_C3	Complete	PGM1, Mir_544, ROR1		3	0
chr6:40,126,876-40,347,588	220,712	p21.2	10	2	Loss	P7_C1	Complete	LINC00951, DQ571907, TDRC		3	0
chr3:126,868,645-126,972,634	103,989	q21.3	10	2	Loss	P7_C1	Complete	C3orf56		1	0
chr20:24,952,121-24,995,906	43,785	p11.21	10	2	Loss	P7_C1	Complete	APMAP, ACSS1		2	0
chr1:26,921,861-27,178,392	256,531	p36.11	10	1	Loss	P6_C1	Complete	LOC101928728, ARID1A, PIG		5	0
chrX:13,235,668-13,983,814	748,146	p22.2	10	1	Loss	P1_C4	Complete	LINC02154, GS1-600G8.3, LO		14	0
chr9:104,152,584-104,184,097	31,513	q31.1	10	1	Gain	P1_C3	Complete	MRPL50, ZNF189, ALDOB		3	0
chrX:2,576,718-2,695,521	118,803	p22.33	10	1	Loss	P1_C4	Complete	CD99, XGY2, XG		3	0
chr17:57,785,690-57,856,830	71,140	q23.1	10	1	Gain	P6_C1	Complete	VMP1		1	0
chr3:76,932,116-77,074,810	142,694	p12.3	10	1	Loss	P1_C3	Complete	ROBO2		1	0
chrX:71,338,015-71,394,240	56,225	q13.1	10	1	AI	P1_C4	Complete	NHSL2, RTL5, RGAG4, FLJ44		4	0
chr1:67,555,967-68,029,315	473,348	p31.3	10	1	AI	P1_C3	Complete	C1orf141, IL23R, IL12RB2, SE		4	0
chr15:50,836,810-50,972,610	135,800	q21.2	10	1	Gain	P7_C1	Complete	USP50, TRPM7, TRPM7		3	0
chr14:101,539,257-101,636,468	97,211	q32.31	10	1	Loss	P7_C1	Complete	MEG9, BC148240, LINC02285		3	0
chr1:66,543,232-66,621,739	78,507	p31.3	10	1	Loss	P1_C3	Complete	PDE4B, U4		2	0
chr1:82,041,786-82,876,615	834,829	p31.1	10	1	AI	P1_C3	Complete	LPHN2, ADGRL2, Mir_544		3	0
chr8:69,349,964-69,503,478	153,514	q13.2	10	1	Gain	P1_C3	Complete	C8orf34		1	0
chr1:66,621,739-67,263,282	641,543	p31.3	10	1	AI	P1_C3	Complete	PDE4B, MIR3117	hsa-mir-3117	5	1
chr5:134,610,869-134,830,296	219,427	q31.1	10	1	Loss	P7_C1	Complete	C5orf66, LOC100996485, AX7		7	0
chr6:169,334,689-169,438,747	104,058	q27	10	1	Loss	P7_C1	Complete	LOC105378146, LOC10192946		2	0
chr1:36,055,940-36,172,748	116,808	p34.3	10	1	Gain	P6_C1	Complete	TFAP2E, PSMB2		2	0
chr10:80,481,660-80,762,906	281,246	q22.3	10	1	Loss	P7_C1	Complete	AX747983, ZMIZ1-AS1		2	0
chrX:9,690,318-9,814,533	124,215	p22.2	10	1	Loss	P1_C4	Complete	GPR143, SHROOM2		2	0
chr1:63,023,781-64,001,362	977,581	p31.3	10	1	AI	P1_C3	Complete	DOCK7, ANGPTL3, ATG4C, I		13	0
chr1:52,854,534-53,183,173	328,639	p32.3	10	1	AI	P1_C3	Complete	ORC1, PRPF38A, TUT4, ZCCH		9	0
chrX:109,697,785-111,200,966	1,503,181	q23	10	1	Loss	P1_C4	Complete	RGAG1, RTL9, TDGF1P3, CH		14	0
chr1:67,555,967-67,772,557	216,590	p31.3	10	0	Loss	P1_C3	Complete	C1orf141, IL23R		2	0
chrX:68,624,022-69,478,500	854,478	q13.1	10	0	AI;Loss	P1_C4;P1_C4	Complete;Complete	FAM155B, EDA, MIR676, AW		10	0
chr1:42,046,315-42,524,413	478,098	p34.2	10	0	AI	P1_C3	Complete	HIVEP3		1	0
chr1:82,876,615-82,987,363	110,748	p31.1	10	0	Loss	P1_C3	Complete			0	0
chr17:37,570,588-37,672,348	101,760	q12	10	0	Gain	P6_C1	Complete	MED1, CDK12		2	0
chr12:122,967,411-123,060,114	92,703	q24.31	10	0	Gain	P7_C1	Complete	ZCCHC8, SNORA9, RSRC2, K		4	0
chr2:119,696,304-119,751,928	55,624	q14.2	10	0	Loss	P7_C1	Complete	MARCO		1	0
chrX:119,509,834-119,703,279	193,445	q24	10	0	Loss	P1_C4	Complete	ATP1B4, LAMP2, CUL4B		3	0
chr8:105,071,716-105,213,705	141,989	q22.3	10	0	Loss	P1_C3	Complete	RIMS2		1	0
chr10:131,426,841-131,696,690	269,849	q26.3	10	0	Loss	P7_C1	Complete	MGMT, MIR4297	hsa-mir-4297	3	1
chr12:114,552,977-114,615,733	62,756	q24.21	10	0	Loss	P7_C1	Complete			0	0
chr19:44,345,900-44,418,841	72,941	q13.31	10	0	Gain	P1_C3	Complete	ZNF283, ZNF404, LOC100505		4	0
chr2:71,839,801-71,912,130	72,329	p13.2	10	0	Loss	P7_C1	Complete	DYSF		1	0
chr17:35,917,877-36,031,415	113,538	q12	10	0	Gain	P6_C1	Complete	SYNRG, MIR378J, DDX52		3	0
chr1:84,895,911-84,935,738	39,827	p31.1 - p22.3	10	0	Loss	P1_C3	Complete			0	0

chr1:112,546,269-112,571,200	24,931	p13.2	10	0	Loss	P7_C1	Complete			0	0
chr1:18,806,851-18,808,014	1,163	p36.13	10	0	Loss	P1_C3	Complete	KLHDC7A		1	0
chr1:19,011,666-19,076,861	65,195	p36.13	10	0	Loss	P1_C3	Complete	PAX7		1	0
chr1:20,456,280-20,475,649	19,369	p36.12	10	0	Loss	P1_C3	Complete	PLA2G2F		1	0
chr1:21,012,602-21,016,228	3,626	p36.12	10	0	Loss	P1_C3	Complete	KIF17		1	0
chr1:21,048,082-21,070,820	22,738	p36.12	10	0	Loss	P1_C3	Complete	SH2D5, HP1BP3		2	0
chr1:22,909,649-22,922,297	12,648	p36.12	10	0	Loss	P1_C3	Complete	EPHA8		1	0
chr1:23,738,696-23,759,728	21,032	p36.12	10	0	Loss	P1_C3	Complete	TCEA3, ASAP3		2	0
chr1:24,433,055-24,435,829	2,774	p36.11	10	0	Loss	P1_C3	Complete	MYOM3		1	0
chr1:24,478,080-24,488,271	10,191	p36.11	10	0	Loss	P1_C3	Complete	IFNLR1		1	0
chr1:35,380,394-35,479,290	98,896	p34.3	10	0	Loss	P1_C3	Complete	DLGAP3, LOC653160, TMEM		5	0
chr1:36,553,483-36,625,781	72,298	p34.3	10	0	Gain	P6_C1	Complete	TEKT2, ADPRHL2, COL8A2,		5	0
chr1:39,876,129-39,884,102	7,973	p34.3	10	0	Loss	P1_C3	Complete	MACF1, KIAA0754		2	0
chr1:42,024,588-42,046,315	21,727	p34.2	10	0	Loss	P1_C3	Complete	HIVEP3		1	0
chr1:42,524,413-42,616,276	91,863	p34.2	10	0	Loss	P1_C3	Complete			0	0
chr1:43,785,044-43,806,113	21,069	p34.2	10	0	Loss	P1_C3	Complete	TIE1, MPL		2	0
chr1:46,743,685-46,746,180	2,495	p34.1	10	0	Loss	P1_C3	Complete	RAD54L, LRRC41		2	0
chr1:48,375,457-48,405,504	30,047	p33	10	0	Loss	P1_C3	Complete	TRABD2B		1	0
chr1:52,829,304-52,854,534	25,230	p32.3	10	0	Loss	P1_C3	Complete	CC2D1B, ORC1		2	0
chr1:53,675,769-53,683,501	7,732	p32.3	10	0	Loss	P1_C3	Complete	CPT2, C1orf123, CZIB		3	0
chr1:54,255,873-54,307,305	51,432	p32.3	10	0	Loss	P1_C3	Complete	NDC1		1	0
chr1:55,242,629-55,266,707	24,078	p32.3	10	0	Loss	P1_C3	Complete	TTC22		1	0
chr1:55,519,773-55,528,204	8,431	p32.3	10	0	Loss	P1_C3	Complete	PCSK9		1	0
chr1:57,309,182-57,383,529	74,347	p32.2	10	0	Loss	P1_C3	Complete	C8A		1	0
chr1:57,599,516-57,641,292	41,776	p32.2	10	0	Loss	P1_C3	Complete	DAB1		1	0
chr1:60,300,944-60,363,611	62,667	p32.1	10	0	Loss	P1_C3	Complete	HOOK1, CYP2J2		2	0
chr1:61,173,805-61,223,186	49,381	p32.1	10	0	Loss	P1_C3	Complete	LOC101926964, AK097193		2	0
chr1:62,224,719-62,271,228	46,509	p31.3	10	0	Loss	P1_C3	Complete	PATJ, INADL		2	0
chr1:62,658,334-62,675,845	17,511	p31.3	10	0	Loss	P1_C3	Complete	L1TD1		1	0
chr1:62,739,633-62,740,576	943	p31.3	10	0	Loss	P1_C3	Complete	KANK4		1	0
chr1:62,850,023-63,023,781	173,758	p31.3	10	0	Loss	P1_C3	Complete	USP1, DOCK7		2	0
chr1:64,001,362-64,076,996	75,634	p31.3	10	0	Loss	P1_C3	Complete	EFCAB7, DLEU2L, PGM1		3	0
chr1:66,901,573-66,957,011	55,438	p31.3	10	0	Loss	P1_C3	Complete			0	0
chr1:66,988,684-67,019,088	30,404	p31.3	10	0	Loss	P1_C3	Complete	SGIP1		1	0
chr1:68,370,341-68,405,797	35,456	p31.3	10	0	Loss	P1_C3	Complete	GNG12-AS1		1	0
chr1:69,622,172-69,682,164	59,992	p31.2	10	0	Loss	P1_C3	Complete	LINC01707		1	0
chr1:70,505,116-70,551,256	46,140	p31.1	10	0	Loss	P1_C3	Complete	LRRC7		1	0
chr1:70,885,469-70,902,004	16,535	p31.1	10	0	Loss	P1_C3	Complete	CTH		1	0
chr1:76,862,102-76,882,130	20,028	p31.1	10	0	Loss	P1_C3	Complete	ST6GALNAC3		1	0
chr1:77,943,052-78,053,358	110,306	p31.1	10	0	Loss	P1_C3	Complete	AK5, ZZZ3		2	0
chr1:81,984,467-82,041,786	57,319	p31.1	10	0	Loss	P1_C3	Complete	LOC101927434, LPHN2, ADG		3	0
chr1:85,010,878-85,037,636	26,758	p22.3	10	0	Loss	P1_C3	Complete	SPATA1, CTBS		2	0
chr2:179,456,516-179,466,174	9,658	q31.2	10	0	Loss	P7_C1	Complete	MIR548N, TTN-AS1, TTN		3	0
chr2:196,866,165-196,889,757	23,592	q32.3	10	0	Loss	P1_C3	Complete	DNAH7		1	0
chr2:238,239,548-238,253,882	14,334	q37.3	10	0	Loss	P7_C1	Complete	COL6A3		1	0
chr2:238,284,545-238,307,688	23,143	q37.3	10	0	Loss	P7_C1	Complete	COL6A3		1	0
chr2:73,676,039-73,678,971	2,932	p13.1	10	0	Gain	P1_C3	Complete	ALMS1		1	0
chr3:121,414,271-121,417,185	2,914	q13.33	10	0	Gain	P1_C3	Complete	GOLGB1		1	0
chr3:125,851,664-125,882,316	30,652	q21.3	10	0	Loss	P7_C1	Complete	ALDH1L1		1	0
chr3:167,018,528-167,028,639	10,111	q26.1	10	0	Loss	P1_C3	Complete	ZBBX		1	0
chr3:167,051,224-167,069,002	17,778	q26.1	10	0	Loss	P1_C3	Complete	ZBBX		1	0
chr3:29,892,053-29,932,801	40,748	p24.1	10	0	Gain	P1_C3	Complete	RBMS3, Mir_544		2	0
chr3:37,365,171-37,368,112	2,941	p22.2	10	0	Gain	P1_C3	Complete	GOLGA4		1	0
chr3:44,759,622-44,776,382	16,760	p21.31	10	0	Gain	P1_C3	Complete	ZNF502, ZNF501		2	0
chr3:88,103,767-88,190,781	87,014	p11.1	10	0	Gain	P1_C3	Complete	CGGBP1, ZNF654		2	0
chr4:126,390,837-126,402,865	12,028	q28.1	10	0	Gain	P1_C3	Complete	FAT4		1	0

chr5:135,223,765-135,284,779	61,014	q31.1	10	0	Loss	P7_C1	Complete	SLC25A48, IL9, FBXL21P, FB	5	0
chr5:178,306,858-178,311,255	4,397	q35.3	10	0	Gain	P1_C3	Complete	ZNF354B	1	0
chr5:178,358,292-178,361,655	3,363	q35.3	10	0	Gain	P1_C3	Complete	ZFP2	1	0
chr6:117,686,547-117,705,835	19,288	q22.1	10	0	Loss	P1_C3	Complete	ROS1, GOPC	2	0
chr6:146,260,717-146,276,271	15,554	q24.3	10	0	Gain	P1_C3	Complete	SHPRH	1	0
chr6:27,416,385-27,426,363	9,978	p22.1	10	0	Gain	P1_C3	Complete	ZNF184	1	0
chr6:46,135,313-46,139,211	3,898	p21.1	10	0	Loss	P1_C3	Complete	ENPP5	1	0
chr6:46,657,807-46,659,906	2,099	p12.3	10	0	Loss	P1_C3	Complete	TDRD6	1	0
chr6:87,966,423-87,970,087	3,664	q14.3	10	0	Gain	P1_C3	Complete	ZNF292	1	0
chr6:97,618,872-97,679,377	60,505	q16.1	10	0	Loss	P1_C3	Complete	MMS22L, MIR548H3	2	0
chr7:106,649,484-106,666,904	17,420	q22.3	10	0	Gain	P1_C3	Complete		0	0
chr7:117,149,145-117,177,746	28,601	q31.2	10	0	Loss	P1_C3	Complete	CFTR	1	0
chr7:5,101,081-5,107,231	6,150	p22.1	10	0	Gain	P1_C3	Complete	RBAK-RBAKDN, RBAK	2	0
chr7:91,622,253-91,631,303	9,050	q21.2	10	0	Gain	P1_C3	Complete	AKAP9	1	0
chr7:92,731,789-92,736,577	4,788	q21.2	10	0	Gain	P1_C3	Complete	SAMD9	1	0
chr8:110,421,287-110,442,154	20,867	q23.1	10	0	Loss	P1_C3	Complete	PKHD1L1	1	0
chr8:12,877,594-12,879,237	1,643	p22	10	0	Loss	P1_C3	Complete	TRMT9B, KIAA1456	2	0
chr8:125,988,984-125,989,909	925	q24.13	10	0	Gain	P1_C3	Complete	ZNF572	1	0
chr8:133,898,916-133,906,154	7,238	q24.22	10	0	Loss	P7_C1	Complete	TG	1	0
chr9:95,594,959-95,610,711	15,752	q22.31	10	0	Gain	P1_C3	Complete	ANKRD19P, ZNF484	2	0
chr10:50,530,713-50,532,747	2,034	q11.23	10	0	Loss	P7_C1	Complete	C10orf71	1	0
chr10:60,369,809-60,415,296	45,487	q21.1	10	0	Gain	P1_C3	Complete	BICC1	1	0
chr11:102,660,761-102,663,452	2,691	q22.2	10	0	Loss	P1_C3	Complete	MMP1, WTAPP1	2	0
chr12:32,134,535-32,137,538	3,003	p11.21	10	0	Gain	P1_C3	Complete	KIAA1551, RESF1	2	0
chr12:80,733,458-80,761,420	27,962	q21.31	10	0	Loss	P1_C3	Complete	OTOGL	1	0
chr12:85,406,598-85,425,717	19,119	q21.31	10	0	Loss	P1_C3	Complete	TSPAN19	1	0
chr13:65,199,451-65,223,125	23,674	q21.31	10	0	Loss	P1_C3	Complete		0	0
chr14:60,903,046-60,933,593	30,547	q23.1	10	0	Loss	P1_C3	Complete	C14orf39	1	0
chr16:71,481,155-71,484,833	3,678	q22.2	10	0	Gain	P1_C3	Complete	ZNF23	1	0
chr16:86,478,529-86,501,120	22,591	q24.1	10	0	Loss	P7_C1	Complete		0	0
chr17:38,398,281-38,483,714	85,433	q21.1 - q21.2	10	0	Gain	P6_C1	Complete	WIPF2, CDC6, RARA	3	0
chr17:40,572,713-40,610,086	37,373	q21.2	10	0	Gain	P6_C1	Complete	PTRF, CAVIN1	2	0
chr17:42,483,189-42,643,635	160,446	q21.31	10	0	Gain	P6_C1	Complete	GPATCH8, FZD2	2	0
chr17:45,352,153-45,462,701	110,548	q21.32	10	0	Loss	P6_C1	Complete	ITGB3, THCAT158, AX74812	4	0
chr18:9,254,426-9,257,868	3,442	p11.22	10	0	Gain	P1_C3	Complete	ANKRD12	1	0
chr19:19,820,946-19,823,562	2,616	p13.11	10	0	Gain	P1_C3	Complete	ZNF14	1	0
chr19:35,434,480-35,435,611	1,131	q13.11	10	0	Gain	P1_C3	Complete	ZNF30	1	0
chr19:37,618,114-37,677,948	59,834	q13.12	10	0	Gain	P1_C3	Complete	ZNF420, ZNF585A, ZNF585B	3	0
chr19:37,877,473-37,905,144	27,671	q13.12	10	0	Gain	P1_C3	Complete	ZNF527, ZNF569	2	0
chr19:38,055,551-38,056,962	1,411	q13.12	10	0	Gain	P1_C3	Complete	ZNF571-AS1, ZNF571, ZNF54	3	0
chr19:38,123,246-38,164,432	41,186	q13.12	10	0	Gain	P1_C3	Complete	ZFP30, ZNF781	2	0
chr19:44,660,578-44,662,056	1,478	q13.31	10	0	Gain	P1_C3	Complete	ZNF234	1	0
chr19:44,680,062-44,740,684	60,622	q13.31	10	0	Gain	P1_C3	Complete	ZNF226, ZNF227, ZNF235	3	0
chr19:52,824,893-52,839,988	15,095	q13.41	10	0	Gain	P1_C3	Complete	ZNF480, AK097759, ZNF610	3	0
chr19:56,880,280-56,954,002	73,722	q13.43	10	0	Gain	P1_C3	Complete	ZNF542, ZNF542P, ZNF582, Z	6	0
chr19:57,036,336-57,047,528	11,192	q13.43	10	0	Gain	P1_C3	Complete	ZNF471	1	0
chr20:45,126,594-45,137,134	10,540	q13.12	10	0	Gain	P1_C3	Complete	ZNF334	1	0
chr20:54,041,419-54,158,579	117,160	q13.2	10	0	Loss	P6_C1	Complete	LINC01441, LINC01440	2	0
chrX:10,105,769-10,176,906	71,137	p22.2	10	0	Loss	P1_C4	Complete	WWC3, CLCN4	2	0
chrX:123,004,158-123,269,043	264,885	q25	10	0	Gain	P7_C1	Complete	XIAP, LOC101928402, STAG2	3	0
chrX:68,382,804-68,524,265	141,461	q13.1	10	0	AI;Loss	P1_C4;P1_C4	Complete;Complete	PJA1, LINC00269	2	0
chrX:9,814,533-9,862,984	48,451	p22.2	10	0	AI	P1_C4	Complete	SHROOM2	1	0
chrX:9,862,984-9,865,363	2,379	p22.2	10	0	Loss	P1_C4	Complete	SHROOM2	1	0
chrX:9,941,005-10,087,293	146,288	p22.2	10	0	Loss	P1_C4	Complete	WWC3	1	0

Supplementary Table S3A. Genes affected by CNVs in 10 spinal meningiomas. Gene ID; chromosome number; gene genomic coordinates (hg19); and type of CNV (gain, loss or allelic imbalance (AI) in each individual tumor are shown from left to right.

Gene ID	Chr	Position	P1_S1	P1_S2	P1_S3	P1_S5	P2_S1	P2_S2	P4_S1	P4_S2	P4_S3	P5_S1
ABL2	1	179,068,461-179,198,819										Gain
AF119915	1	35,833,677-35,835,012										Gain
AKR7A2P1	1	113,465,971-113,467,295										Gain
ARID4B	1	235,294,497-235,491,534										Gain
ARNT	1	150,782,180-150,849,244										Gain
ATP5IF1	1	28,562,601-28,564,616										Gain
ATPIF1	1	28,562,601-28,564,616										Gain
AX748204	1	23,853,364-23,855,542										Gain
CLSTN1	1	9,789,078-9,884,584										Gain
CTNNBIP1	1	9,908,333-9,970,316										Gain
CTSK	1	150,768,683-150,780,917										Gain
CTSS	1	150,702,671-150,738,433										Gain
CTXND2	1	150,859,728-150,884,988										Gain
DD413682	1	179,170,962-179,170,982										Gain
E2F2	1	23,832,919-23,857,712										Gain
EPB41	1	29,213,602-29,446,558										Gain
EYA3	1	28,296,854-28,415,148										Gain
GGPS1	1	235,490,664-235,507,844										Gain
GMEB1	1	28,995,239-29,042,115										Gain
GOLPH3L	1	150,618,700-150,669,672										Gain
GPX7	1	53,068,042-53,074,723										Gain
HORMAD1	1	150,670,534-150,693,364										Gain
ID3	1	23,884,420-23,886,285										Gain
INADL	1	62,208,148-62,644,347										Gain
JA611241	1	28,567,542-28,567,564										Gain
KIF1B	1	10,270,627-10,441,661										Gain
LINC01715	1	28,969,767-28,975,311										Gain
LOC113391335	1	10,010,629-10,020,845										Gain
LZIC	1	9,982,170-10,003,485										Gain
MED18	1	28,655,512-28,662,478										Gain

MIR5697	1	10,027,438-10,027,516										Gain
MIR5698	1	154,076,996-154,077,068										Gain
MRPS21	1	150,266,261-150,281,414										Gain
NMNAT1	1	10,002,980-10,045,556										Gain
NUP210L	1	153,965,167-154,127,592										Gain
OPRD1	1	29,138,653-29,190,208										Gain
PATJ	1	62,208,137-62,629,591										Gain
PHACTR4	1	28,696,064-28,826,881										Gain
PRPF3	1	150,293,916-150,325,704										Gain
RAB42	1	28,918,711-28,921,955										Gain
RBP7	1	10,057,254-10,076,078										Gain
RCC1	1	28,832,454-28,865,708										Gain
RNU11	1	28,975,111-28,975,246										Gain
RNU6-2	1	10,359,023-10,359,128										Gain
RPRD2	1	150,336,586-150,449,041										Gain
SCARNA24	1	29,016,176-29,016,306										Gain
SESN2	1	28,585,962-28,609,002										Gain
SLC16A1	1	113,454,469-113,498,975										Gain
SNHG12	1	28,905,049-28,909,495										Gain
SNHG3	1	28,832,454-28,837,404										Gain
SNORA16A	1	28,907,431-28,907,565										Gain
SNORA44	1	28,906,892-28,907,024										Gain
SNORA61	1	28,906,275-28,906,405										Gain
SNORA73B	1	28,835,069-28,835,274										Gain
SNORD99	1	28,905,254-28,905,334										Gain
SOAT1	1	179,262,848-179,327,814										Gain
TAF12	1	28,929,608-28,969,604										Gain
TBCE	1	235,530,674-235,612,283										Gain
TRNAU1AP	1	28,879,528-28,905,057										Gain
TUT4	1	52,888,947-53,018,762										Gain
UBE4B	1	10,093,040-10,241,296										Gain
YTHDF2	1	29,063,132-29,096,287										Gain
ZBTB8A	1	32,930,657-33,071,551										Gain
ZBTB8OS	1	33,065,772-33,116,533										Gain

ZCCHC11	1	52,888,947-53,019,130										Gain
ZMYM4	1	35,734,567-35,887,545										Gain
ZMYM4-AS1	1	35,824,424-35,831,678										Gain
ZYG11A	1	53,308,182-53,360,247										Gain
ZYG11B	1	53,192,130-53,293,013										Gain
AK093214	1	175,526,123-175,533,005										Loss
BC042538	1	30,486,798-30,510,456										Loss
BC043291	1	175,526,123-175,537,388										Loss
CSMD2	1	33,979,598-34,631,443										Loss
CSMD2-AS1	1	34,334,556-34,351,059										Loss
HIVEP3	1	41,972,035-42,501,596										Loss
IGFN1	1	201,159,952-201,198,080										Loss
KCNC4	1	110,753,335-110,776,674										Loss
KCND3	1	112,318,453-112,531,777										Loss
LINC01648	1	30,486,798-30,510,459										Loss
LINC01750	1	112,533,157-112,541,463										Loss
LINC02586	1	110,751,072-110,752,609										Loss
LOC402779	1	34,334,556-34,351,059										Loss
LOC643355	1	112,533,189-112,541,463										Loss
SLC6A17	1	110,693,135-110,744,823										Loss
TNR	1	175,291,934-175,712,752										Loss
TRABD2B	1	48,226,199-48,462,562										Loss
ABI2	2	204,192,961-204,296,896										Gain
ACTR3BP2	2	92,129,158-92,130,496										Gain
ALS2CR8	2	203,776,977-203,851,060										Gain
BIRC6	2	32,582,095-32,843,965										Gain
BMPR2	2	203,241,049-203,432,474										Gain
CARF	2	203,776,940-203,852,984										Gain
CCDC88A	2	55,514,977-55,647,057										Gain
CCT4	2	62,095,261-62,115,806										Gain
COMMD1	2	62,115,857-62,363,205										Gain
CYP20A1	2	204,103,163-204,170,563										Gain
FAM117B	2	203,499,900-203,634,480										Gain
FAM161A	2	62,051,982-62,081,278										Gain

GGT8P	2	91,963,367-91,970,153										Gain
ICA1L	2	203,637,872-203,736,708										Gain
KIAA1841	2	61,293,004-61,365,169										Gain
KIAA2012	2	202,937,975-203,069,924										Gain
LOC100652824	2	202,937,977-203,061,886										Gain
LOC101927050	2	91,887,713-91,900,136										Gain
LOC654342	2	91,805,187-91,847,975										Gain
LRPPRC	2	44,113,362-44,223,144										Gain
LSP1P4	2	91,824,708-91,847,975										Gain
MTIF2	2	55,463,755-55,496,384										Gain
NBEAL1	2	203,879,601-204,091,101										Gain
NLRC4	2	32,449,517-32,490,812										Gain
NOP58	2	203,130,438-203,168,384										Gain
PEX13	2	61,244,696-61,279,125										Gain
PRORSD1P	2	55,509,454-55,511,607										Gain
PUS10	2	61,167,547-61,245,394										Gain
RAPH1	2	204,298,404-204,400,059										Gain
SLC30A6	2	32,390,909-32,449,448										Gain
SNORA70B	2	61,644,378-61,644,512										Gain
SNORD11	2	203,157,773-203,157,857										Gain
SNORD11B	2	203,156,039-203,156,151										Gain
SNORD70	2	203,141,153-203,141,241										Gain
SNORD70	2	203,142,830-203,142,915										Gain
SNORD70B	2	203,142,835-203,142,910										Gain
SPAST	2	32,288,679-32,382,706										Gain
SUMO1	2	203,070,902-203,103,322										Gain
USP34	2	61,453,924-61,507,483										Gain
USP34	2	61,414,589-61,697,849										Gain
WDR12	2	203,745,322-203,776,949										Gain
XPO1	2	61,705,068-61,765,418										Gain
YIPF4	2	32,502,957-32,531,658										Gain
ACP1	2	264,868-278,282										Loss
ALKAL2	2	279,560-288,308										Loss
ANKMY1	2	241,418,838-241,500,527										Loss

APOB	2	21,224,300-21,266,945									Loss
BC101234	2	242,945,877-242,948,160									Loss
CAPN10	2	241,526,132-241,557,122									Loss
CAPN10-DT	2	241,522,166-241,525,733									Loss
COL6A3	2	238,232,654-238,322,850									Loss
COPS9	2	241,065,979-241,075,764									Loss
DUSP28	2	241,499,470-241,503,431									Loss
DYSF	2	71,680,752-71,913,893									Loss
FAM110C	2	38,813-46,588									Loss
FAM150B	2	279,560-288,308									Loss
GCA	2	163,175,272-163,219,618									Loss
GPC1	2	241,375,114-241,407,495									Loss
GPR35	2	241,544,824-241,570,676									Loss
HDAC4	2	239,969,863-240,323,346									Loss
HDAC4-AS1	2	240,323,129-240,324,058									Loss
IL1RL2	2	102,803,432-102,856,461									Loss
KCNH7	2	163,227,916-163,695,257									Loss
LINC01237	2	242,823,513-243,020,873									Loss
LINC01865	2	316,283-337,054									Loss
LINC01880	2	242,989,834-243,026,289									Loss
LINC01881	2	243,030,783-243,102,476									Loss
LOC100130449	2	241,388,826-241,396,131									Loss
LOC285097	2	242,943,359-242,948,162									Loss
LOC728323	2	243,030,843-243,102,469									Loss
MGC16025	2	240,115,026-240,117,153									Loss
MIR149	2	241,395,417-241,395,506									Loss
MIR2467	2	240,273,418-240,273,499									Loss
MIR4269	2	240,227,156-240,227,240									Loss
MYEOV2	2	241,065,979-241,075,764									Loss
NDUFA10	2	240,831,867-240,964,819									Loss
OR6B2	2	240,968,381-240,972,330									Loss
OTOS	2	241,078,445-241,080,073									Loss
PP14571	2	241,388,835-241,396,117									Loss
RAD51AP2	2	17,691,850-17,711,681									Loss

RNPEPL1	2	241,507,900-241,518,149										Loss
RSAD2	2	7,017,795-7,038,363										Loss
SH3YL1	2	218,135-264,866										Loss
SNTG2	2	946,553-1,371,384										Loss
TMEM18	2	663,877-677,468										Loss
TNS1	2	218,664,511-218,867,718										Loss
TPO	2	1,377,994-1,546,499										Loss
ADAMTS9	3	64,501,330-64,673,365					AI	AI				
ARL13B	3	93,698,982-93,774,522										Gain
AY940074	3	169,858,399-169,859,958										Gain
BC131773	3	169,895,949-169,898,340										Gain
C3orf84	3	49,215,068-49,229,291										Gain
CCDC36	3	49,235,860-49,295,537										Gain
DHX30	3	47,844,398-47,891,686										Gain
DNAH12	3	57,327,726-57,530,071										Gain
KLHDC8B	3	49,209,017-49,213,919										Gain
LOC646498	3	49,215,068-49,229,291										Gain
MAP4	3	47,892,179-48,130,769										Gain
PAK2	3	196,466,727-196,559,518										Gain
PHC3	3	169,805,367-169,899,537										Gain
PRKCI	3	169,940,219-170,023,770										Gain
PROS1	3	93,591,880-93,692,934										Gain
SENP5	3	196,594,726-196,661,584										Gain
SLMAP	3	57,742,982-57,915,741										Gain
SMARCC1	3	47,627,377-47,823,596										Gain
SNORD146	3	47,703,054-47,703,184										Gain
STAG1	3	136,055,998-136,471,245										Gain
TFDP2	3	141,663,269-141,868,386										Gain
ATP2B2	3	10,365,706-10,749,716										Loss
ATP2B2-IT2	3	10,667,700-10,668,492										Loss
BC015846	3	127,041,149-127,109,513										Loss
C3orf56	3	126,911,973-126,917,027										Loss
CHCHD6	3	126,423,062-126,679,263										Loss
CLSTN2	3	139,654,026-140,286,919										Loss

LINC00606	3	10,801,026-10,805,894										Loss
LINC02016	3	127,041,149-127,109,513										Loss
PLXNA1	3	126,707,436-126,756,235										Loss
PRR20G	3	127,002,626-127,006,917										Loss
SLC6A11	3	10,857,884-10,982,419										Loss
SSUH2	3	8,661,085-8,786,726										Loss
APELA	4	165,798,139-165,818,679										Gain
BC040333	4	39,640,759-39,653,681										Gain
DCUN1D4	4	52,709,165-52,783,003										Gain
ELF2	4	139,978,412-140,098,372										Gain
KLB	4	39,408,472-39,453,153										Gain
LIAS	4	39,460,643-39,479,271										Gain
LOC100505989	4	165,675,282-165,724,947										Gain
LOC100506013	4	165,798,155-165,818,676										Gain
LOC344967	4	40,044,536-40,058,819										Gain
LOC401127	4	39,481,874-39,483,523										Gain
MIR5591	4	39,413,529-39,413,594										Gain
N4BP2	4	40,058,523-40,159,872										Gain
NSUN7	4	40,751,913-40,813,199										Gain
PDS5A	4	39,824,482-39,979,576										Gain
RBM47	4	40,425,271-40,632,640										Gain
RPL9	4	39,455,744-39,460,568										Gain
SMIM14	4	39,548,916-39,640,618										Gain
SMIM31	4	165,675,282-165,724,947										Gain
UBE2K	4	39,699,663-39,784,410										Gain
UGDH	4	39,500,374-39,529,218										Gain
UGDH-AS1	4	39,529,458-39,596,327										Gain
DQ572409	4	7,608,584-7,608,614										Loss
DQ573667	4	10,127,121-10,127,151										Loss
DQ581767	4	10,124,402-10,124,435										Loss
DQ583133	4	10,122,826-10,122,861										Loss
DQ584082	4	10,125,235-10,125,264										Loss
DQ589421	4	10,122,105-10,122,135										Loss
DQ591515	4	10,137,431-10,137,474										Loss

DQ599898	4	10,145,243-10,145,285										Loss
MIR3138	4	10,080,234-10,080,316										Loss
MIR4274	4	7,461,754-7,461,845										Loss
PSAPL1	4	7,432,020-7,436,700										Loss
SLC2A9	4	9,827,847-10,041,872										Loss
SORCS2	4	7,194,373-7,744,564										Loss
WDR1	4	10,086,066-10,094,779										Loss
WDR1	4	10,075,962-10,118,658										Loss
AK6	5	68,647,552-68,665,840										Gain
C5orf42	5	37,106,329-37,249,530										Gain
CCDC125	5	68,576,518-68,628,620										Gain
CDK7	5	68,530,621-68,573,257										Gain
CENPH	5	68,485,374-68,506,184										Gain
CPLANE1	5	37,106,329-37,249,530										Gain
DNAJC18	5	138,745,891-138,775,214										Gain
ECSCR	5	138,778,004-138,842,328										Gain
H3.Y	5	17,655,134-17,655,747										Gain
LINC02111	5	17,379,014-17,387,419										Gain
LINC02217	5	17,404,127-17,441,803										Gain
LINC02218	5	17,444,118-17,484,055										Gain
LOC105374727	5	37,249,147-37,250,748										Gain
LOC401177	5	17,379,014-17,387,419										Gain
MRPS36	5	68,513,572-68,525,985										Gain
MTMR12	5	32,227,110-32,313,114										Gain
NUP155	5	37,291,734-37,371,228										Gain
RAD17	5	68,665,123-68,710,630										Gain
SMIM33	5	138,850,588-138,853,285										Gain
TAF9	5	68,647,552-68,665,840										Gain
TMEM173	5	138,855,117-138,862,343										Gain
ZFR	5	32,354,451-32,444,867										Gain
AHRR	5	304,290-438,405										Loss
AX747819	5	134,670,617-134,672,992										Loss
BRD9	5	863,849-892,939										Loss
C5orf20	5	134,779,903-134,783,038										Loss

C5orf66	5	134,368,969-134,680,370										Loss
C5orf66-AS2	5	134,571,923-134,583,867										Loss
CCDC127	5	204,874-218,297										Loss
DCANP1	5	134,779,903-134,783,038										Loss
H2AFY	5	134,670,070-134,735,577										Loss
HRAT5	5	269,670-271,639										Loss
ICE1	5	5,422,785-5,490,347										Loss
KIAA0947	5	5,422,785-5,490,347										Loss
LOC100996485	5	134,368,969-134,680,370										Loss
LOC340073	5	134,571,923-134,583,867										Loss
LRRC14B	5	191,625-196,449										Loss
NEUROG1	5	134,869,971-134,871,639										Loss
PDCD6	5	271,735-315,089										Loss
PLEKHG4B	5	92,265-190,087										Loss
SDHA	5	218,337-257,197										Loss
TIFAB	5	134,784,557-134,788,089										Loss
TRIP13	5	892,968-918,164										Loss
ZDHHC11	5	795,719-851,101										Loss
ZDHHC11B	5	710,469-784,870										Loss
AMD1	6	111,135,823-111,216,915										Gain
BC047513	6	111,133,327-111,136,191										Gain
BEND3	6	107,386,384-107,435,636										Gain
CASC15	6	21,666,643-22,196,049										Gain
CDK19	6	110,931,180-111,137,088										Gain
CGAS	6	74,134,855-74,162,043										Gain
DDX43	6	74,104,284-74,127,289										Gain
DL491467	6	74,228,125-74,228,181										Gain
DQ590492	6	74,228,271-74,228,299										Gain
EEF1A1	6	74,225,472-74,230,755										Gain
GTF3C6	6	111,279,762-111,289,091										Gain
LINC00340	6	21,665,002-22,214,734										Gain
MB21D1	6	74,134,855-74,162,043										Gain
MTO1	6	74,171,453-74,211,179										Gain
PDSS2	6	107,473,760-107,780,779										Gain

RPF2	6	111,303,219-111,349,466										Gain
SLC17A5	6	74,303,101-74,363,737										Gain
SNORA40C	6	111,169,748-111,169,876										Gain
SNORD141A	6	74,227,967-74,228,161										Gain
SNORD141B	6	74,227,967-74,228,161										Gain
AK023627	6	170,149,607-170,151,679										Loss
APOBEC2	6	41,020,939-41,032,630										Loss
C6orf120	6	170,102,207-170,106,453										Loss
C6orf70	6	170,151,720-170,181,617										Loss
DQ571907	6	40,346,131-40,346,176										Loss
ERMARD	6	170,151,717-170,181,680										Loss
LINC00951	6	40,312,083-40,323,745										Loss
LOC101929523	6	169,613,306-169,639,657										Loss
LOC101929555	6	40,846,624-40,991,019										Loss
LRFN2	6	40,359,372-40,555,203										Loss
NFYA	6	41,040,706-41,070,146										Loss
OARD1	6	41,034,526-41,065,526										Loss
PHF10	6	170,104,001-170,124,106										Loss
TCTE3	6	170,140,210-170,151,679										Loss
TDRG1	6	40,346,075-40,347,638										Loss
THBS2	6	169,615,874-169,654,209										Loss
TSPO2	6	41,010,236-41,012,076										Loss
UNC5CL	6	40,994,639-41,039,015										Loss
WDR27	6	169,857,302-170,102,159										Loss
AK096766	7	38,342,348-38,344,378	Gain	Gain	Gain	Gain	Gain	Gain	Gain	Gain	Gain	Gain
TARP	7	38,279,180-38,357,589	Gain	Gain	Gain	Gain	Gain	Gain	Gain	Gain	Gain	Gain
TCRGC2	7	38,288,843-38,356,709	Gain	Gain	Gain	Gain	Gain	Gain	Gain	Gain	Gain	Gain
TCRGV	7	38,339,411-38,339,881	Gain	Gain	Gain	Gain	Gain	Gain	Gain	Gain	Gain	Gain
TRGC2	7	38,279,180-38,356,821	Gain	Gain	Gain	Gain	Gain	Gain	Gain	Gain	Gain	Gain
AX748042	7	142,423,393-142,426,272	Gain	Gain	Gain	Gain	Gain	Gain	Gain	Gain	Gain	
BV02S1J2.2	7	142,334,185-142,334,912	Gain	Gain	Gain	Gain	Gain	Gain	Gain	Gain	Gain	
BV03S1J2.2	7	142,428,689-142,499,111	Gain	Gain	Gain	Gain	Gain	Gain	Gain	Gain	Gain	
BV17S1A1T	7	142,326,946-142,327,046	Gain	Gain	Gain	Gain	Gain	Gain	Gain	Gain	Gain	
MTRNR2L6	7	142,374,130-142,375,525	Gain	Gain	Gain	Gain	Gain	Gain	Gain	Gain	Gain	

TCRBV10S1P	7	142,344,413-142,344,891	Gain	Gain	Gain	Gain	Gain	Gain	Gain	Gain	Gain	
TCRBV11S1A1T	7	142,378,608-142,379,075	Gain	Gain	Gain	Gain	Gain	Gain	Gain	Gain	Gain	
TCRBV14S1	7	142,423,182-142,423,934	Gain	Gain	Gain	Gain	Gain	Gain	Gain	Gain	Gain	
TCRBV15S1	7	142,364,199-142,364,707	Gain	Gain	Gain	Gain	Gain	Gain	Gain	Gain	Gain	
TCRBV17S1A1T	7	142,326,375-142,327,044	Gain	Gain	Gain	Gain	Gain	Gain	Gain	Gain	Gain	
TCRBV19S1P	7	142,353,907-142,428,289	Gain	Gain	Gain	Gain	Gain	Gain	Gain	Gain	Gain	
TCRBV2S1	7	142,334,185-142,494,579	Gain	Gain	Gain	Gain	Gain	Gain	Gain	Gain	Gain	
TCRBV3S1	7	142,428,486-142,428,988	Gain	Gain	Gain	Gain	Gain	Gain	Gain	Gain	Gain	
TCRBV5S1A1T	7	142,020,708-142,470,314	Gain	Gain	Gain	Gain	Gain	Gain	Gain	Gain	Gain	
TCRVB	7	142,344,413-142,344,880	Gain	Gain	Gain	Gain	Gain	Gain	Gain	Gain	Gain	
TCRVB	7	142,353,890-142,500,213	Gain	Gain	Gain	Gain	Gain	Gain	Gain	Gain	Gain	
TRBV	7	142,334,824-142,334,914	Gain	Gain	Gain	Gain	Gain	Gain	Gain	Gain	Gain	
RBAK	7	5,085,451-5,109,119						Gain				
RBAK-RBAKDN	7	5,023,301-5,112,854						Gain				
TCRGV	7	38,402,460-38,403,119						Gain				
14-Sep	7	55,861,236-55,930,482										Gain
ABHD11	7	73,150,424-73,153,197										Gain
ABHD11-AS1	7	73,149,398-73,150,330										Gain
ADCK2	7	140,372,952-140,395,745										Gain
AK098095	7	140,419,137-140,421,887										Gain
ASL	7	65,540,775-65,558,329										Gain
AX747594	7	75,935,608-75,937,653										Gain
BC035176	7	56,601,922-56,605,823										Gain
BC036258	7	56,549,797-56,550,988										Gain
BC063788	7	75,914,374-75,916,618										Gain
BC065735	7	140,405,000-140,406,437										Gain
BRAF	7	140,419,126-140,624,728										Gain
CCL26	7	75,398,841-75,419,064										Gain
CDK13	7	39,989,958-40,136,733										Gain
CLDN3	7	73,183,326-73,184,600										Gain
CLIP2	7	73,703,804-73,820,273										Gain
CYTH3	7	6,201,411-6,312,242										Gain

DAGLB	7	6,448,746-6,523,849										Gain
DD413584	7	55,947,025-55,947,046										Gain
DKFZp434L192	7	56,563,915-56,564,977										Gain
FABP5P3	7	152,133,979-152,140,100										Gain
FAM220A	7	6,369,039-6,388,590										Gain
GBAS	7	56,032,269-56,067,875										Gain
GRID2IP	7	6,536,408-6,591,067										Gain
GTF2IRD1	7	73,868,119-74,016,920										Gain
GUSB	7	65,425,671-65,447,301										Gain
hCPE-R	7	73,242,073-73,247,014										Gain
HIP1	7	75,162,618-75,368,290										Gain
HSPB1	7	75,931,874-75,933,618										Gain
KDELR2	7	6,500,711-6,523,849										Gain
KLHDC10	7	129,710,348-129,775,560										Gain
KMT2C	7	151,832,009-152,133,090										Gain
LAT2	7	73,624,086-73,644,164										Gain
LINC00035	7	73,149,398-73,150,330										Gain
LINC01003	7	152,161,208-152,162,630										Gain
LOC100128325	7	129,781,570-129,782,458										Gain
LOC100128822	7	152,161,208-152,162,630										Gain
LOC100240728	7	56,549,797-56,551,012										Gain
LOC101928401	7	56,601,922-56,605,823										Gain
LOC650226	7	56,491,396-56,516,068										Gain
MIR4284	7	73,125,646-73,125,727										Gain
MRPS17	7	56,019,610-56,023,033										Gain
NDUFB2	7	140,396,480-140,406,446										Gain
NDUFB2-AS1	7	140,395,135-140,396,877										Gain
NIPSNAP2	7	56,032,269-56,067,875										Gain
PUS7	7	105,096,947-105,162,705										Gain
RAC1	7	6,414,125-6,443,598										Gain
RFC2	7	73,645,831-73,668,788										Gain
SRPK2	7	104,755,715-105,029,837										Gain
SRRM3	7	75,831,210-75,916,609										Gain

STX1A	7	73,113,534-73,134,017										Gain
TPST1	7	65,670,258-65,825,438										Gain
USP42	7	6,144,549-6,201,195										Gain
XRCC2	7	152,343,586-152,373,250										Gain
ZDHHC4	7	6,617,064-6,628,610										Gain
ZNF713	7	55,954,969-56,014,143										Gain
ZNF786	7	148,766,732-148,787,869										Gain
ABCA13	7	48,211,056-48,687,091										Loss
AK024243	7	149,596-154,868										Loss
AK310146	7	149,743-154,868										Loss
AX746840	7	48,313,698-48,316,321										Loss
CNPY1	7	155,293,952-155,326,539										Loss
LOC100507642	7	149,717-155,461										Loss
LOC101928807	7	131,945,619-131,949,048										Loss
LOC102723672	7	144,444-149,438										Loss
PLXNA4	7	131,808,090-132,333,447										Loss
C8orf44-SGK3	8	67,579,786-67,774,257										Gain
DPY19L4	8	95,732,102-95,806,076										Gain
MCMDC2	8	67,782,983-67,834,283										Gain
MTDH	8	98,656,411-98,742,488										Gain
PTTG3P	8	67,679,567-67,680,269										Gain
SGK3	8	67,624,652-67,774,257										Gain
AK056623	8	424,454-427,966										Loss
AK307207	8	10,340,387-10,405,095										Loss
AX747544	8	143,760,854-143,763,384										Loss
DLGAP2	8	687,604-1,656,642										Loss
ERICH1	8	564,736-681,239										Loss
ERICH1-AS1	8	687,586-1,087,777										Loss
FAM87A	8	325,930-333,174										Loss
FBXO25	8	356,807-419,875										Loss
JH8	8	143,739,812-143,745,841										Loss
JRK	8	143,738,873-143,751,412										Loss
KCNK9	8	140,613,080-140,715,299										Loss
LNCOC1	8	143,783,669-143,808,391										Loss

LOC100288181	8	143,783,669-143,808,391										Loss
LY6K	8	143,781,528-143,785,584										Loss
MIR4539	8	143,079,636-143,079,657										Loss
OR4F21	8	116,085-117,024										Loss
PRSS55	8	10,383,055-10,411,676										Loss
PSCA	8	143,751,725-143,764,145										Loss
RPL23AP53	8	158,344-182,318										Loss
TG	8	133,879,204-134,147,143										Loss
TRAPPC9	8	140,739,967-141,468,678										Loss
TSNARE1	8	143,293,440-143,484,610										Loss
ZNF596	8	182,136-197,340										Loss
AK000451	9	66,523,813-66,553,911										Gain
AK096159	9	68,743,530-68,769,869										Gain
AK308561	9	66,454,656-66,457,142										Gain
AK309896	9	66,513,679-66,553,531										Gain
AK311167	9	69,065,545-69,106,241										Gain
ANKRD20A1	9	67,926,760-67,969,840										Gain
AQP7P1	9	67,254,266-67,289,492										Gain
AX747850	9	68,795,666-68,798,213										Gain
AX748313	9	127,805,167-127,807,307										Gain
BC000869	9	66,553,275-66,555,595										Gain
BC065763	9	66,688,085-66,699,951										Gain
BC080605	9	68,413,445-68,414,929										Gain
BMS1P14	9	67,324,218-67,340,165										Gain
C9orf84	9	114,448,900-114,557,223										Gain
CBWD6	9	69,204,537-69,262,593										Gain
CR627148	9	66,457,288-66,469,310										Gain
DCAF12	9	34,086,380-34,126,771										Gain
DENND4C	9	19,230,487-19,374,266										Gain
DQ572382	9	19,200,876-19,200,903										Gain
DQ594366	9	67,792,166-67,794,649										Gain
DQ600787	9	66,368,799-66,368,828										Gain
FAM27B	9	67,792,928-67,794,189										Gain
FAM27E3	9	67,784,943-67,786,625										Gain

FNBP1	9	132,649,465-132,805,473										Gain
FOXD4L6	9	69,199,479-69,202,204										Gain
FRG1HP	9	68,726,464-68,748,376										Gain
FRG1HP	9	69,065,489-69,112,332										Gain
FRG1JP	9	68,427,783-68,455,368										Gain
GOLGA1	9	127,640,572-127,703,386										Gain
LINC00537	9	68,408,674-68,414,763										Gain
LINC01410	9	66,457,288-66,469,310										Gain
LOC100132249	9	66,490,071-66,494,170										Gain
LOC100132352	9	68,726,540-68,767,301										Gain
LOC102724580	9	67,290,450-67,334,405										Gain
LOC105379807	9	67,785,633-67,786,685										Gain
LOC286297	9	67,017,374-67,032,072										Gain
LOC403323	9	66,513,679-66,553,911										Gain
LOC440896	9	69,174,195-69,198,043										Gain
LOC642236	9	68,427,782-68,454,375										Gain
LOC728673	9	66,553,199-66,555,601										Gain
MIR1299	9	69,002,238-69,002,321										Gain
MIR3134	9	114,758,831-115,112,545										Gain
MIR4477A	9	68,415,307-68,415,388										Gain
MIR4477B	9	68,415,307-68,415,388										Gain
PGM5P2	9	69,080,243-69,147,854										Gain
PPP6C	9	127,908,851-127,952,218										Gain
PTBP3	9	114,979,994-115,095,944										Gain
PTGER4P2-CDK2AP2P2	9	66,494,268-66,503,030										Gain
RNF38	9	36,336,398-36,401,195										Gain
SCAI	9	127,704,887-127,905,838										Gain
SHOC1	9	114,448,798-114,557,288										Gain
UBAP2	9	33,921,690-34,048,947										Gain
X97876	9	66,499,190-66,500,030										Gain
COL5A1	9	137,533,650-137,736,688										Loss
FCN1	9	137,795,081-137,809,849										Loss
FCN2	9	137,772,657-137,779,366										Loss

LOC101448202	9	137,711,260-137,764,464											Loss
MIR3689A	9	137,741,020-137,742,490											Loss
MIR3689B	9	137,741,970-137,742,118											Loss
MIR3689C	9	137,741,143-137,741,215											Loss
MIR3689D1	9	137,741,454-137,741,528											Loss
MIR3689D2	9	137,742,122-137,742,202											Loss
MIR3689E	9	137,742,415-137,742,487											Loss
MIR3689F	9	137,742,587-137,742,653											Loss
PTPRD	9	8,314,245-10,612,723			Loss								
5-Mar	10	94,050,919-94,113,721											Gain
AK124121	10	94,178,242-94,180,347											Gain
AK124930	10	11,962,020-11,964,278											Gain
ANXA7	10	75,134,858-75,173,843											Gain
ATOH7	10	69,990,351-69,991,870											Gain
CACNB2	10	18,429,605-18,830,688											Gain
CAMK1D	10	12,391,541-12,877,545											Gain
CCAR1	10	70,480,900-70,552,134											Gain
CDC123	10	12,237,960-12,292,589											Gain
CFAP70	10	75,013,515-75,118,617											Gain
CPEB3	10	93,808,396-94,050,875											Gain
DHTKD1	10	12,110,915-12,165,227											Gain
DNA2	10	70,173,820-70,231,878											Gain
HERC4	10	69,681,655-69,835,103											Gain
HHEX	10	94,449,680-94,455,408											Gain
HNRNPH3	10	70,091,449-70,102,953											Gain
IDE	10	94,211,440-94,333,852											Gain
KIF11	10	94,352,824-94,415,152											Gain
MARK2P9	10	94,178,417-94,179,363											Gain
MCU	10	74,451,888-74,647,452											Gain
MICU1	10	74,127,083-74,385,949											Gain
MIR1256	10	74,119,697-74,336,541											Gain
MIR4676	10	74,480,786-74,480,858											Gain
MIR548AK	10	12,172,758-12,172,815											Gain
MYPN	10	69,865,873-69,971,773											Gain

NT5C2	10	104,847,773-104,953,063										Gain
NUDT5	10	12,207,326-12,238,143										Gain
OIT3	10	74,653,313-74,692,794										Gain
PARD3	10	34,398,487-35,104,253										Gain
PARD3-AS1	10	35,104,695-35,105,350										Gain
PBLD	10	70,042,416-70,092,684										Gain
POU5F1P5	10	69,769,703-69,770,640										Gain
RUFY2	10	70,100,863-70,167,051										Gain
SEC61A2	10	12,171,639-12,211,957										Gain
SLC25A16	10	70,242,089-70,287,280										Gain
SNORD98	10	70,514,928-70,514,995										Gain
STOX1	10	70,587,293-70,655,209										Gain
TET1	10	70,320,116-70,454,239										Gain
TTC18	10	75,013,515-75,118,617										Gain
UPF2	10	11,962,020-12,085,169										Gain
AX747983	10	80,760,240-80,763,560										Loss
C10orf71	10	50,507,186-50,535,537										Loss
C10orf71-AS1	10	50,504,327-50,506,938										Loss
CASC2	10	119,806,331-119,969,665										Loss
CFAP46	10	134,621,895-134,756,089										Loss
CYP2E1	10	135,340,866-135,374,724										Loss
DMBT1	10	124,320,180-124,403,252										Loss
DRGX	10	50,574,160-50,604,062										Loss
FAM170B	10	50,339,200-50,342,065										Loss
FAM170B-AS1	10	50,329,883-50,359,592										Loss
GRID1	10	87,359,311-88,126,250										Loss
INPP5A	10	134,351,282-134,596,984										Loss
LINC00840	10	44,354,858-44,390,881										Loss
LINC00841	10	44,404,743-44,476,713										Loss
LINC00856	10	80,008,381-80,311,112										Loss
LINC01166	10	134,757,470-134,778,793										Loss
LOC105378349	10	72,698,809-72,715,398										Loss
MIR4294	10	50,193,556-50,193,632										Loss
NKX6-2	10	134,598,319-134,599,537										Loss

PCBD1	10	72,642,037-72,648,543										Loss
RAB11FIP2	10	119,764,426-119,806,395										Loss
SGPL1	10	72,575,703-72,640,932										Loss
SLC29A3	10	73,079,009-73,123,147										Loss
SPRNP1	10	135,380,437-135,383,462										Loss
SYCE1	10	135,367,403-135,381,779										Loss
TTC40	10	134,621,895-134,756,089										Loss
UNC5B	10	72,972,291-73,062,635										Loss
VSTM4	10	50,222,289-50,323,577										Loss
WDFY4	10	50,010,782-50,032,214										Loss
WDFY4	10	49,892,920-50,191,001										Loss
ZMIZ1	10	80,828,791-81,076,285										Loss
ZMIZ1-AS1	10	80,703,082-80,828,536										Loss
AK057316	10	47,658,233-47,701,017	AI	AI	AI	AI						
ANTXRL	10	47,658,232-47,701,443	AI	AI	AI	AI						
ANTXRLP1	10	47,590,260-47,640,844	AI	AI	AI	AI						
ANXA8L1	10	47,746,849-47,763,040	AI	AI	AI	AI						
ANXA8L2	10	47,746,919-47,770,647	AI	AI	AI	AI						
FAM25BP	10	47,740,303-47,744,822	AI	AI	AI	AI						
CTNNA3	10	67,672,275-69,455,949	Loss									
AGBL2	11	47,681,142-47,736,928										Gain
C1QTNF4	11	47,611,215-47,615,961										Gain
DM376719	11	9,537,858-9,537,883										Gain
DM376720	11	9,522,766-9,522,791										Gain
DM376721	11	9,499,970-9,499,995										Gain
IPO7	11	9,406,168-9,469,674										Gain
LOC644656	11	9,481,102-9,482,245										Gain
MTCH2	11	47,638,857-47,664,206										Gain
NUP160	11	47,799,634-47,870,096										Gain
PTPRJ	11	48,002,109-48,192,394										Gain
SNORA23	11	9,450,312-9,450,501										Gain
TMEM41B	11	9,302,200-9,336,315										Gain
WEE1	11	9,595,227-9,611,313										Gain
Y_RNA	11	47,841,754-47,841,844										Gain

ZNF143	11	9,482,511-9,550,071										Gain
AK125040	11	134,605,838-134,633,704										Loss
AK130852	11	134,855,245-134,856,693										Loss
BC019904	11	2,349,978-2,399,222										Loss
BC028022	11	58,901,711-58,910,281										Loss
CD81	11	2,397,406-2,418,649										Loss
CD81-AS1	11	2,349,978-2,399,222										Loss
CSNK2A3	11	11,373,488-11,374,904										Loss
DJ031150	11	126,522,696-126,551,995										Loss
FAM111A	11	58,910,218-58,922,511										Loss
FAM111B	11	58,874,657-58,894,888										Loss
FLJ41423	11	45,392,947-45,410,059										Loss
GALNT18	11	11,292,420-11,643,561										Loss
KCNQ1	11	2,466,220-2,870,340										Loss
KCNQ1OT1	11	2,629,557-2,721,228										Loss
KIRREL3	11	126,293,395-126,873,355										Loss
LOC100507548	11	134,855,245-134,856,693										Loss
LOC101928812	11	45,376,921-45,378,208										Loss
LOC101929427	11	126,522,672-126,551,999										Loss
LOC399886	11	45,392,947-45,410,059										Loss
LOC729305	11	134,605,838-134,633,705										Loss
MIR4486	11	19,596,856-19,596,919										Loss
MIR5579	11	79,133,212-79,133,270										Loss
NAV2	11	19,372,270-20,143,147										Loss
NAV2-AS4	11	19,532,439-19,545,625										Loss
NTM	11	131,240,370-132,206,716										Loss
OPCML	11	132,284,558-133,402,507										Loss
PRDM11	11	45,115,563-45,256,675										Loss
SNORA1	11	19,612,702-19,612,838										Loss
SYT13	11	45,261,852-45,307,884										Loss
TENM4	11	78,600,829-78,781,054										Loss
TENM4	11	78,364,327-79,151,695										Loss
TRPM5	11	2,425,745-2,444,275										Loss
TSPAN32	11	2,323,242-2,339,430										Loss

TSSC4	11	2,421,717-2,425,108										Loss
ANHX	12	133,794,897-133,812,422						Gain				
ZNF268	12	133,707,212-133,783,697						Gain				
ALDH2	12	112,204,690-112,247,789										Gain
AMN1	12	31,824,070-31,882,108										Gain
ATF1	12	51,157,788-51,214,943										Gain
ATP2A2	12	110,719,031-110,788,897										Gain
ATXN2	12	111,890,017-112,037,480										Gain
BC059370	12	56,879,224-56,882,153										Gain
BC064974	12	110,939,418-110,941,246										Gain
BICD1	12	32,259,740-32,536,567										Gain
CLEC4C	12	7,882,010-7,902,069										Gain
CSRNP2	12	51,454,987-51,477,454										Gain
DENND5B	12	31,535,156-31,744,070										Gain
DENND5B-AS1	12	31,742,856-31,768,285										Gain
ETFBKMT	12	31,800,093-31,822,016										Gain
FAM186A	12	50,720,012-50,790,405										Gain
FGD4	12	32,638,905-32,798,984										Gain
GLS2	12	56,864,727-56,882,198										Gain
H3F3C	12	31,944,118-31,945,175										Gain
HCAR1	12	123,104,823-123,215,390										Gain
HPD	12	122,277,432-122,326,517										Gain
IFT81	12	110,562,139-110,656,600										Gain
JA611269	12	110,719,702-110,719,761										Gain
KIAA1551	12	32,112,352-32,146,043										Gain
KNTC1	12	123,011,792-123,110,947										Gain
LARP4	12	50,794,591-50,873,788										Gain
LINC02422	12	32,029,902-32,040,137										Gain
LOC494150	12	51,517,733-51,519,265										Gain
LRRC43	12	122,652,265-122,688,018										Gain
MAPKAPK5	12	112,280,031-112,331,228										Gain
MAPKAPK5-AS1	12	112,277,570-112,280,809										Gain
METTL20	12	31,800,093-31,822,016										Gain

METTL7A	12	51,318,533-51,326,300										Gain
MIR331	12	95,702,195-95,702,289										Gain
MIR3685	12	95,703,698-95,703,760										Gain
MLXIP	12	122,516,633-122,631,894										Gain
NANOG	12	7,941,991-7,948,657										Gain
NANOGNB	12	7,917,811-7,926,717										Gain
NR2C1	12	95,414,004-95,467,404										Gain
PCBP2	12	53,835,432-53,874,946										Gain
PCBP2-OT1	12	53,858,251-53,858,841										Gain
PEBP1	12	118,573,688-118,583,393										Gain
POU6F1	12	51,580,718-51,611,539										Gain
PPP1CC	12	111,157,612-111,180,783										Gain
PPTC7	12	110,972,236-111,021,064										Gain
PSMD9	12	122,326,636-122,355,771										Gain
RAD9B	12	110,940,004-110,969,891										Gain
RBMS2	12	56,915,608-56,989,980										Gain
RESF1	12	32,112,352-32,146,043										Gain
RNU6-78P	12	32,146,772-32,146,802										Gain
RSRC2	12	122,989,189-123,011,560										Gain
SNORA105C	12	56,906,648-56,906,764										Gain
SNORA9	12	122,976,659-122,976,788										Gain
SNORD133	12	50,850,353-50,850,569										Gain
SUDS3	12	118,814,357-118,855,840										Gain
TAOK3	12	118,587,605-118,810,750										Gain
TFCP2	12	51,487,538-51,566,926										Gain
TMPRSS12	12	51,236,700-51,281,663										Gain
U5	12	31,747,026-31,747,140										Gain
VEZT	12	95,611,521-95,696,566										Gain
VSIG10	12	118,501,397-118,541,810										Gain
WDR66	12	122,356,462-122,441,832										Gain
ZCCHC8	12	122,956,145-122,985,620										Gain
BC042649	12	131,780,655-131,782,517										Loss
CACNA1C	12	2,162,415-2,807,115										Loss
CACNA1C-AS4	12	2,329,702-2,332,647										Loss

CACNA1C-IT3	12	2,378,941-2,397,911										Loss
KRT1	12	53,068,519-53,074,191										Loss
KRT2	12	53,038,341-53,045,959										Loss
KRT5	12	52,908,358-52,914,243										Loss
KRT6A	12	52,880,959-52,887,041										Loss
KRT6B	12	52,840,434-52,845,910										Loss
KRT6C	12	52,862,299-52,867,569										Loss
KRT71	12	52,937,692-52,946,931										Loss
KRT72	12	52,979,372-52,995,322										Loss
KRT73	12	53,001,353-53,012,343										Loss
KRT73-AS1	12	53,003,634-53,009,089										Loss
KRT74	12	52,959,602-52,967,609										Loss
KRT75	12	52,817,853-52,828,110										Loss
KRT77	12	53,083,410-53,097,247										Loss
KRT82	12	52,787,734-52,800,176										Loss
KRT83	12	52,708,084-52,715,182										Loss
KRT84	12	52,771,595-52,779,417										Loss
KRT85	12	52,753,789-52,761,309										Loss
LINC01257	12	131,649,555-131,697,476										Loss
LINC02370	12	131,832,014-131,852,100										Loss
LINC02415	12	131,780,657-131,782,517										Loss
LOC116437	12	131,649,555-131,697,476										Loss
LOC338797	12	131,832,014-131,852,100										Loss
PIWIL1	12	130,822,432-130,856,877										Loss
ADV21S1A1N	14	22,971,217-22,971,276	Gain	Gain	Gain	Gain	Gain	Gain	Gain	Gain	Gain	Gain
AK093552	14	22,849,082-22,951,948	Gain	Gain	Gain	Gain	Gain	Gain	Gain	Gain	Gain	Gain
AK125397	14	22,886,527-22,901,726	Gain	Gain	Gain	Gain	Gain	Gain	Gain	Gain	Gain	Gain
AV25S1	14	22,689,762-22,690,367	Gain	Gain	Gain	Gain	Gain	Gain	Gain	Gain	Gain	Gain
av27s1	14	22,771,875-22,772,438	Gain	Gain	Gain	Gain	Gain	Gain	Gain	Gain	Gain	Gain
AV2S1A1	14	22,356,036-22,745,911	Gain	Gain	Gain	Gain	Gain	Gain	Gain	Gain	Gain	Gain
AV30S1	14	22,509,128-22,509,405	Gain	Gain	Gain	Gain	Gain	Gain	Gain	Gain	Gain	Gain
AV4S1	14	22,670,486-22,981,890	Gain	Gain	Gain	Gain	Gain	Gain	Gain	Gain	Gain	Gain
hADV29S1	14	22,631,190-23,000,088	Gain	Gain	Gain	Gain	Gain	Gain	Gain	Gain	Gain	Gain
hADV36S1	14	22,694,611-23,016,719	Gain	Gain	Gain	Gain	Gain	Gain	Gain	Gain	Gain	Gain

hADV38S2	14	22,748,987-22,962,976	Gain	Gain	Gain	Gain	Gain	Gain	Gain	Gain	Gain	Gain
hDV102S1	14	22,891,471-22,892,033	Gain	Gain	Gain	Gain	Gain	Gain	Gain	Gain	Gain	Gain
hDV103S1	14	22,938,034-22,938,658	Gain	Gain	Gain	Gain	Gain	Gain	Gain	Gain	Gain	Gain
HTJA150	14	22,968,668-22,968,732	Gain	Gain	Gain	Gain	Gain	Gain	Gain	Gain	Gain	Gain
HTJA84	14	22,970,535-22,970,647	Gain	Gain	Gain	Gain	Gain	Gain	Gain	Gain	Gain	Gain
LOC105370401	14	22,847,807-22,951,948	Gain	Gain	Gain	Gain	Gain	Gain	Gain	Gain	Gain	Gain
TCR	14	22,739,820-22,740,442	Gain	Gain	Gain	Gain	Gain	Gain	Gain	Gain	Gain	Gain
TCRA	14	22,314,718-22,534,581	Gain	Gain	Gain	Gain	Gain	Gain	Gain	Gain	Gain	Gain
TCRA	14	22,538,905-22,539,467	Gain	Gain	Gain	Gain	Gain	Gain	Gain	Gain	Gain	Gain
TCRA	14	22,554,668-22,555,238	Gain	Gain	Gain	Gain	Gain	Gain	Gain	Gain	Gain	Gain
TCRA	14	22,600,965-22,600,989	Gain	Gain	Gain	Gain	Gain	Gain	Gain	Gain	Gain	Gain
TCRA	14	22,636,292-22,636,879	Gain	Gain	Gain	Gain	Gain	Gain	Gain	Gain	Gain	Gain
TCRA	14	22,293,655-23,021,097	Gain	Gain	Gain	Gain	Gain	Gain	Gain	Gain	Gain	Gain
TCRAVN1	14	22,580,299-22,580,985	Gain	Gain	Gain	Gain	Gain	Gain	Gain	Gain	Gain	Gain
TCRDV2	14	22,928,088-22,934,912	Gain	Gain	Gain	Gain	Gain	Gain	Gain	Gain	Gain	Gain
TCRDV2J2	14	22,918,106-22,925,700	Gain	Gain	Gain	Gain	Gain	Gain	Gain	Gain	Gain	Gain
TCRVA13	14	22,539,214-22,539,470	Gain	Gain	Gain	Gain	Gain	Gain	Gain	Gain	Gain	Gain
TRA	14	22,458,652-23,016,719	Gain	Gain	Gain	Gain	Gain	Gain	Gain	Gain	Gain	Gain
TRA@	14	22,362,569-22,534,581	Gain	Gain	Gain	Gain	Gain	Gain	Gain	Gain	Gain	Gain
TRA@	14	22,591,337-22,592,252	Gain	Gain	Gain	Gain	Gain	Gain	Gain	Gain	Gain	Gain
TRA@	14	22,615,959-23,021,097	Gain	Gain	Gain	Gain	Gain	Gain	Gain	Gain	Gain	Gain
TRAC	14	22,466,122-23,021,097	Gain	Gain	Gain	Gain	Gain	Gain	Gain	Gain	Gain	Gain
TRAV20	14	22,508,842-22,509,396	Gain	Gain	Gain	Gain	Gain	Gain	Gain	Gain	Gain	Gain
TRD	14	22,465,824-23,016,719	Gain	Gain	Gain	Gain	Gain	Gain	Gain	Gain	Gain	Gain
TRDC	14	22,919,080-22,934,912	Gain	Gain	Gain	Gain	Gain	Gain	Gain	Gain	Gain	Gain
U39094	14	22,636,757-22,636,881	Gain	Gain	Gain	Gain	Gain	Gain	Gain	Gain	Gain	Gain
X61074	14	22,946,618-22,948,187	Gain	Gain	Gain	Gain	Gain	Gain	Gain	Gain	Gain	Gain
TRA	14	22,192,007-22,447,356	Gain	Gain	Gain	Gain	Gain	Gain	Gain	Gain	Gain	
AK128559	14	35,568,881-35,579,932										Gain
AP4S1	14	31,494,311-31,565,656										Gain
BAZ1A	14	35,221,936-35,344,853										Gain
BBOF1	14	74,486,058-74,532,796										Gain
BC016143	14	73,650,022-73,650,578										Gain
BC038204	14	74,289,261-74,296,838										Gain

CCDC176	14	74,486,058-74,549,566										Gain
CFL2	14	35,179,587-35,184,029										Gain
DNAAF2	14	50,091,891-50,101,948										Gain
EAPP	14	34,985,134-35,008,943										Gain
ENTPD5	14	74,424,618-74,486,035										Gain
FAM177A1	14	35,514,112-35,552,589										Gain
HEATR5A	14	31,760,993-31,926,680										Gain
HECTD1	14	31,569,320-31,676,915										Gain
IGBP1P1	14	35,409,127-35,409,702										Gain
JA429503	14	64,086,261-64,086,281										Gain
KIAA0391	14	35,591,526-35,786,682										Gain
KLHDC1	14	50,159,822-50,219,870										Gain
KLHDC2	14	50,234,786-50,249,856										Gain
LINC02274	14	74,289,261-74,296,838										Gain
LOC100506476	14	74,254,062-74,270,331										Gain
LOC101927178	14	35,550,286-35,582,336										Gain
NEMF	14	50,033,026-50,319,921										Gain
POLE2	14	50,110,269-50,155,098										Gain
PPP2R3C	14	35,554,673-35,591,749										Gain
PSEN1	14	73,603,142-73,690,399										Gain
SGPP1	14	64,150,934-64,194,813										Gain
SNX6	14	35,030,298-35,099,366										Gain
SPTSSA	14	34,902,143-34,931,468										Gain
SRP54	14	35,452,103-35,498,773										Gain
SRP54-AS1	14	35,385,685-35,451,772										Gain
SYNE2	14	64,319,682-64,693,167										Gain
WDR89	14	64,063,756-64,108,641										Gain
BC148240	14	101,540,363-101,540,920										Loss
LINC02285	14	101,587,192-101,589,882										Loss
MEG9	14	101,536,247-101,539,273										Loss
MIR1185-1	14	101,509,313-101,509,398										Loss
MIR1185-2	14	101,510,534-101,510,619										Loss
MIR134	14	101,521,023-101,521,096										Loss
MIR154	14	101,526,091-101,526,175										Loss

MIR300	14	101,507,699-101,507,781										Loss
MIR323B	14	101,522,555-101,522,637										Loss
MIR369	14	101,531,934-101,532,004										Loss
MIR377	14	101,528,386-101,528,455										Loss
MIR381	14	101,512,256-101,512,331										Loss
MIR381HG	14	101,511,493-101,518,132										Loss
MIR382	14	101,520,642-101,520,718										Loss
MIR409	14	101,531,636-101,531,715										Loss
MIR410	14	101,532,248-101,532,328										Loss
MIR412	14	101,531,783-101,531,874										Loss
MIR485	14	101,521,755-101,521,828										Loss
MIR487A	14	101,518,782-101,518,862										Loss
MIR487B	14	101,512,791-101,512,875										Loss
MIR496	14	101,526,909-101,527,011										Loss
MIR539	14	101,513,657-101,513,735										Loss
MIR541	14	101,530,831-101,530,915										Loss
MIR544A	14	101,514,994-101,515,085										Loss
MIR655	14	101,515,886-101,515,983										Loss
MIR656	14	101,533,060-101,533,138										Loss
MIR668	14	101,521,594-101,521,660										Loss
MIR889	14	101,514,237-101,514,316										Loss
ECRP	14	21,387,499-21,388,335							AI	AI	AI	
RNASE2	14	21,387,499-21,424,594							AI	AI	AI	
RNASE3	14	21,359,561-21,360,507							AI	AI	AI	
TCRA	14	22,392,580-22,392,828	Gain	Gain	Gain	Gain	Gain		Gain	Gain	Gain	
AK091906	15	51,132,072-51,144,737										Gain
AX746640	15	50,784,502-50,787,028										Gain
BC067230	15	41,594,572-41,598,726										Gain
BC107108	15	20,362,687-20,364,420										Gain
CASC4	15	44,580,908-44,707,959										Gain
CHEK2P2	15	20,487,996-20,496,811										Gain
CHP1	15	41,523,436-41,574,083										Gain
CTDSPL2	15	44,719,578-44,819,429										Gain
DQ571479	15	20,327,817-20,327,845										Gain

DQ576041	15	20,319,594-20,319,625										Gain
DQ582073	15	20,739,311-20,739,342										Gain
DQ582939	15	20,771,429-20,771,455										Gain
DQ592463	15	20,446,055-20,446,082										Gain
DQ594309	15	20,742,234-20,742,263										Gain
DQ595648	15	20,744,538-20,744,567										Gain
DQ600342	15	20,770,228-20,770,259										Gain
EXD1	15	41,474,930-41,522,955										Gain
FRMD5	15	44,162,958-44,487,492										Gain
GOLGA6L6	15	20,737,093-20,747,114										Gain
GOLGA8CP	15	20,767,673-20,781,026										Gain
HERC2P3	15	20,588,367-20,711,433										Gain
HERC2P7	15	20,723,928-20,727,150										Gain
INO80	15	41,267,987-41,408,478										Gain
NDUFAF1	15	41,679,546-41,694,658										Gain
NUSAP1	15	41,624,891-41,673,248										Gain
OIP5	15	41,601,465-41,624,819										Gain
OIP5-AS1	15	41,576,187-41,605,536										Gain
RTF1	15	41,709,301-41,775,761										Gain
SPPL2A	15	50,999,736-51,057,910										Gain
TRIP4	15	64,680,002-64,747,502										Gain
TRPM7	15	50,896,143-50,916,497										Gain
TRPM7	15	50,849,351-50,979,012										Gain
USP50	15	50,792,758-50,838,902										Gain
USP8	15	50,716,573-50,793,280										Gain
ZNF609	15	64,752,940-64,978,266										Gain
LINC00593	15	70,127,572-70,135,306										Loss
MIR629	15	70,371,710-70,371,807										Loss
TLE3	15	70,340,129-70,390,256										Loss
BCAR4	16	11,913,690-11,922,689										Gain
CYB5B	16	69,458,497-69,500,167										Gain
GLG1	16	74,481,325-74,641,042										Gain
GSPT1	16	11,961,984-12,010,519										Gain
HAS3	16	69,139,466-69,152,619										Gain

MIR1538	16	69,599,710-69,599,771										Gain
NFAT5	16	69,599,868-69,738,569										Gain
RFWD3	16	74,655,296-74,700,779										Gain
RSL1D1	16	11,928,054-11,945,442										Gain
SNORD13H	16	69,742,474-69,742,578										Gain
SNTB2	16	69,221,049-69,342,955										Gain
TANGO6	16	68,877,508-69,119,085										Gain
VPS4A	16	69,345,286-69,358,946										Gain
ZC3H7A	16	11,844,441-11,891,114										Gain
ABR	17	906,757-1,132,974										Gain
AK093551	17	41,300,387-41,316,185										Gain
AK311131	17	41,316,199-41,322,420										Gain
ATAD5	17	29,158,987-29,222,883										Gain
BC017255	17	57,183,958-57,195,629										Gain
BCAS3	17	58,755,135-59,470,199										Gain
BPTF	17	65,821,779-65,980,494										Gain
BRCA1	17	41,196,311-41,322,420										Gain
CLTC	17	57,697,049-57,774,317										Gain
CRLF3	17	29,109,701-29,151,778										Gain
DD413682	17	2,489,695-2,489,717										Gain
DHX40	17	57,642,885-57,685,713										Gain
DM119512	17	57,918,623-57,918,701										Gain
EFCAB3	17	60,447,578-60,493,839										Gain
FLJ36000	17	21,904,061-21,913,070										Gain
GDPD1	17	57,297,827-57,353,330										Gain
JB137816	17	22,021,439-22,021,460										Gain
LINC01476	17	57,508,229-57,604,218										Gain
LOC101929767	17	41,300,383-41,322,420										Gain
MED13	17	60,019,965-60,142,643										Gain
MILR1	17	62,461,568-62,464,760										Gain
MIR21	17	57,918,626-57,918,698										Gain
MIR301A	17	57,228,496-57,228,582										Gain
MIR4523	17	27,717,679-27,717,748										Gain
MIR454	17	57,215,118-57,215,233										Gain

MIR4729	17	57,443,443-57,443,515										Gain
MIR548AA2	17	65,467,604-65,467,701										Gain
MIR548D2	17	65,467,606-65,467,701										Gain
MTRNR2L1	17	22,022,436-22,023,991										Gain
NBR1	17	41,322,487-41,363,708										Gain
NBR2	17	41,277,573-41,305,688										Gain
NOL11	17	65,713,948-65,740,324										Gain
PAFAH1B1	17	2,496,922-2,588,909										Gain
PECAM1	17	62,396,776-62,407,083										Gain
PITPNC1	17	65,373,396-65,693,379										Gain
POLG2	17	62,473,901-62,493,184										Gain
PPM1E	17	56,833,229-57,062,540										Gain
PRR11	17	57,232,859-57,284,070										Gain
PSMD12	17	65,334,031-65,362,743										Gain
PTRH2	17	57,774,666-57,784,959										Gain
RAD51C	17	56,769,933-56,811,703										Gain
RHOT1	17	30,469,472-30,552,790										Gain
SCARF1	17	1,537,151-1,549,083										Gain
SKA2	17	57,187,219-57,232,800										Gain
SLC43A2	17	1,472,547-1,532,180										Gain
SMURF2	17	62,540,734-62,658,386										Gain
SNORA38B	17	65,736,785-65,736,917										Gain
SUZ12P1	17	29,036,625-29,119,848										Gain
TAOK1	17	27,717,942-27,878,921										Gain
TBC1D3P2	17	60,342,066-60,353,016										Gain
TEFM	17	29,226,000-29,233,286										Gain
TRIM37	17	57,059,999-57,184,325										Gain
TRNA	17	22,021,364-22,021,435										Gain
TRNA	17	22,025,083-22,025,155										Gain
TUBD1	17	57,936,840-57,970,306										Gain
VMP1	17	57,784,825-57,919,616										Gain
WDR81	17	1,619,816-1,641,893										Gain
YPEL2	17	57,409,052-57,479,095										Gain
YWHAE	17	1,247,833-1,303,556										Gain

ABHD3	18	19,230,857-19,284,766										Gain
ESCO1	18	19,109,261-19,180,693										Gain
GREB1L	18	18,822,202-19,106,073										Gain
MIB1	18	19,284,917-19,450,918										Gain
MIR1-2	18	19,408,964-19,409,049										Gain
MIR133A1	18	19,405,658-19,405,746										Gain
MIR133A1HG	18	19,405,439-19,411,367										Gain
mir-133b	18	19,405,447-19,411,367										Gain
MIR320C1	18	19,263,470-19,263,558										Gain
ROCK1	18	18,529,702-18,691,812										Gain
SNRPD1	18	19,192,229-19,210,206										Gain
CELF4	18	34,823,007-35,146,000										Loss
MIR4318	18	35,237,097-35,237,178										Loss
SMIM21	18	73,121,430-73,139,658										Loss
SNORA111	18	35,046,301-35,046,467										Loss
ZNF569	19	37,902,043-37,960,169	Gain					Gain				Gain
ZNF283	19	44,331,443-44,353,050						Gain				
ZNF546	19	40,502,942-40,526,948						Gain				
ZNF780B	19	40,534,166-40,562,115						Gain				
ZNF85	19	21,106,058-21,133,503						Gain				
ADAMTS10	19	8,645,123-8,675,620										Gain
ADGRE4P	19	6,952,510-6,990,857										Gain
AK023304	19	12,305,829-12,349,077										Gain
ARHGEF18	19	7,447,719-7,537,371										Gain
AX721123	19	12,305,829-12,318,387										Gain
AX746638	19	36,803,968-36,806,639										Gain
AX747088	19	48,285,436-48,287,939										Gain
AX747375	19	37,264,056-37,266,689										Gain
BC018887	19	10,426,884-10,427,510										Gain
BC024306	19	37,266,689-37,267,980										Gain
BC039524	19	37,063,971-37,085,610										Gain
BCAT2	19	49,298,318-49,314,320										Gain
BSPH1	19	48,471,302-48,495,427										Gain
C19orf10	19	4,657,556-4,670,415										Gain

CA11	19	49,141,198-49,149,569									Gain
CAPN12	19	39,220,831-39,235,114									Gain
CCDC130	19	13,842,573-13,874,112									Gain
CD320	19	8,367,010-8,373,240									Gain
CERS4	19	8,274,216-8,327,304									Gain
COX7A1	19	36,641,823-36,643,771									Gain
CRX	19	48,325,096-48,346,586									Gain
DBP	19	49,133,816-49,140,807									Gain
DPP9	19	4,675,235-4,723,855									Gain
DPP9-AS1	19	4,679,293-4,685,960									Gain
ECSIT	19	11,616,730-11,639,987									Gain
ELSPBP1	19	48,497,907-48,528,410									Gain
EMR4P	19	6,952,510-6,990,857									Gain
FAM83E	19	49,103,856-49,116,694									Gain
FBN3	19	8,130,285-8,214,730									Gain
FDX1L	19	10,416,065-10,426,691									Gain
FDX2	19	10,420,886-10,426,691									Gain
FGF21	19	49,258,815-49,261,587									Gain
FLJ25758	19	7,004,963-7,006,426									Gain
FUT1	19	49,251,267-49,258,647									Gain
HDGFL2	19	4,472,192-4,502,232									Gain
HDGFRP2	19	4,472,254-4,502,222									Gain
INSR	19	7,112,265-7,294,313									Gain
KANK2	19	11,274,942-11,308,467									Gain
KEAP1	19	10,596,795-10,614,054									Gain
LGALS7	19	39,261,607-39,264,157									Gain
LGALS7B	19	39,279,849-39,282,394									Gain
LINC00665	19	36,803,962-36,822,620									Gain
LINC01534	19	37,176,340-37,178,351									Gain
LOC100131094	19	4,679,293-4,685,960									Gain
LOC100134317	19	36,802,244-36,803,570									Gain
LOC100289333	19	12,305,829-12,318,391									Gain
LOC100507551	19	17,599,798-17,622,698									Gain
LOC644189	19	36,912,075-36,913,799									Gain

LOC728485	19	37,264,019-37,267,980										Gain
LOC728752	19	36,980,528-36,981,942										Gain
MBD3L1	19	8,953,268-8,954,016										Gain
MBD3L2	19	7,049,350-7,051,746										Gain
MBD3L2B	19	7,049,350-7,051,623										Gain
MBD3L3	19	7,056,215-7,058,645										Gain
MBD3L4	19	7,037,754-7,040,184										Gain
MBD3L5	19	7,030,593-7,033,022										Gain
MED16	19	873,431-873,781										Gain
MED16	19	867,961-893,218										Gain
MIR1283-2	19	54,261,485-54,261,572										Gain
MIR371A	19	54,290,928-54,290,995										Gain
MIR371B	19	54,290,930-54,290,996										Gain
MIR372	19	54,291,143-54,291,210										Gain
MIR373	19	54,291,958-54,292,027										Gain
MIR516A1	19	54,259,994-54,260,084										Gain
MIR516A2	19	54,264,386-54,264,476										Gain
MIR516B1	19	54,240,098-54,240,188										Gain
MIR516B2	19	54,228,695-54,228,780										Gain
MIR517A	19	54,215,521-54,215,608										Gain
MIR517B	19	54,224,329-54,224,396										Gain
MIR517C	19	54,244,566-54,244,661										Gain
MIR518A1	19	54,234,259-54,234,344										Gain
MIR518A2	19	54,242,586-54,242,673										Gain
MIR518B	19	54,205,990-54,206,073										Gain
MIR518C	19	54,211,988-54,212,089										Gain
MIR518D	19	54,238,130-54,238,217										Gain
MIR518E	19	54,233,091-54,233,179										Gain
MIR518F	19	54,203,268-54,203,355										Gain
MIR519A1	19	54,255,650-54,255,735										Gain
MIR519A2	19	54,265,597-54,265,684										Gain
MIR519B	19	54,198,466-54,198,547										Gain
MIR519D	19	54,216,600-54,216,688										Gain
MIR520A	19	54,194,134-54,194,219										Gain

MIR520B	19	54,204,480-54,204,541										Gain
MIR520C	19	54,210,706-54,210,793										Gain
MIR520D	19	54,223,349-54,223,436										Gain
MIR520G	19	54,225,419-54,225,509										Gain
MIR520H	19	54,245,765-54,245,853										Gain
MIR521-1	19	54,251,889-54,251,976										Gain
MIR521-2	19	54,219,847-54,219,934										Gain
MIR522	19	54,254,464-54,254,551										Gain
MIR523	19	54,201,638-54,201,725										Gain
MIR524	19	54,214,255-54,214,342										Gain
MIR525	19	54,200,786-54,200,871										Gain
MIR526A1	19	54,209,505-54,209,590										Gain
MIR526A2	19	54,230,175-54,230,240										Gain
MIR526B	19	54,197,646-54,197,729										Gain
MIR527	19	54,257,271-54,257,356										Gain
MIR7974	19	11,606,358-11,606,437										Gain
MISP	19	751,112-764,319										Gain
MRI1	19	13,875,327-13,885,096										Gain
MYDGF	19	4,657,556-4,670,415										Gain
MYO1F	19	8,585,673-8,642,331										Gain
NDUFA7	19	8,373,486-8,386,297										Gain
NTN5	19	49,164,663-49,176,338										Gain
OPA3	19	46,031,024-46,105,466										Gain
PALM	19	708,952-748,330										Gain
PGLS	19	17,622,431-17,632,097										Gain
PKN1	19	14,544,117-14,582,684										Gain
PLIN4	19	4,502,191-4,517,716										Gain
RAVER1	19	10,426,888-10,444,205										Gain
RETN	19	7,733,971-7,735,340										Gain
RPL18	19	49,118,583-49,122,675										Gain
S1PR5	19	10,623,417-10,628,668										Gain
SEC1P	19	49,141,295-49,185,502										Gain
SELENOW	19	48,281,828-48,287,938										Gain
SEPW1	19	48,281,841-48,287,943										Gain

SLC27A1	19	17,581,252-17,616,977										Gain
SNAR-A1	19	48,421,685-48,421,807										Gain
SNAR-A12	19	48,410,934-48,411,056										Gain
SNAR-A13	19	48,448,178-48,448,300										Gain
SNAR-A14	19	48,427,035-48,427,156										Gain
SNAR-A2	19	48,421,685-48,421,807										Gain
SNAR-A3	19	48,427,035-48,427,156										Gain
SNAR-C1	19	48,432,074-48,432,194										Gain
SNAR-C2	19	48,416,307-48,416,427										Gain
SNAR-C2	19	48,432,074-48,432,194										Gain
SNAR-C3	19	48,453,552-48,453,671										Gain
SNAR-C4	19	48,442,797-48,442,917										Gain
SNAR-C5	19	48,458,938-48,459,058										Gain
SPHK2	19	49,122,547-49,133,974										Gain
STXBP2	19	7,701,986-7,712,760										Gain
SULT2A1	19	48,373,722-48,389,654										Gain
TNFAIP8L1	19	4,639,526-4,655,580										Gain
TPRX1	19	48,304,499-48,306,861										Gain
USHBP1	19	17,360,829-17,375,605										Gain
VASP	19	46,010,687-46,030,240										Gain
ZFP14	19	36,825,354-36,870,105										Gain
ZFP82	19	36,874,003-36,909,576										Gain
ZNF136	19	12,273,861-12,300,696										Gain
ZNF146	19	36,705,503-36,729,675										Gain
ZNF260	19	37,001,588-37,019,248										Gain
ZNF345	19	37,341,259-37,403,930										Gain
ZNF382	19	37,095,718-37,119,499										Gain
ZNF383	19	37,708,827-37,734,826										Gain
ZNF420	19	37,569,123-37,621,268										Gain
ZNF430	19	21,203,425-21,242,852										Gain
ZNF44	19	12,335,500-12,405,714										Gain
ZNF441	19	11,877,814-11,894,893										Gain
ZNF443	19	12,540,519-12,551,926										Gain
ZNF461	19	37,127,522-37,157,755										Gain

ZNF490	19	12,686,919-12,721,623										Gain
ZNF529	19	37,034,516-37,096,178										Gain
ZNF529-AS1	19	37,063,971-37,085,618										Gain
ZNF557	19	7,069,470-7,087,978										Gain
ZNF558	19	8,920,251-8,942,980										Gain
ZNF563	19	12,428,303-12,444,534										Gain
ZNF564	19	12,636,183-12,662,356										Gain
ZNF565	19	36,672,961-36,736,830										Gain
ZNF566	19	36,936,020-36,980,804										Gain
ZNF567	19	37,178,513-37,214,248										Gain
ZNF568	19	37,407,230-37,488,834										Gain
ZNF585A	19	37,638,339-37,663,643										Gain
ZNF585B	19	37,672,480-37,701,451										Gain
ZNF627	19	11,708,234-11,729,974										Gain
ZNF653	19	11,594,241-11,616,738										Gain
ZNF709	19	12,571,997-12,662,356										Gain
ZNF714	19	21,264,952-21,307,883										Gain
ZNF790	19	37,309,223-37,341,689										Gain
ZNF790-AS1	19	37,288,451-37,319,013										Gain
ZNF823	19	11,832,079-11,849,824										Gain
ZNF829	19	37,379,025-37,407,193										Gain
ZNF833P	19	11,750,590-11,797,384										Gain
ZNF850	19	37,234,379-37,263,716										Gain
ZNF432	19	52,534,631-52,552,377			Gain							
ZNF429	19	21,688,353-21,739,070	Gain									
ZNF808	19	53,030,908-53,067,717	Gain									
ZNF846	19	9,862,668-9,896,859	Gain									
LINC01429	20	50,448,335-50,479,452										Gain
LOC284801	20	26,167,654-26,189,869										Gain
MIR663A	20	26,188,821-26,188,914										Gain
MIR663AHG	20	26,167,654-26,189,869										Gain
NCOR1P1	20	26,084,051-26,094,677										Gain
PHF20	20	34,359,922-34,538,288										Gain
RBL1	20	35,624,754-35,724,618										Gain

RBM39	20	34,291,530-34,330,258										Gain
ROMO1	20	34,287,231-34,288,902										Gain
SOGA1	20	35,405,844-35,492,087										Gain
TLDC2	20	35,504,523-35,522,634										Gain
SYCP2	20	58,438,611-58,508,718					Gain					Loss
AK056098	20	56,285,238-56,287,808										Loss
APMAP	20	24,943,579-24,973,425										Loss
AX747264	20	23,026,627-23,028,780										Loss
BC045663	20	22,989,741-23,011,422										Loss
C20orf197	20	58,630,979-58,648,008										Loss
CD93	20	23,059,992-23,066,977										Loss
CDH26	20	58,533,450-58,608,829										Loss
CDH4	20	59,827,481-60,515,673										Loss
CST11	20	23,431,040-23,433,482										Loss
CST13P	20	23,499,782-23,522,655										Loss
CST7	20	24,929,865-24,940,564										Loss
CST8	20	23,471,737-23,476,655										Loss
CST9L	20	23,545,369-23,549,386										Loss
CSTL1	20	23,420,321-23,425,567										Loss
FAM217B	20	58,508,818-58,523,702										Loss
LINC01742	20	56,578,959-56,579,704										Loss
MIR4532	20	56,470,449-56,470,500										Loss
NAPB	20	23,355,155-23,402,156										Loss
NKILA	20	56,285,238-56,287,836										Loss
PCK1	20	56,136,136-56,141,513										Loss
PHACTR3	20	58,152,563-58,422,766										Loss
PMEPA1	20	56,223,447-56,286,592										Loss
PPP1R3D	20	58,511,886-58,515,352										Loss
SIRPB2	20	1,455,235-1,472,233										Loss
SLC24A3	20	19,193,289-19,703,541										Loss
SYNDIG1	20	24,449,834-24,647,253										Loss
THBD	20	23,026,269-23,030,301										Loss
ZBP1	20	56,178,901-56,195,632										Loss
KRTAP10-10	21	46,057,272-46,058,372										Loss

KRTAP10-11	21	46,066,330-46,067,566										Loss
KRTAP10-8	21	46,031,995-46,032,871										Loss
KRTAP10-9	21	46,047,039-46,048,295										Loss
KRTAP12-3	21	46,077,848-46,078,258										Loss
KRTAP12-4	21	46,074,129-46,074,576										Loss
TSPEAR	21	45,917,774-46,131,495										Loss
7SK	22	20,455,668-20,455,969	AI, Loss		AI, Loss	AI, Gain	AI, Loss	AI	AI		AI	AI, Loss
DGCR6L	22	20,301,760-20,307,628	AI, Loss		AI, Loss	AI, Gain	AI, Loss	AI	AI		AI	AI, Loss
FAM230A	22	20,336,993-20,350,461	AI, Loss		AI, Loss	AI, Gain	AI, Loss	AI	AI		AI	AI, Loss
FAM230G	22	20,328,519-20,706,784	AI, Loss		AI, Loss	AI, Gain	AI, Loss	AI	AI		AI	AI, Loss
FAM230J	22	20,632,131-20,656,828	AI, Loss		AI, Loss	AI, Gain	AI, Loss	AI	AI		AI	AI, Loss
FLJ41941	22	18,512,150-18,520,734	AI, Loss		AI, Loss	AI, Gain	AI, Loss	AI	AI		AI	AI, Loss
GGTLC3	22	20,366,210-20,368,028	AI, Loss		AI, Loss	AI, Gain	AI, Loss	AI	AI		AI	AI, Loss
HV593096	22	20,390,284-20,390,311	AI, Loss		AI, Loss	AI, Gain	AI, Loss	AI	AI		AI	AI, Loss
HV593127	22	20,397,894-20,397,917	AI, Loss		AI, Loss	AI, Gain	AI, Loss	AI	AI		AI	AI, Loss
HV593134	22	20,397,144-20,397,171	AI, Loss		AI, Loss	AI, Gain	AI, Loss	AI	AI		AI	AI, Loss
HV593135	22	20,398,579-20,398,601	AI, Loss		AI, Loss	AI, Gain	AI, Loss	AI	AI		AI	AI, Loss
HV593178	22	20,398,307-20,398,335	AI, Loss		AI, Loss	AI, Gain	AI, Loss	AI	AI		AI	AI, Loss
JX456220	22	20,695,276-20,710,250	AI, Loss		AI, Loss	AI, Gain	AI, Loss	AI	AI		AI	AI, Loss
KIAA1653	22	20,291,560-20,299,716	AI, Loss		AI, Loss	AI, Gain	AI, Loss	AI	AI		AI	AI, Loss

KLHL22	22	20,795,805-20,850,170	AI, Loss		AI, Loss	AI, Gain	AI, Loss	AI	AI		AI	AI, Loss
LINC01634	22	18,512,150-18,520,734	AI, Loss		AI, Loss	AI, Gain	AI, Loss	AI	AI		AI	AI, Loss
LOC729444	22	20,325,589-20,350,461	AI, Loss		AI, Loss	AI, Gain	AI, Loss	AI	AI		AI	AI, Loss
MED15	22	20,861,828-20,941,919	AI, Loss		AI, Loss	AI, Gain	AI, Loss	AI	AI		AI	AI, Loss
MICAL3	22	18,270,415-18,507,325	AI, Loss		AI, Loss	AI, Gain	AI, Loss	AI	AI		AI	AI, Loss
MIR1286	22	20,236,656-20,236,734	AI, Loss		AI, Loss	AI, Gain	AI, Loss	AI	AI		AI	AI, Loss
MIR648	22	18,463,633-18,463,727	AI, Loss		AI, Loss	AI, Gain	AI, Loss	AI	AI		AI	AI, Loss
PI4KAP1	22	20,383,730-20,427,815	AI, Loss		AI, Loss	AI, Gain	AI, Loss	AI	AI		AI	AI, Loss
RIMBP3	22	20,455,993-20,461,786	AI, Loss		AI, Loss	AI, Gain	AI, Loss	AI	AI		AI	AI, Loss
RTN4R	22	20,228,937-20,255,816	AI, Loss		AI, Loss	AI, Gain	AI, Loss	AI	AI		AI	AI, Loss
SCARF2	22	20,778,873-20,792,146	AI, Loss		AI, Loss	AI, Gain	AI, Loss	AI	AI		AI	AI, Loss
TMEM191B	22	20,377,668-20,380,440	AI, Loss		AI, Loss	AI, Gain	AI, Loss	AI	AI		AI	AI, Loss
USP41	22	20,704,867-20,731,542	AI, Loss		AI, Loss	AI, Gain	AI, Loss	AI	AI		AI	AI, Loss
ZNF74	22	20,748,404-20,762,753	AI, Loss		AI, Loss	AI, Gain	AI, Loss	AI	AI		AI	AI, Loss
CECR7	22	17,517,459-17,540,960	AI, Loss		AI, Loss	AI, Gain	AI, Loss	AI	AI		AI	Loss
GAB4	22	17,442,825-17,489,112	AI, Loss		AI, Loss	AI, Gain	AI, Loss	AI	AI		AI	Loss
IL17RA	22	17,565,848-17,596,584	AI, Loss		AI, Loss	AI, Gain	AI, Loss	AI	AI		AI	Loss

BC021738	22	17,602,484-17,612,994	AI, Loss		AI, Loss	Gain	AI, Loss	AI	AI		AI	
CECR6	22	17,597,188-17,602,257	AI, Loss		AI, Loss	Gain	AI, Loss	AI	AI		AI	
LINC01664	22	17,602,484-17,612,994	AI, Loss		AI, Loss	Gain	AI, Loss	AI	AI		AI	
TMEM121B	22	17,597,188-17,602,257	AI, Loss		AI, Loss	Gain	AI, Loss	AI	AI		AI	
CLTCL1	22	19,166,986-19,279,239	AI, Loss		AI, Loss	AI, Gain	AI, Loss	AI, Gain	AI	AI	AI	AI, Loss
HIRA	22	19,318,223-19,435,755	AI, Loss		AI, Loss	AI, Gain	AI, Loss	AI, Gain	AI	AI	AI	AI, Loss
MRPL40	22	19,419,424-19,423,601	AI, Loss		AI, Loss	AI, Gain	AI, Loss	AI, Gain	AI	AI	AI	AI, Loss
AK055980	22	27,444,105-27,456,467	AI, Loss	AI	AI, Loss	Loss, LOH	AI, Loss	AI, Loss	AI	AI	AI	AI, Loss
AP1B1P1	22	32,517,963-32,529,456	AI, Loss	AI	AI, Loss	Loss, LOH	AI, Loss	AI, Loss	AI	AI	AI	AI, Loss
C22orf42	22	32,544,992-32,555,279	AI, Loss	AI	AI, Loss	Loss, LOH	AI, Loss	AI, Loss	AI	AI	AI	AI, Loss
CR936633	22	27,706,611-27,713,417	AI, Loss	AI	AI, Loss	Loss, LOH	AI, Loss	AI, Loss	AI	AI	AI	AI, Loss
CRYBA4	22	27,017,927-27,026,636	AI, Loss	AI	AI, Loss	Loss, LOH	AI, Loss	AI, Loss	AI	AI	AI	AI, Loss
CRYBB1	22	26,995,241-27,013,991	AI, Loss	AI	AI, Loss	Loss, LOH	AI, Loss	AI, Loss	AI	AI	AI	AI, Loss
HMGXB4	22	35,653,444-35,691,800	AI, Loss	AI	AI, Loss	Loss, LOH	AI, Loss	AI, Loss	AI	AI	AI	AI, Loss
JB175027	22	32,524,336-32,524,357	AI, Loss	AI	AI, Loss	Loss, LOH	AI, Loss	AI, Loss	AI	AI	AI	AI, Loss
LINC00229	22	45,002,207-45,021,299	AI, Loss	AI	AI, Loss	Loss, LOH	AI, Loss	AI, Loss	AI	AI	AI	AI, Loss
LINC01422	22	27,299,254-27,316,573	AI, Loss	AI	AI, Loss	Loss, LOH	AI, Loss	AI, Loss	AI	AI	AI	AI, Loss

LINC02554	22	27,706,611-27,713,801	AI, Loss	AI	AI, Loss	Loss, LOH	AI, Loss	AI, Loss	AI	AI	AI	AI, Loss
LOC110091768	22	27,256,173-27,277,748	AI, Loss	AI	AI, Loss	Loss, LOH	AI, Loss	AI, Loss	AI	AI	AI	AI, Loss
LOC284898	22	27,444,105-27,456,480	AI, Loss	AI	AI, Loss	Loss, LOH	AI, Loss	AI, Loss	AI	AI	AI	AI, Loss
MB	22	36,002,810-36,019,401	AI, Loss	AI	AI, Loss	Loss, LOH	AI, Loss	AI, Loss	AI	AI	AI	AI, Loss
MIR3201	22	48,670,175-48,670,227	AI, Loss	AI	AI, Loss	Loss, LOH	AI, Loss	AI, Loss	AI	AI	AI	AI, Loss
MIR3909	22	35,731,632-35,731,751	AI, Loss	AI	AI, Loss	Loss, LOH	AI, Loss	AI, Loss	AI	AI	AI	AI, Loss
MIR4764	22	33,832,567-33,832,655	AI, Loss	AI	AI, Loss	Loss, LOH	AI, Loss	AI, Loss	AI	AI	AI	AI, Loss
MIR6069	22	35,732,713-35,732,792	AI, Loss	AI	AI, Loss	Loss, LOH	AI, Loss	AI, Loss	AI	AI	AI	AI, Loss
PARVB	22	44,395,090-44,565,112	AI, Loss	AI	AI, Loss	Loss, LOH	AI, Loss	AI, Loss	AI	AI	AI	AI, Loss
RASD2	22	35,936,540-35,950,045	AI, Loss	AI	AI, Loss	Loss, LOH	AI, Loss	AI, Loss	AI	AI	AI	AI, Loss
RFPL2	22	32,586,421-32,600,836	AI, Loss	AI	AI, Loss	Loss, LOH	AI, Loss	AI, Loss	AI	AI	AI	AI, Loss
SUSD2	22	24,577,443-24,585,074	AI, Loss	AI	AI, Loss	Loss, LOH	AI, Loss	AI, Loss	AI	AI	AI	AI, Loss
TOM1	22	35,695,267-35,743,987	AI, Loss	AI	AI, Loss	Loss, LOH	AI, Loss	AI, Loss	AI	AI	AI	AI, Loss
AX747952	22	44,383,785-44,385,884	AI, Loss	AI	AI, Loss	Loss, LOH	Loss	AI, Loss	AI	AI	AI	AI, Loss
PNPLA3	22	44,319,618-44,360,433	AI, Loss	AI	AI, Loss	Loss, LOH	Loss	AI, Loss	AI	AI	AI	AI, Loss
SAMM50	22	44,351,260-44,392,412	AI, Loss	AI	AI, Loss	Loss, LOH	Loss	AI, Loss	AI	AI	AI	AI, Loss
BIK	22	43,506,753-43,525,718	Loss	AI	AI, Loss	Loss, LOH	AI, Loss	AI, Loss	AI	AI	AI	AI, Loss

MCAT	22	43,528,211-43,539,403	Loss	AI	AI, Loss	Loss, LOH	AI, Loss	AI, Loss	AI	AI	AI	AI, Loss
AK124820	22	27,063,655-27,068,617	AI, Loss	AI	Loss	Loss, LOH	AI, Loss	AI, Loss	AI	AI	AI	AI, Loss
BRD1	22	50,166,925-50,221,575	AI, Loss	AI	Loss	Loss, LOH	AI, Loss	AI, Loss	AI	AI	AI	AI, Loss
CABIN1	22	24,407,764-24,574,596	AI, Loss	AI	Loss	Loss, LOH	AI, Loss	AI, Loss	AI	AI	AI	AI, Loss
DDT	22	24,313,553-24,322,019	AI, Loss	AI	Loss	Loss, LOH	AI, Loss	AI, Loss	AI	AI	AI	AI, Loss
DDTL	22	24,309,025-24,314,748	AI, Loss	AI	Loss	Loss, LOH	AI, Loss	AI, Loss	AI	AI	AI	AI, Loss
DQ571361	22	24,239,835-24,239,864	AI, Loss	AI	Loss	Loss, LOH	AI, Loss	AI, Loss	AI	AI	AI	AI, Loss
DQ574115	22	24,238,568-24,238,598	AI, Loss	AI	Loss	Loss, LOH	AI, Loss	AI, Loss	AI	AI	AI	AI, Loss
DQ575315	22	24,240,066-24,240,132	AI, Loss	AI	Loss	Loss, LOH	AI, Loss	AI, Loss	AI	AI	AI	AI, Loss
DQ579704	22	24,239,638-24,239,678	AI, Loss	AI	Loss	Loss, LOH	AI, Loss	AI, Loss	AI	AI	AI	AI, Loss
DQ584254	22	24,239,748-24,239,792	AI, Loss	AI	Loss	Loss, LOH	AI, Loss	AI, Loss	AI	AI	AI	AI, Loss
DQ586951	22	45,724,002-45,724,028	AI, Loss	AI	Loss	Loss, LOH	AI, Loss	AI, Loss	AI	AI	AI	AI, Loss
DQ596074	22	24,253,496-24,253,538	AI, Loss	AI	Loss	Loss, LOH	AI, Loss	AI, Loss	AI	AI	AI	AI, Loss
DQ596562	22	24,255,602-24,255,629	AI, Loss	AI	Loss	Loss, LOH	AI, Loss	AI, Loss	AI	AI	AI	AI, Loss
DQ597394	22	24,240,744-24,240,774	AI, Loss	AI	Loss	Loss, LOH	AI, Loss	AI, Loss	AI	AI	AI	AI, Loss
DQ600483	22	24,245,625-24,247,864	AI, Loss	AI	Loss	Loss, LOH	AI, Loss	AI, Loss	AI	AI	AI	AI, Loss
DQ601926	22	24,235,191-24,235,227	AI, Loss	AI	Loss	Loss, LOH	AI, Loss	AI, Loss	AI	AI	AI	AI, Loss

EFCAB6-AS1	22	43,912,133-43,932,485	AI, Loss	AI	Loss	Loss, LOH	AI, Loss	AI, Loss	AI	AI	AI	AI, Loss
FAM118A	22	45,704,840-45,737,836	AI, Loss	AI	Loss	Loss, LOH	AI, Loss	AI, Loss	AI	AI	AI	AI, Loss
FBLN1	22	45,898,718-45,997,014	AI, Loss	AI	Loss	Loss, LOH	AI, Loss	AI, Loss	AI	AI	AI	AI, Loss
FOXRED2	22	36,883,232-36,903,148	AI, Loss	AI	Loss	Loss, LOH	AI, Loss	AI, Loss	AI	AI	AI	AI, Loss
GSTT1	22	24,376,132-24,384,311	AI, Loss	AI	Loss	Loss, LOH	AI, Loss	AI, Loss	AI	AI	AI	AI, Loss
GSTT1-AS1	22	24,375,962-24,376,214	AI, Loss	AI	Loss	Loss, LOH	AI, Loss	AI, Loss	AI	AI	AI	AI, Loss
GSTT2	22	24,299,600-24,303,488	AI, Loss	AI	Loss	Loss, LOH	AI, Loss	AI, Loss	AI	AI	AI	AI, Loss
GSTT2	22	24,322,218-24,326,106	AI, Loss	AI	Loss	Loss, LOH	AI, Loss	AI, Loss	AI	AI	AI	AI, Loss
GSTT2B	22	24,299,600-24,303,393	AI, Loss	AI	Loss	Loss, LOH	AI, Loss	AI, Loss	AI	AI	AI	AI, Loss
GSTT4	22	24,340,594-24,401,807	AI, Loss	AI	Loss	Loss, LOH	AI, Loss	AI, Loss	AI	AI	AI	AI, Loss
GSTTP1	22	24,340,594-24,347,258	AI, Loss	AI	Loss	Loss, LOH	AI, Loss	AI, Loss	AI	AI	AI	AI, Loss
GSTTP2	22	24,385,937-24,401,899	AI, Loss	AI	Loss	Loss, LOH	AI, Loss	AI, Loss	AI	AI	AI	AI, Loss
LINC01639	22	43,796,336-43,805,687	AI, Loss	AI	Loss	Loss, LOH	AI, Loss	AI, Loss	AI	AI	AI	AI, Loss
LOC284889	22	24,235,896-24,241,117	AI, Loss	AI	Loss	Loss, LOH	AI, Loss	AI, Loss	AI	AI	AI	AI, Loss
LOC339685	22	47,741,320-47,769,291	AI, Loss	AI	Loss	Loss, LOH	AI, Loss	AI, Loss	AI	AI	AI	AI, Loss
LOC391322	22	24,373,100-24,374,043	AI, Loss	AI	Loss	Loss, LOH	AI, Loss	AI, Loss	AI	AI	AI	AI, Loss
MIAT	22	27,053,445-27,072,440	AI, Loss	AI	Loss	Loss, LOH	AI, Loss	AI, Loss	AI	AI	AI	AI, Loss

MIF	22	24,236,564-24,237,409	AI, Loss	AI	Loss	Loss, LOH	AI, Loss	AI, Loss	AI	AI	AI	AI, Loss
MIF-AS1	22	24,235,896-24,241,117	AI, Loss	AI	Loss	Loss, LOH	AI, Loss	AI, Loss	AI	AI	AI	AI, Loss
MIR3667	22	49,937,040-49,937,114	AI, Loss	AI	Loss	Loss, LOH	AI, Loss	AI, Loss	AI	AI	AI	AI, Loss
MPPED1	22	43,808,019-43,902,800	AI, Loss	AI	Loss	Loss, LOH	AI, Loss	AI, Loss	AI	AI	AI	AI, Loss
PARVG	22	44,568,835-44,604,349	AI, Loss	AI	Loss	Loss, LOH	AI, Loss	AI, Loss	AI	AI	AI	AI, Loss
SLC2A11	22	24,198,889-24,228,299	AI, Loss	AI	Loss	Loss, LOH	AI, Loss	AI, Loss	AI	AI	AI	AI, Loss
VPREB3	22	24,094,929-24,096,630	AI, Loss	AI	Loss	Loss, LOH	AI, Loss	AI, Loss	AI	AI	AI	AI, Loss
ZNF70	22	24,083,770-24,093,279	AI, Loss	AI	Loss	Loss, LOH	AI, Loss	AI, Loss	AI	AI	AI	AI, Loss
ZBED4	22	50,247,496-50,283,726	AI, Loss	AI	Loss	Loss, LOH	Loss	AI, Loss	AI	AI	AI	AI, Loss
U6	22	46,020,410-46,020,512		AI	Loss	Loss, LOH	AI, Loss	AI, Loss	AI	AI	AI	AI, Loss
SMARCB1	22	24,129,117-24,176,705	AI, Loss	AI, Loss	Loss	Loss, LOH	AI, Loss	AI, Loss	AI	AI	AI	AI, Loss
C22orf15	22	24,105,200-24,108,050	AI, Loss	Loss	Loss	Loss, LOH	AI, Loss	AI, Loss	AI	AI	AI	AI, Loss
CHCHD10	22	24,108,020-24,110,159	AI, Loss	Loss	Loss	Loss, LOH	AI, Loss	AI, Loss	AI	AI	AI	AI, Loss
MMP11	22	24,115,005-24,126,503	AI, Loss	Loss	Loss	Loss, LOH	AI, Loss	AI, Loss	AI	AI	AI	AI, Loss
AK096976	22	24,176,691-24,178,933	AI, Loss	AI		Loss, LOH	AI, Loss	AI, Loss	AI	AI	AI	AI, Loss
BC036874	22	43,671,960-43,679,832	AI, Loss	AI		Loss, LOH	AI, Loss	AI, Loss	AI	AI	AI	AI, Loss
DERL3	22	24,176,689-24,181,315	AI, Loss	AI		Loss, LOH	AI, Loss	AI, Loss	AI	AI	AI	AI, Loss

LOC101927447	22	43,671,947-43,679,832	AI, Loss	AI		Loss, LOH	AI, Loss	AI, Loss	AI	AI	AI	AI, Loss
LOC284912	22	36,023,037-36,031,181	AI, Loss	AI		Loss, LOH	AI, Loss	AI, Loss	AI	AI	AI	AI, Loss
LOC90834	22	50,171,537-50,173,958	AI, Loss	AI		Loss, LOH	AI, Loss	AI, Loss	AI	AI	AI	AI, Loss
SLC5A1	22	32,439,018-32,509,011	AI, Loss	AI		Loss, LOH	AI, Loss	AI, Loss	AI	AI	AI	AI, Loss
PANX2	22	50,609,159-50,618,724	AI, Loss	AI		Loss, LOH	Loss	AI, Loss	AI	AI	AI	AI, Loss
TRABD	22	50,624,340-50,638,028	AI, Loss	AI		Loss, LOH	Loss	AI, Loss	AI	AI	AI	AI, Loss
AK095988	22	45,992,868-45,998,697		AI		Loss, LOH	AI, Loss	AI, Loss	AI	AI	AI	AI, Loss
BC045163	22	46,000,311-46,001,501		AI		Loss, LOH	AI, Loss	AI, Loss	AI	AI	AI	AI, Loss
LINC01589	22	46,000,311-46,001,527		AI		Loss, LOH	AI, Loss	AI, Loss	AI	AI	AI	AI, Loss
AX747137	22	44,220,386-44,222,913	AI, Loss	AI	AI	Loss, LOH	AI, Loss	AI, Loss	AI	AI, Loss	AI	AI, Loss
SULT4A1	22	44,220,386-44,258,378	AI, Loss	AI	AI	Loss, LOH	AI, Loss	AI, Loss	AI	AI, Loss	AI	AI, Loss
AK123891	22	32,896,531-32,898,414	AI, Loss	AI	AI, Loss	Loss, LOH	AI, Loss	AI, Loss	AI	AI, Loss	AI	AI, Loss
ASPHD2	22	26,825,279-26,840,978	AI, Loss	AI	AI, Loss	Loss, LOH	AI, Loss	AI, Loss	AI	AI, Loss	AI	AI, Loss
BC015159	22	27,619,257-27,622,760	AI, Loss	AI	AI, Loss	Loss, LOH	AI, Loss	AI, Loss	AI	AI, Loss	AI	AI, Loss
BC033837	22	49,808,173-50,051,190	AI, Loss	AI	AI, Loss	Loss, LOH	AI, Loss	AI, Loss	AI	AI, Loss	AI	AI, Loss
BC089413	22	22,380,461-22,380,936	AI, Loss	AI	AI, Loss	Loss, LOH	AI, Loss	AI, Loss	AI	AI, Loss	AI	AI, Loss
BMS1P20	22	22,652,462-22,677,324	AI, Loss	AI	AI, Loss	Loss, LOH	AI, Loss	AI, Loss	AI	AI, Loss	AI	AI, Loss

C22orf34	22	49,808,173-50,051,190	AI, Loss	AI	AI, Loss	Loss, LOH	AI, Loss	AI, Loss	AI	AI, Loss	AI	AI, Loss
C22orf43	22	23,950,638-23,974,508	AI, Loss	AI	AI, Loss	Loss, LOH	AI, Loss	AI, Loss	AI	AI, Loss	AI	AI, Loss
CERK	22	47,080,306-47,134,152	AI, Loss	AI	AI, Loss	Loss, LOH	AI, Loss	AI, Loss	AI	AI, Loss	AI	AI, Loss
DKFZp667J0810	22	22,786,692-23,248,968	AI, Loss	AI	AI, Loss	Loss, LOH	AI, Loss	AI, Loss	AI	AI, Loss	AI	AI, Loss
DQ586720	22	23,952,777-23,952,803	AI, Loss	AI	AI, Loss	Loss, LOH	AI, Loss	AI, Loss	AI	AI, Loss	AI	AI, Loss
DRICH1	22	23,950,638-23,974,508	AI, Loss	AI	AI, Loss	Loss, LOH	AI, Loss	AI, Loss	AI	AI, Loss	AI	AI, Loss
FAM230I	22	23,804,268-23,829,167	AI, Loss	AI	AI, Loss	Loss, LOH	AI, Loss	AI, Loss	AI	AI, Loss	AI	AI, Loss
FBXO7	22	32,870,706-32,894,818	AI, Loss	AI	AI, Loss	Loss, LOH	AI, Loss	AI, Loss	AI	AI, Loss	AI	AI, Loss
GUSBP11	22	23,980,674-24,059,610	AI, Loss	AI	AI, Loss	Loss, LOH	AI, Loss	AI, Loss	AI	AI, Loss	AI	AI, Loss
HMOX1	22	35,777,059-35,790,207	AI, Loss	AI	AI, Loss	Loss, LOH	AI, Loss	AI, Loss	AI	AI, Loss	AI	AI, Loss
IGLL1	22	23,915,312-23,922,495	AI, Loss	AI	AI, Loss	Loss, LOH	AI, Loss	AI, Loss	AI	AI, Loss	AI	AI, Loss
ISX	22	35,462,128-35,483,380	AI, Loss	AI	AI, Loss	Loss, LOH	AI, Loss	AI, Loss	AI	AI, Loss	AI	AI, Loss
ISX-AS1	22	35,152,655-35,393,905	AI, Loss	AI	AI, Loss	Loss, LOH	AI, Loss	AI, Loss	AI	AI, Loss	AI	AI, Loss
JB153905	22	32,744,122-32,744,144	AI, Loss	AI	AI, Loss	Loss, LOH	AI, Loss	AI, Loss	AI	AI, Loss	AI	AI, Loss
LARGE-AS1	22	34,120,971-34,146,803	AI, Loss	AI	AI, Loss	Loss, LOH	AI, Loss	AI, Loss	AI	AI, Loss	AI	AI, Loss
LDOC1L	22	44,888,449-44,894,005	AI, Loss	AI	AI, Loss	Loss, LOH	AI, Loss	AI, Loss	AI	AI, Loss	AI	AI, Loss
LINC01310	22	49,262,581-49,294,198	AI, Loss	AI	AI, Loss	Loss, LOH	AI, Loss	AI, Loss	AI	AI, Loss	AI	AI, Loss

LINC01638	22	27,617,307-27,620,688	AI, Loss	AI	AI, Loss	Loss, LOH	AI, Loss	AI, Loss	AI	AI, Loss	AI	AI, Loss
LINC01640	22	33,504,514-33,512,280	AI, Loss	AI	AI, Loss	Loss, LOH	AI, Loss	AI, Loss	AI	AI, Loss	AI	AI, Loss
LINC01656	22	44,839,206-44,840,668	AI, Loss	AI	AI, Loss	Loss, LOH	AI, Loss	AI, Loss	AI	AI, Loss	AI	AI, Loss
LINC01659	22	23,775,750-23,777,258	AI, Loss	AI	AI, Loss	Loss, LOH	AI, Loss	AI, Loss	AI	AI, Loss	AI	AI, Loss
LOC100128946	22	49,262,581-49,294,198	AI, Loss	AI	AI, Loss	Loss, LOH	AI, Loss	AI, Loss	AI	AI, Loss	AI	AI, Loss
LOC100286925	22	22,292,608-22,297,805	AI, Loss	AI	AI, Loss	Loss, LOH	AI, Loss	AI, Loss	AI	AI, Loss	AI	AI, Loss
LOC100507599	22	26,908,497-26,910,533	AI, Loss	AI	AI, Loss	Loss, LOH	AI, Loss	AI, Loss	AI	AI, Loss	AI	AI, Loss
LOC105372977	22	27,583,477-27,585,177	AI, Loss	AI	AI, Loss	Loss, LOH	AI, Loss	AI, Loss	AI	AI, Loss	AI	AI, Loss
LOC284933	22	48,934,711-48,943,199	AI, Loss	AI	AI, Loss	Loss, LOH	AI, Loss	AI, Loss	AI	AI, Loss	AI	AI, Loss
LOC339666	22	32,772,650-32,780,329	AI, Loss	AI	AI, Loss	Loss, LOH	AI, Loss	AI, Loss	AI	AI, Loss	AI	AI, Loss
LOC388882	22	23,804,431-23,829,167	AI, Loss	AI	AI, Loss	Loss, LOH	AI, Loss	AI, Loss	AI	AI, Loss	AI	AI, Loss
LOC96610	22	22,652,462-22,677,324	AI, Loss	AI	AI, Loss	Loss, LOH	AI, Loss	AI, Loss	AI	AI, Loss	AI	AI, Loss
MAPK1	22	22,113,946-22,221,970	AI, Loss	AI	AI, Loss	Loss, LOH	AI, Loss	AI, Loss	AI	AI, Loss	AI	AI, Loss
MCM5	22	35,796,115-35,820,495	AI, Loss	AI	AI, Loss	Loss, LOH	AI, Loss	AI, Loss	AI	AI, Loss	AI	AI, Loss
MIR4535	22	49,176,106-49,176,165	AI, Loss	AI	AI, Loss	Loss, LOH	AI, Loss	AI, Loss	AI	AI, Loss	AI	AI, Loss
PRR5	22	45,064,426-45,133,561	AI, Loss	AI	AI, Loss	Loss, LOH	AI, Loss	AI, Loss	AI	AI, Loss	AI	AI, Loss
PRR5-ARHGAP8	22	45,098,077-45,258,664	AI, Loss	AI	AI, Loss	Loss, LOH	AI, Loss	AI, Loss	AI	AI, Loss	AI	AI, Loss

RFPL3	22	32,750,871-32,757,148	AI, Loss	AI	AI, Loss	Loss, LOH	AI, Loss	AI, Loss	AI	AI, Loss	AI	AI, Loss
RFPL3S	22	32,755,892-32,767,251	AI, Loss	AI	AI, Loss	Loss, LOH	AI, Loss	AI, Loss	AI	AI, Loss	AI	AI, Loss
RGL4	22	24,032,960-24,041,363	AI, Loss	AI	AI, Loss	Loss, LOH	AI, Loss	AI, Loss	AI	AI, Loss	AI	AI, Loss
RTCB	22	32,783,561-32,808,274	AI, Loss	AI	AI, Loss	Loss, LOH	AI, Loss	AI, Loss	AI	AI, Loss	AI	AI, Loss
RTL6	22	44,888,449-44,894,005	AI, Loss	AI	AI, Loss	Loss, LOH	AI, Loss	AI, Loss	AI	AI, Loss	AI	AI, Loss
SCUBE1	22	43,599,228-43,739,394	AI, Loss	AI	AI, Loss	Loss, LOH	AI, Loss	AI, Loss	AI	AI, Loss	AI	AI, Loss
SLC5A4	22	32,614,462-32,651,318	AI, Loss	AI	AI, Loss	Loss, LOH	AI, Loss	AI, Loss	AI	AI, Loss	AI	AI, Loss
SLC5A4-AS1	22	32,601,101-32,669,602	AI, Loss	AI	AI, Loss	Loss, LOH	AI, Loss	AI, Loss	AI	AI, Loss	AI	AI, Loss
SNORA50	22	34,100,771-34,100,908	AI, Loss	AI	AI, Loss	Loss, LOH	AI, Loss	AI, Loss	AI	AI, Loss	AI	AI, Loss
SNORA50B	22	34,100,771-34,100,906	AI, Loss	AI	AI, Loss	Loss, LOH	AI, Loss	AI, Loss	AI	AI, Loss	AI	AI, Loss
TBC1D22A	22	47,158,513-47,571,342	AI, Loss	AI	AI, Loss	Loss, LOH	AI, Loss	AI, Loss	AI	AI, Loss	AI	AI, Loss
VPREB1	22	22,599,191-22,599,927	AI, Loss	AI	AI, Loss	Loss, LOH	AI, Loss	AI, Loss	AI	AI, Loss	AI	AI, Loss
ZDHHC8P1	22	23,732,791-23,744,799	AI, Loss	AI	AI, Loss	Loss, LOH	AI, Loss	AI, Loss	AI	AI, Loss	AI	AI, Loss
ZNF280B	22	22,838,770-22,863,505	AI, Loss	AI	AI, Loss	Loss, LOH	AI, Loss	AI, Loss	AI	AI, Loss	AI	AI, Loss
ARFGAP3	22	43,192,531-43,253,408	Loss	AI	AI, Loss	Loss, LOH	AI, Loss	AI, Loss	AI	AI, Loss	AI	AI, Loss
BC039353	22	43,434,590-43,448,371	Loss	AI	AI, Loss	Loss, LOH	AI, Loss	AI, Loss	AI	AI, Loss	AI	AI, Loss
LOC100506679	22	43,434,590-43,448,371	Loss	AI	AI, Loss	Loss, LOH	AI, Loss	AI, Loss	AI	AI, Loss	AI	AI, Loss

PACSIN2	22	43,265,771-43,411,182	Loss	AI	AI, Loss	Loss, LOH	AI, Loss	AI, Loss	AI	AI, Loss	AI	AI, Loss
TRIOBP	22	38,082,343-38,172,563	Loss	AI	AI, Loss	Loss, LOH	AI, Loss	AI, Loss	AI	AI, Loss	AI	AI, Loss
TTLL1	22	43,435,522-43,485,434	Loss	AI	AI, Loss	Loss, LOH	AI, Loss	AI, Loss	AI	AI, Loss	AI	AI, Loss
AK093107	22	48,027,451-48,251,349	AI, Loss	AI, Loss	AI, Loss	Loss, LOH	AI, Loss	AI, Loss	AI	AI, Loss	AI	AI, Loss
AK131325	22	22,345,495-22,398,332	AI, Loss	AI, Loss	AI, Loss	Loss, LOH	AI, Loss	AI, Loss	AI	AI, Loss	AI	AI, Loss
BPIFC	22	32,809,833-32,860,433	AI, Loss	AI, Loss	AI, Loss	Loss, LOH	AI, Loss	AI, Loss	AI	AI, Loss	AI	AI, Loss
FAM19A5	22	48,885,271-49,147,747	AI, Loss	AI, Loss	AI, Loss	Loss, LOH	AI, Loss	AI, Loss	AI	AI, Loss	AI	AI, Loss
LARGE	22	33,669,061-34,318,584	AI, Loss	AI, Loss	AI, Loss	Loss, LOH	AI, Loss	AI, Loss	AI	AI, Loss	AI	AI, Loss
LARGE1	22	33,668,511-34,318,876	AI, Loss	AI, Loss	AI, Loss	Loss, LOH	AI, Loss	AI, Loss	AI	AI, Loss	AI	AI, Loss
LOC284930	22	48,027,422-48,251,349	AI, Loss	AI, Loss	AI, Loss	Loss, LOH	AI, Loss	AI, Loss	AI	AI, Loss	AI	AI, Loss
MYO18B	22	26,138,110-26,427,007	AI, Loss	AI, Loss	AI, Loss	Loss, LOH	AI, Loss	AI, Loss	AI	AI, Loss	AI	AI, Loss
PRAMENP	22	22,345,495-22,398,332	AI, Loss	AI, Loss	AI, Loss	Loss, LOH	AI, Loss	AI, Loss	AI	AI, Loss	AI	AI, Loss
SEZ6L	22	26,565,439-26,779,563	AI, Loss	AI, Loss	AI, Loss	Loss, LOH	AI, Loss	AI, Loss	AI	AI, Loss	AI	AI, Loss
SYN3	22	32,908,539-33,454,377	AI, Loss	AI, Loss	AI, Loss	Loss, LOH	AI, Loss	AI, Loss	AI	AI, Loss	AI	AI, Loss
TPST2	22	26,917,963-26,986,105	AI, Loss	AI, Loss	AI, Loss	Loss, LOH	AI, Loss	AI, Loss	AI	AI, Loss	AI	AI, Loss
TTC28-AS1	22	28,315,363-28,404,267	AI, Loss	AI, Loss	AI, Loss	Loss, LOH	AI, Loss	AI, Loss	AI	AI, Loss	AI	AI, Loss
MIR548J	22	26,951,177-26,951,289	AI, Loss	Loss	AI, Loss	Loss, LOH	AI, Loss	AI, Loss	AI	AI, Loss	AI	AI, Loss

MIR5739	22	28,855,856-28,855,936	AI, Loss	Loss	AI, Loss	Loss, LOH	AI, Loss	AI, Loss	AI	AI, Loss	AI	AI, Loss
TIMP3	22	33,196,801-33,259,028	AI, Loss	AI	AI, Loss	Loss, LOH	AI, Loss	AI, Loss		AI, Loss	AI	AI, Loss
AK026502	22	27,068,848-27,114,949	AI, Loss	AI	Loss	Loss, LOH	AI, Loss	AI, Loss	AI	AI, Loss	AI	AI, Loss
APOL6	22	36,044,423-36,064,456	AI, Loss	AI	Loss	Loss, LOH	AI, Loss	AI, Loss	AI	AI, Loss	AI	AI, Loss
ARHGAP8	22	45,148,437-45,258,664	AI, Loss	AI	Loss	Loss, LOH	AI, Loss	AI, Loss	AI	AI, Loss	AI	AI, Loss
ATXN10	22	46,067,677-46,241,187	AI, Loss	AI	Loss	Loss, LOH	AI, Loss	AI, Loss	AI	AI, Loss	AI	AI, Loss
C22orf26	22	46,446,338-46,450,024	AI, Loss	AI	Loss	Loss, LOH	AI, Loss	AI, Loss	AI	AI, Loss	AI	AI, Loss
CELSR1	22	46,756,730-46,933,067	AI, Loss	AI	Loss	Loss, LOH	AI, Loss	AI, Loss	AI	AI, Loss	AI	AI, Loss
GRAMD4	22	46,972,984-47,078,653	AI, Loss	AI	Loss	Loss, LOH	AI, Loss	AI, Loss	AI	AI, Loss	AI	AI, Loss
KIAA1671	22	25,423,940-25,593,415	AI, Loss	AI	Loss	Loss, LOH	AI, Loss	AI, Loss	AI	AI, Loss	AI	AI, Loss
LINC00899	22	46,435,786-46,440,748	AI, Loss	AI	Loss	Loss, LOH	AI, Loss	AI, Loss	AI	AI, Loss	AI	AI, Loss
LOC100128531	22	25,498,383-25,508,659	AI, Loss	AI	Loss	Loss, LOH	AI, Loss	AI, Loss	AI	AI, Loss	AI	AI, Loss
LOC150381	22	46,449,725-46,454,402	AI, Loss	AI	Loss	Loss, LOH	AI, Loss	AI, Loss	AI	AI, Loss	AI	AI, Loss
LOC554174	22	46,451,619-46,454,040	AI, Loss	AI	Loss	Loss, LOH	AI, Loss	AI, Loss	AI	AI, Loss	AI	AI, Loss
LOC642648	22	46,460,530-46,466,901	AI, Loss	AI	Loss	Loss, LOH	AI, Loss	AI, Loss	AI	AI, Loss	AI	AI, Loss
LOC730668	22	46,402,495-46,406,657	AI, Loss	AI	Loss	Loss, LOH	AI, Loss	AI, Loss	AI	AI, Loss	AI	AI, Loss
MIATNB	22	27,068,805-27,176,856	AI, Loss	AI	Loss	Loss, LOH	AI, Loss	AI, Loss	AI	AI, Loss	AI	AI, Loss

MIR3619	22	46,486,923-46,487,006	AI, Loss	AI	Loss	Loss, LOH	AI, Loss	AI, Loss	AI	AI, Loss	AI	AI, Loss
MIR4762	22	46,156,403-46,156,478	AI, Loss	AI	Loss	Loss, LOH	AI, Loss	AI, Loss	AI	AI, Loss	AI	AI, Loss
MN1	22	28,144,264-28,197,486	AI, Loss	AI	Loss	Loss, LOH	AI, Loss	AI, Loss	AI	AI, Loss	AI	AI, Loss
PRR34-AS1	22	46,449,725-46,454,402	AI, Loss	AI	Loss	Loss, LOH	AI, Loss	AI, Loss	AI	AI, Loss	AI	AI, Loss
TXN2	22	36,863,092-36,877,687	AI, Loss	AI	Loss	Loss, LOH	AI, Loss	AI, Loss	AI	AI, Loss	AI	AI, Loss
WNT7B	22	46,316,247-46,373,008	AI, Loss	AI	Loss	Loss, LOH	AI, Loss	AI, Loss	AI	AI, Loss	AI	AI, Loss
CRELD2	22	50,312,277-50,321,188	AI, Loss	AI	Loss	Loss, LOH	Loss	AI, Loss	AI	AI, Loss	AI	AI, Loss
IL17REL	22	50,432,941-50,451,055	AI, Loss	AI	Loss	Loss, LOH	Loss	AI, Loss	AI	AI, Loss	AI	AI, Loss
MIR6821	22	50,356,513-50,356,587	AI, Loss	AI	Loss	Loss, LOH	Loss	AI, Loss	AI	AI, Loss	AI	AI, Loss
MLC1	22	50,497,819-50,524,358	AI, Loss	AI	Loss	Loss, LOH	Loss	AI, Loss	AI	AI, Loss	AI	AI, Loss
MOV10L1	22	50,528,434-50,600,116	AI, Loss	AI	Loss	Loss, LOH	Loss	AI, Loss	AI	AI, Loss	AI	AI, Loss
PHF21B	22	45,277,042-45,405,809	AI, Loss	AI	Loss	Loss, LOH	Loss	AI, Loss	AI	AI, Loss	AI	AI, Loss
PIM3	22	50,354,142-50,357,720	AI, Loss	AI	Loss	Loss, LOH	Loss	AI, Loss	AI	AI, Loss	AI	AI, Loss
SELENOO	22	50,639,407-50,656,045	AI, Loss	AI	Loss	Loss, LOH	Loss	AI, Loss	AI	AI, Loss	AI	AI, Loss
SELO	22	50,639,407-50,656,045	AI, Loss	AI	Loss	Loss, LOH	Loss	AI, Loss	AI	AI, Loss	AI	AI, Loss
TTLL8	22	50,457,003-50,496,656	AI, Loss	AI	Loss	Loss, LOH	Loss	AI, Loss	AI	AI, Loss	AI	AI, Loss
CRYBB3	22	25,595,816-25,603,326	AI, Loss	AI, Loss	Loss	Loss, LOH	AI, Loss	AI, Loss	AI	AI, Loss	AI	AI, Loss

PITPNB	22	28,247,656-28,315,294	AI, Loss	AI, Loss	Loss	Loss, LOH	AI, Loss	AI, Loss	AI	AI, Loss	AI	AI, Loss
AX748308	22	28,276,021-28,277,106	AI, Loss	Loss	Loss	Loss, LOH	AI, Loss	AI, Loss	AI	AI, Loss	AI	AI, Loss
MIR3199-1	22	28,316,512-28,316,600	AI, Loss	Loss	Loss	Loss, LOH	AI, Loss	AI, Loss	AI	AI, Loss	AI	AI, Loss
MIR3199-2	22	28,316,513-28,316,599	AI, Loss	Loss	Loss	Loss, LOH	AI, Loss	AI, Loss	AI	AI, Loss	AI	AI, Loss
KIAA1644	22	44,639,556-44,708,731	AI, Loss	AI		Loss, LOH	AI, Loss	AI, Loss	AI	AI, Loss	AI	AI, Loss
SHISAL1	22	44,639,556-44,708,731	AI, Loss	AI		Loss, LOH	AI, Loss	AI, Loss	AI	AI, Loss	AI	AI, Loss
ADRBK2	22	25,960,860-26,125,258	AI, Loss	AI	AI, Loss	Loss, LOH	AI, Loss	AI, Loss	AI	Loss	AI	AI, Loss
BC037972	22	47,857,047-47,882,860	AI, Loss	AI	AI, Loss	Loss, LOH	AI, Loss	AI, Loss	AI	Loss	AI	AI, Loss
BCR	22	23,522,551-23,660,224	AI, Loss	AI	AI, Loss	Loss, LOH	AI, Loss	AI, Loss	AI	Loss	AI	AI, Loss
DQ576853	22	24,658,457-24,658,483	AI, Loss	AI	AI, Loss	Loss, LOH	AI, Loss	AI, Loss	AI	Loss	AI	AI, Loss
FBXW4P1	22	23,604,953-23,607,186	AI, Loss	AI	AI, Loss	Loss, LOH	AI, Loss	AI, Loss	AI	Loss	AI	AI, Loss
GGT5	22	24,615,621-24,641,110	AI, Loss	AI	AI, Loss	Loss, LOH	AI, Loss	AI, Loss	AI	Loss	AI	AI, Loss
GRK3	22	25,960,860-26,125,258	AI, Loss	AI	AI, Loss	Loss, LOH	AI, Loss	AI, Loss	AI	Loss	AI	AI, Loss
LINC00207	22	44,965,219-44,968,329	AI, Loss	AI	AI, Loss	Loss, LOH	AI, Loss	AI, Loss	AI	Loss	AI	AI, Loss
LINC01644	22	47,857,047-47,882,860	AI, Loss	AI	AI, Loss	Loss, LOH	AI, Loss	AI, Loss	AI	Loss	AI	AI, Loss
POM121L9P	22	24,647,588-24,661,492	AI, Loss	AI	AI, Loss	Loss, LOH	AI, Loss	AI, Loss	AI	Loss	AI	AI, Loss
RAB36	22	23,487,510-23,507,830	AI, Loss	AI	AI, Loss	Loss, LOH	AI, Loss	AI, Loss	AI	Loss	AI	AI, Loss

UNQ2565	22	24,647,952-24,649,256	AI, Loss	AI	AI, Loss	Loss, LOH	AI, Loss	AI, Loss	AI	Loss	AI	AI, Loss
GUCD1	22	24,936,390-24,951,903	AI, Loss	AI	AI, Loss	Loss, LOH		AI, Loss	AI	Loss	AI	AI, Loss
TSPO	22	43,547,519-43,559,248	Loss	AI	AI, Loss	Loss, LOH	AI, Loss	AI, Loss	AI	Loss	AI	AI, Loss
ADORA2A	22	24,813,708-24,838,328	AI, Loss	AI, Loss	AI, Loss	Loss, LOH	AI, Loss	AI, Loss	AI	Loss	AI	AI, Loss
ADORA2A-AS1	22	24,825,175-24,891,042	AI, Loss	AI, Loss	AI, Loss	Loss, LOH	AI, Loss	AI, Loss	AI	Loss	AI	AI, Loss
SPECC1L	22	24,666,784-24,838,325	AI, Loss	AI, Loss	AI, Loss	Loss, LOH	AI, Loss	AI, Loss	AI	Loss	AI	AI, Loss
SPECC1L-ADORA2A	22	24,666,784-24,838,328	AI, Loss	AI, Loss	AI, Loss	Loss, LOH	AI, Loss	AI, Loss	AI	Loss	AI	AI, Loss
UPB1	22	24,863,205-24,922,553	AI, Loss	AI, Loss	AI, Loss	Loss, LOH	AI, Loss	AI, Loss	AI	Loss	AI	AI, Loss
EU036692	22	24,827,818-24,828,694	AI, Loss	Loss	AI, Loss	Loss, LOH	AI, Loss	AI, Loss	AI	Loss	AI	AI, Loss
APOL1	22	36,649,116-36,663,577	AI, Loss	AI	Loss	Loss, LOH	AI, Loss	AI, Loss	AI	Loss	AI	AI, Loss
APOL2	22	36,622,254-36,636,000	AI, Loss	AI	Loss	Loss, LOH	AI, Loss	AI, Loss	AI	Loss	AI	AI, Loss
APOL3	22	36,536,370-36,562,225	AI, Loss	AI	Loss	Loss, LOH	AI, Loss	AI, Loss	AI	Loss	AI	AI, Loss
APOL4	22	36,585,175-36,600,879	AI, Loss	AI	Loss	Loss, LOH	AI, Loss	AI, Loss	AI	Loss	AI	AI, Loss
APOL5	22	36,113,918-36,125,529	AI, Loss	AI	Loss	Loss, LOH	AI, Loss	AI, Loss	AI	Loss	AI	AI, Loss
AX747758	22	36,633,472-36,635,231	AI, Loss	AI	Loss	Loss, LOH	AI, Loss	AI, Loss	AI	Loss	AI	AI, Loss
CRYBB2	22	25,615,611-25,627,836	AI, Loss	AI	Loss	Loss, LOH	AI, Loss	AI, Loss	AI	Loss	AI	AI, Loss
FLJ32756	22	47,309,987-47,311,916	AI, Loss	AI	Loss	Loss, LOH	AI, Loss	AI, Loss	AI	Loss	AI	AI, Loss

MYH9	22	36,677,322-36,784,112	AI, Loss	AI	Loss	Loss, LOH	AI, Loss	AI, Loss	AI	Loss	AI	AI, Loss
TBC1D22A-AS1	22	47,309,987-47,311,938	AI, Loss	AI	Loss	Loss, LOH	AI, Loss	AI, Loss	AI	Loss	AI	AI, Loss
TRMU	22	46,731,297-46,753,237	AI, Loss	AI	Loss	Loss, LOH	AI, Loss	AI, Loss	AI	Loss	AI	AI, Loss
ALG12	22	50,296,853-50,312,106	AI, Loss	AI	Loss	Loss, LOH	Loss	AI, Loss	AI	Loss	AI	AI, Loss
RBFOX2	22	36,134,780-36,424,585	AI, Loss	AI, Loss	Loss	Loss, LOH	AI, Loss	AI, Loss	AI	Loss	AI	AI, Loss
AX748195	22	36,730,923-36,732,334	AI, Loss	AI		Loss, LOH	AI, Loss	AI, Loss	AI	Loss	AI	AI, Loss
BC039485	22	48,027,451-48,038,545	AI, Loss	AI	AI, Loss	Loss, LOH	AI, Loss	AI, Loss	AI		AI	AI, Loss
BCR	22	22,979,652-22,981,152	AI, Loss	AI	AI, Loss	Loss, LOH	AI, Loss	AI, Loss	AI		AI	AI, Loss
CES5AP1	22	23,701,792-23,724,313	AI, Loss	AI	AI, Loss	Loss, LOH	AI, Loss	AI, Loss	AI		AI	AI, Loss
DQ575049	22	23,236,274-23,238,011	AI, Loss	AI	AI, Loss	Loss, LOH	AI, Loss	AI, Loss	AI		AI	AI, Loss
DQ597441	22	22,984,879-22,984,909	AI, Loss	AI	AI, Loss	Loss, LOH	AI, Loss	AI, Loss	AI		AI	AI, Loss
GGTLC2	22	22,987,090-22,990,368	AI, Loss	AI	AI, Loss	Loss, LOH	AI, Loss	AI, Loss	AI		AI	AI, Loss
GNAZ	22	23,412,539-23,467,224	AI, Loss	AI	AI, Loss	Loss, LOH	AI, Loss	AI, Loss	AI		AI	AI, Loss
IGLL5	22	23,229,959-23,238,013	AI, Loss	AI	AI, Loss	Loss, LOH	AI, Loss	AI, Loss	AI		AI	AI, Loss
LINC00898	22	48,016,791-48,027,318	AI, Loss	AI	AI, Loss	Loss, LOH	AI, Loss	AI, Loss	AI		AI	AI, Loss
LINC02556	22	23,668,802-23,670,680	AI, Loss	AI	AI, Loss	Loss, LOH	AI, Loss	AI, Loss	AI		AI	AI, Loss
LL22NC03-63E9.3	22	22,901,749-22,909,004	AI, Loss	AI	AI, Loss	Loss, LOH	AI, Loss	AI, Loss	AI		AI	AI, Loss

LOC648691	22	22,901,755-22,909,007	AI, Loss	AI	AI, Loss	Loss, LOH	AI, Loss	AI, Loss	AI		AI	AI, Loss
MIR5571	22	23,228,446-23,228,559	AI, Loss	AI	AI, Loss	Loss, LOH	AI, Loss	AI, Loss	AI		AI	AI, Loss
MIR650	22	23,165,269-23,165,365	AI, Loss	AI	AI, Loss	Loss, LOH	AI, Loss	AI, Loss	AI		AI	AI, Loss
POM121L1P	22	22,974,027-22,987,012	AI, Loss	AI	AI, Loss	Loss, LOH	AI, Loss	AI, Loss	AI		AI	AI, Loss
PRAME	22	22,890,117-22,901,768	AI, Loss	AI	AI, Loss	Loss, LOH	AI, Loss	AI, Loss	AI		AI	AI, Loss
RSPH14	22	23,401,592-23,484,241	AI, Loss	AI	AI, Loss	Loss, LOH	AI, Loss	AI, Loss	AI		AI	AI, Loss
RTDR1	22	23,401,592-23,484,241	AI, Loss	AI	AI, Loss	Loss, LOH	AI, Loss	AI, Loss	AI		AI	AI, Loss
ZNF280A	22	22,868,060-22,874,624	AI, Loss	AI	AI, Loss	Loss, LOH	AI, Loss	AI, Loss	AI		AI	AI, Loss
AK056490	22	43,593,288-43,595,059	Loss	AI	AI, Loss	Loss, LOH	AI, Loss	AI, Loss	AI		AI	AI, Loss
FLJ30901	22	43,593,285-43,597,160	Loss	AI	AI, Loss	Loss, LOH	AI, Loss	AI, Loss	AI		AI	AI, Loss
LOC105373051	22	43,608,679-43,609,667	Loss	AI	AI, Loss	Loss, LOH	AI, Loss	AI, Loss	AI		AI	AI, Loss
TTLL12	22	43,562,627-43,583,137	Loss	AI	AI, Loss	Loss, LOH	AI, Loss	AI, Loss	AI		AI	AI, Loss
BC021234	22	46,726,771-46,730,190	AI, Loss	AI	Loss	Loss, LOH	AI, Loss	AI, Loss	AI		AI	AI, Loss
BC069212	22	46,726,771-46,729,595	AI, Loss	AI	Loss	Loss, LOH	AI, Loss	AI, Loss	AI		AI	AI, Loss
MIR6819	22	36,682,892-36,682,953	AI, Loss	AI	Loss	Loss, LOH	AI, Loss	AI, Loss	AI		AI	AI, Loss
HPS4	22	26,839,074-26,879,829	AI, Loss	AI	AI, Loss	Loss, LOH	AI, Loss	AI, Loss	AI	AI, Loss	AI, Loss	AI, Loss
LINC01399	22	35,515,816-35,627,049	AI, Loss	AI	AI, Loss	Loss, LOH	AI, Loss	AI, Loss	AI	AI, Loss	AI, Loss	AI, Loss

PPM1F	22	22,273,791-22,307,250	AI, Loss	AI	AI, Loss	Loss, LOH	AI, Loss	AI, Loss	AI	AI, Loss	AI, Loss	AI, Loss
SRRD	22	26,879,845-26,887,904	AI, Loss	AI	AI, Loss	Loss, LOH	AI, Loss	AI, Loss	AI	AI, Loss	AI, Loss	AI, Loss
TFIP11	22	26,887,205-26,908,472	AI, Loss	AI	AI, Loss	Loss, LOH	AI, Loss	AI, Loss	AI	AI, Loss	AI, Loss	AI, Loss
TOP3B	22	22,311,396-22,337,240	AI, Loss	AI	AI, Loss	Loss, LOH	AI, Loss	AI, Loss	AI	AI, Loss	AI, Loss	AI, Loss
EFCAB6	22	43,924,623-44,208,217	AI, Loss	AI, Loss	AI, Loss	Loss, LOH	AI, Loss	AI, Loss	AI	AI, Loss	AI, Loss	AI, Loss
TTC28	22	28,374,001-29,075,853	AI, Loss	AI, Loss	AI, Loss	Loss, LOH	AI, Loss	AI, Loss	AI	AI, Loss	AI, Loss	AI, Loss
PNPLA5	22	44,275,557-44,287,893	AI, Loss	AI	AI, Loss	Loss, LOH	Loss	AI, Loss		AI, Loss	AI, Loss	AI, Loss
MIRLET7BHG	22	46,449,725-46,509,808	AI, Loss	AI	Loss	Loss, LOH	AI, Loss	AI, Loss	AI	AI, Loss	AI, Loss	AI, Loss
SMC1B	22	45,739,944-45,809,500	AI, Loss	AI	Loss	Loss, LOH	AI, Loss	AI, Loss	AI	AI, Loss	AI, Loss	AI, Loss
RIBC2	22	45,809,571-45,828,302	AI, Loss	AI, Loss	Loss	Loss, LOH	AI, Loss	AI, Loss	AI	AI, Loss	AI, Loss	AI, Loss
SNRPD3	22	24,951,617-24,978,854	AI, Loss	AI	AI, Loss	Loss, LOH	AI, Loss	AI, Loss	AI	Loss	AI, Loss	AI, Loss
GTSE1	22	46,692,637-46,726,707	AI, Loss	AI	Loss	Loss, LOH	AI, Loss	AI, Loss	AI	Loss	AI, Loss	AI, Loss
DM119500	22	46,508,631-46,508,653	AI, Loss	AI	Loss	Loss, LOH	AI, Loss	AI, Loss	AI	AI, Loss	Loss	AI, Loss
MIR4763	22	46,509,445-46,509,537	AI, Loss	AI	Loss	Loss, LOH	AI, Loss	AI, Loss	AI	AI, Loss	Loss	AI, Loss
MIRLET7A3	22	46,508,628-46,508,702	AI, Loss	AI	Loss	Loss, LOH	AI, Loss	AI, Loss	AI	AI, Loss	Loss	AI, Loss
MIRLET7B	22	46,509,565-46,509,648	AI, Loss	AI	Loss	Loss, LOH	AI, Loss	AI, Loss	AI	AI, Loss	Loss	AI, Loss
PPARA	22	46,546,436-46,639,653	AI, Loss	AI	Loss	Loss, LOH	AI, Loss	AI, Loss	AI	AI, Loss	Loss	AI, Loss

FAM211B	22	24,981,590-24,989,035	AI, Loss	AI, Loss	AI, Loss	Loss, LOH	AI, Loss	AI, Loss	AI	Loss	Loss	AI, Loss
GGT1	22	24,979,717-25,024,972	AI, Loss	AI, Loss	AI, Loss	Loss, LOH	AI, Loss	AI, Loss	AI	Loss	Loss	AI, Loss
LRRC75B	22	24,981,590-24,989,035	AI, Loss	AI, Loss	AI, Loss	Loss, LOH	AI, Loss	AI, Loss	AI	Loss	Loss	AI, Loss
PIWIL3	22	25,115,000-25,170,687	AI, Loss	AI, Loss	AI, Loss	Loss, LOH	AI, Loss	AI, Loss	AI	Loss	Loss	AI, Loss
TOP1P2	22	25,160,467-25,161,986	AI, Loss	Loss	AI, Loss	Loss, LOH	AI, Loss	AI, Loss	AI	Loss	Loss	AI, Loss
BCRP3	22	25,028,881-25,049,327	AI, Loss	AI	AI, Loss	Loss, LOH	AI, Loss	AI, Loss	AI	Loss		AI, Loss
CRYBB2P1	22	25,844,053-25,907,585	AI, Loss	AI	AI, Loss	Loss, LOH	AI, Loss	AI, Loss	AI	Loss		AI, Loss
DKFZp434K191	22	25,046,352-25,047,058	AI, Loss	AI	AI, Loss	Loss, LOH	AI, Loss	AI, Loss	AI	Loss		AI, Loss
MIR6817	22	25,851,612-25,851,678	AI, Loss	AI	AI, Loss	Loss, LOH	AI, Loss	AI, Loss	AI	Loss		AI, Loss
POM121L10P	22	25,041,132-25,055,114	AI, Loss	AI	AI, Loss	Loss, LOH	AI, Loss	AI, Loss	AI	Loss		AI, Loss
CDPF1	22	46,639,909-46,646,193	AI, Loss	AI	Loss	Loss, LOH	AI, Loss	AI, Loss	AI	Loss		AI, Loss
LRP5L	22	25,747,384-25,777,544	AI, Loss	AI	AI, Loss	Loss, LOH	AI, Loss	AI, Loss	AI			AI, Loss
BC040576	22	25,677,208-25,679,061	AI, Loss	AI	Loss	Loss, LOH	AI, Loss	AI, Loss	AI			AI, Loss
GTSE1-AS1	22	46,691,039-46,692,557	AI, Loss	AI	Loss	Loss, LOH	AI, Loss	AI, Loss	AI			AI, Loss
GTSE1-DT	22	46,691,039-46,692,557	AI, Loss	AI	Loss	Loss, LOH	AI, Loss	AI, Loss	AI			AI, Loss
IGLL3P	22	25,714,223-25,716,193	AI, Loss	AI	Loss	Loss, LOH	AI, Loss	AI, Loss	AI			AI, Loss
PKDREJ	22	46,651,559-46,659,219	AI, Loss	AI	Loss	Loss, LOH	AI, Loss	AI, Loss	AI			AI, Loss

TTC38	22	46,663,860-46,689,905	AI, Loss	AI	Loss	Loss, LOH	AI, Loss	AI, Loss	AI			AI, Loss
CASTOR1	22	30,681,106-30,685,616	AI, Loss	AI	AI, Loss	Loss, LOH	AI, Loss	AI, Loss	AI	AI	AI	Loss
GATSL3	22	30,681,106-30,695,540	AI, Loss	AI	AI, Loss	Loss, LOH	AI, Loss	AI, Loss	AI	AI	AI	Loss
LOC105372990	22	30,828,672-30,832,488	AI, Loss	AI	AI, Loss	Loss, LOH	AI, Loss	AI, Loss	AI	AI	AI	Loss
MTFP1	22	30,805,175-30,825,041	AI, Loss	AI	AI, Loss	Loss, LOH	AI, Loss	AI, Loss	AI	AI	AI	Loss
SDC4P	22	30,877,276-30,877,743	AI, Loss	AI	AI, Loss	Loss, LOH	AI, Loss	AI, Loss	AI	AI	AI	Loss
SEC14L2	22	30,792,929-30,821,291	AI, Loss	AI	AI, Loss	Loss, LOH	AI, Loss	AI, Loss	AI	AI	AI	Loss
SEC14L3	22	30,855,215-30,868,034	AI, Loss	AI	AI, Loss	Loss, LOH	AI, Loss	AI, Loss	AI	AI	AI	Loss
SF3A1	22	30,727,976-30,752,936	AI, Loss	AI	AI, Loss	Loss, LOH	AI, Loss	AI, Loss	AI	AI	AI	Loss
TBC1D10A	22	30,687,978-30,722,955	AI, Loss	AI	AI, Loss	Loss, LOH	AI, Loss	AI, Loss	AI	AI	AI	Loss
BC036921	22	39,066,472-39,077,825	Loss	AI	AI, Loss	Loss, LOH	AI, Loss	AI, Loss	AI	AI	AI	Loss
CBY1	22	39,052,657-39,069,855	Loss	AI	AI, Loss	Loss, LOH	AI, Loss	AI, Loss	AI	AI	AI	Loss
GTPBP1	22	39,101,806-39,129,592	Loss	AI	AI, Loss	Loss, LOH	AI, Loss	AI, Loss	AI	AI	AI	Loss
JOSD1	22	39,081,547-39,097,423	Loss	AI	AI, Loss	Loss, LOH	AI, Loss	AI, Loss	AI	AI	AI	Loss
TOMM22	22	39,077,953-39,080,766	Loss	AI	AI, Loss	Loss, LOH	AI, Loss	AI, Loss	AI	AI	AI	Loss
CCDC157	22	30,752,623-30,774,644	AI, Loss	AI, Loss	AI, Loss	Loss, LOH	AI, Loss	AI, Loss	AI	AI	AI	Loss
KIAA1656	22	30,764,799-30,773,894	AI, Loss	AI, Loss	AI, Loss	Loss, LOH	AI, Loss	AI, Loss	AI	AI	AI	Loss

RNF215	22	30,774,802-30,783,302	AI, Loss	AI, Loss	AI, Loss	Loss, LOH	AI, Loss	AI, Loss	AI	AI	AI	Loss
FAM227A	22	38,974,124-39,052,634	Loss	AI, Loss	AI, Loss	Loss, LOH	AI, Loss	AI, Loss	AI	AI	AI	Loss
LOC105373031	22	38,966,504-38,967,363	Loss	Loss	AI, Loss	Loss, LOH	AI, Loss	AI, Loss	AI	AI	AI	Loss
BC069815	22	31,601,248-31,601,602	AI, Loss	AI	Loss	Loss, LOH	AI, Loss	AI, Loss	AI	AI	AI	Loss
MIR3928	22	31,556,047-31,556,105	AI, Loss	AI	Loss	Loss, LOH	AI, Loss	AI, Loss	AI	AI	AI	Loss
PLA2G3	22	31,530,792-31,536,593	AI, Loss	AI	Loss	Loss, LOH	AI, Loss	AI, Loss	AI	AI	AI	Loss
RNF185	22	31,556,137-31,603,005	AI, Loss	AI	Loss	Loss, LOH	AI, Loss	AI, Loss	AI	AI	AI	Loss
Y_RNA	22	31,626,056-31,626,158	AI, Loss	AI	Loss	Loss, LOH	AI, Loss	AI, Loss	AI	AI	AI	Loss
APOBEC3D	22	39,417,117-39,429,256	Loss	AI	Loss	Loss, LOH	AI, Loss	AI, Loss	AI	AI	AI	Loss
APOBEC3F	22	39,436,672-39,451,975	Loss	AI	Loss	Loss, LOH	AI, Loss	AI, Loss	AI	AI	AI	Loss
APOBEC3G	22	39,436,672-39,483,748	Loss	AI	Loss	Loss, LOH	AI, Loss	AI, Loss	AI	AI	AI	Loss
CBX6	22	39,257,431-39,268,339	Loss	AI	Loss	Loss, LOH	AI, Loss	AI, Loss	AI	AI	AI	Loss
NPTXR	22	39,214,455-39,240,017	Loss	AI	Loss	Loss, LOH	AI, Loss	AI, Loss	AI	AI	AI	Loss
TCF20	22	42,556,018-42,679,933	Loss	AI	Loss	Loss, LOH	AI, Loss	AI, Loss	AI	AI	AI	Loss
CBX7	22	39,526,778-39,548,655	Loss	AI	Loss	Loss, LOH	Loss	AI, Loss	AI	AI	AI	Loss
CYP2D7P1	22	42,536,213-42,540,575	Loss	AI	Loss	Loss, LOH	Loss	AI, Loss	AI	AI	AI	Loss
PDGFB	22	39,619,363-39,641,060	Loss	AI	Loss	Loss, LOH	Loss	AI, Loss	AI	AI	AI	Loss

RPL3	22	39,708,886-39,716,391	Loss	AI	Loss	Loss, LOH	Loss	AI, Loss	AI	AI	AI	Loss
SNORD43	22	39,715,056-39,715,118	Loss	AI	Loss	Loss, LOH	Loss	AI, Loss	AI	AI	AI	Loss
SYNGR1	22	39,745,953-39,781,593	Loss	AI	Loss	Loss, LOH	Loss	AI, Loss	AI	AI	AI	Loss
LOC388906	22	42,665,758-42,670,868	Loss	AI		Loss, LOH	AI, Loss	AI, Loss	AI	AI	AI	Loss
OGFRP1	22	42,665,758-42,670,868	Loss	AI		Loss, LOH	AI, Loss	AI, Loss	AI	AI	AI	Loss
APOBEC3H	22	39,493,228-39,500,072	Loss	AI		Loss, LOH	Loss	AI, Loss	AI	AI	AI	Loss
SNORD139	22	39,712,846-39,712,901	Loss	AI		Loss, LOH	Loss	AI, Loss	AI	AI	AI	Loss
SNORD83A	22	39,711,217-39,711,312	Loss	AI		Loss, LOH	Loss	AI, Loss	AI	AI	AI	Loss
SNORD83B	22	39,709,823-39,709,916	Loss	AI		Loss, LOH	Loss	AI, Loss	AI	AI	AI	Loss
EWSR1	22	29,663,997-29,696,515	AI, Loss	AI	AI, Loss	Loss, LOH	AI, Loss	AI, Loss	AI	AI, Loss	AI	Loss
GAS2L1	22	29,702,984-29,708,778	AI, Loss	AI	AI, Loss	Loss, LOH	AI, Loss	AI, Loss	AI	AI, Loss	AI	Loss
KREMEN1	22	29,469,065-29,564,321	AI, Loss	AI	AI, Loss	Loss, LOH	AI, Loss	AI, Loss	AI	AI, Loss	AI	Loss
LIF	22	30,636,435-30,642,840	AI, Loss	AI	AI, Loss	Loss, LOH	AI, Loss	AI, Loss	AI	AI, Loss	AI	Loss
LIF-AS1	22	30,635,182-30,636,527	AI, Loss	AI	AI, Loss	Loss, LOH	AI, Loss	AI, Loss	AI	AI, Loss	AI	Loss
LOC91370	22	30,642,198-30,642,982	AI, Loss	AI	AI, Loss	Loss, LOH	AI, Loss	AI, Loss	AI	AI, Loss	AI	Loss
MGC20647	22	30,642,198-30,642,982	AI, Loss	AI	AI, Loss	Loss, LOH	AI, Loss	AI, Loss	AI	AI, Loss	AI	Loss
OSM	22	30,658,816-30,662,832	AI, Loss	AI	AI, Loss	Loss, LOH	AI, Loss	AI, Loss	AI	AI, Loss	AI	Loss

SEC14L4	22	30,884,897-30,901,698	AI, Loss	AI	AI, Loss	Loss, LOH	AI, Loss	AI, Loss	AI	AI, Loss	AI	Loss
FAM83F	22	40,390,952-40,426,043	Loss	AI	AI, Loss	Loss, LOH	AI, Loss	AI, Loss	AI	AI, Loss	AI	Loss
GRAP2	22	40,297,085-40,369,346	Loss	AI	AI, Loss	Loss, LOH	AI, Loss	AI, Loss	AI	AI, Loss	AI	Loss
SUN2	22	39,130,718-39,190,161	Loss	AI	AI, Loss	Loss, LOH	AI, Loss	AI, Loss	AI	AI, Loss	AI	Loss
EMID1	22	29,601,900-29,655,586	AI, Loss	AI, Loss	AI, Loss	Loss, LOH	AI, Loss	AI, Loss	AI	AI, Loss	AI	Loss
HORMAD2-AS1	22	30,404,730-30,476,469	AI, Loss	AI, Loss	AI, Loss	Loss, LOH	AI, Loss	AI, Loss	AI	AI, Loss	AI	Loss
RHBDD3	22	29,655,840-29,664,152	AI, Loss	AI, Loss	AI, Loss	Loss, LOH	AI, Loss	AI, Loss	AI	AI, Loss	AI	Loss
SGSM1	22	25,202,135-25,322,813	AI, Loss	AI, Loss	AI, Loss	Loss, LOH	AI, Loss	AI, Loss	AI	AI, Loss	AI	Loss
MTMR3	22	30,279,157-30,426,857	AI, Loss	AI, Loss	AI, Loss	Loss, LOH	AI, Loss	AI, Loss	AI, Loss	AI, Loss	AI	Loss
MIR6818	22	30,403,037-30,403,102	AI, Loss	AI	AI, Loss	Loss, LOH	AI, Loss	AI, Loss	Loss	AI, Loss	AI	Loss
AP1B1	22	29,723,668-29,784,572	AI, Loss	AI	AI, Loss	Loss, LOH	AI, Loss	AI, Loss		AI, Loss	AI	Loss
ASCC2	22	30,184,596-30,234,293	AI, Loss	AI	AI, Loss	Loss, LOH	AI, Loss	AI, Loss		AI, Loss	AI	Loss
CABP7	22	30,116,343-30,127,820	AI, Loss	AI	AI, Loss	Loss, LOH	AI, Loss	AI, Loss		AI, Loss	AI	Loss
MIR3653	22	29,729,146-29,729,256	AI, Loss	AI	AI, Loss	Loss, LOH	AI, Loss	AI, Loss		AI, Loss	AI	Loss
NF2	22	29,999,544-30,094,589	AI, Loss	AI	AI, Loss	Loss, LOH	AI, Loss	AI, Loss		AI, Loss	AI	Loss
RASL10A	22	29,708,921-29,711,745	AI, Loss	AI	AI, Loss	Loss, LOH	AI, Loss	AI, Loss		AI, Loss	AI	Loss
RFPL1	22	29,834,571-29,838,444	AI, Loss	AI	AI, Loss	Loss, LOH	AI, Loss	AI, Loss		AI, Loss	AI	Loss

RFPL1S	22	29,833,003-29,838,118	AI, Loss	AI	AI, Loss	Loss, LOH	AI, Loss	AI, Loss		AI, Loss	AI	Loss
SNORD125	22	29,729,151-29,729,247	AI, Loss	AI	AI, Loss	Loss, LOH	AI, Loss	AI, Loss		AI, Loss	AI	Loss
THOC5	22	29,904,155-29,949,736	AI, Loss	AI	AI, Loss	Loss, LOH	AI, Loss	AI, Loss		AI, Loss	AI	Loss
UQCR10	22	30,163,357-30,166,402	AI, Loss	AI	AI, Loss	Loss, LOH	AI, Loss	AI, Loss		AI, Loss	AI	Loss
ZMAT5	22	30,126,944-30,163,000	AI, Loss	AI	AI, Loss	Loss, LOH	AI, Loss	AI, Loss		AI, Loss	AI	Loss
LIMK2	22	31,608,249-31,676,066	AI, Loss	AI	Loss	Loss, LOH	AI, Loss	AI, Loss	AI	AI, Loss	AI	Loss
INPP5J	22	31,503,960-31,530,683	AI, Loss	AI	Loss	Loss, LOH	Loss	AI, Loss	AI	AI, Loss	AI	Loss
APOBEC3C	22	39,410,264-39,414,825	Loss	AI	Loss	Loss, LOH	AI, Loss	AI, Loss	AI	AI, Loss	AI	Loss
TAB1	22	39,745,953-39,833,132	Loss	AI	Loss	Loss, LOH	AI, Loss	AI, Loss	AI	AI, Loss	AI	Loss
CYP2D6	22	42,522,500-42,526,883	Loss	AI	Loss	Loss, LOH	Loss	AI, Loss	AI	AI, Loss	AI	Loss
LOC101929829	22	42,522,500-42,526,883	Loss	AI	Loss	Loss, LOH	Loss	AI, Loss	AI	AI, Loss	AI	Loss
NDUFA6-AS1	22	42,486,936-42,532,702	Loss	AI	Loss	Loss, LOH	Loss	AI, Loss	AI	AI, Loss	AI	Loss
DUSP18	22	31,048,037-31,063,877	AI, Loss	AI	AI, Loss	Loss, LOH	AI, Loss	AI, Loss	AI	Loss	AI	Loss
GAL3ST1	22	30,950,621-30,970,574	AI, Loss	AI	AI, Loss	Loss, LOH	AI, Loss	AI, Loss	AI	Loss	AI	Loss
PES1	22	30,972,611-31,003,000	AI, Loss	AI	AI, Loss	Loss, LOH	AI, Loss	AI, Loss	AI	Loss	AI	Loss
SEC14L6	22	30,918,774-30,942,669	AI, Loss	AI	AI, Loss	Loss, LOH	AI, Loss	AI, Loss	AI	Loss	AI	Loss
SLC35E4	22	31,031,792-31,065,003	AI, Loss	AI	AI, Loss	Loss, LOH	AI, Loss	AI, Loss	AI	Loss	AI	Loss

MORC2	22	31,321,116-31,364,273	AI, Loss	AI	AI, Loss	Loss, LOH	Loss	AI, Loss	AI	Loss	AI	Loss
LOC107985544	22	31,282,042-31,317,766	AI, Loss	AI	AI, Loss	Loss, LOH		AI, Loss	AI	Loss	AI	Loss
MORC2-AS1	22	31,318,294-31,322,640	AI, Loss	AI	AI, Loss	Loss, LOH		AI, Loss	AI	Loss	AI	Loss
ADSL	22	40,742,503-40,783,480	Loss	AI	AI, Loss	Loss, LOH	AI, Loss	AI, Loss	AI	Loss	AI	Loss
AK098727	22	38,381,941-38,384,339	Loss	AI	AI, Loss	Loss, LOH	AI, Loss	AI, Loss	AI	Loss	AI	Loss
BAIAP2L2	22	38,480,895-38,506,676	Loss	AI	AI, Loss	Loss, LOH	AI, Loss	AI, Loss	AI	Loss	AI	Loss
C22orf23	22	38,339,056-38,349,676	Loss	AI	AI, Loss	Loss, LOH	AI, Loss	AI, Loss	AI	Loss	AI	Loss
ENTHD1	22	40,139,048-40,289,794	Loss	AI	AI, Loss	Loss, LOH	AI, Loss	AI, Loss	AI	Loss	AI	Loss
GALR3	22	38,219,388-38,221,502	Loss	AI	AI, Loss	Loss, LOH	AI, Loss	AI, Loss	AI	Loss	AI	Loss
LOC100130899	22	40,428,335-40,432,581	Loss	AI	AI, Loss	Loss, LOH	AI, Loss	AI, Loss	AI	Loss	AI	Loss
MIR4534	22	38,384,800-38,384,860	Loss	AI	AI, Loss	Loss, LOH	AI, Loss	AI, Loss	AI	Loss	AI	Loss
MIR658	22	38,240,278-38,240,378	Loss	AI	AI, Loss	Loss, LOH	AI, Loss	AI, Loss	AI	Loss	AI	Loss
MIR6820	22	38,363,569-38,363,631	Loss	AI	AI, Loss	Loss, LOH	AI, Loss	AI, Loss	AI	Loss	AI	Loss
PICK1	22	38,453,261-38,471,708	Loss	AI	AI, Loss	Loss, LOH	AI, Loss	AI, Loss	AI	Loss	AI	Loss
PLA2G6	22	38,507,501-38,577,916	Loss	AI	AI, Loss	Loss, LOH	AI, Loss	AI, Loss	AI	Loss	AI	Loss
POLR2F	22	38,349,669-38,437,922	Loss	AI	AI, Loss	Loss, LOH	AI, Loss	AI, Loss	AI	Loss	AI	Loss
SGSM3	22	40,766,565-40,806,293	Loss	AI	AI, Loss	Loss, LOH	AI, Loss	AI, Loss	AI	Loss	AI	Loss

SLC16A8	22	38,474,143-38,479,170	Loss	AI	AI, Loss	Loss, LOH	AI, Loss	AI, Loss	AI	Loss	AI	Loss
SOX10	22	38,368,318-38,383,429	Loss	AI	AI, Loss	Loss, LOH	AI, Loss	AI, Loss	AI	Loss	AI	Loss
TNRC6B	22	40,440,820-40,731,812	Loss	AI	AI, Loss	Loss, LOH	AI, Loss	AI, Loss	AI	Loss	AI	Loss
TCN2	22	31,003,069-31,023,047	AI, Loss	AI, Loss	AI, Loss	Loss, LOH	AI, Loss	AI, Loss	AI	Loss	AI	Loss
ANKRD54	22	38,226,861-38,245,325	Loss	AI, Loss	AI, Loss	Loss, LOH	AI, Loss	AI, Loss	AI	Loss	AI	Loss
MICALL1	22	38,302,154-38,338,465	Loss	AI, Loss	AI, Loss	Loss, LOH	AI, Loss	AI, Loss	AI	Loss	AI	Loss
MKL1	22	40,806,291-41,032,690	Loss	AI, Loss	AI, Loss	Loss, LOH	AI, Loss	AI, Loss	AI	Loss	AI	Loss
MRTFA	22	40,806,284-41,032,723	Loss	AI, Loss	AI, Loss	Loss, LOH	AI, Loss	AI, Loss	AI	Loss	AI	Loss
EIF3L	22	38,245,378-38,284,789	Loss	Loss	AI, Loss	Loss, LOH	AI, Loss	AI, Loss	AI	Loss	AI	Loss
MIR659	22	38,243,684-38,243,781	Loss	Loss	AI, Loss	Loss, LOH	AI, Loss	AI, Loss	AI	Loss	AI	Loss
PIK3IP1	22	31,677,578-31,688,520	AI, Loss	AI	Loss	Loss, LOH	AI, Loss	AI, Loss	AI	Loss	AI	Loss
SELENOM	22	31,500,757-31,503,573	AI, Loss	AI	Loss	Loss, LOH	Loss	AI, Loss	AI	Loss	AI	Loss
SELM	22	31,500,762-31,503,551	AI, Loss	AI	Loss	Loss, LOH	Loss	AI, Loss	AI	Loss	AI	Loss
APOBEC3B-AS1	22	39,387,563-39,394,225	Loss	AI	Loss	Loss, LOH	AI, Loss	AI, Loss	AI	Loss	AI	Loss
ATF4	22	39,916,563-39,918,691	Loss	AI	Loss	Loss, LOH	AI, Loss	AI, Loss	AI	Loss	AI	Loss
BC038245	22	42,760,534-42,765,180	Loss	AI	Loss	Loss, LOH	AI, Loss	AI, Loss	AI	Loss	AI	Loss
DQ589665	22	39,917,590-39,917,619	Loss	AI	Loss	Loss, LOH	AI, Loss	AI, Loss	AI	Loss	AI	Loss

DQ601415	22	39,918,126-39,918,157	Loss	AI	Loss	Loss, LOH	AI, Loss	AI, Loss	AI	Loss	AI	Loss
LINC01315	22	42,760,405-42,765,214	Loss	AI	Loss	Loss, LOH	AI, Loss	AI, Loss	AI	Loss	AI	Loss
LOC100506472	22	39,828,164-39,833,133	Loss	AI	Loss	Loss, LOH	AI, Loss	AI, Loss	AI	Loss	AI	Loss
MGAT3	22	39,853,324-39,888,199	Loss	AI	Loss	Loss, LOH	AI, Loss	AI, Loss	AI	Loss	AI	Loss
MGAT3-AS1	22	39,871,811-39,872,827	Loss	AI	Loss	Loss, LOH	AI, Loss	AI, Loss	AI	Loss	AI	Loss
MIEF1	22	39,896,104-39,914,139	Loss	AI	Loss	Loss, LOH	AI, Loss	AI, Loss	AI	Loss	AI	Loss
NFAM1	22	42,776,413-42,828,401	Loss	AI	Loss	Loss, LOH	AI, Loss	AI, Loss	AI	Loss	AI	Loss
SMCR7L	22	39,896,104-39,914,137	Loss	AI	Loss	Loss, LOH	AI, Loss	AI, Loss	AI	Loss	AI	Loss
MEI1	22	42,095,517-42,195,459	Loss	AI	Loss	Loss, LOH	Loss	AI, Loss	AI	Loss	AI	Loss
MIR33A	22	42,296,947-42,297,016	Loss	AI	Loss	Loss, LOH	Loss	AI, Loss	AI	Loss	AI	Loss
MIR378I	22	42,319,225-42,319,301	Loss	AI	Loss	Loss, LOH	Loss	AI, Loss	AI	Loss	AI	Loss
NAGA	22	42,454,337-42,466,846	Loss	AI	Loss	Loss, LOH	Loss	AI, Loss	AI	Loss	AI	Loss
NDUFA6	22	42,481,529-42,486,776	Loss	AI	Loss	Loss, LOH	Loss	AI, Loss	AI	Loss	AI	Loss
NDUFA6-DT	22	42,486,936-42,521,354	Loss	AI	Loss	Loss, LOH	Loss	AI, Loss	AI	Loss	AI	Loss
SHISA8	22	42,305,539-42,311,099	Loss	AI	Loss	Loss, LOH	Loss	AI, Loss	AI	Loss	AI	Loss
SREBF2	22	42,229,082-42,303,312	Loss	AI	Loss	Loss, LOH	Loss	AI, Loss	AI	Loss	AI	Loss
TNFRSF13C	22	42,321,035-42,322,821	Loss	AI	Loss	Loss, LOH	Loss	AI, Loss	AI	Loss	AI	Loss

bK250D10.C22.8	22	42,120,358-42,354,544	Loss	AI, Loss	Loss	Loss, LOH	Loss	AI, Loss	AI	Loss	AI	Loss
CENPM	22	42,334,724-42,343,168	Loss	AI, Loss	Loss	Loss, LOH	Loss	AI, Loss	AI	Loss	AI	Loss
WBP2NL	22	42,394,728-42,454,460	Loss	AI, Loss	Loss	Loss, LOH	Loss	AI, Loss	AI	Loss	AI	Loss
3-Sep	22	42,365,441-42,394,225	Loss	Loss	Loss	Loss, LOH	Loss	AI, Loss	AI	Loss	AI	Loss
LINC00634	22	42,348,190-42,354,946	Loss	Loss	Loss	Loss, LOH	Loss	AI, Loss	AI	Loss	AI	Loss
FAM109B	22	42,470,254-42,475,442	Loss	AI		Loss, LOH	Loss	AI, Loss	AI	Loss	AI	Loss
PHETA2	22	42,470,254-42,475,442	Loss	AI		Loss, LOH	Loss	AI, Loss	AI	Loss	AI	Loss
SMDT1	22	42,475,694-42,480,288	Loss	AI		Loss, LOH	Loss	AI, Loss	AI	Loss	AI	Loss
BC127823	22	38,615,652-38,616,226	Loss	AI	AI, Loss	Loss, LOH	AI, Loss	AI, Loss	AI		AI	Loss
DKFZp686N1150	22	38,615,297-38,620,110	Loss	AI	AI, Loss	Loss, LOH	AI, Loss	AI, Loss	AI		AI	Loss
MAFF	22	38,597,938-38,612,517	Loss	AI	AI, Loss	Loss, LOH	AI, Loss	AI, Loss	AI		AI	Loss
SNORA92	22	38,620,485-38,620,727	Loss	AI	AI, Loss	Loss, LOH	AI, Loss	AI, Loss	AI		AI	Loss
TMEM184B	22	38,615,297-38,669,040	Loss	AI	AI, Loss	Loss, LOH	AI, Loss	AI, Loss	AI		AI	Loss
KIAA0845	22	29,885,264-29,887,277	AI, Loss	AI	AI, Loss	Loss, LOH	AI, Loss	AI, Loss			AI	Loss
NEFH	22	29,876,180-29,887,277	AI, Loss	AI	AI, Loss	Loss, LOH	AI, Loss	AI, Loss			AI	Loss
AX747817	22	31,497,861-31,500,743	AI, Loss	AI	Loss	Loss, LOH	Loss	AI, Loss	AI		AI	Loss
SMTN	22	31,477,281-31,500,610	AI, Loss	AI	Loss	Loss, LOH	Loss	AI, Loss	AI		AI	Loss

TUG1	22	31,365,196-31,375,380	AI, Loss	AI	Loss	Loss, LOH	Loss	AI, Loss	AI		AI	Loss
APOBEC3A	22	39,353,526-39,359,188	Loss	AI	Loss	Loss, LOH	AI, Loss	AI, Loss	AI		AI	Loss
APOBEC3A_B	22	39,353,526-39,388,783	Loss	AI	Loss	Loss, LOH	AI, Loss	AI, Loss	AI		AI	Loss
APOBEC3B	22	39,378,403-39,388,784	Loss	AI	Loss	Loss, LOH	AI, Loss	AI, Loss	AI		AI	Loss
POLDIP3	22	42,979,726-43,010,968	Loss	AI	Loss	Loss, LOH	AI, Loss	AI, Loss	AI		AI	Loss
RRP7A	22	42,904,340-42,915,829	Loss	AI	Loss	Loss, LOH	AI, Loss	AI, Loss	AI		AI	Loss
RRP7B	22	42,951,228-42,978,017	Loss	AI	Loss	Loss, LOH	AI, Loss	AI, Loss	AI		AI	Loss
RRP7BP	22	42,969,265-42,978,017	Loss	AI	Loss	Loss, LOH	AI, Loss	AI, Loss	AI		AI	Loss
SERHL	22	42,896,584-42,908,566	Loss	AI	Loss	Loss, LOH	AI, Loss	AI, Loss	AI		AI	Loss
SERHL2	22	42,949,867-42,970,388	Loss	AI	Loss	Loss, LOH	AI, Loss	AI, Loss	AI		AI	Loss
C22orf31	22	29,454,659-29,457,907	AI, Loss	AI	AI, Loss	Loss, LOH	AI, Loss	AI, Loss	AI	AI, Loss	AI, Loss	Loss
DMC1	22	38,914,953-38,966,201	Loss	AI, Loss	AI, Loss	Loss, LOH	AI, Loss	AI, Loss	AI	AI, Loss	AI, Loss	Loss
HORMAD2	22	30,476,057-30,573,064	AI, Loss	Loss	AI, Loss	Loss, LOH	AI, Loss	AI, Loss	AI	AI, Loss	AI, Loss	Loss
CCDC116	22	21,987,085-21,991,616	AI, Loss	AI	AI, Loss	Loss, LOH	AI, Loss	AI, Loss	AI	Loss	AI, Loss	Loss
OSBP2	22	31,089,768-31,303,811	AI, Loss	AI	AI, Loss	Loss, LOH	AI, Loss	AI, Loss	AI	Loss	AI, Loss	Loss
CACNA1I	22	39,966,757-40,085,740	Loss	AI	AI, Loss	Loss, LOH	AI, Loss	AI, Loss	AI	Loss	AI, Loss	Loss
KDELR3	22	38,864,066-38,879,452	Loss	AI	AI, Loss	Loss, LOH	AI, Loss	AI, Loss	AI	Loss	AI, Loss	Loss

RPS19BP1	22	39,925,097-39,928,860	Loss	AI	Loss	Loss, LOH	AI, Loss	AI, Loss	AI	Loss	AI, Loss	Loss
DDX17	22	38,879,442-38,903,665	Loss	AI	AI, Loss	Loss, LOH	AI, Loss	AI, Loss	AI	Loss	Loss	Loss
C22orf24	22	32,329,506-32,341,348	AI, Loss	AI	Loss	Loss, LOH	AI, Loss	AI, Loss	AI	AI	AI	
TMEM211	22	25,331,064-25,342,662	AI, Loss	AI	Loss	Loss, LOH	AI, Loss	AI, Loss	AI	AI	AI	
YWHAH	22	32,340,478-32,353,590	AI, Loss	AI	Loss	Loss, LOH	AI, Loss	AI, Loss	AI	AI	AI	
LINC02558	22	32,366,809-32,433,186	AI, Loss	AI		Loss, LOH	AI, Loss	AI, Loss	AI	AI	AI	
DNAL4	22	39,174,512-39,190,161	Loss	AI		Loss, LOH	AI, Loss	AI, Loss	AI	AI	AI	
CCDC117	22	29,168,661-29,185,289	AI, Loss	AI	AI, Loss	Loss, LOH	AI, Loss	AI, Loss	AI	AI, Loss	AI	
CHEK2	22	29,083,730-29,137,822	AI, Loss	AI	AI, Loss	Loss, LOH	AI, Loss	AI, Loss	AI	AI, Loss	AI	
HSCB	22	29,138,019-29,153,506	AI, Loss	AI	AI, Loss	Loss, LOH	AI, Loss	AI, Loss	AI	AI, Loss	AI	
XBP1	22	29,190,547-29,196,560	AI, Loss	AI	AI, Loss	Loss, LOH	AI, Loss	AI, Loss	AI	AI, Loss	AI	
ZNRF3-AS1	22	29,420,986-29,427,464	AI, Loss	AI	AI, Loss	Loss, LOH	AI, Loss	AI, Loss	AI	AI, Loss	AI	
CS330190	22	43,172,869-43,172,890	Loss	AI	AI, Loss	Loss, LOH	AI, Loss	AI, Loss	AI	AI, Loss	AI	
DQ595055	22	43,182,195-43,182,228	Loss	AI	AI, Loss	Loss, LOH	AI, Loss	AI, Loss	AI	AI, Loss	AI	
DNAJB7	22	41,255,553-41,258,130	Loss	AI	AI, Loss	Loss, LOH	Loss	AI, Loss	AI	AI, Loss	AI	
MIR4766	22	41,209,886-41,209,962	Loss	AI	AI, Loss	Loss, LOH	Loss	AI, Loss	AI	AI, Loss	AI	
SLC25A17	22	41,165,633-41,215,403	Loss	AI	AI, Loss	Loss, LOH	Loss	AI, Loss	AI	AI, Loss	AI	

ST13	22	41,220,538-41,253,012	Loss	AI	AI, Loss	Loss, LOH	Loss	AI, Loss	AI	AI, Loss	AI	
XPNPEP3	22	41,253,084-41,363,888	Loss	AI	AI, Loss	Loss, LOH	Loss	AI, Loss	AI	AI, Loss	AI	
NIPSNAP1	22	29,950,797-29,977,326	AI, Loss	AI	AI, Loss	Loss, LOH	AI, Loss	AI, Loss		AI, Loss	AI	
DEPDC5	22	32,149,936-32,303,020	AI, Loss	AI	Loss	Loss, LOH	AI, Loss	AI, Loss	AI	AI, Loss	AI	
EIF4ENIF1	22	31,835,344-31,885,923	AI, Loss	AI	Loss	Loss, LOH	AI, Loss	AI, Loss	AI	AI, Loss	AI	
A4GALT	22	43,088,117-43,117,307	Loss	AI	Loss	Loss, LOH	AI, Loss	AI, Loss	AI	AI, Loss	AI	
CSNK1E	22	38,686,696-38,794,527	Loss	AI	AI, Loss	Loss, LOH	AI, Loss	AI, Loss	AI	Loss	AI	
KCNJ4	22	38,822,332-38,851,205	Loss	AI	AI, Loss	Loss, LOH	AI, Loss	AI, Loss	AI	Loss	AI	
LOC400927	22	38,698,865-38,794,931	Loss	AI	AI, Loss	Loss, LOH	AI, Loss	AI, Loss	AI	Loss	AI	
MCHR1	22	41,075,181-41,078,818	Loss	AI	AI, Loss	Loss, LOH	AI, Loss	AI, Loss	AI	Loss	AI	
TPTEP2	22	38,740,669-38,794,931	Loss	AI	AI, Loss	Loss, LOH	AI, Loss	AI, Loss	AI	Loss	AI	
TPTEP2-CSNK1E	22	38,686,696-38,794,527	Loss	AI	AI, Loss	Loss, LOH	AI, Loss	AI, Loss	AI	Loss	AI	
EP300	22	41,488,613-41,576,081	Loss	AI	AI, Loss	Loss, LOH	Loss	AI, Loss	AI	Loss	AI	
MIR1281	22	41,488,516-41,488,570	Loss	AI	AI, Loss	Loss, LOH	Loss	AI, Loss	AI	Loss	AI	
MRTFA-AS1	22	40,917,803-40,922,711	Loss	Loss	AI, Loss	Loss, LOH	AI, Loss	AI, Loss	AI	Loss	AI	
DRG1	22	31,795,538-31,830,172	AI, Loss	AI	Loss	Loss, LOH	AI, Loss	AI, Loss	AI	Loss	AI	
LINC01521	22	31,742,744-31,747,141	AI, Loss	AI	Loss	Loss, LOH	AI, Loss	AI, Loss	AI	Loss	AI	

PATZ1	22	31,721,789-31,742,249	AI, Loss	AI	Loss	Loss, LOH	AI, Loss	AI, Loss	AI	Loss	AI	
PIK3IP1-AS1	22	31,731,334-31,734,007	AI, Loss	AI	Loss	Loss, LOH	AI, Loss	AI, Loss	AI	Loss	AI	
PRR14L	22	32,072,732-32,146,120	AI, Loss	AI	Loss	Loss, LOH	AI, Loss	AI, Loss	AI	Loss	AI	
CYB5R3	22	43,013,845-43,045,405	Loss	AI	Loss	Loss, LOH	AI, Loss	AI, Loss	AI	Loss	AI	
DL490307	22	43,019,890-43,019,952	Loss	AI	Loss	Loss, LOH	AI, Loss	AI, Loss	AI	Loss	AI	
ACO2	22	41,865,128-41,924,993	Loss	AI	Loss	Loss, LOH	Loss	AI, Loss	AI	Loss	AI	
C22orf46	22	42,086,546-42,094,140	Loss	AI	Loss	Loss, LOH	Loss	AI, Loss	AI	Loss	AI	
CCDC134	22	42,196,625-42,222,303	Loss	AI	Loss	Loss, LOH	Loss	AI, Loss	AI	Loss	AI	
CHADL	22	41,625,513-41,636,935	Loss	AI	Loss	Loss, LOH	Loss	AI, Loss	AI	Loss	AI	
CSDC2	22	41,957,013-41,972,670	Loss	AI	Loss	Loss, LOH	Loss	AI, Loss	AI	Loss	AI	
DESI1	22	41,994,031-42,017,061	Loss	AI	Loss	Loss, LOH	Loss	AI, Loss	AI	Loss	AI	
DQ596940	22	42,222,470-42,222,500	Loss	AI	Loss	Loss, LOH	Loss	AI, Loss	AI	Loss	AI	
FLJ23584	22	42,090,412-42,094,139	Loss	AI	Loss	Loss, LOH	Loss	AI, Loss	AI	Loss	AI	
LOC105373044	22	41,947,712-41,956,887	Loss	AI	Loss	Loss, LOH	Loss	AI, Loss	AI	Loss	AI	
MIR6889	22	41,648,995-41,649,054	Loss	AI	Loss	Loss, LOH	Loss	AI, Loss	AI	Loss	AI	
NHP2L1	22	42,069,936-42,084,913	Loss	AI	Loss	Loss, LOH	Loss	AI, Loss	AI	Loss	AI	
PHF5A	22	41,855,720-41,864,708	Loss	AI	Loss	Loss, LOH	Loss	AI, Loss	AI	Loss	AI	

PMM1	22	41,972,889-41,985,871	Loss	AI	Loss	Loss, LOH	Loss	AI, Loss	AI	Loss	AI	
POLR3H	22	41,921,805-41,940,610	Loss	AI	Loss	Loss, LOH	Loss	AI, Loss	AI	Loss	AI	
RANGAP1	22	41,640,780-41,698,217	Loss	AI	Loss	Loss, LOH	Loss	AI, Loss	AI	Loss	AI	
SNU13	22	42,069,936-42,084,913	Loss	AI	Loss	Loss, LOH	Loss	AI, Loss	AI	Loss	AI	
SREBF2-AS1	22	42,227,218-42,230,669	Loss	AI	Loss	Loss, LOH	Loss	AI, Loss	AI	Loss	AI	
TEF	22	41,763,336-41,795,332	Loss	AI	Loss	Loss, LOH	Loss	AI, Loss	AI	Loss	AI	
TOB2	22	41,829,491-41,843,027	Loss	AI	Loss	Loss, LOH	Loss	AI, Loss	AI	Loss	AI	
XRCC6	22	42,017,166-42,060,052	Loss	AI	Loss	Loss, LOH	Loss	AI, Loss	AI	Loss	AI	
ZC3H7B	22	41,697,506-41,756,151	Loss	AI	Loss	Loss, LOH	Loss	AI, Loss	AI	Loss	AI	
L3MBTL2	22	41,601,312-41,627,275	Loss	AI		Loss, LOH	Loss	AI, Loss	AI	Loss	AI	
MIR130B	22	22,007,592-22,007,674	AI, Loss	AI	AI, Loss	Loss, LOH	AI, Loss	AI, Loss	AI		AI	
MIR301B	22	22,007,269-22,007,347	AI, Loss	AI	AI, Loss	Loss, LOH	AI, Loss	AI, Loss	AI		AI	
PPIL2	22	22,020,272-22,052,202	AI, Loss	AI	AI, Loss	Loss, LOH	AI, Loss	AI, Loss	AI		AI	
SDF2L1	22	21,996,541-21,998,588	AI, Loss	AI	AI, Loss	Loss, LOH	AI, Loss	AI, Loss	AI		AI	
YPEL1	22	22,051,825-22,090,123	AI, Loss	AI	AI, Loss	Loss, LOH	AI, Loss	AI, Loss	AI		AI	
RBX1	22	41,347,350-41,369,019	Loss	AI	AI, Loss	Loss, LOH	Loss	AI, Loss	AI		AI	
SNORD140	22	41,469,605-41,469,725	Loss	AI	AI, Loss	Loss, LOH	Loss	AI, Loss	AI		AI	

HV452684	22	41,077,559-41,077,932	Loss	AI	AI, Loss	Loss, LOH		AI, Loss	AI		AI	
AK074476	22	31,831,222-31,834,552	AI, Loss	AI	Loss	Loss, LOH	AI, Loss	AI, Loss	AI		AI	
MIR7109	22	32,017,452-32,017,517	AI, Loss	AI	Loss	Loss, LOH	AI, Loss	AI, Loss	AI		AI	
ATP5L2	22	43,035,808-43,036,607	Loss	AI	Loss	Loss, LOH	AI, Loss	AI, Loss	AI		AI	
ATP5MGL	22	43,035,808-43,036,607	Loss	AI	Loss	Loss, LOH	AI, Loss	AI, Loss	AI		AI	
RNU12	22	43,011,250-43,011,399	Loss	AI	Loss	Loss, LOH	AI, Loss	AI, Loss	AI		AI	
AK057177	22	41,605,125-41,613,360	Loss	AI		Loss, LOH	Loss	AI, Loss	AI		AI	
BC040700	22	41,581,218-41,593,505	Loss	AI		Loss, LOH	Loss	AI, Loss	AI		AI	
EP300-AS1	22	41,581,218-41,593,505	Loss	AI		Loss, LOH	Loss	AI, Loss	AI		AI	
LOC100506544	22	41,605,125-41,613,631	Loss	AI		Loss, LOH	Loss	AI, Loss	AI		AI	
ZNRF3	22	29,279,754-29,453,476	AI, Loss	AI	AI, Loss	Loss, LOH	AI, Loss	AI, Loss	AI	AI, Loss	AI, Loss	
PISD	22	32,014,476-32,058,550	AI, Loss	AI	Loss	Loss, LOH	AI, Loss	AI, Loss	AI	AI, Loss	AI, Loss	
SFI1	22	31,892,124-32,014,537	AI, Loss	AI	Loss	Loss, LOH	AI, Loss	AI, Loss	AI	AI, Loss	AI, Loss	
UBE2L3	22	21,903,735-21,978,323	AI, Loss	AI	AI, Loss	Loss, LOH	AI, Loss	AI, Loss	AI	Loss	AI, Loss	
MIR3200	22	31,127,543-31,127,628	AI, Loss	AI	AI, Loss	Loss, LOH	AI, Loss	AI, Loss	AI	Loss	Loss	
YDJC	22	21,982,377-21,984,340	AI, Loss	AI	AI, Loss	Loss, LOH	AI, Loss	AI, Loss	AI	Loss	Loss	
GCAT	22	38,203,911-38,213,183	Loss	AI	AI, Loss	Loss, LOH	AI, Loss	AI, Loss	AI	Loss		

H1F0	22	38,201,113-38,203,443	Loss	AI	AI, Loss	Loss, LOH	AI, Loss	AI, Loss	AI	Loss		
5-Sep	22	19,701,986-19,710,845	AI, Loss		AI, Loss	AI, Gain	AI, Loss	Gain	AI		AI	AI, Loss
GP1BB	22	19,711,065-19,712,297	AI, Loss		AI, Loss	AI, Gain	AI, Loss	Gain	AI		AI	AI, Loss
SEPT5-GP1BB	22	19,701,986-19,712,297	AI, Loss		AI, Loss	AI, Gain	AI, Loss	Gain	AI		AI	AI, Loss
HSFY1P1	22	17,308,363-17,310,225	AI, Loss		Loss	AI, Gain	AI, Loss	Gain	AI		AI	Loss
IGKV1-12	22	17,385,314-17,385,395	AI, Loss		Loss	AI, Gain	AI, Loss	Gain	AI		AI	Loss
XKR3	22	17,264,305-17,302,589	AI, Loss		Loss	AI, Gain	AI, Loss	Gain	AI		AI, Loss	Loss
KCTD17	22	37,447,775-37,459,430	AI, Loss	AI	AI, Loss	Loss, LOH	AI, Loss	Loss	AI	AI	AI	AI, Loss
KIAA0930	22	45,588,122-45,636,650	AI, Loss	AI	AI, Loss	Loss, LOH	AI, Loss	Loss	AI	AI	AI	AI, Loss
MIR1249	22	45,596,834-45,596,900	AI, Loss	AI	AI, Loss	Loss, LOH	AI, Loss	Loss	AI	AI	AI	AI, Loss
TMPRSS6	22	37,461,475-37,505,603	AI, Loss	AI	AI, Loss	Loss, LOH	AI, Loss	Loss	AI	AI	AI	AI, Loss
UPK3A	22	45,680,867-45,691,755	AI, Loss	AI	AI, Loss	Loss, LOH	AI, Loss	Loss	AI	AI	AI	AI, Loss
AK123632	22	37,099,962-37,117,494	AI, Loss	AI	Loss	Loss, LOH	AI, Loss	Loss	AI	AI	AI	AI, Loss
BX537634	22	37,154,254-37,158,116	AI, Loss	AI	Loss	Loss, LOH	AI, Loss	Loss	AI	AI	AI	AI, Loss
ELFN2	22	37,736,684-37,823,505	AI, Loss	AI	Loss	Loss, LOH	AI, Loss	Loss	AI	AI	AI	AI, Loss
IFT27	22	37,154,245-37,172,177	AI, Loss	AI	Loss	Loss, LOH	AI, Loss	Loss	AI	AI	AI	AI, Loss
IL2RB	22	37,521,879-37,571,158	AI, Loss	AI	Loss	Loss, LOH	AI, Loss	Loss	AI	AI	AI	AI, Loss

LOC105373021	22	37,099,920-37,163,133	AI, Loss	AI	Loss	Loss, LOH	AI, Loss	Loss	AI	AI	AI	AI, Loss
PVALB	22	37,196,744-37,215,517	AI, Loss	AI	Loss	Loss, LOH	AI, Loss	Loss	AI	AI	AI	AI, Loss
AK097791	22	38,037,838-38,054,384	Loss	AI	Loss	Loss, LOH	AI, Loss	Loss	AI	AI	AI	AI, Loss
DM074112	22	38,075,645-38,075,666	Loss	AI	Loss	Loss, LOH	AI, Loss	Loss	AI	AI	AI	AI, Loss
GGA1	22	38,004,480-38,029,571	Loss	AI	Loss	Loss, LOH	AI, Loss	Loss	AI	AI	AI	AI, Loss
LGALS1	22	38,071,612-38,075,809	Loss	AI	Loss	Loss, LOH	AI, Loss	Loss	AI	AI	AI	AI, Loss
LOC101927051	22	38,037,838-38,054,384	Loss	AI	Loss	Loss, LOH	AI, Loss	Loss	AI	AI	AI	AI, Loss
PDXP	22	38,054,736-38,062,939	Loss	AI	Loss	Loss, LOH	AI, Loss	Loss	AI	AI	AI	AI, Loss
SH3BP1	22	38,030,660-38,062,939	Loss	AI	Loss	Loss, LOH	AI, Loss	Loss	AI	AI	AI	AI, Loss
AK097787	22	37,748,230-37,750,879	AI, Loss	AI		Loss, LOH	AI, Loss	Loss	AI	AI	AI	AI, Loss
DQ570280	22	37,757,138-37,757,172	AI, Loss	AI		Loss, LOH	AI, Loss	Loss	AI	AI	AI	AI, Loss
DQ571088	22	37,760,168-37,760,236	AI, Loss	AI		Loss, LOH	AI, Loss	Loss	AI	AI	AI	AI, Loss
DQ571864	22	37,754,335-37,754,480	AI, Loss	AI		Loss, LOH	AI, Loss	Loss	AI	AI	AI	AI, Loss
DQ572877	22	37,756,430-37,756,466	AI, Loss	AI		Loss, LOH	AI, Loss	Loss	AI	AI	AI	AI, Loss
DQ572884	22	37,757,194-37,757,225	AI, Loss	AI		Loss, LOH	AI, Loss	Loss	AI	AI	AI	AI, Loss
DQ573975	22	37,755,236-37,755,266	AI, Loss	AI		Loss, LOH	AI, Loss	Loss	AI	AI	AI	AI, Loss
DQ574091	22	37,760,768-37,760,798	AI, Loss	AI		Loss, LOH	AI, Loss	Loss	AI	AI	AI	AI, Loss

DQ574365	22	37,756,945-37,756,982	AI, Loss	AI		Loss, LOH	AI, Loss	Loss	AI	AI	AI	AI, Loss
DQ574556	22	37,759,407-37,759,438	AI, Loss	AI		Loss, LOH	AI, Loss	Loss	AI	AI	AI	AI, Loss
DQ574964	22	37,756,836-37,756,881	AI, Loss	AI		Loss, LOH	AI, Loss	Loss	AI	AI	AI	AI, Loss
DQ575042	22	37,759,935-37,759,985	AI, Loss	AI		Loss, LOH	AI, Loss	Loss	AI	AI	AI	AI, Loss
DQ576223	22	37,761,799-37,761,854	AI, Loss	AI		Loss, LOH	AI, Loss	Loss	AI	AI	AI	AI, Loss
DQ576685	22	37,759,487-37,759,517	AI, Loss	AI		Loss, LOH	AI, Loss	Loss	AI	AI	AI	AI, Loss
DQ577118	22	37,753,888-37,753,926	AI, Loss	AI		Loss, LOH	AI, Loss	Loss	AI	AI	AI	AI, Loss
DQ580960	22	37,756,486-37,756,548	AI, Loss	AI		Loss, LOH	AI, Loss	Loss	AI	AI	AI	AI, Loss
DQ581304	22	37,760,018-37,760,101	AI, Loss	AI		Loss, LOH	AI, Loss	Loss	AI	AI	AI	AI, Loss
DQ581393	22	37,751,669-37,751,701	AI, Loss	AI		Loss, LOH	AI, Loss	Loss	AI	AI	AI	AI, Loss
DQ581625	22	37,754,870-37,754,908	AI, Loss	AI		Loss, LOH	AI, Loss	Loss	AI	AI	AI	AI, Loss
DQ584235	22	37,755,123-37,755,155	AI, Loss	AI		Loss, LOH	AI, Loss	Loss	AI	AI	AI	AI, Loss
DQ584633	22	37,756,901-37,756,938	AI, Loss	AI		Loss, LOH	AI, Loss	Loss	AI	AI	AI	AI, Loss
DQ584694	22	37,755,718-37,755,757	AI, Loss	AI		Loss, LOH	AI, Loss	Loss	AI	AI	AI	AI, Loss
DQ584932	22	37,761,280-37,761,317	AI, Loss	AI		Loss, LOH	AI, Loss	Loss	AI	AI	AI	AI, Loss
DQ584963	22	37,760,568-37,760,601	AI, Loss	AI		Loss, LOH	AI, Loss	Loss	AI	AI	AI	AI, Loss
DQ587342	22	37,751,349-37,751,380	AI, Loss	AI		Loss, LOH	AI, Loss	Loss	AI	AI	AI	AI, Loss

DQ587429	22	37,760,686-37,760,760	AI, Loss	AI		Loss, LOH	AI, Loss	Loss	AI	AI	AI	AI, Loss
DQ588058	22	37,757,457-37,757,487	AI, Loss	AI		Loss, LOH	AI, Loss	Loss	AI	AI	AI	AI, Loss
DQ589322	22	37,761,389-37,761,439	AI, Loss	AI		Loss, LOH	AI, Loss	Loss	AI	AI	AI	AI, Loss
DQ590895	22	37,758,472-37,758,522	AI, Loss	AI		Loss, LOH	AI, Loss	Loss	AI	AI	AI	AI, Loss
DQ592963	22	37,760,254-37,760,285	AI, Loss	AI		Loss, LOH	AI, Loss	Loss	AI	AI	AI	AI, Loss
DQ593233	22	37,755,506-37,755,566	AI, Loss	AI		Loss, LOH	AI, Loss	Loss	AI	AI	AI	AI, Loss
DQ594524	22	37,758,364-37,758,404	AI, Loss	AI		Loss, LOH	AI, Loss	Loss	AI	AI	AI	AI, Loss
DQ595101	22	37,758,976-37,759,044	AI, Loss	AI		Loss, LOH	AI, Loss	Loss	AI	AI	AI	AI, Loss
DQ595731	22	37,754,523-37,754,581	AI, Loss	AI		Loss, LOH	AI, Loss	Loss	AI	AI	AI	AI, Loss
DQ596669	22	37,757,558-37,757,596	AI, Loss	AI		Loss, LOH	AI, Loss	Loss	AI	AI	AI	AI, Loss
DQ597182	22	37,760,893-37,760,926	AI, Loss	AI		Loss, LOH	AI, Loss	Loss	AI	AI	AI	AI, Loss
DQ597571	22	37,756,606-37,756,659	AI, Loss	AI		Loss, LOH	AI, Loss	Loss	AI	AI	AI	AI, Loss
DQ597697	22	37,761,714-37,761,755	AI, Loss	AI		Loss, LOH	AI, Loss	Loss	AI	AI	AI	AI, Loss
DQ599403	22	37,756,691-37,756,734	AI, Loss	AI		Loss, LOH	AI, Loss	Loss	AI	AI	AI	AI, Loss
DQ601805	22	37,761,451-37,761,479	AI, Loss	AI		Loss, LOH	AI, Loss	Loss	AI	AI	AI	AI, Loss
EIF3D	22	36,906,896-36,925,277	AI, Loss	AI		Loss, LOH	AI, Loss	Loss	AI	AI	AI	AI, Loss
LOC100506271	22	37,748,230-37,750,879	AI, Loss	AI		Loss, LOH	AI, Loss	Loss	AI	AI	AI	AI, Loss

LGALS2	22	37,966,252-37,976,024	Loss	AI		Loss, LOH	AI, Loss	Loss	AI	AI	AI	AI, Loss
DQ574393	22	37,763,100-37,763,154		AI		Loss, LOH	AI, Loss	Loss	AI	AI	AI	AI, Loss
DQ576178	22	37,762,487-37,762,529		AI		Loss, LOH	AI, Loss	Loss	AI	AI	AI	AI, Loss
DQ576602	22	37,762,153-37,762,183		AI		Loss, LOH	AI, Loss	Loss	AI	AI	AI	AI, Loss
DQ577109	22	37,762,757-37,762,788		AI		Loss, LOH	AI, Loss	Loss	AI	AI	AI	AI, Loss
DQ588809	22	37,762,387-37,762,419		AI		Loss, LOH	AI, Loss	Loss	AI	AI	AI	AI, Loss
DQ601762	22	37,763,031-37,763,060		AI		Loss, LOH	AI, Loss	Loss	AI	AI	AI	AI, Loss
DQ579677	22	37,763,545-37,763,576		AI		Loss, LOH		Loss	AI	AI	AI	AI, Loss
DQ593994	22	37,763,935-37,764,007		AI		Loss, LOH		Loss	AI	AI	AI	AI, Loss
DQ594390	22	37,763,675-37,763,723		AI		Loss, LOH		Loss	AI	AI	AI	AI, Loss
DQ594725	22	37,763,329-37,763,375		AI		Loss, LOH		Loss	AI	AI	AI	AI, Loss
LOC100506714	22	45,529,638-45,559,662	AI, Loss	AI	AI, Loss	Loss, LOH	AI, Loss	Loss	AI	AI, Loss	AI	AI, Loss
LOC105373064	22	45,572,122-45,587,493	AI, Loss	AI	AI, Loss	Loss, LOH	AI, Loss	Loss	AI	AI, Loss	AI	AI, Loss
C1QTNF6	22	37,576,205-37,595,463	AI, Loss	AI	Loss	Loss, LOH	AI, Loss	Loss	AI	AI, Loss	AI	AI, Loss
CYTH4	22	37,678,423-37,711,389	AI, Loss	AI	Loss	Loss, LOH	AI, Loss	Loss	AI	AI, Loss	AI	AI, Loss
NCF4-AS1	22	37,243,414-37,266,485	AI, Loss	AI	Loss	Loss, LOH	AI, Loss	Loss	AI	AI, Loss	AI	AI, Loss
PLXNB2	22	50,713,407-50,746,001	AI, Loss	AI	Loss	Loss, LOH	AI, Loss	Loss	AI	AI, Loss	AI	AI, Loss

RAC2	22	37,621,300-37,640,339	AI, Loss	AI	Loss	Loss, LOH	AI, Loss	Loss	AI	AI, Loss	AI	AI, Loss
SSTR3	22	37,600,276-37,608,416	AI, Loss	AI	Loss	Loss, LOH	AI, Loss	Loss	AI	AI, Loss	AI	AI, Loss
MFNG	22	37,865,100-37,882,478	Loss	AI	Loss	Loss, LOH	AI, Loss	Loss	AI	AI, Loss	AI	AI, Loss
NOL12	22	38,082,343-38,089,485	Loss	AI	Loss	Loss, LOH	AI, Loss	Loss	AI	AI, Loss	AI	AI, Loss
CACNG2	22	36,956,915-37,098,903	AI, Loss	AI, Loss	Loss	Loss, LOH	AI, Loss	Loss	AI	AI, Loss	AI	AI, Loss
CDC42EP1	22	37,956,470-37,965,410	Loss	AI		Loss, LOH	AI, Loss	Loss	AI	AI, Loss	AI	AI, Loss
CSF2RB	22	37,309,674-37,336,479	AI, Loss	AI	AI, Loss	Loss, LOH	AI, Loss	Loss	AI	Loss	AI	AI, Loss
LL22NC01-81G9.3	22	37,361,288-37,364,213	AI, Loss	AI	AI, Loss	Loss, LOH	AI, Loss	Loss	AI	Loss	AI	AI, Loss
LOC100506241	22	37,361,288-37,364,213	AI, Loss	AI	AI, Loss	Loss, LOH	AI, Loss	Loss	AI	Loss	AI	AI, Loss
MPST	22	37,415,682-37,425,863	AI, Loss	AI	AI, Loss	Loss, LOH	AI, Loss	Loss	AI	Loss	AI	AI, Loss
NUP50	22	45,559,725-45,583,890	AI, Loss	AI	AI, Loss	Loss, LOH	AI, Loss	Loss	AI	Loss	AI	AI, Loss
TEX33	22	37,387,159-37,403,877	AI, Loss	AI, Loss	AI, Loss	Loss, LOH	AI, Loss	Loss	AI	Loss	AI	AI, Loss
TST	22	37,406,899-37,416,224	AI, Loss	AI, Loss	AI, Loss	Loss, LOH	AI, Loss	Loss	AI	Loss	AI	AI, Loss
MAPK11	22	50,702,141-50,709,340	AI, Loss	AI	Loss	Loss, LOH	AI, Loss	Loss	AI	Loss	AI	AI, Loss
MAPK12	22	50,688,491-50,700,239	AI, Loss	AI	Loss	Loss, LOH	AI, Loss	Loss	AI	Loss	AI	AI, Loss
CARD10	22	37,875,382-37,915,378	Loss	AI	Loss	Loss, LOH	AI, Loss	Loss	AI	Loss	AI	AI, Loss
HDAC10	22	50,683,612-50,689,834	AI, Loss	AI, Loss	Loss	Loss, LOH	AI, Loss	Loss	AI	Loss	AI	AI, Loss

TUBGCP6	22	50,656,117-50,683,400	AI, Loss	AI, Loss	Loss	Loss, LOH	AI, Loss	Loss	AI	Loss	AI	AI, Loss
NCF4	22	37,257,029-37,274,059	AI, Loss	AI		Loss, LOH	AI, Loss	Loss	AI	Loss	AI	AI, Loss
DENND6B	22	50,750,391-50,765,489	Loss	AI	Loss	Loss, LOH	AI, Loss	Loss	AI	AI	AI	Loss
PPP6R2	22	50,781,745-50,883,518	Loss	AI	Loss	Loss, LOH	AI, Loss	Loss	AI	AI	AI	Loss
AB372727	22	50,862,771-50,862,865		AI		Loss, LOH	AI, Loss	Loss	AI	AI	AI	Loss
ACR	22	51,176,651-51,183,727	Loss	AI		Loss, LOH	AI, Loss	Loss		AI	AI	Loss
BC050343	22	51,174,256-51,176,567	Loss	AI		Loss, LOH	AI, Loss	Loss		AI	AI	Loss
LOC105373100	22	51,174,256-51,176,597	Loss	AI		Loss, LOH	AI, Loss	Loss		AI	AI	Loss
RABL2B	22	51,205,919-51,222,110	Loss	AI		Loss, LOH	AI, Loss	Loss		AI	AI	Loss
RPL23AP82	22	51,195,513-51,238,065	Loss	AI		Loss, LOH	AI, Loss	Loss		AI	AI	Loss
SBF1	22	50,883,430-50,913,500	Loss	AI	Loss	Loss, LOH	AI, Loss	Loss	AI	AI, Loss	AI	Loss
ARSA	22	51,061,181-51,066,601	Loss	AI		Loss, LOH	AI, Loss	Loss	AI	AI, Loss	AI	Loss
MAPK8IP2	22	51,039,113-51,052,409	Loss	AI		Loss, LOH	AI, Loss	Loss	AI	AI, Loss	AI	Loss
SHANK3	22	51,113,069-51,171,640	Loss	AI		Loss, LOH	AI, Loss	Loss		AI, Loss	AI	Loss
KLHDC7B	22	50,984,631-50,989,452	Loss	AI	Loss	Loss, LOH	AI, Loss	Loss	AI	Loss	AI	Loss
LMF2	22	50,941,375-50,946,135	Loss	AI	Loss	Loss, LOH	AI, Loss	Loss	AI	Loss	AI	Loss
NCAPH2	22	50,946,644-50,963,209	Loss	AI	Loss	Loss, LOH	AI, Loss	Loss	AI	Loss	AI	Loss

ODF3B	22	50,968,837-50,971,008	Loss	AI	Loss	Loss, LOH	AI, Loss	Loss	AI	Loss	AI	Loss
SYCE3	22	50,989,540-51,001,381	Loss	AI	Loss	Loss, LOH	AI, Loss	Loss	AI	Loss	AI	Loss
TYMP	22	50,964,180-50,968,514	Loss	AI	Loss	Loss, LOH	AI, Loss	Loss	AI	Loss	AI	Loss
MIOX	22	50,925,212-50,928,750	Loss	AI, Loss	Loss	Loss, LOH	AI, Loss	Loss	AI	Loss	AI	Loss
BC048192	22	51,009,427-51,010,882	Loss	AI	Loss	Loss, LOH	AI, Loss	Loss	AI		AI	Loss
CHKB-CPT1B	22	51,007,289-51,021,428	Loss	AI	Loss	Loss, LOH	AI, Loss	Loss	AI		AI	Loss
CPT1B	22	51,007,289-51,017,096	Loss	AI	Loss	Loss, LOH	AI, Loss	Loss	AI		AI	Loss
SCO2	22	50,961,996-50,964,868	Loss	AI	Loss	Loss, LOH	AI, Loss	Loss	AI		AI	Loss
CHKB	22	51,017,386-51,021,428	Loss	AI		Loss, LOH	AI, Loss	Loss	AI		AI	Loss
CHKB-AS1	22	51,021,454-51,022,355	Loss	AI		Loss, LOH	AI, Loss	Loss	AI		AI	Loss
CHKB-DT	22	51,021,454-51,033,710	Loss	AI		Loss, LOH	AI, Loss	Loss	AI		AI	Loss
ADM2	22	50,919,984-50,924,866	Loss	Loss	Loss	Loss, LOH	AI, Loss	Loss	AI	Loss	AI, Loss	Loss
ARVCF	22	19,957,401-20,004,309	AI, Loss		AI, Loss	AI, Gain	AI, Loss		AI	AI	AI	AI, Loss
BX648073	22	19,429,055-19,431,418	AI, Loss		AI, Loss	AI, Gain	AI, Loss		AI	AI	AI	AI, Loss
C22orf29	22	19,833,660-19,842,462	AI, Loss		AI, Loss	AI, Gain	AI, Loss		AI	AI	AI	AI, Loss
C22orf39	22	19,428,409-19,435,755	AI, Loss		AI, Loss	AI, Gain	AI, Loss		AI	AI	AI	AI, Loss
CDC45	22	19,467,348-19,508,135	AI, Loss		AI, Loss	AI, Gain	AI, Loss		AI	AI	AI	AI, Loss

CLDN5	22	19,510,546-19,512,860	AI, Loss		AI, Loss	AI, Gain	AI, Loss		AI	AI	AI	AI, Loss
LINC00895	22	19,553,652-19,554,362	AI, Loss		AI, Loss	AI, Gain	AI, Loss		AI	AI	AI	AI, Loss
RTL10	22	19,833,660-19,842,371	AI, Loss		AI, Loss	AI, Gain	AI, Loss		AI	AI	AI	AI, Loss
TBX1	22	19,744,225-19,771,112	AI, Loss		AI, Loss	AI, Gain	AI, Loss		AI	AI	AI	AI, Loss
U84523	22	19,459,917-19,460,033	AI, Loss		AI, Loss	AI, Gain	AI, Loss		AI	AI	AI	AI, Loss
UFD1	22	19,437,463-19,466,738	AI, Loss		AI, Loss	AI, Gain	AI, Loss		AI	AI	AI	AI, Loss
UFD1L	22	19,437,463-19,466,738	AI, Loss		AI, Loss	AI, Gain	AI, Loss		AI	AI	AI	AI, Loss
DQ574263	22	21,355,229-21,355,259	AI, Loss		Loss	AI	AI, Loss		AI	AI	AI	AI, Loss
LZTR1	22	21,333,750-21,353,326	AI, Loss		Loss	AI	AI, Loss		AI	AI	AI	AI, Loss
P2RX6	22	21,369,441-21,382,302	AI, Loss		Loss	AI	AI, Loss		AI	AI	AI	AI, Loss
SLC7A4	22	21,383,006-21,386,847	AI, Loss		Loss	AI	AI, Loss		AI	AI	AI	AI, Loss
THAP7	22	21,354,060-21,356,404	AI, Loss		Loss	AI	AI, Loss		AI	AI	AI	AI, Loss
THAP7-AS1	22	21,356,210-21,364,663	AI, Loss		Loss	AI	AI, Loss		AI	AI	AI	AI, Loss
TUBA3FP	22	21,362,495-21,368,576	AI, Loss		Loss	AI	AI, Loss		AI	AI	AI	AI, Loss
BCRP2	22	21,457,304-21,476,575	AI, Loss		Loss	Gain	AI, Loss		AI	AI	AI	AI, Loss
HIC2	22	21,771,692-21,805,750	AI, Loss	AI	Loss		AI, Loss		AI	AI	AI	AI, Loss
7SK	22	21,743,479-21,743,780	AI, Loss		Loss		AI, Loss		AI	AI	AI	AI, Loss

AK128837	22	21,538,002-21,546,445	AI, Loss		Loss		AI, Loss		AI	AI	AI	AI, Loss
AX748067	22	21,642,518-21,646,214	AI, Loss		Loss		AI, Loss		AI	AI	AI	AI, Loss
BC039313	22	21,538,002-21,546,445	AI, Loss		Loss		AI, Loss		AI	AI	AI	AI, Loss
BCR	22	21,642,517-21,644,017	AI, Loss		Loss		AI, Loss		AI	AI	AI	AI, Loss
DQ570150	22	21,481,136-21,481,174	AI, Loss		Loss		AI, Loss		AI	AI	AI	AI, Loss
FAM230B	22	21,521,191-21,546,445	AI, Loss		Loss		AI, Loss		AI	AI	AI	AI, Loss
FAM230H	22	21,655,548-21,680,925	AI, Loss		Loss		AI, Loss		AI	AI	AI	AI, Loss
GGT2	22	21,562,261-21,632,151	AI, Loss		Loss		AI, Loss		AI	AI	AI	AI, Loss
LOC102724728	22	21,480,427-21,482,309	AI, Loss		Loss		AI, Loss		AI	AI	AI	AI, Loss
POM121L7	22	21,477,049-21,482,095	AI, Loss		Loss		AI, Loss		AI	AI	AI	AI, Loss
POM121L7	22	21,637,768-21,638,950	AI, Loss		Loss		AI, Loss		AI	AI	AI	AI, Loss
POM121L8P	22	21,636,713-21,652,015	AI, Loss		Loss		AI, Loss		AI	AI	AI	AI, Loss
RIMBP3C	22	21,737,662-21,743,455	AI, Loss		Loss		AI, Loss		AI	AI	AI	AI, Loss
LOC400891	22	21,400,248-21,418,457	AI, Loss			AI	AI, Loss		AI	AI	AI	AI, Loss
LRRC74B	22	21,400,248-21,418,457	AI, Loss			AI	AI, Loss		AI	AI	AI	AI, Loss
Mir_649	22	21,388,464-21,388,561	AI, Loss			AI	AI, Loss		AI	AI	AI	AI, Loss
MIR649	22	21,388,464-21,388,561	AI, Loss			AI	AI, Loss		AI	AI	AI	AI, Loss

P2RX6P	22	21,396,680-21,398,538	AI, Loss			AI	AI, Loss		AI	AI	AI	AI, Loss
LOC101927551	22	45,396,445-45,399,500	AI, Loss	AI		Loss, LOH			AI	AI	AI	AI, Loss
BC033336	22	37,767,723-37,768,896		AI		Loss, LOH			AI	AI	AI	AI, Loss
BC035867	22	20,970,516-21,011,201	AI, Loss		AI, Loss	AI, Gain	AI, Loss		AI		AI	AI, Loss
CCDC188	22	20,135,949-20,138,578	AI, Loss		AI, Loss	AI, Gain	AI, Loss		AI		AI	AI, Loss
DGCR14	22	19,117,791-19,132,190	AI, Loss		AI, Loss	AI, Gain	AI, Loss		AI		AI	AI, Loss
ESS2	22	19,117,791-19,132,190	AI, Loss		AI, Loss	AI, Gain	AI, Loss		AI		AI	AI, Loss
GSC2	22	19,136,503-19,137,796	AI, Loss		AI, Loss	AI, Gain	AI, Loss		AI		AI	AI, Loss
LINC00896	22	20,193,854-20,196,060	AI, Loss		AI, Loss	AI, Gain	AI, Loss		AI		AI	AI, Loss
LINC01311	22	19,159,218-19,160,345	AI, Loss		AI, Loss	AI, Gain	AI, Loss		AI		AI	AI, Loss
LOC284865	22	20,186,251-20,192,441	AI, Loss		AI, Loss	AI, Gain	AI, Loss		AI		AI	AI, Loss
LOC388849	22	20,136,108-20,137,431	AI, Loss		AI, Loss	AI, Gain	AI, Loss		AI		AI	AI, Loss
PI4KA	22	21,061,978-21,213,100	AI, Loss		AI, Loss	AI, Gain	AI, Loss		AI		AI	AI, Loss
SLC25A1	22	19,163,087-19,166,376	AI, Loss		AI, Loss	AI, Gain	AI, Loss		AI		AI	AI, Loss
ZDHHC8	22	20,119,363-20,135,530	AI, Loss		AI, Loss	AI, Gain	AI, Loss		AI		AI	AI, Loss
MIR185	22	20,020,661-20,020,743	AI, Loss		AI, Loss	AI, Gain	Loss		AI		AI	AI, Loss
TANGO2	22	20,004,522-20,054,687	AI, Loss		AI, Loss	AI, Gain	Loss		AI		AI	AI, Loss

AK129567	22	18,660,804-18,685,242	AI, Loss		AI, Loss	Gain	AI, Loss		AI		AI	AI, Loss
AK302545	22	18,721,396-18,736,925	AI, Loss		AI, Loss	Gain	AI, Loss		AI		AI	AI, Loss
AL117485	22	18,840,391-18,847,477	AI, Loss		AI, Loss	Gain	AI, Loss		AI		AI	AI, Loss
BC051721	22	18,837,630-18,843,431	AI, Loss		AI, Loss	Gain	AI, Loss		AI		AI	AI, Loss
BC112340	22	18,834,323-18,839,322	AI, Loss		AI, Loss	Gain	AI, Loss		AI		AI	AI, Loss
DGCR10	22	19,010,136-19,011,063	AI, Loss		AI, Loss	Gain	AI, Loss		AI		AI	AI, Loss
DGCR11	22	19,033,674-19,035,888	AI, Loss		AI, Loss	Gain	AI, Loss		AI		AI	AI, Loss
DGCR2	22	19,023,794-19,109,967	AI, Loss		AI, Loss	Gain	AI, Loss		AI		AI	AI, Loss
DGCR5	22	18,958,010-19,018,755	AI, Loss		AI, Loss	Gain	AI, Loss		AI		AI	AI, Loss
DGCR6	22	18,893,540-18,899,601	AI, Loss		AI, Loss	Gain	AI, Loss		AI		AI	AI, Loss
DGCR8	22	20,067,754-20,099,400	AI, Loss		AI, Loss	Gain	AI, Loss		AI		AI	AI, Loss
DGCR9	22	19,005,346-19,007,761	AI, Loss		AI, Loss	Gain	AI, Loss		AI		AI	AI, Loss
DQ585141	22	18,985,168-18,985,205	AI, Loss		AI, Loss	Gain	AI, Loss		AI		AI	AI, Loss
DQ786190	22	18,847,155-18,849,294	AI, Loss		AI, Loss	Gain	AI, Loss		AI		AI	AI, Loss
FAM230D	22	18,660,792-18,688,630	AI, Loss		AI, Loss	Gain	AI, Loss		AI		AI	AI, Loss
FAM230E	22	18,721,426-18,745,407	AI, Loss		AI, Loss	Gain	AI, Loss		AI		AI	AI, Loss
FAM230F	22	18,852,555-18,881,944	AI, Loss		AI, Loss	Gain	AI, Loss		AI		AI	AI, Loss

GGT3P	22	18,761,201-18,792,992	AI, Loss		AI, Loss	Gain	AI, Loss		AI		AI	AI, Loss
LOC102725072	22	18,837,630-18,848,564	AI, Loss		AI, Loss	Gain	AI, Loss		AI		AI	AI, Loss
LOC105379550	22	18,660,803-18,668,247	AI, Loss		AI, Loss	Gain	AI, Loss		AI		AI	AI, Loss
MIR6816	22	20,102,208-20,102,274	AI, Loss		AI, Loss	Gain	AI, Loss		AI		AI	AI, Loss
PRODH	22	18,900,286-18,924,066	AI, Loss		AI, Loss	Gain	AI, Loss		AI		AI	AI, Loss
RANBP1	22	20,103,460-20,114,880	AI, Loss		AI, Loss	Gain	AI, Loss		AI		AI	AI, Loss
SNORA77B	22	20,113,924-20,114,049	AI, Loss		AI, Loss	Gain	AI, Loss		AI		AI	AI, Loss
TRMT2A	22	20,099,388-20,104,818	AI, Loss		AI, Loss	Gain	AI, Loss		AI		AI	AI, Loss
TUBA8	22	18,593,452-18,614,498	AI, Loss		AI, Loss	Gain	AI, Loss		AI		AI	AI, Loss
USP18	22	18,632,757-18,660,162	AI, Loss		AI, Loss	Gain	AI, Loss		AI		AI	AI, Loss
Y_RNA	22	19,032,768-19,032,880	AI, Loss		AI, Loss	Gain	AI, Loss		AI		AI	AI, Loss
DQ571461	22	21,045,981-21,046,036	AI, Loss		AI, Loss		AI, Loss		AI		AI	AI, Loss
HV593110	22	21,083,681-21,083,711	AI, Loss		AI, Loss		AI, Loss		AI		AI	AI, Loss
HV593183	22	21,068,942-21,068,966	AI, Loss		AI, Loss		AI, Loss		AI		AI	AI, Loss
PEX26	22	18,560,685-18,573,797	AI, Loss		AI, Loss		AI, Loss		AI		AI	AI, Loss
POM121L4P	22	21,043,842-21,046,009	AI, Loss		AI, Loss		AI, Loss		AI		AI	AI, Loss
SERPIND1	22	21,128,382-21,142,008	AI, Loss		AI, Loss		AI, Loss		AI		AI	AI, Loss

TMEM191A	22	21,055,401-21,058,891	AI, Loss		AI, Loss		AI, Loss		AI		AI	AI, Loss
AIFM3	22	21,319,417-21,335,649	AI, Loss			AI, Gain	AI, Loss		AI		AI	AI, Loss
BC033281	22	21,305,018-21,308,035	AI, Loss			AI, Gain	AI, Loss		AI		AI	AI, Loss
BC127858	22	21,311,690-21,318,877	AI, Loss			AI, Gain	AI, Loss		AI		AI	AI, Loss
CRKL	22	21,271,713-21,308,037	AI, Loss			AI, Gain	AI, Loss		AI		AI	AI, Loss
LINC01637	22	21,311,379-21,318,968	AI, Loss			AI, Gain	AI, Loss		AI		AI	AI, Loss
SNAP29	22	21,213,291-21,245,501	AI, Loss			AI, Gain	AI, Loss		AI		AI	AI, Loss
MIR1306	22	20,073,580-20,073,665				Gain			AI		AI	AI, Loss
MIR3618	22	20,073,268-20,073,356				Gain			AI		AI	AI, Loss
COMT	22	19,929,262-19,957,498	AI, Loss		AI, Loss	AI, Gain	AI, Loss		AI	AI	AI, Loss	AI, Loss
GNB1L	22	19,775,933-19,842,462	AI, Loss		AI, Loss	AI, Gain	AI, Loss		AI	AI	AI, Loss	AI, Loss
TXNRD2	22	19,863,039-19,929,515	AI, Loss		AI, Loss	AI, Gain	AI, Loss		AI	AI	AI, Loss	AI, Loss
MIR4761	22	19,951,275-19,951,357	AI, Loss		AI, Loss	AI, Gain	AI, Loss		AI	AI	Loss	AI, Loss
BC038197	22	17,227,758-17,229,328	AI, Loss		Loss	AI, Gain	AI, Loss		AI		Loss	Loss
LINC01665	22	17,227,758-17,229,328	AI, Loss		Loss	AI, Gain	AI, Loss		AI		Loss	Loss
DQ571479	22	17,029,615-17,029,643	AI, Loss		Loss		AI, Loss		AI			Loss
PI4KAP2	22	21,827,286-21,871,780	AI, Loss	AI		Loss, LOH	AI, Loss		AI	AI, Loss	AI	

RIMBP3B	22	21,899,957-21,905,373	AI, Loss	AI		Loss, LOH	AI, Loss		AI	Loss	AI	
CECR2	22	17,840,838-18,037,856	AI, Loss		AI, Loss	AI, Gain	AI, Loss		AI		AI	
ADA2	22	17,659,679-17,702,744	AI, Loss		AI, Loss	Gain	AI, Loss		AI		AI	
CECR1	22	17,660,191-17,702,738	AI, Loss		AI, Loss	Gain	AI, Loss		AI		AI	
CECR5	22	17,618,409-17,646,177	AI, Loss		AI, Loss	Gain	AI, Loss		AI		AI	
CECR5-AS1	22	17,640,278-17,646,335	AI, Loss		AI, Loss	Gain	AI, Loss		AI		AI	
HDHD5	22	17,618,409-17,646,177	AI, Loss		AI, Loss	Gain	AI, Loss		AI		AI	
HDHD5-AS1	22	17,640,278-17,646,335	AI, Loss		AI, Loss	Gain	AI, Loss		AI		AI	
ATP6V1E1	22	18,074,902-18,111,588	AI, Loss		Loss	AI, Gain	AI, Loss		AI		AI	
BCL2L13	22	18,111,681-18,213,621	AI, Loss		Loss	AI, Gain	AI, Loss		AI		AI	
BID	22	18,216,905-18,257,431	AI, Loss		Loss	AI, Gain	AI, Loss		AI		AI	
DQ570096	22	18,215,565-18,217,980	AI, Loss		Loss	AI, Gain	AI, Loss		AI		AI	
LINC00528	22	18,260,055-18,262,247	AI, Loss		Loss	AI, Gain	AI, Loss		AI		AI	
LOC101929372	22	18,062,922-18,071,958	AI, Loss		Loss	AI, Gain	AI, Loss		AI		AI	
MIR3198-1	22	18,246,945-18,247,025	AI, Loss		Loss	AI, Gain	AI, Loss		AI		AI	
SLC25A18	22	18,043,138-18,073,656	AI, Loss		Loss	AI, Gain	AI, Loss		AI		AI	
HDHD1	X	6,966,960-7,066,231										AI
MIR4767	X	7,065,900-7,065,978										AI

MIR651	X	8,095,005-8,095,102									AI
PNPLA4	X	7,866,803-7,895,780									AI
PUDP	X	6,966,960-7,066,231									AI
STS	X	7,065,292-7,272,684									AI
VCX	X	7,810,302-7,812,184									AI
VCX2	X	8,137,984-8,139,308									AI
AK310094	X	23,801,293-23,801,569									Gain
AKAP17A	X	1,710,485-1,721,411									Gain
APOO	X	23,851,464-23,926,057									Gain
ASMT	X	1,714,347-1,761,974									Gain
ASMTL	X	1,522,031-1,572,655									Gain
ASMTL-AS1	X	1,519,423-1,534,314									Gain
CENPI	X	100,353,170-100,418,662									Gain
CRLF2	X	1,314,886-1,656,037									Gain
CSF2RA	X	1,387,692-1,428,828									Gain
CXorf58	X	23,926,122-23,957,624									Gain
DANT1	X	114,957,296-115,021,860									Gain
DANT2	X	114,956,728-115,085,444									Gain
DDX3X	X	41,192,560-41,223,725									Gain
DHRSX	X	2,137,554-2,419,015									Gain
DQ576039	X	800,013-800,044									Gain
DUSP21	X	44,703,248-44,704,134									Gain
EIF2S3	X	24,073,064-24,096,927									Gain
FAM120C	X	54,094,756-54,209,714									Gain
FUNDC1	X	44,382,884-44,402,221									Gain
IL3RA	X	1,455,508-1,501,582									Gain
KDM6A	X	44,732,420-44,971,857									Gain
KLHL15	X	24,001,832-24,045,303									Gain
LINC00106	X	1,515,917-1,518,291									Gain
LINC02601	X	41,134,991-41,136,030									Gain
LOC101928402	X	123,093,082-123,095,191									Gain
MIR3690	X	1,412,810-1,412,885									Gain
P2RY8	X	1,581,465-1,656,037									Gain
PHF8	X	53,963,112-54,071,569									Gain

PLS3	X	114,795,176-114,885,179										Gain
RPL9	X	23,854,760-23,855,459										Gain
SAT1	X	23,801,274-23,804,340										Gain
SLC25A6	X	1,505,044-1,511,039										Gain
STAG2	X	123,094,409-123,236,505										Gain
THOC2	X	122,734,411-122,866,904										Gain
USP9X	X	40,944,887-41,095,832										Gain
WNK3	X	54,219,255-54,384,438										Gain
XIAP	X	122,993,661-123,047,829										Gain
Y_RNA	X	23,800,326-23,800,427										Gain
ZFX	X	24,167,105-24,234,372										Gain
ZFX-AS1	X	24,164,341-24,167,771										Gain
ARMCX5-GPRASP2	X	101,854,275-102,163,032										Loss
GPRASP1	X	101,906,293-101,914,010										Loss
LINC01281	X	39,164,209-39,186,616										Loss
LINC01282	X	39,226,538-39,251,028										Loss
LOC286442	X	39,164,209-39,186,616										Loss
DMD	X	31,137,344-33,357,726	AI, Loss									
JA202295	X	32,503,097-32,503,131	AI, Loss									
JA202330	X	32,398,676-32,398,705	AI, Loss									
JA202335	X	32,583,973-32,584,010	AI, Loss									
JA783414	X	32,490,280-32,490,302	AI, Loss									
JA783415	X	32,482,743-32,482,766	AI, Loss									
JA783548	X	32,563,365-32,563,401	AI, Loss									
JA783560	X	32,486,729-32,486,759	AI, Loss									

JA783566	X	32,472,915-32,472,956	AI, Loss									
JA783574	X	32,408,163-32,408,193	AI, Loss									
JA783579	X	32,398,725-32,398,757	AI, Loss									
JA783587	X	32,383,263-32,383,303	AI, Loss									
JA783589	X	32,382,749-32,382,818	AI, Loss									
JA783867	X	32,563,416-32,563,458	AI, Loss									
MIR3915	X	32,601,772-32,601,869	AI, Loss									

Supplementary Table S3B. Known cancer genes affected by CNVs in 10 spinal meningiomas. Gene ID; gene name; chromosome number; gene genomic coordinates (hg19); role in cancer (fusion, oncogene or tumor suppressor gene (TSG)); and type of CNV (gain, loss, loss of heterozygosity (LOH) or allelic imbalance (AI)) in each individual tumor are shown from left to right.

Gene symbol	Gene name	Chr	Position	Role in cancer	P1_S1	P1_S2	P1_S3	P1_S5	P2_S1	P2_S2	P4_S1	P4_S2	P4_S3	P5_S1
FNBP1	formin binding protein 1 (FBP17)	9	132,649,465-132,805,473	fusion										Gain
ALDH2	aldehyde dehydrogenase 2 family (mitoch	12	112,204,690-112,247,789	fusion										Gain
ZCCHC8	zinc finger, CCHC domain containing 8	12	122,956,145-122,985,620	fusion										Gain
TRA	T cell receptor alpha locus	14	22,458,652-23,016,719	fusion	Gain	Gain	Gain	Gain	Gain	Gain	Gain	Gain	Gain	Gain
TRA	T cell receptor alpha locus	14	22,192,007-22,447,356	fusion	Gain	Gain	Gain	Gain	Gain	Gain	Gain	Gain	Gain	
TRD	T cell receptor delta locus	14	22,465,824-23,016,719	fusion	Gain	Gain	Gain	Gain	Gain	Gain	Gain	Gain	Gain	Gain
BCR	breakpoint cluster region	22	23,522,551-23,660,224	fusion	AI, Loss	AI	AI, Loss	Loss, LOH	AI, Loss	AI, Loss	AI	Loss	AI	AI, Loss
BCR	breakpoint cluster region	22	22,979,652-22,981,152	fusion	AI, Loss	AI	AI, Loss	Loss, LOH	AI, Loss	AI, Loss	AI		AI	AI, Loss
BCR	breakpoint cluster region	22	21,642,517-21,644,017	fusion	AI, Loss		Loss		AI, Loss		AI	AI	AI	AI, Loss
ABL2	c-abl oncogene 2, non-receptor tyrosine k	1	179,068,461-179,198,819	oncogene, fusion										Gain
XPO1	exportin 1 (CRM1 homolog, yeast)	2	61,705,068-61,765,418	oncogene										Gain
BIRC6	baculoviral IAP repeat containing 6	2	32,582,095-32,843,965	oncogene, fusion										Gain
RAC1	ras-related C3 botulinum toxin substrate 1	7	6,414,125-6,443,598	oncogene										Gain
BRAF	v-raf murine sarcoma viral oncogene hom	7	140,419,126-140,624,728	oncogene, fusion										Gain
HIP1	huntingtin interacting protein 1	7	75,162,618-75,368,290	oncogene, fusion										Gain
NT5C2	5'-nucleotidase, cytosolic II	10	104,847,773-104,953,063	oncogene										Gain
ATF1	activating transcription factor 1	12	51,157,788-51,214,943	oncogene, fusion										Gain
USP8	ubiquitin specific peptidase 8	15	50,716,573-50,793,280	oncogene										Gain
DGCR8	DGCR8, microprocessor complex subuni	22	20,067,754-20,099,400	oncogene	AI, Loss		AI, Loss	Gain	AI, Loss		AI		AI	AI, Loss
MAPK1	mitogen-activated protein kinase 1	22	22,113,946-22,221,970	oncogene	AI, Loss	AI	AI, Loss	Loss, LOH	AI, Loss	AI, Loss	AI	AI, Loss	AI	AI, Loss
EWSR1	Ewing sarcoma breakpoint region 1 (EWS	22	29,663,997-29,696,515	oncogene, fusion	AI, Loss	AI	AI, Loss	Loss, LOH	AI, Loss	AI, Loss	AI	AI, Loss	AI	Loss
MN1	MN1 proto-oncogene, transcriptional regu	22	28,144,264-28,197,486	oncogene, fusion	AI, Loss	AI	Loss	Loss, LOH	AI, Loss	AI, Loss	AI	AI, Loss	AI	AI, Loss
PDGFB	platelet-derived growth factor beta polype	22	39,619,363-39,641,060	oncogene, fusion	Loss	AI	Loss	Loss, LOH	Loss	AI, Loss	AI	AI	AI	Loss
CRLF2	cytokine receptor-like factor 2	X	1,314,886-1,656,037	oncogene, fusion										Gain
P2RY8	purinergic receptor P2Y, G-protein coupl	X	1,581,465-1,656,037	oncogene, fusion										Gain
ARNT	aryl hydrocarbon receptor nuclear translo	1	150,782,180-150,849,244	oncogene, TSG, fusion										Gain
TET1	tet methylcytosine dioxygenase 1	10	70,320,116-70,454,239	oncogene, TSG, fusion										Gain
APOBEC3B	apolipoprotein B mRNA editing enzyme	22	39,378,403-39,388,784	oncogene, TSG	Loss	AI	Loss	Loss, LOH	AI, Loss	AI, Loss	AI		AI	Loss
MKL1	megakaryoblastic leukemia (translocation	22	40,806,291-41,032,690	oncogene, TSG, fusion	Loss	AI, Loss	AI, Loss	Loss, LOH	AI, Loss	AI, Loss	AI	Loss	AI	Loss
KDM6A	lysine (K)-specific demethylase 6A, UTX	X	44,732,420-44,971,857	oncogene, TSG										Gain
ID3	inhibitor of DNA binding 3, HLH protein	1	23,884,420-23,886,285	TSG										Gain
STAG1	stromal antigen 1	3	136,055,998-136,471,245	TSG										Gain
N4BP2	NEDD4 binding protein 2	4	40,058,523-40,159,872	TSG										Gain
RAD17	RAD17 checkpoint clamp loader compon	5	68,665,123-68,710,630	TSG										Gain
SDHA	succinate dehydrogenase complex, subun	5	218,337-257,197	TSG										Loss
KMT2C	lysine (K)-specific methyltransferase 2C	7	151,832,009-152,133,090	TSG										Gain
PPP6C	protein phosphatase 6, catalytic subunit	9	127,908,851-127,952,218	TSG										Gain
PTPRD	protein tyrosine phosphatase, receptor typ	9	8,314,245-10,612,723	TSG			Loss							
CPEB3	cytoplasmic polyadenylation element bin	10	93,808,396-94,050,875	TSG										Gain
BAZ1A	bromodomain adjacent to zinc finger dom	14	35,221,936-35,344,853	TSG										Gain
RFWD3	ring finger and WD repeat domain 3	16	74,655,296-74,700,779	TSG										Gain
BRCA1	familial breast/ovarian cancer gene 1	17	41,196,311-41,322,420	TSG										Gain
CLTC	clathrin, heavy polypeptide (Hc)	17	57,697,049-57,774,317	TSG, fusion										Gain
YWHAE	tyrosine 3-monooxygenase/tryptophan 5-r	17	1,247,833-1,303,556	TSG, fusion										Gain
KEAP1	kelch like ECH associated protein 1	19	10,596,795-10,614,054	TSG										Gain
CHEK2	CHK2 checkpoint homolog (S. pombe)	22	29,083,730-29,137,822	TSG	AI, Loss	AI	AI, Loss	Loss, LOH	AI, Loss	AI, Loss	AI	AI, Loss	AI	
LZTR1	leucine-zipper-like transcription regulator	22	21,333,750-21,353,326	TSG	AI, Loss		Loss	AI	AI, Loss		AI	AI	AI	AI, Loss
NF2	neurofibromatosis type 2 gene	22	29,999,544-30,094,589	TSG	AI, Loss	AI	AI, Loss	Loss, LOH	AI, Loss	AI, Loss		AI, Loss	AI	Loss
SMARCB1	SWI/SNF related, matrix associated, actin	22	24,129,117-24,176,705	TSG	AI, Loss	AI, Loss	Loss	Loss, LOH	AI, Loss	AI, Loss	AI	AI	AI	AI, Loss
ZNRF3	zinc and ring finger 3	22	29,279,754-29,453,476	TSG	AI, Loss	AI	AI, Loss	Loss, LOH	AI, Loss	AI, Loss	AI	AI, Loss	AI, Loss	
CLTCL1	clathrin, heavy polypeptide-like 1	22	19,166,986-19,279,239	TSG, fusion	AI, Loss		AI, Loss	AI, Gain	AI, Loss	AI, Gain	AI	AI	AI	AI, Loss
EP300	300 kd E1A-Binding protein gene	22	41,488,613-41,576,081	TSG, fusion	Loss	AI	AI, Loss	Loss, LOH	Loss	AI, Loss	AI	Loss	AI	
MYH9	myosin, heavy polypeptide 9, non-muscle	22	36,677,322-36,784,112	TSG, fusion	AI, Loss	AI	Loss	Loss, LOH	AI, Loss	AI, Loss	AI	Loss	AI	AI, Loss
DDX3X	DEAD-box helicase 3, X-linked	X	41,192,560-41,223,725	TSG										Gain
STAG2	stromal antigen 2	X	123,094,409-123,236,505	TSG										Gain

ZNF429	zinc finger protein 429	19	21,688,353-21,739,070		Gain									
ISX	intestine specific homeobox	22	35,462,128-35,483,380		AI, Loss	AI	AI, Loss	Loss, LOH	AI, Loss	AI, Loss	AI	AI, Loss	AI	AI, Loss

Supplementary Table S3C. Genes affected by CNVs in 10 cranial meningiomas. Gene ID; chromosome number; gene genomic coordinates (hg19); and type of CNV (gain, loss or alleli imbalance (AI)) in each individual tumor are shown from left to right.

Gene ID	Chr	Position	P1_C1	P1_C2	P1_C3	P1_C4	P1_C5	P3_C1	P3_C2	P3_C3	P6_C1	P7_C1
7SK	1	33,802,166-33,802,465			AI						AI, Loss	
7SK	1	63,704,613-63,704,845			AI							
A3GALT2	1	33,772,366-33,786,699			AI						AI, Loss	
AADACL3	1	12,776,117-12,788,726			AI, Loss						Loss	
AADACL4	1	12,704,565-12,727,097			AI						Loss	
AB075489	1	40,222,853-40,225,715			AI						AI	
ACADM	1	76,190,031-76,229,363			AI, Loss							
ACOT11	1	55,007,929-55,100,417			AI, Loss							
ACOT7	1	6,324,331-6,453,826			AI						AI, Loss	
ACTRT2	1	2,938,045-2,939,467			AI						AI, Loss	
ADC	1	33,546,713-33,586,131			AI						AI, Loss	
ADGRB2	1	32,192,705-32,229,664			AI						AI, Loss	
ADGRL2	1	81,771,876-82,459,616			AI, Loss							
ADGRL4	1	79,355,448-79,472,495			AI, Loss							
AGBL4	1	48,998,526-50,489,626			AI, Loss							
AGBL4-IT1	1	49,839,872-49,937,757			AI, Loss							
AGO1	1	36,335,408-36,389,899			AI						AI, Gain	
AGO3	1	36,396,318-36,522,063			AI						AI, Gain	
AGO4	1	36,273,224-36,323,490			AI						AI, Gain	
AGRN	1	955,502-991,499			AI						Loss	
AGTRAP	1	11,796,141-11,810,828			AI						Loss	
AIM1L	1	26,648,349-26,680,621			AI, Loss						Loss	
AJAP1	1	4,715,104-4,843,851			AI						AI, Loss	
AK025726	1	36,391,432-36,395,210			AI						AI, Gain	
AK025975	1	23,243,782-23,247,347			AI							
AK026777	1	65,154,981-65,158,736			AI							
AK054635	1	24,291,417-24,295,600			AI							
AK054708	1	1,944,651-1,946,969			AI						AI, Loss	
AK055631	1	75,595,658-75,598,261			AI, Loss							
AK055853	1	15,653,175-15,670,372			AI						AI, Loss	
AK056486	1	846,814-850,328			AI						Loss	
AK090844	1	52,302,636-52,305,398			AI							
AK094692	1	1,535,818-1,543,166			AI						AI, Loss	
AK096291	1	64,571,005-64,636,980			AI							
AK097193	1	61,125,302-61,291,256			AI, Loss							
AK097571	1	53,793,904-53,802,889			AI							
AK097722	1	84,267,442-84,326,229			AI, Loss							
AK097814	1	1,656,053-1,663,343			AI						AI, Loss	
AK098438	1	21,749,600-21,754,300			AI							

AK123450	1	65,720,144-65,721,848			AI							
AK124197	1	14,146,461-14,150,513			AI						AI, Loss	
AK125078	1	5,621,768-5,728,315			AI						AI, Loss	
AK125437	1	11,782,186-11,785,914			AI						Loss	
AK127270	1	56,046,709-56,200,675			AI							
AK128734	1	65,338,382-65,343,856			AI							
AK2	1	33,473,540-33,502,512			AI						AI, Loss	
AK298300	1	67,132,271-67,142,710			AI							
AK304759	1	40,319,464-40,319,738			AI						AI	
AK309744	1	43,585,818-43,611,958			AI						AI	
AK310350	1	995,082-997,436			AI						Loss	
AK310751	1	674,239-679,736			AI						Loss	
AK4	1	65,613,231-65,697,828			AI							
AK5	1	77,747,661-78,025,654			AI, Loss							
AKIRIN1	1	39,456,915-39,471,737			AI						AI, Gain	
ALG6	1	63,833,297-63,904,233			AI							
ALPL	1	21,835,850-21,904,905			AI							
ANGPTL3	1	63,063,157-63,071,976			AI							
ANGPTL7	1	11,249,345-11,256,038			AI						Loss	
APITD1-CORT	1	10,490,158-10,512,060			AI, Loss						Loss	
ARHGEF16	1	3,371,146-3,397,677			AI						AI, Loss	
ARID1A	1	27,022,521-27,108,601			AI						Loss	
ASAP3	1	23,755,055-23,811,057			AI, Loss							
ASB17	1	76,384,557-76,398,116			AI, Loss							
ATG4C	1	63,249,776-63,330,941			AI							
ATPAF1	1	47,098,410-47,134,099			AI							
AUNIP	1	26,158,403-26,185,949			AI						AI, Loss	
AX746780	1	58,933,598-58,934,677			AI							
AX747064	1	33,607,471-33,608,831			AI						AI, Loss	
AX747125	1	8,043,018-8,045,341			AI						AI, Loss	
AX747205	1	25,629,228-25,631,643			AI						AI, Loss	
AX747207	1	25,227,540-25,230,414			AI						AI, Loss	
AX747530	1	2,112,574-2,114,663			AI						AI, Loss	
AX747565	1	32,095,467-32,098,435			AI						AI, Loss	
AX747766	1	21,602,542-21,604,868			AI							
AX748181	1	47,859,449-47,861,215			AI							
AX748204	1	23,853,364-23,855,542			AI							
AX748428	1	53,580,247-53,584,281			AI							
AZIN2	1	33,546,713-33,586,132			AI						AI, Loss	
BAI2	1	32,192,717-32,229,648			AI						AI, Loss	
BC016143	1	27,145,536-27,392,034			AI						Loss	
BC018779	1	2,121,236-2,123,179			AI						AI, Loss	
BC020917	1	68,962,358-69,004,310			AI, Loss							
BC030753	1	61,405,915-61,436,448			AI							

BC030768	1	26,551,810-26,556,331			AI						AI, Loss	
BC033949	1	995,116-1,001,833			AI						Loss	
BC033978	1	58,326,265-58,328,786			AI							
BC035370	1	65,445,259-65,468,159			AI							
BC036251	1	420,205-421,839			AI						Loss	
BC036308	1	33,452,675-33,498,070			AI						AI, Loss	
BC036594	1	84,041,470-84,326,679			AI, Loss							
BC037304	1	84,543,635-84,546,350			AI, Loss							
BC037321	1	4,847,557-4,852,183			AI						AI, Loss	
BC038455	1	23,801,092-23,803,135			AI							
BC040909	1	64,560,124-64,577,888			AI							
BC041341	1	73,771,852-73,804,560			AI, Loss							
BC041441	1	71,172,135-71,252,151			AI, Loss							
BC042048	1	60,238,466-60,254,501			AI							
BC042538	1	30,486,798-30,510,456			AI						AI, Loss	
BC043544	1	83,911,736-83,920,454			AI							
BC047487	1	58,326,214-58,328,786			AI							
BC069257	1	32,256,024-32,258,424			AI, Loss						AI, Loss	
BC069694	1	40,420,819-40,422,587			AI						AI	
BC113958	1	8,440,651-8,441,235			AI						Loss	
BC127868	1	21,761,832-21,762,609			AI							
BMP8A	1	39,957,317-39,995,541			AI						AI	
BMP8B	1	40,222,853-40,254,533			AI						AI	
BSDC1	1	32,830,233-32,860,062			AI						AI, Loss	
BTF3L4	1	52,521,856-52,556,388			AI							
C1orf123	1	53,679,771-53,686,289			AI, Loss							
C1orf127	1	11,006,529-11,042,094			AI, Loss						Loss	
C1orf141	1	67,557,858-67,600,654			AI, Loss							
C1orf158	1	12,806,133-12,821,102			Loss						Loss	
C1orf159	1	1,017,197-1,051,736			AI						Loss	
C1orf167	1	11,822,249-11,849,642			AI						Loss	
C1orf168	1	57,184,476-57,285,369			AI, Loss							
C1orf172	1	27,276,046-27,286,901			AI						Loss	
C1orf173	1	75,033,794-75,139,422			AI, Loss							
C1orf174	1	3,805,696-3,816,857			AI						AI, Loss	
C1orf185	1	51,567,899-51,613,757			Loss							
C1orf195	1	15,490,691-15,498,120			AI						AI, Loss	
C1orf210	1	43,747,555-43,751,288			AI						AI	
C1orf211	1	6,297,870-6,299,502			AI						AI, Loss	
C1orf213	1	23,695,463-23,698,330			AI							
C1orf222	1	1,853,395-1,859,368			AI						AI, Loss	
C1orf232	1	26,490,651-26,495,073			AI						AI, Loss	
C1orf63	1	25,568,739-25,664,656			AI						AI, Loss	
C1orf64	1	16,330,730-16,333,184			AI						AI, Loss	

C1orf86	1	2,115,898-2,139,172			AI						AI, Loss	
C1orf87	1	60,454,823-60,539,442			AI							
C1orf94	1	34,632,483-34,684,731			AI, Loss						AI, Loss	
C1QA	1	22,962,998-22,966,175			AI							
C1QB	1	22,979,473-22,988,129			AI							
C1QC	1	22,970,109-22,974,604			AI							
C8A	1	57,320,442-57,383,894			AI, Loss							
C8B	1	57,394,882-57,431,813			AI							
CACHD1	1	64,935,811-65,158,741			AI							
CALML6	1	1,846,265-1,848,733			AI						AI, Loss	
CAMK2N1	1	20,808,883-20,812,728			AI							
CAP1	1	40,505,736-40,538,321			AI						AI	
CASZ1	1	10,696,665-10,856,733			AI						Loss	
CATSPER4	1	26,517,118-26,529,033			AI						AI, Loss	
CC2D1B	1	52,816,264-52,831,877			AI, Loss							
CCDC27	1	3,668,964-3,688,209			AI						AI, Loss	
CCDC28B	1	32,665,986-32,670,991			AI						AI, Loss	
CDA	1	20,915,443-20,945,400			AI							
CDC20	1	43,824,625-43,828,873			AI						AI	
CDC2L1	1	1,586,822-1,590,469			AI						AI, Loss	
CDC42	1	22,379,119-22,419,436			AI							
CDK11A	1	1,633,822-1,656,004			AI						AI, Loss	
CDK11B	1	1,577,746-1,580,625			AI						AI, Loss	
CDK11B	1	1,570,602-1,655,859			AI						AI, Loss	
CELA2A	1	15,783,222-15,798,586			Loss						AI, Loss	
CELA3A	1	22,328,148-22,339,035			AI							
CELA3B	1	22,303,386-22,333,141			AI							
CENPS-CORT	1	10,490,158-10,512,060			AI, Loss						Loss	
CEP104	1	3,728,644-3,773,797			AI						AI, Loss	
CEP85	1	26,560,643-26,605,301			AI						AI, Loss	
CFAP57	1	43,638,000-43,720,029			AI						AI	
CFAP74	1	1,853,389-1,935,276			AI						AI, Loss	
CFHR5	1	196,946,666-196,978,803			Loss							
CHD5	1	6,161,846-6,240,194			AI						AI, Loss	
CITED4	1	41,326,727-41,328,018			AI						AI	
CLCA4	1	87,012,758-87,046,432			Loss							
CLCA4-AS1	1	87,036,863-87,170,176			Loss							
CLCN6	1	11,866,152-11,903,201			AI						Loss	
CLCNKA	1	16,345,369-16,360,545			AI						AI, Loss	
CLCNKB	1	16,355,620-16,383,821			AI						AI, Loss	
CLIC4	1	25,071,759-25,170,815			AI						AI, Loss	
CNKSR1	1	26,503,980-26,516,376			AI						AI, Loss	
CNR2	1	24,200,459-24,239,817			AI							
COA7	1	53,152,013-53,164,038			AI							

COL16A1	1	32,117,847-32,169,768			AI						AI, Loss	
COL9A2	1	40,766,162-40,783,060			AI						Gain	
CORT	1	10,509,775-10,512,060			AI, Loss						Loss	
CPT2	1	53,662,100-53,679,869			AI, Loss							
CR627203	1	75,043,113-75,091,782			Loss							
CR936677	1	76,103,850-76,188,721			AI, Loss							
CRYBG2	1	26,648,349-26,680,621			AI, Loss						Loss	
CRYZ	1	75,171,171-75,199,092			AI, Loss							
CSF3R	1	36,931,643-36,948,915			AI						AI	
CSMD2	1	33,979,598-34,631,443			AI, Loss						AI, Loss	
CSMD2-AS1	1	34,334,556-34,351,059			AI						AI, Loss	
CTH	1	70,876,900-70,905,534			Loss							
CTRC	1	15,764,937-15,773,153			Loss						AI, Loss	
CYP2J2	1	60,358,979-60,392,470			AI, Loss							
CYP4A11	1	47,394,845-47,407,157			AI							
CYP4A22	1	47,603,096-47,614,526			AI							
CYP4B1	1	47,264,669-47,285,021			AI							
CYP4X1	1	47,427,035-47,516,426			AI							
CYP4Z1	1	47,533,159-47,583,992			AI							
CYP4Z2P	1	47,308,766-47,366,147			AI							
CZIB	1	53,679,771-53,686,311			AI, Loss							
DAB1	1	57,460,450-59,012,446			AI, Loss							
DAB1-AS1	1	58,326,252-58,328,888			AI							
DCDC2B	1	32,674,694-32,681,797			AI						AI, Loss	
DDOST	1	20,978,259-20,988,037			AI							
DDX11L1	1	11,873-14,409			AI						Loss	
DEPDC1	1	68,939,834-68,962,904			AI, Loss							
DEPDC1-AS1	1	68,962,358-69,004,310			AI, Loss							
DFFA	1	10,520,602-10,532,613			AI, Loss						Loss	
DFFB	1	3,773,830-3,801,993			AI						AI, Loss	
DHRS3	1	12,627,938-12,677,820			AI						Loss	
DIRAS3	1	68,511,644-68,517,314			AI							
DISP3	1	11,539,294-11,597,640			AI						Loss	
DL489965	1	43,637,279-43,637,416			AI						AI	
DLEU2L	1	64,014,650-64,016,307			Loss							
DLGAP3	1	35,331,036-35,395,186			AI, Loss						AI, Loss	
DLSTP1	1	76,207,686-76,210,698			AI, Loss							
DMBX1	1	46,972,667-46,979,886			AI							
DMRTB1	1	53,925,071-53,933,160			AI							
DNAJC6	1	65,720,147-65,881,552			AI							
DNASE2B	1	84,864,214-84,880,691			AI							
DOCK7	1	62,920,396-63,154,039			AI, Loss							
DQ575786	1	668,417-668,479			AI						Loss	
DQ576383	1	16,317,618-16,317,647			AI						AI, Loss	

DQ588542	1	24,320,925-24,320,957			AI							
DQ597235	1	321,083-321,115			AI						Loss	
DQ599768	1	321,145-321,207			AI						Loss	
DQ599872	1	668,509-668,541			AI						Loss	
DRAXIN	1	11,751,780-11,780,336			AI						Loss	
E2F2	1	23,832,919-23,857,712			AI							
EBNA1BP2	1	43,629,844-43,736,607			AI						AI	
ECE1	1	21,543,739-21,672,034			AI							
ECHDC2	1	53,361,581-53,392,851			AI, Loss							
EFCAB14	1	47,124,358-47,184,736			AI							
EFCAB14-AS1	1	47,139,707-47,157,769			AI							
EFCAB7	1	63,988,971-64,038,364			AI, Loss							
EFHD2	1	15,736,390-15,756,839			AI, Loss						AI, Loss	
EIF3I	1	32,687,184-32,697,205			AI						AI, Loss	
EIF4G3	1	21,132,784-21,503,381			AI							
ELAVL4	1	50,513,685-50,669,457			AI, Loss							
ELOA	1	24,069,855-24,088,549			AI							
ELOA-AS1	1	24,086,871-24,104,787			AI							
ELOVL1	1	43,829,067-43,833,745			AI						AI	
ELTD1	1	79,355,448-79,472,495			AI, Loss							
EPB41	1	29,213,602-29,446,558			AI, Loss						Loss	
EPHA2	1	16,450,831-16,482,604			AI						AI, Loss	
EPHA8	1	22,889,994-22,930,087			AI, Loss							
EPHB2	1	23,037,330-23,247,993			AI							
EPS15	1	51,819,934-51,984,995			AI							
ERICH3	1	75,033,794-75,139,422			AI, Loss							
ERICH3-AS1	1	75,043,113-75,091,782			Loss							
ESPN	1	6,484,835-6,521,004			AI						AI, Loss	
EVA1B	1	36,787,630-36,789,755			AI						AI	
EXO5	1	40,974,390-40,982,214			AI						AI, Gain	
EXOSC10	1	11,126,669-11,159,967			AI						Loss	
EXOSC10-AS1	1	11,159,731-11,162,162			AI						Loss	
EXTL1	1	26,348,270-26,362,954			AI						AI, Loss	
EYA3	1	28,296,854-28,415,148			AI						Loss	
FAAH	1	46,859,938-46,879,520			AI							
FAAHP1	1	46,899,498-46,911,374			AI							
FAAP20	1	2,115,898-2,144,159			AI						AI, Loss	
FABP3	1	31,838,099-31,846,135			AI						AI, Loss	
FAM110D	1	26,485,510-26,489,119			AI						AI, Loss	
FAM131C	1	16,384,263-16,400,127			AI						AI, Loss	
FAM138A	1	34,610-36,081			AI						Loss	
FAM138F	1	34,610-36,081			AI						Loss	
FAM151A	1	55,074,849-55,089,200			AI, Loss							
FAM159A	1	53,099,065-53,135,339			AI							

FAM167B	1	32,712,817-32,714,461			AI						AI, Loss	
FAM183A	1	43,613,593-43,622,067			AI						AI	
FAM213B	1	2,517,898-2,522,908			AI						AI, Loss	
FAM229A	1	32,826,870-32,827,844			AI						AI, Loss	
FAM41C	1	803,450-812,182			AI						Loss	
FAM43B	1	20,878,931-20,881,513			AI							
FAM46B	1	27,331,510-27,339,333			AI						Loss	
FAM76A	1	28,052,489-28,089,423			AI						Loss	
FAM87B	1	752,750-755,214			AI						Loss	
FBLIM1	1	16,083,153-16,113,089			AI, Loss						AI, Loss	
FBXO2	1	11,708,417-11,714,888			AI						Loss	
FBXO44	1	11,714,431-11,723,384			AI						Loss	
FBXO6	1	11,724,149-11,734,409			AI						Loss	
FGGY	1	59,762,309-60,228,402			AI							
FHAD1	1	15,573,720-15,726,778			AI						AI, Loss	
FLJ37453	1	16,160,709-16,174,642			AI						AI, Loss	
FLJ42875	1	2,976,180-2,980,350			AI						AI, Loss	
FLJ42875	1	2,980,635-2,984,289			AI						AI, Loss	
FMO2	1	171,154,346-171,181,822			Gain							
FNDC5	1	33,327,868-33,338,093			AI						AI, Loss	
FOXD2	1	47,901,688-47,906,363			AI							
FOXD2-AS1	1	47,897,806-47,900,313			AI							
FOXD3	1	63,788,729-63,790,797			AI							
FOXD3-AS1	1	63,786,554-63,790,112			AI							
FOXE3	1	47,881,743-47,883,724			AI							
FPGT	1	74,663,895-74,674,386			AI, Loss							
FPGT-TNNI3K	1	74,663,895-75,010,116			AI, Loss							
FUCA1	1	24,171,571-24,194,859			AI							
FYB2	1	57,184,476-57,285,369			AI, Loss							
GABRD	1	1,950,767-1,962,192			AI						AI, Loss	
GADD45A	1	68,150,859-68,154,021			AI							
GALE	1	24,122,088-24,127,294			AI							
GJA4	1	35,258,170-35,261,348			Loss						AI, Loss	
GJA9	1	39,339,738-39,347,298			AI						AI	
GJA9-MYCBP	1	39,328,161-39,347,298			AI						AI	
GJB3	1	35,246,789-35,251,967			AI, Loss						AI, Loss	
GJB4	1	35,225,341-35,229,325			AI						AI, Loss	
GJB5	1	35,220,647-35,224,113			AI						AI, Loss	
GLIS1	1	53,971,905-54,199,877			AI							
GNB1	1	1,716,723-1,822,556			AI						AI, Loss	
GNG12	1	68,167,148-68,299,155			AI							
GNG12-AS1	1	68,297,970-68,668,670			AI, Loss							
GPATCH3	1	27,216,978-27,226,962			AI						Loss	
GPN2	1	27,205,872-27,216,869			AI						Loss	

GPR153	1	6,307,405-6,321,035			AI						AI, Loss	
GPR157	1	9,164,475-9,189,229			AI						Loss	
GPX7	1	53,068,042-53,074,723			AI							
GRIK3	1	37,261,127-37,499,844			AI, Loss						AI	
H6PD	1	9,294,862-9,331,394			AI						Loss	
HCRTR1	1	32,083,300-32,098,119			AI, Loss						AI, Loss	
HDAC1	1	32,757,707-32,799,224			AI						AI, Loss	
HEATR8-TTC4	1	55,107,426-55,208,328			AI, Loss							
HES2	1	6,472,497-6,484,730			AI						AI, Loss	
HES3	1	6,304,251-6,305,638			AI						AI, Loss	
HES5	1	2,460,183-2,461,684			AI						AI, Loss	
HEYL	1	40,089,102-40,105,348			AI						AI	
HH834010	1	84,259,583-84,259,634			AI							
HIVEP3	1	41,972,035-42,501,596			AI, Loss							
HMGB4	1	34,326,075-34,330,392			AI						AI, Loss	
HMGCL	1	24,128,366-24,165,110			AI							
HNRNPR	1	23,631,180-23,670,857			AI							
HOOK1	1	60,280,462-60,342,050			AI, Loss							
HP1BP3	1	21,069,170-21,113,799			AI, Loss							
HPCA	1	33,352,097-33,360,247			AI						AI, Loss	
HPCAL4	1	40,144,319-40,157,382			AI						AI	
HSD52	1	59,597,607-59,612,479			AI							
HSPB7	1	16,340,522-16,346,089			AI						AI, Loss	
HSPG2	1	22,148,724-22,263,790			AI, Loss							
HTR1D	1	23,518,387-23,521,222			AI							
ICMT	1	6,281,252-6,296,044			AI						AI, Loss	
ID3	1	23,884,420-23,886,285			AI							
IFI44	1	79,115,473-79,129,763			AI							
IFI44L	1	79,086,066-79,111,830			AI							
IFI6	1	27,992,571-27,998,724			AI						Loss	
IFNLR1	1	24,480,646-24,513,765			AI, Loss							
IL12RB2	1	67,773,046-67,862,583			AI							
IL23R	1	67,604,589-67,725,650			AI, Loss							
INADL	1	62,208,148-62,644,347			AI, Loss							
INSL5	1	67,263,423-67,266,942			Loss							
IPP	1	46,159,997-46,216,485			AI, Loss							
IQCC	1	32,671,235-32,674,288			AI						AI, Loss	
ITGB3BP	1	63,906,440-63,988,944			AI							
JA375062	1	47,691,628-47,691,655			AI							
JA429830	1	566,092-566,115			AI						Loss	
JA429831	1	566,134-566,155			AI						Loss	
JAK1	1	65,298,905-65,533,429			AI							
JB137814	1	566,239-566,263			AI						Loss	
JB175146	1	68,649,301-68,649,321			AI							

JUN	1	59,246,462-59,249,785			AI							
KANK4	1	62,701,836-62,785,085			Loss							
KAZN	1	14,925,212-15,444,544			AI						AI, Loss	
KAZN-AS1	1	14,675,449-14,746,469			AI						AI, Loss	
KCNAB2	1	6,052,357-6,161,253			AI						AI, Loss	
KCNQ4	1	41,249,683-41,306,124			AI						AI	
KDF1	1	27,276,046-27,286,901			AI						Loss	
KDM1A	1	23,345,940-23,410,184			AI							
KHDRBS1	1	32,479,294-32,526,460			AI						AI, Loss	
KIAA0754	1	39,875,175-39,882,154			AI, Loss						AI	
KIAA1522	1	33,207,511-33,240,571			AI						AI, Loss	
KIAA1751	1	1,884,751-1,935,276			AI						AI, Loss	
KIAA2013	1	11,979,644-11,986,485			AI						Loss	
KIF17	1	20,990,506-21,044,510			AI, Loss							
KLHDC7A	1	18,807,423-18,812,540			Loss							
KLHL17	1	895,966-901,099			AI						Loss	
KNCN	1	47,011,309-47,017,329			AI							
KPNA6	1	32,573,643-32,642,168			AI						AI, Loss	
KTI12	1	52,497,776-52,499,472			AI							
L1TD1	1	62,660,473-62,678,001			Loss							
LACTBL1	1	23,279,535-23,299,359			AI							
LAPTM5	1	31,205,314-31,230,683			AI						AI, Loss	
LCK	1	32,716,839-32,751,768			AI						AI, Loss	
LDLRAD1	1	54,472,970-54,483,859			Loss							
LDLRAD2	1	22,138,757-22,151,714			AI							
LDLRAP1	1	25,870,075-25,895,377			AI						AI, Loss	
LEPR	1	65,886,130-66,103,176			AI							
LEPROT	1	65,886,130-65,901,690			AI							
LHX8	1	75,594,118-75,627,218			AI, Loss							
LINC00115	1	761,585-762,902			AI						Loss	
LINC00337	1	6,296,299-6,299,502			AI						AI, Loss	
LINC00339	1	22,351,683-22,357,717			AI							
LINC00466	1	63,624,753-63,782,928			AI, Loss							
LINC00853	1	47,644,921-47,646,011			AI							
LINC00982	1	2,976,180-2,984,289			AI						AI, Loss	
LINC01128	1	762,970-794,826			AI						Loss	
LINC01134	1	3,816,967-3,832,011			AI						AI, Loss	
LINC01135	1	59,250,822-59,365,384			AI							
LINC01141	1	20,686,293-20,755,287			AI							
LINC01225	1	31,971,838-31,974,167			AI						AI, Loss	
LINC01226	1	31,984,035-31,989,846			AI, Loss						AI, Loss	
LINC01342	1	1,072,396-1,079,434			AI						Loss	
LINC01345	1	4,007,208-4,009,040			AI						AI, Loss	
LINC01346	1	4,000,671-4,012,643			AI						AI, Loss	

LINC01355	1	23,607,801-23,612,011			AI							
LINC01358	1	59,486,147-59,553,919			AI							
LINC01359	1	65,445,259-65,468,159			AI							
LINC01360	1	73,771,852-73,804,560			AI, Loss							
LINC01362	1	83,368,865-83,632,498			AI, Loss							
LINC01389	1	47,846,467-47,902,989			AI, Loss							
LINC01398	1	46,912,344-46,915,376			AI							
LINC01562	1	51,660,766-51,700,768			AI, Loss							
LINC01635	1	22,350,486-22,352,541			AI							
LINC01646	1	4,631,546-4,643,764			AI, Loss						AI, Loss	
LINC01647	1	11,669,587-11,673,411			AI						Loss	
LINC01648	1	30,486,798-30,510,459			AI						AI, Loss	
LINC01707	1	69,521,580-69,650,685			AI, Loss							
LINC01712	1	83,911,736-83,920,454			AI							
LINC01725	1	84,041,470-84,326,679			AI, Loss							
LINC01739	1	63,466,167-63,478,142			AI							
LINC01748	1	60,981,387-61,088,357			AI							
LINC01753	1	56,381,281-56,410,636			AI							
LINC01755	1	56,410,759-56,415,792			AI							
LINC01756	1	29,656,131-29,676,626			AI						AI, Loss	
LINC01757	1	20,569,587-20,571,147			AI							
LINC01758	1	69,898,937-69,901,092			AI							
LINC01767	1	56,880,634-56,881,638			AI							
LINC01771	1	53,793,904-53,802,181			AI							
LINC01777	1	4,472,110-4,484,744			AI, Loss						AI, Loss	
LINC01778	1	31,297,498-31,307,131			AI						AI, Loss	
LINC01781	1	81,001,439-81,112,473			AI, Loss							
LINC01788	1	71,172,135-71,252,151			AI, Loss							
LINC02238	1	74,140,361-74,204,325			AI, Loss							
LINC02574	1	27,986,838-27,989,233			AI						Loss	
LINC02593	1	852,197-855,072			AI						Loss	
LNCTAM34A	1	9,242,262-9,252,144			AI						Loss	
LOC100129534	1	2,281,852-2,284,100			AI						AI, Loss	
LOC100130417	1	852,952-854,817			AI						Loss	
LOC100130557	1	41,154,751-41,157,933			AI						AI	
LOC100131060	1	59,250,822-59,365,384			AI							
LOC100132062	1	323,891-328,581			AI						Loss	
LOC100132287	1	323,891-328,581			AI						Loss	
LOC100133331	1	322,036-328,581			AI						Loss	
LOC100133331	1	661,138-679,736			AI						Loss	
LOC100133445	1	2,486,162-2,488,450			AI						AI, Loss	
LOC100133612	1	3,816,967-3,833,879			AI						AI, Loss	
LOC100288069	1	700,244-714,068			AI						Loss	
LOC100288175	1	995,113-1,001,833			AI						Loss	

LOC100506801	1	21,619,782-21,626,362			AI							
LOC100506963	1	24,086,871-24,104,787			AI							
LOC100506985	1	24,865,763-24,882,515			AI						AI, Loss	
LOC100507564	1	53,704,281-53,708,455			AI							
LOC100507634	1	55,681,080-55,683,128			AI							
LOC100996583	1	2,497,973-2,515,973			AI						AI, Loss	
LOC101926944	1	60,238,466-60,254,501			AI							
LOC101926964	1	61,125,302-61,291,256			AI, Loss							
LOC101927139	1	66,508,182-66,516,401			AI							
LOC101927342	1	76,477,008-76,485,041			AI, Loss							
LOC101927417	1	15,653,175-15,670,379			AI						AI, Loss	
LOC101927434	1	81,979,564-82,023,387			AI, Loss							
LOC101927560	1	84,267,198-84,326,229			AI, Loss							
LOC101928163	1	23,848,421-23,854,911			AI							
LOC101928303	1	26,551,810-26,556,331			AI						AI, Loss	
LOC101928626	1	562,759-564,389			AI						Loss	
LOC101928728	1	27,018,400-27,020,622			AI						Loss	
LOC101929464	1	33,815,952-33,828,846			AI						AI, Loss	
LOC101929536	1	40,254,647-40,255,842			AI						AI	
LOC101929721	1	49,723,082-49,734,966			AI, Loss							
LOC101929935	1	57,093,463-57,110,991			AI							
LOC102724450	1	6,264,899-6,265,840			AI						AI, Loss	
LOC102724659	1	11,837,133-11,839,676			AI						Loss	
LOC102725121	1	11,868-14,362			AI						Loss	
LOC105378586	1	1,534,812-1,542,590			AI						AI, Loss	
LOC105378591	1	1,980,639-1,981,509			AI						AI, Loss	
LOC105378663	1	39,339,072-39,341,898			AI						AI	
LOC105378696	1	46,998,665-47,001,187			AI							
LOC110117498-PIK3R3	1	46,505,811-46,642,167			Loss							
LOC115110	1	2,481,358-2,484,284			AI						AI, Loss	
LOC149086	1	31,971,838-31,974,167			AI						AI, Loss	
LOC254099	1	1,072,396-1,079,434			AI						Loss	
LOC284551	1	31,984,035-31,989,846			AI, Loss						AI, Loss	
LOC284661	1	4,472,110-4,484,744			AI, Loss						AI, Loss	
LOC339505	1	20,686,293-20,755,287			AI							
LOC388312	1	327,545-328,439			AI						Loss	
LOC402779	1	34,334,556-34,351,059			AI						AI, Loss	
LOC643837	1	762,970-794,826			AI						Loss	
LOC646471	1	26,146,444-26,150,097			AI						AI, Loss	
LOC653160	1	35,441,299-35,450,948			Loss							
LOC728716	1	4,000,671-4,012,643			AI						AI, Loss	
LOC729041	1	46,899,498-46,911,374			AI							
LOC729059	1	23,337,326-23,342,343			AI							
LOC729737	1	134,772-140,566			AI						Loss	

LPHN2	1	81,771,844-82,458,107			AI, Loss							
LRP8	1	53,708,040-53,793,821			AI							
LRRC40	1	70,610,484-70,671,361			Loss							
LRRC41	1	46,744,071-46,769,038			AI, Loss							
LRRC42	1	54,411,998-54,433,841			AI, Loss							
LRRC47	1	3,696,783-3,713,068			AI						AI, Loss	
LRRC53	1	74,934,663-74,978,298			AI							
LRRC7	1	70,032,867-70,610,046			AI, Loss							
LRRIQ3	1	74,491,701-74,663,871			AI, Loss							
LSM10	1	36,859,020-36,863,560			AI						AI	
LUZP1	1	23,410,515-23,504,301			AI							
LYPLA2	1	24,117,645-24,122,029			AI							
M37726	1	568,843-568,913			AI						Loss	
MACF1	1	39,670,422-39,748,740			AI						AI, Gain	
MACF1	1	39,547,088-39,952,810			AI, Loss						AI, Gain	
MACO1	1	25,757,348-25,826,698			AI						AI, Loss	
MAD2L2	1	11,734,536-11,751,678			AI						Loss	
MAGOH	1	53,692,563-53,704,282			AI							
MAN1C1	1	25,943,958-26,112,698			AI						AI, Loss	
MAP7D1	1	36,621,565-36,646,451			AI						AI, Gain	
MARCKSL1	1	32,799,429-32,801,840			AI						AI, Loss	
MASP2	1	11,086,579-11,107,296			AI						Loss	
MAST2	1	46,269,278-46,501,796			AI, Loss							
MATN1	1	31,184,123-31,196,432			AI						AI, Loss	
MATN1-AS1	1	31,191,618-31,199,593			AI						AI, Loss	
MDS2	1	23,907,984-23,967,058			AI							
MED8	1	43,849,578-43,855,483			AI						AI	
MEGF6	1	3,404,505-3,528,059			AI						AI, Loss	
MFN2	1	12,040,237-12,073,572			AI						Loss	
MFSD2A	1	40,420,783-40,435,640			AI						AI	
MGC27382	1	78,695,282-78,835,147			AI, Loss							
MGC34796	1	62,119,913-62,121,800			AI							
MIB2	1	1,550,794-1,565,990			AI						AI, Loss	
MIIP	1	12,079,298-12,092,106			AI						Loss	
mir-34	1	9,208,345-9,242,451			AI						Loss	
MIR101-1	1	65,524,116-65,524,191			AI							
MIR1256	1	21,314,806-21,314,925			AI							
MIR1262	1	68,649,200-68,649,293			AI							
MIR1302-10	1	30,365-30,503			AI						Loss	
MIR1302-11	1	30,365-30,503			AI						Loss	
MIR1302-2	1	30,365-30,503			AI						Loss	
MIR1302-9	1	30,365-30,503			AI						Loss	
MIR186	1	71,533,313-71,533,399			AI							
MIR30C1	1	41,222,955-41,223,044			AI						AI	

MIR30E	1	41,220,026-41,220,118			AI						AI	
MIR3115	1	23,370,797-23,370,865			AI							
MIR3116-1	1	62,544,457-62,544,531			AI							
MIR3116-2	1	62,544,460-62,544,528			AI							
MIR3117	1	67,094,122-67,094,200			AI							
MIR34A	1	9,211,726-9,211,836			AI						Loss	
MIR34AHG	1	9,208,069-9,242,397			AI						Loss	
MIR3605	1	33,797,993-33,798,093			AI						AI, Loss	
MIR3671	1	65,523,437-65,523,525			AI							
MIR378F	1	24,255,559-24,255,637			AI							
MIR3917	1	26,232,852-26,232,945			AI						AI, Loss	
MIR3972	1	17,604,383-17,604,470			AI, Loss							
MIR4251	1	3,044,538-3,044,599			AI						AI, Loss	Loss
MIR4252	1	6,489,893-6,489,956			AI						AI, Loss	
MIR4253	1	23,189,651-23,189,719			AI							
MIR4254	1	32,224,260-32,224,336			AI						AI, Loss	
MIR4255	1	37,627,163-37,627,235			AI, Loss						AI	
MIR4417	1	5,624,130-5,624,203			AI						AI, Loss	
MIR4418	1	22,592,731-22,592,793			AI							
MIR4420	1	31,212,002-31,212,079			AI						AI, Loss	
MIR4422	1	55,691,313-55,691,396			AI							
MIR4422HG	1	55,683,533-55,699,850			AI							
MIR4425	1	25,349,993-25,350,077			AI						AI, Loss	
MIR4632	1	12,251,769-12,251,830			AI							
MIR4684	1	23,046,009-23,046,091			AI							
MIR4689	1	5,922,731-5,922,801			AI						AI, Loss	
MIR4711	1	60,198,898-60,198,968			AI							
MIR4781	1	54,519,751-54,519,827			Loss							
MIR4794	1	65,045,529-65,045,604			AI							
MIR548AP	1	84,259,597-84,379,059			AI, Loss							
MIR551A	1	3,477,259-3,477,354			AI						AI, Loss	
MIR552	1	35,135,199-35,135,295			AI						AI, Loss	
MIR5585	1	32,552,549-32,552,608			AI						AI, Loss	
MIR6068	1	63,792,595-63,792,655			AI							
MIR6084	1	20,960,171-20,960,281			AI							
MIR6127	1	22,959,750-22,959,859			AI							
MIR6729	1	12,089,214-12,089,279			AI						Loss	
MIR6730	1	12,638,984-12,639,051			AI							
MIR6731	1	25,245,835-25,245,907			AI						AI, Loss	
MIR6733	1	43,637,322-43,637,383			AI						AI	
MIR6734	1	43,830,318-43,830,386			AI						AI	
MIR6859-1	1	17,368-17,436			AI						Loss	
MIR6859-2	1	17,368-17,436			AI						Loss	
MIR6859-3	1	17,368-17,436			AI						Loss	

MIR6859-4	1	17,368-17,436			AI						Loss	
MIR761	1	52,302,015-52,302,074			AI							
MIR7846	1	12,226,999-12,227,095			AI							
MKNK1	1	47,023,078-47,082,563			AI							
MKNK1-AS1	1	47,004,367-47,035,927			AI							
MMEL1	1	2,522,080-2,564,481			AI						AI, Loss	
MMP23A	1	1,568,158-1,570,027			AI						AI, Loss	
MMP23A	1	1,631,785-1,633,247			AI						AI, Loss	
MMP23B	1	1,567,559-1,570,030			AI						AI, Loss	
MOB3C	1	47,073,386-47,082,563			AI							
MORN1	1	2,252,691-2,323,190			AI						AI, Loss	
MPL	1	43,803,474-43,820,135			AI, Loss						AI	
MROH7	1	55,107,412-55,175,940			AI, Loss							
MROH7-TTC4	1	55,107,412-55,208,328			AI, Loss							
MRPS15	1	36,921,361-36,930,040			AI						AI	
MSH4	1	76,262,555-76,378,923			AI, Loss							
MTFR1L	1	26,146,396-26,159,433			AI						AI, Loss	
MTHFR	1	11,845,786-11,866,160			AI						Loss	
MTMR9LP	1	32,697,260-32,707,311			AI						AI, Loss	
MTOR	1	11,166,587-11,322,608			AI						Loss	
MTOR-AS1	1	11,203,954-11,209,595			AI						Loss	
MUL1	1	20,825,940-20,834,674			AI							
MYCBP	1	39,328,161-39,339,050			AI						AI	
MYCL	1	40,361,095-40,367,687			AI						AI	
MYCL1	1	40,361,095-40,367,687			AI						AI	
MYOM3	1	24,382,530-24,438,665			AI, Loss							
MYSM1	1	59,120,410-59,165,747			AI							
NADK	1	1,682,670-1,711,586			AI						AI, Loss	
NBPF3	1	21,766,582-21,811,393			AI							
NCMAP	1	24,882,566-24,935,818			AI						AI, Loss	
NDC1	1	54,231,133-54,304,225			AI, Loss							
NDUFS5	1	39,491,966-39,500,308			AI						AI, Gain	
NEGR1	1	71,868,624-72,748,405			AI, Loss							
NEGR1-IT1	1	72,259,914-72,302,695			AI, Loss							
NFIA	1	61,542,945-61,928,460			AI							
NFIA-AS1	1	61,714,616-61,719,190			AI							
NFIA-AS2	1	61,405,915-61,436,448			AI							
NFYC	1	41,157,241-41,237,276			AI						AI	
NFYC-AS1	1	41,154,751-41,157,933			AI						AI	
NKAIN1	1	31,652,591-31,712,734			AI						AI, Loss	
NOC2L	1	879,582-894,679			AI						Loss	
NOL9	1	6,581,406-6,614,658			AI, Loss						AI, Loss	
NPHP4	1	5,946,554-5,965,543			AI, Loss						AI, Loss	
NPHP4	1	5,922,867-6,052,533			AI, Loss						AI, Loss	

NPPA	1	11,905,766-11,907,840			AI						Loss	
NPPA-AS1	1	11,900,375-11,907,674			AI						Loss	
NPPB	1	11,917,520-11,918,992			AI						Loss	
NR0B2	1	27,237,974-27,240,567			AI						Loss	
NRD1	1	52,254,865-52,344,609			AI							
NRDC	1	52,254,865-52,344,609			AI							
NSUN4	1	46,805,848-46,830,824			AI							
NT5C1A	1	40,124,792-40,137,710			AI						AI	
NUDC	1	27,248,212-27,273,362			AI						Loss	
OMA1	1	58,939,503-59,012,471			AI							
OR11L1	1	248,004,229-248,005,198										AI
OR13G1	1	247,835,419-247,836,343										AI
OR14A16	1	247,978,101-247,979,031										AI
OR14A2	1	247,886,258-247,911,460										AI
OR14K1	1	247,901,916-247,902,861										AI
OR1C1	1	247,920,763-247,921,708										AI
OR2AJ1	1	248,097,067-248,098,057										AI
OR2AK2	1	248,128,633-248,129,641										AI
OR2L13	1	248,100,330-248,264,224										AI
OR2L1P	1	248,153,568-248,154,493										AI
OR2L2	1	248,201,473-248,202,607										AI
OR2L3	1	248,223,983-248,224,922										AI
OR2L5	1	248,185,249-248,186,188										AI
OR2L8	1	248,112,159-248,113,098										AI
OR2M1P	1	248,285,437-248,286,082										AI
OR2M5	1	248,308,449-248,309,388										AI
OR2T8	1	248,084,319-248,085,258										AI
OR2W3	1	248,058,888-248,059,833										AI
OR4F16	1	367,658-368,597			AI						Loss	
OR4F29	1	367,658-368,597			AI						Loss	
OR4F3	1	367,658-368,597			AI						Loss	
OR4F5	1	69,090-70,008			AI						Loss	
OR6F1	1	247,875,130-247,876,057										AI
ORC1	1	52,838,500-52,870,143			AI, Loss							
OSBPL9	1	52,042,850-52,254,891			AI							
OSCP1	1	36,883,506-36,916,086			AI						AI	
OXCT2	1	40,235,196-40,237,020			AI						AI	
OXCT2P1	1	39,980,537-39,982,341			AI						AI	
PABPC4	1	40,026,484-40,042,521			AI						AI	
PABPC4-AS1	1	40,030,741-40,038,875			AI						AI	
PADI1	1	17,531,620-17,572,501			AI, Loss							
PADI3	1	17,575,592-17,610,727			AI, Loss							
PADI4	1	17,634,689-17,690,495			AI, Loss							
PADI6	1	17,698,690-17,728,195			AI, Loss							

PAFAH2	1	26,286,257-26,324,648			AI						AI, Loss	
PANK4	1	2,439,969-2,458,035			AI						AI, Loss	
PAQR7	1	26,187,974-26,197,744			AI						AI, Loss	
PARK7	1	8,021,713-8,045,342			AI						AI, Loss	
PATJ	1	62,208,137-62,629,591			AI, Loss							
PAX7	1	18,957,499-19,075,360			Loss							
PCSK9	1	55,505,148-55,530,526			AI, Loss							
PDE4B	1	66,258,192-66,840,262			AI, Loss							
PDIK1L	1	26,437,655-26,452,039			AI						AI, Loss	
PDZK1IP1	1	47,649,260-47,655,771			AI							
PEF1	1	32,095,462-32,110,838			AI						AI, Loss	
PER3	1	7,844,379-7,905,240			AI, Loss						AI, Loss	
PEX10	1	2,336,240-2,344,010			AI						AI, Loss	
PEX14	1	10,535,002-10,690,815			AI						Loss	
PGM1	1	64,058,946-64,125,916			AI, Loss							
PHC2	1	33,789,223-33,896,615			AI						AI, Loss	
PIGV	1	27,114,453-27,124,894			AI						Loss	
PIK3R3	1	46,505,811-46,642,160			Loss							
PIN1P1	1	70,385,004-70,386,000			AI							
PINK1	1	20,959,947-20,978,004			AI							
PINK1-AS	1	20,969,149-20,978,686			AI							
PITHD1	1	24,104,875-24,114,722			AI							
PLA2G2C	1	20,489,584-20,503,857			AI							
PLA2G2F	1	20,465,822-20,476,879			AI, Loss							
PLCH2	1	2,398,897-2,436,969			AI						AI, Loss	
PLEKHG5	1	6,526,151-6,580,121			AI						AI, Loss	
PLEKHN1	1	901,876-910,484			AI						Loss	
PLOD1	1	11,994,723-12,035,599			AI						Loss	
PLPP3	1	56,960,418-57,045,257			AI							
PNRC2	1	24,286,300-24,289,949			AI							
PODN	1	53,527,723-53,551,174			AI							
PPAP2B	1	56,960,418-57,045,257			AI							
PPIE	1	40,204,516-40,229,586			AI						AI	
PPIEL	1	39,987,951-40,025,370			AI						AI	
PPP1R8	1	28,157,251-28,178,183			AI						Loss	
PPT1	1	40,538,381-40,563,142			AI						AI, Gain	
PRAMEF12	1	12,834,990-12,838,048			AI, Loss						Loss	
PRDM16	1	2,985,741-3,355,185			AI						AI, Loss	Loss
PRDM2	1	14,026,734-14,151,574			AI						AI, Loss	
PRKAA2	1	57,110,989-57,181,008			AI							
PRKACB	1	84,543,657-84,704,181			AI, Loss							
PRKCZ	1	1,981,908-2,116,838			AI						AI, Loss	
PRKCZ-AS1	1	2,113,232-2,115,314			AI						AI, Loss	
PRPF38A	1	52,870,218-52,883,992			AI							

PRXL2B	1	2,517,898-2,522,908			AI						AI, Loss	
PTCHD2	1	11,539,294-11,597,640			AI						Loss	
PTGER3	1	71,318,035-71,513,491			AI, Loss							
PTGFR	1	78,956,727-79,006,386			AI, Loss							
PTP4A2	1	32,372,021-32,403,988			AI						AI, Loss	
PTPRU	1	29,563,027-29,653,325			AI						AI, Loss	
PUM1	1	31,404,352-31,538,763			AI						AI, Loss	
RAB3B	1	52,373,627-52,456,436			AI							
RABGGTB	1	76,251,878-76,260,775			AI, Loss							
RAD54L	1	46,713,366-46,744,145			Loss							
RAP1GAP	1	21,922,707-21,995,873			AI							
RAVER2	1	65,210,655-65,298,914			AI							
RCAN3	1	24,828,840-24,863,510			AI						AI, Loss	
RER1	1	2,323,213-2,336,885			AI						AI, Loss	
RERE	1	8,412,463-8,877,699			AI						Loss	
RERE-AS1	1	8,484,704-8,495,076			AI						Loss	
RHBDL2	1	39,351,477-39,407,502			AI						AI	
RHCE	1	25,688,739-25,756,683			AI						AI, Loss	
RHD	1	25,598,976-25,656,936			AI						AI, Loss	
RIMS3	1	41,086,351-41,131,354			AI						AI, Gain	
RLF	1	40,627,040-40,706,593			AI						Gain	
RNF11	1	51,701,944-51,739,119			AI							
RNF19B	1	33,402,046-33,430,414			AI						AI, Loss	
RNF207	1	6,268,677-6,278,429			AI						AI, Loss	
RNF207	1	6,266,188-6,281,359			AI						AI, Loss	
RNF223	1	1,007,125-1,009,687			AI						Loss	
ROR1	1	64,239,689-64,647,179			AI							
ROR1-AS1	1	64,560,124-64,579,474			AI							
RPA2	1	28,218,035-28,241,308			AI						Loss	
RPE65	1	68,894,506-68,915,642			AI, Loss							
RPL11	1	24,018,268-24,023,325			AI							
RPL22	1	6,245,079-6,259,679			AI						AI, Loss	
RRAGC	1	39,303,868-39,340,166			AI						AI	
RSRP1	1	25,568,727-25,664,745			AI						AI, Loss	
RUNX3	1	25,226,001-25,291,612			AI						AI, Loss	
S100PBP	1	33,283,042-33,324,480			AI						AI, Loss	
SAMD11	1	860,529-879,961			AI						Loss	
SAMD13	1	84,764,048-84,816,481			AI							
SCARNA1	1	28,160,911-28,161,077			AI						Loss	
SCP2	1	53,392,900-53,517,289			AI							
SDC3	1	31,342,312-31,381,480			AI						AI, Loss	
SELENON	1	26,126,666-26,144,713			AI						AI, Loss	
SELRC1	1	53,152,013-53,164,038			AI							
SEPN1	1	26,126,666-26,144,713			AI						AI, Loss	

SERBP1	1	67,873,492-67,896,123			AI							
SERINC2	1	31,882,411-31,907,527			AI						AI, Loss	
SGIP1	1	66,999,251-67,216,822			AI, Loss							
SH2D5	1	21,046,224-21,059,330			Loss							
SH3BGRL3	1	26,606,212-26,608,013			AI						AI, Loss	
SH3D21	1	36,771,993-36,790,484			AI						AI	
SHISAL2A	1	53,099,015-53,134,792			AI							
SKI	1	2,160,133-2,241,652			AI						AI, Loss	
SLC1A7	1	53,552,850-53,608,304			AI							
SLC25A34-AS1	1	16,066,545-16,076,385			Loss						AI, Loss	
SLC25A3P1	1	53,904,042-53,905,693			AI							
SLC2A5	1	9,097,004-9,148,510			AI						Loss	
SLC2A7	1	9,063,358-9,086,404			AI						Loss	
SLC30A2	1	26,364,512-26,372,629			AI						AI, Loss	
SLC35E2	1	1,656,276-1,677,438			AI						AI, Loss	
SLC35E2A	1	1,656,276-1,677,438			AI						AI, Loss	
SLC35E2B	1	1,590,987-1,677,438			AI						AI, Loss	
SLC44A5	1	75,667,815-76,143,610			AI, Loss							
SLC45A1	1	8,378,144-8,404,227			AI						Loss	
SMAP2	1	40,839,377-40,888,998			AI						AI, Gain	
SMIM1	1	3,689,324-3,692,546			AI						AI, Loss	
SMIM12	1	35,315,962-35,325,417			AI						AI, Loss	
SMPDL3B	1	28,261,465-28,285,668			AI						Loss	
SNORA2	1	84,743,003-84,743,140			AI							
SNORA55	1	40,033,045-40,033,182			AI						AI	
SNORA59A	1	12,567,299-12,567,451			AI						Loss	
SNORA59B	1	12,567,299-12,567,451			AI						Loss	
SNORA63C	1	36,884,048-36,884,179			AI						AI	
SNORD103A	1	31,408,535-31,408,623			AI						AI, Loss	
SNORD103B	1	31,421,964-31,422,052			AI						AI, Loss	
SNORD103C	1	31,441,009-31,441,084			AI						AI, Loss	
SNORD128	1	8,554,859-8,554,973			AI						Loss	
SNORD45A	1	76,253,573-76,253,657			AI, Loss							
SNORD45B	1	76,255,161-76,255,232			AI, Loss							
SNORD45C	1	76,252,756-76,252,834			AI, Loss							
SNORD85	1	31,441,009-31,441,084			AI						AI, Loss	
SNRNP40	1	31,732,414-31,769,644			AI						AI, Loss	
SPEN	1	16,174,358-16,266,950			AI						AI, Loss	
SPOCD1	1	32,256,022-32,281,652			AI, Loss						AI, Loss	
SPSB1	1	9,352,940-9,429,590			AI						Loss	
SRARP	1	16,330,730-16,333,190			AI						AI, Loss	
SRM	1	11,114,648-11,120,091			AI						Loss	
SRRM1	1	24,969,593-24,999,772			AI						AI, Loss	
SRSF10	1	24,290,836-24,306,953			AI							

ST6GALNAC3	1	76,540,368-77,103,001			AI, Loss							
STIL	1	47,694,867-47,779,819			AI, Loss							
STK40	1	36,805,219-36,851,528			AI						AI	
STMN1	1	26,210,676-26,233,368			AI						AI, Loss	
STX12	1	28,099,693-28,150,963			AI						Loss	
SYF2	1	25,548,766-25,559,013			AI						AI, Loss	
TACSTD2	1	59,041,094-59,043,166			AI							
TAL1	1	47,681,961-47,698,007			AI							
TARDBP	1	11,072,678-11,085,549			AI						Loss	
TAS1R1	1	6,615,337-6,639,817			AI, Loss						AI, Loss	
TCEA3	1	23,707,401-23,751,272			AI, Loss							
TCEANC2	1	54,519,244-54,578,192			AI, Loss							
TCEB3	1	24,069,855-24,088,549			AI							
TCTEX1D1	1	67,218,139-67,244,730			AI							
TEKT2	1	36,549,675-36,553,876			AI						AI, Gain	
TENT5B	1	27,331,510-27,339,333			AI						Loss	
TEX38	1	47,137,496-47,139,256			AI							
TEX46	1	23,337,326-23,342,343			AI							
THEMIS2	1	28,199,053-28,213,196			AI						Loss	
THRAP3	1	36,690,012-36,770,957			AI						AI	
TIE1	1	43,766,565-43,791,552			AI, Loss						AI	
TM2D1	1	62,146,718-62,191,095			AI							
TMCO2	1	40,713,572-40,717,365			AI						Gain	
TMEM125	1	43,735,664-43,739,673			AI						AI	
TMEM234	1	32,680,077-32,687,972			AI						AI, Loss	
TMEM35B	1	35,447,126-35,450,948			Loss							
TMEM39B	1	32,537,631-32,568,467			AI						AI, Loss	
TMEM50A	1	25,664,788-25,688,852			AI						AI, Loss	
TMEM51	1	15,479,027-15,546,976			AI						AI, Loss	
TMEM51-AS1	1	15,438,310-15,478,960			AI						AI, Loss	
TMEM52	1	1,849,028-1,850,740			AI						AI, Loss	
TMEM54	1	33,360,193-33,367,041			AI						AI, Loss	
TMEM57	1	25,757,387-25,826,698			AI						AI, Loss	
TMEM59	1	54,492,353-54,519,246			Loss							
TMEM82	1	16,068,986-16,074,292			Loss						AI, Loss	
TNFRSF14	1	2,487,803-2,497,061			AI						AI, Loss	
TNFRSF14-AS1	1	2,481,358-2,488,450			AI						AI, Loss	
TNFRSF1B	1	12,227,059-12,269,277			AI						Loss	
TNFRSF25	1	6,521,213-6,526,255			AI						AI, Loss	
TNFRSF8	1	12,123,433-12,204,264			AI						Loss	
TNFRSF9	1	7,975,930-8,003,225			AI						AI, Loss	
TNNI3K	1	74,701,070-75,010,116			AI, Loss							
TP73	1	3,569,128-3,652,765			AI						AI, Loss	
TP73-AS1	1	3,652,547-3,663,937			AI						AI, Loss	

TPRG1L	1	3,541,555-3,546,694			AI						AI, Loss	
TRABD2B	1	48,226,199-48,462,562			AI, Loss							
TRIM58	1	248,020,500-248,060,142										AI
TRIM62	1	33,611,002-33,647,671			AI						AI, Loss	
TRIM63	1	26,377,795-26,394,125			AI						AI, Loss	
TRIT1	1	40,306,705-40,349,183			AI						AI	
TRNP1	1	27,320,194-27,327,377			AI						Loss	
TSSK3	1	32,827,861-32,829,924			AI						AI, Loss	
TTC34	1	2,572,707-2,717,433			AI						AI, Loss	
TTC39A	1	51,752,929-51,810,788			AI							
TTC39A-AS1	1	51,795,325-51,796,953			AI							
TTC4	1	55,181,494-55,208,328			AI, Loss							
TTLL7	1	84,330,714-84,464,870			AI, Loss							
TUT4	1	52,888,947-53,018,762			AI							
TXLNA	1	32,645,344-32,663,886			AI						AI, Loss	
TXNDC12	1	52,485,803-52,521,843			AI							
TXNDC12-AS1	1	52,516,589-52,518,355			AI							
TYW3	1	75,198,835-75,232,360			AI, Loss							
U4	1	66,560,143-66,560,229			Loss							
U6	1	23,490,445-23,490,546			AI							
U6	1	43,489,219-43,489,308			AI							
U7	1	63,799,429-63,799,491			AI							
U7	1	68,238,275-68,238,336			AI							
UBE2U	1	64,669,300-64,733,065			AI							
UBIAD1	1	11,333,254-11,359,628			AI						Loss	
UBXN10	1	20,512,570-20,522,541			AI							
UBXN10-AS1	1	20,510,735-20,512,979			AI							
UBXN11	1	26,608,772-26,644,756			AI						AI, Loss	
UOX	1	84,830,640-84,850,484			AI							
UQCRH	1	46,769,284-46,782,449			AI							
UQCRHL	1	16,133,656-16,134,194			AI						AI, Loss	
USP24	1	55,532,031-55,681,039			AI							
USP48	1	22,004,791-22,109,692			AI							
UTS2	1	7,903,142-7,973,294			AI						AI, Loss	
VPS13D	1	12,290,086-12,572,098			AI						Loss	
VWA5B1	1	20,617,411-20,681,387			AI							
WASH7P	1	14,361-29,961			AI						Loss	
WDR65	1	43,638,000-43,720,029			AI						AI	
WDR78	1	67,278,571-67,390,570			Loss							
WLS	1	68,564,141-68,698,284			AI							
WNT4	1	22,443,797-22,470,385			AI							
WRAP73	1	3,547,330-3,566,671			AI						AI, Loss	
XKR8	1	28,285,972-28,294,607			AI						Loss	
Y_RNA	1	55,950,543-55,950,645			AI							

YARS	1	33,240,839-33,283,633			AI						AI, Loss	
Z24749	1	25,169,384-25,170,807			AI						AI, Loss	
ZBTB17	1	16,268,363-16,302,627			AI						AI, Loss	
ZBTB40	1	22,778,343-22,857,650			AI							
ZCCHC11	1	52,888,947-53,019,130			AI							
ZCCHC17	1	31,769,828-31,837,800			AI						AI, Loss	
ZDHHC18	1	27,153,200-27,182,211			AI						Loss	
ZFP69	1	40,943,301-40,962,015			AI						AI, Gain	
ZFP69B	1	40,915,773-40,929,390			AI						AI, Gain	
ZFYVE9	1	52,607,765-52,812,358			AI							
ZMPSTE24	1	40,723,721-40,759,856			AI						Gain	
ZMYM6	1	35,449,367-35,497,569			Loss						Gain	
ZMYM6NB	1	35,447,126-35,450,948			Loss							
ZNF362	1	33,722,173-33,766,320			AI						AI, Loss	
ZNF436	1	23,685,940-23,696,357			AI							
ZNF436-AS1	1	23,695,463-23,698,330			AI							
ZNF593	1	26,496,387-26,497,364			AI						AI, Loss	
ZNF684	1	40,997,232-41,013,841			AI						AI, Gain	
ZRANB2	1	71,528,973-71,546,972			AI							
ZRANB2-AS1	1	71,512,188-71,532,865			AI							
ZRANB2-AS2	1	71,547,006-71,703,406			AI							
ZSCAN20	1	33,938,231-33,961,995			AI						AI, Loss	
ZZZ3	1	78,028,100-78,149,112			Loss							
ACP1	2	264,868-278,282										Loss
ALKAL2	2	279,560-288,308										Loss
ALMS1	2	73,612,885-73,837,046			Gain							
ANKMY1	2	241,418,838-241,500,527										Loss
BMPR2	2	203,241,049-203,432,474										Gain
COL6A3	2	238,232,654-238,322,850										Loss
DNAH7	2	196,602,426-196,933,536			Loss							
DUSP28	2	241,499,470-241,503,431										Loss
DYSF	2	71,680,752-71,913,893										Loss
FAM110C	2	38,813-46,588										Loss
FAM117B	2	203,499,900-203,634,480										Gain
FAM150B	2	279,560-288,308										Loss
FSIP2	2	186,603,621-186,698,016			Loss							
GPC1	2	241,375,114-241,407,495										Loss
HDAC4	2	239,969,863-240,323,346										Loss
LINC01115	2	779,836-864,112										Loss
LINC01874	2	490,946-492,655										Loss
LINC01875	2	545,804-546,667										Loss
LINC01939	2	895,901-901,137										Loss
LOC105373352	2	558,200-562,092										Loss
LOC339822	2	779,836-864,112										Loss

MARCO	2	119,699,744-119,752,236										Loss
MIR4440	2	239,990,512-239,990,610										Loss
MIR4441	2	240,007,522-240,007,622										Loss
MIR548N	2	179,246,804-179,541,009									Loss	Loss
PPIG	2	170,440,849-170,494,254			Gain							
PXDN	2	1,635,658-1,748,319										Loss
RNPEPL1	2	241,507,900-241,518,149										Loss
SH3YL1	2	218,135-264,866										Loss
SNTG2	2	946,553-1,371,384										Loss
SNTG2-AS1	2	945,320-947,078										Loss
TMEM18	2	663,877-677,468										Loss
TTN	2	179,390,716-179,672,150									Loss	Loss
TTN	2	179,504,425-179,516,263									Loss	
TTN	2	179,522,607-179,536,994									Loss	
TTN-AS1	2	179,387,553-179,484,944										Loss
ALDH1L1	3	125,822,403-125,900,029										Loss
C3orf56	3	126,911,973-126,917,027										Loss
CGGBP1	3	88,101,099-88,199,016			Gain							
CLSTN2	3	139,654,026-140,286,919										Loss
GOLGA4	3	37,284,681-37,408,370			Gain							
GOLGB1	3	121,382,045-121,468,614			Gain							
LINC00606	3	10,801,026-10,805,894										Loss
PROS1	3	93,591,880-93,692,934										Gain
RBMS3	3	29,322,802-30,051,886			Gain							
ROBO2	3	75,986,644-77,699,114			Loss							
SLC6A11	3	10,857,884-10,982,419										Loss
ZNF654	3	88,108,404-88,193,814			Gain							
BC040333	4	39,640,759-39,653,681									Gain	Gain
C4orf40	4	71,017,792-71,032,326								Gain		
DQ573667	4	10,127,121-10,127,151										Loss
DQ581767	4	10,124,402-10,124,435										Loss
DQ583133	4	10,122,826-10,122,861										Loss
DQ584082	4	10,125,235-10,125,264										Loss
DQ589421	4	10,122,105-10,122,135										Loss
DQ591515	4	10,137,431-10,137,474										Loss
DQ599898	4	10,145,243-10,145,285										Loss
FAT4	4	126,237,566-126,414,087			Gain							
MIR3138	4	10,080,234-10,080,316										Loss
PDS5A	4	39,824,482-39,979,576									Gain	Gain
PRR27	4	71,019,903-71,032,326								Gain		
PSAPL1	4	7,432,020-7,436,700									Loss	Loss
SLC2A9	4	9,827,847-10,041,872										Loss
SMIM14	4	39,548,916-39,640,618									Gain	Gain
SORCS2	4	7,194,373-7,744,564									Loss	Loss

UBE2K	4	39,699,663-39,784,410									Gain	Gain
WDR1	4	10,086,066-10,094,779										Loss
WDR1	4	10,075,962-10,118,658										Loss
AK6	5	68,647,552-68,665,840										Gain
AX747819	5	134,670,617-134,672,992										Loss
C5orf20	5	134,779,903-134,783,038										Loss
C5orf66	5	134,368,969-134,680,370										Loss
CCDC127	5	204,874-218,297										Loss
CEP72	5	612,404-653,666										Loss
DCANP1	5	134,779,903-134,783,038										Loss
DNAH5	5	13,690,436-13,944,589			Loss							
FBXL21	5	135,266,005-135,287,280										Loss
FBXL21P	5	135,265,985-135,277,685										Loss
H2AFY	5	134,670,070-134,735,577										Loss
HRAT5	5	269,670-271,639										Loss
IL9	5	135,227,934-135,231,516										Loss
IRX2	5	2,746,278-2,751,769										Loss
LECT2	5	135,282,599-135,290,723										Loss
LOC100996485	5	134,368,969-134,680,370										Loss
LRRC14B	5	191,625-196,449										Loss
LSINCT5	5	2,712,704-2,715,351										Loss
PDCD6	5	271,735-315,089										Loss
PLEKHG4B	5	92,265-190,087										Loss
RAD17	5	68,665,123-68,710,630										Gain
SDHA	5	218,337-257,197										Loss
SLC25A48	5	134,914,854-135,224,326										Loss
TAF9	5	68,647,552-68,665,840										Gain
TIFAB	5	134,784,557-134,788,089										Loss
DL491467	6	74,228,125-74,228,181										Gain
DQ571907	6	40,346,131-40,346,176										Loss
DQ590492	6	74,228,271-74,228,299										Gain
EEF1A1	6	74,225,472-74,230,755										Gain
ENPP5	6	46,126,918-46,138,747			Loss							
GOPC	6	117,639,350-117,923,705			Loss							
LINC00951	6	40,312,083-40,323,745										Loss
LOC101929460	6	169,434,265-169,435,738										Loss
LOC101929523	6	169,613,306-169,639,657										Loss
LOC105378146	6	169,327,835-169,364,477										Loss
MIR548H3	6	97,537,842-97,862,283			Loss							
MMS22L	6	97,590,036-97,731,153			Loss							
MTO1	6	74,171,453-74,211,179										Gain
ROS1	6	117,609,529-117,747,018			Loss							
SHPRH	6	146,205,944-146,285,233			Gain							
SMOC2	6	168,841,830-169,068,674										Loss

SNORD141A	6	74,227,967-74,228,161										Gain
SNORD141B	6	74,227,967-74,228,161										Gain
TDRG1	6	40,346,075-40,347,638										Loss
THBS2	6	169,615,874-169,654,209										Loss
ABCA13	7	48,211,056-48,687,091	Loss		Loss						Loss	Loss
AKAP9	7	91,570,188-91,739,987			Gain							
AX746840	7	48,313,698-48,316,321	Loss		Loss						Loss	Loss
CFTR	7	117,120,016-117,308,718			Loss							
ISPD	7	16,127,151-16,460,947			Gain							
LOC100506585	7	157,647,276-157,658,782										Loss
MEOX2	7	15,650,836-15,726,308			Gain							
PTPRN2	7	157,331,745-158,380,494										Loss
RBAK	7	5,085,451-5,109,119			Gain							
RBAK-RBAKDN	7	5,023,301-5,112,854			Gain							
SAMD9	7	92,728,825-92,747,336			Gain							
ADAM18	8	39,442,086-39,587,583			Loss							
AK056623	8	424,454-427,966										Loss
ARC	8	143,692,404-143,695,833										Loss
C8orf34	8	69,242,607-69,731,258			Gain							
ERICH1	8	564,736-681,239										Loss
JH8	8	143,739,812-143,745,841										Loss
JRK	8	143,738,873-143,751,412										Loss
LOC100132891	8	72,755,357-72,968,547			Loss							
LOC101928087	8	143,719,948-143,722,024										Loss
MIR1302-7	8	142,867,602-142,867,674										Loss
MIR4539	8	143,079,636-143,079,657										Loss
MROH5	8	142,443,928-142,517,330										Loss
MSC-AS1	8	72,755,357-72,968,547			Loss							
NPBWR1	8	53,852,467-53,853,454										AI
OR4F21	8	116,085-117,024			Gain							Loss
PKHD1L1	8	110,374,705-110,549,443			Loss							
RIMS2	8	104,512,975-105,266,656			Loss							
RPL23AP53	8	158,344-182,318			Gain							Loss
TDRP	8	439,789-495,781										Loss
TRPA1	8	72,933,485-72,987,819			Loss							
ZNF596	8	182,136-197,340			Gain							Loss
AK000451	9	66,523,813-66,553,911			Gain							
AK096159	9	68,743,530-68,769,869			Gain							
AK308561	9	66,454,656-66,457,142			Gain							
AK309896	9	66,513,679-66,553,531			Gain							
AK311167	9	69,065,545-69,106,241			Gain							
ALDOB	9	104,182,841-104,198,056			Gain							
ANKRD19P	9	95,571,892-95,650,975			Gain							
ANKRD20A1	9	67,926,760-67,969,840			Gain							

AQP7P1	9	67,254,266-67,289,492			Gain							
AX747850	9	68,795,666-68,798,213			Gain							
BC000869	9	66,553,275-66,555,595			Gain							
BC065763	9	66,688,085-66,699,951			Gain							
BC080605	9	68,413,445-68,414,929			Gain							
BMS1P14	9	67,324,218-67,340,165			Gain							
CBWD6	9	69,204,537-69,262,593			Gain							
COL5A1	9	137,533,650-137,736,688										Loss
CR627148	9	66,457,288-66,469,310			Gain							
DQ594366	9	67,792,166-67,794,649			Gain							
DQ600787	9	66,368,799-66,368,828			Gain							
FAM27B	9	67,792,928-67,794,189			Gain							
FAM27E3	9	67,784,943-67,786,625			Gain							
FCN1	9	137,795,081-137,809,849										Loss
FCN2	9	137,772,657-137,779,366										Loss
FOXD4L6	9	69,199,479-69,202,204			Gain							
FRG1HP	9	68,726,464-68,748,376			Gain							
FRG1HP	9	69,065,489-69,112,332			Gain							
FRG1JP	9	68,427,783-68,455,368			Gain							
KIF12	9	116,853,917-116,861,507										Loss
LINC00537	9	68,408,674-68,414,763			Gain							
LINC01410	9	66,457,288-66,469,310			Gain							
LOC100132249	9	66,490,071-66,494,170			Gain							
LOC100132352	9	68,726,540-68,767,301			Gain							
LOC102724580	9	67,290,450-67,334,405			Gain							
LOC105379807	9	67,785,633-67,786,685			Gain							
LOC286297	9	67,017,374-67,032,072			Gain							
LOC403323	9	66,513,679-66,553,911			Gain							
LOC440896	9	69,174,195-69,198,043			Gain							
LOC642236	9	68,427,782-68,454,375			Gain							
LOC728673	9	66,553,199-66,555,601			Gain							
MIR1299	9	69,002,238-69,002,321			Gain							
MIR4477A	9	68,415,307-68,415,388			Gain							
MIR4477B	9	68,415,307-68,415,388			Gain							
MRPL50	9	104,151,121-104,160,919			Gain							
PGM5P2	9	69,080,243-69,147,854			Gain							
PTGER4P2-CDK2AP2P2	9	66,494,268-66,503,030			Gain							
SH3GL2	9	17,578,951-17,797,126									AI	
X97876	9	66,499,190-66,500,030			Gain							
ZNF189	9	104,161,135-104,172,942			Gain							
ZNF484	9	95,606,477-95,640,320			Gain							
ADARB2	10	1,223,252-1,779,670										Loss
AK057316	10	47,658,233-47,701,017	AI	AI	AI	AI	AI					
AK124121	10	94,178,242-94,180,347										Gain

ANTXRL	10	47,658,232-47,701,443	AI	AI	AI	AI	AI					
ANTXRLP1	10	47,590,260-47,640,844	AI	AI	AI	AI	AI					
AX747983	10	80,760,240-80,763,560										Loss
BICC1	10	60,272,903-60,591,197			Gain							
C10orf71	10	50,507,186-50,535,537										Loss
EBF3	10	131,633,495-131,762,091										Loss
IDE	10	94,211,440-94,333,852										Gain
LIPJ	10	90,346,518-90,366,733			Loss							
MARK2P9	10	94,178,417-94,179,363										Gain
MGMT	10	131,265,504-131,569,247										Loss
MIR4297	10	131,641,562-131,641,638										Loss
SLC25A16	10	70,242,089-70,287,280										Gain
ZMIZ1-AS1	10	80,703,082-80,828,536										Loss
AK130852	11	134,855,245-134,856,693										Loss
ANKK1	11	113,258,512-113,271,140										Loss
KCNQ1	11	2,466,220-2,870,340										Loss
KCNQ1OT1	11	2,629,557-2,721,228										Loss
LOC100507548	11	134,855,245-134,856,693										Loss
MMP1	11	102,660,640-102,668,966			Loss							
WTAPP1	11	102,654,406-102,707,497			Loss							
ADGRD1	12	131,438,451-131,626,013										Loss
AMN1	12	31,824,070-31,882,108										Gain
BC042649	12	131,780,655-131,782,517										Loss
ETFBKMT	12	31,800,093-31,822,016										Gain
GPR133	12	131,438,451-131,626,008										Loss
H3F3C	12	31,944,118-31,945,175										Gain
KIAA1551	12	32,112,352-32,146,043			Gain							
KRT74	12	52,959,602-52,967,609									Loss	Loss
KRT81	12	52,679,696-52,702,155										Loss
KRT82	12	52,787,734-52,800,176										Loss
KRT83	12	52,708,084-52,715,182										Loss
KRT84	12	52,771,595-52,779,417										Loss
KRT85	12	52,753,789-52,761,309										Loss
KRT86	12	52,643,083-52,702,947										Loss
LINC01257	12	131,649,555-131,697,476										Loss
LINC02370	12	131,832,014-131,852,100										Loss
LINC02415	12	131,780,657-131,782,517										Loss
LOC116437	12	131,649,555-131,697,476										Loss
LOC338797	12	131,832,014-131,852,100										Loss
METTL20	12	31,800,093-31,822,016										Gain
OTOGL	12	80,603,232-80,772,870			Loss							
RESF1	12	32,112,352-32,146,043			Gain							
SLCO1B1	12	21,284,127-21,392,730			Loss							
SLCO1B3	12	20,963,637-21,243,040			Loss							

SLCO1B7	12	20,968,607-21,243,040			Loss							
AK022914	14	19,650,031-19,692,916										AI
AK056135	14	19,919,491-19,925,333										AI
AK093552	14	22,849,082-22,951,948	Gain	Gain	Gain	Gain	Gain	Gain	Gain	Gain	Gain	Gain
AK125397	14	22,886,527-22,901,726	Gain	Gain	Gain	Gain	Gain	Gain	Gain	Gain	Gain	Gain
AV2S1A1	14	22,356,036-22,745,911	Gain	Gain	Gain	Gain	Gain	Gain	Gain	Gain	Gain	Gain
AV4S1	14	22,670,486-22,981,890	Gain	Gain	Gain	Gain	Gain	Gain	Gain	Gain	Gain	Gain
AX746996	14	104,975,961-104,978,198										Loss
BAZ1A	14	35,221,936-35,344,853									Gain	Gain
BC016035	14	19,650,031-19,719,035										AI
BC017398	14	19,911,927-19,913,315										AI
BC041856	14	19,880,208-19,925,333										AI
BC148240	14	101,540,363-101,540,920										Loss
BMS1P17	14	19,894,368-19,904,572										AI
BMS1P18	14	19,670,791-19,681,016										AI
BMS1P22	14	19,670,791-19,686,002										AI
BX248778	14	19,650,031-19,684,290										AI
C14orf39	14	60,902,673-60,952,764			Loss							
DQ573684	14	19,972,600-19,972,632										AI
DQ582260	14	19,597,269-19,597,306										AI
DQ582484	14	19,976,262-19,976,303										AI
DQ590589	14	19,596,015-19,596,045										AI
DQ591735	14	19,596,078-19,596,110										AI
DQ595048	14	19,598,183-19,598,223										AI
DQ595091	14	19,595,543-19,595,584										AI
DQ599717	14	19,596,711-19,596,739										AI
DQ786293	14	19,662,047-19,663,434										AI
DUXAP10	14	19,650,031-19,692,916										AI
DUXAP9	14	19,650,036-19,684,290										AI
DUXAP9	14	19,880,208-19,925,329										AI
hADV29S1	14	22,631,190-23,000,088	Gain	Gain	Gain	Gain	Gain	Gain	Gain	Gain	Gain	Gain
hADV36S1	14	22,694,611-23,016,719	Gain	Gain	Gain	Gain	Gain	Gain	Gain	Gain	Gain	Gain
hADV38S2	14	22,748,987-22,962,976	Gain	Gain	Gain	Gain	Gain	Gain	Gain	Gain	Gain	Gain
hDV102S1	14	22,891,471-22,892,033	Gain	Gain	Gain	Gain	Gain	Gain	Gain	Gain	Gain	Gain
hDV103S1	14	22,938,034-22,938,658	Gain	Gain	Gain	Gain	Gain	Gain	Gain	Gain	Gain	Gain
LINC00516	14	19,894,368-19,904,572										AI
LINC02285	14	101,587,192-101,589,882										Loss
LINC02297	14	19,407,014-19,410,111										AI
LOC100508046	14	20,006,912-20,010,956										AI
LOC101101776	14	19,670,791-19,681,016										AI
LOC101929572	14	19,563,121-19,566,680										AI
LOC105370401	14	22,847,807-22,951,948	Gain	Gain	Gain	Gain	Gain	Gain	Gain	Gain	Gain	Gain
LOC642426	14	19,407,014-19,410,111										AI
MEG9	14	101,536,247-101,539,273										Loss

OR11H12	14	19,377,593-19,378,574										AI
OR11H2	14	20,181,062-20,182,491										AI
OR4K1	14	20,403,766-20,404,842										AI
OR4K15	14	20,443,677-20,444,724										AI
OR4K2	14	20,344,426-20,345,371										AI
OR4K3	14	20,336,355-20,337,332										AI
OR4K5	14	20,388,765-20,389,737										AI
OR4M1	14	20,248,481-20,249,423										AI
OR4N2	14	20,271,928-20,296,531										AI
OR4Q3	14	20,215,586-20,216,528										AI
P712P	14	19,562,690-19,566,731										AI
P775P	14	19,605,327-19,639,485										AI
POTEG	14	19,553,364-19,584,942										AI
POTEH-AS1	14	19,563,711-19,566,680										AI
POTEM	14	19,983,953-20,020,272										AI
TCR	14	22,739,820-22,740,442	Gain	Gain	Gain	Gain	Gain	Gain	Gain	Gain	Gain	Gain
TCRA	14	22,293,655-23,021,097	Gain	Gain	Gain	Gain	Gain	Gain	Gain	Gain	Gain	Gain
TCRA	14	22,314,718-22,534,581	Gain	Gain	Gain	Gain	Gain	Gain	Gain	Gain		Gain
TCRA	14	22,538,905-22,539,467	Gain	Gain	Gain	Gain	Gain	Gain	Gain	Gain		Gain
TCRA	14	22,554,668-22,555,238	Gain	Gain	Gain	Gain	Gain	Gain	Gain	Gain		Gain
TCRA	14	22,600,965-22,600,989	Gain	Gain	Gain	Gain	Gain	Gain	Gain	Gain		Gain
TCRA	14	22,636,292-22,636,879	Gain	Gain	Gain	Gain	Gain	Gain	Gain	Gain		Gain
TCRA	14	22,392,580-22,392,828	Gain	Gain	Gain	Gain	Gain	Gain	Gain	Gain		
TCRDV2	14	22,928,088-22,934,912	Gain	Gain	Gain	Gain	Gain	Gain	Gain	Gain	Gain	Gain
TCRDV2J2	14	22,918,106-22,925,700	Gain	Gain	Gain	Gain	Gain	Gain	Gain	Gain	Gain	Gain
TRA	14	22,458,652-23,016,719	Gain	Gain	Gain	Gain	Gain	Gain	Gain	Gain	Gain	Gain
TRA	14	22,192,007-22,447,356	Gain	Gain	Gain	Gain	Gain	Gain	Gain	Gain		
TRA@	14	22,615,959-23,021,097	Gain	Gain	Gain	Gain	Gain	Gain	Gain	Gain	Gain	Gain
TRA@	14	22,362,569-22,534,581	Gain	Gain	Gain	Gain	Gain	Gain	Gain	Gain		Gain
TRA@	14	22,591,337-22,592,252	Gain	Gain	Gain	Gain	Gain	Gain	Gain	Gain		Gain
TRAC	14	22,466,122-23,021,097	Gain	Gain	Gain	Gain	Gain	Gain	Gain	Gain	Gain	Gain
TRD	14	22,465,824-23,016,719	Gain	Gain	Gain	Gain	Gain	Gain	Gain	Gain	Gain	Gain
TRDC	14	22,919,080-22,934,912	Gain	Gain	Gain	Gain	Gain	Gain	Gain	Gain	Gain	Gain
X61074	14	22,946,618-22,948,187	Gain	Gain	Gain	Gain	Gain	Gain	Gain	Gain	Gain	Gain
BC107108	15	20,362,687-20,364,420										Gain
CHEK2P2	15	20,487,996-20,496,811										Gain
DQ571479	15	20,327,817-20,327,845										Gain
DQ576041	15	20,319,594-20,319,625										Gain
DQ582073	15	20,739,311-20,739,342										Gain
DQ582939	15	20,771,429-20,771,455										Gain
DQ592463	15	20,446,055-20,446,082										Gain
DQ594309	15	20,742,234-20,742,263										Gain
DQ595648	15	20,744,538-20,744,567										Gain
DQ600342	15	20,770,228-20,770,259										Gain

GOLGA6L6	15	20,737,093-20,747,114										Gain
GOLGA8CP	15	20,767,673-20,781,026										Gain
HERC2P3	15	20,588,367-20,711,433										Gain
HERC2P7	15	20,723,928-20,727,150										Gain
7SK	17	35,038,648-35,038,961									AI	
AA06	17	31,856,805-31,860,779									AI	
AATF	17	35,306,174-35,414,171									AI	
ACACA	17	35,441,926-35,766,902									AI, Gain	
ACBD4	17	43,209,966-43,221,548									Loss	
ACLY	17	40,023,169-40,075,275									AI	
ADAM11	17	42,836,546-42,859,214									Loss	
AK024535	17	40,465,341-40,466,880									AI	
AK057317	17	31,890,356-31,899,364									AI	
AK057897	17	43,717,727-43,719,669									Loss	
AK090604	17	39,675,603-39,678,026									AI	
AK092965	17	40,466,889-40,468,869									AI	
AK124465	17	42,982,992-42,985,615									Loss	
AK307275	17	30,814,813-30,816,538									AI	
AP2B1	17	33,914,281-34,053,436									AI, Gain	
APPBP2	17	58,520,509-58,603,601										Gain
ARGFXP2	17	30,477,386-30,478,590									AI, Gain	
ARHGAP27	17	43,471,267-43,510,282									Loss	
ARL17	17	45,110,625-45,137,515									Loss	
ARL17A	17	43,580,624-43,623,697									Loss	
ARL17A	17	44,363,861-44,439,163									Loss	
ARL17A	17	44,586,046-44,657,088									Loss	
ARL17A	17	45,108,951-45,120,079									Loss	
ARL17A	17	45,125,774-45,126,718									Loss	
ARL17B	17	43,580,624-43,581,003									Loss	
ARL17B	17	44,351,549-44,439,153									Loss	
ARL17B	17	44,624,889-44,657,078									Loss	
ARL5C	17	37,313,146-37,323,318									Gain	
ASIC2	17	31,340,105-32,483,825									AI, Loss	
AX747150	17	43,590,733-43,593,064									Loss	
AX747639	17	34,575,459-34,577,063									AI	
AX747793	17	43,484,898-43,487,751									Loss	
AX748120	17	45,400,563-45,401,497									Loss	
BC030591	17	34,136,485-34,138,588									AI	
BC043620	17	40,704,453-40,706,728									Gain	
BC045769	17	43,295,636-43,299,132									Loss	
BC047651	17	40,894,171-40,895,176									Gain	
BC062794	17	32,496,394-32,510,651									AI, Loss	
BC070118	17	34,312,625-34,317,306									AI	
BC084573	17	35,289,408-35,293,921									AI	

C17orf102	17	32,901,141-32,906,388									AI, Loss	
C17orf104	17	42,733,761-42,755,658									Loss	
C17orf113	17	40,191,722-40,202,617									AI	
C17orf50	17	34,087,915-34,092,098									AI	
C17orf66	17	34,181,959-34,195,895									AI	
C17orf75	17	30,655,459-30,669,228									Gain	
C17orf78	17	35,732,927-35,749,662									Gain	
C1QL1	17	43,037,060-43,045,644									Loss	
CACNB1	17	37,329,708-37,353,956									AI, Gain	
CASC3	17	38,296,506-38,328,431									Gain	
CAVIN1	17	40,554,466-40,575,338									AI, Gain	
CCDC103	17	42,977,079-42,981,047									Loss	
CCDC43	17	42,754,804-42,767,165									Loss	
CCL1	17	32,687,346-32,690,252									AI, Loss	
CCL11	17	32,612,686-32,615,199									AI, Loss	
CCL13	17	32,683,470-32,685,629									AI, Loss	
CCL14	17	34,310,691-34,313,764									AI	
CCL15	17	34,324,617-34,329,084									AI	
CCL15-CCL14	17	34,310,691-34,329,084									AI	
CCL16	17	34,303,528-34,308,532									AI	
CCL18	17	34,391,631-34,398,841									AI	
CCL2	17	32,582,295-32,584,220									AI, Loss	
CCL23	17	34,340,095-34,345,005									AI	
CCL3	17	34,415,602-34,417,506									AI	
CCL3L1	17	34,623,841-34,625,731									AI	
CCL3L3	17	34,522,267-34,524,147									AI	
CCL4	17	34,431,219-34,433,014									AI	
CCL4L1	17	34,538,309-34,540,280									AI	
CCL4L1	17	34,639,875-34,641,846									AI	
CCL4L2	17	34,538,464-34,540,280									AI	
CCL4L2	17	34,640,030-34,641,846									AI	
CCL5	17	34,198,495-34,207,377									AI	
CCL7	17	32,597,234-32,599,261									AI, Loss	
CCL8	17	32,646,065-32,648,421									AI, Loss	
CCR7	17	38,710,021-38,721,736									Loss	
CCT6B	17	33,254,877-33,288,528									AI	
CDC27	17	45,195,062-45,266,705									Loss	
CDK12	17	37,617,738-37,690,818									AI, Gain	
CDK5R1	17	30,814,104-30,818,271									AI	
CNP	17	40,118,758-40,129,758									AI	
COASY	17	40,714,091-40,718,299									Gain	
COPRS	17	30,178,883-30,186,326									AI, Gain	
CRHR1	17	43,697,709-43,913,194									Loss	
CRHR1-IT1	17	43,716,340-43,723,595									Loss	

CSF3	17	38,171,613-38,174,066									AI	
D63785	17	34,610,748-34,611,354									AI	
DBF4B	17	42,785,975-42,829,636									Loss	
DCAKD	17	43,100,705-43,138,497									Loss	
DHRS11	17	34,948,225-34,957,233									AI	
DHX58	17	40,253,421-40,264,751									AI	
DKFZp586B1922	17	38,285,502-38,286,560									Gain	
DNAJC7	17	40,128,438-40,169,715									AI	
DQ573965	17	34,498,252-34,498,289									AI	
DQ574804	17	34,497,545-34,497,574									AI	
DQ574805	17	34,585,589-34,585,618									AI	
DQ575686	17	34,499,866-34,499,925									AI	
DQ578599	17	43,596,759-43,596,794									Loss	
DQ579850	17	34,591,424-34,591,451									AI	
DQ580766	17	34,578,079-34,578,960									AI	
DQ581677	17	40,120,393-40,120,546									AI	
DQ588732	17	43,593,072-43,593,494									Loss	
DQ592711	17	34,242,761-34,242,791									AI	
DQ593188	17	34,499,312-34,499,341									AI	
DQ597730	17	45,126,237-45,127,789									Loss	
EFCAB13	17	45,401,141-45,518,677									Loss	
EFTUD2	17	42,927,654-42,976,993									Loss	
EIF1	17	39,845,126-39,847,898									AI	
ERBB2	17	37,844,336-37,884,915									AI	
EZH1	17	40,852,292-40,897,114									Gain	
FAM187A	17	42,980,126-42,982,758									Loss	
FBXL20	17	37,408,896-37,557,909									AI, Gain	
FBXO47	17	37,092,684-37,123,655									AI, Gain	
FKBP10	17	39,968,961-39,979,469									AI	
FMNL1	17	43,299,291-43,324,686									Loss	
FNDC8	17	33,448,597-33,457,751									AI	
FOXK2	17	80,477,593-80,562,483			Gain							
FZD2	17	42,634,811-42,638,630									Gain	
GAS2L2	17	34,071,529-34,079,897									AI	
GAST	17	39,868,577-39,872,221									AI	
GFAP	17	42,982,993-42,992,920									Loss	
GGNBP2	17	34,900,736-34,946,276									AI	
GHDC	17	40,341,104-40,346,550									AI	
GJC1	17	42,875,815-42,908,179									Loss	
GJD3	17	38,516,904-38,520,945									Loss	
GOSR2	17	45,000,482-45,104,994									Loss	
GPATCH8	17	42,472,644-42,580,970									AI, Gain	
GRB7	17	37,894,153-37,903,538									AI	
GSDMA	17	38,119,225-38,134,019									AI	

GSDMB	17	38,060,847-38,074,903									AI	
HAP1	17	39,878,890-39,890,898									AI	
HCRT	17	40,336,077-40,337,470									AI	
HEATR9	17	34,181,949-34,195,895									AI	
HEXIM1	17	43,224,683-43,229,468									Loss	
HEXIM2	17	43,237,540-43,248,852									Loss	
HIGD1B	17	42,923,720-42,927,848									Loss	
HSD17B1	17	40,703,983-40,707,232									Gain	
HSPB9	17	40,274,755-40,275,371									AI	
IGFBP4	17	38,599,675-38,613,982									Loss	
IKZF3	17	37,913,967-38,020,441									AI	
ITGB3	17	45,331,207-45,421,658									Loss	
JA429848	17	43,550,252-43,550,274									Loss	
JB074907	17	30,318,433-30,318,456									AI, Gain	
JUP	17	39,679,868-39,943,020									AI	
KANSL1	17	44,107,281-44,302,740									Loss	
KANSL1-AS1	17	44,270,938-44,274,089									Loss	
KAT2A	17	40,265,128-40,273,382									AI	
KCNH4	17	40,308,909-40,333,296									AI	
KIF18B	17	43,002,078-43,025,079									Loss	
KLHL10	17	39,991,936-40,004,615									AI	
KLHL11	17	40,004,770-40,021,684									AI	
KRT10	17	38,974,368-38,978,863									Loss	
KRT12	17	39,017,429-39,023,462									Loss	
KRT13	17	39,657,232-39,661,865									AI	
KRT14	17	39,738,530-39,743,147									AI	
KRT15	17	39,669,996-39,678,026									AI	
KRT16	17	39,766,030-39,769,079									AI	
KRT17	17	39,775,691-39,780,882									AI	
KRT19	17	39,679,868-39,684,641									AI	
KRT20	17	39,032,140-39,041,495									Loss	
KRT222	17	38,811,871-38,821,416									Loss	
KRT23	17	39,078,947-39,093,895									AI	
KRT24	17	38,854,242-38,860,002									Loss	
KRT25	17	38,904,272-38,911,584									Loss	
KRT26	17	38,922,489-38,928,411									Loss	
KRT27	17	38,933,059-38,938,786									Loss	
KRT28	17	38,948,447-38,956,211									Loss	
KRT31	17	39,549,976-39,553,844									AI	
KRT32	17	39,615,764-39,623,638									AI	
KRT33A	17	39,502,344-39,507,064									AI	
KRT33B	17	39,519,745-39,526,052									AI	
KRT34	17	39,533,920-39,538,636									AI	
KRT35	17	39,632,940-39,637,392									AI	

KRT36	17	39,642,387-39,646,116								AI	
KRT37	17	39,576,808-39,580,822								AI	
KRT38	17	39,592,620-39,597,596								AI	
KRT39	17	39,114,668-39,123,144								AI	
KRT40	17	39,133,967-39,143,387								AI	
KRT42P	17	39,782,578-39,796,451								AI	
KRT9	17	39,722,093-39,728,310								AI	
KRTAP1-1	17	39,196,810-39,197,713								AI	
KRTAP1-3	17	39,190,136-39,191,107								AI	
KRTAP1-4	17	39,185,928-39,186,377								AI	
KRTAP1-5	17	39,182,278-39,183,454								AI	
KRTAP16-1	17	39,463,951-39,465,505								AI	
KRTAP17-1	17	39,471,168-39,471,947								AI	
KRTAP2-1	17	39,202,795-39,203,568								AI	
KRTAP2-2	17	39,210,750-39,211,463								AI	
KRTAP2-3	17	39,215,492-39,216,344								AI	
KRTAP2-4	17	39,221,367-39,222,131								AI	
KRTAP29-1	17	39,458,077-39,459,103								AI	
KRTAP3-1	17	39,164,773-39,165,366								AI	
KRTAP3-2	17	39,155,444-39,156,138								AI	
KRTAP3-3	17	39,149,681-39,150,385								AI	
KRTAP4-1	17	39,340,351-39,363,126								AI	
KRTAP4-11	17	39,273,433-39,274,606								AI	
KRTAP4-12	17	39,279,344-39,280,419								AI	
KRTAP4-2	17	39,333,697-39,334,460								AI	
KRTAP4-3	17	39,323,482-39,324,424								AI	
KRTAP4-4	17	39,315,905-39,316,983								AI	
KRTAP4-5	17	39,305,175-39,306,054								AI	
KRTAP4-6	17	39,295,684-39,296,739								AI	
KRTAP4-7	17	39,240,458-39,241,396								AI	
KRTAP4-8	17	39,253,233-39,274,600								AI	
KRTAP4-9	17	39,261,640-39,262,740								AI	
KRTAP9-1	17	39,346,138-39,346,891								AI	
KRTAP9-2	17	39,382,899-39,383,904								AI	
KRTAP9-3	17	39,388,714-39,389,706								AI	
KRTAP9-4	17	39,405,938-39,406,905								AI	
KRTAP9-6	17	39,421,629-39,422,112								AI	
KRTAP9-7	17	39,431,949-39,432,459								AI	
KRTAP9-8	17	39,394,269-39,395,256								AI	
KRTAP9-9	17	39,388,751-39,412,616								AI	
LASP1	17	37,026,111-37,078,023								AI, Gain	
LEPREL4	17	39,958,204-39,968,855								AI	
LHX1	17	35,294,771-35,301,915								AI	
LHX1-DT	17	35,218,934-35,293,960								AI	

LIG3	17	33,307,516-33,332,088									AI	
LINC00672	17	37,081,420-37,085,637									AI, Gain	
LINC00974	17	39,705,857-39,710,747									AI	
LINC01180	17	42,723,731-42,727,717									Loss	
LINC01989	17	32,496,394-32,510,651									AI, Loss	
LINC02079	17	37,182,967-37,183,363									AI, Gain	
LINC02210	17	43,697,694-43,723,597									Loss	
LINC02210-CRHR1	17	43,697,694-43,913,194									Loss	
LOC100131347	17	37,213,271-37,264,608									AI, Gain	
LOC100505782	17	39,558,667-39,568,840									AI	
LOC100996750	17	39,240,418-39,262,740									AI	
LOC105371730	17	30,468,244-30,470,154									AI, Gain	
LOC105371795	17	43,246,171-43,248,852									Loss	
LOC108783654	17	40,704,453-40,706,820									Gain	
LOC147093	17	39,705,857-39,710,747									AI	
LOC339192	17	43,268,297-43,299,132									Loss	
LOC51326	17	43,623,170-43,623,702									Loss	
LOC51326	17	45,098,945-45,099,089									Loss	
LOC644172	17	43,677,490-43,679,748									Loss	
LRRC37	17	43,592,714-43,625,261									Loss	
LRRc37A	17	43,627,369-43,664,137									Loss	
LRRc37A	17	44,372,496-44,415,160									Loss	
LRRC37A11P	17	37,186,158-37,209,458									AI, Gain	
LRRC37A2	17	44,590,075-44,633,014									Loss	
LRRC37A3	17	43,592,714-43,625,261									Loss	
LRRC37A4P	17	43,578,683-43,597,889									Loss	
LRRC37A8P	17	34,231,223-34,238,027									AI	
LRRC37B	17	30,334,890-30,380,519									AI, Gain	
LRRC3C	17	38,097,726-38,100,987									AI	
LYZL6	17	34,261,521-34,270,714									AI	
MAP3K14	17	43,340,485-43,394,386									Loss	
MAP3K14-AS1	17	43,325,291-43,345,997									Loss	
MAPK8IP1P2	17	43,677,468-43,679,228									Loss	
MAPT	17	43,971,747-44,105,699									Loss	
MAPT-AS1	17	43,920,721-43,972,879									Loss	
MAPT-IT1	17	43,973,148-43,976,164									Loss	
MED1	17	37,560,537-37,607,527									Gain	
MED24	17	38,175,349-38,210,995									AI	
MEIOC	17	42,733,761-42,753,165									Loss	
MGC57346	17	43,697,709-43,715,329									Loss	
MIEN1	17	37,884,752-37,886,816									AI	
mir-108-1	17	29,902,442-29,902,532									AI, Gain	
MIR193A	17	29,887,014-29,887,102									AI, Gain	
MIR2909	17	35,391,041-35,391,110									AI	

MIR365B	17	29,902,429-29,902,540									AI, Gain	
MIR4315-1	17	43,552,728-43,552,801									Loss	
MIR4724	17	29,861,900-29,861,989									AI	
MIR4725	17	29,902,287-29,902,377									AI, Gain	
MIR4728	17	37,882,747-37,882,814									AI	
MIR5089	17	45,050,382-45,050,466									Loss	
MIR632	17	30,677,127-30,677,221									Gain	
MIR6510	17	39,673,415-39,673,469									AI	
MIR6779	17	37,071,231-37,071,295									AI, Gain	
MIR6783	17	43,011,985-43,012,049									Loss	
MIR6784	17	43,191,734-43,191,801									Loss	
MIR6884	17	38,182,584-38,182,662									AI	
MMP28	17	34,083,267-34,122,711									AI	
MRM1	17	34,958,024-34,965,407									AI	
MSL1	17	38,278,223-38,293,170									Gain	
MYO19	17	34,851,598-34,891,305									AI	
MYO1D	17	30,819,539-31,204,191									AI	
NAGLU	17	40,687,950-40,696,466									Gain	
NEUROD2	17	37,760,020-37,764,175									AI	
NKIRAS2	17	40,169,593-40,177,656									AI	
NLE1	17	33,458,340-33,469,334									AI	
NMT1	17	43,138,666-43,186,382									Loss	
NR1D1	17	38,249,036-38,256,978									AI, Gain	
NSF	17	44,668,034-44,834,828									Loss	
NSFP1	17	44,668,099-44,718,040									Loss	
NT5C3B	17	39,981,333-39,992,523									AI	
ORMDL3	17	38,077,293-38,084,057									AI	
P3H4	17	39,958,204-39,968,451									AI	
PGAP3	17	37,827,374-37,844,323									AI	
PIGW	17	34,890,588-34,895,152									AI	
PLCD3	17	43,189,007-43,209,900									Loss	
PLEKHM1	17	43,513,265-43,568,146									Loss	
PLXDC1	17	37,219,555-37,309,485									AI, Gain	
PNMT	17	37,824,233-37,826,728									AI	
PPP1R1B	17	37,783,176-37,792,878									AI	
PSMD11	17	30,771,480-30,810,337									AI, Gain	
PSMD3	17	38,137,020-38,154,213									AI	
PTRF	17	40,554,466-40,575,338									AI, Gain	
RAB11FIP4	17	29,718,641-29,865,236									AI	
RAB5C	17	40,276,993-40,307,062									AI	
RAD51D	17	33,426,810-33,448,541									AI	
RAD51L3-RFFL	17	33,336,130-33,448,541									AI	
RAMP2	17	40,913,211-40,915,060									Gain	
RAMP2-AS1	17	40,905,946-40,913,275									Gain	

RARA	17	38,465,422-38,566,941									Gain, Loss	
RARA-AS1	17	38,497,118-38,499,388									Loss	
RASL10B	17	34,058,678-34,070,540									AI	
RDM1	17	34,245,084-34,257,780									AI	
RDM1P5	17	37,213,271-37,237,704									AI, Gain	
RFFL	17	33,336,130-33,416,348									AI	
RHOT1	17	30,469,472-30,552,790									AI, Gain	
RPL19	17	37,356,535-37,360,985									AI, Gain	
RPRML	17	45,055,521-45,056,614									Loss	
SH3GL1P1	17	30,367,354-30,369,851									AI, Gain	
SLC35G3	17	33,519,538-33,521,412									AI	
SLFN11	17	33,677,328-33,700,720									AI	
SLFN12	17	33,737,940-33,760,195									AI	
SLFN12L	17	33,801,941-33,864,880									AI	
SLFN13	17	33,762,114-33,775,856									AI	
SLFN5	17	33,570,054-33,600,673									AI	
SMARCE1	17	38,783,975-38,804,103									Loss	
SNORA90	17	35,601,716-35,601,891									AI	
SNORD124	17	38,183,795-38,183,898									AI	
SPACA3	17	31,318,881-31,324,895									AI	
SPATA32	17	43,331,759-43,339,479									Loss	
SPPL2C	17	43,922,255-43,924,438									Loss	
STAC2	17	37,366,788-37,382,125									AI, Gain	
STARD3	17	37,793,332-37,820,454									AI	
STAT3	17	40,465,342-40,540,513									AI	
STAT5A	17	40,439,564-40,463,960									AI	
STAT5B	17	40,351,194-40,428,424									AI	
STH	17	44,076,615-44,077,060									Loss	
SUZ12	17	30,264,028-30,328,064									AI, Gain	
TADA2A	17	35,766,976-35,837,226									Gain	
TAF15	17	34,136,458-34,174,246									AI	
TBC1D3B	17	34,797,171-34,808,092									AI	
TBC1D3G	17	34,581,084-34,808,103									AI	
TBC1D3G	17	36,284,016-36,294,915									AI	
TBC1D3H	17	34,745,935-34,806,015									AI	
TBC1D3H	17	36,337,654-36,348,623									AI	
TBC1D3P2	17	60,342,066-60,353,016										Gain
TCAP	17	37,821,598-37,822,807									AI	
THCAT158	17	45,380,811-45,401,267									Loss	
THRA	17	38,218,162-38,250,120									AI	
THRA1	17	38,249,026-38,250,547									AI	
TMEM132E	17	32,907,767-32,966,337									AI, Loss	
TMEM98	17	31,254,927-31,268,667									AI	
TMEM99	17	38,975,343-38,992,526									Loss	

TNS4	17	38,632,079-38,657,854									Loss	
TOP2A	17	38,544,772-38,574,202									Loss	
TTC25	17	40,086,846-40,118,535									AI	
UNC45B	17	33,474,835-33,516,364									AI	
UTP6	17	30,190,189-30,228,729									AI, Gain	
VPS25	17	40,925,453-40,931,618									Gain	
WNT3	17	44,839,871-44,896,126									Loss	
WNT9B	17	44,928,951-44,964,096									Loss	
Y_RNA	17	43,148,809-43,148,911									Loss	
ZNF207	17	30,677,156-30,707,975									Gain	
ZNF385C	17	40,177,593-40,190,044									AI	
ZNF830	17	33,288,548-33,290,205									AI	
ZNHIT3	17	34,842,470-34,855,154									AI	
ZPBP2	17	38,024,454-38,034,149									AI	
ANKRD12	18	9,136,750-9,285,983			Gain							
ROCK1	18	18,529,702-18,691,812									Gain	Gain
AK075337	19	28,129,390-28,137,384			Gain		Gain					
AK128361	19	52,494,585-52,496,450			Gain							
ZNF615	19	52,494,586-52,511,483			Gain							
ACSS1	20	24,986,865-25,038,818										Loss
AK097866	20	60,292,561-60,294,804										Loss
APMAP	20	24,943,579-24,973,425										Loss
CDH4	20	59,827,481-60,515,673										Loss
CST1	20	23,728,189-23,731,574										Loss
CST4	20	23,666,276-23,669,662										Loss
FLJ33581	20	24,180,402-24,205,224										Loss
LINC01440	20	54,039,580-54,043,329									Loss	
LINC01441	20	54,036,873-54,043,735									Loss	
LINC01721	20	24,180,402-24,205,224										Loss
LOC100128310	20	60,292,561-60,294,804										Loss
PHACTR3	20	58,152,563-58,422,766										Loss
SYCP2	20	58,438,611-58,508,718										Loss
SEPT3	22	42,365,441-42,394,225	AI, Loss	Loss	AI, Loss	AI, Loss	AI, Loss	Loss	AI, Loss	AI, Loss	Loss	
SEPT5	22	19,701,986-19,710,845	AI, Loss	AI, Loss		AI, Loss		AI	AI	AI, Loss		
7SK	22	20,455,668-20,455,969	Loss	Loss		AI, Loss	Gain	AI	AI	AI, Loss		
7SK	22	21,743,479-21,743,780	Loss	Loss		AI, Loss		AI, Loss	AI	AI, Loss		
AB372727	22	50,862,771-50,862,865			AI	AI, Loss	AI, Loss		AI			
ACR	22	51,176,651-51,183,727	Loss	Loss	Loss	Loss	AI, Loss		AI		Loss	
ADA2	22	17,659,679-17,702,744	AI, Loss	AI, Loss		AI, Loss		AI		AI, Loss		
ADM2	22	50,919,984-50,924,866	Loss	Loss	Loss	Loss	AI, Loss	AI, Loss	AI	AI, Loss	Loss	
ADORA2A	22	24,813,708-24,838,328	AI, Loss	AI, Loss	AI, Loss	AI, Loss	AI, Loss	AI, Loss	AI	AI, Loss		
ADORA2A-AS1	22	24,825,175-24,891,042	AI, Loss	AI, Loss	AI, Loss	AI, Loss	AI, Loss	AI, Loss	AI	AI, Loss		
ADRBK2	22	25,960,860-26,125,258	AI, Loss	AI, Loss	AI, Loss	AI, Loss	AI, Loss	AI, Loss	AI, Loss	AI, Loss	AI, Loss	
AK026502	22	27,068,848-27,114,949	AI, Loss	AI, Loss	Loss	AI, Loss	AI, Loss	AI	AI	AI, Loss	AI, Loss	

AK055980	22	27,444,105-27,456,467	Loss	AI, Loss	Loss	AI, Loss	AI, Loss	AI, Loss	AI, Loss	AI, Loss	AI, Loss	
AK074476	22	31,831,222-31,834,552		AI, Loss	AI, Loss	Loss	AI, Loss	AI, Loss	AI	Loss		
AK093107	22	48,027,451-48,251,349	AI, Loss	AI, Loss	AI, Loss	AI, Loss	AI, Loss	AI, Loss	AI	AI, Loss	AI, Loss	
AK097791	22	38,037,838-38,054,384	Loss	Loss	Loss	Loss	Loss	Loss	AI	Loss	AI, Loss	
AK123632	22	37,099,962-37,117,494	AI, Loss	AI, Loss	AI, Loss	AI, Loss	AI, Loss	Loss	AI	Loss	AI, Loss	
AK123891	22	32,896,531-32,898,414	AI, Loss	AI, Loss	AI, Loss	AI, Loss	AI, Loss	AI, Loss	AI	AI, Loss	AI, Loss	
AK128837	22	21,538,002-21,546,445	Loss	Loss		AI, Loss		AI, Loss	AI	AI, Loss		
AK131325	22	22,345,495-22,398,332	AI, Loss	AI, Loss	AI, Loss	AI, Loss	AI, Loss	AI, Loss	AI, Loss	AI, Loss		
ANKRD54	22	38,226,861-38,245,325	Loss	Loss	Loss	Loss	AI, Loss	Loss	Loss	Loss	Loss	
ANKRD62P1-PARP4P3	22	17,134,598-17,156,430	Loss	AI, Loss		Loss		AI		AI, Loss		
AP1B1	22	29,723,668-29,784,572	AI, Loss	AI, Loss	AI, Loss	AI, Loss	AI, Loss	AI, Loss	AI, Loss	AI, Loss	AI, Loss	
AP1B1P1	22	32,517,963-32,529,456	AI, Loss	AI, Loss	AI, Loss	AI, Loss	AI, Loss	AI, Loss	Loss	AI, Loss	AI, Loss	
APOBEC3F	22	39,436,672-39,451,975	Loss	Loss	Loss	Loss	AI, Loss	AI	AI		Loss	
APOBEC3G	22	39,436,672-39,483,748	Loss	Loss	Loss	Loss	AI, Loss	AI	AI		Loss	
APOBEC3H	22	39,493,228-39,500,072		Loss	AI, Loss	Loss	Loss	AI	AI		Loss	
APOL1	22	36,649,116-36,663,577	Loss	Loss		AI, Loss	AI, Loss	AI, Loss	AI	AI, Loss	AI, Loss	
APOL2	22	36,622,254-36,636,000	Loss	Loss	Loss	AI, Loss	AI, Loss	AI, Loss	AI	AI, Loss	AI, Loss	
APOL3	22	36,536,370-36,562,225	Loss	Loss	Loss	AI, Loss	AI, Loss	AI, Loss	AI	AI, Loss	AI, Loss	
APOL4	22	36,585,175-36,600,879	Loss	Loss	Loss	AI, Loss	AI, Loss	AI, Loss	AI	AI, Loss	AI, Loss	
ARFGAP3	22	43,192,531-43,253,408	AI, Loss	AI, Loss	Loss	Loss	AI, Loss	Loss	AI	AI, Loss	AI, Loss	
ARHGAP8	22	45,148,437-45,258,664	Loss	Loss	Loss	AI, Loss	AI, Loss	AI, Loss	AI	AI, Loss	AI, Loss	
ARSA	22	51,061,181-51,066,601	Loss	Loss		Loss	AI, Loss	AI, Loss	AI	AI, Loss	Loss	
ARVCF	22	19,957,401-20,004,309	AI, Loss	AI, Loss		AI, Loss	Gain	AI		AI, Loss		
ASCC2	22	30,184,596-30,234,293	AI, Loss	AI, Loss	AI, Loss	AI, Loss	AI, Loss	AI, Loss	AI	AI, Loss	AI, Loss	
ASPHD2	22	26,825,279-26,840,978	AI, Loss	AI, Loss	AI, Loss	AI, Loss	AI, Loss	AI, Loss	AI	AI, Loss	AI, Loss	
ATP6V1E1	22	18,074,902-18,111,588	AI, Loss	Loss		Loss	AI	AI		AI, Loss		
ATXN10	22	46,067,677-46,241,187	Loss	AI, Loss	AI, Loss	AI, Loss	AI, Loss	AI, Loss	AI, Loss	AI, Loss	AI, Loss	
AX747137	22	44,220,386-44,222,913	Loss	AI	AI, Loss	AI, Loss	AI	AI, Loss	AI	AI, Loss	AI, Loss	
AX747758	22	36,633,472-36,635,231		Loss		AI, Loss	AI, Loss	AI, Loss	AI	AI, Loss	AI, Loss	
AX747952	22	44,383,785-44,385,884	AI, Loss		AI, Loss	AI, Loss	AI, Loss	AI, Loss	AI	AI, Loss	AI, Loss	
AX748067	22	21,642,518-21,646,214	Loss	Loss		AI, Loss		AI, Loss	AI	AI, Loss		
AX748308	22	28,276,021-28,277,106	AI, Loss	AI, Loss	AI, Loss	AI, Loss	AI, Loss	AI, Loss	Loss	Loss	AI, Loss	
BC015159	22	27,619,257-27,622,760	Loss	AI, Loss		AI, Loss	AI, Loss	AI, Loss	AI	AI, Loss	AI, Loss	
BC021738	22	17,602,484-17,612,994	AI, Loss	AI, Loss		AI, Loss		AI		Loss		
BC033837	22	49,808,173-50,051,190	AI, Loss	AI, Loss	AI, Loss	AI, Loss	AI, Loss	AI, Loss	AI	AI, Loss	AI, Loss	
BC035867	22	20,970,516-21,011,201	Loss	AI, Loss		AI, Loss	AI, Gain	AI	AI	AI, Loss		
BC037972	22	47,857,047-47,882,860	AI, Loss	Loss	AI, Loss	AI, Loss	AI, Loss	AI, Loss	AI, Loss	AI, Loss	AI, Loss	
BC038197	22	17,227,758-17,229,328	Loss	AI, Loss		Loss	Gain	AI		AI, Loss		
BC038245	22	42,760,534-42,765,180		Loss		Loss	AI, Loss	AI	AI	AI	Loss	
BC039313	22	21,538,002-21,546,445	Loss	Loss		AI, Loss		AI, Loss	AI	AI, Loss		
BC050343	22	51,174,256-51,176,567	Loss	Loss	Loss	Loss	AI, Loss		AI		Loss	
BC069815	22	31,601,248-31,601,602	Loss	AI, Loss	AI, Loss	Loss	AI, Loss	AI, Loss	AI	Loss		
BC089413	22	22,380,461-22,380,936	AI, Loss	AI, Loss	AI, Loss	AI, Loss	AI, Loss	AI, Loss	AI, Loss	AI, Loss		
BC127823	22	38,615,652-38,616,226		Loss		Loss	AI, Loss	AI	AI	AI	Loss	

BCL2L13	22	18,111,681-18,213,621	AI, Loss	Loss		Loss	AI, Gain	AI		AI, Loss		
BCR	22	23,522,551-23,660,224	AI, Loss	AI, Loss	AI, Loss	AI, Loss	AI, Loss	AI, Loss	AI	AI		
BCR	22	22,979,652-22,981,152	AI, Loss	AI, Loss	AI, Loss	AI, Loss	AI, Loss		AI			
BCR	22	21,642,517-21,644,017	Loss	Loss		AI, Loss		AI, Loss	AI	AI, Loss		
BCRP2	22	21,457,304-21,476,575	Loss	Loss		AI, Loss		AI, Loss	AI	AI, Loss		
BCRP3	22	25,028,881-25,049,327	AI, Loss	AI, Loss	AI, Loss	AI, Loss	AI, Loss	AI, Loss	Loss	AI, Loss		
BID	22	18,216,905-18,257,431	Loss	Loss		Loss	Gain	AI		AI, Loss		
BIK	22	43,506,753-43,525,718	AI, Loss	AI, Loss	Loss	Loss	AI, Loss	AI, Loss	AI	AI	AI, Loss	
bK250D10.C22.8	22	42,120,358-42,354,544	AI, Loss	Loss	AI, Loss	AI, Loss	AI, Loss	Loss	Loss	AI, Loss	Loss	
BMS1P20	22	22,652,462-22,677,324	AI, Loss	AI, Loss	AI, Loss	AI, Loss	AI, Loss	AI, Loss	AI	AI, Loss		
BPIFC	22	32,809,833-32,860,433	AI, Loss	AI, Loss	AI, Loss	AI, Loss	AI, Loss	AI, Loss	AI, Loss	AI, Loss	AI, Loss	
BRD1	22	50,166,925-50,221,575	AI, Loss		AI, Loss	AI, Loss	AI, Loss	AI, Loss	AI		AI, Loss	
BX537634	22	37,154,254-37,158,116	AI, Loss	AI, Loss	AI, Loss	AI, Loss	AI, Loss	Loss	AI	Loss	AI, Loss	
C1QTNF6	22	37,576,205-37,595,463	Loss	Loss		Loss	AI, Loss	Loss	AI	Loss	AI, Loss	
C22orf29	22	19,833,660-19,842,462	AI, Loss	AI, Loss		AI, Loss		AI	AI	AI, Loss		
C22orf34	22	49,808,173-50,051,190	AI, Loss	AI, Loss	AI, Loss	AI, Loss	AI, Loss	AI, Loss	AI	AI, Loss	AI, Loss	
C22orf42	22	32,544,992-32,555,279	AI, Loss	AI, Loss	AI, Loss	AI, Loss	AI, Loss	AI, Loss	Loss	AI, Loss	AI, Loss	
C22orf43	22	23,950,638-23,974,508	Loss	AI, Loss	AI, Loss	AI, Loss	AI, Loss	AI, Loss	AI	Loss		
CABIN1	22	24,407,764-24,574,596	Loss	AI, Loss	Loss	Loss	AI, Loss	AI, Loss	AI	AI, Loss		
CABP7	22	30,116,343-30,127,820	AI, Loss	AI, Loss	AI, Loss	AI, Loss	AI, Loss	AI, Loss	AI	AI, Loss	AI, Loss	
CACNA1I	22	39,966,757-40,085,740	AI, Loss	AI, Loss	AI, Loss	AI, Loss	AI, Loss	AI, Loss	AI	AI, Loss	Loss	
CACNG2	22	36,956,915-37,098,903	AI, Loss	AI, Loss	AI, Loss	AI, Loss	AI, Loss	AI, Loss	AI	AI, Loss	AI, Loss	
CARD10	22	37,875,382-37,915,378	Loss	Loss	Loss	Loss	Loss	AI, Loss	AI	AI, Loss	AI, Loss	
CBX7	22	39,526,778-39,548,655			AI	Loss	Loss	AI	AI		Loss	
CCDC117	22	29,168,661-29,185,289	AI, Loss	AI, Loss	AI, Loss	AI, Loss	AI, Loss	AI, Loss	AI, Loss	AI, Loss	AI, Loss	
CCDC134	22	42,196,625-42,222,303	AI, Loss	Loss	AI, Loss	AI, Loss	AI, Loss	Loss	Loss	AI, Loss	Loss	
CCDC157	22	30,752,623-30,774,644	Loss	Loss	AI, Loss	AI, Loss	AI, Loss	AI, Loss	AI, Loss	AI, Loss	AI, Loss	
CCT8L2	22	17,071,647-17,073,700	Loss	AI, Loss		Loss		AI		AI, Loss		
CDC45	22	19,467,348-19,508,135	AI, Loss	AI, Loss		AI, Loss		AI		AI, Loss		
CECR1	22	17,660,191-17,702,738	AI, Loss	AI, Loss		AI, Loss		AI		AI, Loss		
CECR2	22	17,840,838-18,037,856	AI, Loss	AI, Loss		AI, Loss	Gain	AI	AI	AI, Loss		
CECR5	22	17,618,409-17,646,177	AI, Loss	AI, Loss		AI, Loss		AI		Loss		
CECR5-AS1	22	17,640,278-17,646,335	AI, Loss	AI, Loss		AI, Loss		AI		Loss		
CECR6	22	17,597,188-17,602,257	AI, Loss	AI, Loss		AI, Loss		AI		Loss		
CECR7	22	17,517,459-17,540,960	AI, Loss	AI, Loss		AI, Loss		AI		AI, Loss		
CELSR1	22	46,756,730-46,933,067	Loss	Loss	Loss	AI, Loss	AI, Loss	AI, Loss	AI, Loss	AI, Loss	AI, Loss	
CENPM	22	42,334,724-42,343,168	AI, Loss	Loss	AI, Loss	AI, Loss	AI, Loss	Loss	Loss	AI, Loss	Loss	
CERK	22	47,080,306-47,134,152	Loss	Loss	AI, Loss	AI, Loss	Loss	Loss	AI	AI	AI, Loss	
CES5AP1	22	23,701,792-23,724,313	Loss	AI, Loss	AI, Loss	AI, Loss	AI, Loss	AI, Loss	AI	AI		
CHADL	22	41,625,513-41,636,935	AI, Loss	AI, Loss	AI, Loss	AI, Loss	AI, Loss	Loss		AI, Loss		
CLDN5	22	19,510,546-19,512,860	AI, Loss	AI, Loss		AI, Loss		AI		AI, Loss		
CLTCL1	22	19,166,986-19,279,239	AI, Loss	AI, Loss		AI, Loss	Gain	AI	AI	Loss		
COMT	22	19,929,262-19,957,498	AI, Loss	AI, Loss		AI, Loss	Gain	AI		AI, Loss		
CR936633	22	27,706,611-27,713,417	Loss	AI, Loss	Loss	AI, Loss	AI, Loss	AI, Loss	AI	AI, Loss	AI, Loss	

CRYBA4	22	27,017,927-27,026,636	AI, Loss	AI, Loss	AI, Loss	AI, Loss	AI, Loss	AI	AI	AI, Loss	AI, Loss	
CRYBB1	22	26,995,241-27,013,991	AI, Loss	AI, Loss	AI, Loss	AI, Loss	AI, Loss	AI	AI	AI, Loss	AI, Loss	
CRYBB2P1	22	25,844,053-25,907,585	AI, Loss	AI, Loss	AI, Loss	AI, Loss	AI, Loss	AI, Loss	AI, Loss	AI, Loss	AI, Loss	
CS330190	22	43,172,869-43,172,890	AI, Loss	AI, Loss	Loss	Loss	AI, Loss	Loss	AI	AI, Loss	AI, Loss	
CSF2RB	22	37,309,674-37,336,479	AI, Loss	AI, Loss	AI, Loss	AI, Loss	AI, Loss	AI, Loss	AI	Loss	AI, Loss	
CYP2D6	22	42,522,500-42,526,883	Loss	Loss	Loss	AI, Loss	AI, Loss	Loss	AI	AI, Loss	Loss	
CYTH4	22	37,678,423-37,711,389	Loss	Loss	Loss	Loss	AI, Loss	AI, Loss	AI	AI, Loss	AI, Loss	
DDT	22	24,313,553-24,322,019	Loss	AI, Loss	Loss	Loss	AI, Loss	AI, Loss	AI	AI, Loss		
DDTL	22	24,309,025-24,314,748	Loss	AI, Loss	Loss	Loss	AI, Loss	AI, Loss	AI	AI, Loss		
DDX17	22	38,879,442-38,903,665	Loss	Loss	Loss	Loss	AI, Loss	Loss	Loss	Loss	Loss	
DENND6B	22	50,750,391-50,765,489		Loss	AI	AI, Loss	AI, Loss		AI	Loss	Loss	
DEPDC5	22	32,149,936-32,303,020	AI, Loss	AI, Loss	AI, Loss	Loss	AI, Loss	AI, Loss	AI, Loss	AI, Loss	AI, Loss	
DGCR10	22	19,010,136-19,011,063	AI, Loss	AI, Loss		Loss		AI	AI	Loss		AI
DGCR11	22	19,033,674-19,035,888	AI, Loss	AI, Loss		Loss		AI	AI	Loss		
DGCR2	22	19,023,794-19,109,967	AI, Loss	AI, Loss		Loss		AI	AI	Loss		
DGCR5	22	18,958,010-19,018,755	AI, Loss	AI, Loss		Loss		AI	AI	Loss		AI
DGCR6L	22	20,301,760-20,307,628	Loss	Loss		AI, Loss	Gain	AI	AI	AI, Loss		
DGCR8	22	20,067,754-20,099,400	Loss			AI, Loss	Gain	AI		AI, Loss		
DGCR9	22	19,005,346-19,007,761	AI, Loss	AI, Loss		Loss		AI	AI	Loss		AI
DKFZp434K191	22	25,046,352-25,047,058	AI, Loss	AI, Loss	AI, Loss	AI, Loss	AI, Loss	AI, Loss	Loss	AI, Loss		
DKFZp667J0810	22	22,786,692-23,248,968	AI, Loss	AI, Loss	AI, Loss	AI, Loss	AI, Loss	AI, Loss	AI	AI, Loss		
DKFZp686N1150	22	38,615,297-38,620,110		Loss		Loss	AI, Loss	AI	AI	AI	Loss	
DM074112	22	38,075,645-38,075,666	Loss	Loss	Loss	Loss	Loss	Loss	AI	Loss	AI, Loss	
DMC1	22	38,914,953-38,966,201	Loss	Loss	Loss	Loss	AI, Loss	Loss	Loss	Loss	Loss	
DQ570096	22	18,215,565-18,217,980		Loss		Loss	Gain	AI		AI, Loss		
DQ570150	22	21,481,136-21,481,174	Loss	Loss		AI, Loss		AI, Loss	AI	AI, Loss		
DQ571361	22	24,239,835-24,239,864	Loss	AI, Loss	Loss	Loss	AI, Loss	AI, Loss	AI, Loss	AI, Loss		
DQ571479	22	17,029,615-17,029,643	Loss	AI, Loss		Loss		AI		AI, Loss		
DQ573684	22	16,240,245-16,240,277	Loss			Loss		AI		AI, Loss		
DQ574115	22	24,238,568-24,238,598	Loss	AI, Loss	Loss	Loss	AI, Loss	AI, Loss	AI, Loss	AI, Loss		
DQ574263	22	21,355,229-21,355,259	Loss	Loss		AI, Loss		AI	AI			
DQ575315	22	24,240,066-24,240,132	Loss	AI, Loss	Loss	Loss	AI, Loss	AI, Loss	AI, Loss	AI, Loss		
DQ576853	22	24,658,457-24,658,483	AI, Loss	AI, Loss	AI, Loss	AI, Loss	AI, Loss	AI, Loss	AI	AI, Loss		
DQ579704	22	24,239,638-24,239,678	Loss	AI, Loss	Loss	Loss	AI, Loss	AI, Loss	AI, Loss	AI, Loss		
DQ582484	22	16,243,907-16,243,948	Loss			Loss		AI		AI, Loss		
DQ584254	22	24,239,748-24,239,792	Loss	AI, Loss	Loss	Loss	AI, Loss	AI, Loss	AI, Loss	AI, Loss		
DQ585141	22	18,985,168-18,985,205	AI, Loss	AI, Loss		Loss		AI	AI	Loss		AI
DQ586720	22	23,952,777-23,952,803	Loss	AI, Loss	AI, Loss	AI, Loss	AI, Loss	AI, Loss	AI	Loss		
DQ590589	22	16,239,287-16,239,327	Loss			Loss		AI		AI, Loss		
DQ595048	22	16,240,300-16,240,340	Loss			Loss		AI		AI, Loss		
DQ595055	22	43,182,195-43,182,228	AI, Loss	AI, Loss	Loss	Loss	AI, Loss	Loss	AI	AI, Loss	AI, Loss	
DQ596074	22	24,253,496-24,253,538	Loss	AI, Loss	Loss	Loss	AI, Loss	AI, Loss	AI	AI, Loss		
DQ596562	22	24,255,602-24,255,629	Loss	AI, Loss	Loss	Loss	AI, Loss	AI, Loss	AI	AI, Loss		
DQ596940	22	42,222,470-42,222,500	AI, Loss	Loss	AI, Loss	AI, Loss	AI, Loss	Loss	Loss	AI, Loss	Loss	

DQ597394	22	24,240,744-24,240,774	Loss	AI, Loss	Loss	Loss	AI, Loss	AI, Loss	AI, Loss	AI, Loss		
DQ600483	22	24,245,625-24,247,864	Loss	AI, Loss	Loss	Loss	AI, Loss	AI, Loss	AI, Loss	AI, Loss		
DQ601926	22	24,235,191-24,235,227	Loss	AI, Loss	Loss	Loss	AI, Loss	AI, Loss	AI, Loss	AI, Loss		
DRG1	22	31,795,538-31,830,172	Loss	AI, Loss	AI, Loss	Loss	AI, Loss	AI, Loss	AI	Loss		
DRICH1	22	23,950,638-23,974,508	Loss	AI, Loss	AI, Loss	AI, Loss	AI, Loss	AI, Loss	AI	Loss		
EFCAB6	22	43,924,623-44,208,217	Loss	Loss	AI, Loss	AI, Loss	AI, Loss	AI, Loss	AI, Loss	AI, Loss	AI, Loss	Loss
EFCAB6-AS1	22	43,912,133-43,932,485	Loss	Loss	AI, Loss	Loss	AI, Loss	AI, Loss	AI	Loss	AI, Loss	Loss
EIF3L	22	38,245,378-38,284,789	Loss	Loss	Loss	Loss	AI, Loss	Loss	Loss	Loss	Loss	
EIF4ENIF1	22	31,835,344-31,885,923	AI, Loss	AI, Loss	AI, Loss	Loss	AI, Loss	AI, Loss	AI, Loss	Loss		
ELFN2	22	37,736,684-37,823,505	Loss	Loss	Loss	Loss	Loss	AI, Loss	AI	AI, Loss	AI, Loss	
Em:AC005003.4	22	31,742,871-31,744,504	Loss	AI, Loss	AI, Loss	Loss	AI, Loss	AI, Loss	AI	Loss		
EMID1	22	29,601,900-29,655,586	AI, Loss	AI, Loss	AI, Loss	AI, Loss	AI, Loss	AI, Loss	AI, Loss	AI, Loss	AI, Loss	
ENTHD1	22	40,139,048-40,289,794	AI, Loss	AI, Loss	AI, Loss	AI, Loss	AI, Loss	AI, Loss	AI	AI, Loss	Loss	
EU036692	22	24,827,818-24,828,694	AI, Loss	AI, Loss	AI, Loss	AI, Loss	AI, Loss	AI, Loss	AI	AI, Loss		
EWSR1	22	29,663,997-29,696,515	AI, Loss	AI, Loss	AI, Loss	AI, Loss	AI, Loss	AI, Loss	Loss	AI, Loss	AI, Loss	
FAM118A	22	45,704,840-45,737,836	Loss	Loss	Loss	AI, Loss	AI, Loss	AI, Loss	AI, Loss	AI, Loss	AI, Loss	
FAM19A5	22	48,885,271-49,147,747	AI, Loss	AI, Loss	AI, Loss	AI, Loss	AI, Loss	AI, Loss	AI	AI, Loss	AI, Loss	
FAM211B	22	24,981,590-24,989,035	AI, Loss	AI, Loss	AI, Loss	AI, Loss	AI, Loss	AI, Loss	Loss	AI, Loss		
FAM227A	22	38,974,124-39,052,634	Loss	Loss	Loss	Loss	AI, Loss	Loss	Loss	Loss	Loss	
FAM230A	22	20,336,993-20,350,461	Loss	Loss		AI, Loss	Gain	AI	AI	AI, Loss		
FAM230B	22	21,521,191-21,546,445	Loss	Loss		AI, Loss		AI, Loss	AI	AI, Loss		
FAM230G	22	20,328,519-20,706,784	Loss	Loss		AI, Loss	Gain	AI	AI	AI, Loss		
FAM230H	22	21,655,548-21,680,925	Loss	Loss		AI, Loss		AI, Loss	AI	AI, Loss		
FAM230I	22	23,804,268-23,829,167	Loss	AI, Loss	AI, Loss	AI, Loss	AI, Loss	AI, Loss	AI	AI		
FAM230J	22	20,632,131-20,656,828	Loss	Loss		AI, Loss	Gain	AI	AI	AI, Loss		
FAM83F	22	40,390,952-40,426,043	AI, Loss	AI, Loss	AI, Loss	AI, Loss	AI, Loss	AI, Loss	AI	AI, Loss	Loss	
FBLN1	22	45,898,718-45,997,014	Loss	AI	Loss	AI, Loss	Loss	AI	AI	AI	AI, Loss	
FBXO7	22	32,870,706-32,894,818	AI, Loss	AI, Loss	AI, Loss	AI, Loss	AI, Loss	AI, Loss	AI	AI, Loss	AI, Loss	
FBXW4P1	22	23,604,953-23,607,186	AI, Loss	AI, Loss	AI, Loss	AI, Loss	AI, Loss	AI, Loss	AI	AI		
FOXRED2	22	36,883,232-36,903,148				AI, Loss	AI, Loss	AI	AI	AI		
GAB4	22	17,442,825-17,489,112	AI, Loss	AI, Loss		AI, Loss	Gain	AI		AI, Loss		
GAL3ST1	22	30,950,621-30,970,574	Loss	Loss	AI, Loss	Loss	AI, Loss	AI, Loss	AI	AI, Loss	AI, Loss	
GALR3	22	38,219,388-38,221,502	Loss	Loss	Loss	Loss	AI, Loss	Loss	Loss	Loss	Loss	
GAS2L1	22	29,702,984-29,708,778	AI, Loss	AI, Loss	AI, Loss	AI, Loss	AI, Loss	AI, Loss	Loss	AI, Loss	AI, Loss	
GGA1	22	38,004,480-38,029,571	Loss	Loss	Loss	Loss	Loss	Loss	AI	Loss	AI, Loss	
GGT1	22	24,979,717-25,024,972	AI, Loss	AI, Loss	AI, Loss	AI, Loss	AI, Loss	AI, Loss	Loss	AI, Loss		
GGT2	22	21,562,261-21,632,151	Loss	Loss		AI, Loss		AI, Loss	AI	AI, Loss		
GGT5	22	24,615,621-24,641,110	AI, Loss	AI, Loss	AI, Loss	AI, Loss	AI, Loss	AI, Loss	AI	AI, Loss		
GGTLC3	22	20,366,210-20,368,028	Loss	Loss		AI, Loss	Gain	AI	AI	AI, Loss		
GNAZ	22	23,412,539-23,467,224	AI, Loss	AI, Loss	AI, Loss	AI, Loss	AI, Loss	AI, Loss	AI	AI		
GNB1L	22	19,775,933-19,842,462	AI, Loss	AI, Loss		AI, Loss	Gain	AI	AI	AI, Loss		
GP1BB	22	19,711,065-19,712,297	AI, Loss	AI, Loss		AI, Loss		AI	AI	AI, Loss		
GRAMD4	22	46,972,984-47,078,653	Loss	Loss	Loss	AI, Loss	Loss	AI, Loss	AI	AI	AI, Loss	
GRAP2	22	40,297,085-40,369,346	AI, Loss	AI, Loss	AI, Loss	AI, Loss	AI, Loss	AI, Loss	AI	AI, Loss	Loss	

GRK3	22	25,960,860-26,125,258	AI, Loss	AI, Loss	AI, Loss	AI, Loss	AI, Loss	AI, Loss	AI, Loss	AI, Loss	AI, Loss	
GSC2	22	19,136,503-19,137,796	AI, Loss	AI, Loss		AI, Loss		AI	AI	Loss		
GSTT1	22	24,376,132-24,384,311	Loss	AI, Loss	Loss	Loss	AI, Loss	AI, Loss	AI	AI, Loss		
GSTT1-AS1	22	24,375,962-24,376,214	Loss	AI, Loss	Loss	Loss	AI, Loss	AI, Loss	AI	AI, Loss		
GSTT2	22	24,299,600-24,303,488	Loss	AI, Loss	Loss	Loss	AI, Loss	AI, Loss	AI	AI, Loss		
GSTT2	22	24,322,218-24,326,106	Loss	AI, Loss	Loss	Loss	AI, Loss	AI, Loss	AI	AI, Loss		
GSTT2B	22	24,299,600-24,303,393	Loss	AI, Loss	Loss	Loss	AI, Loss	AI, Loss	AI	AI, Loss		
GSTT4	22	24,340,594-24,401,807	Loss	AI, Loss	Loss	Loss	AI, Loss	AI, Loss	AI	AI, Loss		
GSTTP1	22	24,340,594-24,347,258	Loss	AI, Loss	Loss	Loss	AI, Loss	AI, Loss	AI	AI, Loss		
GSTTP2	22	24,385,937-24,401,899	Loss	AI, Loss	Loss	Loss	AI, Loss	AI, Loss	AI	AI, Loss		
GTPBP1	22	39,101,806-39,129,592	Loss	Loss	Loss	Loss	AI, Loss	Loss	AI, Loss		Loss	
GUCD1	22	24,936,390-24,951,903	AI, Loss	AI, Loss	AI, Loss	AI, Loss	AI, Loss	AI, Loss	AI	AI, Loss		
GUSBP11	22	23,980,674-24,059,610	Loss	AI, Loss	AI, Loss	AI, Loss	AI, Loss	AI, Loss	AI	Loss		
HDAC10	22	50,683,612-50,689,834	Loss	Loss	AI	AI, Loss	AI, Loss	Loss	AI	Loss		
HDHD5	22	17,618,409-17,646,177	AI, Loss	AI, Loss		AI, Loss		AI		Loss		
HDHD5-AS1	22	17,640,278-17,646,335	AI, Loss	AI, Loss		AI, Loss		AI		Loss		
HIC2	22	21,771,692-21,805,750	Loss	Loss		AI, Loss		AI, Loss	AI	AI, Loss		
HIRA	22	19,318,223-19,435,755	AI, Loss	AI, Loss		AI, Loss		AI		Loss		
HMGXB4	22	35,653,444-35,691,800	AI, Loss	AI, Loss	AI, Loss	AI, Loss	AI, Loss	AI, Loss	AI	AI, Loss	AI, Loss	
HMOX1	22	35,777,059-35,790,207	AI, Loss	AI, Loss	AI, Loss	AI, Loss	AI, Loss	AI	AI	AI	AI, Loss	
HORMAD2	22	30,476,057-30,573,064	AI, Loss	AI, Loss	AI, Loss	AI, Loss	AI, Loss	AI, Loss	AI	AI, Loss	AI, Loss	
HORMAD2-AS1	22	30,404,730-30,476,469	AI, Loss	AI, Loss	AI, Loss	AI, Loss	AI, Loss	AI, Loss	AI, Loss	AI, Loss	AI, Loss	
HPS4	22	26,839,074-26,879,829	AI, Loss	AI, Loss	AI, Loss	AI, Loss	AI, Loss	AI, Loss	AI	AI, Loss	AI, Loss	
HSFY1P1	22	17,308,363-17,310,225	Loss	AI, Loss		Loss	Gain	AI		AI, Loss		
HV593096	22	20,390,284-20,390,311	Loss	Loss		AI, Loss	Gain	AI	AI	AI, Loss		
HV593127	22	20,397,894-20,397,917	Loss	Loss		AI, Loss	Gain	AI	AI	AI, Loss		
HV593134	22	20,397,144-20,397,171	Loss	Loss		AI, Loss	Gain	AI	AI	AI, Loss		
HV593135	22	20,398,579-20,398,601	Loss	Loss		AI, Loss	Gain	AI	AI	AI, Loss		
HV593178	22	20,398,307-20,398,335	Loss	Loss		AI, Loss	Gain	AI	AI	AI, Loss		
IFT27	22	37,154,245-37,172,177	AI, Loss	AI, Loss	AI, Loss	AI, Loss	AI, Loss	Loss	AI	Loss	AI, Loss	
IGKV1-12	22	17,385,314-17,385,395	Loss	AI, Loss		Loss	Gain	Loss		AI, Loss		
IGLL1	22	23,915,312-23,922,495	Loss	AI, Loss	AI, Loss	AI, Loss	AI, Loss	AI, Loss	AI	Loss		
IL17RA	22	17,565,848-17,596,584	AI, Loss	AI, Loss		AI, Loss		AI		Loss		
IL2RB	22	37,521,879-37,571,158	AI, Loss	AI, Loss	AI, Loss	Loss	AI, Loss	Loss	AI	AI, Loss	AI, Loss	
INPP5J	22	31,503,960-31,530,683	Loss	Loss	AI, Loss	Loss	AI, Loss	AI, Loss	AI	Loss	Loss	
ISX-AS1	22	35,152,655-35,393,905	AI, Loss	AI, Loss	AI, Loss	AI, Loss	AI, Loss	AI, Loss	AI, Loss	AI, Loss	AI, Loss	
JB153905	22	32,744,122-32,744,144	AI, Loss	AI, Loss	AI, Loss	AI, Loss	AI, Loss	AI, Loss	AI	AI, Loss	AI, Loss	
JB175027	22	32,524,336-32,524,357	AI, Loss	AI, Loss	AI, Loss	AI, Loss	AI, Loss	AI, Loss	Loss	AI, Loss	AI, Loss	
JX456220	22	20,695,276-20,710,250	Loss	Loss		AI, Loss	Gain	AI	AI	AI, Loss		
KDELR3	22	38,864,066-38,879,452	Loss	Loss	Loss	Loss	AI, Loss	Loss	AI, Loss	Loss	Loss	
KIAA0845	22	29,885,264-29,887,277	AI, Loss	AI, Loss	AI, Loss	AI, Loss	AI, Loss	AI, Loss	AI	AI, Loss	AI, Loss	
KIAA0930	22	45,588,122-45,636,650	AI, Loss	AI, Loss	AI, Loss	AI, Loss	AI, Loss	AI, Loss	AI	AI, Loss	AI, Loss	
KIAA1644	22	44,639,556-44,708,731	AI, Loss	AI, Loss		AI, Loss	AI, Loss	AI, Loss	AI	AI, Loss	AI, Loss	
KIAA1653	22	20,291,560-20,299,716	Loss	Loss		AI, Loss	Gain	AI	AI	AI, Loss		

KIAA1656	22	30,764,799-30,773,894	Loss	Loss	AI, Loss	AI, Loss	AI, Loss	AI, Loss	AI, Loss	AI, Loss	AI, Loss	
KLHDC7B	22	50,984,631-50,989,452	Loss	Loss	Loss	Loss	AI, Loss	AI, Loss	AI	AI, Loss	Loss	
KLHL22	22	20,795,805-20,850,170	Loss	AI, Loss		AI, Loss		AI		AI, Loss		
L3MBTL2	22	41,601,312-41,627,275	AI, Loss	AI, Loss	AI, Loss	AI, Loss	AI, Loss	Loss		AI, Loss		
LARGE	22	33,669,061-34,318,584	AI, Loss	AI, Loss	AI, Loss	AI, Loss	AI, Loss	AI, Loss	AI, Loss	AI, Loss	AI, Loss	
LARGE-AS1	22	34,120,971-34,146,803	Loss		AI, Loss	AI, Loss	AI, Loss	AI, Loss	AI	AI, Loss	AI, Loss	
LARGE1	22	33,668,511-34,318,876	AI, Loss	AI, Loss	AI, Loss	AI, Loss	AI, Loss	AI, Loss	AI, Loss	AI, Loss	AI, Loss	
LDOC1L	22	44,888,449-44,894,005	AI, Loss	AI, Loss	AI, Loss	AI, Loss	AI, Loss	AI, Loss	AI	AI, Loss	AI, Loss	
LGALS1	22	38,071,612-38,075,809	Loss	Loss	Loss	Loss	Loss	Loss	AI	Loss	AI, Loss	
LIMK2	22	31,608,249-31,676,066	Loss	AI, Loss	AI, Loss	Loss	AI, Loss	AI, Loss	AI	Loss		
LINC00207	22	44,965,219-44,968,329	AI, Loss	AI, Loss	AI, Loss	AI, Loss	AI, Loss	AI, Loss	AI	AI, Loss	AI, Loss	
LINC00229	22	45,002,207-45,021,299	AI, Loss	AI, Loss	AI, Loss	AI, Loss	AI, Loss	AI, Loss	AI	AI, Loss	AI, Loss	
LINC00528	22	18,260,055-18,262,247	Loss	Loss		Loss		AI	AI	AI, Loss		
LINC00634	22	42,348,190-42,354,946	AI, Loss	Loss	AI, Loss	AI, Loss	AI, Loss	Loss	Loss	AI, Loss	Loss	
LINC00895	22	19,553,652-19,554,362	AI, Loss	AI, Loss		AI, Loss		AI		AI, Loss		
LINC00896	22	20,193,854-20,196,060	Loss	Loss		AI, Loss		AI	AI	AI, Loss		
LINC00899	22	46,435,786-46,440,748		AI, Loss	AI, Loss	AI, Loss	AI, Loss	AI, Loss	AI, Loss	AI, Loss	AI, Loss	
LINC01310	22	49,262,581-49,294,198	AI, Loss	AI, Loss	AI, Loss	AI, Loss	AI, Loss	AI, Loss	AI	AI, Loss	AI, Loss	
LINC01311	22	19,159,218-19,160,345	AI, Loss	AI, Loss		AI, Loss		AI	AI	Loss		
LINC01315	22	42,760,405-42,765,214		Loss		Loss	AI, Loss	AI	AI	AI	Loss	
LINC01399	22	35,515,816-35,627,049	AI, Loss	AI, Loss	AI, Loss	AI, Loss	AI, Loss	AI, Loss	AI	AI, Loss	AI, Loss	
LINC01521	22	31,742,744-31,747,141	Loss	AI, Loss	AI, Loss	Loss	AI, Loss	AI, Loss	AI	Loss		
LINC01638	22	27,617,307-27,620,688	Loss	AI, Loss		AI, Loss	AI, Loss	AI, Loss	AI	AI, Loss	AI, Loss	
LINC01639	22	43,796,336-43,805,687	Loss	Loss	AI, Loss	Loss	AI, Loss	AI, Loss	AI	Loss	AI, Loss	
LINC01640	22	33,504,514-33,512,280	AI, Loss	AI, Loss	AI, Loss	AI, Loss	AI, Loss	AI, Loss	AI	AI, Loss	AI, Loss	
LINC01644	22	47,857,047-47,882,860	AI, Loss	Loss	AI, Loss	AI, Loss	AI, Loss	AI, Loss	AI, Loss	AI, Loss	AI, Loss	
LINC01656	22	44,839,206-44,840,668	AI, Loss	AI, Loss	AI, Loss	AI, Loss	AI, Loss	AI, Loss	AI	AI, Loss	AI, Loss	
LINC01659	22	23,775,750-23,777,258	Loss	AI, Loss	AI, Loss	AI, Loss	AI, Loss	AI, Loss	AI	AI		
LINC01664	22	17,602,484-17,612,994	AI, Loss	AI, Loss		AI, Loss		AI		Loss		
LINC01665	22	17,227,758-17,229,328	Loss	AI, Loss		Loss	Gain	AI		AI, Loss		
LINC02554	22	27,706,611-27,713,801	Loss	AI, Loss	Loss	AI, Loss	AI, Loss	AI, Loss	AI	AI, Loss	AI, Loss	
LOC100128946	22	49,262,581-49,294,198	AI, Loss	AI, Loss	AI, Loss	AI, Loss	AI, Loss	AI, Loss	AI	AI, Loss	AI, Loss	
LOC100130899	22	40,428,335-40,432,581	AI, Loss	AI, Loss	AI, Loss	AI, Loss	AI, Loss	AI, Loss	AI	AI, Loss	Loss	
LOC100286925	22	22,292,608-22,297,805	AI, Loss	AI, Loss	AI, Loss	AI, Loss	AI, Loss	AI, Loss	Loss	AI, Loss		
LOC100506241	22	37,361,288-37,364,213	AI, Loss	AI, Loss	AI, Loss	AI, Loss	AI, Loss	AI, Loss	AI	Loss	AI, Loss	
LOC100506714	22	45,529,638-45,559,662	AI, Loss	AI, Loss	AI, Loss	AI, Loss	AI, Loss	AI, Loss	Loss	AI, Loss	AI, Loss	
LOC100507599	22	26,908,497-26,910,533	AI, Loss	AI, Loss	AI, Loss	AI, Loss	AI, Loss	AI, Loss	AI	AI, Loss	AI, Loss	
LOC101927051	22	38,037,838-38,054,384	Loss	Loss	Loss	Loss	Loss	Loss	AI	Loss	AI, Loss	
LOC101929372	22	18,062,922-18,071,958	AI, Loss	Loss		Loss		AI		AI, Loss		
LOC101929829	22	42,522,500-42,526,883	Loss	Loss	Loss	AI, Loss	AI, Loss	Loss	AI	AI, Loss	Loss	
LOC102724728	22	21,480,427-21,482,309	Loss	Loss		AI, Loss		AI, Loss	AI	AI, Loss		
LOC105372977	22	27,583,477-27,585,177	Loss	AI, Loss		AI, Loss	AI, Loss	AI, Loss	AI	AI, Loss	AI, Loss	
LOC105372990	22	30,828,672-30,832,488	Loss	Loss	AI, Loss	AI, Loss	AI, Loss	AI, Loss	AI	AI, Loss	AI, Loss	
LOC105373021	22	37,099,920-37,163,133	AI, Loss	AI, Loss	AI, Loss	AI, Loss	AI, Loss	Loss	AI	Loss	AI, Loss	

LOC105373031	22	38,966,504-38,967,363	Loss	Loss	Loss	Loss	AI, Loss	Loss	Loss	Loss	Loss	
LOC105373051	22	43,608,679-43,609,667	AI, Loss	Loss	Loss	Loss	AI, Loss	Loss	AI	AI	AI, Loss	
LOC105373064	22	45,572,122-45,587,493	AI, Loss	AI, Loss	AI, Loss	AI, Loss	AI, Loss	AI, Loss	AI, Loss	AI, Loss	AI, Loss	
LOC105373100	22	51,174,256-51,176,597	Loss	Loss	Loss	Loss	AI, Loss		AI		Loss	
LOC110091768	22	27,256,173-27,277,748		Loss	AI	AI, Loss	AI, Loss	AI, Loss	AI	AI, Loss	AI, Loss	
LOC284865	22	20,186,251-20,192,441	Loss	Loss		AI, Loss		AI	AI	AI, Loss		
LOC284889	22	24,235,896-24,241,117	Loss	AI, Loss	Loss	Loss	AI, Loss	AI, Loss	AI, Loss	AI, Loss		
LOC284898	22	27,444,105-27,456,480	Loss	AI, Loss	Loss	AI, Loss	AI, Loss	AI, Loss	AI, Loss	AI, Loss	AI, Loss	
LOC284930	22	48,027,422-48,251,349	AI, Loss	AI, Loss	AI, Loss	AI, Loss	AI, Loss	AI, Loss	AI	AI, Loss	AI, Loss	
LOC284933	22	48,934,711-48,943,199	AI, Loss	AI, Loss	AI, Loss	AI, Loss	AI, Loss	AI, Loss	AI	AI, Loss	AI, Loss	
LOC339666	22	32,772,650-32,780,329	AI, Loss	AI, Loss	AI, Loss	AI, Loss	AI, Loss	AI, Loss	AI	AI, Loss	AI, Loss	
LOC339685	22	47,741,320-47,769,291	AI, Loss	Loss	AI, Loss	AI, Loss	AI, Loss	AI, Loss	AI	AI, Loss	AI, Loss	
LOC388882	22	23,804,431-23,829,167	Loss	AI, Loss	AI, Loss	AI, Loss	AI, Loss	AI, Loss	AI	AI		
LOC391322	22	24,373,100-24,374,043	Loss	AI, Loss	Loss	Loss	AI, Loss	AI, Loss	AI	AI, Loss		
LOC729444	22	20,325,589-20,350,461	Loss	Loss		AI, Loss	Gain	AI	AI	AI, Loss		
LOC730668	22	46,402,495-46,406,657		AI, Loss	AI, Loss	AI, Loss	AI, Loss	AI, Loss	AI, Loss	AI, Loss	AI, Loss	
LOC96610	22	22,652,462-22,677,324	AI, Loss	AI, Loss	AI, Loss	AI, Loss	AI, Loss	AI, Loss	AI	AI, Loss		
LRRC75B	22	24,981,590-24,989,035	AI, Loss	AI, Loss	AI, Loss	AI, Loss	AI, Loss	AI, Loss	Loss	AI, Loss		
LZTR1	22	21,333,750-21,353,326	Loss	Loss		AI, Loss		AI	AI			
MAFF	22	38,597,938-38,612,517		Loss		Loss	AI, Loss	AI	AI	AI	Loss	
MAPK1	22	22,113,946-22,221,970	AI, Loss	AI, Loss	AI, Loss	AI, Loss	AI, Loss	AI, Loss	Loss	AI, Loss		
MAPK11	22	50,702,141-50,709,340	Loss	Loss	AI	AI, Loss	AI, Loss	Loss	AI	Loss		
MAPK12	22	50,688,491-50,700,239	Loss	Loss	AI	AI, Loss	AI, Loss	Loss	AI	Loss		
MAPK8IP2	22	51,039,113-51,052,409	Loss	Loss		Loss	AI, Loss	AI, Loss	AI	AI, Loss	Loss	
MCAT	22	43,528,211-43,539,403	AI, Loss	AI, Loss	Loss	Loss	AI, Loss	AI, Loss	AI	AI	AI, Loss	
MCM5	22	35,796,115-35,820,495	AI, Loss	AI, Loss	AI, Loss	AI, Loss	AI, Loss	AI, Loss	AI	AI	AI, Loss	
MED15	22	20,861,828-20,941,919	Loss	AI, Loss		AI, Loss	AI, Gain	AI		AI, Loss		
MEI1	22	42,095,517-42,195,459	AI, Loss	Loss	AI, Loss	AI, Loss	AI, Loss	Loss	Loss	AI, Loss	Loss	
MFNG	22	37,865,100-37,882,478	Loss	Loss	Loss	Loss	Loss	AI, Loss	AI	AI, Loss	AI, Loss	
MIATNB	22	27,068,805-27,176,856	AI, Loss	AI, Loss	AI, Loss	AI, Loss	AI, Loss	AI, Loss	AI	AI, Loss	AI, Loss	
MICAL3	22	18,270,415-18,507,325	AI, Loss	AI, Loss		Loss		AI	AI	AI, Loss		
MICALL1	22	38,302,154-38,338,465	Loss	Loss	Loss	Loss	AI, Loss	Loss	AI, Loss	Loss	Loss	
MIF	22	24,236,564-24,237,409	Loss	AI, Loss	Loss	Loss	AI, Loss	AI, Loss	AI, Loss	AI, Loss		
MIF-AS1	22	24,235,896-24,241,117	Loss	AI, Loss	Loss	Loss	AI, Loss	AI, Loss	AI, Loss	AI, Loss		
MIOX	22	50,925,212-50,928,750	Loss	Loss	Loss	Loss	AI, Loss	AI, Loss	AI	AI, Loss	Loss	
MIR1249	22	45,596,834-45,596,900	AI, Loss	AI, Loss	AI, Loss	AI, Loss	AI, Loss	AI, Loss	AI	AI, Loss	AI, Loss	
MIR1286	22	20,236,656-20,236,734	Loss	Loss		AI, Loss		AI	AI	AI, Loss		
MIR185	22	20,020,661-20,020,743	Loss	AI, Loss		AI, Loss		AI		AI, Loss		
MIR3198-1	22	18,246,945-18,247,025	Loss	Loss		Loss		AI		AI, Loss		
MIR3199-1	22	28,316,512-28,316,600	AI, Loss	AI, Loss	AI, Loss	AI, Loss	AI, Loss	AI, Loss	Loss	Loss	AI, Loss	
MIR3199-2	22	28,316,513-28,316,599	AI, Loss	AI, Loss	AI, Loss	AI, Loss	AI, Loss	AI, Loss	Loss	Loss	AI, Loss	
MIR3200	22	31,127,543-31,127,628	Loss	Loss	AI, Loss	Loss	AI, Loss	AI, Loss	AI	Loss	AI, Loss	
MIR33A	22	42,296,947-42,297,016	AI, Loss	Loss	AI, Loss	AI, Loss	AI, Loss	Loss	Loss	AI, Loss	Loss	
MIR3653	22	29,729,146-29,729,256	AI, Loss	AI, Loss	AI, Loss	AI, Loss	AI, Loss	AI, Loss	AI	AI, Loss	AI, Loss	

MIR3667	22	49,937,040-49,937,114	AI, Loss	AI, Loss	AI, Loss	AI, Loss	AI, Loss	AI	AI	AI	AI, Loss	
MIR378I	22	42,319,225-42,319,301	AI, Loss	Loss	AI, Loss	AI, Loss	AI, Loss	Loss	Loss	AI, Loss	Loss	
MIR3909	22	35,731,632-35,731,751	AI, Loss	AI, Loss	AI, Loss	AI, Loss	AI, Loss	AI, Loss	AI	AI, Loss	AI, Loss	
MIR3928	22	31,556,047-31,556,105	Loss	AI, Loss	AI, Loss	Loss	AI, Loss	AI, Loss	AI	Loss		
MIR4761	22	19,951,275-19,951,357	AI, Loss	AI, Loss		AI, Loss	Gain	AI		AI, Loss		
MIR4762	22	46,156,403-46,156,478	Loss	AI, Loss	AI, Loss	AI, Loss	AI, Loss	AI, Loss	AI, Loss	AI, Loss	AI, Loss	
MIR4764	22	33,832,567-33,832,655	AI, Loss	AI, Loss	AI, Loss	AI, Loss	AI, Loss	AI, Loss	AI	AI, Loss	AI, Loss	
MIR548J	22	26,951,177-26,951,289	AI, Loss	AI, Loss	AI, Loss	AI, Loss	AI, Loss	AI	AI	AI, Loss	AI, Loss	
MIR6069	22	35,732,713-35,732,792	AI, Loss	AI, Loss	AI, Loss	AI, Loss	AI, Loss	AI, Loss	AI	AI, Loss	AI, Loss	
MIR658	22	38,240,278-38,240,378	Loss	Loss	Loss	Loss	AI, Loss	Loss	Loss	Loss	Loss	
MIR659	22	38,243,684-38,243,781	Loss	Loss	Loss	Loss	AI, Loss	Loss	Loss	Loss	Loss	
MIR6816	22	20,102,208-20,102,274	Loss			AI, Loss	Gain	AI		AI, Loss		
MIR6818	22	30,403,037-30,403,102	AI, Loss	AI, Loss	AI, Loss	AI, Loss	AI, Loss	AI, Loss	Loss	AI, Loss	AI, Loss	
MIR6819	22	36,682,892-36,682,953				AI, Loss	AI, Loss	AI, Loss	AI	AI, Loss	AI, Loss	
MIR6889	22	41,648,995-41,649,054	AI, Loss	AI, Loss	AI, Loss	AI, Loss	AI, Loss	Loss		AI, Loss		
MKL1	22	40,806,291-41,032,690	AI, Loss	AI, Loss	AI, Loss	AI, Loss	AI, Loss	AI, Loss	AI, Loss	AI, Loss	Loss	
MLC1	22	50,497,819-50,524,358	Loss	Loss	AI, Loss	AI, Loss	AI, Loss	AI, Loss	AI		AI, Loss	
MN1	22	28,144,264-28,197,486	AI, Loss			AI, Loss	AI, Loss	AI, Loss	AI	AI, Loss	AI, Loss	
MORC2	22	31,321,116-31,364,273	Loss	Loss	AI, Loss	Loss	AI, Loss	AI, Loss	AI	Loss	AI, Loss	
MOV10L1	22	50,528,434-50,600,116	Loss	Loss	AI, Loss	AI, Loss	AI, Loss	AI, Loss	AI	Loss	AI, Loss	
MPPED1	22	43,808,019-43,902,800	Loss	Loss	AI, Loss	Loss	AI, Loss	AI, Loss	AI	Loss	AI, Loss	
MRTFA	22	40,806,284-41,032,723	AI, Loss	AI, Loss	AI, Loss	AI, Loss	AI, Loss	AI, Loss	AI, Loss	AI, Loss	Loss	
MTFP1	22	30,805,175-30,825,041	Loss	Loss	AI, Loss	AI, Loss	AI, Loss	AI, Loss	AI	AI, Loss	AI, Loss	
MTMR3	22	30,279,157-30,426,857	AI, Loss	AI, Loss	AI, Loss	AI, Loss	AI, Loss	AI, Loss	AI, Loss	AI, Loss	AI, Loss	
MYH9	22	36,677,322-36,784,112				AI, Loss	AI, Loss	AI, Loss	AI	AI, Loss	AI, Loss	
MYO18B	22	26,138,110-26,427,007	AI, Loss	AI, Loss	AI, Loss	AI, Loss	AI, Loss	AI, Loss	AI, Loss	AI, Loss	AI, Loss	
NCAPH2	22	50,946,644-50,963,209	Loss	Loss	Loss	Loss	AI, Loss	AI, Loss	AI	AI, Loss	Loss	
NCF4	22	37,257,029-37,274,059	AI, Loss	Loss	AI, Loss	AI, Loss	AI, Loss	AI	AI	AI	AI, Loss	
NCF4-AS1	22	37,243,414-37,266,485	AI, Loss	AI, Loss	AI, Loss	AI, Loss	AI, Loss	AI	AI	AI	AI, Loss	
NDUFA6	22	42,481,529-42,486,776	Loss	Loss	Loss	AI, Loss	AI, Loss	Loss	AI	AI, Loss	Loss	
NDUFA6-AS1	22	42,486,936-42,532,702	Loss	Loss	Loss	AI, Loss	AI, Loss	Loss	AI	AI, Loss	Loss	
NDUFA6-DT	22	42,486,936-42,521,354	Loss	Loss	Loss	AI, Loss	AI, Loss	Loss	AI	AI, Loss	Loss	
NEFH	22	29,876,180-29,887,277	AI, Loss	AI, Loss	AI, Loss	AI, Loss	AI, Loss	AI, Loss	AI	AI, Loss	AI, Loss	
NF2	22	29,999,544-30,094,589	AI, Loss	AI, Loss	AI, Loss	AI, Loss	AI, Loss	AI, Loss	AI	AI, Loss	AI, Loss	
NFAM1	22	42,776,413-42,828,401	Loss	Loss	Loss	Loss	AI, Loss	AI, Loss	AI	AI, Loss	Loss	
NIPSNAP1	22	29,950,797-29,977,326	AI, Loss	AI, Loss	AI, Loss	AI, Loss	AI, Loss	AI, Loss	AI	AI, Loss	AI, Loss	
NOL12	22	38,082,343-38,089,485	Loss	Loss	Loss	Loss	Loss	Loss	AI	Loss	AI, Loss	
NPTXR	22	39,214,455-39,240,017	Loss	Loss		Loss	AI, Loss	AI, Loss	AI		Loss	
NUP50	22	45,559,725-45,583,890	AI, Loss	AI, Loss	AI, Loss	AI, Loss	AI, Loss	AI, Loss	Loss	AI, Loss	AI, Loss	
NUP50-DT	22	45,529,638-45,559,662	AI, Loss	AI, Loss	AI, Loss	AI, Loss	AI, Loss	AI, Loss	Loss	AI, Loss	AI, Loss	
OSBP2	22	31,089,768-31,303,811	Loss	Loss	AI, Loss	Loss	AI, Loss	AI, Loss	AI	Loss	AI, Loss	
OSM	22	30,658,816-30,662,832	AI, Loss	AI, Loss		AI, Loss	AI, Loss	AI, Loss	AI	AI, Loss	AI, Loss	
P2RX6	22	21,369,441-21,382,302	Loss	Loss		AI, Loss		AI	AI			
P712P	22	16,274,557-16,278,600	Loss			Loss		AI		AI, Loss		

PACSIN2	22	43,265,771-43,411,182	AI, Loss	AI, Loss	Loss	Loss	AI, Loss		AI	AI, Loss	AI, Loss	
PARVB	22	44,395,090-44,565,112	AI, Loss	AI, Loss	AI, Loss	AI, Loss	AI, Loss	AI, Loss	AI	AI, Loss	AI, Loss	
PARVG	22	44,568,835-44,604,349	Loss	AI, Loss	AI, Loss	AI, Loss	AI, Loss	AI	AI		AI, Loss	
PATZ1	22	31,721,789-31,742,249	Loss	AI, Loss	AI, Loss	Loss	AI, Loss	AI, Loss	AI	Loss		
PDGFB	22	39,619,363-39,641,060	AI, Loss	AI, Loss	AI	Loss	Loss	AI	AI	AI, Loss		
PDXP	22	38,054,736-38,062,939	Loss	Loss	Loss	Loss	Loss	Loss	AI	Loss	AI, Loss	
PHF21B	22	45,277,042-45,405,809	AI, Loss	Loss	Loss	AI, Loss	Loss	AI, Loss	AI	AI, Loss	AI, Loss	
PI4KA	22	21,061,978-21,213,100	Loss	AI, Loss		AI, Loss	AI	AI	AI	Loss		
PI4KAP1	22	20,383,730-20,427,815	Loss	Loss		AI, Loss	Gain	AI	AI	AI, Loss		
PIK3IP1	22	31,677,578-31,688,520	Loss	AI, Loss	AI, Loss	Loss	AI, Loss	AI, Loss	AI	Loss		
PIK3IP1-AS1	22	31,731,334-31,734,007	Loss	AI, Loss	AI, Loss	Loss	AI, Loss	AI, Loss	AI	Loss		
PISD	22	32,014,476-32,058,550	AI, Loss	AI, Loss	AI, Loss	Loss	AI, Loss	AI, Loss	Loss	Loss		
PITPNB	22	28,247,656-28,315,294	AI, Loss	AI, Loss	AI, Loss	AI, Loss	AI, Loss	AI, Loss	Loss	Loss	AI, Loss	
PIWIL3	22	25,115,000-25,170,687	AI, Loss	AI, Loss	AI, Loss	AI, Loss	AI, Loss	AI, Loss	AI, Loss	AI, Loss		
PKDREJ	22	46,651,559-46,659,219	Loss	Loss	Loss	AI, Loss	AI, Loss	Loss	Loss	Loss	AI, Loss	
PLA2G3	22	31,530,792-31,536,593	Loss	AI, Loss	AI, Loss	Loss	AI, Loss	AI, Loss	AI	Loss	Loss	
PLA2G6	22	38,507,501-38,577,916	Loss	Loss	Loss	Loss	AI, Loss	AI, Loss	AI	AI, Loss	Loss	
PLXNB2	22	50,713,407-50,746,001	Loss	Loss	AI	AI, Loss	AI, Loss	Loss	AI	Loss	Loss	
PNPLA3	22	44,319,618-44,360,433	AI, Loss	Loss	AI, Loss	AI, Loss	AI, Loss	AI, Loss	AI	AI, Loss	AI, Loss	
PNPLA5	22	44,275,557-44,287,893	AI, Loss	AI, Loss	AI, Loss	AI, Loss	AI, Loss	AI, Loss	AI	AI, Loss	AI, Loss	
POLDIP3	22	42,979,726-43,010,968		Loss		Loss	AI, Loss		AI	AI, Loss	Loss	
POM121L10P	22	25,041,132-25,055,114	AI, Loss	AI, Loss	AI, Loss	AI, Loss	AI, Loss	AI, Loss	Loss	AI, Loss		
POM121L7	22	21,477,049-21,482,095	Loss	Loss		AI, Loss		AI, Loss	AI	AI, Loss		
POM121L7	22	21,637,768-21,638,950	Loss	Loss		AI, Loss		AI, Loss	AI	AI, Loss		
POM121L8P	22	21,636,713-21,652,015	Loss	Loss		AI, Loss		AI, Loss	AI	AI, Loss		
POM121L9P	22	24,647,588-24,661,492	AI, Loss	AI, Loss	AI, Loss	AI, Loss	AI, Loss	AI, Loss	AI	AI, Loss		
POTEH-AS1	22	16,274,608-16,277,577	Loss			Loss		AI		AI, Loss		
PPARA	22	46,546,436-46,639,653	Loss	AI, Loss	Loss	AI, Loss	AI, Loss	AI, Loss	Loss	AI, Loss	AI, Loss	
PPIL2	22	22,020,272-22,052,202	AI, Loss	AI, Loss	AI, Loss	AI, Loss	AI, Loss	AI, Loss	Loss	AI, Loss		
PPM1F	22	22,273,791-22,307,250	AI, Loss	AI, Loss	AI, Loss	AI, Loss	AI, Loss	AI, Loss	Loss	AI, Loss		
PPP6R2	22	50,781,745-50,883,518	Loss	Loss	AI	AI, Loss	AI, Loss		AI		Loss	
PRAMENP	22	22,345,495-22,398,332	AI, Loss	AI, Loss	AI, Loss	AI, Loss	AI, Loss	AI, Loss	AI, Loss	AI, Loss		
PRODH	22	18,900,286-18,924,066	AI, Loss	AI, Loss		Loss		AI	AI	AI, Loss		AI
PRR5	22	45,064,426-45,133,561	AI, Loss	AI, Loss	AI, Loss	AI, Loss	AI, Loss	AI, Loss	AI	AI, Loss	AI, Loss	
PRR5-ARHGAP8	22	45,098,077-45,258,664	AI, Loss	AI, Loss	AI, Loss	AI, Loss	AI, Loss	AI, Loss	AI	AI, Loss	AI, Loss	
PVALB	22	37,196,744-37,215,517	AI, Loss	AI, Loss	AI, Loss	AI, Loss	AI, Loss	AI, Loss	AI	AI, Loss	AI, Loss	
RAB36	22	23,487,510-23,507,830	AI, Loss	AI, Loss	AI, Loss	AI, Loss	AI, Loss	AI, Loss	AI	AI		
RABL2B	22	51,205,919-51,222,110	Loss	Loss	Loss	Loss	AI, Loss		AI		Loss	
RAC2	22	37,621,300-37,640,339	Loss	Loss	Loss	Loss	AI, Loss	AI, Loss	AI	AI, Loss	AI, Loss	
RANBP1	22	20,103,460-20,114,880	Loss			AI, Loss	Gain	AI		AI, Loss		
RANGAP1	22	41,640,780-41,698,217	AI, Loss	AI, Loss	AI, Loss	AI, Loss	AI, Loss	Loss		AI, Loss		
RASD2	22	35,936,540-35,950,045	Loss	AI, Loss	AI, Loss	AI, Loss	AI, Loss	AI, Loss	AI	Loss	AI, Loss	
RASL10A	22	29,708,921-29,711,745	AI, Loss	AI, Loss	AI, Loss	AI, Loss	AI, Loss	AI, Loss	Loss	AI, Loss	AI, Loss	
RBFOX2	22	36,134,780-36,424,585	Loss	Loss	Loss	AI, Loss	AI, Loss	AI, Loss	AI, Loss	AI, Loss	AI, Loss	

RFPL1	22	29,834,571-29,838,444	AI, Loss	AI, Loss	AI, Loss	AI, Loss	AI, Loss	AI, Loss	AI	AI, Loss	AI, Loss	
RFPL1S	22	29,833,003-29,838,118	AI, Loss	AI, Loss	AI, Loss	AI, Loss	AI, Loss	AI, Loss	AI	AI, Loss	AI, Loss	
RFPL2	22	32,586,421-32,600,836	AI, Loss	AI, Loss	AI, Loss	AI, Loss	AI, Loss	AI, Loss	Loss	AI, Loss	AI, Loss	
RFPL3	22	32,750,871-32,757,148	AI, Loss	AI, Loss	AI, Loss	AI, Loss	AI, Loss	AI, Loss	AI	AI, Loss	AI, Loss	
RFPL3S	22	32,755,892-32,767,251	AI, Loss	AI, Loss	AI, Loss	AI, Loss	AI, Loss	AI, Loss	AI	AI, Loss	AI, Loss	
RGL4	22	24,032,960-24,041,363	Loss	AI, Loss	AI, Loss	AI, Loss	AI, Loss	AI, Loss	AI	Loss		
RHBDD3	22	29,655,840-29,664,152	AI, Loss	AI, Loss	AI, Loss	AI, Loss	AI, Loss	AI, Loss	Loss	AI, Loss	AI, Loss	
RIBC2	22	45,809,571-45,828,302	Loss	Loss	Loss	AI, Loss	AI, Loss	AI, Loss	AI, Loss	AI, Loss	AI, Loss	
RIMBP3	22	20,455,993-20,461,786	Loss	Loss		AI, Loss	Gain	AI	AI	AI, Loss		
RIMBP3C	22	21,737,662-21,743,455	Loss	Loss		AI, Loss		AI, Loss	AI	AI, Loss		
RNF185	22	31,556,137-31,603,005	Loss	AI, Loss	AI, Loss	Loss	AI, Loss	AI, Loss	AI	Loss		
RNF215	22	30,774,802-30,783,302	Loss	Loss	AI, Loss	AI, Loss	AI, Loss	AI, Loss	AI	AI, Loss	AI, Loss	
RPL23AP82	22	51,195,513-51,238,065	Loss	Loss	Loss	Loss	AI, Loss		AI		Loss	
RPS19BP1	22	39,925,097-39,928,860	Loss	AI, Loss	AI, Loss	Loss	AI, Loss	AI, Loss	AI	AI, Loss	Loss	
RRP7B	22	42,951,228-42,978,017		Loss		Loss	AI, Loss		AI	AI, Loss	Loss	
RRP7BP	22	42,969,265-42,978,017		Loss		Loss	AI, Loss		AI	AI, Loss	Loss	
RSPH14	22	23,401,592-23,484,241	AI, Loss	AI, Loss	AI, Loss	AI, Loss	AI, Loss	AI, Loss	AI	AI		
RTCB	22	32,783,561-32,808,274	AI, Loss	AI, Loss	AI, Loss	AI, Loss	AI, Loss	AI, Loss	AI, Loss	AI, Loss	AI, Loss	
RTDR1	22	23,401,592-23,484,241	AI, Loss	AI, Loss	AI, Loss	AI, Loss	AI, Loss	AI, Loss	AI	AI		
RTL10	22	19,833,660-19,842,371	AI, Loss	AI, Loss		AI, Loss		AI	AI	AI, Loss		
RTL6	22	44,888,449-44,894,005	AI, Loss	AI, Loss	AI, Loss	AI, Loss	AI, Loss	AI, Loss	AI	AI, Loss	AI, Loss	
RTN4R	22	20,228,937-20,255,816	Loss	Loss		AI, Loss		AI	AI	AI, Loss		
SAMM50	22	44,351,260-44,392,412	AI, Loss		AI, Loss	AI, Loss	AI, Loss	AI, Loss	AI	AI, Loss	AI, Loss	
SBF1	22	50,883,430-50,913,500	Loss		AI	AI, Loss	AI, Loss	AI, Loss	AI	AI, Loss		
SCUBE1	22	43,599,228-43,739,394	AI, Loss	Loss	Loss	Loss	AI, Loss	AI, Loss	AI	AI, Loss	AI, Loss	
SDC4P	22	30,877,276-30,877,743	Loss	Loss	AI, Loss	Loss	AI, Loss	AI, Loss	AI	AI, Loss	AI, Loss	
SEC14L2	22	30,792,929-30,821,291	Loss	Loss	AI, Loss	AI, Loss	AI, Loss	AI, Loss	AI	AI, Loss	AI, Loss	
SEC14L3	22	30,855,215-30,868,034	Loss	Loss	AI, Loss	Loss	AI, Loss	AI, Loss	AI	AI, Loss	AI, Loss	
SEC14L4	22	30,884,897-30,901,698	Loss	Loss	AI, Loss	Loss	AI, Loss	AI, Loss	AI	AI, Loss	AI, Loss	
SEC14L6	22	30,918,774-30,942,669	Loss	Loss	AI, Loss	Loss	AI, Loss	AI, Loss	AI	AI, Loss	AI, Loss	
SELENOO	22	50,639,407-50,656,045				AI, Loss	AI, Loss	AI, Loss	AI	Loss	AI, Loss	
SELO	22	50,639,407-50,656,045				AI, Loss	AI, Loss	AI, Loss	AI	Loss	AI, Loss	
SEPT5-GP1BB	22	19,701,986-19,712,297	AI, Loss	AI, Loss		AI, Loss		AI	AI	AI, Loss		
SERHL2	22	42,949,867-42,970,388		Loss		Loss	AI, Loss		AI	AI, Loss	Loss	
SERPIND1	22	21,128,382-21,142,008	Loss	AI, Loss		AI, Loss	AI	AI	AI	Loss		
SEZ6L	22	26,565,439-26,779,563	AI, Loss	AI, Loss	AI, Loss	AI, Loss	AI, Loss	AI, Loss	AI	AI, Loss	AI, Loss	
SFI1	22	31,892,124-32,014,537	AI, Loss	AI, Loss	AI, Loss	Loss	AI, Loss	AI, Loss	Loss	Loss	Loss	
SGSM1	22	25,202,135-25,322,813	AI, Loss	AI, Loss	AI, Loss	AI, Loss	AI, Loss	AI, Loss	AI	AI, Loss		
SH3BP1	22	38,030,660-38,062,939	Loss	Loss	Loss	Loss	Loss	Loss	AI	Loss	AI, Loss	
SHANK3	22	51,113,069-51,171,640	Loss	Loss	Loss	Loss	AI, Loss	AI, Loss	AI	AI, Loss	Loss	
SHISA8	22	42,305,539-42,311,099	AI, Loss	Loss	AI, Loss	AI, Loss	AI, Loss	Loss	Loss	AI, Loss	Loss	
SHISAL1	22	44,639,556-44,708,731	AI, Loss	AI, Loss		AI, Loss	AI, Loss	AI, Loss	AI	AI, Loss	AI, Loss	
SLC25A1	22	19,163,087-19,166,376	AI, Loss	AI, Loss		AI, Loss		AI	AI	Loss		
SLC25A18	22	18,043,138-18,073,656	AI, Loss	Loss		Loss		AI		AI, Loss		

SLC2A11	22	24,198,889-24,228,299	Loss	AI, Loss	Loss	Loss	AI, Loss	AI, Loss	AI, Loss	AI, Loss		
SLC5A1	22	32,439,018-32,509,011	AI, Loss	AI, Loss	AI, Loss	AI, Loss	AI, Loss	AI, Loss	AI, Loss	AI, Loss	AI, Loss	
SLC5A4	22	32,614,462-32,651,318	AI, Loss	AI, Loss	AI, Loss	AI, Loss	AI, Loss	AI, Loss	AI, Loss	AI, Loss	AI, Loss	
SLC5A4-AS1	22	32,601,101-32,669,602	AI, Loss	AI, Loss	AI, Loss	AI, Loss	AI, Loss	AI, Loss	AI, Loss	AI, Loss	AI, Loss	
SLC7A4	22	21,383,006-21,386,847	Loss	Loss		AI, Loss		AI	AI			
SMC1B	22	45,739,944-45,809,500	Loss	Loss	Loss	AI, Loss	AI, Loss	AI, Loss	AI, Loss	AI, Loss	AI, Loss	
SMTN	22	31,477,281-31,500,610	Loss	Loss	AI, Loss	Loss	AI, Loss	AI, Loss	AI	Loss	AI, Loss	
SNORA50	22	34,100,771-34,100,908	Loss	AI, Loss	AI, Loss	AI, Loss	AI, Loss		AI	AI, Loss	AI, Loss	
SNORA50B	22	34,100,771-34,100,906	Loss	AI, Loss	AI, Loss	AI, Loss	AI, Loss		AI	AI, Loss	AI, Loss	
SNORA77B	22	20,113,924-20,114,049	Loss			AI, Loss	Gain	AI		AI, Loss		
SNORA92	22	38,620,485-38,620,727		Loss		Loss	AI, Loss	AI	AI	AI		
SNORD125	22	29,729,151-29,729,247	AI, Loss	AI, Loss	AI, Loss	AI, Loss	AI, Loss	AI, Loss	AI	AI, Loss	AI, Loss	
SNRPD3	22	24,951,617-24,978,854	AI, Loss	AI, Loss	AI, Loss	AI, Loss	AI, Loss	AI, Loss	AI, Loss	AI, Loss		
SPECC1L	22	24,666,784-24,838,325	AI, Loss	AI, Loss	AI, Loss	AI, Loss	AI, Loss	AI, Loss	AI	AI, Loss		
SPECC1L-ADORA2A	22	24,666,784-24,838,328	AI, Loss	AI, Loss	AI, Loss	AI, Loss	AI, Loss	AI, Loss	AI	AI, Loss		
SREBF2	22	42,229,082-42,303,312	AI, Loss	Loss	AI, Loss	AI, Loss	AI, Loss	Loss	Loss	AI, Loss	Loss	
SREBF2-AS1	22	42,227,218-42,230,669	AI, Loss	Loss	AI, Loss	AI, Loss	AI, Loss	Loss	Loss	AI, Loss	Loss	
SRRD	22	26,879,845-26,887,904	AI, Loss	AI, Loss	AI, Loss	AI, Loss	AI, Loss	AI, Loss	AI	AI, Loss	AI, Loss	
SULT4A1	22	44,220,386-44,258,378	Loss	AI	AI, Loss	AI, Loss	AI	AI, Loss	AI	AI, Loss	AI, Loss	
SUN2	22	39,130,718-39,190,161	Loss	Loss	Loss	Loss	AI, Loss	AI, Loss	AI		Loss	
SYN3	22	32,908,539-33,454,377	AI, Loss	AI, Loss	AI, Loss	AI, Loss	AI, Loss	AI, Loss	AI, Loss	AI, Loss	AI, Loss	
TANGO2	22	20,004,522-20,054,687	Loss	AI, Loss		AI, Loss	Gain	AI		AI, Loss		
TBX1	22	19,744,225-19,771,112	AI, Loss	AI, Loss		AI, Loss	Gain	AI	AI	AI, Loss		
TFIP11	22	26,887,205-26,908,472	AI, Loss	AI, Loss	AI, Loss	AI, Loss	AI, Loss	AI, Loss	AI	AI, Loss	AI, Loss	
THAP7	22	21,354,060-21,356,404	Loss	Loss		AI, Loss		AI	AI			
THAP7-AS1	22	21,356,210-21,364,663	Loss	Loss		AI, Loss		AI	AI			
THOC5	22	29,904,155-29,949,736	AI, Loss	AI, Loss	AI, Loss	AI, Loss	AI, Loss	AI, Loss	AI	AI, Loss	AI, Loss	
TMEM121B	22	17,597,188-17,602,257	AI, Loss	AI, Loss		AI, Loss		AI		Loss		
TMEM184B	22	38,615,297-38,669,040	Loss	Loss		Loss	AI, Loss	AI	AI	AI	Loss	
TMEM191B	22	20,377,668-20,380,440	Loss	Loss		AI, Loss	Gain	AI	AI	AI, Loss		
TMEM211	22	25,331,064-25,342,662	AI, Loss	AI, Loss	AI, Loss	AI, Loss	AI, Loss	AI, Loss	AI	AI, Loss		
TNFRSF13C	22	42,321,035-42,322,821	AI, Loss	Loss	AI, Loss	AI, Loss	AI, Loss	Loss	Loss	AI, Loss	Loss	
TNRC6B	22	40,440,820-40,731,812	AI, Loss	AI, Loss	AI, Loss	AI, Loss	AI, Loss	AI, Loss	AI	AI, Loss	Loss	
TOM1	22	35,695,267-35,743,987	AI, Loss	AI, Loss	AI, Loss	AI, Loss	AI, Loss	AI, Loss	AI	AI, Loss	AI, Loss	
TOP1P2	22	25,160,467-25,161,986	AI, Loss	AI, Loss	AI, Loss	AI, Loss	AI, Loss	AI, Loss	AI	AI, Loss		
TOP3B	22	22,311,396-22,337,240	AI, Loss	AI, Loss	AI, Loss	AI, Loss	AI, Loss	AI, Loss	Loss	AI, Loss		
TPST2	22	26,917,963-26,986,105	AI, Loss	AI, Loss	AI, Loss	AI, Loss	AI, Loss	AI, Loss	AI, Loss	AI, Loss	AI, Loss	
TPTEP1	22	17,082,800-17,179,521	Loss	AI, Loss		Loss		AI		AI, Loss		
TRIOBP	22	38,082,343-38,172,563	Loss	Loss	Loss	Loss	AI, Loss	Loss	AI, Loss	Loss	AI, Loss	
TRMT2A	22	20,099,388-20,104,818	Loss			AI, Loss	Gain	AI		AI, Loss		
TRNA_SeC	22	44,546,536-44,546,622		AI, Loss	AI, Loss	AI, Loss	AI, Loss	AI	AI	AI, Loss	AI, Loss	
TSPO	22	43,547,519-43,559,248	AI, Loss	AI, Loss	Loss	Loss	AI, Loss	AI, Loss	AI	AI	AI, Loss	
TTC28-AS1	22	28,315,363-28,404,267	AI, Loss	AI, Loss	AI, Loss	AI, Loss	AI, Loss	AI, Loss	Loss	Loss	AI, Loss	
TTC38	22	46,663,860-46,689,905	Loss	Loss	Loss	AI, Loss	AI, Loss	Loss	Loss	Loss	AI, Loss	

TTLL12	22	43,562,627-43,583,137	AI, Loss	AI, Loss	Loss	Loss	AI, Loss	AI, Loss	AI	AI	AI, Loss	
TUBA3FP	22	21,362,495-21,368,576	Loss	Loss		AI, Loss		AI	AI			
TUBGCP6	22	50,656,117-50,683,400	Loss	Loss	AI, Loss	AI, Loss	AI, Loss	AI, Loss	AI		AI, Loss	
TUG1	22	31,365,196-31,375,380	Loss	Loss	AI, Loss	Loss	AI, Loss	AI, Loss	AI	Loss	AI, Loss	
TXNRD2	22	19,863,039-19,929,515	AI, Loss	AI, Loss		AI, Loss		AI		AI, Loss		
U6	22	46,020,410-46,020,512	Loss	AI		AI, Loss		AI, Loss	AI	AI	AI, Loss	
UNQ2565	22	24,647,952-24,649,256	AI, Loss	AI, Loss	AI, Loss	AI, Loss	AI, Loss	AI, Loss	AI	AI, Loss		
UPB1	22	24,863,205-24,922,553	AI, Loss	AI, Loss	AI, Loss	AI, Loss	AI, Loss	AI, Loss	AI	AI, Loss		
UPK3A	22	45,680,867-45,691,755	AI, Loss	AI, Loss	AI, Loss	AI, Loss	AI, Loss	AI, Loss	AI, Loss	AI, Loss	AI, Loss	
UQCR10	22	30,163,357-30,166,402	AI, Loss	AI, Loss	AI, Loss	AI, Loss	AI, Loss	AI, Loss	AI	AI, Loss	AI, Loss	
USP41	22	20,704,867-20,731,542	Loss	Loss		AI, Loss	Gain	AI	AI	AI, Loss		
VPREB1	22	22,599,191-22,599,927	AI, Loss	AI, Loss	AI, Loss	AI, Loss	AI, Loss	AI, Loss	AI	AI, Loss		
WBP2NL	22	42,394,728-42,454,460	AI, Loss	Loss	AI, Loss	AI, Loss	AI, Loss		AI	AI, Loss	Loss	
WNT7B	22	46,316,247-46,373,008	Loss	AI, Loss	AI, Loss	AI, Loss	AI, Loss	AI, Loss	AI, Loss	AI, Loss	AI, Loss	
XBP1	22	29,190,547-29,196,560	AI, Loss	AI, Loss	AI, Loss	AI, Loss	AI, Loss	AI, Loss	AI, Loss	AI, Loss	AI, Loss	
XKR3	22	17,264,305-17,302,589	Loss	AI, Loss		Loss	Gain	AI		AI, Loss		
Y_RNA	22	19,032,768-19,032,880	AI, Loss	AI, Loss		Loss		AI	AI	Loss		
Y_RNA	22	31,626,056-31,626,158	Loss	AI, Loss	AI, Loss	Loss	AI, Loss	AI, Loss	AI	Loss		
YPEL1	22	22,051,825-22,090,123	AI, Loss	AI, Loss	AI, Loss	AI, Loss	AI, Loss	AI, Loss	Loss	AI, Loss		
ZC3H7B	22	41,697,506-41,756,151	AI, Loss	AI, Loss	AI, Loss	AI, Loss	AI, Loss	Loss		AI, Loss		
ZDHHC8	22	20,119,363-20,135,530	Loss	Loss		AI, Loss	Gain	AI		AI, Loss		
ZDHHC8P1	22	23,732,791-23,744,799	Loss	AI, Loss	AI, Loss	AI, Loss	AI, Loss	AI, Loss	AI	AI		
ZMAT5	22	30,126,944-30,163,000	AI, Loss	AI, Loss	AI, Loss	AI, Loss	AI, Loss	AI, Loss	AI	AI, Loss	AI, Loss	
ZNF74	22	20,748,404-20,762,753	Loss	Loss		AI, Loss	Gain	AI	AI	AI, Loss		
ZNRF3	22	29,279,754-29,453,476	AI, Loss	AI, Loss	AI, Loss	AI, Loss	AI, Loss	AI, Loss	AI, Loss	AI, Loss	AI, Loss	
SEPT6	X	118,749,687-118,827,333				AI, Loss						
5S_rRNA	X	131,072,873-131,072,984				AI, Loss						
7SK	X	13,614,354-13,614,667				AI, Loss						
7SK	X	95,665,430-95,665,781				AI, Loss						
AB062081	X	134,540,020-134,540,794				AI, Loss						
ABCB7	X	74,270,882-74,376,175				AI, Loss						
ABCD1	X	152,990,322-153,010,216				AI						
ACE2	X	15,579,155-15,620,192				AI, Loss						
ACOT9	X	23,719,173-23,761,407				AI, Loss						
ACRC	X	70,797,873-70,833,433				Loss						
ACSL4	X	108,884,558-108,976,632				AI, Loss						
ACTRT1	X	127,184,940-127,186,382				AI, Loss						
ADGRG2	X	19,007,424-19,140,755				AI, Loss						
ADGRG4	X	135,383,121-135,499,047				AI, Loss						
AF070581	X	110,367,492-110,372,431				Loss						
AGTR2	X	115,301,957-115,306,225				AI, Loss						
AIFM1	X	129,263,337-129,299,861				AI, Loss						
AK000470	X	3,771,064-3,781,615				AI, Loss						
AK055694	X	135,991,553-136,075,814				AI, Loss						

AK056105	X	43,036,242-43,085,847				AI, Loss						
AK056524	X	40,671,202-40,704,710				AI, Loss						
AK057519	X	117,973,518-117,991,683				AI, Loss						
AK094280	X	118,599,996-118,602,225				AI, Loss						
AK095439	X	131,352,649-131,566,839				AI, Loss						
AK096379	X	51,942,921-51,946,358				AI, Loss						
AK097803	X	3,782,438-3,799,884				AI, Loss						
AK098783	X	45,707,326-45,710,447				AI, Loss						
AK123758	X	39,868,528-39,871,435				AI						
AK123976	X	119,170,200-119,280,760				AI, Loss						
AK125288	X	91,354,535-91,360,178				AI, Loss						
AK125301	X	73,421,436-73,461,983				AI, Loss						
AK127380	X	114,752,496-114,797,058				AI, Loss						
AK127697	X	45,364,632-45,386,484				AI, Loss						
AK307233	X	153,625,074-153,627,178				AI						
AK310094	X	23,801,293-23,801,569				AI, Loss						
AK311342	X	75,878,197-76,234,957				AI, Loss						
AKAP14	X	119,029,935-119,054,679				AI, Loss						
AKAP17A	X	1,710,485-1,721,411				AI, Loss						
AKAP4	X	49,955,410-49,965,664				AI, Loss						
ALAS2	X	55,035,487-55,057,497				Loss						
ALG13	X	110,924,345-111,003,875				Loss						
AMELX	X	11,311,532-11,318,881				AI, Loss						
AMMECR1	X	109,437,413-109,683,461				AI, Loss						
AMOT	X	112,018,104-112,084,043				AI, Loss						
ANOS1	X	8,496,914-8,700,228				AI, Loss						
AP1S2	X	15,843,928-15,873,137				AI, Loss						
APLN	X	128,779,235-128,788,933				AI, Loss						
APOO	X	23,851,464-23,926,057				AI, Loss					Gain	Gain
APOOL	X	84,258,897-84,348,323				AI, Loss						
AR	X	66,763,873-66,950,461				Loss						
ARAF	X	47,420,498-47,431,320				AI, Loss						
ARHGAP36	X	130,192,215-130,223,859				AI, Loss						
ARHGAP4	X	153,172,829-153,191,714				AI						
ARHGAP6	X	11,155,662-11,683,821				AI, Loss						
ARHGEF6	X	135,747,711-135,863,503				AI, Loss						
ARR3	X	69,488,184-69,501,690				AI, Loss						
ARSF	X	2,958,274-3,030,770				AI, Loss						
ARSH	X	2,924,653-2,951,426				AI, Loss						
ASB11	X	15,299,830-15,333,746				AI, Loss						
ASB12	X	63,444,071-63,450,511				Loss						
ASB9	X	15,262,108-15,288,589				AI, Loss						
ASMT	X	1,714,347-1,761,974				AI, Loss						
ASMTL	X	1,522,031-1,572,655				AI, Loss						

ASMTL-AS1	X	1,519,423-1,534,314				AI, Loss						
ATG4A	X	107,334,896-107,397,901				AI, Loss						
ATP1B4	X	119,495,939-119,517,104				AI, Loss						
ATP2B3	X	152,801,579-152,848,387				AI						
ATP6AP1	X	153,656,977-153,664,863				AI						
ATP6AP2	X	40,440,215-40,465,888				AI, Loss						
ATP7A	X	77,166,152-77,305,892				AI, Loss						
ATRX	X	76,760,355-77,041,755				AI, Loss						
ATXN3L	X	13,336,767-13,338,518				AI, Loss						
AVPR2	X	153,167,984-153,172,620				AI						
AWAT1	X	69,454,504-69,460,511				AI, Loss						
AWAT2	X	69,260,391-69,269,788				AI, Loss						
AX746622	X	16,853,680-16,855,808				AI, Loss						
AX747333	X	46,319,732-46,321,944				AI, Loss						
AX748273	X	50,331,931-50,333,932				AI, Loss						
AX748371	X	74,544,123-74,546,232				AI, Loss						
AY660577	X	10,981,490-11,125,110				AI, Loss						
AY660578	X	10,988,010-11,129,258				AI, Loss						
BC009467	X	153,652,727-153,656,796				AI						
BC015977	X	46,185,713-46,187,080				AI, Loss						
BC018767	X	152,614,942-152,617,045				AI, Loss						
BC028211	X	118,599,996-118,603,061				AI, Loss						
BC029787	X	134,382,887-134,478,012				AI, Loss						
BC039399	X	40,594,651-40,597,946				AI, Loss						
BC043223	X	129,611,042-129,658,231				AI, Loss						
BC046187	X	19,931,205-19,934,282				AI, Loss						
BC061642	X	134,254,548-134,257,529				AI, Loss						
BC067907	X	64,808,260-64,845,760				Loss						
BC070370	X	107,173,842-107,174,868				AI, Loss						
BC094793	X	52,108,069-52,111,918				AI, Loss						
BC132858	X	37,478,968-37,480,588				AI, Loss						
BC137157	X	51,793,955-51,797,392				AI, Loss						
BCAP31	X	152,965,946-152,990,201				AI						
BCLAF3	X	19,930,979-19,988,382				AI, Loss						
BCOR	X	39,910,498-40,036,582				AI, Loss						
BCYRN1	X	70,430,034-70,948,962				Loss						
BEND2	X	18,181,050-18,239,024				AI, Loss						
BEX1	X	102,317,580-102,319,168				AI, Loss						
BEX2	X	102,564,273-102,565,974				AI, Loss						
BEX3	X	102,631,250-102,633,092				AI, Loss						
BEX4	X	102,470,019-102,472,128				AI, Loss						
BGN	X	152,760,346-152,775,012				AI						
BMP15	X	50,653,734-50,659,641				AI, Loss						
BMX	X	15,482,368-15,574,652				AI, Loss						

BRCC3	X	154,299,694-154,351,349				AI, Loss						
BRDTP1	X	95,592,084-95,592,901				AI, Loss						
BRS3	X	135,570,124-135,574,598				AI, Loss						
BRWD3	X	79,924,986-80,065,233				AI, Loss						
C1GALT1C1	X	119,759,528-119,764,005				AI, Loss						
CA5B	X	15,756,411-15,805,748				AI, Loss						
CA5BP1	X	15,693,038-15,721,474				AI, Loss						
CACNA1F	X	49,061,522-49,089,833				Loss						
CAPN6	X	110,488,326-110,513,774				Loss						
CASK	X	41,374,188-41,782,287				AI, Loss						
CBLL2	X	22,291,029-22,292,576				AI, Loss						
CCDC120	X	48,910,960-48,927,510				Loss						
CCDC160	X	133,371,242-133,380,237				AI, Loss						
CCNB3	X	49,969,415-50,094,911				AI, Loss						
CCNQ	X	152,853,382-152,864,632				AI						
CD40LG	X	135,730,335-135,742,549				AI, Loss						
CD99	X	2,609,173-2,659,350				Loss						
CDK16	X	47,077,527-47,089,394				AI, Loss						
CDKL5	X	18,443,724-18,671,749				AI, Loss						
CDX4	X	72,667,089-72,674,421				AI, Loss						
CENPVL1	X	51,424,145-51,425,764				AI, Loss						
CENPVL2	X	51,453,607-51,455,226				AI, Loss						
CENPVL3	X	51,359,873-51,361,770				AI, Loss						
CENPVP1	X	51,424,145-51,425,447				AI, Loss						
CETN2	X	151,995,870-151,999,301				AI, Loss						
CFAP47	X	35,937,850-36,403,434				AI, Loss						
CFP	X	47,483,611-47,489,704				AI						
CHDC2	X	36,065,052-36,163,187				AI, Loss						
CHIC1	X	72,782,983-72,906,944				AI, Loss						
CHM	X	85,116,184-85,302,569				AI, Loss						
CHMP1B2P	X	79,483,987-79,590,817				AI, Loss						
CHRDL1	X	109,917,083-110,039,286				Loss						
CHST7	X	46,433,121-46,457,931				AI, Loss						
CLCN4	X	10,124,984-10,205,699				AI, Loss						
CLCN5	X	49,687,224-49,863,892				AI, Loss						
CLDN2	X	106,143,393-106,174,091				AI, Loss						
CLIC2	X	154,505,495-154,563,990				AI, Loss						
CLTRN	X	15,645,438-15,683,154				AI, Loss						
CMC4	X	154,289,899-154,299,547				AI, Loss						
CNKSR2	X	21,392,415-21,672,813				AI, Loss						
COL4A5	X	107,683,073-107,940,775			Loss	AI, Loss						
COL4A6	X	107,386,779-107,682,727				AI, Loss						
COX7B	X	77,154,964-77,162,873				AI, Loss						
CPXCR1	X	88,002,225-88,009,785				AI, Loss						

CR627394	X	79,483,989-79,565,732				AI, Loss						
CRLF2	X	1,314,886-1,656,037				AI, Loss					Gain	Gain
CS706497	X	91,651,900-91,651,987				AI, Loss						
CSAG1	X	151,903,226-151,909,518				AI, Loss						
CSAG2	X	151,876,742-151,877,747				AI, Loss						
CSAG2	X	151,927,733-151,928,738				AI, Loss						
CSAG3	X	151,876,742-151,883,038				AI, Loss						
CSAG3	X	151,927,733-151,928,738				AI, Loss						
CSAG4	X	151,895,977-151,903,136				AI, Loss						
CSF2RA	X	1,387,692-1,428,828				AI, Loss					Gain	Gain
CT45A1	X	134,847,184-134,891,539				AI, Loss						
CT45A10	X	134,945,650-134,975,579				AI, Loss						
CT45A2	X	134,866,213-134,874,268				AI, Loss						
CT45A3	X	134,883,487-134,891,519				AI, Loss						
CT45A4	X	134,866,213-134,891,519				AI, Loss						
CT45A5	X	134,866,213-134,874,561				AI, Loss						
CT45A5	X	134,945,650-134,971,244				AI, Loss						
CT45A6	X	134,883,487-134,891,519				AI, Loss						
CT45A7	X	134,866,414-134,874,244				AI, Loss						
CT45A8	X	134,866,213-134,874,249				AI, Loss						
CT45A9	X	134,866,213-134,874,608				AI, Loss						
CT47A1	X	120,116,320-120,119,638				AI, Loss						
CT47A10	X	120,011,344-120,066,151				AI, Loss						
CT47A11	X	120,067,694-120,071,012				AI, Loss						
CT47A12	X	120,011,344-120,066,151				AI, Loss						
CT47A2	X	120,072,555-120,075,873				AI, Loss						
CT47A2	X	120,077,415-120,080,733				AI, Loss						
CT47A3	X	120,082,276-120,085,594				AI, Loss						
CT47A4	X	120,067,694-120,071,012				AI, Loss						
CT47A4	X	120,096,880-120,100,198				AI, Loss						
CT47A5	X	120,077,415-120,080,733				AI, Loss						
CT47A5	X	120,101,740-120,105,058				AI, Loss						
CT47A6	X	120,067,694-120,071,012				AI, Loss						
CT47A6	X	120,116,320-120,119,638				AI, Loss						
CT47A7	X	120,087,136-120,090,454				AI, Loss						
CT47A8	X	120,011,344-120,066,151				AI, Loss						
CT47A9	X	120,011,344-120,066,151				AI, Loss						
CT47A9	X	120,106,600-120,109,918				AI, Loss						
CT47B1	X	120,006,451-120,009,779				AI, Loss						
CT55	X	134,290,460-134,305,751				AI, Loss						
CT83	X	115,592,852-115,594,194				AI, Loss						
CTPS2	X	16,606,121-16,731,102				AI, Loss						
CUL4B	X	119,658,445-119,709,684				Loss						
CXorf22	X	35,937,850-36,008,269				AI, Loss						

CXorf23	X	19,930,979-19,988,382				AI, Loss						
CXorf27	X	37,850,069-37,850,570				AI, Loss						
CXorf28	X	3,189,860-3,202,694				AI, Loss						
CXorf30	X	36,246,895-36,403,434				AI, Loss						
CXorf31	X	46,746,853-46,759,139				AI, Loss						
CXorf36	X	45,007,617-45,060,146				AI, Loss						
CXorf38	X	40,486,172-40,506,935				AI, Loss						
CXorf48	X	134,290,460-134,305,751				AI, Loss						
CXorf49	X	70,934,223-70,938,135				Loss						
CXorf49B	X	70,934,223-70,938,135				Loss						
CXorf56	X	118,672,111-118,699,397				AI, Loss						
CXorf57	X	105,855,159-105,922,673				AI, Loss						
CXorf58	X	23,926,122-23,957,624				AI, Loss					Gain	Gain
CXorf61	X	115,592,852-115,594,194				AI, Loss						
CXorf64	X	125,953,746-125,955,768				AI, Loss						
CXorf67	X	51,149,766-51,151,689				AI, Loss						
CXXC1P1	X	47,566,589-47,596,027				AI, Loss						
CYBB	X	37,639,269-37,672,714				AI, Loss						
CYLC1	X	83,116,133-83,141,708				AI, Loss						
CYSLTR1	X	77,526,968-77,583,188				AI, Loss						
DACH2	X	85,403,454-86,087,605				AI, Loss						
DANT1	X	114,957,296-115,021,860				AI, Loss						
DANT2	X	114,956,728-115,085,444				AI, Loss						
DCAF12L1	X	125,683,365-125,686,842				AI, Loss						
DCAF12L2	X	125,297,481-125,300,080				AI, Loss						
DCX	X	110,537,006-110,655,460				Loss						
DD413549	X	42,729,652-42,729,672				AI, Loss						
DD413682	X	91,368,316-91,368,338				AI, Loss						
DDX11L16	X	155,255,322-155,257,848				AI, Loss						
DDX26B	X	134,654,554-134,716,460				AI, Loss						
DDX3X	X	41,192,560-41,223,725				AI, Loss						Gain
DDX53	X	23,018,077-23,020,206				AI, Loss						
DGAT2L6	X	69,397,332-69,425,553				AI, Loss						
DGKK	X	50,108,405-50,213,737				AI, Loss						
DHRSX	X	2,137,554-2,419,015				AI, Loss						
DIAPH2	X	95,939,661-96,855,597				AI, Loss						
DIAPH2-AS1	X	96,783,362-96,819,534				AI, Loss						
DIPK2B	X	45,007,617-45,060,146				AI, Loss						
DKC1	X	153,991,016-154,005,964				AI, Loss						
DKFZp451F083	X	134,382,887-134,477,957				AI, Loss						
DL490658	X	153,209,600-153,209,674				AI						
DLG3	X	69,664,704-69,725,339				AI, Loss						
DLG3-AS1	X	69,672,805-69,675,844				AI, Loss						
DM119510	X	133,303,712-133,303,735				AI, Loss						

DMD	X	31,137,344-33,357,726				AI, Loss	AI, Loss					
DMRTC1	X	72,062,799-72,068,636				AI, Loss						
DMRTC1B	X	72,064,875-72,068,636				AI, Loss						
DNASE1L1	X	153,629,576-153,640,428				AI						
DOCK11	X	117,629,871-117,820,123				AI, Loss						
DQ570500	X	43,137,548-43,137,585				AI, Loss						
DQ570720	X	153,628,902-153,628,928				AI						
DQ574810	X	40,219,039-40,219,066				AI, Loss						
DQ576039	X	800,013-800,044				AI, Loss					Gain	Gain
DQ579129	X	40,219,847-40,219,877				AI, Loss						
DQ580189	X	95,306,294-95,306,323				AI, Loss						
DQ580770	X	9,375,782-9,375,838				AI, Loss						
DQ594696	X	40,218,597-40,218,633				AI, Loss						
DQ595787	X	9,373,384-9,373,450				AI, Loss						
DQ596633	X	40,217,979-40,218,014				AI, Loss						
DQ597811	X	9,374,425-9,374,460				AI, Loss						
DUSP21	X	44,703,248-44,704,134				AI, Loss						
DUSP9	X	152,907,896-152,916,781				AI						
DYNLT3	X	37,698,088-37,706,889				AI, Loss						
EBP	X	48,380,163-48,387,104				Loss						
EDA	X	68,835,910-69,259,321				AI, Loss						
EDA2R	X	65,815,481-65,859,140				Loss						
EFHC2	X	44,007,127-44,202,923				AI, Loss						
EFNB1	X	68,048,839-68,066,029				AI						
EGFL6	X	13,587,693-13,651,694				AI, Loss						
EIF1AX	X	20,142,635-20,159,966				AI, Loss						
EIF1AX-AS1	X	20,158,085-20,158,562				AI, Loss						
EIF2S3	X	24,073,064-24,096,927				AI, Loss					Gain	Gain
ELF4	X	129,198,894-129,244,688				AI, Loss						
ELK1	X	47,494,918-47,510,003				AI						
EMD	X	153,607,596-153,609,883				AI						
ENOX2	X	129,757,356-130,037,291				AI, Loss						
ERCC6L	X	71,424,504-71,458,897				Loss						
ETDA	X	134,386,008-134,387,530				AI, Loss						
ETDB	X	134,252,881-134,254,454				AI, Loss						
ETDC	X	134,443,405-134,443,585				AI, Loss						
F8	X	154,124,351-154,134,848				AI, Loss						
F8	X	154,064,063-154,255,215				AI, Loss						
F8A1	X	154,114,634-154,116,336				AI, Loss						
F8A2	X	154,687,145-154,688,261				AI, Loss						
F8A3	X	154,114,649-154,115,765				AI, Loss						
FAAH2	X	57,313,109-57,515,629				Loss						
FAM104B	X	55,169,534-55,187,628				Loss						
FAM120C	X	54,094,756-54,209,714				AI, Loss						

FAM122B	X	133,903,595-133,931,262				AI, Loss						
FAM122C	X	133,930,424-133,988,641				AI, Loss						
FAM127A	X	134,166,332-134,167,575				AI, Loss						
FAM127B	X	134,184,962-134,186,221				AI, Loss						
FAM127C	X	134,154,533-134,156,566				AI, Loss						
FAM133A	X	92,929,011-92,967,273				AI, Loss						
FAM155B	X	68,725,077-68,752,351				AI, Loss						
FAM156A	X	52,926,321-52,937,585				AI, Loss						
FAM156A	X	52,976,463-53,024,651				AI, Loss						
FAM156B	X	52,920,628-52,937,585				AI, Loss						
FAM226A	X	72,161,567-72,163,589				AI, Loss						
FAM226B	X	72,161,567-72,163,589				AI, Loss						
FAM236A	X	72,001,688-72,002,484				AI, Loss						
FAM236B	X	72,001,688-72,002,484				AI, Loss						
FAM236B	X	72,158,002-72,158,798				AI, Loss						
FAM236C	X	72,027,258-72,028,044				AI, Loss						
FAM236D	X	72,027,258-72,028,044				AI, Loss						
FAM236D	X	72,132,452-72,133,238				AI, Loss						
FAM239A	X	3,769,032-3,800,392				AI, Loss						
FAM239B	X	3,803,315-3,838,783				AI, Loss						
FAM45B	X	129,628,914-129,631,421				AI, Loss						
FAM45BP	X	129,628,914-129,631,421				AI, Loss						
FAM46D	X	79,591,002-79,700,810				AI, Loss						
FAM47C	X	37,026,431-37,029,739				AI, Loss						
FAM50A	X	153,672,472-153,679,002				AI						
FAM58A	X	152,853,382-152,864,632				AI						
FAM9A	X	8,758,836-8,769,424				AI, Loss						
FAM9B	X	8,992,272-9,002,168				AI, Loss						
FAM9C	X	13,053,735-13,062,917				AI, Loss						
FANCB	X	14,861,528-14,891,191				AI, Loss						
FGD1	X	54,471,886-54,522,599				AI, Loss						
FGF16	X	76,709,646-76,712,013				AI, Loss						
FHL1	X	135,228,860-135,293,518				AI, Loss						
FIGF	X	15,363,712-15,402,535				AI, Loss						
FIRRE	X	130,825,555-130,964,671				AI, Loss						
FLICR	X	49,122,682-49,122,937				AI, Loss						
FLJ44635	X	71,364,033-71,381,600				AI						
FLNA	X	153,576,899-153,603,006				AI						
FOXP3	X	49,106,896-49,121,288				AI, Loss						
FOXR2	X	55,649,832-55,652,621				Loss						
FRMD7	X	131,211,013-131,262,050				AI, Loss						
FRMD8P1	X	64,770,501-64,772,301				Loss						
FRMPD3	X	106,765,679-106,848,481				AI, Loss						
FRMPD3-AS1	X	106,756,212-106,789,051				AI, Loss						

FRMPD4	X	12,156,584-12,742,642				AI, Loss					Loss	
FTH1P18	X	37,060,954-37,061,867				AI, Loss						
FTSJ1	X	48,334,408-48,344,752				Loss						
FTX	X	73,247,970-73,513,409				AI, Loss						
FUNDC1	X	44,382,884-44,402,221				AI, Loss						Gain
FUNDC2	X	154,255,063-154,285,191				AI, Loss						
GAB3	X	153,903,526-153,979,858				AI, Loss						
GABRA3	X	151,335,633-151,619,831				AI, Loss						
GABRE	X	151,121,595-151,143,151				AI, Loss						
GABRQ	X	151,806,636-151,821,825				AI, Loss						
GAGE1	X	49,354,117-49,373,139				AI, Loss						
GAGE10	X	49,160,124-49,176,323				AI, Loss						
GAGE12B	X	49,335,037-49,342,296				AI, Loss						
GAGE12C	X	49,335,002-49,342,360				AI, Loss						
GAGE12D	X	49,296,768-49,304,154				AI, Loss						
GAGE12D	X	49,325,433-49,332,821				AI, Loss						
GAGE12D	X	49,344,540-49,351,917				AI, Loss						
GAGE12E	X	49,296,779-49,304,144				AI, Loss						
GAGE12F	X	49,306,301-49,313,700				AI, Loss						
GAGE12G	X	49,306,332-49,313,700				AI, Loss						
GAGE12G	X	49,325,441-49,332,811				AI, Loss						
GAGE12H	X	49,296,779-49,304,144				AI, Loss						
GAGE12I	X	49,335,064-49,342,360				AI, Loss						
GAGE12J	X	49,178,508-49,294,598				AI, Loss						
GAGE13	X	49,188,080-49,323,262				AI, Loss						
GAGE2A	X	49,216,633-49,294,598				AI, Loss						
GAGE2A	X	49,354,117-49,361,430				AI, Loss						
GAGE2B	X	49,235,707-49,242,997				AI, Loss						
GAGE2C	X	49,216,647-49,294,598				AI, Loss						
GAGE2D	X	49,207,115-49,214,430				AI, Loss						
GAGE2D	X	49,226,137-49,233,491				AI, Loss						
GAGE2E	X	49,207,138-49,214,416				AI, Loss						
GAGE4	X	49,216,633-49,294,598				AI, Loss						
GAGE5	X	49,216,656-49,223,943				AI, Loss						
GAGE6	X	49,325,479-49,332,807				AI, Loss						
GAGE7	X	49,216,651-49,223,939				AI, Loss						
GAGE8	X	49,188,103-49,313,700				AI, Loss						
GCNA	X	70,797,873-70,833,433				Loss						
GDI1	X	153,665,258-153,671,814				AI						
GDPD2	X	69,642,880-69,653,241				AI, Loss						
GEMIN8	X	14,024,844-14,048,035				AI, Loss						
GJB1	X	70,435,061-70,445,065				Loss						
GLRA2	X	14,547,419-14,749,933				AI, Loss						
GLRA4	X	102,962,271-102,983,552				AI, Loss						

GLUD2	X	120,181,461-120,183,796				AI, Loss						
GNL3L	X	54,556,643-54,593,720				AI, Loss						
GPC3	X	132,669,775-133,119,673				AI, Loss						
GPC3-AS1	X	133,031,469-133,031,948				AI, Loss						
GPC4	X	132,435,063-132,549,205				AI, Loss						
GPM6B	X	13,789,040-13,956,943				AI, Loss						
GPR101	X	136,112,306-136,113,833				AI, Loss						
GPR112	X	135,383,121-135,499,047				AI, Loss						
GPR119	X	129,518,318-129,519,511				AI, Loss						
GPR143	X	9,693,452-9,734,005				Loss						
GPR174	X	78,426,468-78,430,814				AI, Loss						
GPR34	X	41,548,225-41,556,530				AI, Loss						
GPR64	X	19,007,424-19,140,755				AI, Loss						
GPR82	X	41,583,407-41,589,388				AI, Loss						
GRIA3	X	122,318,095-122,624,766				AI, Loss						
GRIPAP1	X	48,830,130-48,858,675				Loss						
GRPR	X	16,141,423-16,171,641				AI, Loss						
GSPT2	X	51,486,480-51,489,326				AI, Loss						
GUCY2F	X	108,616,134-108,725,285				AI, Loss						
H2AFB1	X	154,689,079-154,689,596				AI, Loss						
H2AFB2	X	154,689,075-154,689,669				AI, Loss						
H2AFB3	X	154,610,427-154,610,944				AI, Loss						
H2AFB3	X	154,689,079-154,689,596				AI, Loss						
HAUS7	X	152,710,177-152,760,983				AI, Loss						
HCCS	X	11,129,405-11,141,204				AI, Loss						
HCFC1	X	153,213,007-153,236,819				AI						
HCFC1-AS1	X	153,234,215-153,235,542				AI						
HDAC8	X	71,549,365-71,792,953				AI, Loss						
HDHD1	X	6,966,960-7,066,231				AI, Loss						
HDX	X	83,572,881-83,757,487				AI, Loss						
HEPH	X	65,382,390-65,487,230				Loss						
HMGN5	X	80,369,199-80,457,441				AI, Loss						
HPRT1	X	133,594,174-133,634,698				AI, Loss						
HS6ST2	X	131,760,037-132,095,423				AI, Loss						
HS6ST2-AS1	X	131,801,669-131,803,915				AI, Loss						
HTATSF1	X	135,579,237-135,594,503				AI, Loss						
HTR2C	X	113,818,550-114,144,624				AI, Loss						
HUWE1	X	53,559,056-53,713,674				AI, Loss						
HYPM	X	37,850,069-37,850,570				AI, Loss						
IDH3G	X	153,051,220-153,059,978				AI						
IGBP1	X	69,353,298-69,386,173				AI, Loss						
IGSF1	X	130,407,482-130,712,873				AI, Loss						
IL13RA1	X	117,861,558-117,928,496				AI, Loss						
IL13RA2	X	114,238,537-114,252,207				AI, Loss						

IL1RAPL2	X	103,810,995-105,011,822				AI, Loss						
IL3RA	X	1,455,508-1,501,582				AI, Loss						Gain
IL9R	X	155,227,245-155,240,482				AI, Loss						
INE2	X	15,803,838-15,805,712				AI, Loss						
INGX	X	70,711,530-70,712,299				Loss						
INTS6L	X	134,654,550-134,716,460				AI, Loss						
INTS6L-AS1	X	134,654,007-134,654,599				AI, Loss						
IRES_Cx43	X	18,552,981-18,553,187				AI, Loss						
IRS4	X	107,975,726-107,979,607				AI, Loss						
ITGB1BP2	X	70,521,583-70,525,221				Loss						
ITIH6	X	54,775,331-54,824,673				AI, Loss						
ITM2A	X	78,615,880-78,623,049				AI, Loss						
JA202257	X	32,834,661-32,834,692				AI, Loss	AI					
JA202295	X	32,503,097-32,503,131				AI, Loss	Loss					
JA202330	X	32,398,676-32,398,705				AI, Loss	AI					
JA202335	X	32,583,973-32,584,010				AI, Loss	AI					
JA611242	X	64,905,377-64,905,400				Loss						
JA611288	X	21,671,685-21,671,755				AI, Loss						
JA783409	X	32,632,483-32,632,519				AI, Loss	AI					
JA783414	X	32,490,280-32,490,302				AI, Loss	Loss					
JA783415	X	32,482,743-32,482,766				AI, Loss	Loss					
JA783489	X	32,867,872-32,867,918				AI, Loss	AI					
JA783498	X	32,862,937-32,862,967				AI, Loss	AI					
JA783500	X	32,862,888-32,862,913				AI, Loss	AI					
JA783507	X	32,834,568-32,834,591				AI, Loss	AI					
JA783513	X	32,827,657-32,827,688				AI, Loss	AI					
JA783516	X	32,717,390-32,717,417				AI, Loss	Loss					
JA783533	X	32,662,333-32,662,381				AI, Loss	Loss					
JA783536	X	32,662,291-32,662,321				AI, Loss	Loss					
JA783548	X	32,563,365-32,563,401				AI, Loss	AI					
JA783560	X	32,486,729-32,486,759				AI, Loss	Loss					
JA783566	X	32,472,915-32,472,956				AI, Loss	AI					
JA783574	X	32,408,163-32,408,193				AI, Loss	AI					
JA783579	X	32,398,725-32,398,757				AI, Loss	AI					
JA783867	X	32,563,416-32,563,458				AI, Loss	AI					
JADE3	X	46,771,710-46,920,641				AI, Loss						
JPX	X	73,164,158-73,290,217				AI, Loss						
KAL1	X	8,496,914-8,700,227				AI, Loss						
KCNE1L	X	108,866,928-108,868,393				AI, Loss						
KCNE5	X	108,866,928-108,868,397				AI, Loss						
KDM6A	X	44,732,420-44,971,857				AI, Loss						
KIAA1210	X	118,212,597-118,284,542				AI, Loss						
KIAA1817	X	106,843,634-106,848,479				AI, Loss						
KIAA2022	X	73,952,690-74,145,287				AI, Loss						

KIF4A	X	69,509,878-69,640,774				AI, Loss							
KLF8	X	55,934,555-56,317,963				Loss							
KLHL13	X	117,031,775-117,251,303				AI, Loss							
KLHL15	X	24,001,832-24,045,303				AI, Loss							Gain
KLHL34	X	21,673,466-21,676,505				AI, Loss							
KLHL4	X	86,772,714-86,925,050				AI, Loss							
KRBOX4	X	46,306,623-46,334,074				AI, Loss							
L1CAM	X	153,126,968-153,151,628				AI							
LAMP2	X	119,560,002-119,603,204				Loss							
LANCL3	X	37,430,821-37,536,750				AI, Loss							
LAS1L	X	64,732,461-64,754,686				Loss							
LCA10	X	153,146,126-153,154,427				AI							
LHFPL1	X	111,873,878-111,923,375				AI, Loss							
LINC00086	X	134,555,867-134,560,225				AI, Loss							
LINC00087	X	134,229,014-134,232,733				AI, Loss							
LINC00106	X	1,515,917-1,518,291				AI, Loss							
LINC00269	X	68,399,399-68,429,767				AI, Loss							
LINC00629	X	133,684,053-133,694,428				AI, Loss							
LINC00630	X	102,024,080-102,219,450				AI, Loss							
LINC00633	X	134,252,881-134,254,405				AI, Loss							
LINC00684	X	72,001,688-72,002,484				AI, Loss							
LINC00890	X	110,754,889-110,765,627				Loss							
LINC00891	X	70,917,045-70,923,256				Loss							
LINC00892	X	135,721,701-135,724,588				AI, Loss							
LINC01186	X	46,185,358-46,187,109				AI, Loss							
LINC01201	X	130,150,442-130,192,120				AI, Loss							
LINC01203	X	13,353,359-13,359,944				AI, Loss							
LINC01204	X	45,364,632-45,386,484				AI, Loss							
LINC01281	X	39,164,209-39,186,616				AI, Loss							Loss
LINC01282	X	39,226,538-39,251,028				AI, Loss							Loss
LINC01284	X	50,838,681-50,914,253				AI, Loss							
LINC01285	X	117,973,518-118,015,977				AI, Loss							
LINC01402	X	119,251,551-119,253,610				AI, Loss							
LINC01456	X	17,988,292-18,122,764				AI, Loss							
LINC01496	X	51,242,760-51,250,293				AI, Loss							
LINC01545	X	46,746,853-46,759,139				AI, Loss							
LINC01546	X	3,189,860-3,202,694				AI, Loss							
LINC01560	X	47,342,114-47,344,626				AI, Loss							
LINC02154	X	13,284,166-13,321,571				AI, Loss							
LINC02243	X	134,083,578-134,087,411				AI, Loss							
LINC02589	X	102,785,694-102,809,881				AI, Loss							
LINC02595	X	45,707,508-45,710,920				AI, Loss							
LINC02601	X	41,134,991-41,136,030				AI, Loss							Gain
LOC100093698	X	13,328,770-13,338,052				AI, Loss							

LOC100126447	X	115,944,898-115,951,828				AI, Loss						
LOC100129291	X	70,882,840-70,886,718				Loss						
LOC100129520	X	124,453,968-124,456,950				AI, Loss						
LOC100132741	X	70,917,045-70,923,256				Loss						
LOC100132831	X	40,690,469-40,692,449				AI, Loss						
LOC100133123	X	13,353,359-13,359,944				AI, Loss						
LOC100133957	X	47,518,231-47,519,776				AI, Loss						
LOC100421746	X	55,306,520-55,315,219				Loss						
LOC100506790	X	134,530,353-134,531,672				AI, Loss						
LOC100873065	X	22,277,913-23,311,263				AI, Loss						
LOC101059915	X	70,887,551-70,891,374				Loss						
LOC101927476	X	40,122,169-40,146,974				AI, Loss						
LOC101927635	X	48,364,915-48,367,227				Loss						
LOC101927830	X	154,696,200-154,723,771				AI, Loss						
LOC101928128	X	84,465,711-84,474,295				AI, Loss						
LOC101928201	X	4,545,240-4,551,613				AI, Loss						
LOC101928335	X	107,137,826-107,179,210				AI, Loss						
LOC101928336	X	118,425,491-118,469,573				AI, Loss						
LOC101928358	X	107,979,769-107,982,133				AI, Loss						
LOC101928389	X	17,546,557-17,605,281				AI, Loss						
LOC101928402	X	123,093,082-123,095,191				AI, Loss						Gain
LOC101928437	X	112,285,954-112,763,885				AI, Loss						
LOC101928495	X	125,243,744-125,249,545				AI, Loss						
LOC101928627	X	36,383,740-36,458,375				AI, Loss						
LOC105373156	X	6,144,398-6,148,332				AI, Loss						
LOC105373311	X	108,975,739-108,978,187				AI, Loss						
LOC105373338	X	129,936,609-129,940,372				AI, Loss						
LOC105373378	X	152,617,486-152,625,567				AI, Loss						
LOC105373383	X	152,864,794-152,865,348				AI						
LOC286442	X	39,164,209-39,186,616				AI, Loss						Loss
LOC286467	X	130,836,677-130,964,671				AI, Loss						
LOC389906	X	3,735,575-3,761,935				AI, Loss						
LOC392452	X	45,590,576-45,591,246				AI, Loss						
LOC441493	X	47,693,820-47,696,192				AI, Loss						
LOC550643	X	56,755,717-56,844,004				Loss						
LOC642776	X	115,033,603-115,085,422				AI, Loss						
LOC643486	X	95,592,084-95,592,901				AI, Loss						
LOC729609	X	20,004,934-20,007,897				AI, Loss						
LONRF3	X	118,108,576-118,152,318				AI, Loss						
LPAR4	X	78,003,205-78,012,578				AI, Loss						
LRCH2	X	114,345,182-114,468,635				AI, Loss						
LUZP4	X	114,524,282-114,542,123				AI, Loss						
MAGEA1	X	152,481,521-152,486,116				AI, Loss						
MAGEA10	X	151,301,782-151,307,050				AI, Loss						

MAGEA10-MAGEA5	X	151,282,520-151,307,050				AI, Loss						
MAGEA12	X	151,899,292-151,903,184				AI, Loss						
MAGEA2	X	151,883,074-151,887,096				AI, Loss						
MAGEA2	X	151,918,386-151,922,408				AI, Loss						
MAGEA2B	X	151,883,074-151,887,096				AI, Loss						
MAGEA2B	X	151,918,386-151,922,408				AI, Loss						
MAGEA3	X	151,934,651-151,938,240				AI, Loss						
MAGEA4	X	151,080,995-151,093,642				Loss						
MAGEA4-AS1	X	151,072,902-151,079,555				Loss						
MAGEA5	X	151,282,520-151,286,411				AI, Loss						
MAGEA6	X	151,867,231-151,870,825				AI, Loss						
MAGEB16	X	35,816,458-35,821,852				AI, Loss						
MAGEB17	X	16,185,603-16,189,516				AI, Loss						
MAGED1	X	51,546,154-51,645,450				AI, Loss						
MAGED2	X	54,834,031-54,842,448				AI, Loss						
MAGED4	X	51,804,922-51,812,368				AI, Loss						
MAGED4	X	51,927,918-51,935,366				AI, Loss						
MAGED4B	X	51,804,922-51,812,368				AI, Loss						
MAGED4B	X	51,927,918-51,935,364				AI, Loss						
MAGEE1	X	75,648,045-75,651,746				AI, Loss						
MAGEE2	X	75,002,822-75,005,079				AI, Loss						
MAGEH1	X	55,478,521-55,480,001				Loss						
MAGT1	X	77,081,861-77,151,065				AI, Loss						
MAOA	X	43,514,154-43,606,071				AI, Loss						
MAOB	X	43,625,856-43,741,721				AI, Loss						
MAP2K4P1	X	72,744,110-72,782,921				AI, Loss						
MAP3K15	X	19,378,175-19,533,379				AI, Loss						
MAP7D2	X	20,024,830-20,135,114				AI, Loss						
MAP7D3	X	135,295,378-135,338,641				AI, Loss						
MBNL3	X	131,503,342-131,623,996				AI, Loss						
MBTPS2	X	21,857,655-21,903,541				AI, Loss						
MCTS1	X	119,737,743-119,755,016				AI, Loss						
MECP2	X	153,287,263-153,363,188				Loss						
MED12	X	70,338,405-70,362,304				Loss						
MED14	X	40,508,794-40,595,136				AI, Loss						
MED14OS	X	40,594,647-40,597,953				AI, Loss						
MID1	X	10,413,349-10,851,809				AI, Loss						
MID1IP1	X	38,660,684-38,665,783				AI, Loss						
MID1IP1-AS1	X	38,660,500-38,663,136				AI, Loss						
MID2	X	107,069,083-107,174,867				AI, Loss						
Mir_105	X	151,561,431-151,561,510				AI, Loss						
Mir_1302	X	12,251,432-12,251,484				AI, Loss						
Mir_340	X	97,607,750-97,607,847				AI, Loss						
Mir_361	X	85,158,640-85,158,712				AI, Loss						

Mir_500	X	49,771,257-49,771,345				AI, Loss						
Mir_544	X	15,470,854-15,470,959				AI, Loss						
Mir_548	X	94,318,142-94,318,224				AI, Loss						
Mir_584	X	11,615,063-11,615,191				AI, Loss						
Mir_633	X	109,657,139-109,657,216				AI, Loss						
Mir_652	X	10,939,960-10,940,057				AI, Loss						
mir-220	X	122,695,945-122,696,055				AI, Loss						
mir-223	X	65,235,302-65,240,264				Loss						
MIR105-1	X	151,560,690-151,560,771				AI, Loss						
MIR105-2	X	151,562,883-151,562,964				AI, Loss						
MIR106A	X	133,304,227-133,304,308				AI, Loss						
MIR1184-1	X	154,687,177-154,687,276				AI, Loss						
MIR1184-2	X	154,115,634-154,115,733				AI, Loss						
MIR1184-3	X	154,115,634-154,115,733				AI, Loss						
MIR1264	X	113,887,129-113,887,198				AI, Loss						
MIR1277	X	117,520,356-117,520,434				AI, Loss						
MIR1298	X	113,949,649-113,949,761				AI, Loss						
MIR1321	X	85,090,784-85,090,863				AI, Loss						
MIR188	X	49,768,108-49,768,194				AI, Loss						
MIR18B	X	133,304,070-133,304,141				AI, Loss						
MIR1911	X	113,997,743-113,997,823				AI, Loss						
MIR1912	X	113,886,018-113,886,098				AI, Loss						
MIR19B2	X	133,303,700-133,303,796				AI, Loss						
MIR20B	X	133,303,838-133,303,907				AI, Loss						
MIR221	X	45,605,584-45,605,694				AI, Loss						
MIR222	X	45,606,420-45,606,530				AI, Loss						
MIR223	X	65,238,711-65,238,821				Loss						
MIR23C	X	20,035,205-20,035,305				AI, Loss						
MIR3202-1	X	153,246,547-153,246,628				AI						
MIR3202-2	X	153,246,548-153,246,627				AI						
MIR325	X	76,225,828-76,225,926				AI, Loss						
MIR325HG	X	75,878,198-76,234,957				AI, Loss						
MIR361	X	85,158,640-85,158,712				AI, Loss						
MIR362	X	49,773,571-49,773,635				AI, Loss						
MIR363	X	133,303,407-133,303,482				AI, Loss						
MIR3672	X	120,504,825-120,504,907				AI, Loss						
MIR374A	X	73,507,120-73,507,192				AI, Loss						
MIR374B	X	73,438,381-73,438,453				AI, Loss						
MIR374C	X	73,438,383-73,438,453				AI, Loss						
MIR384	X	76,139,697-76,139,785				AI, Loss						
MIR3915	X	32,601,772-32,601,869				AI, Loss	AI					
MIR3937	X	39,520,469-39,520,575				AI, Loss						
MIR3978	X	109,325,345-109,325,446				AI, Loss						
MIR421	X	73,438,211-73,438,296				AI, Loss						

MIR424	X	133,680,643-133,680,741				AI, Loss						
MIR4328	X	78,156,690-78,156,746				AI, Loss						
MIR4329	X	112,023,945-112,024,016				AI, Loss						
MIR4454	X	109,113,629-109,113,681				AI, Loss						
MIR448	X	114,058,016-114,058,127				AI, Loss						
MIR450A1	X	133,674,370-133,674,461				AI, Loss						
MIR450A2	X	133,674,537-133,674,637				AI, Loss						
MIR450B	X	133,674,214-133,674,292				AI, Loss						
MIR4536-1	X	55,477,927-55,478,015				Loss						
MIR4536-2	X	55,477,927-55,478,015				Loss						
MIR4767	X	7,065,900-7,065,978				AI, Loss						
MIR4768	X	17,444,003-17,444,077				AI, Loss						
MIR4769	X	47,446,827-47,446,904				AI						
MIR4770	X	6,301,946-6,302,004				AI, Loss						
MIR500A	X	49,773,038-49,773,122				AI, Loss						
MIR500B	X	49,775,279-49,775,358				AI, Loss						
MIR501	X	49,774,329-49,774,413				AI, Loss						
MIR502	X	49,779,205-49,779,291				AI, Loss						
MIR503	X	133,680,357-133,680,428				AI, Loss						
MIR503HG	X	133,677,406-133,680,660				AI, Loss						
MIR532	X	49,767,753-49,767,844				AI, Loss						
MIR542	X	133,675,370-133,675,467				AI, Loss						
MIR545	X	73,506,938-73,507,044				AI, Loss						
MIR548AJ2	X	37,883,147-37,883,239				AI, Loss						
MIR548AM	X	16,645,134-16,645,208				AI, Loss						
MIR548AN	X	105,883,043-105,883,126				AI, Loss						
MIR548AX	X	11,336,733-11,336,806				AI, Loss						
MIR548F5	X	32,659,590-32,659,676				AI, Loss	Loss					
MIR548I4	X	83,480,759-83,480,836				AI, Loss						
MIR548M	X	94,318,139-94,318,225				AI, Loss						
MIR6086	X	13,608,410-13,608,465				AI, Loss						
MIR651	X	8,095,005-8,095,102				AI, Loss						
MIR652	X	109,298,556-109,298,654				AI, Loss						
MIR660	X	49,777,848-49,777,945				AI, Loss						
MIR664B	X	153,996,870-153,996,931				AI, Loss						
MIR676	X	69,242,706-69,242,773				AI, Loss						
MIR6858	X	153,678,667-153,678,734				AI						
MIR764	X	113,873,917-113,874,002				AI, Loss						
MIR766	X	118,780,700-118,780,811				AI, Loss						
MIR767	X	151,561,892-151,562,001				AI, Loss						
MIR8088	X	52,079,698-52,079,784				AI, Loss						
MIR92A2	X	133,303,567-133,303,642				AI, Loss						
MIR934	X	135,633,036-135,633,119				AI, Loss						
MMGT1	X	135,042,746-135,056,222				AI, Loss						

MORC4	X	106,057,100-106,243,474				AI, Loss						
MORF4L2	X	102,930,425-102,943,086				AI, Loss						
MORF4L2-AS1	X	102,942,211-102,947,484				AI, Loss						
MOSPD1	X	134,021,655-134,049,374				AI, Loss						
MOSPD2	X	14,891,526-14,940,288				AI, Loss						
MPC1L	X	40,482,817-40,483,391				AI, Loss						
MPP1	X	154,006,958-154,033,802				AI, Loss						
MSL3	X	11,776,277-11,793,872				AI, Loss						
MSN	X	64,887,510-64,961,793				Loss						
MST4	X	131,157,244-131,209,971				AI, Loss						
MTCP1	X	154,292,308-154,299,547				AI, Loss						
MTMR8	X	63,444,071-63,615,333				Loss						
MUM1L1	X	105,412,297-105,452,949				AI, Loss						
MXRA5	X	3,226,605-3,264,684				Loss						
NAA10	X	153,195,279-153,200,607				AI						
NAP1L2	X	72,432,136-72,434,710				AI, Loss						
NAP1L3	X	92,925,924-92,928,682				AI, Loss						
NAP1L6	X	72,345,875-72,347,919				AI, Loss						
NBDY	X	56,755,651-56,845,611				Loss						
NCBP2L	X	107,020,962-107,039,007				AI, Loss						
NDP	X	43,808,023-43,832,921				AI, Loss						
NDP-AS1	X	43,808,977-43,830,798				AI, Loss						
NDUFA1	X	119,005,733-119,010,629				AI, Loss						
NDUFB11	X	47,001,614-47,004,609				AI, Loss						
NEXMIF	X	73,952,690-74,145,287				AI, Loss						
NGFRAP1	X	102,631,267-102,633,001				AI, Loss						
NHS	X	17,393,542-17,754,113				AI, Loss						Loss
NHS-AS1	X	17,570,469-17,577,248				AI, Loss						
NHSL2	X	71,130,937-71,363,424				AI, Loss						
NKAP	X	119,059,012-119,077,735				AI, Loss						
NKAPP1	X	119,370,308-119,379,122				AI, Loss						
NKRF	X	118,722,299-118,739,846				AI, Loss						
NLGN3	X	70,364,680-70,391,051				Loss						
NLGN4X	X	5,808,066-6,146,923				AI, Loss						
NLRP2B	X	57,703,499-57,706,693				Loss						
NONO	X	70,503,041-70,521,018				Loss						
NRK	X	105,066,535-105,202,602				AI, Loss						
NSDHL	X	151,999,510-152,037,907				AI, Loss						
NUDT10	X	51,075,082-51,080,377				AI, Loss						
NUDT11	X	51,232,862-51,239,459				AI, Loss						
NUP62CL	X	106,366,656-106,449,670				AI, Loss						
NXF3	X	102,330,749-102,348,022				AI, Loss						
NXT2	X	108,779,009-108,787,927				AI, Loss						
NYX	X	41,306,712-41,334,905				AI, Loss						

OCRL	X	128,674,231-128,726,537				AI, Loss						
OFD1	X	13,752,831-13,787,480				AI, Loss						
OGT	X	70,752,911-70,795,747				Loss						
OPHN1	X	67,262,185-67,653,299				Loss						
OPN1LW	X	153,409,697-153,424,507				AI, Loss						
OPN1MW	X	153,448,084-153,462,352				AI						
OPN1MW2	X	153,448,084-153,461,637				AI						
OPN1MW3	X	153,448,106-153,461,637				AI						
OR13H1	X	130,678,047-130,678,974				AI, Loss						
OTC	X	38,211,735-38,280,703				AI, Loss						
OTUD6A	X	69,282,340-69,284,029				AI, Loss						
P2RY10	X	78,200,828-78,219,224				AI, Loss						
P2RY4	X	69,478,015-69,479,654				AI, Loss						
P2RY8	X	1,581,465-1,656,037				AI, Loss						
PABPC1L2A	X	72,297,176-72,299,351				AI, Loss						
PABPC1L2B	X	72,223,351-72,225,551				AI, Loss						
PABPC1L2B-AS1	X	72,218,226-72,222,695				AI, Loss						
PABPC5	X	90,689,596-90,693,583				AI, Loss						
PABPC5-AS1	X	90,669,901-90,689,998				AI, Loss						
PAGE1	X	49,452,053-49,460,596				AI, Loss						
PAGE2	X	55,115,484-55,119,269				Loss						
PAGE2B	X	55,101,488-55,119,260				Loss						
PAGE3	X	55,284,848-55,291,349				Loss						
PAGE4	X	49,593,862-49,598,867				AI, Loss						
PAGE5	X	55,246,778-55,250,541				Loss						
PAK3	X	110,187,468-110,470,589				Loss						
PASD1	X	150,732,006-150,845,211				AI, Loss					Loss	
PBDC1	X	75,392,763-75,398,145				AI, Loss						
PCDH11X	X	91,034,259-91,878,228				AI, Loss						
PCDH19	X	99,546,641-99,665,271				AI, Loss						
PCDHX	X	91,064,464-91,064,633				AI, Loss						
PDHA1	X	19,362,010-19,379,826				AI, Loss						
PDZD11	X	69,506,210-69,509,798				AI, Loss						
PDZD4	X	153,067,620-153,096,022				AI						
PGAM4	X	77,223,457-77,225,135				AI, Loss						
PGK1	X	77,359,665-77,382,324				AI, Loss						
PGRMC1	X	118,370,207-118,378,429				AI, Loss						
PHEX	X	22,050,561-22,269,427				AI, Loss						
PHEX-AS1	X	22,180,848-22,191,100				AI, Loss						
PHF16	X	46,771,735-46,920,641				AI, Loss						
PHF6	X	133,507,341-133,562,822				AI, Loss						
PHF8	X	53,963,112-54,071,569				AI, Loss						
PHKA1	X	71,798,663-71,934,029				AI, Loss						
PHKA1-AS1	X	71,908,798-71,932,190				AI, Loss						

PHKA2	X	18,910,415-19,002,480				AI, Loss						
PHKA2-AS1	X	18,908,413-18,913,093				AI, Loss						
PIGA	X	15,337,572-15,353,676				AI, Loss						
PIH1D3	X	106,449,861-106,487,473				AI, Loss						
PIN4	X	71,401,525-71,483,814				Loss						
PINCR	X	43,036,242-43,085,847				AI, Loss						
PIR	X	15,402,923-15,511,711				AI, Loss						
PIR-FIGF	X	15,363,712-15,509,432				AI, Loss						
PJA1	X	68,380,580-68,385,365				AI, Loss						
PLAC1	X	133,699,872-133,898,352				AI, Loss						
PLP1	X	103,031,433-103,047,547				Loss						
PLS3	X	114,795,176-114,885,179				AI, Loss						
PLS3-AS1	X	114,752,496-114,797,058				AI, Loss						
PLXNA3	X	153,686,620-153,701,989				AI, Loss						
PLXNB3	X	153,029,650-153,045,940				AI						
PNCK	X	152,935,187-152,953,226				AI						
PNMA3	X	152,224,765-152,228,827				Loss						
PNMA5	X	152,157,367-152,162,671				Loss						
PNMA6E	X	152,661,096-152,666,878				AI, Loss						
PNMA6F	X	152,583,146-152,587,238				AI, Loss						
PNPLA4	X	7,866,803-7,895,780				AI, Loss						
POF1B	X	84,532,394-84,634,748				AI, Loss						
PORCN	X	48,367,346-48,379,202				Loss						
POU3F4	X	82,763,268-82,764,775				AI, Loss						
PPEF1	X	18,709,044-18,846,034				AI, Loss						
PPEF1-AS1	X	18,706,762-18,710,806				AI, Loss						
PPP1R2C	X	42,636,616-42,637,486				AI, Loss						
PPP1R2P9	X	42,636,616-42,637,486				AI, Loss						
PPP1R3F	X	49,126,305-49,144,555				AI, Loss						
PPP2R3B	X	294,667-347,690				AI, Loss						
PRAF2	X	48,928,812-48,937,564				Loss						
PRDX4	X	23,685,644-23,704,514				AI, Loss						
PRICKLE3	X	49,031,150-49,042,853				Loss						
PRKX	X	3,522,383-3,631,675				AI, Loss						
PRKX-AS1	X	3,577,527-3,586,231				AI, Loss						
PRPS1	X	106,871,653-106,894,256				AI, Loss						
PRPS2	X	12,809,473-12,842,346				AI, Loss						
PRR32	X	125,953,746-125,955,768				AI, Loss						
PRRG1	X	37,208,527-37,316,548				AI, Loss						
PSMD10	X	107,327,434-107,334,874				AI, Loss						
PTCHD1	X	23,352,984-23,414,918				AI, Loss						
PTCHD1-AS	X	22,211,121-23,311,263				AI, Loss						
PUDP	X	6,966,960-7,066,231				AI, Loss						
PWWP3B	X	105,412,297-105,452,949				AI, Loss						

RAB33A	X	129,305,772-129,318,844				AI, Loss						
RAB39B	X	154,487,518-154,493,874				AI, Loss						
RAB40A	X	102,754,680-102,774,417				AI, Loss						
RAB41	X	69,502,021-69,504,852				AI, Loss						
RAB9A	X	13,707,239-13,727,944				AI, Loss						
RAB9B	X	103,031,251-103,087,212				Loss						
RADX	X	105,855,159-105,922,673				AI, Loss						
RAI2	X	17,818,168-17,879,457				AI, Loss						
RAP2C	X	131,337,051-131,353,508				AI, Loss						
RAP2C-AS1	X	131,352,534-131,566,839				AI, Loss						
RBBP7	X	16,862,774-16,888,534				AI, Loss						
RBM10	X	47,004,616-47,046,214				AI, Loss						
RBM41	X	106,305,117-106,362,057				AI, Loss						
RBMX	X	135,951,352-135,962,939				AI, Loss						
RBMX2	X	129,535,922-129,547,630				AI, Loss						
RBSG1	X	96,692,825-96,819,534				AI, Loss						
RENBP	X	153,200,721-153,210,232				AI						
REPS2	X	16,964,813-17,171,403				AI, Loss						
RGAG1	X	109,602,043-109,699,562				AI, Loss						
RGAG4	X	71,346,960-71,351,751				AI						
RGN	X	46,937,753-46,952,713				AI, Loss						
RHOXF1	X	119,243,010-119,249,847				AI, Loss						
RHOXF1-AS1	X	119,170,200-119,280,761				AI, Loss						
RHOXF1P1	X	119,144,673-119,149,508				AI, Loss						
RHOXF2	X	119,206,228-119,211,707				AI, Loss						
RHOXF2B	X	119,206,228-119,211,707				AI, Loss						
RHOXF2B	X	119,292,515-119,297,945				AI, Loss						
RIPPLY1	X	106,143,292-106,146,561				AI, Loss						
RLIM	X	73,802,810-73,834,461				AI, Loss						
RNF113A	X	119,004,494-119,005,791				AI						
RNF128	X	105,937,067-106,040,246				AI, Loss						
RNU6-28P	X	110,664,770-110,664,835				Loss						
RP2	X	46,696,346-46,741,791				AI, Loss						
RPA4	X	96,138,906-96,140,450				AI, Loss						
RPGR	X	38,128,415-38,186,788				AI, Loss						
RPL10	X	153,626,405-153,632,038				AI						
RPL39	X	118,920,466-118,925,622				AI						
RPL9	X	23,854,760-23,855,459				AI, Loss					Gain	Gain
RPS26P11	X	71,264,258-71,264,811				Loss						
RPS6KA3	X	20,168,028-20,285,523				AI, Loss						
RPS6KA6	X	83,313,353-83,442,943				AI, Loss						
RRAGB	X	55,744,109-55,785,207				Loss						
RS1	X	18,657,807-18,690,223				AI, Loss						
RTL3	X	77,911,565-77,914,825				AI, Loss						

RTL4	X	111,326,252-111,700,473				AI, Loss						
RTL5	X	71,346,960-71,351,751				AI						
RTL8A	X	134,184,962-134,186,221				AI, Loss						
RTL8B	X	134,154,533-134,156,566				AI, Loss						
RTL8C	X	134,166,332-134,167,575				AI, Loss						
RTL9	X	109,662,284-109,699,562				AI, Loss						
S100G	X	16,668,280-16,672,791				AI, Loss						
SAGE1	X	134,975,784-134,995,221				AI, Loss						
SAGE2P	X	134,812,380-134,819,809				AI, Loss						
SASH3	X	128,913,891-128,929,176				Loss						
SAT1	X	23,801,274-23,804,340				AI, Loss						
SATL1	X	84,347,291-84,363,974				AI, Loss						
SCARNA9L	X	20,154,183-20,154,531				AI, Loss						
SCML1	X	17,755,568-17,773,108				AI, Loss						Loss
SCML2	X	18,257,432-18,372,844				AI, Loss						
SERPINA7	X	105,277,189-105,282,718				AI, Loss						
SERTM2	X	110,754,889-110,765,627				Loss						
SH2D1A	X	123,480,131-123,507,010				AI, Loss						
SH3BGRL	X	80,457,302-80,554,046				AI, Loss						
SH3KBP1	X	19,552,082-19,905,744				AI, Loss						
SHOX	X	585,078-620,146				AI, Loss						
SHROOM2	X	9,754,495-9,917,483				AI, Loss						
SHROOM4	X	50,334,642-50,557,044				AI, Loss						
SLC16A2	X	73,641,327-73,753,764				AI, Loss						
SLC25A14	X	129,473,861-129,507,335				AI, Loss						
SLC25A43	X	118,533,257-118,588,437				AI, Loss						
SLC25A5	X	118,602,362-118,605,359				AI, Loss						
SLC25A5-AS1	X	118,599,995-118,603,083				AI, Loss						
SLC25A6	X	1,505,044-1,511,039				AI, Loss						
SLC6A14	X	115,567,746-115,592,625				AI, Loss						
SLC6A8	X	152,953,751-152,962,048				AI						
SLC7A3	X	70,145,429-70,150,975				AI, Loss						
SLC9A6	X	135,067,582-135,129,428				AI, Loss						
SLC9A7	X	46,458,685-46,618,607				AI, Loss						
SMARCA1	X	128,580,477-128,657,482				AI, Loss						
SMIM10	X	134,124,967-134,126,503				AI, Loss						
SMIM10L2A	X	134,555,857-134,561,999				AI, Loss						
SMIM10L2B	X	134,229,014-134,232,664				AI, Loss						
SMIM10L2B-AS1	X	134,254,548-134,257,529				AI, Loss						
SMIM9	X	154,051,622-154,062,937				AI, Loss						
SMPX	X	21,724,089-21,776,278				AI, Loss						
SMS	X	21,958,690-22,012,955				AI, Loss						
SNORA11	X	54,840,802-54,840,933				AI, Loss						
SNORA11	X	54,953,737-54,953,866				AI, Loss						

SNORA11C	X	47,248,048-47,248,175				AI, Loss						
SNORA11D	X	51,806,442-51,806,569				AI, Loss						
SNORA11E	X	51,806,442-51,806,569				AI, Loss						
SNORA11G	X	54,953,737-54,953,868				AI, Loss						
SNORA35	X	113,865,258-113,865,386				AI, Loss						
SNORA35	X	114,360,878-114,361,007				AI, Loss						
SNORA35	X	117,872,017-117,872,145				AI, Loss						
SNORA35B	X	114,360,878-114,361,005				AI, Loss						
SNORA36A	X	153,996,802-153,996,932				AI, Loss						
SNORA48B	X	3,450,157-3,450,292				AI						
SNORA56	X	154,003,272-154,003,401				AI, Loss						
SNORA69	X	118,921,315-118,921,447				AI						
SNORA70	X	153,628,621-153,628,756				AI						
SNORD112	X	128,652,552-128,652,624				AI, Loss						
SNORD3E	X	70,065,930-70,066,145				AI, Loss						
SNORD61	X	135,961,357-135,961,430				AI, Loss						
SNORD96B	X	109,468,216-109,468,288				AI, Loss						
SNX12	X	70,256,097-70,293,276				AI, Loss						
SOWAHD	X	118,892,575-118,894,165				AI						
SPACA5	X	47,863,733-47,869,126				AI, Loss						
SPACA5B	X	47,867,138-47,869,133				AI, Loss						
SPANXN5	X	52,825,185-52,826,388				AI, Loss						
SPIN2A	X	57,162,082-57,164,058				Loss						
SPIN2B	X	57,146,114-57,147,989				Loss						
SPIN3	X	57,002,802-57,021,988				Loss						
SPRY3	X	154,842,225-155,012,119				AI, Loss						
SRPK3	X	153,041,810-153,051,187				AI						
SRPX	X	38,008,587-38,080,177				AI, Loss						
SSR4	X	153,059,629-153,063,967				AI						
SSX1	X	48,114,751-48,126,879				AI, Loss						
SSX2	X	52,725,945-52,736,277				AI, Loss						
SSX2B	X	52,725,945-52,736,276				AI, Loss						
SSX3	X	48,205,862-48,216,188				AI, Loss						
SSX4	X	48,242,967-48,252,785				AI, Loss						
SSX4B	X	48,242,956-48,252,785				AI, Loss						
SSX5	X	48,045,655-48,056,199				AI, Loss						
SSX6	X	47,967,366-47,980,068				AI, Loss						
SSX6P	X	47,967,366-47,980,068				AI, Loss						
SSX7	X	52,673,110-52,683,950				AI, Loss						
SSX8	X	52,651,984-52,662,998				AI, Loss						
SSX8P	X	52,651,984-52,662,998				AI, Loss						
SSX9	X	48,160,984-48,165,614				AI, Loss						
SSX9P	X	48,160,984-48,165,614				AI, Loss						
STAG2	X	123,094,409-123,236,505				AI, Loss						Gain

STARD8	X	67,867,510-67,945,684				Loss						
STK26	X	131,157,244-131,209,971				AI, Loss						
STS	X	7,065,292-7,272,684				AI, Loss						
SUPT20HL2	X	24,326,331-24,331,510				AI, Loss						
SYAP1	X	16,737,706-16,780,807				AI, Loss						
SYN1	X	47,431,299-47,479,256				AI						
SYP	X	49,044,264-49,056,661				Loss						
SYP-AS1	X	49,055,297-49,058,913				Loss						
SYTL5	X	37,865,834-37,988,073				AI, Loss						
TAF1	X	70,586,088-70,752,224				Loss						
TAF9B	X	77,385,244-77,395,179				AI, Loss						
TAZ	X	153,639,853-153,650,063				AI						
TBC1D8B	X	106,045,918-106,119,377				AI, Loss						
TBL1X	X	9,431,334-9,687,780				AI, Loss						
TBX22	X	79,270,254-79,287,268				AI, Loss						
TCEAL1	X	102,883,647-102,885,876				AI, Loss						
TCEAL3	X	102,862,833-102,864,855				AI, Loss						
TCEAL4	X	102,831,158-102,842,664				AI, Loss						
TCEAL5	X	102,528,617-102,531,797				AI, Loss						
TCEAL7	X	102,585,113-102,587,254				AI, Loss						
TCEAL8	X	102,507,922-102,510,121				AI, Loss						
TCEAL9	X	102,611,379-102,613,397				AI, Loss						
TCEANC	X	13,671,224-13,700,083				AI, Loss						
TDGF1P3	X	109,763,539-109,766,249				Loss						
TENM1	X	123,509,755-124,097,666				AI, Loss						
TENT5D	X	79,591,002-79,700,810				AI, Loss						
TEX11	X	69,748,789-70,128,567				AI, Loss						
TEX13A	X	104,463,610-104,465,377				AI, Loss						
TEX13B	X	107,224,093-107,225,600				AI, Loss						
TEX13C	X	124,453,719-124,459,063				AI, Loss						
TEX13D	X	123,466,509-123,470,712				AI, Loss						
TEX28	X	153,424,832-153,441,837				AI						
TEX28	X	153,461,962-153,482,668				AI						
TEX28	X	153,498,929-153,523,796				AI						
TFDP3	X	132,350,696-132,352,376				AI, Loss						
TFE3	X	48,886,237-48,901,043				Loss						
TGIF2LX	X	89,176,939-89,177,882				AI, Loss						
THOC2	X	122,745,290-122,757,146				AI, Loss						
THOC2	X	122,734,411-122,866,904				AI, Loss						
TIMP1	X	47,441,689-47,446,190				AI						
TKTL1	X	153,524,026-153,558,713				AI						
TLR7	X	12,885,201-12,908,480				AI, Loss						
TLR8	X	12,924,738-12,941,288				AI, Loss						
TLR8-AS1	X	12,920,935-12,961,419				AI, Loss						

TMEM164	X	109,245,862-109,427,460				AI, Loss						
TMEM187	X	153,237,990-153,248,646				AI, Loss						
TMEM255A	X	119,392,504-119,445,391				AI, Loss						
TMEM27	X	15,645,440-15,683,154				AI, Loss						
TMEM31	X	102,965,836-102,968,960				AI, Loss						
TMLHE	X	154,718,672-154,842,622				AI, Loss						
TMLHE-AS1	X	154,576,449-154,603,823				AI, Loss						
TMSB4X	X	12,993,225-12,995,346				AI, Loss						
TRAPPC2	X	13,730,360-13,752,754				AI, Loss						
TREX2	X	152,710,177-152,713,367				AI, Loss						
TRNA_Ile	X	3,756,417-3,756,491				AI, Loss						
TRNA_Val	X	18,693,028-18,693,101				AI, Loss						
TRO	X	54,946,995-54,957,866				AI, Loss						
TRPC5	X	111,017,541-111,326,004				Loss						
TRPC5OS	X	111,119,278-111,147,213				Loss						
TSC22D3	X	106,956,451-107,019,218				AI, Loss						
TSIX	X	73,012,039-73,049,066				AI, Loss						
TSPAN7	X	38,420,730-38,548,172				AI, Loss						
TSR2	X	54,466,807-54,474,460				AI, Loss						
TTC3P1	X	74,960,372-74,962,914				AI, Loss						
TXLNG	X	16,804,554-16,862,642				AI, Loss						
U00684	X	154,697,946-154,716,707				AI, Loss						
U3	X	121,974,278-121,974,498				AI, Loss						
U4	X	16,893,268-16,893,390				AI, Loss						
U4	X	69,670,728-69,670,872				AI, Loss						
U6	X	10,550,856-10,550,959				AI, Loss						
U6	X	41,778,506-41,778,602				AI, Loss						
U7	X	139,533,312-139,533,373				AI, Loss						
UBA1	X	47,050,198-47,074,527				AI, Loss						
UBE2A	X	118,708,429-118,718,392				AI, Loss						
UBE2DNL	X	84,189,156-84,189,896				AI, Loss						
UBE2E4P	X	14,262,386-14,263,545				AI, Loss						
UBQLN2	X	56,590,025-56,593,443				Loss						
UPF3B	X	118,967,988-118,986,991				AI						
UPRT	X	74,376,203-74,524,732				AI, Loss						
UQCRBP1	X	56,763,220-56,764,017				Loss						
USP11	X	47,092,313-47,107,727				AI, Loss						
USP26	X	132,159,053-132,231,135				AI, Loss						
USP27X	X	49,644,469-49,647,168				AI, Loss						
USP27X-AS1	X	49,641,326-49,643,959				AI, Loss						
USP51	X	55,511,048-55,515,631				Loss						
USP9X	X	40,944,887-41,095,832				AI, Loss						Gain
UTP14A	X	129,040,096-129,063,738				Loss						
UXT	X	47,511,190-47,518,579				AI, Loss						

UXT-AS1	X	47,518,231-47,519,510				AI, Loss						
VAMP7	X	155,110,942-155,173,433				AI, Loss						
VBP1	X	154,425,283-154,468,122				AI, Loss						
VCX	X	7,810,302-7,812,184				AI, Loss						
VCX-8r	X	8,434,331-8,434,546				AI, Loss						
VCX2	X	8,137,984-8,139,308				AI, Loss						
VCX3A	X	6,451,658-6,453,159				AI, Loss						
VCX3B	X	8,432,870-8,434,551				AI, Loss						
VEGFD	X	15,363,712-15,402,535				AI, Loss						
VGLL1	X	135,614,310-135,638,966				AI, Loss						
VSIG1	X	107,288,199-107,322,414				AI, Loss						
VSIG4	X	65,241,579-65,259,967				Loss						
WASH1	X	155,249,966-155,253,125				AI, Loss						
WASH6P	X	155,251,978-155,255,330				AI, Loss						
WASIR1	X	155,244,228-155,246,495				AI, Loss						
WBP5	X	102,611,379-102,613,397				AI, Loss						
WDR44	X	117,480,035-117,583,923				AI, Loss						
WDR45	X	48,932,091-48,958,059				Loss						
WNK3	X	54,219,255-54,384,438				AI, Loss						
XACT	X	112,859,586-113,181,506				AI, Loss						
XAGE1A	X	52,238,962-52,243,953				AI, Loss						
XAGE1A	X	52,255,219-52,260,154				AI, Loss						
XAGE1A	X	52,528,159-52,533,094				AI, Loss						
XAGE1A	X	52,541,053-52,546,197				AI, Loss						
XAGE1B	X	52,238,809-52,243,953				AI, Loss						
XAGE1B	X	52,255,219-52,260,363				AI, Loss						
XAGE1C	X	52,239,018-52,243,953				AI, Loss						
XAGE1C	X	52,541,053-52,546,197				AI, Loss						
XAGE1D	X	52,238,809-52,243,953				AI, Loss						
XAGE2	X	52,112,152-52,118,826				AI, Loss						
XAGE3	X	52,891,557-52,897,119				AI, Loss						
XAGE5	X	52,841,227-52,847,322				AI, Loss						
XG	X	2,670,092-2,734,541				Loss						
XGY2	X	2,670,336-2,693,037				Loss						
XIAP	X	122,993,661-123,047,829				AI, Loss						Gain
XIST	X	73,040,485-73,072,588				AI, Loss						
XK	X	37,545,043-37,591,383				AI, Loss						
XPNPEP2	X	128,872,945-128,903,525				AI, Loss						
XRCC6P5	X	98,716,599-99,194,841				AI, Loss						
Y_RNA	X	12,072,942-12,073,045				AI, Loss						
Y_RNA	X	23,800,326-23,800,427				AI, Loss						
YIPF6	X	67,718,623-67,757,127				Loss						
YY2	X	21,874,104-21,876,845				AI, Loss						
ZBED1	X	2,404,454-2,419,008				AI, Loss						

ZBTB33	X	119,384,606-119,392,251				AI, Loss						
ZC3H12B	X	64,708,614-64,727,767				Loss						
ZC4H2	X	64,135,681-64,254,624				Loss						
ZCCHC12	X	117,957,705-117,960,936				AI, Loss						
ZCCHC13	X	73,524,024-73,524,869				AI, Loss						
ZCCHC16	X	111,326,252-111,700,473				AI, Loss						
ZCCHC5	X	77,911,565-77,914,825				AI, Loss						
ZDHHC15	X	74,588,261-74,743,337				AI, Loss						
ZDHHC9	X	128,937,263-128,978,124				Loss						
ZFP92	X	152,683,780-152,687,086				AI, Loss						
ZFX	X	24,167,105-24,234,372				AI, Loss					Gain	Gain
ZFX-AS1	X	24,164,341-24,167,771				AI, Loss					Gain	Gain
ZMYM3	X	70,459,473-70,475,047				Loss						
ZNF157	X	47,229,998-47,273,098				AI, Loss						
ZNF182	X	47,834,249-47,863,394				AI, Loss						
ZNF185	X	152,082,985-152,142,025				AI, Loss						
ZNF275	X	152,599,612-152,618,384				AI, Loss						
ZNF280C	X	129,336,672-129,402,922				AI, Loss						
ZNF41	X	47,304,576-47,342,668				AI, Loss						
ZNF449	X	134,477,627-134,497,338				AI, Loss						
ZNF630	X	47,842,684-47,931,025				AI, Loss						
ZNF630-AS1	X	47,915,698-47,926,696				AI, Loss						
ZNF645	X	22,291,029-22,292,576				AI, Loss						
ZNF674	X	46,357,159-46,404,892				AI, Loss						
ZNF674-AS1	X	46,404,924-46,407,910				AI, Loss						
ZNF711	X	84,498,996-84,528,368				AI, Loss						
ZNF75D	X	134,382,535-134,478,012				AI, Loss						
ZNF81	X	47,696,300-47,781,655				AI, Loss						
ZRSR2	X	15,808,573-15,841,382				AI, Loss						
ZXDA	X	57,931,863-57,937,067				Loss						
ZXDB	X	57,618,268-57,623,910				Loss						

Supplementary Table S3D. Known cancer genes affected by CNVs in 10 cranial meningiomas. Gene ID; gene name; chromosome number; gene genomic coordinates (hg19); role in cancer (fusion, oncogene or tumor suppressor gene (TSG) and type of CNV (gain, loss, loss of heterozygosity (LOH) or allelic imbalance (AI)) in each individual tumor are shown from left to right.

Gene symbol	Gene name	Chr	Position	Role in cancer	P1_C1	P1_C2	P1_C3	P1_C4	P1_C5	P3_C1	P3_C2	P3_C3	P6_C1	P7_C1
MDS2	myelodysplastic syndrome 2	1	23,907,984-23,967,058	fusion			AI							
PAX7	paired box gene 7	1	18,957,499-19,075,360	fusion			Loss							
THRAP3	thyroid hormone receptor associated protein 3 (	1	36,690,012-36,770,957	fusion			AI						AI	
GOPC	golgi associated PDZ and coiled-coil motif cont	6	117,639,350-117,923,705	fusion			Loss							
AKAP9	A kinase (PRKA) anchor protein (yotiao) 9	7	91,570,188-91,739,987	fusion			Gain							
ZCCHC8	zinc finger, CCHC domain containing 8	12	122,956,145-122,985,620	fusion										Gain
TRA	T cell receptor alpha locus	14	22,458,652-23,016,719	fusion	Gain	Gain	Gain	Gain	Gain	Gain	Gain	Gain	Gain	Gain
TRA	T cell receptor alpha locus	14	22,192,007-22,447,356	fusion	Gain	Gain	Gain	Gain	Gain	Gain	Gain	Gain		
TRD	T cell receptor delta locus	14	22,465,824-23,016,719	fusion	Gain	Gain	Gain	Gain	Gain	Gain	Gain	Gain	Gain	Gain
LASP1	LIM and SH3 protein 1	17	37,026,111-37,078,023	fusion									AI, Gain	
MLLT6	myeloid/lymphoid or mixed-lineage leukemia (t	17	36,861,872-36,886,056	fusion									AI	
BCR	breakpoint cluster region	22	23,522,551-23,660,224	fusion	AI, Loss	AI, Loss	AI, Loss	AI, Loss	AI, Loss	AI, Loss	AI	AI		
BCR	breakpoint cluster region	22	22,979,652-22,981,152	fusion	AI, Loss	AI, Loss	AI, Loss	AI, Loss	AI, Loss		AI			
BCR	breakpoint cluster region	22	21,642,517-21,644,017	fusion	Loss	Loss		AI, Loss		AI, Loss	AI	AI, Loss		
MSN	moesin	X	64,887,510-64,961,793	fusion				Loss						
NONO	non-POU domain containing, octamer-binding	X	70,503,041-70,521,018	fusion				Loss						
CSF3R	colony stimulating factor 3 receptor (granulocyt	1	36,931,643-36,948,915	oncogene			AI						AI	
FUBP1	far upstream element (FUSE) binding protein 1	1	78,409,735-78,444,889	oncogene			AI, Loss							
JUN	jun oncogene	1	59,246,462-59,249,785	oncogene			AI							
MPL	myeloproliferative leukaemia virus oncogene, t	1	43,803,474-43,820,135	oncogene			AI, Loss						AI	
MTOR	mechanistic target of rapamycin	1	11,166,587-11,322,608	oncogene			AI						Loss	
MYCL	v-myc avian myelocytomatosis viral oncogene	1	40,361,095-40,367,687	oncogene			AI						AI	
SKI	SKI proto-oncogene	1	2,160,133-2,241,652	oncogene			AI						AI, Loss	
LCK	lymphocyte-specific protein tyrosine kinase	1	32,716,839-32,751,768	oncogene, fusion			AI						AI, Loss	
PRDM16	PR domain containing 16	1	2,985,741-3,355,185	oncogene, fusion			AI						AI, Loss	Loss
STIL	SCL/TAL1 interrupting locus	1	47,694,867-47,779,819	oncogene, fusion			AI, Loss							
TAL1	T-cell acute lymphocytic leukemia 1 (SCL)	1	47,681,961-47,698,007	oncogene, fusion			AI							
ROS1	v-ros UR2 sarcoma virus oncogene homolog 1 (	6	117,609,529-117,747,018	oncogene, fusion			Loss							
CCR7	C-C motif chemokine receptor 7	17	38,710,021-38,721,736	oncogene									Loss	
STAT3	signal transducer and activator of transcription	17	40,465,342-40,540,513	oncogene									AI	
ERBB2	v-erb-b2 erythroblastic leukemia viral oncogene	17	37,844,336-37,884,915	oncogene, fusion									AI	
ETV4	ets variant gene 4 (E1A enhancer binding protei	17	41,605,210-41,623,800	oncogene, fusion									AI	
RARA	retinoic acid receptor, alpha	17	38,465,422-38,566,941	oncogene, fusion									Gain, Loss	
TAF15	TAF15 RNA polymerase II, TATA box binding	17	34,136,458-34,174,246	oncogene, fusion									AI	
DGCR8	DGCR8, microprocessor complex subunit	22	20,067,754-20,099,400	oncogene	Loss			AI, Loss	Gain	AI		AI, Loss		
MAPK1	mitogen-activated protein kinase 1	22	22,113,946-22,221,970	oncogene	AI, Loss	AI, Loss	AI, Loss	AI, Loss	AI, Loss	AI, Loss	Loss	AI, Loss		
EWSR1	Ewing sarcoma breakpoint region 1 (EWS)	22	29,663,997-29,696,515	oncogene, fusion	AI, Loss	AI, Loss	AI, Loss	AI, Loss	AI, Loss	AI, Loss	Loss	AI, Loss	AI, Loss	
MN1	MN1 proto-oncogene, transcriptional regulator	22	28,144,264-28,197,486	oncogene, fusion	AI, Loss			AI, Loss	AI, Loss	AI, Loss	AI	AI, Loss	AI, Loss	
PDGFB	platelet-derived growth factor beta polypeptide	22	39,619,363-39,641,060	oncogene, fusion	AI, Loss	AI, Loss	AI	Loss	Loss	AI	AI	AI, Loss		
AR	Androgen Receptor	X	66,763,873-66,950,461	oncogene				Loss						
ARAF	A-Raf proto-oncogene, serine/threonine kinase	X	47,420,498-47,431,320	oncogene				AI, Loss						
WAS	Wiskott-Aldrich syndrome	X	48,542,185-48,549,817	oncogene				Loss						
CRLF2	cytokine receptor-like factor 2	X	1,314,886-1,656,037	oncogene, fusion				AI, Loss					Gain	Gain
MTCP1	mature T-cell proliferation 1	X	154,292,308-154,299,547	oncogene, fusion				AI, Loss						
P2RY8	purinergic receptor P2Y, G-protein coupled, 8	X	1,581,465-1,656,037	oncogene, fusion				AI, Loss						
SSX1	synovial sarcoma, X breakpoint 1	X	48,114,751-48,126,879	oncogene, fusion				AI, Loss						
SSX2	synovial sarcoma, X breakpoint 2	X	52,725,945-52,736,277	oncogene, fusion				AI, Loss						

SSX4	synovial sarcoma, X breakpoint 4	X	48,242,967-48,252,785	oncogene, fusion				AI, Loss						
TFE3	transcription factor binding to IGHM enhancer	X	48,886,237-48,901,043	oncogene, fusion				Loss						
JAK1	Janus kinase 1	1	65,298,905-65,533,429	oncogene, TSG			AI							
STAT5B	signal transducer and activator of transcription	17	40,351,194-40,428,424	oncogene, TSG, fusion									AI	
SUZ12	suppressor of zeste 12 homolog (Drosophila)	17	30,264,028-30,328,064	oncogene, TSG, fusion									AI, Gain	
APOBEC3B	apolipoprotein B mRNA editing enzyme catalyt	22	39,378,403-39,388,784	oncogene, TSG	Loss	Loss	Loss	Loss	AI, Loss	AI	AI		Loss	
MKL1	megakaryoblastic leukemia (translocation) 1	22	40,806,291-41,032,690	oncogene, TSG, fusion	AI, Loss	AI, Loss	AI, Loss	AI, Loss	AI, Loss	AI, Loss	AI, Loss	AI, Loss	Loss	
BTK	Bruton agammaglobulinemia tyrosine kinase	X	100,604,434-100,645,784	oncogene, TSG				AI, Loss						
GATA1	GATA binding protein 1 (globin transcription fa	X	48,644,981-48,652,717	oncogene, TSG				Loss						
GPC3	glypican 3	X	132,669,775-133,119,673	oncogene, TSG				AI, Loss						
IRS4	insulin receptor substrate 4	X	107,975,726-107,979,607	oncogene, TSG				AI, Loss						
KDM6A	lysine (K)-specific demethylase 6A, UTX	X	44,732,420-44,971,857	oncogene, TSG				AI, Loss						
ELF4	E74-like factor 4 (ets domain transcription facto	X	129,198,894-129,244,688	oncogene, TSG, fusion				AI, Loss						
ARHGEF10L	Rho guanine nucleotide exchange factor 10 like	1	17,846,826-18,024,370	TSG			AI							
ARHGEF10L	Rho guanine nucleotide exchange factor 10 like	1	17,914,910-17,966,476	TSG			AI							
CASP9	caspase 9	1	15,817,323-15,851,285	TSG									AI, Loss	
CDKN2C	cyclin-dependent kinase inhibitor 2C (p18, inhil	1	51,434,366-51,440,309	TSG			AI							
ID3	inhibitor of DNA binding 3, HLH protein	1	23,884,420-23,886,285	TSG			AI							
MUTYH	mutY homolog (E. coli)	1	45,794,913-45,806,142	TSG			AI							
PRDM2	PR/SET domain 2	1	14,026,734-14,151,574	TSG			AI						AI, Loss	
SDHB	succinate dehydrogenase complex, subunit B, ir	1	17,345,224-17,380,665	TSG			AI							
SPEN	spen family transcriptional repressor	1	16,174,358-16,266,950	TSG			AI						AI, Loss	
TNFRSF14	tumor necrosis factor receptor superfamily, mem	1	2,487,803-2,497,061	TSG			AI						AI, Loss	
ARID1A	AT rich interactive domain 1A (SWI-like)	1	27,022,521-27,108,601	TSG, fusion			AI						Loss	
CAMTA1	calmodulin binding transcription activator 1	1	6,845,383-7,829,766	TSG, fusion			AI						AI, Loss	
EPS15	epidermal growth factor receptor pathway subst	1	51,819,934-51,984,995	TSG, fusion			AI							
RPL22	ribosomal protein L22 (EAP)	1	6,245,079-6,259,679	TSG, fusion			AI						AI, Loss	
SFPQ	splicing factor proline/glutamine rich(polypyrin	1	35,641,978-35,658,746	TSG, fusion			AI						Gain	
ROBO2	roundabout guidance receptor 2	3	75,986,644-77,699,114	TSG			Loss							
FAT4	FAT atypical cadherin 4	4	126,237,566-126,414,087	TSG			Gain							
N4BP2	NEDD4 binding protein 2	4	40,058,523-40,159,872	TSG										Gain
RAD17	RAD17 checkpoint clamp loader component	5	68,665,123-68,710,630	TSG										Gain
SDHA	succinate dehydrogenase complex, subunit A, fl	5	218,337-257,197	TSG										Loss
MGMT	O-6-methylguanine-DNA methyltransferase	10	131,265,504-131,569,247	TSG										Loss
BAZ1A	bromodomain adjacent to zinc finger domain 1A	14	35,221,936-35,344,853	TSG									Gain	Gain
BRCA1	familial breast/ovarian cancer gene 1	17	41,196,311-41,322,420	TSG									AI, Gain	
CDK12	cyclin-dependent kinase 12	17	37,617,738-37,690,818	TSG									AI, Gain	
SMARCE1	SWI/SNF related, matrix associated, actin deper	17	38,783,975-38,804,103	TSG									Loss	
CHEK2	CHK2 checkpoint homolog (S. pombe)	22	29,083,730-29,137,822	TSG	AI, Loss	AI, Loss	AI, Loss	AI, Loss	AI, Loss	AI, Loss	AI, Loss	AI, Loss	AI, Loss	
LZTR1	leucine-zipper-like transcription regulator 1	22	21,333,750-21,353,326	TSG	Loss	Loss		AI, Loss		AI	AI			
NF2	neurofibromatosis type 2 gene	22	29,999,544-30,094,589	TSG	AI, Loss	AI, Loss	AI, Loss	AI, Loss	AI, Loss	AI, Loss	AI	AI, Loss	AI, Loss	
SMARCB1	SWI/SNF related, matrix associated, actin deper	22	24,129,117-24,176,705	TSG	Loss	Loss	AI, Loss	Loss	AI, Loss	AI, Loss	AI			
ZNRF3	zinc and ring finger 3	22	29,279,754-29,453,476	TSG	AI, Loss	AI, Loss	AI, Loss	AI, Loss	AI, Loss	AI, Loss	AI, Loss	AI, Loss	AI, Loss	
CLTCL1	clathrin, heavy polypeptide-like 1	22	19,166,986-19,279,239	TSG, fusion	AI, Loss	AI, Loss		AI, Loss	Gain	AI	AI	Loss		
EP300	300 kd E1A-Binding protein gene	22	41,488,613-41,576,081	TSG, fusion	AI, Loss	AI, Loss	AI, Loss	AI, Loss	AI, Loss	AI, Loss		AI, Loss		
MYH9	myosin, heavy polypeptide 9, non-muscle	22	36,677,322-36,784,112	TSG, fusion				AI, Loss	AI, Loss	AI, Loss	AI	AI, Loss	AI, Loss	
AMER1	APC membrane recruitment protein 1	X	63,404,996-63,425,588	TSG				Loss						
ATP2B3	ATPase, Ca++ transporting, plasma membrane	X	152,801,579-152,848,387	TSG				AI						
ATRX	alpha thalassemia/mental retardation syndrome	X	76,760,355-77,041,755	TSG				AI, Loss						
DDX3X	DEAD-box helicase 3, X-linked	X	41,192,560-41,223,725	TSG				AI, Loss						Gain
MED12	mediator complex subunit 12	X	70,338,405-70,362,304	TSG				Loss						

PHF6	PHD finger protein 6	X	133,507,341-133,562,822	TSG				AI, Loss						
RBM10	RNA binding motif protein 10	X	47,004,616-47,046,214	TSG				AI, Loss						
RPL10	ribosomal protein L10	X	153,626,405-153,632,038	TSG				AI						
STAG2	stromal antigen 2	X	123,094,409-123,236,505	TSG				AI, Loss						Gain
ZMYM3	zinc finger MYM-type containing 3	X	70,459,473-70,475,047	TSG				Loss						
ZRSR2	zinc finger (CCCH type), RNA-binding motif a	X	15,808,573-15,841,382	TSG				AI, Loss						
BCOR	BCL6 corepressor	X	39,910,498-40,036,582	TSG, fusion				AI, Loss						
ZNF429	zinc finger protein 429	19	21,688,353-21,739,070			Gain	Gain							
ISX	intestine specific homeobox	22	35,462,128-35,483,380		AI, Loss	AI, Loss	AI, Loss	AI, Loss	AI, Loss	AI, Loss	AI		AI, Loss	
DCAF12L2	DDB1 and CUL4 associated factor 12 like 2	X	125,297,481-125,300,080					AI, Loss						
EIF1AX	eukaryotic translation initiation factor 1A, X-lin	X	20,142,635-20,159,966					AI, Loss						
FAM47C	family with sequence similarity 47 member C	X	37,026,431-37,029,739					AI, Loss						
FLNA	filamin A	X	153,576,899-153,603,006					AI						

Supplementary Table S4. CNVs in P6_C1 genome. Chromosomal coordinates of the CNV (hg19), type of CNV (gain, loss, or allelic imbalance (AI)), the CNV length in bp, the chromosomal cytoband, percent of the region's overlap with a known CNV, number of probes in t CNV, micro RNA symbols, number of miRNA in the CNV, gene symbols, number of genes found in the CNV, known cancer genes present in the CNV, type of cancer gene (oncogene, tumor suppressor gene (TSG) and fusion), and whether the CNV is relevant for tumor progression (loss of TSG or gain of oncogene => Yes; otherwise => No; Allelic Imbalance => Unknown) are shown in the columns from left to right. CNVs comprising cancer genes are highlighted in red. *EZH1* containing region is highlighted in blue font. Although *EZH1* is not a cancer gene, it interacts with *SUZ12* in PRC2 complex. Cancer genes in chromosome 22 are not shown.

Chromosomal region	Type of CNV	Length (bp)	Cytoband	Percent of overlap with known CNV	Probes	miRNAs	Count of miRNAs	Gene symbols	Count of genes	Cancer gene	Type of cancer gene	Relevant?
chr1:0-909,867	Loss	909,868	p36.33	98.86	140	hsa-mir-130	2	*AK056486, AK310751, BC03625*	39			
chr1:979,185-1,093,517	Loss	114,333	p36.33	100.00	81		0	*AGRN, AK310350, BC033949, C*	7			
chr1:1,116,668-1,435,417	Loss	318,750	p36.33	100.00	334		0	*ACAP3, ANKRD65, ATAD3B, AT*	29			
chr1:1,535,600-8,049,903	Loss	6,514,304	p36.33 - p36.23	48.01	3026	hsa-mir-425	5	*ACOT7, ACTRT2, AJAP1, AK054*	99	*TNFRSF14, RPL22, CAMTA1*	TSG, TSG, TSG	Yes, Yes, Yes
chr1:8,279,708-8,614,184	Loss	334,477	p36.23	33.71	72		0	*BC113958, LOC102724552, RER*	4			
chr1:9,063,251-9,365,743	Loss	302,493	p36.23 - p36.22	52.22	200	hsa-mir-34a	1	*GPR157, H6PD, MIR34A, SLC2A*	7			
chr1:9,656,788-9,795,378	Loss	138,591	p36.22	9.82	74		0	*BC038541, C1orf200, CLSTN1, P*	5			
chr1:10,501,145-11,854,257	Loss	1,353,113	p36.22	38.15	631		0	*AGTRAP, AK125437, ANGPTL7,*	28	*MTOR*	Oncogene	No
chr1:11,865,993-12,202,875	Loss	336,883	p36.22	19.21	214		0	*CLCN6, KIAA2013, MFN2, MIIP,*	11			
chr1:12,253,008-12,606,559	Loss	353,552	p36.22	33.90	146		0	*SNORA59A, SNORA59B, TNFRS*	4			
chr1:12,670,812-13,907,019	Loss	1,236,208	p36.22 - p36.21	97.59	238		0	*AADACL3, AADACL4, C1orf158,*	35			
chr1:14,005,776-16,500,273	Loss	2,494,498	p36.21 - p36.13	17.19	1276		0	*AGMAT, AK055853, AK124197,*	33	*SPEN*	TSG	Yes
chr1:16,532,477-16,890,472	Loss	357,996	p36.13	42.05	106		0	*AL832937, ARHGEF19, AX74798*	14			
chr1:24,853,163-26,623,937	Loss	1,770,775	p36.11	17.64	695	hsa-mir-442	2	*AUNIP, AX747205, AX747207, B*	40			
chr1:26,650,741-26,691,446	Loss	40,706	p36.11	0.00	62		0	*AIM1L, ZNF683*	2			
chr1:26,921,861-27,178,392	Loss	256,532	p36.11	50.97	70		0	*ARID1A, BC016143, PIGV, ZDH*	4	*ARID1A*	TSG	Yes
chr1:27,215,989-27,410,945	Loss	194,957	p36.11	53.86	108		0	*BC016143, C1orf172, FAM46B,*	9			
chr1:27,724,551-27,875,310	Loss	150,760	p36.11	73.23	32		0	*AHDC1, WASF2*	2			
chr1:27,970,491-28,421,615	Loss	451,125	p36.11 - p35.3	4.21	146		0	*EYA3, FAM76A, IFI6, PPP1R8, R*	10			
chr1:29,260,519-29,444,133	Loss	183,615	p35.3	17.37	30		0	*EPB41*	1			
chr1:29,573,696-32,834,096	Loss	3,260,401	p35.3 - p35.1	27.89	1349	hsa-mir-425	3	*AX747565, BAI2, BC042538, BC*	49	*LCK*	Oncogene	No
chr1:33,229,096-35,361,311	Loss	2,132,216	p35.1 - p34.3	34.97	939	hsa-mir-360	2	*7SK, A3GALT2, ADC, AK2, AX74*	32			
chr1:35,479,290-35,902,952	Gain	423,663	p34.3	10.79	100		0	*AF119915, AL831889, BX537811*	8	*SFPQ*	TSG	No
chr1:36,055,940-36,172,748	Gain	116,809	p34.3	0.00	30		0	*PSMB2, TFAP2E*	2			
chr1:36,238,746-36,550,625	Gain	311,880	p34.3	0.00	52		0	*AGO1, AGO3, AGO4, AK025726,*	5			
chr1:36,553,483-36,625,781	Gain	72,299	p34.3	25.39	45		0	*ADPRHL2, COL8A2, MAP7D1, T*	5			
chr1:36,625,781-37,890,108	AI	1,264,328	p34.3	21.07	108	hsa-mir-425	1	*CSF3R, EVA1B, GRIK3, LSM10,*	11	*CSF3R*	Oncogene	Unknown
chr1:37,940,154-40,539,413	AI	2,599,260	p34.3 - p34.2	23.68	198	hsa-mir-558	2	*AB075489, AK304759, AKIRIN1,*	55	*MYCL*	Oncogene	Unknown
chr1:39,412,023-39,710,788	Gain	298,766	p34.3	6.10	56		0	*AKIRIN1, MACF1, Mir_562, ND*	4			
chr1:40,539,413-40,869,908	Gain	330,496	p34.2	58.30	137		0	*COL9A2, PPT1, RLF, SMAP2, T*	6			
chr1:40,882,598-40,980,681	Gain	98,084	p34.2	5.98	57		0	*EXO5, SMAP2, ZFP69, ZFP69B*	4			
chr1:40,981,271-41,089,463	Gain	108,193	p34.2	6.85	32		0	*EXO5, RIMS3, ZNF684*	3			
chr1:40,981,271-41,347,650	AI	366,380	p34.2	3.17	33	hsa-mir-30c	2	*CITED4, EXO5, KCNQ4, LOC10*	10			
chr1:43,608,641-43,854,818	AI	246,178	p34.2	2.06	30		0	*AK309744, C1orf210, CDC20, C*	15	*MPL*	Oncogene	Unknown
chr2:179,498,140-179,539,809	Loss	41,670	q31.2	100.00	29		0	*MIR548N, TTN*	2			
chr2:179,613,414-179,616,057	Loss	2,644	q31.2	100.00	29		0	*TTN*	1			
chr4:7,435,824-7,437,211	Loss	1,388	p16.1	0.00	16		0	*PSAPL1, SORCS2*	2			
chr4:39,604,400-39,838,356	Gain	233,957	p14	11.77	54		0	*BC040333, PDS5A, SMIM14, UB*	4			
chr6:61,000,000-62,229,529	Gain	1,229,530	q11.1	28.41	12		0		0			
chr7:38,307,524-38,342,505	Gain	34,982	p14.1	100.00	15		0	*AK096766, TARP, TCRGC2, TCR*	5			
chr7:48,315,169-48,318,967	Loss	3,799	p12.3	0.00	32		0	*ABCA13, AX746840*	2			
chr7:61,074,194-62,395,530	Gain	1,321,337	q11.1 - q11.21	99.61	21		0		0			
chr9:17,576,554-17,849,639	AI	273,086	p22.2	100.00	14		0	*SH3GL2*	1			
chr9:122,278,536-122,391,755	Loss	113,220	q33.1	30.35	30		0		0			
chr9:122,763,700-122,904,242	Loss	140,543	q33.2	0.00	45		0		0			
chr11:50,472,623-51,525,878	Gain	1,053,256	p11.12	100.00	15		0	*OR4A5, OR4C46, TRNA_Lys*	3			
chr12:37,876,400-38,255,277	Gain	378,878	q11 - q12	100.00	14		0		0			
chr12:52,961,642-52,967,401	Loss	5,760	q13.13	29.22	33		0	*KRT74*	1			
chr14:22,718,405-22,953,551	Gain	235,147	q11.2	100.00	99		0	*AK093552, AK125397, AV2S1A1,*	23			
chr14:35,307,619-35,344,875	Gain	37,257	q13.2	40.58	9		0	*BAZ1A*	1			
chr17:29,873,697-30,587,181	Gain	713,485	q11.2	47.02	184	hsa-mir-365	3	*ARGFXP2, COPRS, JB074907, L*	14	*SUZ12*	Oncogene/TSG	Yes
chr17:30,652,705-30,789,283	Gain	136,579	q11.2	0.22	26	hsa-mir-632	1	*C17orf75, MIR632, PSMD11, ZN*	4			
chr17:30,789,283-33,816,223	AI	3,026,941	q11.2 - q12	12.61	182		0	*AA06, AK057317, AK307275, AS*	34			
chr17:32,340,948-32,942,574	Loss	601,627	q12	10.69	290		0	*ASIC2, BC062794, C17orf102, C*	11			
chr17:33,917,012-34,051,150	Gain	134,139	q12	9.84	16		0	*AP2B1*	1			
chr17:34,051,150-35,632,081	AI	1,580,932	q12	85.82	73	hsa-mir-290	1	*7SK, AATF, ACACA, AP2B1, AX7*	51	*TAF15*	Oncogene	Unknown
chr17:35,632,081-35,819,506	Gain	187,426	q12	100.00	33		0	*ACACA, C17orf78, TADA2A*	3			

chr17:35,917,877-36,031,415	Gain	113,539	q12	100.00	49	hsa-mir-378	1	*DDX52, MIR378J, SYNRG*	3			
chr17:35,917,877-36,137,066	AI	219,190	q12	100.00	22	hsa-mir-378	1	*DDX52, HNF1B, MIR378J, SYNR*	4			
chr17:36,137,066-36,179,875	Gain	42,810	q12	100.00	14		0		0			
chr17:36,179,875-37,223,923	AI	1,044,049	q12	56.92	53	hsa-mir-472	3	*AL832615, ARHGAP23, C17orf9*	42	*MLLT6, LASP1*	Fusion, Fusion	Unknown, Unknown
chr17:37,066,637-37,223,923	Gain	157,287	q12	1.17	62		0	*FBXO47, LASP1, LINC00672, LC*	7	*LASP1*	Fusion	Unknown
chr17:37,297,287-37,319,084	Gain	21,798	q12	0.00	11		0	*ARL5C, PLXDC1*	2			
chr17:37,342,709-37,557,267	Gain	214,559	q12	62.49	46		0	*CACNB1, FBXL20, RPL19, STAC*	4			
chr17:37,570,588-37,672,348	Gain	101,761	q12	2.77	24		0	*CDK12, MED1*	2	*CDK12*	TSG	No
chr17:37,672,348-38,254,073	AI	581,726	q12 - q21.1	19.84	54	hsa-mir-472	1	*CDK12, CSF3, ERBB2, GRB7, G*	25	*CDK12, ERBB2*	TSG, Oncogene	Unknown, Unknown
chr17:38,254,073-38,303,951	Gain	49,879	q21.1	0.00	13		0	*CASC3, DKFZp586B1922, MSL1,*	5			
chr17:38,398,281-38,483,714	Gain	85,434	q21.1 - q21.2	7.91	29		0	*CDC6, RARA, WIPF2*	3	*RARA*	Oncogene	Yes
chr17:38,483,714-39,059,866	Loss	576,153	q21.2	21.22	317		0	*CCR7, GJD3, IGFBP4, KRT10, K*	18	*RARA, SMARCE1*	Oncogene, TSG	No, Yes
chr17:39,059,866-39,593,520	AI	533,655	q21.2	71.71	23		0	*KRT23, KRT31, KRT33A, KRT33*	43			
chr17:39,623,397-40,572,713	AI	949,317	q21.2	34.17	66	hsa-mir-651	1	*ACLY, AK024535, AK090604, AK*	45	*STAT5B, STAT3*	Oncogene, Oncogene	Unknown
chr17:40,572,713-40,610,086	Gain	37,374	q21.2	0.00	17		0	*PTRF*	1			
chr17:40,694,197-40,716,961	Gain	22,765	q21.2	73.84	29		0	*BC043620, COASY, HSD17B1, N*	4			
chr17:40,865,367-40,931,054	Gain	65,688	q21.2 - q21.31	0.00	23		0	*BC047651, EZH1, RAMP2, RAM*	5	*EZH1*	PRC2 complex	Yes
chr17:41,170,205-41,569,311	Gain	399,107	q21.31	33.72	157	hsa-mir-211	1	*AK027091, AK093551, AK31113*	15	*BRCA1, ETV4*	TSG, Oncogene	No, Yes
chr17:42,483,189-42,643,635	Gain	160,447	q21.31	8.08	23		0	*FZD2, GPATCH8*	2			
chr17:42,643,635-42,841,449	Loss	197,815	q21.31	69.40	65		0	*ADAM11, C17orf104, CCDC43, I*	5			
chr17:42,877,811-43,373,464	Loss	495,654	q21.31	47.90	253		0	*ACBD4, AK124465, BC045769, C*	23			
chr17:43,472,805-45,234,631	Loss	1,761,827	q21.31 - q21.32	100.00	393	hsa-mir-431	2	*AK057897, ARHGAP27, ARL17,*	40			
chr17:45,352,153-45,462,701	Loss	110,549	q21.32	64.11	65		0	*AX748120, EFCAB13, ITGB3, LC*	4			
chr17:57,785,690-57,856,830	Gain	71,141	q23.1	0.00	14		0	*VMP1*	1			
chr18:18,570,235-18,751,151	Gain	180,917	q11.1	3.32	12		0	*ROCK1*	1			
chr19:12,186,566-12,192,228	Gain	5,663	p13.2	0.00	10		0	*ZNF844*	1			
chr19:12,444,164-12,461,889	Gain	17,726	p13.2	0.00	10		0	*ZNF442, ZNF563*	2			
chr19:36,826,837-37,038,568	Gain	211,732	q13.12	18.59	59		0	*LOC644189, LOC728752, ZFP14*	7			
chr20:54,041,419-54,158,579	Loss	117,161	q13.2	2.11	65		0	*LINC01440, LINC01441*	2			
chr22:25,897,536-27,034,913	Loss	1,137,378	q11.23 - q12.1	53.92	663	hsa-mir-548	1	*ADRBK2, ASPHD2, CRYBA4, CR*	12			
chr22:27,072,717-29,125,669	Loss	2,052,953	q12.1	28.12	815	hsa-mir-573	3	*AK026502, AK055980, AX748308*	15			
chr22:29,182,334-31,486,489	Loss	2,304,156	q12.1 - q12.2	34.42	947	hsa-mir-365	2	*AP1B1, ASCC2, C22orf31, CABP*	55			
chr22:31,514,266-31,533,966	Loss	19,701	q12.2	100.00	39		0	*INPP5J, PLA2G3*	2			
chr22:31,924,763-31,977,167	Loss	52,405	q12.2	77.01	29		0	*SFI1*	1			
chr22:32,188,783-35,982,736	Loss	3,793,954	q12.2 - q12.3	22.29	1514	hsa-mir-476	3	*5S_rRNA, AK123891, AP1B1P1,*	33			
chr22:36,042,642-36,700,131	Loss	657,490	q12.3	41.52	302		0	*APOL1, APOL2, APOL3, APOL4,*	10			
chr22:36,941,694-38,109,336	Loss	1,167,643	q12.3 - q13.1	91.40	750		0	*AK097787, AK097791, AK123632*	85			
chr22:38,151,247-38,616,133	Loss	464,887	q13.1	74.76	244	hsa-mir-659	3	*AK098727, ANKRD54, BAIAP2L2*	23			
chr22:38,662,192-39,392,388	Loss	730,197	q13.1	49.57	246		0	*APOBEC3A, APOBEC3A_B, APC*	21			
chr22:39,440,420-39,527,598	Loss	87,179	q13.1	4.01	62		0	*APOBEC3F, APOBEC3G, APOB*	4			
chr22:39,815,183-40,814,665	Loss	999,483	q13.1	19.61	327		0	*ADSL, ATF4, CACNA1I, DQ5896*	18			
chr22:42,075,539-42,676,097	Loss	600,559	q13.2	10.96	269	hsa-mir-378	2	*C22orf46, CCDC134, CENPM, C*	27			
chr22:42,753,900-42,981,017	Loss	227,118	q13.2	100.00	71		0	*BC038245, LINC01315, NFAM1,*	8			
chr22:43,167,430-50,662,848	Loss	7,495,419	q13.2 - q13.33	48.95	3984	hsa-let-7a-3	9	*AK056490, AK093107, AK095988*	108			
chr22:50,722,105-50,819,715	Loss	97,611	q13.33	36.95	37		0	*DENND6B, PLXNB2, PPP6R2*	3			
chr22:50,919,714-50,943,294	Loss	23,581	q13.33	100.00	50		0	*ADM2, LMF2, MIOX*	3			
chr22:50,950,090-51,304,566	Loss	354,477	q13.33	77.60	200		0	*ACR, ARSA, BC048192, BC05034*	18			
chrX:740,019-1,402,609	Gain	662,591	p22.33	77.23	45		0	*CRLF2, CSF2RA, DQ576039*	3	*CRLF2*	Oncogene	Yes
chrX:12,201,134-12,233,556	Loss	32,423	p22.2	28.65	11		0	*FRMPD4*	1			
chrX:23,815,820-23,928,519	Gain	112,700	p22.11	0.00	23		0	*APOO, CXorf58, RPL9*	3			
chrX:24,073,875-24,193,755	Gain	119,881	p22.11	46.07	16		0	*EIF2S3, ZFX, ZFX-AS1*	3			
chrX:150,830,685-150,840,724	Loss	10,040	q28	44.90	18		0	*PASD1*	1			
